# Different PfEMP1-expressing *Plasmodium falciparum* variants induce divergent endothelial transcriptional responses during co-culture

**DOI:** 10.1371/journal.pone.0295053

**Published:** 2023-11-30

**Authors:** Basim Othman, Leo Zeef, Tadge Szestak, Zineb Rchiad, Janet Storm, Caroline Askonas, Rohit Satyam, Aymen Madkhali, Michael Haley, Simon Wagstaff, Kevin Couper, Arnab Pain, Alister Craig

**Affiliations:** 1 Tropical Disease Biology, Liverpool School of Tropical Medicine, Pembroke Place, Liverpool, United Kingdom; 2 Faculty of Biology, Medicine and Health, The Lydia Becker Institute of Immunology and Inflammation, University of Manchester, Manchester, United Kingdom; 3 Pathogen Genomics Laboratory, Bioscience Program, Biological and Environmental Sciences and Engineering (BESE) Division, King Abdullah University of Science and Technology, Thuwal, KSA; Cochin Institute: Institut Cochin, FRANCE

## Abstract

The human malaria parasite *Plasmodium falciparum* is responsible for the majority of mortality and morbidity caused by malaria infection and differs from other human malaria species in the degree of accumulation of parasite-infected red blood cells in the microvasculature, known as cytoadherence or sequestration. In *P*. *falciparum*, cytoadherence is mediated by a protein called PfEMP1 which, due to its exposure to the host immune system, undergoes antigenic variation resulting in the expression of different PfEMP1 variants on the infected erythrocyte membrane. These PfEMP1s contain various combinations of adhesive domains, which allow for the differential engagement of a repertoire of endothelial receptors on the host microvasculature, with specific receptor usage associated with severe disease. We used a co-culture model of cytoadherence incubating human brain microvascular endothelial cells with erythrocytes infected with two parasite lines expressing different PfEMP1s that demonstrate different binding profiles to vascular endothelium. We determined the transcriptional profile of human brain microvascular endothelial cells (HBMEC) following different incubation periods with infected erythrocytes, identifying different transcriptional profiles of pathways previously found to be involved in the pathology of severe malaria, such as inflammation, apoptosis and barrier integrity, induced by the two PfEMP1 variants.

## Introduction

The human malaria parasite *Plasmodium falciparum* modifies the host erythrocyte membrane [1], inserting several parasite-derived proteins including the major variant surface antigen *Plasmodium falciparum* erythrocyte membrane protein-1 (PfEMP1). This modification supports the binding of *P*. *falciparum*-infected erythrocytes (Pf-IE) to microvascular endothelial cells and is thought to have evolved to prevent the passage and clearance of rigid infected erythrocytes by the spleen. The molecular basis of adhesion has been studied in some detail [2] and a broad range of host receptors have been identified for cytoadherence, as well as other Pf-IE/ host cell interactions (rosetting and clumping) (see S1 Fig for a summary of host interactions).

**Fig 1:**
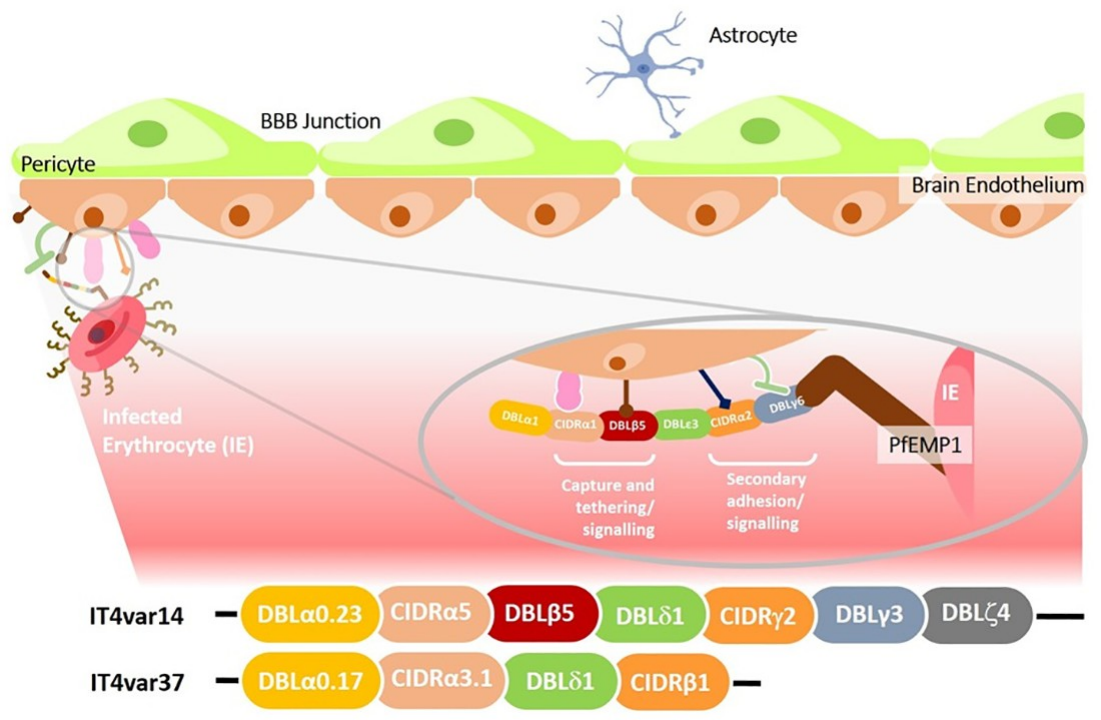
Schematic diagram of a typical PfEMP1 structure interacting with host endothelial receptors. The multi-domain structure of PfEMP1 may facilitate interactions that enhance capture and binding of Pf-IE from flow, as well as other interactions involved in signalling that occur once the Pf-IE have been bound, however the involvement of multiple interactions along these pathways is largely hypothetical, with limited data to support it. The lower section shows the domain content (adapted from [10]) of the expressed PfEMP1s of the IT4var14 and IT4var37 isolates used in this study. The var domain structures were determined using the VarDom server [11].

PfEMP1 is expressed on the infected erythrocyte surface and recognised by the host immune system resulting in the generation of antibodies that control parasite replication. To allow for parasite persistence in the host, a system of antigenic variation has been developed in *P*. *falciparum* that, for PfEMP1, relies on the mutually exclusive expression of a single *var* gene from a pool of approximately 60 per parasite genome (reviewed in [3]). The overall structure of PfEMP1 is preserved across the protein family, with an extracellular region comprised of varying numbers of Duffy-binding like domains (DBL) and Cysteine-rich interspersed domain regions (CIDR) attached to a conserved intracellular region (Fig 1). The specific types of DBL and CIDR determine the binding repertoire of the infected erythrocyte, and although the range of receptors that PfEMP1 can bind to is large, the actual repertoire per PfEMP1 is restricted. The *var* genes that encode PfEMP1 have been classified into three major groups, A, B and C, with some genes spanning the boundaries between A/B and B/C and a small number of specialised var genes (e.g., *var2csa*) [4]. In addition to this classification, *var* genes are also grouped into ‘long’ and ‘short’ forms [5], represented by the PfEMP1 variants selected for this study–IT4var14 (group B, long) and IT4var37 (group C, short) (Fig 1). IT4var14 binds strongly to tumour necrosis factor (TNF)-activated human brain microvascular endothelial cells (HBMEC) via ICAM-1 as well as to other endothelia (human umbilical vein endothelial cells (HUVEC) & human dermal microvascular endothelial cells (HDMEC)), the latter including interaction via CD36, whereas IT4var37 demonstrates strong binding to HDMEC via CD36, but weak or no binding to HBMEC and HUVEC, that do not express CD36 (S2 Fig (see Materials and methods section) and [6, 7]). Group C variants, represented here by IT4var37, have been associated with chronic infection and uncomplicated/ asymptomatic malaria [8]. Longer-form PfEMP1s are seen more frequently in severe disease, although the association with cerebral malaria is most clearly observed with endothelial protein C receptor (EPCR)-binding variants [9] of Groups A or B/A rather than the group B (IT4var14) and group C (IT4var37) variants represented in this study.

The multi-domain structure of PfEMP1 allows infected erythrocytes to bind to multiple receptors on the host cell surface, but the avidity of binding varies widely, which has prompted the hypothesis that some interactions are designed to capture Pf-IE from blood flow (e.g., ICAM-1) or to tether Pf-IE to endothelium (e.g., CD36) [12]. In some cases, primary adhesion may facilitate lower avidity secondary interactions to take place once the Pf-IE and host endothelial cells are adjacent. This mechanism has been suggested to explain some behaviours seen with some EPCR binding Pf-IE, with the hypothesis of ICAM-1/ EPCR dual binding required to produce efficient adhesion and loss of barrier function [13, 14]. However, evidence for a direct link between these multi-receptor interactions and malaria pathology shown in Fig 1 is indirect and limited. Loss of endothelial barrier function is thought to be particularly important in cerebral malaria as disruption of the blood brain barrier causes swelling in the brain, causing cerebral displacement onto the top of the spinal cord and respiratory arrest [15].

The pathogenesis of cerebral malaria is multifactorial (for reviews see [16, 17]), depending on a range of factors such as reduction in microvascular perfusion and activation of endothelium [18]. Endothelial activation is thought to occur through the release of parasite-derived material acting locally on endothelial cells during sequestration, such as heme, *P*. *falciparum* histidine-rich protein-2 (PfHRP2) [19, 20], histones [21, 22] and extracellular vesicles [23] or via Pf-IE signalling induced directly by ligand-receptor binding to the microvasculature endothelium [24–26]. To investigate the specific effects of Pf-IE cytoadherence on endothelial cell activity we have used a co-culture model of non-activated and TNF-activated HBMEC and Pf-IE using two PfEMP1 variants from the same parental background (IT4var14 and IT4var37, Fig 1) but with different adhesion profiles. We used RNAseq approaches to analyse endothelial cell transcriptional changes over time and have shown that different PfEMP1 variants induce different patterns of endothelial responses, including pathways previously associated with the pathology of severe disease.

## Results

### Effects of Pf-IE and RBC co-culture on HBMEC

**Fig 2:**
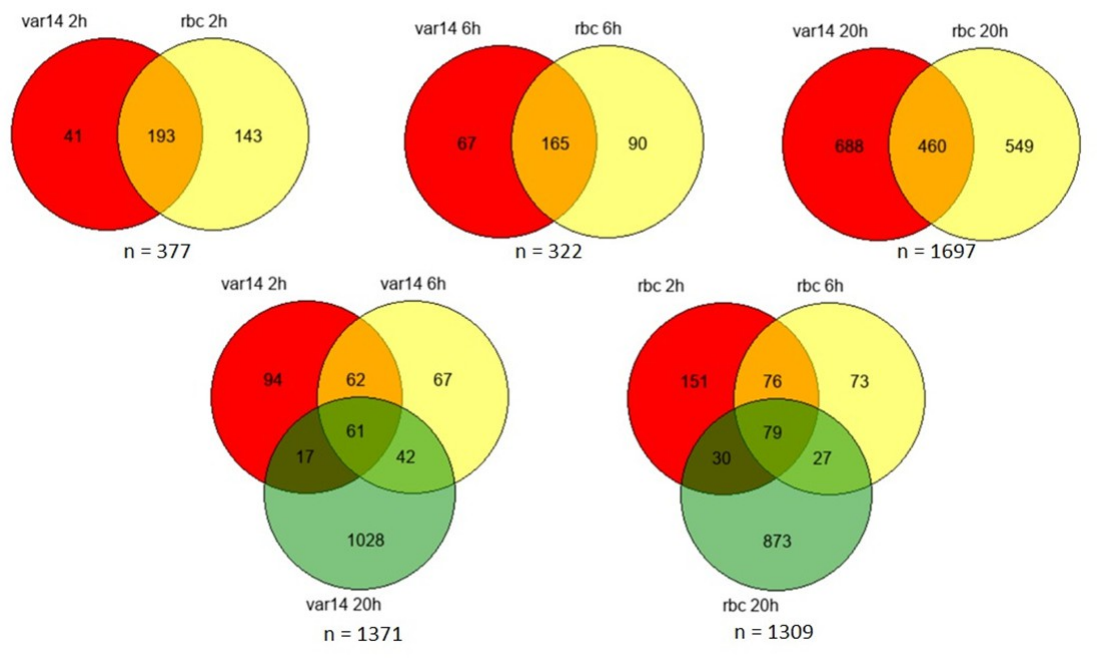
Venn diagrams showing the relationship between differentially expressed genes (DEGs). DEGs induced in non-activated (no TNF) HBMEC by Pf-IE (IT4var14) and RBC over time in co-culture. Data from S1 File using Padj<0.05 & transcriptional changes (up or down) greater than 2-fold.

**Fig 3:**
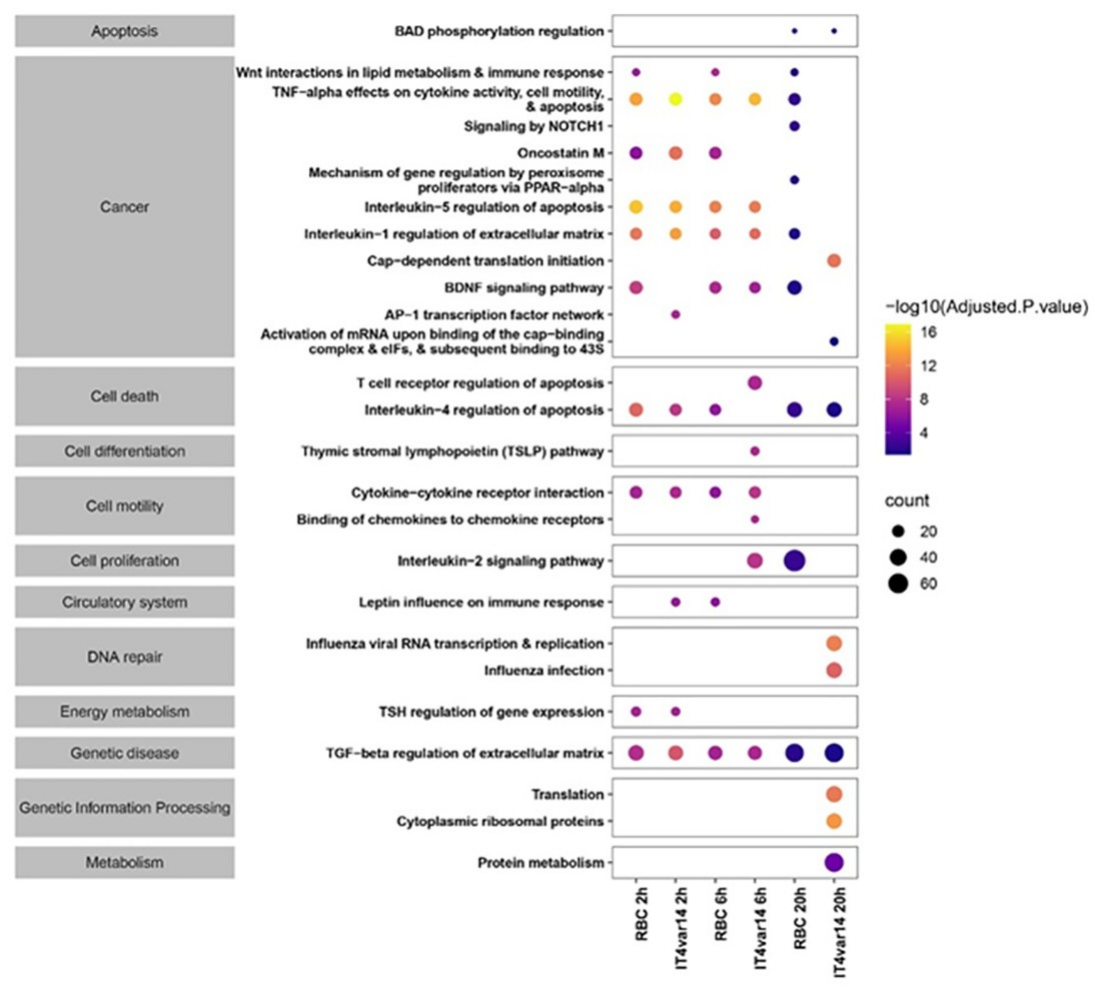
Presence-absence bubble plot for the top 26 pathways perturbed in a time course co-culture experiment between non-activated (no TNF) HBMEC and IT4var14 and RBC. The size and colour of the bubbles represent the gene counts (number of genes associated with the pathway) and their statistical significance (-log10(Adjusted p-value)). The broad categorization of pathways was obtained from Bioplanet [31].

Several groups have shown that co-culture of endothelial cells with infected and uninfected erythrocytes results in changes in their gene expression profiles [25, 27–30]. As a baseline for our study, we were interested in how HBMEC (without TNF activation) would react to the presence of Pf-IE and RBC over time, particularly whether these responses would cause overlapping responses and how much would be specific to each condition. A schematic overview of the experimental design is presented in Fig 11 as part of the Materials and methods section. Fig 2 and S3 Fig plus the analyses contained in supplementary material (S1 & S2 Files (R2 analyses with p<0.05 and fold change >2)) confirms the findings of earlier studies, that transcription in endothelial cells is modified by the presence of both Pf-IE and RBC and extends these observations by showing in parallel experiments that the repertoires of DEGs induced by both Pf-IE and RBC overlap significantly, which is not unexpected given the presence of the malaria parasite within the erythrocyte. What is perhaps unexpected is the major component of DEGs that can be assigned to RBC alone, and not seen in Pf-IE co-culture, although it has been known for some time that RBC can induce significant changes in endothelium associated with disease [30]. An analysis of the major pathways identified from these results is presented in Fig 3 and supplementary material (S3 File) identifying several interesting pathways including one already associated with malaria infection and others involved in apoptosis, cytokine interactions and Wnt regulated pathways. It is notable that there is considerable overlap of the top 20 pathways identified by HBMEC co-culture with Pf-IE (IT4var14) and RBC, particularly at 2 and 6 hours of co-culture with 15/20 and 16/20 categories in common respectively, which is reduced to 6/20 at 20 hours. This implies that the apposition of the RBC to EC during cytoadherence has a significant influence on the response of endothelial cells as well as effects caused directly by parasite-derived proteins and modifications.

### Analysis framework for HBMEC + TNF RNAseq data

To replicate the pro-inflammatory conditions of cytoadherence seen during malaria infection, we used TNF-activated HBMEC. Initial PCA analyses of the time course experiments (S4 Fig) show clear differential biological effects induced by co-culture with RBC, IT4var14 and IT4var37. As expected, the PCA indicated a temporal transcriptional response of the ECs within the experiments. This was most pronounced for the IT4var37, which showed a clear stepwise change at 6h and 20h from the 0h samples. Interestingly, the RBC and IT4var14 6h and 20h samples exhibited a similar transcriptional deviation from the 0h starting point, but this was stronger within the IT4var37 samples.

In the PCA plot for the samples using TNF-activated HBMEC, we also observed noticeable variation within the 0h time points from the different time course experiments, in particular, 0h from the IT4var37 experiment compared with the IT4var14 and RBC samples (S4 Fig). As these 0h samples should theoretically be the same (the endothelial cells underwent comparable culture and activation conditions to that point), this likely reflects experimental variation due to the different times at which the co-cultures were performed, with IT4var14 and RBC experiments being conducted during the same period of 2–3 months and the IT4var37 co-cultures coming 5–6 months later over 2–3 weeks. Closer manual inspection of the RNAseq data showed that the TNF activation of the HBMEC had failed in two experiments, as shown by looking at genes known to be upregulated by TNF (ICAM-1 and VCAM-1), and only samples with validated TNF activation were used in the analyses.

**Fig 4:**
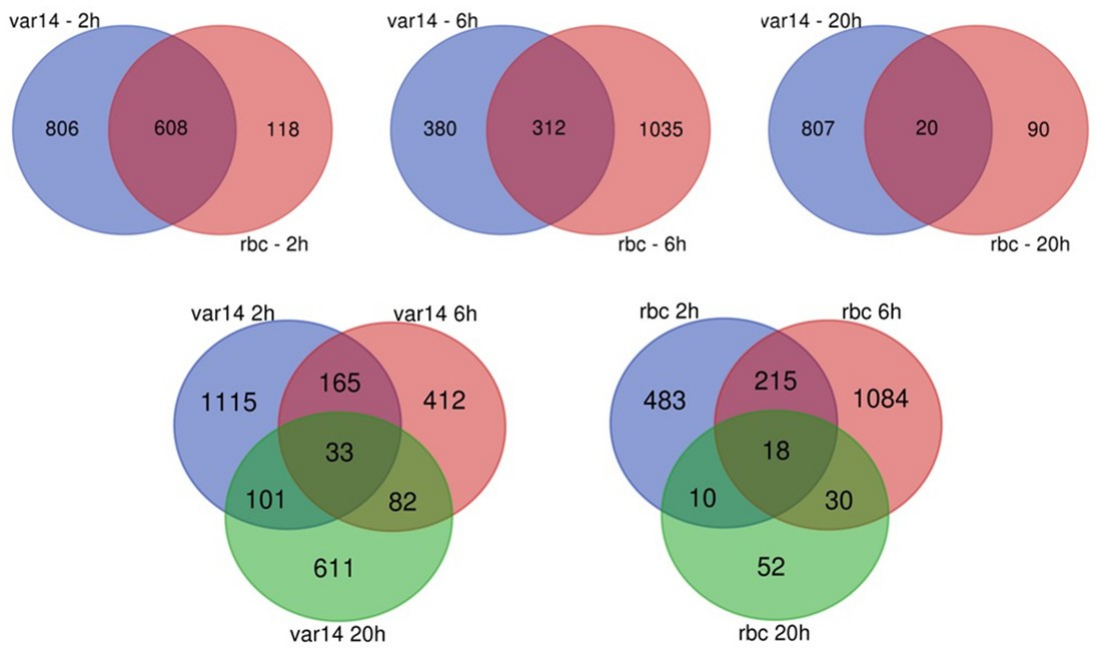
Venn diagrams showing the relationship between DEGs induced in TNF-activated HBMEC by Pf-IE (IT4var14) and RBC over time in co-culture. Data from S1 File using Padj<0.05 & transcriptional changes (up or down) greater than 2-fold.

The first phase of analysis was to look at the changes taking place during IT4var14 and RBC co-culture with TNF-activated HBMEC over time. Multiple interactions take place during co-culture (Fig 1 & S1 Fig), including contributions from the erythrocyte proteins brought into contact with HBMEC in addition to parasite-derived antigens, as well as the residual effects on transcription of the TNF treatment used to activate the endothelial cells prior to co-culture. Thus, both RBC proteins and systemic TNF contribute to host endothelial effects during malaria infection. Using the 0 hours co-culture condition as a baseline, Fig 4 shows extensive changes in transcription during HBMEC co-culture with RBC and IT4var14, with greater overlap between these conditions at 2 hours, decreasing progressively over 6 and 20 hours. An interesting observation to come from our experiments is the comparison of the DEG profiles for IT4var14/HBMEC with and without TNF activation of the endothelial cells (S5 Fig). Our results show that TNF has a major role in shaping the endothelial response to Pf-IE co-culture, with a small proportion of DEGs being independent of TNF status (3–4%), but the majority being specific to the TNF activation status of the HBMEC used in co-culture, and is consistent with results from other studies showing different activation profiles in brain microvascular endothelium caused by TNF and Pf-IE material [32].

**Fig 5:**
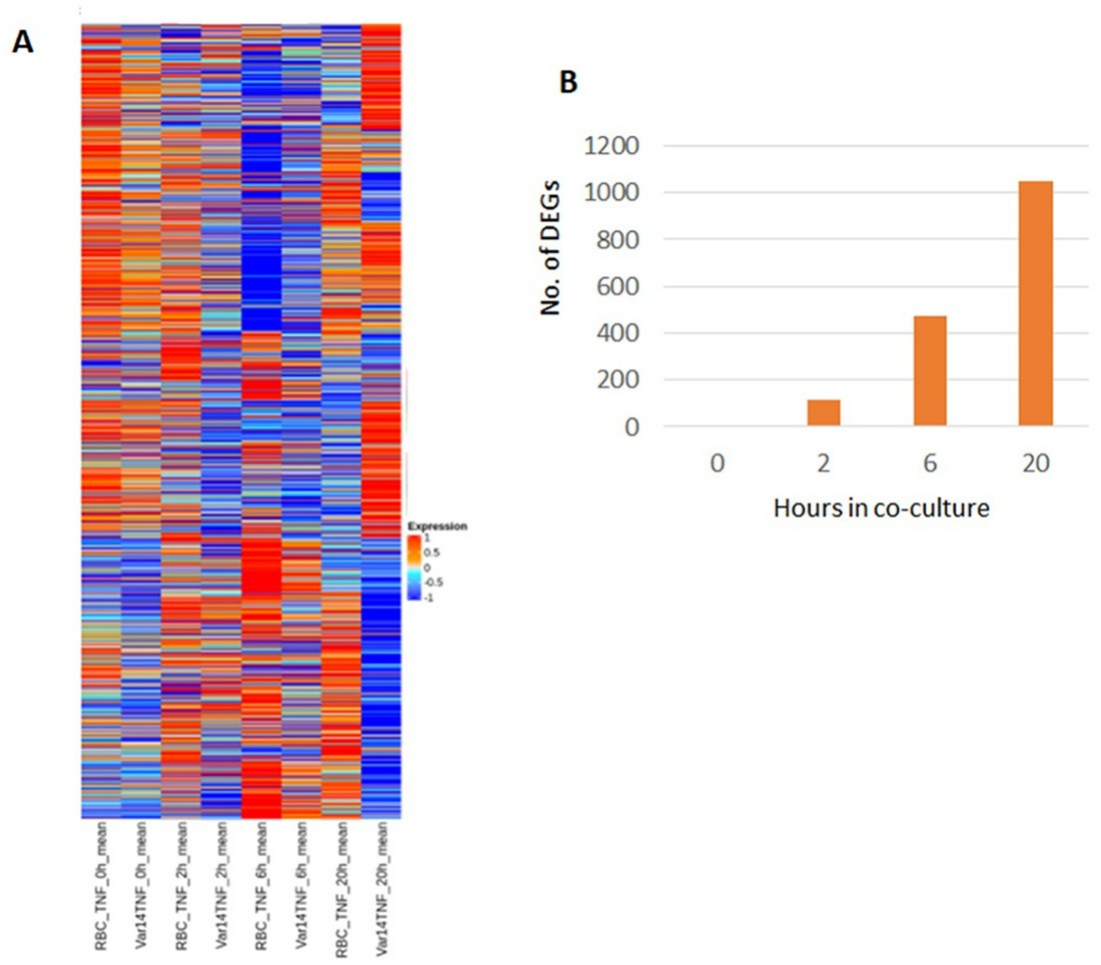
Gene expression heatmaps. (A) Heatmaps of gene expression obtained from RBC and IT4var14 co-culture with TNF-activated HBMEC at different time points and (B) a graph showing the number of DEGs identified over time in co-culture (see S4 File–‘R5’ analysis for details).

The patterns of expression obtained with Pf-IE and RBC co-culture is shown as a heatmap in Fig 5. These demonstrate RBC induce transcriptional changes in endothelial cells, and that the patterns from this overlap with those for IT4var14 at 0 h, consistent with the PCA (S4 Fig), and diverge at later time points. To control for the effects of TNF and RBC, and focus on parasite-derived effects, the analysis compared infected erythrocyte (IT4var14) co-culture samples with corresponding uninfected erythrocyte (RBC) co-cultures to produce a time-course of parasite-induced transcriptional changes during co-culture with TNF-activated HBMEC (see S4 File–the data used in this manuscript comes from the R5 analysis with p<0.05 and fold change >2). Comparison across these time course experiments was possible as the expression profiles for the 0 hours conditions were similar, with only 1 identified DEG (Fig 5 & S6 Fig).

**Fig 6:**
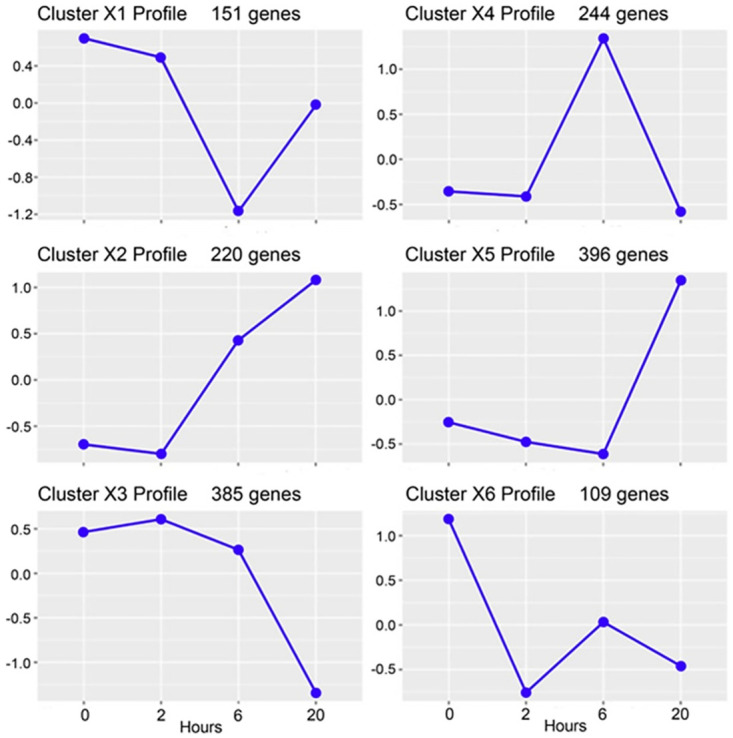
Cluster profiles of DEGs during IT4var14 co-culture with TNF-activated HBMEC after removal of corresponding RBC responses. The y-axis indicates log(fold change)–see ‘R5’ analysis in S4 File for details.

The expression patterns are dynamic, altering significantly during the experiment, with little overlap between gene lists (adjusted p-value < 0.05) at the different time points beyond 0 h (S6 Fig). The number of DEGs identified increases with time, indicating continuing crosstalk between the Pf-IE and endothelium and the release of internal parasite-derived mediators (such as heme; PfHRP2) at the 20 h stage due to Pf-IE lysis. The analysis using DEG lists with RBC data subtracted generated six clusters exhibiting varied expression profiles over time (Fig 6). The genes in these clusters have been analysed using the Enrichr platform (https://maayanlab.cloud/Enrichr/) [33–35] using BioPlanet_2019 [31], KEGG_2021 [36, 37] and WikiPathway_2021 [38] for preferential representation of human pathways (see S5–S7 Files). Table 1 shows the pathways identified using BioPlanet 2019 with Padj<0.05, which include regulation of apoptosis, cytokine signalling networks, Notch signalling (involved in vascular regulation/ repair) and a Syndecan-1 pathway. Gene Ontology (GO) analyses identified a range of biological functions associated with IT4var14 co-culture, in particular signalling pathways involved in cell adhesion, endothelial cell remodelling and inflammation, including CXCL10, CLDN4, HGF, SOD2, ADAMTS1, TGFB2, BMP4, JUN and JAG2.

**Table 1:** Biological pathways identified from co-culture between TNF-activated HBMEC and IT4var14.

**Cluster X1**				
**Term**	**Overlap**	**P-value**	**Adjusted P-value**	**Genes**
Interleukin-1 regulation of extracellular matrix	11/120	1.80E-09	7.06E-07	BMP4;GREM1;BMP2;CXCL8;SERPINB2; CCL20;ALDH1A1;LIF;INHBA;SOD2;BIRC3
Interleukin-4 regulation of apoptosis	11/267	6.03E-06	0.00118	BMP4;POSTN;BMP2;VCAN;CXCL8; CLEC2B;CITED2;CCL20;INHBA;LITAF;EGFR
Cytokine-cytokine receptor interaction	10/265	3.43E-05	0.00447	BMP2;IL18RAP;CXCL8;TSLP;CCL20;HGF; LIF;LTA;INHBA;EGFR
Nitric oxide stimulation of guanylate cyclase	4/28	5.55E-05	0.00543	GUCY1A3;PDE2A;PDE5A;PDE9A
TGF-beta regulation of extracellular matrix	14/565	9.99E-05	0.00734	POSTN;SYNM;CITED2;HGF;INHBA;CLDN1;BMP4;ALDH1A3;VCAN;CSRP2;CLEC2B;HAS2;PDE5A;MAP3K5
Interleukin-5 regulation of apoptosis	7/144	1.13E-04	0.00735	CXCL8;CLEC2B;HAS2;INHBA;SGK1;SOD2;BIRC3
Hyaluronan metabolism	3/14	1.44E-04	0.00807	STAB2;HAS2;LYVE1
Gastrin pathway	4/44	3.35E-04	0.01638	CXCL8;SERPINB2;HGF;BIRC3
Syndecan 1 pathway	4/46	3.98E-04	0.01730	COL17A1;COL3A1;HGF;COL11A2
TNF-alpha effects on cytokine activity, cell motility, and apoptosis	6/135	5.66E-04	0.02159	TNFAIP8;CXCL8;HGF;INHBA;SOD2;BIRC3
Oncostatin M	9/311	6.07E-04	0.02159	SYNM;CXCL8;SERPINB2;CCL20;HGF; CDH11;NEDD4L;SOD2;EGFR
Glycosaminoglycan metabolism	5/110	0.00148719	0.04672	VCAN;STAB2;BGN;HAS2;LYVE1
Purine metabolism	6/164	0.00155328	0.04672	GUCY1A3;ADCY10;PDE2A;PDE5A;PDE9A;ADA
				
**Cluster X2**				
**Term**	**Overlap**	**P-value**	**Adjusted P-value**	**Genes**
Nitric oxide effects	3/9	1.05E-04	0.05094	NOS2;NOS3;HBA2
				
**Cluster X3**				
none				
				
**Cluster X4**				
**Term**	**Overlap**	**P-value**	**Adjusted P-value**	**Genes**
Cytokine-cytokine receptor interaction	13/265	2.41E-05	0.00958	CSF3;CXCR6;IL2RG;CXCL5;EPOR;TNFSF13B;CXCL11;CCL8;CCL5;KIT;KDR;IL12RB1;TNFRSF4
Interleukin-1 regulation of extracellular matrix	8/120	1.14E-04	0.02188	C3;CSF3;CCL8;FABP5;C1R;CCL5;CXCL5; DCN
GPCR ligand binding	15/410	1.65E-04	0.02188	GPR39;FZD2;GPR68;CXCR6;CXCL5;C3; CXCL11;P2RY6;ADORA3;CCL5;C3AR1; BDKRB2;F2RL1;WNT3;F2RL3
Activated NOTCH1 signalling in the nucleus	4/31	5.25E-04	0.03425	DLL4;JAG2;DTX4;DLL1
Signalling by NOTCH2	3/14	5.91E-04	0.03425	JAG2;DLL4;DLL1
Initiation of the second proteolytic cleavage of Notch receptor by receptor-ligand binding	3/14	5.91E-04	0.03425	JAG2;DLL4;DLL1
Peptide G-protein coupled receptors	9/192	6.04E-04	0.03425	C3;CXCL11;CCL5;C3AR1;BDKRB2;F2RL1;CXCR6;CXCL5;F2RL3
Other semaphorin interactions	3/16	8.93E-04	0.04432	SEMA4A;SEMA7A;ITGA1
				
**Cluster X5**				
**Term**	**Overlap**	**P-value**	**Adjusted P-value**	**Genes**
TGF-beta regulation of extracellular matrix	28/565	9.09E-06	0.00559	CAMK2B;CRABP2;STC1;PCSK6;ARHGAP6;SAT1;CYR61;SELENBP1;PTTG1; ADAMTS1;MYC;LEPR;PIM1;CKB;GBP2; MYOZ2;AKR1C1;F3;COL1A1;RCAN1; COL1A2;BFSP1;LOX;PSG4;CTH;PPARG; ANG;ATF3
TSH regulation of gene expression	9/97	1.31E-04	0.03458	NOTCH3;EGR1;NR4A3;ADAMTS1;MYC; RGS16;ID1;FOS;CKB
Oncostatin M	17/311	1.69E-04	0.03458	CRABP2;KLK1;AKR1C1;RGS16;KRT8; PLAT;F3;CYR61;COL1A1;SELENBP1; CLDN4;COL1A2;PTTG1;MYC;ALPL; PPARG;ATF3
				
**Cluster X6**				
**Term**	**Overlap**	**P-value**	**Adjusted P-value**	**Genes**
Oncostatin M	10/311	7.84E-06	0.00203	JUN;EDN1;SLCO2B1;KRT7;ITGB8;IGFBP6;ESR1;CTGF;MT1E;CDK5R1
TGF-beta regulation of extracellular matrix	13/565	1.25E-05	0.00203	TGFB2;EGR2;EDN1;PTGIS;SLC40A1; ZNF10;DKK1;CTGF;SLC7A5;SULT1E1; SNAI2;SLIT3;CDK5R1
BDNF signalling pathway	8/261	9.22E-05	0.00781	SLC7A5;JUN;EGR2;GPRC5B;FOSB;SLIT3;DKK1;GEM
HNF3A pathway	4/44	9.58E-05	0.00781	SHH;JUN;DSCAM;ESR1
TGF-beta signalling pathway	6/185	5.32E-04	0.02786	TGFB2;JUN;FST;FOSB;SIK1;CTGF
AP-1 transcription factor network	4/70	5.80E-04	0.02786	EDN1;JUN;FOSB;ESR1
FSH regulation of apoptosis	7/263	5.98E-04	0.02786	GPRC5B;BDNF;FST;IGFBP6;DKK1;ESR1; GEM

Biological pathways identified from the data from the analysis of IT4var14-specific DEGs, with RBC DEGs subtracted, produced on co-culture between TNF-activated HBMEC and IT4var14. The “Overlap” column refers to the number of DEGs discovered within specific pathways as a fraction of the genes mapped in that pathway.

### Differential transcription induced by PfEMP1 variants

**Fig 7:**
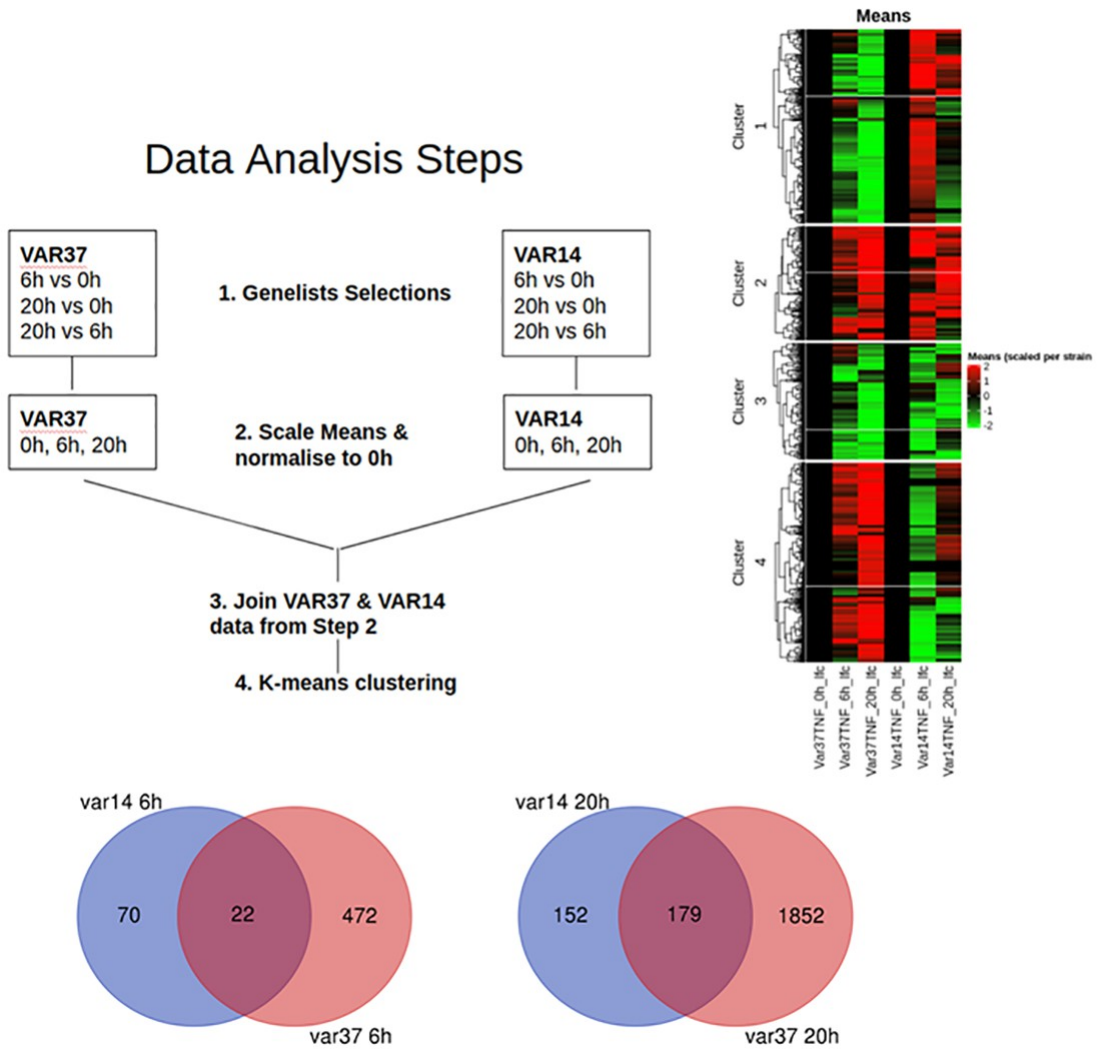
Analytical framework used to derive differentially expressed gene clusters. Analysis approach for HBMEC co-culture with IT4var14 and IT4var37 Pf-IE and Venn diagrams showing the relationship between DEGs induced in TNF-activated HBMEC by Pf-IE IT4var14 and IT4var37 over 6 and 20 hours in co-culture. Data from S5 File, with the removal of corresponding RBC responses.

To normalise and control for experimental variation, DEG lists were generated by comparison of changes in RNAseq data for each parasite variant at 6 h and 20 h compared to a baseline at 0 h (S8 File–R1&R4 analysis; p<0.05, fold change>2). The lists were compiled with an adjusted P-value < 0.05 and then sorted for changes in expression greater than two-fold. These gene lists were then compared between IT4var14 and IT4var37 to identify different patterns of transcription between these two parasite lines (Fig 7).

**Fig 8:**
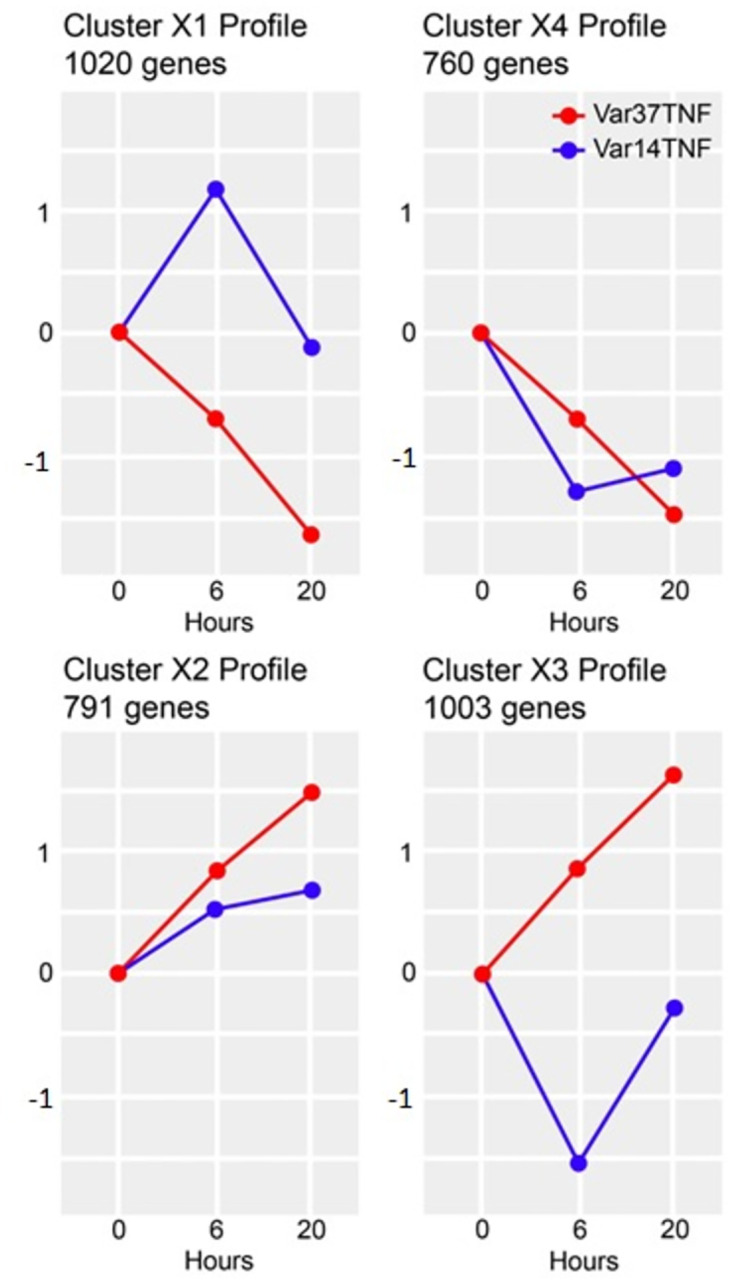
Transcription profiles of gene clusters showing differential expression between IT4var37 and IT4var14 co-culture with TNF-activated HBMEC. The y-axis indicates log(fold-change) and the x-axis the hours of co-culture. See S8 File for details.

**Fig 9:**
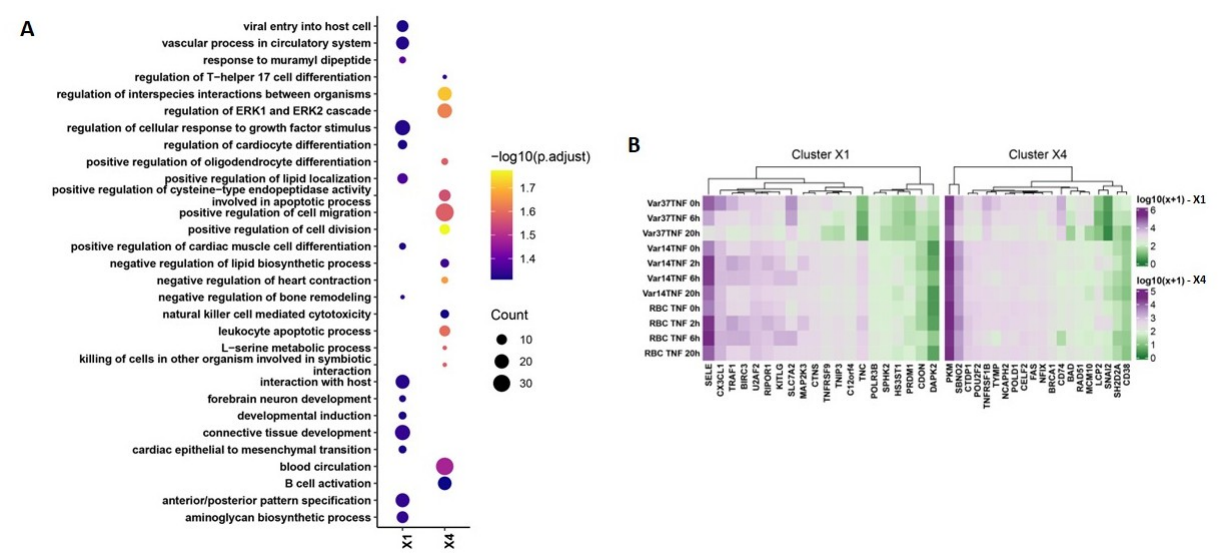
Analysis of differential expression between IT4var14 and IT4var37 experiments. (A) GO pathway analysis (Biological processes and molecular function) of the clusters showing differential patterns of expression in IT4var14 and IT4var37 co-culture with HBMEC (S10 File). The outcome is represented as presence-absence bubble plot for perturbed pathways (top 15 enriched pathways obtained from cluster X1 and X4). The bubble size and colour respectively depict the gene counts (number of genes associated with pathway) and their statistical significance (-log10(Adjusted p-value)). (B)Heatmap of gene expression perturbation for 20 genes in cluster X1 and X4 each (top 10 Upregulated and 10 Downregulated). The expression values are plotted as mean of normalized counts followed by log10 transformation.

This analysis revealed major differences in the patterns of gene expression (clusters) produced on co-culture with HBMEC using the two parasite lines that differ solely in the PfEMP1 being expressed on the infected erythrocyte membrane surface (Fig 8). Pathway analyses of these clusters indicate major differences in inflammation/ immune activation and signalling induced by co-culture with different PfEMP1 variants (Fig 9 and S9 & S10 Files).

## Discussion

Initially seen as a passive process based on vessel blockage, the impact of cytoadherence on the pathology of severe malaria is now recognised as having both passive (microvascular occlusion) and active (endothelial inflammation) components, with several groups identifying host pathways differentially expressed due to the action of *P*. *falciparum* infected erythrocytes on human endothelium. These experiments have been conducted on a variety of endothelial cell types (cell lines and primary endothelium, as well as from different tissues) using different parasite variants and have identified changes in endothelial cell gene expression associated with exposure to infected erythrocytes. The models used to recapitulate cytoadherence interactions have also varied, ranging from simple culture monolayers under static conditions to sophisticated 3D-vessels under flow conditions. The actual physiological conditions mimicking the situation in cytoadherence foci in the microvasculature *in vivo* is a moot point, and all models probably have some relevance to the situation in cerebral malaria cases. For our work, we have chosen a relatively simple model that supports endothelial viability, based on the co-culture under static conditions of mature trophozoite stage *Plasmodium falciparum* variants with primary HBMEC, non-activated or activated with TNF. The latter condition is designed to replicate the systemic endothelial activation observed post-mortem in cases of cerebral malaria. The situation is complicated by the different *in vitro* culture conditions used for endothelium and parasites, and the system has been optimised to preserve the integrity of the endothelium. During co-culture we see some development of the infected erythrocytes, but this is slower than seen in standard parasite culture conditions. The parasite lines are both from the IT lineage, differing in the major *var* gene being transcribed, and represent very different adhesion characteristics. IT4var14 is a long-form PfEMP1 from the upsB class that binds strongly to HBMEC via ICAM-1; IT4var37 is an upsC (short-form) PfEMP1 showing no/very low binding to HBMEC, but strong adhesion to CD36-expressing HDMEC.

Analyses of transcriptional changes that differ between contemporary experiments using IT4var14 IE and uninfected RBC in co-culture with TNF-activated HBMEC (Fig 5) show a variety of responses. Pathway analysis using BioPlanet (2019), KEGG (2021) and WikiPathways (2021) accessed via Enrichr identified several interesting groups of genes (see S5–S7 Files for details). Amongst those that are statistically significant are several relevant pathways:

- Cluster X1 –two examples from this cluster are the Syndecan-1 pathway, which has recently been implicated in degradation of the endothelial glycocalyx during malaria infection, and ‘regulation of apoptosis’, which may be involved in localised endothelial changes during cytoadherence.
- Cluster X3 –a pathway linked to the immune response to SARS-CoV-2 is identified, which is interesting due to the similarities in vascular pathology seen with this virus and during malaria infections, particularly linked to changes to CXCL family members [39]. One member of this family, CXCL10, has previously been shown to overexpressed during malaria infection [40], is transcribed at lower levels in blood samples taken from CM cases compared to UM [41].
- Cluster X4 –Notch signalling pathways, which are involved in a wide range of endothelial functions including cell-cell interactions and apoptosis, are seen in this cluster [42, 43]. The identification of complement and coagulation cascade genes is also interesting given the role of these processes in malaria pathogenesis [44, 45].
- Cluster X5 –the finding of the ‘TGF-beta pathway of extracellular regulation’ fits with previous work showing an association with TGF-beta and malaria [46], including potential roles in modulation of pathology [47] and overlap with genes controlled by specific miRNA and TGF-beta in experimental cerebral malaria [48].
- Cluster X6 –in addition to TGF-beta signalling, this cluster also identified the brain-derived neurotrophic factor (BDNF) signalling pathway, which may play a role in neuroprotection during hypoxia or oxidative damage [49].

The key message from these results is that the presence of IE causes significant changes in endothelial cell transcription and identifies pathways that have been previously associated with malaria infection and pathology of severe disease. To highlight some of the interesting pathways, changes in the endothelial glycocalyx associated with CM pathology were originally identified in mouse models [50] but recent data from several groups have validated a role for the glycocalyx in human disease [51–54]. This work has identified downstream effects of this phenotype based on genes linked to Syndecan-1 mediated signalling (e.g., HGF) that have the potential to alter several endothelial phenotypes, including the induction of pro-inflammatory and pro-adhesive states [55]. Endothelial apoptosis has been linked with malaria [56, 57], including variation in the degree of apoptosis induced by different parasite isolates [58], and could, at least in part, explain the formation of highly localised lesions (ring haemorrhages) in cerebral microvessels during CM. Our study identified a number of genes linked to apoptosis, including BMP4, which induces EC cell death [59], the Notch signalling pathway [60] and CXCL8 (also known as IL-8). CXCL8 is increased in severe malaria [61], its expression associated with parasite density [62] and has been consistently identified as a DEG in co-culture studies [25, 28, 63]. This protein inhibits endothelial apoptosis [64], which may be an adaptation by the parasite to reduce host cell damage. Interestingly, IL8/CXCL8 is also involved in neutrophil recruitment, which is increasingly being seen as having an important role in malaria pathogenesis [65].

Several members of the eicosanoid synthesis pathway were identified as DEGs in our study, particularly in the production of prostacyclin (PLA2; PTGES; PTGIS; CYP1A1). There is little information about prostacyclin and malaria disease, with a single case study showing amelioration of CM in a patient using prostacyclin [66], and inhibition of the development of experimental CM in the mouse model using a prostacyclin analogue, Iloprost [67]. The best evidence to support a role for this pathway in human CM comes from a study showing associations between phospholipase A2, which is an intermediate in the eicosanoid pathway, and brain volume in CM [68]. Prostacyclin is interesting because its roles include regulation of endothelial inflammation and apoptosis, both of which have been implicated in pathology.

Several other pathways, including those involved in the hypoxia protective response and Wnt signalling, showed some preferential expression but did not reach formal statistical significance in our experiments, although the pathway ‘cell-cell signalling by wnt’ was identified in the analysis of GO Biological Pathways (R5) (see Supplementary Information), which links with findings of the wnt/beta-catenin pathway being involved in brain endothelial integrity in malaria [69]. BBB integrity is important in the pathology of cerebral malaria as brain swelling is strongly associated with mortality [15] and as well as relevant pathways involved in the regulation of endothelial barrier function, individual DEGs associated with barrier integrity, such as EpHA2 [70], were also identified in our study.

The findings in Fig 8 of the induced transcriptional profiles obtained by co-culture with two different parasite lines, IT4var14 and IT4var37, show significant differences, suggesting some role for specific PfEMP1 in the modulation of the endothelial cell response to IE cytoadherence. Gene Ontology analyses (see Fig 9 and S9 & S10 Files) identify a range of biological processes and molecular functions associated with the differentially expressed clusters, particularly pathways linked to inflammation and cell adhesion as well as those involved in DNA replication and repair, potentially reflecting a role in apoptosis which has previously been associated with endothelial cell responses to co-culture with *P*. *falciparum* patient isolates [57] but less so with some laboratory parasite lines [29]. Indeed, Avril et al. (2019) [29] showed several differences in co-culture results than those previously reported, as well as variability in pro-inflammatory cytokine secretion across the three *P*. *falciparum* strains they tested, consistent with our suggestion that endothelial responses are influenced by the parasite variant being used, and potentially providing an explanation for the different clinical outcomes seen in malaria infections based on ‘virulent’ PfEMP1 types. This is also consistent with the link discovered between PfEMP1 variants and severe malaria disease, namely the identification of DC8 and DC13 variants that bind to EPCR and are seen frequently in parasites sampled from severely ill patients, including CM [71, 72].

From the clusters of DEGs shown in Fig 8, two have relatively similar patterns of expression over time with some moderation of effect (clusters X2 and X4), whereas X1 and X3 show very different patterns of expression between IT4var37 and IT4var14. Of the latter two clusters, X3 showed very little significant grouping of function using GO term analysis, but cluster X1 identified several biological processes (BP) and molecular functions (MF) that showed different patterns of expression between HBMEC co-culture with the two parasite variants. For example, ‘BP-positive regulation of cell-cell adhesion’, which includes genes for adhesion proteins such as ICAM-1 and VCAM as well as a range of chemokines (CX3CL1; CCL2) and cytokines (IL6; TNF; IL1B), was down-regulated in IT4var37 co-culture but showed a transient increase with IT4var14, which could influence both Pf-IE and monocyte recruitment within a pro-inflammatory microenvironment. ‘MF-metalloendopeptidase activity’ is also identified in this cluster, containing genes for ADAMTS1/4/5/6/12/18 that regulate vascular homeostasis and are involved in cell adhesion and inflammation, as well as MMP7, which through degradation of soluble VEGFR-1, promotes VEGF binding to endothelium [73], with elevated levels of VEGF being reported in patients with cerebral malaria [74] and increased VEGF expression in HBMEC treated with malaria patient sera [75]. Cluster X1 shows a major difference in the expression of Tenascin C (TNC) (Fig 9B) linked to the induction of expression by IT4var37, IT4var14 or RBC co-culture. TNC is a multi-functional protein [76] that has been implicated in modulating cell adhesion [77] and endothelial signalling [78]. It is expressed at low levels in under normal conditions but upregulated in a range of cell types, including endothelium, during inflammation and cell repair [79]. TNFAIP3 Interacting Protein-3 (TNIP3) shows a similar pattern of expression to TNC and is interesting due to its role in reducing cell death and inflammation during ischemia [80]. For Cluster X4, CD74 expression is enhanced in IT4var37 co-culture compared to IT4var14 and RBC. The CD74/ macrophage migration inhibitory factor (MIF) interaction has been shown to modulate endothelial damage [81] and pericyte contractility and neutrophil extravasation [82]. SNAI2 is another multi-function protein with a differential pattern of transcription in our co-culture experiments, with roles in several areas associated with malaria disease, including apoptosis and signalling (Wnt; Notch) [83].

As part of our analysis, we also looked for ‘discordant’ DEGs–genes with significant changes in transcription during co-culture, but that changed in different directions with IT4var14 and IT4var37. S7 and S8 Figs show Venn diagrams of these comparisons. Common DEGs between IT4var14 and IT4var37 are more frequently seen at 20 hours co-culture (S7 Fig) and tend to be concordant between the parasite variants, probably reflecting a substantive role of non-variant, parasite-released factors in modulating endothelial transcription. There are few shared DEGs seen at 6 hours co-culture (S8 Fig) and these are distributed evenly between concordant and discordant categories. However, it was interesting to note that the six DEGs with increased transcription in IT4var14 and decreased in IT4var37 (S8 Fig–chart) included four (CXCL10; IL-6; IL-8 (CXCL8); VCAM-1) showing associations with severe malaria [41, 84–86].

These types of parasite-endothelium co-culture studies provide an opportunity to identify plausible genes and pathways associated with malaria disease. From clinical studies and *in vitro* research, it seems likely that contributions to pathology come from many different factors, including the location of sequestered infected erythrocytes, reduction in vascular flow, host inflammation and endothelial dysfunction caused by the presence and rupture of infected erythrocytes. Models of malaria cytoadherence can help by providing supporting data on endothelial responses to a range of components derived from IE adhesion or locally released factors, and some of these models have become quite sophisticated [87, 88]. In carrying out these experiments we became aware of some of the variables involved:

- Complexity of the model–the endothelial environment in which cytoadherence takes place is not solely defined by the presence of endothelial cells and Pf-IE. In addition to other cells involved in the neurovascular unit in the brain, pericytes and astrocytes, there are levels of systemic inflammatory mediators and inflammatory cells that will also influence the ‘health’ of the micro-endothelium. The influence of blood flow on the expression of a range of markers is known to be important, although whether endothelium in cytoadherence foci is operating under flow or static conditions is not known. We chose to employ a relatively simple model using primary microvascular human brain endothelium in a static environment with trophozoite stage IE. Co-culture was optimised for endothelium, as moving towards parasite permissive conditions resulted in endothelial cell death. This does lead to IE not developing normally during co-culture. After 6 hours, the parasites were still at trophozoite stage and although an occasional schizont or ring was detected, there was little parasite development. Thus, IE would be still bound to HBMEC. After 20 hours, the parasites appeared pyknotic and did not progress to the ring stage. Parasites were seen outside RBC, likely due to RBC lysis.
  The choice of primary brain endothelium was to replicate the type of environment for cerebral malaria, and our use of primary cells came from several groups’ findings of changes in endothelial responses seen in immortalised lines compared to primary cells. Activation of the endothelium with TNF prior to co-culture was used to replicate systemic activation recorded in fatal cases of cerebral malaria [89, 90], although this subsequently had to be controlled for due to uncertainty in the decay of TNF transcriptional signals during IE co-culture. Our subsequent studies to this work have included a TNF-only treatment group as a control.
- Choice of parasite lines–both PfEMP1 variants are derived from the same parental line (IT4) and so should have little genomic variation. IT4var14 is a group B, long-form PfEMP1 (Fig 1) showing relatively high levels of binding to TNF-activated HBMEC. IT4var37 is a group C, short form PfEMP1 that binds poorly to brain endothelial cells. These variants were chosen to represent very different cytoadherence characteristics. However, these co-cultures studies were initiated in 2016, prior to the publication of work identifying ICAM-1/ EPCR dual binding as being associated with cerebral malaria [14], and had this information been available at the time we would have considered using one of the group B/A, dual-binding variants in our experiments.
  The group C variants [7, 91] are interesting as they are thought to be associated with asymptomatic chronic infections. The low levels of adhesion to some types of endothelia seen with this group may allow larger effects mediated by Pf-IE on endothelial function to be tolerated by the host, which might not be permissive for variants displaying high levels of binding.
  Antigenic switching during *in vitro* culture is well-known and will result in some heterogeneity in the variants present in the parasite cultures. A monoclonal antibody for IT4VAR14 protein is available (mAb BC6 [92]) and this was used to select and monitor PfEMP1 expression in the cultures, which was maintained at > 70% of the Pf-IE expressing this variant. Group C *var* genes including IT4var37 have been shown to switch at low rates and so naturally maintain relative high proportions of specific variant types (see Materials and methods section).
- Reproducibility–there is a trade-off between having a depth of data and affordability. We chose to perform three biological replicates for each condition as we had a wide range of variables to cover (+/- TNF; RBC vs IT4var14 vs IT4var37). As can be seen from S4 Fig, the IT4var37 experiments gave excellent reproducibility and were conducted after our experience with the IT4var14 co-cultures and done over a period of 3 weeks. We also had to remove some data as the TNF pre-activation step occasionally failed, which was monitored by looking at known induced genes (e.g., ICAM1; VCAM1). The baseline 0 h results were different between the IT4var14 and RBC co-culture experiments (which were conducted at the same time) and those carried out for IT4var37, therefore we were not able to use the RBC/TNF comparison with IT4var37, hence the different approaches used in the analyses.
  As part of another study, we have produced a Fluidigm chip set which contains several of the differentially regulated genes identified from these experiments. Two new independent co-culture experiments were carried out using TNF-activated HBMEC and IT4var14 or RBC and the mRNA derived from these tested using a Fluidigm panel (*actb*, *actg1*, *adam10*, *adam17*, *angpt2*, *ank1*, *bax*, *bcl2a1*, *c3*, *casp3*, *ccl14*, *cdh5*, *cxcl3*, *cyp1a1*, *dusp5*, *eef1a1*, *fas*, *hes1*, *hey1*, *hey2*, *icam1*, *il1a*, *il1b*, *il6*, *klf2*, *klf3*, *klf4*, *klf10*, *lamc2*, *mib1*, *nfkb1*, *nfkb1a*, *notch2*, *ocln*, *papln*, *pecam1*, *plas2g4a*, *plxna4*, *procr*, *ptgis*, *ptgs2*, *s100a10*, *sele*, *smad6*, *tgfb1*, *tjp1*, *tnfrsf12a*, *tspan13*, *tubb*, *txnip*, *vcam1*). Simple linear regression analysis (S9 Fig) of the Fluidigm and RNAseq results for these genes suggests some reproducibility, but statistical significance (p = 0.01) is achieved only with the removal of four ‘outliers’ (*ccl14*, *il1b*, *klf4*, *txnip*) out of 51 genes tested. The observation of some concordance between repeat experiments suggests that these types of studies can provide a basis for the development of hypotheses on the role of specific pathways in malaria disease for further testing.
- Parasite viability–the conditions during co-culture favour endothelial cell viability, otherwise the main transcriptional signature that would be derived would be of dying endothelial cells. Our observation of the IE during co-culture is that their development in the erythrocytic cycle is significantly retarded and very few schizonts were observed after co-culture. After 2 and 6 hours of co-culture the IE are mainly intact, but after 20 hours there is visible IE lysis, which will expose the endothelial cells to the contents of the IE as well as interactions with the IE membrane. Other studies have shown that some effects are due to IE contact or soluble factors from lysed IE, and we are not able to discriminate between these effects in our work.
- What would we do differently–hindsight is a wonderful thing and through its lens we have an opportunity to improve the experimental design of these studies. In addition to increasing the number of biological replicates and being more focussed, we would;
  - Carry out pilot experiments, including transcriptional analysis, to look for novel variables. We spent a long time optimising co-culture conditions but had not predicted the longevity and sometimes variability of the TNF induction effect on transcription, and the variation in baseline transcription in endothelium from the same batch but cultured at different times (and different passage number). Also, we cannot discount the effect of using different Group O blood donors for parasite culture.
  - Incorporate trophozoite and schizont stage IE into the co-culture experiments, with the latter replicating what happens during schizogony in the sequestered blood vessel.
  - Consider single cell transcriptional analysis. We know that there is variation in the endothelial population, for example in terms of surface receptor expression, which may affect interactions with IE. Bulk transcriptional analysis may miss key effects due to the variation in endothelial responses within a population. Single cell analysis would provide an opportunity to look for different cell populations and conduct more specific interrogation of the data.

**Fig 10:**
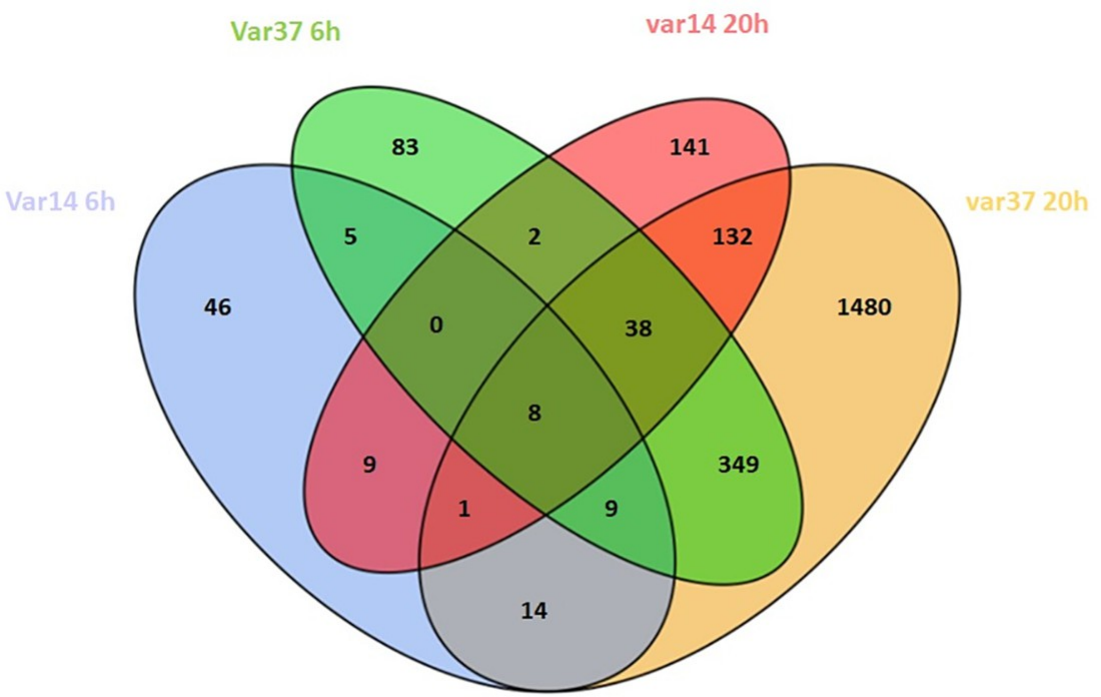
Venn diagram of differentially expressed genes (DEG) identified during HBMEC co-culture with IT4var14 and IT4var37 Pf-IE at 6 hours and 20 hours (DEG lists from S5 File).

We have performed a series of endothelium/ Pf-IE co-culture experiments using an *in vitro* model to look at the time course of changes in endothelial transcription due to contact with *Plasmodium falciparum* infected red blood cells. Our results show a range of profiles of DEGs in pathways involved in signalling, inflammation, cell-cell interactions, apoptosis and barrier integrity, as well as other processes for which information is not available to associate them with disease severity. IT4var14 (group B) and IT4var37 (group C) co-culture with HBMEC produce DEG lists that have some overlap but show considerable differences between them (Fig 10), suggesting that different parasite variants will produce varying endothelial responses during infection, which may influence disease pathology. As with all models, some caution needs to be exercised in the direct attribution of biological pathways identified in our study to pathology, but the differential DEG signatures seen here and the association of disease with specific classes of PfEMP1 [9, 93–95] do support a role for virulence linked to PfEMP1 variant type resulting in cytoadherence-mediated endothelial dysfunction and disease. This works alongside mechanisms that are variant-independent and based on the release of parasite-derived material during schizont rupture, such as seen in the recent work of Zuniga et al. (2022) [32]. Our work has identified a number of plausible new pathways linked to malaria disease. Further studies using more sophisticated models of IE cytoadherence and validation in clinical studies are needed to provide a better understanding of the mechanisms underpinning the pathology of cerebral malaria.

## Materials and methods

### Malaria parasite culture

Laboratory-adapted IT4var14 (A4) [92] and IT4var37 (4E12) [7] *P*. *falciparum* isolates were cultured at 2% haematocrit in O+ human erythrocytes in complete RPMI 1640 medium (supplemented with 10% human serum, 37.5 mM HEPES, 11 mM D-glucose, 25 mg/ml gentamicin sulphate, 2 mM L-glutamine, pH 7.4) [96]. The endothelial binding characteristics of the two parasite lines are shown in S2 Fig.

IT4var14 binds to ICAM-1 and CD36, whereas IT4var37 is a strong CD36-binder but does not bind to ICAM-1 [7]. This is consistent with the patterns of endothelial binding see in S2 Fig, with IT4var37 showing high levels of adhesion to HDMEC, but almost no binding to HUVEC and HBMEC, which do not express CD36 (binding to HBMEC is similar to HUVEC, as both cell types do not express CD36). IT4var14 binds to HUVEC, HBMEC and HDMEC.

*P*. *falciparum* undergoes antigenic variation in culture, which changes the proportion of IE expressing specific PfEMP1s in the parasite populations. To minimize *var* gene switching, IT4var14 was selected with specific BC6 antibody [92] coated protein G Dynabeads using standard protocols [6]. Previous work from the Newbold laboratory has shown that IT4var14 expression is reduced by around 10% over 10 cycles of growth [97]. IT4var37 does not have a specific antibody available for selection, but this variant is common [7] and the presence of a significant proportion of this PfEMP1 expression was observed by qRT-PCR using specific primers (ABRA6 –[98]). IT4var37 has been shown previously to maintain its *var* gene expression after 4–5 cycles of growth [7]. The selected and screened parasite strains were used for experiments for up to 3 weeks in culture.

The parasite lines, EC cultures, washed blood cells and parasite culture media were regularly monitored for mycoplasma contamination using the Universal Mycoplasma Detection Kit (ATCC^®^ 30-1012K^™^).

### Endothelial cell culturing

Primary human brain microvascular endothelial cells (HBMEC) were obtained from Cell Systems, USA (ACBRI 376). HBMEC were cultured in 1% gelatin-coated flasks in Promocell’s Endothelial Cell Growth Medium MV (C-22020) supplemented with Endothelial Cell Growth Medium MV Supplement Mix (C-39225) and passaged using the Detach Kit (C-41220), following manufacturer’s instructions.

### Co-culture of EC with malaria parasite

HBMEC at passage 4 to 8 were cultured until 80–90% confluency in 25 cm^2^ flasks and, for +TNF conditions, stimulated with 10 ng/ml TNF overnight. Pf-IE were synchronised with 5% sorbitol, cultured until they reached 5–8% parasitaemia (mature trophozoite stages) and enriched by Plasmion to obtain 50–60% parasitaemia. The enriched Pf-IE were resuspended in EC medium to a standard parasitemia of 50% at 1% haematocrit and overlayed onto the HBMEC for 0 (on-off), 2, 6 and 20 hours, in triplicate, in a 5% CO_2_ incubator. Uninfected red blood cells were used as a control. One T25 flask at full confluence (approximately 1.2 x 10^6^ HBMEC) was used per experiment with the addition of 2.5 x 10^8^ infected or uninfected erythrocytes, giving an IE/HBMEC ratio of approximately 200.

At each time point, the co-culture medium was removed, and the cells were washed once with EC medium to remove unbound IE or uninfected RBC. 1.5 ml of EC medium was added to each flask prior to harvesting the cells by cell-scraper. The cells were transferred to 1.5 ml sterile tubes and stored at -80°C.

**Fig 11:**
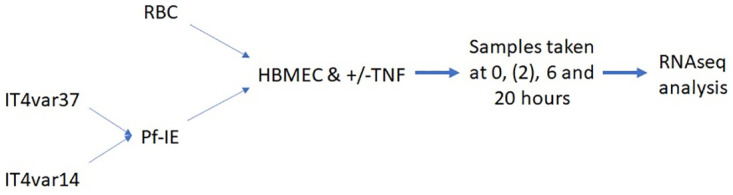
Schematic workflow of the co-culture experiments. Samples for IT4var37 experiments were taken at 0, 6 and 20 hours only.

The experimental structure is summarised in Fig 11.

### RNA extraction and quality assessment

RNA was extracted using the RNeasy Mini Kit (Qiagen, 74104) following manufacturer’s instructions. In short, samples were centrifuged at 13,000 rpm for 1 min, 350 μl RTL buffer was added to the cell pellet and homogenized for 1 minute by vortexing, 350 μl 70% ethanol was added and the homogenate transferred to an RNeasy mini spin column. The column was washed three times and the total RNA was eluted with 2 x 50 μl RNase-free water and stored at -80°C.

The quality of the RNA samples was assessed using an Agilent 2100 Bioanalyzer (RNA 6000 Nano Kit, 5067–1511, Agilent Technologies) following manufacturer instructions. All RNA samples had a RIN of 8.5 or higher, indicating high-quality RNA.

### RNA sequencing

Strand-specific RNA-sequencing (ssRNA-seq) libraries were prepared using the Illumina Truseq Stranded mRNA Sample Preparation Kit (Illumina, RS-122-2101) following the manufacturer’s instructions. Briefly, 1ug of total RNA was used to purify mRNA using poly-T oligo-attached magnetic beads. mRNA was then fragmented and cDNA was synthesised using SuperScript III reverse transcriptase (Thermofisher, 18080044), followed by adenylation on the 3′ end, barcoding and adapter ligation. The adapter ligated cDNA fragments were then enriched and cleaned with Agencourt Ampure XP beads (Agencourt, A63880). Libraries validation was conducted using the 1000 DNA kit on 2100 Bioanalyzer (Agilent Technologies, 5067–1504) and quantified using qubit (Thermofisher, Q32850). ssRNA libraries were sequenced on Illumina Hiseq4000.

### Bioinformatics for RNA sequencing data

Unmapped paired-end sequences from an Illumina HiSeq4000 sequencer were tested by FastQC (http://www.bioinformatics.babraham.ac.uk/projects/fastqc/). Sequence adapters were removed, and reads were quality trimmed using Trimmomatic_0.39 [99]. The reads were mapped against the reference human genome (hg38) using STAR_2.7.7a [100]. Counts per gene will be calculated with HTSeq [101] using annotation from GENCODE 27 (http://www.gencodegenes.org/). Normalisation, Principal Components Analysis, and differential expression were calculated with DESeq2_1.18.1 [102]. Adjusted *p*-values were corrected for multiple testing (Benjamini and Hochberg method). K-means clustering was performed in R_4.1.1. Heatmaps were drawn with complexHeatmap v2.12.1 [103]. Gene ontology enrichment was studied using clusterProfiler [104]. Details of the analyses, including code, are in S2, S4 and S8 Files.

### Gene expression assays using the Fluidigm system

Gene expression qPCR assays were conducted on the IT4var14 RNA samples for a panel of 57 genes selected following the analysis of the RNA-seq dataset. In short, cDNA was prepared with reverse transcription before pre-amplification (13-cycles) and clean up of the reactions using Exonuclease 1, following the manufacturer protocols (100–6472 B1, PN 100–5875 C1) and reagents (Fluidigm,100–6297; Fluidigm, 100–5580; New England Biolabs, M0293S; TEKnova, 10221). Real-time PCR data was collected using the Biomark HD system with an 96.96 IFC chip using Fluidigm Delta-Gene Assays on the pre-amplified cDNA. The manufacturing protocol was followed for use of the Biomark HD system (PN 100–9792 A1) using the reagents specified for preparing samples (Biorad, 172–5211; Fluidigm, 100–7609)) and assays (Fluidigm 100–7611; TEKnova, 10221). Each reaction was performed in triplicate. Relative gene expression was calculated using the 2^-ΔΔCt^ method as previously described [105]. The average Ct of each sample triplicate was determined, and calculations were performed using GAPDH as the endogenous control (ΔCt). The RBC samples at 20 hours were used as the normalization controls to calculate relative fold changes (ΔΔCt) for each experiment. The exponential fold changes were calculated, and the values converted to log2FC.

## Supporting information

S1 Fig Summary diagram of host proteins identified as receptors for Pf-IE interactions. (TIF)

S2 Fig Endothelial cell adhesion assays under flow conditions: TNF-activated ECs were seeded in Vena8 biochips (Cellix) pre-coated with fibronectin; an IE suspension of 3% parasitaemia and 2% haematocrit in binding buffer (RPMI 1640 with 25 mM HEPES, 11 mM glucose, 2 mM glutamine, pH 7.2) was passed over confluent ECs for five minutes followed by washing with binding buffer for two minutes before counting six fields. The results show IE binding (mean ± SD, n = 3). For detailed assay conditions see [6]. Binding to TNF-activated HUVEC and HBMEC by IT4var14 is via ICAM-1 using the DBLβ5 domain whereas binding to TNF-activated HDMEC is mediated for IT4var14 by ICAM-1 and CD36 using the DBLβ5 and CIDRα5 domain. Binding to TNF-activated HDMEC by IT4var37 is via CD36 and uses the CIDRα3.1 domain.
(TIF)

S3 Fig K-means clustering of the means of expression levels of DEGs in clusters identified in RBC and IT4var14 co-culture with HBMEC without prior TNF activation. (TIF)

S4 Fig PCA of HBMEC/TNF co-culture samples for IT4var14 and IT4var37 used in the analysis. (TIF)

S5 Fig Venn diagrams showing the relationship between DEGs over time induced in HBMEC by Pf-IE (IT4var14) co-culture without and with TNF activation of HBMEC. Data from S1 File using Padj<0.05 & transcriptional changes (up or down) greater than 2-fold.
(TIF)

S6 Fig Distribution of DEGs at varying time points of IT4var14 co-culture with TNF-activated HBMEC with corresponding RBC-induced DEGs removed (S5 File). (TIF)

S7 Fig Comparison of up and down regulated DEGs (compared to 0 hours control) for IT4var14 and IT4var37 with TNF-activated HBMEC at 20 hours co-culture. Data from S5 File.
(TIF)

S8 Fig Comparison of up and down regulated DEGs (compared to 0 hours control) for IT4var14 and IT4var37 with TNF-activated HBMEC at 6 hours co-culture. The inset graph shows details of fold changes (compared to the relevant 0 hours control) for a subset of genes showing ‘discordant’ expression between the two parasite variants. DEG list data from S5 File and expression data from S1 File.
(TIF)

S9 Fig Comparison of fold changes in gene expression between independent RNAseq (FC (RNAseq)) and Fluidigm (FC (Fldgm)) experiments using TNF-activated HBMEC co-cultured with IT4var14. The results shown are for 6 hours of co-culture. Outliers are marked with blue triangles and are not included in the regression analysis.
(TIF)

S1 File RNAseq data counts and fold changes. (XLSX)

S2 File HBMEC noTNF—Pf-IE and RBC comparison. (HTML)

S3 File HBMEC noTNF—Padj < 0.05, fold change > 2. (XLSX)

S4 File Pf-IE (IT4var14) comparison with RBC. (HTML)

S5 File DEG lists and pathways. (XLSX)

S6 File R5 cluster analysis. (ZIP)

S7 File R5 clusters—Top pathways. (XLSX)

S8 File IT4var14 and IT4var37 comparison. (HTML)

S9 File R1 & R4 analyses. (ZIP)

S10 File var14/ var37 clusters GO lists. (XLSX)

S11 File RNAsequences raw data list for analyses. (XLSX)
