# Supplementary figures and images for: Different PfEMP1-expressing Plasmodium falciparum variants induce divergent endothelial transcriptional responses during co-culture
Source: PLoS One. 2023 Nov 30;18(11):e0295053. doi: 10.1371/journal.pone.0295053 (PMC10688957; doi:10.1371/journal.pone.0295053)

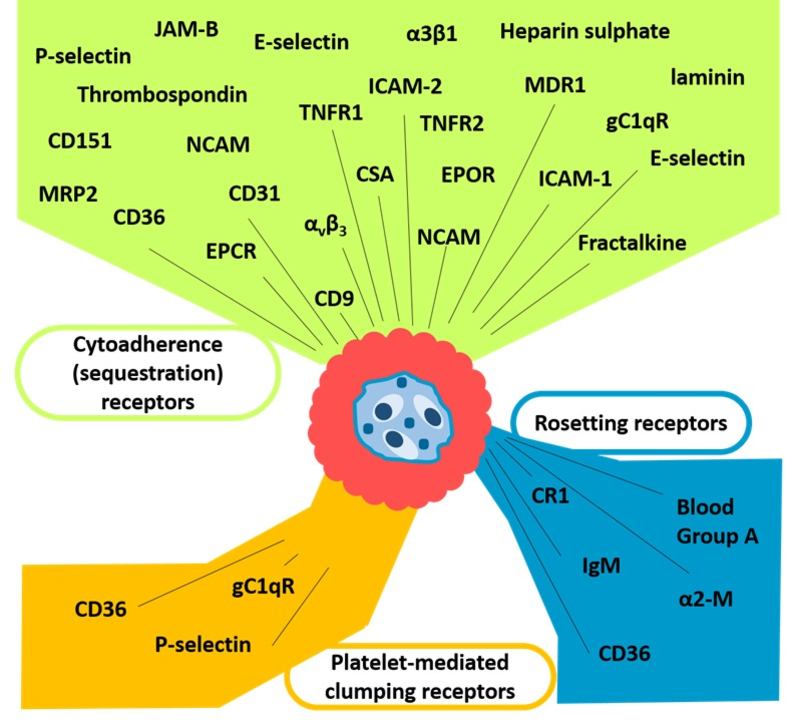

Supplement: S1 Fig — (TIF) [file pone.0295053.s001.tif]

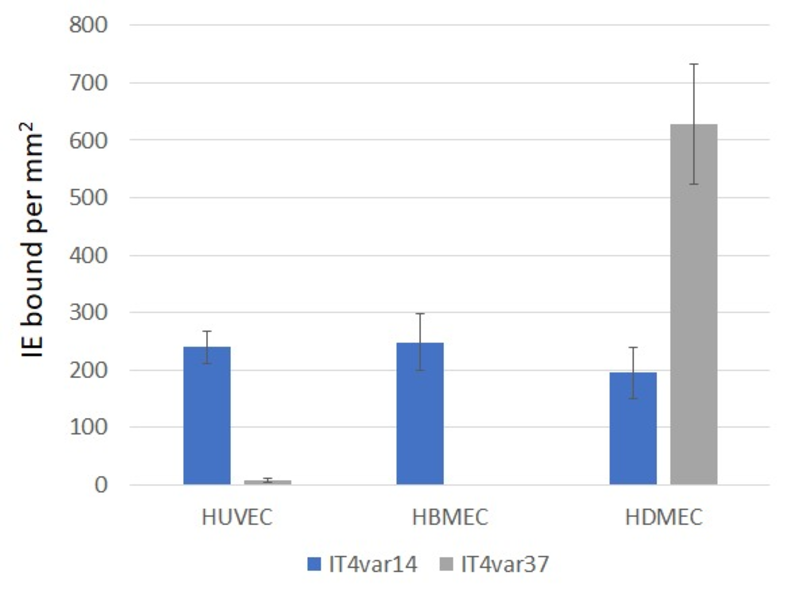

Supplement: S2 Fig — The results show IE binding (mean ± SD, n = 3). For detailed assay conditions see [6]. Binding to TNF-activated HUVEC and HBMEC by IT4var14 is via ICAM-1 using the DBLβ5 domain whereas binding to TNF-activated HDMEC is mediated for IT4var14 by ICAM-1 and CD36 using the DBLβ5 and CIDRα5 domain. Binding to TNF-activated HDMEC by IT4var37 is via CD36 and uses the CIDRα3.1 domain. (TIF) [file pone.0295053.s002.tif]

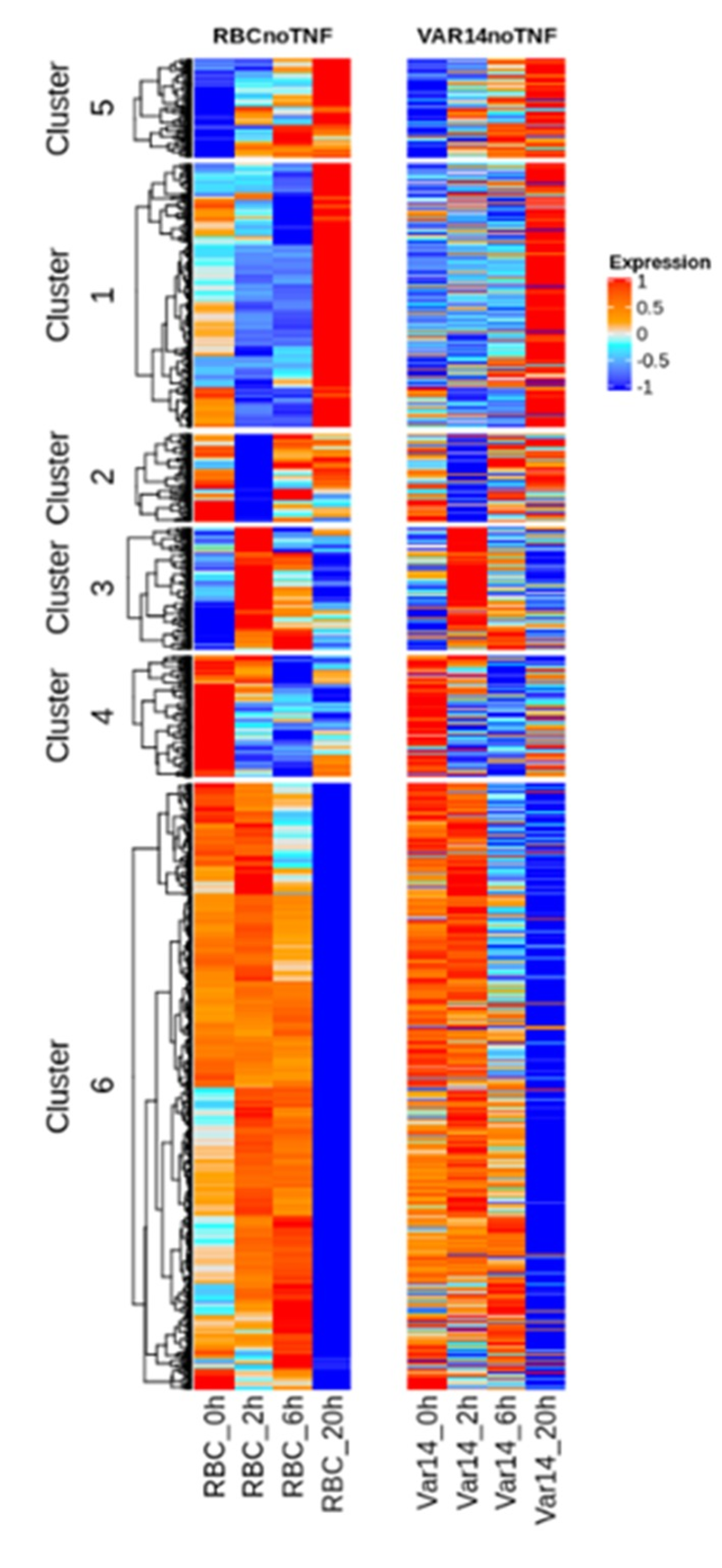

Supplement: S3 Fig — (TIF) [file pone.0295053.s003.tif]

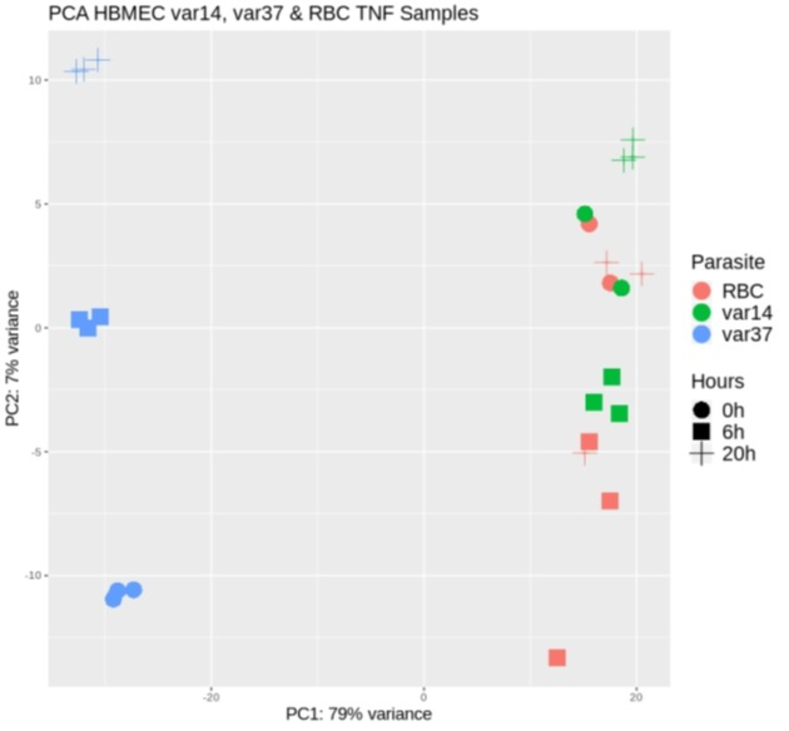

Supplement: S4 Fig — (TIF) [file pone.0295053.s004.tif]

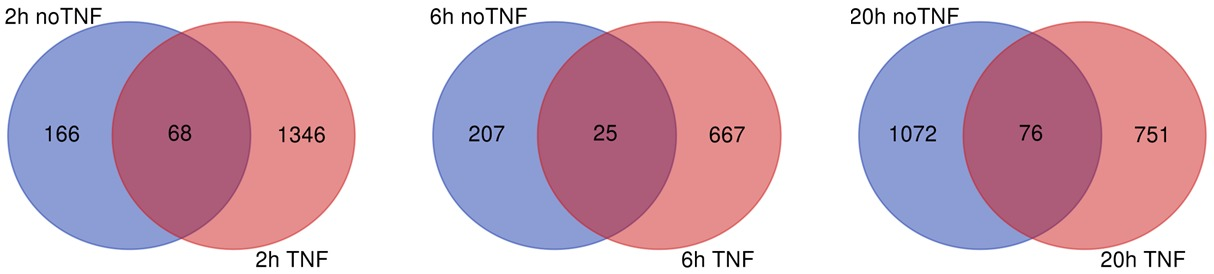

Supplement: S5 Fig — Data from S1 File using Padj<0.05 & transcriptional changes (up or down) greater than 2-fold. (TIF) [file pone.0295053.s005.tif]

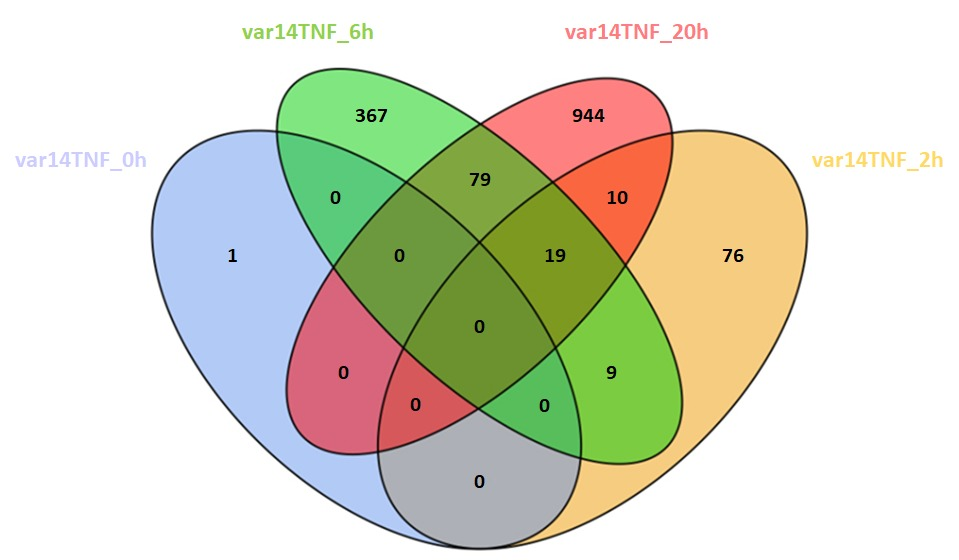

Supplement: S6 Fig — (TIF) [file pone.0295053.s006.tif]

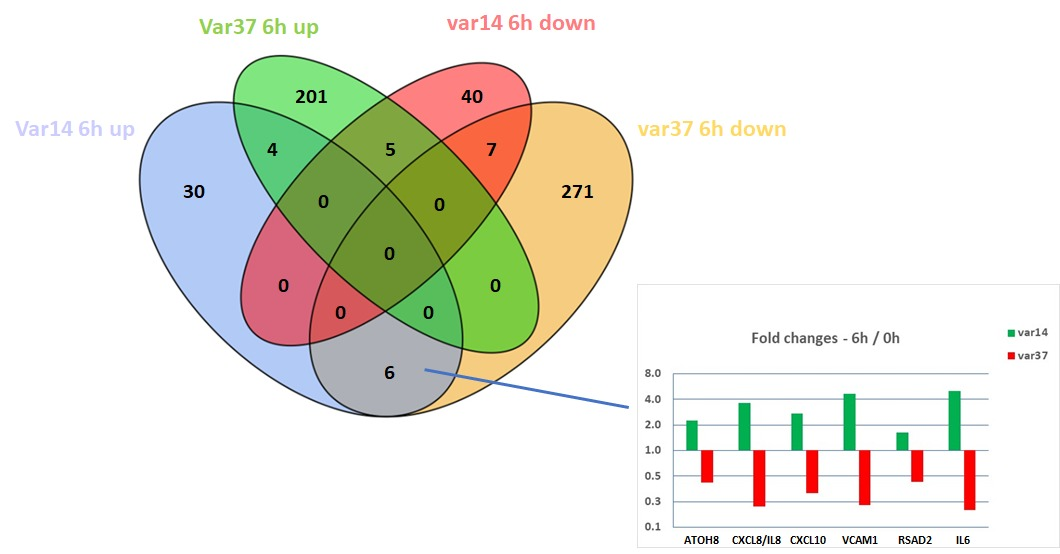

Supplement: S7 Fig — Data from S5 File. (TIF) [file pone.0295053.s007.tif]

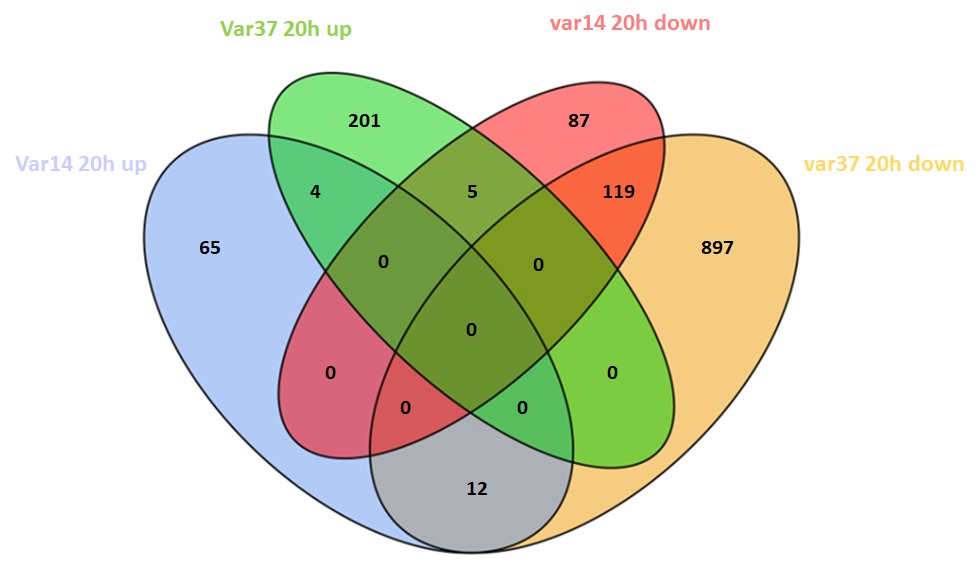

Supplement: S8 Fig — The inset graph shows details of fold changes (compared to the relevant 0 hours control) for a subset of genes showing ‘discordant’ expression between the two parasite variants. DEG list data from S5 File and expression data from S1 File. (TIF) [file pone.0295053.s008.tif]

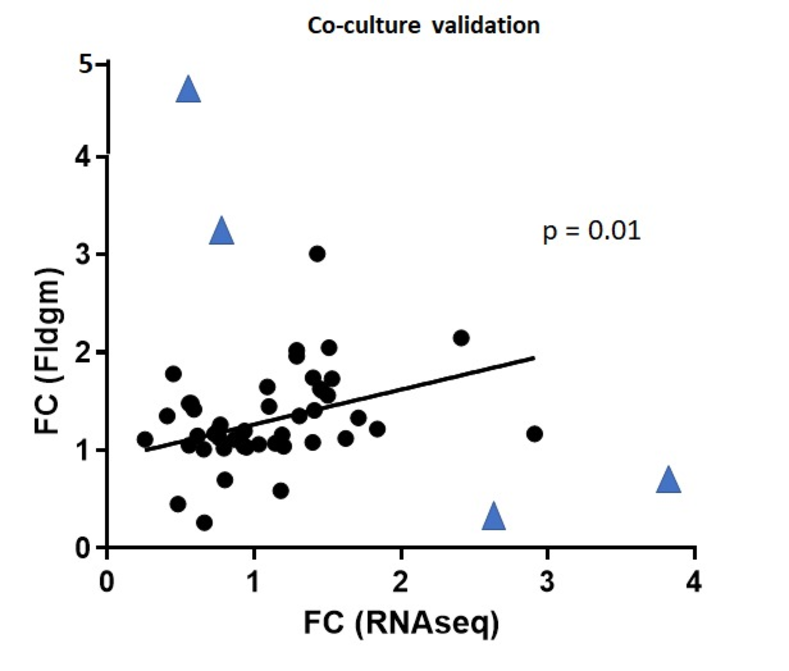

Supplement: S9 Fig — The results shown are for 6 hours of co-culture. Outliers are marked with blue triangles and are not included in the regression analysis. (TIF) [file pone.0295053.s009.tif]
