# Supplementary material for: Different PfEMP1-expressing Plasmodium falciparum variants induce divergent endothelial transcriptional responses during co-culture
Source: PLoS One. 2023 Nov 30;18(11):e0295053. doi: 10.1371/journal.pone.0295053 (PMC10688957; doi:10.1371/journal.pone.0295053)
Supplement: S2 File — (HTML) [file pone.0295053.s011.html]

 

 

 

 
 
 


 


 KCouper/Liverpool K-means RNAseq Analysis November 2020 

 
 
 
 
 
 
 
 
 
 
 
 
 

 
 
 


 


 

 


 

 

 
 


 

 


 


 

 
  Code     
 
  Show All Code  
  Hide All Code  
  
  Download Rmd  
 
 


 KCouper/Liverpool K-means RNAseq Analysis November 2020 
 Leo Zeef 

 


 
 
 Analysis Sections 
 Viewing is better if Code is hidden (Top Right drop down list) 
 
 
 
  sink(file="RsessionInfoDESeq2.txt")
library('DESeq2')
library("ggplot2")
library(reshape2)
####library(tidyverse)
####library(splitstackshape)
####library(data.table)
library("RColorBrewer")
library("gplots")
####library('ggdendro')
library('ggrepel')
library("dplyr")
library("ComplexHeatmap")
library("clusterProfiler")
library(VennDiagram) ######
library(UpSetR)
library(gridExtra)
library(cluster)
library(circlize)
library(factoextra)
library(NbClust)
library("biomaRt")
library("org.Hs.eg.db")####human
library("org.Mm.eg.db")####mouse
library(venn)
####library(org.At.tair.db)####arabidopsis
sessionInfo()
sink()
#########################################
####multiplot
#########################################
#### Multiple plot function
####
#### ggplot objects can be passed in ..., or to plotlist (as a list of ggplot objects)
#### - cols:   Number of columns in layout
#### - layout: A matrix specifying the layout. If present, 'cols' is ignored.
#### If the layout is something like matrix(c(1,2,3,3), nrow=2, byrow=TRUE),
#### then plot 1 will go in the upper left, 2 will go in the upper right, and
#### 3 will go all the way across the bottom.
multiplot <- function(..., plotlist=NULL, file, cols=1, layout=NULL) {
  library(grid)
  
  #### Make a list from the ... arguments and plotlist
  plots <- c(list(...), plotlist)
  
  numPlots = length(plots)
  
  #### If layout is NULL, then use 'cols' to determine layout
  if (is.null(layout)) {
    #### Make the panel
    #### ncol: Number of columns of plots
    #### nrow: Number of rows needed, calculated from #### of cols
    layout <- matrix(seq(1, cols * ceiling(numPlots/cols)),
                     ncol = cols, nrow = ceiling(numPlots/cols))
  }
  
  if (numPlots==1) {
    print(plots[[1]])
    
  } else {
    #### Set up the page
    grid.newpage()
    pushViewport(viewport(layout = grid.layout(nrow(layout), ncol(layout))))
    
    #### Make each plot, in the correct location
    for (i in 1:numPlots) {
      #### Get the i,j matrix positions of the regions that contain this subplot
      matchidx <- as.data.frame(which(layout == i, arr.ind = TRUE))
      
      print(plots[[i]], vp = viewport(layout.pos.row = matchidx$row,
                                      layout.pos.col = matchidx$col))
    }
  }
}
####function my code edit of plotPCA
####################################
plotPCALeo<-function (x, intgroup = "Treatment", ntop = 500, returnData = FALSE, PCx=1, PCy=2)
{
  ####rv <- rowVars(assay(x))
  rv = apply((assay(x)), 1, var)
  select <- order(rv, decreasing = TRUE)[seq_len(min(ntop, 
                                                     length(rv)))]
  pca <- prcomp(t(assay(x)[select, ]))
  percentVar <- pca$sdev^2/sum(pca$sdev^2)
  if (!all(intgroup %in% names(colData(x)))) {
    stop("the argument 'intgroup' should specify columns of colData(dds)")
  }
  intgroup.df <- as.data.frame(colData(x)[, intgroup, drop = FALSE])
  group <- factor(apply(intgroup.df, 1, paste, collapse = " : "))
  d <- data.frame(PCX = pca$x[, PCx], PCY = pca$x[, PCy], group = group, 
                  intgroup.df, names = colnames(x))
  if (returnData) {
    attr(d, "percentVar") <- percentVar[PCx:PCy]
    return(d)
  }
  ggplot(data = d, aes_string(x = "PCX", y = "PCY", color = "group")) + 
    ####ggplot(data = d, aes_string(x = "PCX", y = "PCY", color=Tgfb1, shape=Treatment)) + 
    geom_point(size = 3) + xlab(paste0("PC",PCx,": ", round(percentVar[1] * 
                                                              100), "% variance")) + ylab(paste0("PC",PCy,": ", round(percentVar[2] * 
                                                                                                                        100), "% variance"))
}  
 
 
 
 
 
 
  col_fun = colorRamp2(c(-1,-0.2, 0,0.2, 1), c("blue","cyan", "grey90","orange", "red"))#heatmap colours
colorsV <- c("cornflowerblue", "yellow3", "brown1")#Venn colours
colorsV2 <- c("mediumorchid1",  "chartreuse3")#Venn colours
colorsV5<-c("cornflowerblue", "orange2", "green3","purple","red")#Venn colours
#col_fun(seq(-3, 3))  
 
 
 
 
 VAR37TNF k-means q0.05 
 
 1. Genelist Selection 
 
 
 
  r r groupsName<-1_VAR37TNF_kmeans_q0.05 
   
 
 
 
 
 
 
  countsTable<-read.delim("RNAseq2019July_5.txt", header = TRUE, sep = "\t",check.names=FALSE,row.names=1)
head(countsTable)  
 
 
 
 
 
 
 
 
 
 
 
  AllGeneNames<-countsTable$Gene_Symbol
#head(AllGeneNames)  
 
 
 
 
 
 
  tempA<-countsTable  
 
 
 
 
 
 
  topDEgenes <- which(tempA$padj_R1_Var37_Hours_6h_vs_0h<0.05&!is.na(tempA$padj_R1_Var37_Hours_6h_vs_0h))####find indexes 
listA<-tempA[ topDEgenes, ]$Gene_Symbol
topDEgenes <- which(tempA$padj_R1_Var37_Hours_20h_vs_0h<0.05&!is.na(tempA$padj_R1_Var37_Hours_20h_vs_0h))####find indexes 
listB<-tempA[ topDEgenes, ]$Gene_Symbol
topDEgenes <- which(tempA$padj_R1_Var37_Hours_20h_vs_6h<0.05&!is.na(tempA$padj_R1_Var37_Hours_20h_vs_6h))####find indexes 
listC<-tempA[ topDEgenes, ]$Gene_Symbol
vennq<-venn.diagram(x = list(listA,listB,listC) ,
            category.names = c("Var37_6h_vs_0h","Var37_20h_vs_0h","Var37_20h_vs_6h"),
            main="padj<0.05",
            filename = NULL,  scaled = FALSE, fill = colorsV, cat.col = colorsV, cat.cex = 1, cat.dist=0.1,  margin = 0.15)
topDEgenes <- which(tempA$pvalue_R1_Var37_Hours_6h_vs_0h<0.05&abs(tempA$log2FoldChange_R1_Var37_Hours_6h_vs_0h)>1&!is.na(tempA$padj_R1_Var37_Hours_6h_vs_0h))####find indexes 
listA<-tempA[ topDEgenes, ]$Gene_Symbol
topDEgenes <- which(tempA$pvalue_R1_Var37_Hours_20h_vs_0h<0.05&abs(tempA$log2FoldChange_R1_Var37_Hours_20h_vs_0h)>1&!is.na(tempA$padj_R1_Var37_Hours_20h_vs_0h))####find indexes 
listB<-tempA[ topDEgenes, ]$Gene_Symbol
topDEgenes <- which(tempA$pvalue_R1_Var37_Hours_20h_vs_6h<0.05&abs(tempA$log2FoldChange_R1_Var37_Hours_20h_vs_6h)>1&!is.na(tempA$padj_R1_Var37_Hours_20h_vs_6h))####find indexes 
listC<-tempA[ topDEgenes, ]$Gene_Symbol
vennp<-venn.diagram(x = list(listA,listB,listC) ,
            category.names = c("Var37_6h_vs_0h","Var37_20h_vs_0h","Var37_20h_vs_6h"),
            main="pvalue<0.05&fold change>2",
            filename = NULL,  scaled = FALSE, fill = colorsV, cat.col = colorsV, cat.cex = 1, cat.dist=0.1,  margin = 0.15)  
 
 
 
 
 
 
  topDEgenes <- which((tempA$padj_R1_Var37_Hours_6h_vs_0h<0.05&!is.na(tempA$padj_R1_Var37_Hours_6h_vs_0h))|
                      (tempA$padj_R1_Var37_Hours_20h_vs_0h<0.05&!is.na(tempA$padj_R1_Var37_Hours_20h_vs_0h))|
                      (tempA$padj_R1_Var37_Hours_20h_vs_6h<0.05&!is.na(tempA$padj_R1_Var37_Hours_20h_vs_6h))
)
listA<-tempA[ topDEgenes, ]$Gene_Symbol
topDEgenes <- which((tempA$pvalue_R1_Var37_Hours_6h_vs_0h<0.05&abs(tempA$log2FoldChange_R1_Var37_Hours_6h_vs_0h)>1&!is.na(tempA$padj_R1_Var37_Hours_6h_vs_0h))|####find indexes 
  (tempA$pvalue_R1_Var37_Hours_20h_vs_0h<0.05&abs(tempA$log2FoldChange_R1_Var37_Hours_20h_vs_0h)>1&!is.na(tempA$padj_R1_Var37_Hours_20h_vs_0h))|####find indexes 
 (tempA$pvalue_R1_Var37_Hours_20h_vs_6h<0.05&abs(tempA$log2FoldChange_R1_Var37_Hours_20h_vs_6h)>1&!is.na(tempA$padj_R1_Var37_Hours_20h_vs_6h))
 )####find indexes 
listC<-tempA[ topDEgenes, ]$Gene_Symbol
vennpq<-venn.diagram(x = list(listA,listC) ,
            category.names = c("padj<0.05","p<0.05&fc>2"),
            main="padj compared to pvalue",
            filename = NULL,  scaled = FALSE, fill = colorsV2, cat.col = colorsV2, cat.cex = 1, cat.dist=0.1,  margin = 0.15)  
 
 
 
 
 
 
  grid.arrange(gTree(children=vennq), gTree(children=vennpq) , ncol=2,top="R1 Var37_TNF")  
 
 
   
 
 
 
 
 
 
  #tempA<-resAll[-c(10:30) ]
tempA<-countsTable
#rownames(tempA)
rownames(tempA) <- NULL
tempA = mutate(tempA, Include=
                   ifelse(tempA$padj_R1_Var37_Hours_6h_vs_0h<0.05&!is.na(tempA$padj_R1_Var37_Hours_6h_vs_0h), "in", 
                          ifelse(tempA$padj_R1_Var37_Hours_20h_vs_0h<0.05&!is.na(tempA$padj_R1_Var37_Hours_20h_vs_0h), "in", 
                                  ifelse(tempA$padj_R1_Var37_Hours_20h_vs_6h<0.05&!is.na(tempA$padj_R1_Var37_Hours_20h_vs_6h), "in",
                                                                       "out"))))
tempA  
 
 
 
 
 
 
 
  ####library(dplyr)
tempA %>%
     group_by(Include) %>% 
     tally()  
 
 
 
 
 
 
 
 
 
 
 
  topDEgenes <- which(tempA$Include=="in")####find indexes   
 
 
 
 
 
 NB Please check columns used and renamed for plots 
 
 
 
  baseMeansHm <-countsTable[,c(48:50)]
head(baseMeansHm)  
 
 
 
 
 
 
 
  colnames(baseMeansHm)<-c("Var37TNF_0h","Var37TNF_6h","Var37TNF_20h")
head(baseMeansHm)  
 
 
 
 
 
 
 
 
 
 
 
  dataHi <-countsTable[,c(18:26)]
colnames(dataHi)<-c("Var37TNF_0h_1","Var37TNF_0h_2","Var37TNF_0h_3","Var37TNF_6h_1","Var37TNF_6h_2","Var37TNF_6h_3","Var37TNF_20h_1","Var37TNF_20h_2","Var37TNF_20h_3")
head(dataHi)  
 
 
 
 
 
 
 
  dataHi<-dataHi[ topDEgenes, ]
dataHi <- log2(dataHi+1)
dataHi<- t(as.matrix(dataHi))
dataHi <- t(scale(dataHi))
####str(dataHi)  
 
 
 
 
 
 
  topDEgenes <- which(tempA$Include=="in")####find indexes   
 
 
 
 
 
 
  #### NB edits required to colours of levels of factor for each experiment & if levels are dropped
#dataHM<-assay(logData)
Hours<-factor(c(rep("V37_0h",3),rep("V37_0h",3),rep("Var37_20h",3)),levels=c("0h","6h","20h"))
####y<-data.frame(t(dataHM),Genotype,Drug)####2 factors
y<-data.frame(t(dataHi),Hours)####single factor
metadataPlot<-y[,c(ncol(y)-1,ncol(y))]
####metadataPlot
#### Add factor annotation (from https://www.biostars.org/p/317349/)
####ann <- data.frame(metadataPlot$Genotype,metadataPlot$Drug)####2 factors
ann <- data.frame(metadataPlot$Hours)####single factor
####colnames(ann) <- c("Genotype","Drug")####2 factors
colnames(ann) <- c("ExpFactor")####1 factor
####colours <- list("Genotype"=c("WT"="seagreen","KO"="rosybrown"), "Drug"=c("Cntl"="cyan","AZT"="khaki1"))
colours <- list("ExpFactor"=c("V37_0h"="red","V37_0h"="rosybrown","Var37_20h"="cyan"))
colAnn <- HeatmapAnnotation(df=ann, which="col", col=colours, annotation_width=unit(c(2, 4), "cm"), gap=unit(1, "mm"))  
 
 
 
 
 
 
  ####ym<-data.frame(t(dataHM),Age,Strain)####2 factors
ExpFactorMn<-factor(c(rep("0h",1),rep("6h",1),rep("20h",1)),
             levels=c("0h","6h","20h"))#### i.e. add each level of factor once for means
dataHMm<-baseMeansHm
ym<-data.frame(t(dataHMm),ExpFactorMn)####single factor
####head(ym)
metadataPlotm<-ym[,c(ncol(ym)-1,ncol(ym))]
####metadataPlot
#### Add factor annotation (from https://www.biostars.org/p/317349/)
####ann <- data.frame(metadataPlot$Genotype,metadataPlot$Drug)####2 factors
annm <- data.frame(metadataPlotm$ExpFactorMn)####single factor
####colnames(ann) <- c("Genotype","Drug")####2 factors
colnames(annm) <- c("ExpFactorMn")####1 factor
####colours <- list("Genotype"=c("WT"="seagreen","KO"="rosybrown"), "Drug"=c("Cntl"="cyan","AZT"="khaki1"))
coloursm <- list("ExpFactor"=c("0h"="green","6h"="rosybrown","20h"="cyan"))
colAnnm <- HeatmapAnnotation(df=annm, which="col", col=coloursm, annotation_width=unit(c(2, 4), "cm"), gap=unit(1, "mm"))  
 
 
 
 
 
 2. Hierachical clustering of means (individual samples added for inspection) 
 
 
 
  hmap_hier_factors1 <- Heatmap(
  dataHi,  name = "ExpressionI",
  column_title = paste0("Individual Samples"), 
  column_title_gp = gpar(fontsize = 16, fontface = "bold"),
  width = unit(300, "mm"),
  col = col_fun,
  cluster_rows = FALSE,
  cluster_columns = FALSE,
  show_row_names = FALSE)
  #top_annotation=colAnn  )
####means
dataHMm<-baseMeansHm[ topDEgenes, ]
dataHMm <- log2(dataHMm+1)
dataHMm<- t(as.matrix(dataHMm))
dataHMm <- t(scale(dataHMm))
#colAnnm <- HeatmapAnnotation(df=annm, which="col", col=coloursm, annotation_width=unit(c(2, 4), "cm"), gap=unit(1, "mm"))
hmap_hier_factors4 <- Heatmap(
  dataHMm,  name = "Expression",
  row_labels = paste0(rownames(dataHMm)," ",(tempA[ topDEgenes, ])$Gene_Symbol),
  column_title = paste0("Means"), 
  col = col_fun,
  column_title_gp = gpar(fontsize = 16, fontface = "bold"),
  width = unit(50, "mm"),
  cluster_columns = FALSE,
  show_row_names = FALSE)
  #top_annotation=colAnnm  )
hmap_hier_factors4+hmap_hier_factors1  
 
 
   
 
 
 
 
 
 
  r r par(mfrow=c(1,2)) #### Silhouette method fviz_nbclust(dataHMm, kmeans, method = ,k.max = 16)+ labs(subtitle = method) 
    
 
 
   
 
 
  r r #### Elbow method fviz_nbclust(dataHMm, kmeans, method = ,k.max = 16) + labs(subtitle = method) 
    
 
 
   
 
 
 
 
 
 
  r r ####gap stat slow!!! ####set.seed(123) ####fviz_nbclust(dataHMm, kmeans, nstart = 25, method = _stat, nboot = 100,k.max = 16)+ #### labs(subtitle = statistic method) 
    
 
 
 
 
 
 
  r r #kclust1 <- kmeans(dataHMm, 6) #silhouette plot distK<-daisy(dataHMm) plot(silhouette(kclust1$cluster, distK), col=1:6, border=NA) 
    
 
 
   
 
 
 
 
 
 3. K-means clustering of means 
 
 
 
  #split <- paste0("Cluster\n", kclust1$cluster)
split <- factor(paste0("Cluster\n", kclust1$cluster), levels=c("Cluster\n5","Cluster\n6","Cluster\n4","Cluster\n2","Cluster\n1","Cluster\n3"))
hmap_k <- Heatmap(dataHMm, split=split, cluster_row_slices = FALSE,
                  cluster_columns = FALSE,
                  show_row_names = FALSE,
                  name = "Expression",
                  col = col_fun,
                  width = unit(20, "mm"),
                  column_title = "means", 
                  column_title_gp = gpar(fontsize = 16, fontface = "bold")
                  
                        )#top_annotation=colAnn)
hmap_hier_factors1 <- Heatmap(
  dataHi,  name = "ExpressionI",
  col = col_fun,
  column_title = paste0("individual samples"), 
  column_title_gp = gpar(fontsize = 16, fontface = "bold"),
  width = unit(60, "mm"),
  cluster_rows = FALSE,
  cluster_columns = FALSE,
  show_row_names = FALSE)
hmap_k  
 
 
   
 
 
 
 
 
 K-means clustering of means (with cluster annotation and individual samples added for inspection) 
 
 
 
  Response_Time<-data.frame(kclust1$cluster)
Response_Time = mutate(Response_Time, Response=
                   ifelse(Response_Time$kclust1.cluster==1, "early", 
                          ifelse(Response_Time$kclust1.cluster==2, "transient",
                                 ifelse(Response_Time$kclust1.cluster==3, "late",
                                        ifelse(Response_Time$kclust1.cluster==4, "transient",
                                               ifelse(Response_Time$kclust1.cluster==5, "early",
                                                      ifelse(Response_Time$kclust1.cluster==6, "late",
                                                                       "out")))))))
Response_Time<-Response_Time[c(2)]
rownames(Response_Time) <- NULL
ha = HeatmapAnnotation(df = Response_Time, which = "row", width = unit(1, "cm"),col = list(Response = c("early" =  "green3", "late" = "brown", "transient" = "violet")))
hmap_k+ha+hmap_hier_factors1  
 
 
   
 
 
 
 Mean profiles of clusters 
 
 
 
  r r clustercount<-data.frame(kclust1 \(cluster) clustersizes<-table(clustercount\) kclust1.cluster) clusterMeans<-data.frame(kclust1$centers) clusterMeans1<-data.frame(t(clusterMeans)) clusterMeans1 <- cbind(rownames(clusterMeans1), clusterMeans1) orderN<-c(37TNF_0h,37TNF_6h,37TNF_20h)#### manual rownames(clusterMeans1) <- NULL names(clusterMeans1)[names(clusterMeans1)==(clusterMeans1)] <-  
####clusterMeans1 pX1<-ggplot(data=clusterMeans1, aes(x=Sample, y=X1,group=1)) + geom_line()+ geom_point()+ggtitle(paste(X1 Profile ,clustersizes[1], genes))+ scale_x_discrete(limits=orderN)+ theme(axis.title.x = element_blank(),axis.title.y = element_blank()) pX2<-ggplot(data=clusterMeans1, aes(x=Sample, y=X2,group=1)) + geom_line()+ geom_point()+ggtitle(paste(X2 Profile ,clustersizes[2], genes))+ scale_x_discrete(limits=orderN)+ theme(axis.title.x = element_blank(),axis.title.y = element_blank()) pX3<-ggplot(data=clusterMeans1, aes(x=Sample, y=X3,group=1)) + geom_line()+ geom_point()+ggtitle(paste(X3 Profile ,clustersizes[3], genes))+ scale_x_discrete(limits=orderN)+ theme(axis.title.x = element_blank(),axis.title.y = element_blank()) pX4<-ggplot(data=clusterMeans1, aes(x=Sample, y=X4,group=1)) + geom_line()+ geom_point()+ggtitle(paste(X4 Profile ,clustersizes[4], genes))+ scale_x_discrete(limits=orderN)+ theme(axis.title.x = element_blank(),axis.title.y = element_blank()) pX5<-ggplot(data=clusterMeans1, aes(x=Sample, y=X5,group=1)) + geom_line()+ geom_point()+ggtitle(paste(X5 Profile ,clustersizes[5], genes))+ scale_x_discrete(limits=orderN)+ theme(axis.title.x = element_blank(),axis.title.y = element_blank()) pX6<-ggplot(data=clusterMeans1, aes(x=Sample, y=X6,group=1)) + geom_line()+ geom_point()+ggtitle(paste(X6 Profile ,clustersizes[6], genes))+ scale_x_discrete(limits=orderN)+ theme(axis.title.x = element_blank(),axis.title.y = element_blank()) #plot multiplot(pX1, pX2, pX3, pX4,pX5, pX6, cols=2) 
    
 
 
   
 
 
 
 
 
 K-means clustering of means (other treatment means added for inspection) 
 
 
 
  split <- factor(paste0("Cluster\n", kclust1$cluster), levels=c("Cluster\n5","Cluster\n6","Cluster\n4","Cluster\n2","Cluster\n1","Cluster\n3"))
hmap_k <- Heatmap(dataHMm, split=split, cluster_row_slices = FALSE,
                  cluster_columns = FALSE,
                  show_row_names = FALSE,
                  name = "Expression",
                  col = col_fun,
                  width = unit(20, "mm"),
                  column_title = "Var37TNF", 
                  column_title_gp = gpar(fontsize = 10, fontface = "bold"))
                  
                  
baseMeansHmTemp <-countsTable[,c(48:50)]
colnames(baseMeansHmTemp)<-c("Var37TNF_0h","Var37TNF_6h","Var37TNF_20h")
dataHMmR1_37Y<-baseMeansHmTemp[ topDEgenes, ]
dataHMmR1_37Y <- log2(dataHMmR1_37Y+1)
dataHMmR1_37Y<- t(as.matrix(dataHMmR1_37Y))
dataHMmR1_37Y <- t(scale(dataHMmR1_37Y))
baseMeansHmTemp <-countsTable[,c(60:63)]
colnames(baseMeansHmTemp)<-c("Var14_0h","Var14_2h","Var14_6h","Var14_20h")
dataHMmR2_14<-baseMeansHmTemp[ topDEgenes, ]
dataHMmR2_14 <- log2(dataHMmR2_14+1)
dataHMmR2_14<- t(as.matrix(dataHMmR2_14))
dataHMmR2_14 <- t(scale(dataHMmR2_14))
baseMeansHmTemp <-countsTable[,c(79:82)]
colnames(baseMeansHmTemp)<-c("RBC_0h","RBC_2h","RBC_6h","RBC_20h")
dataHMmR2_R<-baseMeansHmTemp[ topDEgenes, ]
dataHMmR2_R <- log2(dataHMmR2_R+1)
dataHMmR2_R<- t(as.matrix(dataHMmR2_R))
dataHMmR2_R <- t(scale(dataHMmR2_R))
baseMeansHmTemp <-countsTable[,c(110:113)]
colnames(baseMeansHmTemp)<-c("Var14TNF_0h","Var14TNF_2h","Var14TNF_6h","Var14TNF_20h")
dataHMmR4_14T<-baseMeansHmTemp[ topDEgenes, ]
dataHMmR4_14T <- log2(dataHMmR4_14T+1)
dataHMmR4_14T<- t(as.matrix(dataHMmR4_14T))
dataHMmR4_14T <- t(scale(dataHMmR4_14T))
baseMeansHmTemp <-countsTable[,c(129:132)]
colnames(baseMeansHmTemp)<-c("RBC_TNF_0h","RBC_TNF_2h","RBC_TNF_6h","RBC_TNF_20h")
dataHMmR4_RT<-baseMeansHmTemp[ topDEgenes, ]
dataHMmR4_RT <- log2(dataHMmR4_RT+1)
dataHMmR4_RT<- t(as.matrix(dataHMmR4_RT))
dataHMmR4_RT <- t(scale(dataHMmR4_RT))
hmap_37T <- Heatmap(dataHMmR1_37Y, split=split, 
                  name = "Expression37T",  
                  column_title = "VAR37TNF", 
                  cluster_columns = FALSE,  show_row_names = FALSE,col = col_fun,width = unit(20, "mm"),
                  column_title_gp = gpar(fontsize = 10, fontface = "bold"))
hmap_k14 <- Heatmap(dataHMmR2_14, split=split, 
                  name = "Expression14",  
                  column_title = "VAR14noTNF", 
                  cluster_columns = FALSE,  show_row_names = FALSE,col = col_fun,width = unit(25, "mm"),
                  column_title_gp = gpar(fontsize = 10, fontface = "bold"))
hmap_R <- Heatmap(dataHMmR2_R, split=split, 
                  name = "ExpressionR",  
                  column_title = "RBCnoTNF", 
                  cluster_columns = FALSE,  show_row_names = FALSE,col = col_fun,width = unit(25, "mm"),
                  column_title_gp = gpar(fontsize = 10, fontface = "bold"))
hmap_k14T <- Heatmap(dataHMmR4_14T, split=split, 
                  name = "Expression14T",  
                  column_title = "VAR14TNF", 
                  cluster_columns = FALSE,  show_row_names = FALSE,col = col_fun,width = unit(25, "mm"),
                  column_title_gp = gpar(fontsize = 10, fontface = "bold"))
hmap_RT <- Heatmap(dataHMmR4_RT, split=split, 
                  name = "ExpressionRT",  
                  column_title = "RBCTNF", 
                  cluster_columns = FALSE,  show_row_names = FALSE,col = col_fun,width = unit(25, "mm"),
                  column_title_gp = gpar(fontsize = 10, fontface = "bold"))
hmap_k+ha +hmap_k14T+hmap_RT+hmap_k14+hmap_R  
 
 
   
 
 
 
 
 
 
  r r topDEgenes <- which(tempA \(Include==\in\)####find indexes tempAkm<-tempA[ topDEgenes, ] SymbolsKm<-dplyr::pull(tempAkm, Gene_Symbol) #### export the gene expression data for the clusters write.table(clusterMeans,paste0(\ClusterMeansKm_\,groupsName,\.txt\), sep = \\t\) ClusteredGenes<-data.frame(kclust1\) cluster,SymbolsKm,dataHMm) write.table(ClusteredGenes,paste0(_,groupsName,.txt), sep = \t) #head(ClusteredGenes) 
    
 
 
 
 
 
 
  r r bottomDEgenes<-which(tempA$Include==)####find indexes bottomG<-tempA[ bottomDEgenes, ] bottomG<-dplyr::pull(bottomG, Gene_Symbol) write.table(bottomG,paste0(_,groupsName,.txt), sep = \t) 
 topDEgenes <- which(tempA \(Include==\in\)####find indexes tempAkm<-tempA[ topDEgenes, ] SymbolsKm<-dplyr::pull(tempAkm, Gene_Symbol) ipaKmeans<-ClusteredGenes #countsTable <-countsTable[,c(1:15)]####if samples need removing ipaKmeans<-ipaKmeans[,c(1:2)] ipaKmeans\) name2<-rownames(ipaKmeans) #ipaKmeans%>% rownames_to_column(var = ) #ipaKmeans #rowid_to_column(ipaKmeans) ipaKmeans = mutate(ipaKmeans, x1= ifelse(ipaKmeans \(kclust1.cluster==1, \1\, \0\)) ipaKmeans = mutate(ipaKmeans, x2= ifelse(ipaKmeans\) kclust1.cluster==2, , )) ipaKmeans = mutate(ipaKmeans, x3= ifelse(ipaKmeans \(kclust1.cluster==3, \1\, \0\)) ipaKmeans = mutate(ipaKmeans, x4= ifelse(ipaKmeans\) kclust1.cluster==4, , )) ipaKmeans = mutate(ipaKmeans, x5= ifelse(ipaKmeans \(kclust1.cluster==5, \1\, \0\)) ipaKmeans = mutate(ipaKmeans, x6= ifelse(ipaKmeans\) kclust1.cluster==6, , )) #ipaKmeans write.table(ipaKmeans,paste0(_,groupsName,.txt), sep = \t) #head(ipaKmeans) 
    
 
 
 
 
 
 
  r r ClusteredGenes2<-ClusteredGenes[c(1)] #ClusteredGenes2 listAll<-list() for(i in 1:6) { clusterName<-paste0(,i) #clusterName<-row.names(subset(ClusteredGenes,ClusteredGenes==i)) clusterName<-(subset(ClusteredGenes$SymbolsKm,ClusteredGenes==i)) listAll[[i]]<-clusterName } #need to name the vectors in the list, example here is for 8 clusters names(listAll)<-c(1, 2, 3, 4,5, 6) #if you want to rearrange the order #listAll<-listAll[c(3, 7, 8, 2, 6, 5, 4, 1)] #lapply(listAll, head) 
    
 
 
 
 
 
 4. Annotation of K-means clusters 
 
 CC cellular compartment 
 BP biological process 
 MF molecular function 
 
 The simplify function has been used to cut down on GO redundancy 
 
 
 
  r r #str(AllGeneNames) 
    
 
 
 
 
 
 
  r r ####CC cgoCC <- compareCluster(geneCluster = listAll, universe = AllGeneNames, fun = , OrgDb=org.Hs.eg.db, ####OrgDb=org.Mm.eg.db, keyType=, ont = , pvalueCutoff=0.05, qvalueCutoff = 0.10) cgoCC2 <- simplify(cgoCC, cutoff=0.7, by=.adjust, select_fun=min) ####write as spreadsheet write.csv(as.data.frame(cgoCC2),paste0( CC ,groupsName,.csv)) dotplot(cgoCC2,showCategory = 30, title = paste0(Cellular Compartment ,groupsName))+ theme(axis.text.x = element_text(angle = 90, vjust = 0.5, hjust=1)) 
    
 
 
   
 
 
 
 Plots and GO data were written to files 
 
 
 
  r r png(paste0( CC ,groupsName,.png), width = 1224, height = 424) dotplot(cgoCC2,showCategory = 30, title = paste0(Cellular Compartment ,groupsName))+ theme(axis.text.x = element_text(angle = 90, vjust = 0.5, hjust=1)) dev.off() 
    
 
 
  null device 
          1   
 
 
 
 GO BP 
 
 
 
  r r ####CC cgoBP <- compareCluster(geneCluster = listAll, universe = AllGeneNames, fun = , OrgDb=org.Hs.eg.db, keyType=, ont = , pvalueCutoff=0.05, qvalueCutoff = 0.10) cgoBP2 <- simplify(cgoBP, cutoff=0.7, by=.adjust, select_fun=min) ####write as spreadsheet write.csv(as.data.frame(cgoBP2),paste0( BP ,groupsName,.csv)) dotplot(cgoBP2,showCategory = 30, title = paste0(Biological Process ,groupsName))+ theme(axis.text.x = element_text(angle = 90, vjust = 0.5, hjust=1)) 
    
 
 
   
 
 
 
 
 
 
  r r png(paste0( BP ,groupsName,.png), width = 1024, height = 1224) dotplot(cgoBP2,showCategory = 30, title = paste0(Biological Process ,groupsName))+ theme(axis.text.x = element_text(angle = 90, vjust = 0.5, hjust=1)) dev.off() 
    
 
 
  null device 
          1   
 
 
 
 GO MF 
 
 
 
  r r ####MF cgoMF <- compareCluster(geneCluster = listAll, universe = AllGeneNames, fun = , OrgDb=org.Hs.eg.db, keyType=, ont = , pvalueCutoff=0.05, qvalueCutoff = 0.10) cgoMF2 <- simplify(cgoMF, cutoff=0.7, by=.adjust, select_fun=min) ####write as spreadsheet write.csv(as.data.frame(cgoMF2),paste0( MF ,groupsName,.csv)) dotplot(cgoMF2,showCategory = 30, title = paste0(Molecular Function ,groupsName))+ theme(axis.text.x = element_text(angle = 90, vjust = 0.5, hjust=1)) 
    
 
 
   
 
 
 
 
 
 
  r r png(paste0( MF ,groupsName,.png), width = 1424, height = 424) dotplot(cgoMF2,showCategory = 30, title = paste0(Molecular Function ,groupsName))+ theme(axis.text.x = element_text(angle = 90, vjust = 0.5, hjust=1)) dev.off() 
    
 
 
  null device 
          1   
 
 
 
 
 
 
 VAR37TNF k-means p0.05fc2 
 
 1. Genelist Selection 
 
 
 
  groupsName<-"R1_VAR37TNF_kmeans_p0.05fc2"  
 
 
 
 
 
 
  countsTable<-read.delim("RNAseq2019July_5.txt", header = TRUE, sep = "\t",check.names=FALSE,row.names=1)
head(countsTable)  
 
 
 
 
 
 
 
 
 
 
 
  r r AllGeneNames<-countsTable$Gene_Symbol #head(AllGeneNames) 
    
 
 
 
 
 
 
  grid.arrange(gTree(children=vennp), gTree(children=vennpq) , ncol=2,top="R1 Var37_TNF")  
 
 
   
 
 
 
 
 
 
  #tempA<-resAll[-c(10:30) ]
tempA<-countsTable
#rownames(tempA)
rownames(tempA) <- NULL
tempA = mutate(tempA, Include=
                   ifelse(tempA$pvalue_R1_Var37_Hours_6h_vs_0h<0.05&abs(tempA$log2FoldChange_R1_Var37_Hours_6h_vs_0h)>1&!is.na(tempA$padj_R1_Var37_Hours_6h_vs_0h), "in", 
                          ifelse(tempA$pvalue_R1_Var37_Hours_20h_vs_0h<0.05&abs(tempA$log2FoldChange_R1_Var37_Hours_20h_vs_0h)>1&!is.na(tempA$padj_R1_Var37_Hours_20h_vs_0h), "in", 
                                  ifelse(tempA$pvalue_R1_Var37_Hours_20h_vs_6h<0.05&abs(tempA$log2FoldChange_R1_Var37_Hours_20h_vs_6h)>1&!is.na(tempA$padj_R1_Var37_Hours_20h_vs_6h), "in",
                                                                       "out"))))
tempA  
 
 
 
 
 
 
 
  ####library(dplyr)
tempA %>%
     group_by(Include) %>% 
     tally()  
 
 
 
 
 
 
 
 
 
 
 
  topDEgenes <- which(tempA$Include=="in")####find indexes   
 
 
 
 
 
 NB Please check columns used and renamed for plots 
 
 
 
  baseMeansHm <-countsTable[,c(48:50)]
colnames(baseMeansHm)<-c("Var37TNF_0h","Var37TNF_6h","Var37TNF_20h")
head(baseMeansHm)  
 
 
 
 
 
 
 
 
 
 
 
  dataHi <-countsTable[,c(18:26)]
colnames(dataHi)<-c("Var37TNF_0h_1","Var37TNF_0h_2","Var37TNF_0h_3","Var37TNF_6h_1","Var37TNF_6h_2","Var37TNF_6h_3","Var37TNF_20h_1","Var37TNF_20h_2","Var37TNF_20h_3")
head(dataHi)  
 
 
 
 
 
 
 
  dataHi<-dataHi[ topDEgenes, ]
dataHi <- log2(dataHi+1)
dataHi<- t(as.matrix(dataHi))
dataHi <- t(scale(dataHi))
####str(dataHi)  
 
 
 
 
 
 
  topDEgenes <- which(tempA$Include=="in")####find indexes   
 
 
 
 
 
 2. Hierachical clustering of means (individual samples added for inspection) 
 
 
 
  hmap_hier_factors1 <- Heatmap(
  dataHi,  name = "ExpressionI",
  column_title = paste0("Individual Samples"), 
  column_title_gp = gpar(fontsize = 16, fontface = "bold"),
  width = unit(300, "mm"),
  col = col_fun,
  cluster_rows = FALSE,
  cluster_columns = FALSE,
  show_row_names = FALSE)
  #top_annotation=colAnn  )
####means
dataHMm<-baseMeansHm[ topDEgenes, ]
dataHMm <- log2(dataHMm+1)
dataHMm<- t(as.matrix(dataHMm))
dataHMm <- t(scale(dataHMm))
#colAnnm <- HeatmapAnnotation(df=annm, which="col", col=coloursm, annotation_width=unit(c(2, 4), "cm"), gap=unit(1, "mm"))
hmap_hier_factors4 <- Heatmap(
  dataHMm,  name = "Expression",
  row_labels = paste0(rownames(dataHMm)," ",(tempA[ topDEgenes, ])$Gene_Symbol),
  column_title = paste0("Means"), 
  col = col_fun,
  column_title_gp = gpar(fontsize = 16, fontface = "bold"),
  width = unit(50, "mm"),
  cluster_columns = FALSE,
  show_row_names = FALSE)
  #top_annotation=colAnnm  )
hmap_hier_factors4+hmap_hier_factors1  
 
 
   
 
 
 
 
 
 
  r r par(mfrow=c(1,2)) #### Silhouette method fviz_nbclust(dataHMm, kmeans, method = ,k.max = 16)+ labs(subtitle = method) 
    
 
 
   
 
 
  r r #### Elbow method fviz_nbclust(dataHMm, kmeans, method = ,k.max = 16) + labs(subtitle = method) 
    
 
 
   
 
 
 
 
 
 
  r r ####gap stat slow!!! ####set.seed(123) ####fviz_nbclust(dataHMm, kmeans, nstart = 25, method = _stat, nboot = 100,k.max = 16)+ #### labs(subtitle = statistic method) 
    
 
 
 
 
 
 
  r r #kclust2 <- kmeans(dataHMm, 6) #silhouette plot distK<-daisy(dataHMm) plot(silhouette(kclust2$cluster, distK), col=1:6, border=NA) 
    
 
 
   
 
 
 
 
 
 3. K-means clustering of means 
 
 
 
  #split <- paste0("Cluster\n", kclust2$cluster)
split <- factor(paste0("Cluster\n", kclust2$cluster), levels=c("Cluster\n2","Cluster\n5","Cluster\n1","Cluster\n6","Cluster\n4","Cluster\n3"))
hmap_k <- Heatmap(dataHMm, split=split, cluster_row_slices = FALSE,
                  cluster_columns = FALSE,
                  show_row_names = FALSE,
                  name = "Expression",
                  col = col_fun,
                  width = unit(20, "mm"),
                  column_title = "means", 
                  column_title_gp = gpar(fontsize = 16, fontface = "bold")
                  
                        )#top_annotation=colAnn)
hmap_hier_factors1 <- Heatmap(
  dataHi,  name = "ExpressionI",
  col = col_fun,
  column_title = paste0("individual samples"), 
  column_title_gp = gpar(fontsize = 16, fontface = "bold"),
  width = unit(60, "mm"),
  cluster_rows = FALSE,
  cluster_columns = FALSE,
  show_row_names = FALSE)
hmap_k  
 
 
   
 
 
 
 
 
 K-means clustering of means (with cluster annotation and individual samples added for inspection) 
 
 
 
  r r Response_Time<-data.frame(kclust2 \(cluster) Response_Time = mutate(Response_Time, Response=  ifelse(Response_Time\) kclust2.cluster==1, , ifelse(Response_Time \(kclust2.cluster==2, \early\,  ifelse(Response_Time\) kclust2.cluster==3, , ifelse(Response_Time \(kclust2.cluster==4, \early\,  ifelse(Response_Time\) kclust2.cluster==5, , ifelse(Response_Time$kclust2.cluster==6, , ))))))) Response_Time<-Response_Time[c(2)] rownames(Response_Time) <- NULL ha = HeatmapAnnotation(df = Response_Time, which = , width = unit(1, ),col = list(Response = c( = 3,  = ,  = ))) hmap_k+ha+hmap_hier_factors1 
    
 
 
   
 
 
 
 Mean profiles of clusters 
 
 
 
  r r clustercount<-data.frame(kclust2 \(cluster) clustersizes<-table(clustercount\) kclust2.cluster) clusterMeans<-data.frame(kclust2$centers) clusterMeans1<-data.frame(t(clusterMeans)) clusterMeans1 <- cbind(rownames(clusterMeans1), clusterMeans1) orderN<-c(37TNF_0h,37TNF_6h,37TNF_20h)#### manual rownames(clusterMeans1) <- NULL names(clusterMeans1)[names(clusterMeans1)==(clusterMeans1)] <-  
####clusterMeans1 pX1<-ggplot(data=clusterMeans1, aes(x=Sample, y=X1,group=1)) + geom_line()+ geom_point()+ggtitle(paste(X1 Profile ,clustersizes[1], genes))+ scale_x_discrete(limits=orderN)+ theme(axis.title.x = element_blank(),axis.title.y = element_blank()) pX2<-ggplot(data=clusterMeans1, aes(x=Sample, y=X2,group=1)) + geom_line()+ geom_point()+ggtitle(paste(X2 Profile ,clustersizes[2], genes))+ scale_x_discrete(limits=orderN)+ theme(axis.title.x = element_blank(),axis.title.y = element_blank()) pX3<-ggplot(data=clusterMeans1, aes(x=Sample, y=X3,group=1)) + geom_line()+ geom_point()+ggtitle(paste(X3 Profile ,clustersizes[3], genes))+ scale_x_discrete(limits=orderN)+ theme(axis.title.x = element_blank(),axis.title.y = element_blank()) pX4<-ggplot(data=clusterMeans1, aes(x=Sample, y=X4,group=1)) + geom_line()+ geom_point()+ggtitle(paste(X4 Profile ,clustersizes[4], genes))+ scale_x_discrete(limits=orderN)+ theme(axis.title.x = element_blank(),axis.title.y = element_blank()) pX5<-ggplot(data=clusterMeans1, aes(x=Sample, y=X5,group=1)) + geom_line()+ geom_point()+ggtitle(paste(X5 Profile ,clustersizes[5], genes))+ scale_x_discrete(limits=orderN)+ theme(axis.title.x = element_blank(),axis.title.y = element_blank()) pX6<-ggplot(data=clusterMeans1, aes(x=Sample, y=X6,group=1)) + geom_line()+ geom_point()+ggtitle(paste(X6 Profile ,clustersizes[6], genes))+ scale_x_discrete(limits=orderN)+ theme(axis.title.x = element_blank(),axis.title.y = element_blank()) #plot multiplot(pX1, pX2, pX3, pX4,pX5, pX6, cols=2) 
    
 
 
   
 
 
 
 
 
 K-means clustering of means (other treatment means added for inspection) 
 
 
 
  r r split <- factor(paste0(, kclust2$cluster), levels=c(5,6,4,2,1,3)) hmap_k <- Heatmap(dataHMm, split=split, cluster_row_slices = FALSE, cluster_columns = FALSE, show_row_names = FALSE, name = , col = col_fun, width = unit(20, ), column_title = 37TNF, column_title_gp = gpar(fontsize = 10, fontface = )) 
 baseMeansHmTemp <-countsTable[,c(48:50)] colnames(baseMeansHmTemp)<-c(37TNF_0h,37TNF_6h,37TNF_20h) dataHMmR1_37Y<-baseMeansHmTemp[ topDEgenes, ] dataHMmR1_37Y <- log2(dataHMmR1_37Y+1) dataHMmR1_37Y<- t(as.matrix(dataHMmR1_37Y)) dataHMmR1_37Y <- t(scale(dataHMmR1_37Y)) baseMeansHmTemp <-countsTable[,c(60:63)] colnames(baseMeansHmTemp)<-c(14_0h,14_2h,14_6h,14_20h) dataHMmR2_14<-baseMeansHmTemp[ topDEgenes, ] dataHMmR2_14 <- log2(dataHMmR2_14+1) dataHMmR2_14<- t(as.matrix(dataHMmR2_14)) dataHMmR2_14 <- t(scale(dataHMmR2_14)) baseMeansHmTemp <-countsTable[,c(79:82)] colnames(baseMeansHmTemp)<-c(_0h,_2h,_6h,_20h) dataHMmR2_R<-baseMeansHmTemp[ topDEgenes, ] dataHMmR2_R <- log2(dataHMmR2_R+1) dataHMmR2_R<- t(as.matrix(dataHMmR2_R)) dataHMmR2_R <- t(scale(dataHMmR2_R)) baseMeansHmTemp <-countsTable[,c(110:113)] colnames(baseMeansHmTemp)<-c(14TNF_0h,14TNF_2h,14TNF_6h,14TNF_20h) dataHMmR4_14T<-baseMeansHmTemp[ topDEgenes, ] dataHMmR4_14T <- log2(dataHMmR4_14T+1) dataHMmR4_14T<- t(as.matrix(dataHMmR4_14T)) dataHMmR4_14T <- t(scale(dataHMmR4_14T)) baseMeansHmTemp <-countsTable[,c(129:132)] colnames(baseMeansHmTemp)<-c(_TNF_0h,_TNF_2h,_TNF_6h,_TNF_20h) dataHMmR4_RT<-baseMeansHmTemp[ topDEgenes, ] dataHMmR4_RT <- log2(dataHMmR4_RT+1) dataHMmR4_RT<- t(as.matrix(dataHMmR4_RT)) dataHMmR4_RT <- t(scale(dataHMmR4_RT)) hmap_37T <- Heatmap(dataHMmR1_37Y, split=split, name = 37T, 
column_title = 37TNF, cluster_columns = FALSE, show_row_names = FALSE,col = col_fun,width = unit(20, ), column_title_gp = gpar(fontsize = 10, fontface = )) hmap_k14 <- Heatmap(dataHMmR2_14, split=split, name = 14, 
column_title = 14noTNF, cluster_columns = FALSE, show_row_names = FALSE,col = col_fun,width = unit(25, ), column_title_gp = gpar(fontsize = 10, fontface = )) hmap_R <- Heatmap(dataHMmR2_R, split=split, name = , 
column_title = , cluster_columns = FALSE, show_row_names = FALSE,col = col_fun,width = unit(25, ), column_title_gp = gpar(fontsize = 10, fontface = )) hmap_k14T <- Heatmap(dataHMmR4_14T, split=split, name = 14T, 
column_title = 14TNF, cluster_columns = FALSE, show_row_names = FALSE,col = col_fun,width = unit(25, ), column_title_gp = gpar(fontsize = 10, fontface = )) hmap_RT <- Heatmap(dataHMmR4_RT, split=split, name = , 
column_title = , cluster_columns = FALSE, show_row_names = FALSE,col = col_fun,width = unit(25, ), column_title_gp = gpar(fontsize = 10, fontface = )) hmap_k+ha +hmap_k14T+hmap_RT+hmap_k14+hmap_R 
    
 
 
   
 
 
 
 
 
 
  r r topDEgenes <- which(tempA \(Include==\in\)####find indexes tempAkm<-tempA[ topDEgenes, ] SymbolsKm<-dplyr::pull(tempAkm, Gene_Symbol) #### export the gene expression data for the clusters write.table(clusterMeans,paste0(\ClusterMeansKm_\,groupsName,\.txt\), sep = \\t\) ClusteredGenes<-data.frame(kclust2\) cluster,SymbolsKm,dataHMm) write.table(ClusteredGenes,paste0(_,groupsName,.txt), sep = \t) #head(ClusteredGenes) 
    
 
 
 
 
 
 
  bottomDEgenes<-which(tempA$Include=="out")####find indexes 
bottomG<-tempA[ bottomDEgenes, ]
bottomG<-dplyr::pull(bottomG, Gene_Symbol)
write.table(bottomG,paste0("ipaBottomKmeans_",groupsName,".txt"),  sep = "\t")
                         

topDEgenes <- which(tempA$Include=="in")####find indexes 
tempAkm<-tempA[ topDEgenes, ]
SymbolsKm<-dplyr::pull(tempAkm, Gene_Symbol)

ipaKmeans<-ClusteredGenes
#countsTable <-countsTable[,c(1:15)]####if samples need removing
ipaKmeans<-ipaKmeans[,c(1:2)]
ipaKmeans$name2<-rownames(ipaKmeans)
#ipaKmeans%>% rownames_to_column(var = "rowname")
#ipaKmeans
#rowid_to_column(ipaKmeans)
ipaKmeans = mutate(ipaKmeans, x1= ifelse(ipaKmeans$kclust2.cluster==1, "1", "0"))
ipaKmeans = mutate(ipaKmeans, x2= ifelse(ipaKmeans$kclust2.cluster==2, "1", "0"))
ipaKmeans = mutate(ipaKmeans, x3= ifelse(ipaKmeans$kclust2.cluster==3, "1", "0"))
ipaKmeans = mutate(ipaKmeans, x4= ifelse(ipaKmeans$kclust2.cluster==4, "1", "0"))
ipaKmeans = mutate(ipaKmeans, x5= ifelse(ipaKmeans$kclust2.cluster==5, "1", "0"))
ipaKmeans = mutate(ipaKmeans, x6= ifelse(ipaKmeans$kclust2.cluster==6, "1", "0"))
#ipaKmeans
write.table(ipaKmeans,paste0("ipaKmeans_",groupsName,".txt"),  sep = "\t")
#head(ipaKmeans)
  
 
 
 
 
 
 
  r r ClusteredGenes2<-ClusteredGenes[c(1)] #ClusteredGenes2 listAll<-list() for(i in 1:6) { clusterName<-paste0(,i) #clusterName<-row.names(subset(ClusteredGenes,ClusteredGenes==i)) clusterName<-(subset(ClusteredGenes$SymbolsKm,ClusteredGenes==i)) listAll[[i]]<-clusterName } #need to name the vectors in the list, example here is for 8 clusters names(listAll)<-c(1, 2, 3, 4,5, 6) #if you want to rearrange the order #listAll<-listAll[c(3, 7, 8, 2, 6, 5, 4, 1)] #lapply(listAll, head) 
    
 
 
 
 
 
 4. Annotation of K-means clusters 
 
 CC cellular compartment 
 BP biological process 
 MF molecular function 
 
 The simplify function has been used to cut down on GO redundancy 
 
 
 
  r r #str(AllGeneNames) 
    
 
 
 
 
 
 
  r r ####CC cgoCC <- compareCluster(geneCluster = listAll, universe = AllGeneNames, fun = , OrgDb=org.Hs.eg.db, ####OrgDb=org.Mm.eg.db, keyType=, ont = , pvalueCutoff=0.05, qvalueCutoff = 0.10) cgoCC2 <- simplify(cgoCC, cutoff=0.7, by=.adjust, select_fun=min) ####write as spreadsheet write.csv(as.data.frame(cgoCC2),paste0( CC ,groupsName,.csv)) dotplot(cgoCC2,showCategory = 30, title = paste0(Cellular Compartment ,groupsName))+ theme(axis.text.x = element_text(angle = 90, vjust = 0.5, hjust=1)) 
    
 
 
   
 
 
 
 Plots and GO data were written to files 
 
 
 
  r r png(paste0( CC ,groupsName,.png), width = 1224, height = 424) dotplot(cgoCC2,showCategory = 30, title = paste0(Cellular Compartment ,groupsName))+ theme(axis.text.x = element_text(angle = 90, vjust = 0.5, hjust=1)) dev.off() 
    
 
 
  null device 
          1   
 
 
 
 GO BP 
 
 
 
  r r ####CC cgoBP <- compareCluster(geneCluster = listAll, universe = AllGeneNames, fun = , OrgDb=org.Hs.eg.db, keyType=, ont = , pvalueCutoff=0.05, qvalueCutoff = 0.10) cgoBP2 <- simplify(cgoBP, cutoff=0.7, by=.adjust, select_fun=min) ####write as spreadsheet write.csv(as.data.frame(cgoBP2),paste0( BP ,groupsName,.csv)) dotplot(cgoBP2,showCategory = 30, title = paste0(Biological Process ,groupsName))+ theme(axis.text.x = element_text(angle = 90, vjust = 0.5, hjust=1)) 
    
 
 
   
 
 
 
 
 
 
  r r png(paste0( BP ,groupsName,.png), width = 1024, height = 1224) dotplot(cgoBP2,showCategory = 30, title = paste0(Biological Process ,groupsName))+ theme(axis.text.x = element_text(angle = 90, vjust = 0.5, hjust=1)) dev.off() 
    
 
 
  null device 
          1   
 
 
 
 GO MF 
 
 
 
  r r ####MF cgoMF <- compareCluster(geneCluster = listAll, universe = AllGeneNames, fun = , OrgDb=org.Hs.eg.db, keyType=, ont = , pvalueCutoff=0.05, qvalueCutoff = 0.10) cgoMF2 <- simplify(cgoMF, cutoff=0.7, by=.adjust, select_fun=min) ####write as spreadsheet write.csv(as.data.frame(cgoMF2),paste0( MF ,groupsName,.csv)) dotplot(cgoMF2,showCategory = 30, title = paste0(Molecular Function ,groupsName))+ theme(axis.text.x = element_text(angle = 90, vjust = 0.5, hjust=1)) 
    
 
 
   
 
 
 
 
 
 
  r r png(paste0( MF ,groupsName,.png), width = 1424, height = 424) dotplot(cgoMF2,showCategory = 30, title = paste0(Molecular Function ,groupsName))+ theme(axis.text.x = element_text(angle = 90, vjust = 0.5, hjust=1)) dev.off() 
    
 
 
  null device 
          1   
 
 
 
 
 
 
 R2 VAR14 no TNF k-means q0.05 
 
 1. Genelist Selection 
 
 
 
  groupsName<-"R2_VAR14_kmeans_q0.05"  
 
 
 
 
 
 
  countsTable<-read.delim("RNAseq2019July_5.txt", header = TRUE, sep = "\t",check.names=FALSE,row.names=1)
head(countsTable)  
 
 
 
 
 
 
 
 
 
 
 
  AllGeneNames<-countsTable$Gene_Symbol
#head(AllGeneNames)  
 
 
 
 
 
 
  tempA<-countsTable  
 
 
 
 
 
 
  topDEgenes <- which(tempA$padj_R2Var14noTNF_Hours_2h_vs_0h<0.05&!is.na(tempA$padj_R2Var14noTNF_Hours_2h_vs_0h))####find indexes 
listA<-tempA[ topDEgenes, ]$Gene_Symbol
topDEgenes <- which(tempA$padj_R2Var14noTNF_Hours_6h_vs_0h<0.05&!is.na(tempA$padj_R2Var14noTNF_Hours_6h_vs_0h))####find indexes 
listB<-tempA[ topDEgenes, ]$Gene_Symbol
topDEgenes <- which(tempA$padj_R2Var14noTNF_Hours_20h_vs_0h<0.05&!is.na(tempA$padj_R2Var14noTNF_Hours_20h_vs_0h))####find indexes 
listC<-tempA[ topDEgenes, ]$Gene_Symbol
topDEgenes <- which(tempA$padj_R2Var14noTNF_Hours_6h_vs_2h<0.05&!is.na(tempA$padj_R2Var14noTNF_Hours_6h_vs_2h))####find indexes 
listD<-tempA[ topDEgenes, ]$Gene_Symbol
topDEgenes <- which(tempA$padj_R2Var14noTNF_Hours_20h_vs_6h<0.05&!is.na(tempA$padj_R2Var14noTNF_Hours_20h_vs_6h))####find indexes 
listE<-tempA[ topDEgenes, ]$Gene_Symbol
vennq<-venn.diagram(x = list(listA,listB,listC,listD,listE) ,
            category.names = c("Var14noTNF_2h_vs_0h","Var14noTNF_6h_vs_0h","Var14noTNF_20h_vs_0h","Var14noTNF_6h_vs_2h","Var14noTNF_20h_vs_6h"),
            main="padj<0.05",
            filename = NULL,  scaled = FALSE, fill = colorsV5, cat.col = colorsV5, cat.cex = 1, cat.dist=0.3,  margin = 0.3)
topDEgenes <- which(tempA$pvalue_R2Var14noTNF_Hours_2h_vs_0h<0.05&abs(tempA$log2FoldChange_R2Var14noTNF_Hours_2h_vs_0h)>1&!is.na(tempA$pvalue_R2Var14noTNF_Hours_2h_vs_0h))####find indexes 
listA<-tempA[ topDEgenes, ]$Gene_Symbol
topDEgenes <- which(tempA$pvalue_R2Var14noTNF_Hours_6h_vs_0h<0.05&abs(tempA$log2FoldChange_R2Var14noTNF_Hours_6h_vs_0h)>1&!is.na(tempA$pvalue_R2Var14noTNF_Hours_6h_vs_0h))####find indexes 
listB<-tempA[ topDEgenes, ]$Gene_Symbol
topDEgenes <- which(tempA$pvalue_R2Var14noTNF_Hours_20h_vs_0h<0.05&abs(tempA$log2FoldChange_R2Var14noTNF_Hours_20h_vs_0h)>1&!is.na(tempA$pvalue_R2Var14noTNF_Hours_20h_vs_0h))####find indexes 
listC<-tempA[ topDEgenes, ]$Gene_Symbol
topDEgenes <- which(tempA$pvalue_R2Var14noTNF_Hours_6h_vs_2h<0.05&abs(tempA$log2FoldChange_R2Var14noTNF_Hours_6h_vs_2h)>1&!is.na(tempA$pvalue_R2Var14noTNF_Hours_6h_vs_2h))####find indexes 
listD<-tempA[ topDEgenes, ]$Gene_Symbol
topDEgenes <- which(tempA$pvalue_R2Var14noTNF_Hours_20h_vs_6h<0.05&abs(tempA$log2FoldChange_R2Var14noTNF_Hours_20h_vs_6h)>1&!is.na(tempA$pvalue_R2Var14noTNF_Hours_20h_vs_6h))####find indexes 
listE<-tempA[ topDEgenes, ]$Gene_Symbol
vennp<-venn.diagram(x = list(listA,listB,listC,listD,listE) ,
            category.names = c("Var14noTNF_2h_vs_0h","Var14noTNF_6h_vs_0h","Var14noTNF_20h_vs_0h","Var14noTNF_6h_vs_2h","Var14noTNF_20h_vs_6h"),
            main="pvalue<0.05&fold change>2",
            filename = NULL,  scaled = FALSE, fill = colorsV5, cat.col = colorsV5, cat.cex = 1, cat.dist=0.3,  margin = 0.3)  
 
 
 
 
 
 
  topDEgenes <- which((tempA$padj_R2Var14noTNF_Hours_2h_vs_0h<0.05&!is.na(tempA$padj_R2Var14noTNF_Hours_2h_vs_0h))| 
(tempA$padj_R2Var14noTNF_Hours_6h_vs_0h<0.05&!is.na(tempA$padj_R2Var14noTNF_Hours_6h_vs_0h))|
(tempA$padj_R2Var14noTNF_Hours_20h_vs_0h<0.05&!is.na(tempA$padj_R2Var14noTNF_Hours_20h_vs_0h))| 
(tempA$padj_R2Var14noTNF_Hours_6h_vs_2h<0.05&!is.na(tempA$padj_R2Var14noTNF_Hours_6h_vs_2h))|
(tempA$padj_R2Var14noTNF_Hours_20h_vs_6h<0.05&!is.na(tempA$padj_R2Var14noTNF_Hours_20h_vs_6h)) 
)
listA<-tempA[ topDEgenes, ]$Gene_Symbol
topDEgenes <- which((tempA$pvalue_R2Var14noTNF_Hours_2h_vs_0h<0.05&abs(tempA$log2FoldChange_R2Var14noTNF_Hours_2h_vs_0h)>1&!is.na(tempA$pvalue_R2Var14noTNF_Hours_2h_vs_0h))| 
(tempA$pvalue_R2Var14noTNF_Hours_6h_vs_0h<0.05&abs(tempA$log2FoldChange_R2Var14noTNF_Hours_6h_vs_0h)>1&!is.na(tempA$pvalue_R2Var14noTNF_Hours_6h_vs_0h))| 
(tempA$pvalue_R2Var14noTNF_Hours_20h_vs_0h<0.05&abs(tempA$log2FoldChange_R2Var14noTNF_Hours_20h_vs_0h)>1&!is.na(tempA$pvalue_R2Var14noTNF_Hours_20h_vs_0h))| 
(tempA$pvalue_R2Var14noTNF_Hours_6h_vs_2h<0.05&abs(tempA$log2FoldChange_R2Var14noTNF_Hours_6h_vs_2h)>1&!is.na(tempA$pvalue_R2Var14noTNF_Hours_6h_vs_2h))| 
(tempA$pvalue_R2Var14noTNF_Hours_20h_vs_6h<0.05&abs(tempA$log2FoldChange_R2Var14noTNF_Hours_20h_vs_6h)>1&!is.na(tempA$pvalue_R2Var14noTNF_Hours_20h_vs_6h))
 )####find indexes 
listC<-tempA[ topDEgenes, ]$Gene_Symbol
vennpq<-venn.diagram(x = list(listA,listC) ,
            category.names = c("padj<0.05","p<0.05&fc>2"),
            main="padj compared to pvalue",
            filename = NULL,  scaled = FALSE, fill = colorsV2, cat.col = colorsV2, cat.cex = 1, cat.dist=0.1,  margin = 0.15)  
 
 
 
 
 
 
  grid.arrange(gTree(children=vennq), gTree(children=vennpq) , ncol=2,top="R2 Var14 no TNF")  
 
 
   
 
 
 
 
 
 
  #tempA<-resAll[-c(10:30) ]
tempA<-countsTable
#rownames(tempA)
rownames(tempA) <- NULL
tempA = mutate(tempA, Include=
                   ifelse(tempA$padj_R2Var14noTNF_Hours_2h_vs_0h<0.05&!is.na(tempA$padj_R2Var14noTNF_Hours_2h_vs_0h), "in",
                          ifelse(tempA$padj_R2Var14noTNF_Hours_6h_vs_0h<0.05&!is.na(tempA$padj_R2Var14noTNF_Hours_6h_vs_0h), "in",
                                 ifelse(tempA$padj_R2Var14noTNF_Hours_20h_vs_0h<0.05&!is.na(tempA$padj_R2Var14noTNF_Hours_20h_vs_0h), "in",
                                        ifelse(tempA$padj_R2Var14noTNF_Hours_6h_vs_2h<0.05&!is.na(tempA$padj_R2Var14noTNF_Hours_6h_vs_2h), "in",
                                               ifelse(tempA$padj_R2Var14noTNF_Hours_20h_vs_6h<0.05&!is.na(tempA$padj_R2Var14noTNF_Hours_20h_vs_6h), "in",
                                                                       "out"))))))
tempA  
 
 
 
 
 
 
 
  ####library(dplyr)
tempA %>%
     group_by(Include) %>% 
     tally()  
 
 
 
 
 
 
 
 
 
 
 
  topDEgenes <- which(tempA$Include=="in")####find indexes   
 
 
 
 
 
 NB Please check columns used and renamed for plots 
 
 
 
  baseMeansHm <-countsTable[,c(60:63)]
head(baseMeansHm)  
 
 
 
 
 
 
 
  colnames(baseMeansHm)<-c("Var14_0h","Var14_2h","Var14_6h","Var14_20h")
head(baseMeansHm)  
 
 
 
 
 
 
 
 
 
  dataHi <-countsTable[,c(2:9)]
head(dataHi)  
 
 
 
 
 
 
 
 
 
 
 
  dataHi <-countsTable[,c(2:9)]
colnames(dataHi)<-c("Var14_0h_2","Var14_0h_3","Var14_2h_2","Var14_2h_3","Var14_6h_2","Var14_6h_3","Var14_20h_2","Var14_20h_3")
head(dataHi)  
 
 
 
 
 
 
 
  dataHi<-dataHi[ topDEgenes, ]
dataHi <- log2(dataHi+1)
dataHi<- t(as.matrix(dataHi))
dataHi <- t(scale(dataHi))
####str(dataHi)  
 
 
 
 
 
 
  topDEgenes <- which(tempA$Include=="in")####find indexes   
 
 
 
 
 
 2. Hierachical clustering of means (individual samples added for inspection) 
 
 
 
  hmap_hier_factors1 <- Heatmap(
  dataHi,  name = "ExpressionI",
  column_title = paste0("Individual Samples"), 
  column_title_gp = gpar(fontsize = 16, fontface = "bold"),
  width = unit(300, "mm"),
  col = col_fun,
  cluster_rows = FALSE,
  cluster_columns = FALSE,
  show_row_names = FALSE)
  #top_annotation=colAnn  )
####means
dataHMm<-baseMeansHm[ topDEgenes, ]
dataHMm <- log2(dataHMm+1)
dataHMm<- t(as.matrix(dataHMm))
dataHMm <- t(scale(dataHMm))
#colAnnm <- HeatmapAnnotation(df=annm, which="col", col=coloursm, annotation_width=unit(c(2, 4), "cm"), gap=unit(1, "mm"))
hmap_hier_factors4 <- Heatmap(
  dataHMm,  name = "Expression",
  row_labels = paste0(rownames(dataHMm)," ",(tempA[ topDEgenes, ])$Gene_Symbol),
  column_title = paste0("Means"), 
  col = col_fun,
  column_title_gp = gpar(fontsize = 16, fontface = "bold"),
  width = unit(50, "mm"),
  cluster_columns = FALSE,
  show_row_names = FALSE)
  #top_annotation=colAnnm  )
hmap_hier_factors4+hmap_hier_factors1  
 
 
   
 
 
 
 
 
 
  r r par(mfrow=c(1,2)) #### Silhouette method fviz_nbclust(dataHMm, kmeans, method = ,k.max = 16)+ labs(subtitle = method) 
    
 
 
  did not converge in 10 iterations  
 
 
   
 
 
  r r #### Elbow method fviz_nbclust(dataHMm, kmeans, method = ,k.max = 16) + labs(subtitle = method) 
    
 
 
   
 
 
 
 
 
 
  r r ####gap stat slow!!! ####set.seed(123) ####fviz_nbclust(dataHMm, kmeans, nstart = 25, method = _stat, nboot = 100,k.max = 16)+ #### labs(subtitle = statistic method) 
    
 
 
 
 
 
 
  r r #kclust3 <- kmeans(dataHMm, 6) #silhouette plot distK<-daisy(dataHMm) plot(silhouette(kclust3$cluster, distK), col=1:6, border=NA) 
    
 
 
   
 
 
 
 
 
 3. K-means clustering of means 
 
 
 
  #split <- paste0("Cluster\n", kclust3$cluster)
split <- factor(paste0("Cluster\n", kclust3$cluster), levels=c("Cluster\n3","Cluster\n1","Cluster\n4","Cluster\n5","Cluster\n2","Cluster\n6"))
hmap_k <- Heatmap(dataHMm, split=split, cluster_row_slices = FALSE,
                  cluster_columns = FALSE,
                  show_row_names = FALSE,
                  name = "Expression",
                  col = col_fun,
                  width = unit(20, "mm"),
                  column_title = "means", 
                  column_title_gp = gpar(fontsize = 16, fontface = "bold")
                  
                        )#top_annotation=colAnn)
hmap_hier_factors1 <- Heatmap(
  dataHi,  name = "ExpressionI",
  col = col_fun,
  column_title = paste0("individual samples"), 
  column_title_gp = gpar(fontsize = 16, fontface = "bold"),
  width = unit(60, "mm"),
  cluster_rows = FALSE,
  cluster_columns = FALSE,
  show_row_names = FALSE)
hmap_k  
 
 
   
 
 
 
 
 
 K-means clustering of means (with cluster annotation and individual samples added for inspection) 
 
 
 
  Response_Time<-data.frame(kclust3$cluster)
Response_Time = mutate(Response_Time, Response=
                   ifelse(Response_Time$kclust3.cluster==3, "early", 
                          ifelse(Response_Time$kclust3.cluster==1, "late",
                                 ifelse(Response_Time$kclust3.cluster==4, "transient",
                                        ifelse(Response_Time$kclust3.cluster==5, "transient",
                                               ifelse(Response_Time$kclust3.cluster==2, "early",
                                                      ifelse(Response_Time$kclust3.cluster==6, "late",
                                                                       "out")))))))
Response_Time<-Response_Time[c(2)]
rownames(Response_Time) <- NULL
ha = HeatmapAnnotation(df = Response_Time, which = "row", width = unit(1, "cm"),col = list(Response = c("early" =  "green3", "late" = "brown", "transient" = "violet")))
hmap_k+ha+hmap_hier_factors1  
 
 
   
 
 
 
 Mean profiles of clusters 
 
 
 
  clustercount<-data.frame(kclust3$cluster)
clustersizes<-table(clustercount$kclust3.cluster)
clusterMeans<-data.frame(kclust3$centers)
clusterMeans1<-data.frame(t(clusterMeans))
clusterMeans1 <- cbind(rownames(clusterMeans1), clusterMeans1)
orderN<-c("Var14_0h","Var14_2h","Var14_6h","Var14_20h")#### manual
rownames(clusterMeans1) <- NULL
names(clusterMeans1)[names(clusterMeans1)=="rownames(clusterMeans1)"] <- "Sample"
####clusterMeans1
pX1<-ggplot(data=clusterMeans1, aes(x=Sample, y=X1,group=1)) +
  geom_line()+  geom_point()+ggtitle(paste("Cluster X1 Profile ",clustersizes[1]," genes"))+  scale_x_discrete(limits=orderN)+
  theme(axis.title.x = element_blank(),axis.title.y = element_blank())
pX2<-ggplot(data=clusterMeans1, aes(x=Sample, y=X2,group=1)) +
  geom_line()+  geom_point()+ggtitle(paste("Cluster X2 Profile ",clustersizes[2]," genes"))+  scale_x_discrete(limits=orderN)+
  theme(axis.title.x = element_blank(),axis.title.y = element_blank())
pX3<-ggplot(data=clusterMeans1, aes(x=Sample, y=X3,group=1)) +
  geom_line()+  geom_point()+ggtitle(paste("Cluster X3 Profile ",clustersizes[3]," genes"))+  scale_x_discrete(limits=orderN)+
  theme(axis.title.x = element_blank(),axis.title.y = element_blank())
pX4<-ggplot(data=clusterMeans1, aes(x=Sample, y=X4,group=1)) +
  geom_line()+  geom_point()+ggtitle(paste("Cluster X4 Profile ",clustersizes[4]," genes"))+  scale_x_discrete(limits=orderN)+
  theme(axis.title.x = element_blank(),axis.title.y = element_blank())
pX5<-ggplot(data=clusterMeans1, aes(x=Sample, y=X5,group=1)) +
  geom_line()+  geom_point()+ggtitle(paste("Cluster X5 Profile ",clustersizes[5]," genes"))+  scale_x_discrete(limits=orderN)+
  theme(axis.title.x = element_blank(),axis.title.y = element_blank())
pX6<-ggplot(data=clusterMeans1, aes(x=Sample, y=X6,group=1)) +
  geom_line()+  geom_point()+ggtitle(paste("Cluster X6 Profile ",clustersizes[6]," genes"))+  scale_x_discrete(limits=orderN)+
  theme(axis.title.x = element_blank(),axis.title.y = element_blank())
#plot
multiplot(pX1, pX2, pX3, pX4,pX5, pX6, cols=2)  
 
 
   
 
 
 
 
 
 K-means clustering of means (other treatment means added for inspection) 
 
 
 
  split <- factor(paste0("Cluster\n", kclust3$cluster), levels=c("Cluster\n5","Cluster\n6","Cluster\n4","Cluster\n2","Cluster\n1","Cluster\n3"))
hmap_k <- Heatmap(dataHMm, split=split, cluster_row_slices = FALSE,
                  cluster_columns = FALSE,
                  show_row_names = FALSE,
                  name = "Expression",
                  col = col_fun,
                  width = unit(25, "mm"),
                  column_title = "Var14noTNF", 
                  column_title_gp = gpar(fontsize = 10, fontface = "bold"))
                  
                  
baseMeansHmTemp <-countsTable[,c(48:50)]
colnames(baseMeansHmTemp)<-c("Var37TNF_0h","Var37TNF_6h","Var37TNF_20h")
dataHMmR1_37Y<-baseMeansHmTemp[ topDEgenes, ]
dataHMmR1_37Y <- log2(dataHMmR1_37Y+1)
dataHMmR1_37Y<- t(as.matrix(dataHMmR1_37Y))
dataHMmR1_37Y <- t(scale(dataHMmR1_37Y))
baseMeansHmTemp <-countsTable[,c(60:63)]
colnames(baseMeansHmTemp)<-c("Var14_0h","Var14_2h","Var14_6h","Var14_20h")
dataHMmR2_14<-baseMeansHmTemp[ topDEgenes, ]
dataHMmR2_14 <- log2(dataHMmR2_14+1)
dataHMmR2_14<- t(as.matrix(dataHMmR2_14))
dataHMmR2_14 <- t(scale(dataHMmR2_14))
baseMeansHmTemp <-countsTable[,c(79:82)]
colnames(baseMeansHmTemp)<-c("RBC_0h","RBC_2h","RBC_6h","RBC_20h")
dataHMmR2_R<-baseMeansHmTemp[ topDEgenes, ]
dataHMmR2_R <- log2(dataHMmR2_R+1)
dataHMmR2_R<- t(as.matrix(dataHMmR2_R))
dataHMmR2_R <- t(scale(dataHMmR2_R))
baseMeansHmTemp <-countsTable[,c(110:113)]
colnames(baseMeansHmTemp)<-c("Var14TNF_0h","Var14TNF_2h","Var14TNF_6h","Var14TNF_20h")
dataHMmR4_14T<-baseMeansHmTemp[ topDEgenes, ]
dataHMmR4_14T <- log2(dataHMmR4_14T+1)
dataHMmR4_14T<- t(as.matrix(dataHMmR4_14T))
dataHMmR4_14T <- t(scale(dataHMmR4_14T))
baseMeansHmTemp <-countsTable[,c(129:132)]
colnames(baseMeansHmTemp)<-c("RBC_TNF_0h","RBC_TNF_2h","RBC_TNF_6h","RBC_TNF_20h")
dataHMmR4_RT<-baseMeansHmTemp[ topDEgenes, ]
dataHMmR4_RT <- log2(dataHMmR4_RT+1)
dataHMmR4_RT<- t(as.matrix(dataHMmR4_RT))
dataHMmR4_RT <- t(scale(dataHMmR4_RT))
hmap_37T <- Heatmap(dataHMmR1_37Y, split=split, 
                  name = "Expression37T",  
                  column_title = "VAR37TNF", 
                  cluster_columns = FALSE,  show_row_names = FALSE,col = col_fun,width = unit(20, "mm"),
                  column_title_gp = gpar(fontsize = 10, fontface = "bold"))
hmap_k14 <- Heatmap(dataHMmR2_14, split=split, 
                  name = "Expression14",  
                  column_title = "VAR14noTNF", 
                  cluster_columns = FALSE,  show_row_names = FALSE,col = col_fun,width = unit(25, "mm"),
                  column_title_gp = gpar(fontsize = 10, fontface = "bold"))
hmap_R <- Heatmap(dataHMmR2_R, split=split, 
                  name = "ExpressionR",  
                  column_title = "RBCnoTNF", 
                  cluster_columns = FALSE,  show_row_names = FALSE,col = col_fun,width = unit(25, "mm"),
                  column_title_gp = gpar(fontsize = 10, fontface = "bold"))
hmap_k14T <- Heatmap(dataHMmR4_14T, split=split, 
                  name = "Expression14T",  
                  column_title = "VAR14TNF", 
                  cluster_columns = FALSE,  show_row_names = FALSE,col = col_fun,width = unit(25, "mm"),
                  column_title_gp = gpar(fontsize = 10, fontface = "bold"))
hmap_RT <- Heatmap(dataHMmR4_RT, split=split, 
                  name = "ExpressionRT",  
                  column_title = "RBCTNF", 
                  cluster_columns = FALSE,  show_row_names = FALSE,col = col_fun,width = unit(25, "mm"),
                  column_title_gp = gpar(fontsize = 10, fontface = "bold"))
hmap_k+ha+hmap_R+hmap_RT+hmap_k14T+hmap_37T  
 
 
   
 
 
 
 
 
 
  topDEgenes <- which(tempA$Include=="in")####find indexes
tempAkm<-tempA[ topDEgenes, ]
SymbolsKm<-dplyr::pull(tempAkm, Gene_Symbol)
#### export the gene expression data for the clusters
write.table(clusterMeans,paste0("ClusterMeansKm_",groupsName,".txt"),  sep = "\t")
ClusteredGenes<-data.frame(kclust3$cluster,SymbolsKm,dataHMm)
write.table(ClusteredGenes,paste0("ScaledDataInClustersKm_",groupsName,".txt"),  sep = "\t")
#head(ClusteredGenes)  
 
 
 
 
 
 
  bottomDEgenes<-which(tempA$Include=="out")####find indexes 
bottomG<-tempA[ bottomDEgenes, ]
bottomG<-dplyr::pull(bottomG, Gene_Symbol)
write.table(bottomG,paste0("ipaBottomKmeans_",groupsName,".txt"),  sep = "\t")
                         
topDEgenes <- which(tempA$Include=="in")####find indexes 
tempAkm<-tempA[ topDEgenes, ]
SymbolsKm<-dplyr::pull(tempAkm, Gene_Symbol)
ipaKmeans<-ClusteredGenes
#countsTable <-countsTable[,c(1:15)]####if samples need removing
ipaKmeans<-ipaKmeans[,c(1:2)]
ipaKmeans$name2<-rownames(ipaKmeans)
#ipaKmeans%>% rownames_to_column(var = "rowname")
#ipaKmeans
#rowid_to_column(ipaKmeans)
ipaKmeans = mutate(ipaKmeans, x1= ifelse(ipaKmeans$kclust3.cluster==1, "1", "0"))
ipaKmeans = mutate(ipaKmeans, x2= ifelse(ipaKmeans$kclust3.cluster==2, "1", "0"))
ipaKmeans = mutate(ipaKmeans, x3= ifelse(ipaKmeans$kclust3.cluster==3, "1", "0"))
ipaKmeans = mutate(ipaKmeans, x4= ifelse(ipaKmeans$kclust3.cluster==4, "1", "0"))
ipaKmeans = mutate(ipaKmeans, x5= ifelse(ipaKmeans$kclust3.cluster==5, "1", "0"))
ipaKmeans = mutate(ipaKmeans, x6= ifelse(ipaKmeans$kclust3.cluster==6, "1", "0"))
#ipaKmeans
write.table(ipaKmeans,paste0("ipaKmeans_",groupsName,".txt"),  sep = "\t")
#head(ipaKmeans)  
 
 
 
 
 
 
  ClusteredGenes2<-ClusteredGenes[c(1)]
#ClusteredGenes2
listAll<-list()
for(i in 1:6) {
  clusterName<-paste0("x",i)
  #clusterName<-row.names(subset(ClusteredGenes,ClusteredGenes==i))
  clusterName<-(subset(ClusteredGenes$SymbolsKm,ClusteredGenes==i))
  listAll[[i]]<-clusterName
}
#need to name the vectors in the list, example here is for 8 clusters
names(listAll)<-c("X1", "X2", "X3", "X4","X5", "X6")
#if you want to rearrange the order
#listAll<-listAll[c("x3", "x7", "x8", "x2", "x6", "x5", "x4", "x1")]
#lapply(listAll, head)  
 
 
 
 
 
 4. Annotation of K-means clusters 
 
 CC cellular compartment 
 BP biological process 
 MF molecular function 
 
 The simplify function has been used to cut down on GO redundancy 
 
 
 
  #str(AllGeneNames)  
 
 
 
 
 
 
  ####CC
cgoCC <- compareCluster(geneCluster = listAll, 
                      universe = AllGeneNames,
                      fun = "enrichGO",
                      OrgDb=org.Hs.eg.db, 
                      ####OrgDb=org.Mm.eg.db,
                      keyType="SYMBOL",
                      ont = "CC", 
                      pvalueCutoff=0.05,
                      qvalueCutoff = 0.10)
cgoCC2 <- simplify(cgoCC, cutoff=0.7, by="p.adjust", select_fun=min)
####write as spreadsheet
write.csv(as.data.frame(cgoCC2),paste0("GO_CC_",groupsName,".csv"))
dotplot(cgoCC2,showCategory = 30,
        title = paste0("GO Cellular Compartment ",groupsName))+
  theme(axis.text.x = element_text(angle = 90, vjust = 0.5, hjust=1))  
 
 
   
 
 
 
 Plots and GO data were written to files 
 
 
 
  png(paste0("GO_CC_",groupsName,".png"), width = 1224, height = 824)
dotplot(cgoCC2,showCategory = 30,
        title = paste0("GO Cellular Compartment ",groupsName))+
  theme(axis.text.x = element_text(angle = 90, vjust = 0.5, hjust=1))
dev.off()  
 
 
  null device 
          1   
 
 
 
 GO BP 
 
 
 
  ####CC
cgoBP <- compareCluster(geneCluster = listAll, 
                      universe = AllGeneNames,
                      fun = "enrichGO",
                      OrgDb=org.Hs.eg.db,
                      keyType="SYMBOL",
                      ont = "BP", 
                      pvalueCutoff=0.05,
                      qvalueCutoff = 0.10)
cgoBP2 <- simplify(cgoBP, cutoff=0.7, by="p.adjust", select_fun=min)
####write as spreadsheet
write.csv(as.data.frame(cgoBP2),paste0("GO_BP_",groupsName,".csv"))
dotplot(cgoBP2,showCategory = 30,
        title = paste0("GO Biological Process ",groupsName))+
  theme(axis.text.x = element_text(angle = 90, vjust = 0.5, hjust=1))  
 
 
   
 
 
 
 
 
 
  png(paste0("GO_BP_",groupsName,".png"), width = 1024, height = 1224)
dotplot(cgoBP2,showCategory = 30,
        title = paste0("GO Biological Process ",groupsName))+
  theme(axis.text.x = element_text(angle = 90, vjust = 0.5, hjust=1))
dev.off()  
 
 
  null device 
          1   
 
 
 
 GO MF 
 
 
 
  ####MF
cgoMF <- compareCluster(geneCluster = listAll, 
                      universe = AllGeneNames,
                      fun = "enrichGO",
                      OrgDb=org.Hs.eg.db, 
                      keyType="SYMBOL",
                      ont = "MF", 
                      pvalueCutoff=0.05,
                      qvalueCutoff = 0.10)
cgoMF2 <- simplify(cgoMF, cutoff=0.7, by="p.adjust", select_fun=min)
####write as spreadsheet
write.csv(as.data.frame(cgoMF2),paste0("GO_MF_",groupsName,".csv"))
dotplot(cgoMF2,showCategory = 30,
        title = paste0("GO Molecular Function  ",groupsName))+
  theme(axis.text.x = element_text(angle = 90, vjust = 0.5, hjust=1))  
 
 
   
 
 
 
 
 
 
  png(paste0("GO_MF_",groupsName,".png"), width = 1424, height = 824)
dotplot(cgoMF2,showCategory = 30,
        title = paste0("GO Molecular Function  ",groupsName))+
  theme(axis.text.x = element_text(angle = 90, vjust = 0.5, hjust=1))
dev.off()  
 
 
  null device 
          1   
 
 
 
 
 
 
 R2 VAR14 no TNF k-means p0.05fc2 
 
 1. Genelist Selection 
 
 
 
  groupsName<-"R2_VAR14_kmeans_p0.05fc2"  
 
 
 
 
 
 
  countsTable<-read.delim("RNAseq2019July_5.txt", header = TRUE, sep = "\t",check.names=FALSE,row.names=1)
head(countsTable)  
 
 
 
 
 
 
 
 
 
 
 
  AllGeneNames<-countsTable$Gene_Symbol
#head(AllGeneNames)  
 
 
 
 
 
 
  grid.arrange(gTree(children=vennp), gTree(children=vennpq) , ncol=2,top="R2 Var14 no TNF")  
 
 
   
 
 
 
 
 
 
  #tempA<-resAll[-c(10:30) ]
tempA<-countsTable
#rownames(tempA)
rownames(tempA) <- NULL
tempA = mutate(tempA, Include=
                   ifelse(tempA$pvalue_R2Var14noTNF_Hours_2h_vs_0h<0.05&abs(tempA$log2FoldChange_R2Var14noTNF_Hours_2h_vs_0h)>1&!is.na(tempA$pvalue_R2Var14noTNF_Hours_2h_vs_0h), "in",
                          ifelse(tempA$pvalue_R2Var14noTNF_Hours_6h_vs_0h<0.05&abs(tempA$log2FoldChange_R2Var14noTNF_Hours_6h_vs_0h)>1&!is.na(tempA$pvalue_R2Var14noTNF_Hours_6h_vs_0h), "in",
                                 ifelse(tempA$pvalue_R2Var14noTNF_Hours_20h_vs_0h<0.05&abs(tempA$log2FoldChange_R2Var14noTNF_Hours_20h_vs_0h)>1&!is.na(tempA$pvalue_R2Var14noTNF_Hours_20h_vs_0h), "in",
                                        ifelse(tempA$pvalue_R2Var14noTNF_Hours_6h_vs_2h<0.05&abs(tempA$log2FoldChange_R2Var14noTNF_Hours_6h_vs_2h)>1&!is.na(tempA$pvalue_R2Var14noTNF_Hours_6h_vs_2h), "in",
                                               ifelse(tempA$pvalue_R2Var14noTNF_Hours_20h_vs_6h<0.05&abs(tempA$log2FoldChange_R2Var14noTNF_Hours_20h_vs_6h)>1&!is.na(tempA$pvalue_R2Var14noTNF_Hours_20h_vs_6h), "in",
                                                                       "out"))))))
tempA  
 
 
 
 
 
 
 
  ####library(dplyr)
tempA %>%
     group_by(Include) %>% 
     tally()  
 
 
 
 
 
 
 
 
 
 
 
  topDEgenes <- which(tempA$Include=="in")####find indexes   
 
 
 
 
 
 NB Please check columns used and renamed for plots 
 
 
 
  baseMeansHm <-countsTable[,c(60:63)]
head(baseMeansHm)  
 
 
 
 
 
 
 
  colnames(baseMeansHm)<-c("Var14_0h","Var14_2h","Var14_6h","Var14_20h")
head(baseMeansHm)  
 
 
 
 
 
 
 
 
 
 
 
  dataHi <-countsTable[,c(2:9)]
head(dataHi)  
 
 
 
 
 
 
 
 
 
 
 
  dataHi <-countsTable[,c(2:9)]
colnames(dataHi)<-c("Var14_0h_2","Var14_0h_3","Var14_2h_2","Var14_2h_3","Var14_6h_2","Var14_6h_3","Var14_20h_2","Var14_20h_3")
head(dataHi)  
 
 
 
 
 
 
 
  dataHi<-dataHi[ topDEgenes, ]
dataHi <- log2(dataHi+1)
dataHi<- t(as.matrix(dataHi))
dataHi <- t(scale(dataHi))
####str(dataHi)  
 
 
 
 
 
 
  topDEgenes <- which(tempA$Include=="in")####find indexes   
 
 
 
 
 
 2. Hierachical clustering of means (individual samples added for inspection) 
 
 
 
  hmap_hier_factors1 <- Heatmap(
  dataHi,  name = "ExpressionI",
  column_title = paste0("Individual Samples"), 
  column_title_gp = gpar(fontsize = 16, fontface = "bold"),
  width = unit(300, "mm"),
  col = col_fun,
  cluster_rows = FALSE,
  cluster_columns = FALSE,
  show_row_names = FALSE)
  #top_annotation=colAnn  )
####means
dataHMm<-baseMeansHm[ topDEgenes, ]
dataHMm <- log2(dataHMm+1)
dataHMm<- t(as.matrix(dataHMm))
dataHMm <- t(scale(dataHMm))
#colAnnm <- HeatmapAnnotation(df=annm, which="col", col=coloursm, annotation_width=unit(c(2, 4), "cm"), gap=unit(1, "mm"))
hmap_hier_factors4 <- Heatmap(
  dataHMm,  name = "Expression",
  row_labels = paste0(rownames(dataHMm)," ",(tempA[ topDEgenes, ])$Gene_Symbol),
  column_title = paste0("Means"), 
  col = col_fun,
  column_title_gp = gpar(fontsize = 16, fontface = "bold"),
  width = unit(50, "mm"),
  cluster_columns = FALSE,
  show_row_names = FALSE)
  #top_annotation=colAnnm  )
hmap_hier_factors4+hmap_hier_factors1  
 
 
   
 
 
 
 
 
 
  par(mfrow=c(1,2))
#### Silhouette method
fviz_nbclust(dataHMm, kmeans, method = "silhouette",k.max = 16)+
  labs(subtitle = "Silhouette method")  
 
 
   
 
 
  #### Elbow method
fviz_nbclust(dataHMm, kmeans, method = "wss",k.max = 16) +
  labs(subtitle = "Elbow method")  
 
 
   
 
 
 
 
 
 
  ####gap stat slow!!!
####set.seed(123)
####fviz_nbclust(dataHMm, kmeans, nstart = 25,  method = "gap_stat", nboot = 100,k.max = 16)+
####  labs(subtitle = "Gap statistic method")  
 
 
 
 
 
 
  #kclust4 <- kmeans(dataHMm, 6)
#silhouette plot
distK<-daisy(dataHMm)
plot(silhouette(kclust4$cluster, distK), col=1:6, border=NA)  
 
 
   
 
 
 
 
 
 3. K-means clustering of means 
 
 
 
  #split <- paste0("Cluster\n", kclust8$cluster)
split <- factor(paste0("Cluster\n", kclust4$cluster), levels=c("Cluster\n2","Cluster\n1","Cluster\n5","Cluster\n6","Cluster\n3","Cluster\n4"))
hmap_k <- Heatmap(dataHMm, split=split, cluster_row_slices = FALSE,
                  cluster_columns = FALSE,
                  show_row_names = FALSE,
                  name = "Expression",
                  col = col_fun,
                  width = unit(20, "mm"),
                  column_title = "means", 
                  column_title_gp = gpar(fontsize = 16, fontface = "bold")
                  
                        )#top_annotation=colAnn)
hmap_hier_factors1 <- Heatmap(
  dataHi,  name = "ExpressionI",
  col = col_fun,
  column_title = paste0("individual samples"), 
  column_title_gp = gpar(fontsize = 16, fontface = "bold"),
  width = unit(60, "mm"),
  cluster_rows = FALSE,
  cluster_columns = FALSE,
  show_row_names = FALSE)
hmap_k  
 
 
   
 
 
 
 
 
 K-means clustering of means (with cluster annotation and individual samples added for inspection) 
 
 
 
  Response_Time<-data.frame(kclust4$cluster)
Response_Time = mutate(Response_Time, Response=
                   ifelse(Response_Time$kclust4.cluster==3, "early", 
                          ifelse(Response_Time$kclust4.cluster==1, "late",
                                 ifelse(Response_Time$kclust4.cluster==4, "late",
                                        ifelse(Response_Time$kclust4.cluster==5, "transient",
                                               ifelse(Response_Time$kclust4.cluster==2, "early",
                                                      ifelse(Response_Time$kclust4.cluster==6, "transient",
                                                                       "out")))))))
Response_Time<-Response_Time[c(2)]
rownames(Response_Time) <- NULL
ha = HeatmapAnnotation(df = Response_Time, which = "row", width = unit(1, "cm"),col = list(Response = c("early" =  "green3", "late" = "brown", "transient" = "violet")))
hmap_k+ha+hmap_hier_factors1  
 
 
   
 
 
 
 Mean profiles of clusters 
 
 
 
  clustercount<-data.frame(kclust4$cluster)
clustersizes<-table(clustercount$kclust4.cluster)
clusterMeans<-data.frame(kclust4$centers)
clusterMeans1<-data.frame(t(clusterMeans))
clusterMeans1 <- cbind(rownames(clusterMeans1), clusterMeans1)
orderN<-c("Var14_0h","Var14_2h","Var14_6h","Var14_20h")#### manual
rownames(clusterMeans1) <- NULL
names(clusterMeans1)[names(clusterMeans1)=="rownames(clusterMeans1)"] <- "Sample"
####clusterMeans1
pX1<-ggplot(data=clusterMeans1, aes(x=Sample, y=X1,group=1)) +
  geom_line()+  geom_point()+ggtitle(paste("Cluster X1 Profile ",clustersizes[1]," genes"))+  scale_x_discrete(limits=orderN)+
  theme(axis.title.x = element_blank(),axis.title.y = element_blank())
pX2<-ggplot(data=clusterMeans1, aes(x=Sample, y=X2,group=1)) +
  geom_line()+  geom_point()+ggtitle(paste("Cluster X2 Profile ",clustersizes[2]," genes"))+  scale_x_discrete(limits=orderN)+
  theme(axis.title.x = element_blank(),axis.title.y = element_blank())
pX3<-ggplot(data=clusterMeans1, aes(x=Sample, y=X3,group=1)) +
  geom_line()+  geom_point()+ggtitle(paste("Cluster X3 Profile ",clustersizes[3]," genes"))+  scale_x_discrete(limits=orderN)+
  theme(axis.title.x = element_blank(),axis.title.y = element_blank())
pX4<-ggplot(data=clusterMeans1, aes(x=Sample, y=X4,group=1)) +
  geom_line()+  geom_point()+ggtitle(paste("Cluster X4 Profile ",clustersizes[4]," genes"))+  scale_x_discrete(limits=orderN)+
  theme(axis.title.x = element_blank(),axis.title.y = element_blank())
pX5<-ggplot(data=clusterMeans1, aes(x=Sample, y=X5,group=1)) +
  geom_line()+  geom_point()+ggtitle(paste("Cluster X5 Profile ",clustersizes[5]," genes"))+  scale_x_discrete(limits=orderN)+
  theme(axis.title.x = element_blank(),axis.title.y = element_blank())
pX6<-ggplot(data=clusterMeans1, aes(x=Sample, y=X6,group=1)) +
  geom_line()+  geom_point()+ggtitle(paste("Cluster X6 Profile ",clustersizes[6]," genes"))+  scale_x_discrete(limits=orderN)+
  theme(axis.title.x = element_blank(),axis.title.y = element_blank())
#plot
multiplot(pX1, pX2, pX3, pX4,pX5, pX6, cols=2)  
 
 
   
 
 
 
 
 
 K-means clustering of means (other treatment means added for inspection) 
 
 
 
  hmap_k <- Heatmap(dataHMm, split=split, cluster_row_slices = FALSE,
                  cluster_columns = FALSE,
                  show_row_names = FALSE,
                  name = "Expression",
                  col = col_fun,
                  width = unit(25, "mm"),
                  column_title = "Var14noTNF", 
                  column_title_gp = gpar(fontsize = 10, fontface = "bold"))
                  
                  
baseMeansHmTemp <-countsTable[,c(48:50)]
colnames(baseMeansHmTemp)<-c("Var37TNF_0h","Var37TNF_6h","Var37TNF_20h")
dataHMmR1_37Y<-baseMeansHmTemp[ topDEgenes, ]
dataHMmR1_37Y <- log2(dataHMmR1_37Y+1)
dataHMmR1_37Y<- t(as.matrix(dataHMmR1_37Y))
dataHMmR1_37Y <- t(scale(dataHMmR1_37Y))
baseMeansHmTemp <-countsTable[,c(60:63)]
colnames(baseMeansHmTemp)<-c("Var14_0h","Var14_2h","Var14_6h","Var14_20h")
dataHMmR2_14<-baseMeansHmTemp[ topDEgenes, ]
dataHMmR2_14 <- log2(dataHMmR2_14+1)
dataHMmR2_14<- t(as.matrix(dataHMmR2_14))
dataHMmR2_14 <- t(scale(dataHMmR2_14))
baseMeansHmTemp <-countsTable[,c(79:82)]
colnames(baseMeansHmTemp)<-c("RBC_0h","RBC_2h","RBC_6h","RBC_20h")
dataHMmR2_R<-baseMeansHmTemp[ topDEgenes, ]
dataHMmR2_R <- log2(dataHMmR2_R+1)
dataHMmR2_R<- t(as.matrix(dataHMmR2_R))
dataHMmR2_R <- t(scale(dataHMmR2_R))
baseMeansHmTemp <-countsTable[,c(110:113)]
colnames(baseMeansHmTemp)<-c("Var14TNF_0h","Var14TNF_2h","Var14TNF_6h","Var14TNF_20h")
dataHMmR4_14T<-baseMeansHmTemp[ topDEgenes, ]
dataHMmR4_14T <- log2(dataHMmR4_14T+1)
dataHMmR4_14T<- t(as.matrix(dataHMmR4_14T))
dataHMmR4_14T <- t(scale(dataHMmR4_14T))
baseMeansHmTemp <-countsTable[,c(129:132)]
colnames(baseMeansHmTemp)<-c("RBC_TNF_0h","RBC_TNF_2h","RBC_TNF_6h","RBC_TNF_20h")
dataHMmR4_RT<-baseMeansHmTemp[ topDEgenes, ]
dataHMmR4_RT <- log2(dataHMmR4_RT+1)
dataHMmR4_RT<- t(as.matrix(dataHMmR4_RT))
dataHMmR4_RT <- t(scale(dataHMmR4_RT))
hmap_37T <- Heatmap(dataHMmR1_37Y, split=split, 
                  name = "Expression37T",  
                  column_title = "VAR37TNF", 
                  cluster_columns = FALSE,  show_row_names = FALSE,col = col_fun,width = unit(20, "mm"),
                  column_title_gp = gpar(fontsize = 10, fontface = "bold"))
hmap_k14 <- Heatmap(dataHMmR2_14, split=split, 
                  name = "Expression14",  
                  column_title = "VAR14noTNF", 
                  cluster_columns = FALSE,  show_row_names = FALSE,col = col_fun,width = unit(25, "mm"),
                  column_title_gp = gpar(fontsize = 10, fontface = "bold"))
hmap_R <- Heatmap(dataHMmR2_R, split=split, 
                  name = "ExpressionR",  
                  column_title = "RBCnoTNF", 
                  cluster_columns = FALSE,  show_row_names = FALSE,col = col_fun,width = unit(25, "mm"),
                  column_title_gp = gpar(fontsize = 10, fontface = "bold"))
hmap_k14T <- Heatmap(dataHMmR4_14T, split=split, 
                  name = "Expression14T",  
                  column_title = "VAR14TNF", 
                  cluster_columns = FALSE,  show_row_names = FALSE,col = col_fun,width = unit(25, "mm"),
                  column_title_gp = gpar(fontsize = 10, fontface = "bold"))
hmap_RT <- Heatmap(dataHMmR4_RT, split=split, 
                  name = "ExpressionRT",  
                  column_title = "RBCTNF", 
                  cluster_columns = FALSE,  show_row_names = FALSE,col = col_fun,width = unit(25, "mm"),
                  column_title_gp = gpar(fontsize = 10, fontface = "bold"))
hmap_k+ha+hmap_R+hmap_RT+hmap_k14T+hmap_37T  
 
 
   
 
 
 
 
 
 
  topDEgenes <- which(tempA$Include=="in")####find indexes
tempAkm<-tempA[ topDEgenes, ]
SymbolsKm<-dplyr::pull(tempAkm, Gene_Symbol)
#### export the gene expression data for the clusters
write.table(clusterMeans,paste0("ClusterMeansKm_",groupsName,".txt"),  sep = "\t")
ClusteredGenes<-data.frame(kclust4$cluster,SymbolsKm,dataHMm)
write.table(ClusteredGenes,paste0("ScaledDataInClustersKm_",groupsName,".txt"),  sep = "\t")
#head(ClusteredGenes)  
 
 
 
 
 
 
  bottomDEgenes<-which(tempA$Include=="out")####find indexes 
bottomG<-tempA[ bottomDEgenes, ]
bottomG<-dplyr::pull(bottomG, Gene_Symbol)
write.table(bottomG,paste0("ipaBottomKmeans_",groupsName,".txt"),  sep = "\t")
                         
topDEgenes <- which(tempA$Include=="in")####find indexes 
tempAkm<-tempA[ topDEgenes, ]
SymbolsKm<-dplyr::pull(tempAkm, Gene_Symbol)
ipaKmeans<-ClusteredGenes
#countsTable <-countsTable[,c(1:15)]####if samples need removing
ipaKmeans<-ipaKmeans[,c(1:2)]
ipaKmeans$name2<-rownames(ipaKmeans)
#ipaKmeans%>% rownames_to_column(var = "rowname")
#ipaKmeans
#rowid_to_column(ipaKmeans)
ipaKmeans = mutate(ipaKmeans, x1= ifelse(ipaKmeans$kclust4.cluster==1, "1", "0"))
ipaKmeans = mutate(ipaKmeans, x2= ifelse(ipaKmeans$kclust4.cluster==2, "1", "0"))
ipaKmeans = mutate(ipaKmeans, x3= ifelse(ipaKmeans$kclust4.cluster==3, "1", "0"))
ipaKmeans = mutate(ipaKmeans, x4= ifelse(ipaKmeans$kclust4.cluster==4, "1", "0"))
ipaKmeans = mutate(ipaKmeans, x5= ifelse(ipaKmeans$kclust4.cluster==5, "1", "0"))
ipaKmeans = mutate(ipaKmeans, x6= ifelse(ipaKmeans$kclust4.cluster==6, "1", "0"))
#ipaKmeans
write.table(ipaKmeans,paste0("ipaKmeans_",groupsName,".txt"),  sep = "\t")
#head(ipaKmeans)  
 
 
 
 
 
 
  ClusteredGenes2<-ClusteredGenes[c(1)]
#ClusteredGenes2
listAll<-list()
for(i in 1:6) {
  clusterName<-paste0("x",i)
  #clusterName<-row.names(subset(ClusteredGenes,ClusteredGenes==i))
  clusterName<-(subset(ClusteredGenes$SymbolsKm,ClusteredGenes==i))
  listAll[[i]]<-clusterName
}
#need to name the vectors in the list, example here is for 8 clusters
names(listAll)<-c("X1", "X2", "X3", "X4","X5", "X6")
#if you want to rearrange the order
#listAll<-listAll[c("x3", "x7", "x8", "x2", "x6", "x5", "x4", "x1")]
#lapply(listAll, head)  
 
 
 
 
 
 4. Annotation of K-means clusters 
 
 CC cellular compartment 
 BP biological process 
 MF molecular function 
 
 The simplify function has been used to cut down on GO redundancy 
 
 
 
  #str(AllGeneNames)  
 
 
 
 
 
 
  ####CC
cgoCC <- compareCluster(geneCluster = listAll, 
                      universe = AllGeneNames,
                      fun = "enrichGO",
                      OrgDb=org.Hs.eg.db, 
                      ####OrgDb=org.Mm.eg.db,
                      keyType="SYMBOL",
                      ont = "CC", 
                      pvalueCutoff=0.05,
                      qvalueCutoff = 0.10)
cgoCC2 <- simplify(cgoCC, cutoff=0.7, by="p.adjust", select_fun=min)
####write as spreadsheet
write.csv(as.data.frame(cgoCC2),paste0("GO_CC_",groupsName,".csv"))
dotplot(cgoCC2,showCategory = 30,
        title = paste0("GO Cellular Compartment ",groupsName))+
  theme(axis.text.x = element_text(angle = 90, vjust = 0.5, hjust=1))  
 
 
   
 
 
 
 Plots and GO data were written to files 
 
 
 
  png(paste0("GO_CC_",groupsName,".png"), width = 1224, height = 824)
dotplot(cgoCC2,showCategory = 30,
        title = paste0("GO Cellular Compartment ",groupsName))+
  theme(axis.text.x = element_text(angle = 90, vjust = 0.5, hjust=1))
dev.off()  
 
 
  null device 
          1   
 
 
 
 GO BP 
 
 
 
  ####CC
cgoBP <- compareCluster(geneCluster = listAll, 
                      universe = AllGeneNames,
                      fun = "enrichGO",
                      OrgDb=org.Hs.eg.db,
                      keyType="SYMBOL",
                      ont = "BP", 
                      pvalueCutoff=0.05,
                      qvalueCutoff = 0.10)
cgoBP2 <- simplify(cgoBP, cutoff=0.7, by="p.adjust", select_fun=min)
####write as spreadsheet
write.csv(as.data.frame(cgoBP2),paste0("GO_BP_",groupsName,".csv"))
dotplot(cgoBP2,showCategory = 30,
        title = paste0("GO Biological Process ",groupsName))+
  theme(axis.text.x = element_text(angle = 90, vjust = 0.5, hjust=1))  
 
 
   
 
 
 
 
 
 
  png(paste0("GO_BP_",groupsName,".png"), width = 1024, height = 1224)
dotplot(cgoBP2,showCategory = 30,
        title = paste0("GO Biological Process ",groupsName))+
  theme(axis.text.x = element_text(angle = 90, vjust = 0.5, hjust=1))
dev.off()  
 
 
  null device 
          1   
 
 
 
 GO MF 
 
 
 
  ####MF
cgoMF <- compareCluster(geneCluster = listAll, 
                      universe = AllGeneNames,
                      fun = "enrichGO",
                      OrgDb=org.Hs.eg.db, 
                      keyType="SYMBOL",
                      ont = "MF", 
                      pvalueCutoff=0.05,
                      qvalueCutoff = 0.10)
cgoMF2 <- simplify(cgoMF, cutoff=0.7, by="p.adjust", select_fun=min)
####write as spreadsheet
write.csv(as.data.frame(cgoMF2),paste0("GO_MF_",groupsName,".csv"))
dotplot(cgoMF2,showCategory = 30,
        title = paste0("GO Molecular Function  ",groupsName))+
  theme(axis.text.x = element_text(angle = 90, vjust = 0.5, hjust=1))  
 
 
   
 
 
 
 
 
 
  png(paste0("GO_MF_",groupsName,".png"), width = 1424, height = 824)
dotplot(cgoMF2,showCategory = 30,
        title = paste0("GO Molecular Function  ",groupsName))+
  theme(axis.text.x = element_text(angle = 90, vjust = 0.5, hjust=1))
dev.off()  
 
 
  null device 
          1   
 
 
 
 
 
 
 R2 RBC no TNF k-means q0.05 
 
 1. Genelist Selection 
 
 
 
  groupsName<-"R2_RBC_kmeans_q0.05"  
 
 
 
 
 
 
  countsTable<-read.delim("RNAseq2019July_5.txt", header = TRUE, sep = "\t",check.names=FALSE,row.names=1)
head(countsTable)  
 
 
 
 
 
 
  AllGeneNames<-countsTable$Gene_Symbol
#head(AllGeneNames)  
 
 
 
 
 
 
  tempA<-countsTable  
 
 
 
 
 
 
  topDEgenes <- which(tempA$padj_R2RBCnoTNF_Hours_2h_vs_0h<0.05&!is.na(tempA$padj_R2RBCnoTNF_Hours_2h_vs_0h))####find indexes 
listA<-tempA[ topDEgenes, ]$Gene_Symbol
topDEgenes <- which(tempA$padj_R2RBCnoTNF_Hours_6h_vs_0h<0.05&!is.na(tempA$padj_R2RBCnoTNF_Hours_6h_vs_0h))####find indexes 
listB<-tempA[ topDEgenes, ]$Gene_Symbol
topDEgenes <- which(tempA$padj_R2RBCnoTNF_Hours_20h_vs_0h<0.05&!is.na(tempA$padj_R2RBCnoTNF_Hours_20h_vs_0h))####find indexes 
listC<-tempA[ topDEgenes, ]$Gene_Symbol
topDEgenes <- which(tempA$padj_R2RBCnoTNF_Hours_6h_vs_2h<0.05&!is.na(tempA$padj_R2RBCnoTNF_Hours_6h_vs_2h))####find indexes 
listD<-tempA[ topDEgenes, ]$Gene_Symbol
topDEgenes <- which(tempA$padj_R2RBCnoTNF_Hours_20h_vs_6h<0.05&!is.na(tempA$padj_R2RBCnoTNF_Hours_20h_vs_6h))####find indexes 
listE<-tempA[ topDEgenes, ]$Gene_Symbol
vennq<-venn.diagram(x = list(listA,listB,listC,listD,listE) ,
            category.names = c("RBCnoTNF_2h_vs_0h","RBCnoTNF_6h_vs_0h","RBCnoTNF_20h_vs_0h","RBCnoTNF_6h_vs_2h","RBCnoTNF_20h_vs_6h"),
            main="padj<0.05",
            filename = NULL,  scaled = FALSE, fill = colorsV5, cat.col = colorsV5, cat.cex = 1, cat.dist=0.3,  margin = 0.3)
topDEgenes <- which(tempA$pvalue_R2RBCnoTNF_Hours_2h_vs_0h<0.05&abs(tempA$log2FoldChange_R2RBCnoTNF_Hours_2h_vs_0h)>1&!is.na(tempA$pvalue_R2RBCnoTNF_Hours_2h_vs_0h))####find indexes 
listA<-tempA[ topDEgenes, ]$Gene_Symbol
topDEgenes <- which(tempA$pvalue_R2RBCnoTNF_Hours_6h_vs_0h<0.05&abs(tempA$log2FoldChange_R2RBCnoTNF_Hours_6h_vs_0h)>1&!is.na(tempA$pvalue_R2RBCnoTNF_Hours_6h_vs_0h))####find indexes 
listB<-tempA[ topDEgenes, ]$Gene_Symbol
topDEgenes <- which(tempA$pvalue_R2RBCnoTNF_Hours_20h_vs_0h<0.05&abs(tempA$log2FoldChange_R2RBCnoTNF_Hours_20h_vs_0h)>1&!is.na(tempA$pvalue_R2RBCnoTNF_Hours_20h_vs_0h))####find indexes 
listC<-tempA[ topDEgenes, ]$Gene_Symbol
topDEgenes <- which(tempA$pvalue_R2RBCnoTNF_Hours_6h_vs_2h<0.05&abs(tempA$log2FoldChange_R2RBCnoTNF_Hours_6h_vs_2h)>1&!is.na(tempA$pvalue_R2RBCnoTNF_Hours_6h_vs_2h))####find indexes 
listD<-tempA[ topDEgenes, ]$Gene_Symbol
topDEgenes <- which(tempA$pvalue_R2RBCnoTNF_Hours_20h_vs_6h<0.05&abs(tempA$log2FoldChange_R2RBCnoTNF_Hours_20h_vs_6h)>1&!is.na(tempA$pvalue_R2RBCnoTNF_Hours_20h_vs_6h))####find indexes 
listE<-tempA[ topDEgenes, ]$Gene_Symbol
vennp<-venn.diagram(x = list(listA,listB,listC,listD,listE) ,
            category.names = c("RBCnoTNF_2h_vs_0h","RBCnoTNF_6h_vs_0h","RBCnoTNF_20h_vs_0h","RBCnoTNF_6h_vs_2h","RBCnoTNF_20h_vs_6h"),
            main="pvalue<0.05&fold change>2",
            filename = NULL,  scaled = FALSE, fill = colorsV5, cat.col = colorsV5, cat.cex = 1, cat.dist=0.3,  margin = 0.3)  
 
 
 
 
 
 
  topDEgenes <- which((tempA$padj_R2RBCnoTNF_Hours_2h_vs_0h<0.05&!is.na(tempA$padj_R2RBCnoTNF_Hours_2h_vs_0h))| 
(tempA$padj_R2RBCnoTNF_Hours_6h_vs_0h<0.05&!is.na(tempA$padj_R2RBCnoTNF_Hours_6h_vs_0h))|
(tempA$padj_R2RBCnoTNF_Hours_20h_vs_0h<0.05&!is.na(tempA$padj_R2RBCnoTNF_Hours_20h_vs_0h))| 
(tempA$padj_R2RBCnoTNF_Hours_6h_vs_2h<0.05&!is.na(tempA$padj_R2RBCnoTNF_Hours_6h_vs_2h))|
(tempA$padj_R2RBCnoTNF_Hours_20h_vs_6h<0.05&!is.na(tempA$padj_R2RBCnoTNF_Hours_20h_vs_6h)) 
)
listA<-tempA[ topDEgenes, ]$Gene_Symbol
topDEgenes <- which((tempA$pvalue_R2RBCnoTNF_Hours_2h_vs_0h<0.05&abs(tempA$log2FoldChange_R2RBCnoTNF_Hours_2h_vs_0h)>1&!is.na(tempA$pvalue_R2RBCnoTNF_Hours_2h_vs_0h))| 
(tempA$pvalue_R2RBCnoTNF_Hours_6h_vs_0h<0.05&abs(tempA$log2FoldChange_R2RBCnoTNF_Hours_6h_vs_0h)>1&!is.na(tempA$pvalue_R2RBCnoTNF_Hours_6h_vs_0h))| 
(tempA$pvalue_R2RBCnoTNF_Hours_20h_vs_0h<0.05&abs(tempA$log2FoldChange_R2RBCnoTNF_Hours_20h_vs_0h)>1&!is.na(tempA$pvalue_R2RBCnoTNF_Hours_20h_vs_0h))| 
(tempA$pvalue_R2RBCnoTNF_Hours_6h_vs_2h<0.05&abs(tempA$log2FoldChange_R2RBCnoTNF_Hours_6h_vs_2h)>1&!is.na(tempA$pvalue_R2RBCnoTNF_Hours_6h_vs_2h))| 
(tempA$pvalue_R2RBCnoTNF_Hours_20h_vs_6h<0.05&abs(tempA$log2FoldChange_R2RBCnoTNF_Hours_20h_vs_6h)>1&!is.na(tempA$pvalue_R2RBCnoTNF_Hours_20h_vs_6h))
 )####find indexes 
listC<-tempA[ topDEgenes, ]$Gene_Symbol
vennpq<-venn.diagram(x = list(listA,listC) ,
            category.names = c("padj<0.05","p<0.05&fc>2"),
            main="padj compared to pvalue",
            filename = NULL,  scaled = FALSE, fill = colorsV2, cat.col = colorsV2, cat.cex = 1, cat.dist=0.1,  margin = 0.15)  
 
 
 
 
 
 
  grid.arrange(gTree(children=vennq), gTree(children=vennpq) , ncol=2,top="R2 RBC no TNF")  
 
 
   
 
 
 
 
 
 
  tempA<-countsTable  
 
 
 
 
 
 
  #tempA<-resAll[-c(10:30) ]
tempA<-countsTable
#rownames(tempA)
rownames(tempA) <- NULL
tempA = mutate(tempA, Include=
                   ifelse(tempA$padj_R2RBCnoTNF_Hours_2h_vs_0h<0.05&!is.na(tempA$padj_R2RBCnoTNF_Hours_2h_vs_0h), "in",
                          ifelse(tempA$padj_R2RBCnoTNF_Hours_6h_vs_0h<0.05&!is.na(tempA$padj_R2RBCnoTNF_Hours_6h_vs_0h), "in",
                                 ifelse(tempA$padj_R2RBCnoTNF_Hours_20h_vs_0h<0.05&!is.na(tempA$padj_R2RBCnoTNF_Hours_20h_vs_0h), "in",
                                        ifelse(tempA$padj_R2RBCnoTNF_Hours_6h_vs_2h<0.05&!is.na(tempA$padj_R2RBCnoTNF_Hours_6h_vs_2h), "in",
                                               ifelse(tempA$padj_R2RBCnoTNF_Hours_20h_vs_6h<0.05&!is.na(tempA$padj_R2RBCnoTNF_Hours_20h_vs_6h), "in",
                                                                       "out"))))))
tempA  
 
 
 
 
 
 
 
  ####library(dplyr)
tempA %>%
     group_by(Include) %>% 
     tally()  
 
 
 
 
 
 
 
 
 
 
 
  topDEgenes <- which(tempA$Include=="in")####find indexes   
 
 
 
 
 
 
  head(baseMeansHm)  
 
 
 
 
 
 
 
 
 
 
 NB Please check columns used and renamed for plots 
 
 
 
  baseMeansHm <-countsTable[,c(79:82)]
head(baseMeansHm)  
 
 
 
 
 
 
 
  colnames(baseMeansHm)<-c("RBC_0h","RBC_2h","RBC_6h","RBC_20h")
head(baseMeansHm)  
 
 
 
 
 
 
 
 
 
  dataHi <-countsTable[,c(10:17)]
head(dataHi)  
 
 
 
 
 
 
 
 
 
 
 
  dataHi <-countsTable[,c(10:17)]
colnames(dataHi)<-c("RBC_0h_2","RBC_0h_3","RBC_2h_2","RBC_2h_3","RBC_6h_2","RBC_6h_3","RBC_20h_2","RBC_20h_3")
head(dataHi)  
 
 
 
 
 
 
 
  dataHi<-dataHi[ topDEgenes, ]
dataHi <- log2(dataHi+1)
dataHi<- t(as.matrix(dataHi))
dataHi <- t(scale(dataHi))
####str(dataHi)  
 
 
 
 
 
 
  topDEgenes <- which(tempA$Include=="in")####find indexes   
 
 
 
 
 
 2. Hierachical clustering of means (individual samples added for inspection) 
 
 
 
  hmap_hier_factors1 <- Heatmap(
  dataHi,  name = "ExpressionI",
  column_title = paste0("Individual Samples"), 
  column_title_gp = gpar(fontsize = 16, fontface = "bold"),
  width = unit(300, "mm"),
  col = col_fun,
  cluster_rows = FALSE,
  cluster_columns = FALSE,
  show_row_names = FALSE)
  #top_annotation=colAnn  )
####means
dataHMm<-baseMeansHm[ topDEgenes, ]
dataHMm <- log2(dataHMm+1)
dataHMm<- t(as.matrix(dataHMm))
dataHMm <- t(scale(dataHMm))
#colAnnm <- HeatmapAnnotation(df=annm, which="col", col=coloursm, annotation_width=unit(c(2, 4), "cm"), gap=unit(1, "mm"))
hmap_hier_factors4 <- Heatmap(
  dataHMm,  name = "Expression",
  row_labels = paste0(rownames(dataHMm)," ",(tempA[ topDEgenes, ])$Gene_Symbol),
  column_title = paste0("Means"), 
  col = col_fun,
  column_title_gp = gpar(fontsize = 16, fontface = "bold"),
  width = unit(50, "mm"),
  cluster_columns = FALSE,
  show_row_names = FALSE)
  #top_annotation=colAnnm  )
hmap_hier_factors4+hmap_hier_factors1  
 
 
   
 
 
 
 
 
 
  par(mfrow=c(1,2))
#### Silhouette method
fviz_nbclust(dataHMm, kmeans, method = "silhouette",k.max = 16)+
  labs(subtitle = "Silhouette method")  
 
 
   
 
 
  #### Elbow method
fviz_nbclust(dataHMm, kmeans, method = "wss",k.max = 16) +
  labs(subtitle = "Elbow method")  
 
 
   
 
 
 
 
 
 
  ####gap stat slow!!!
####set.seed(123)
####fviz_nbclust(dataHMm, kmeans, nstart = 25,  method = "gap_stat", nboot = 100,k.max = 16)+
####  labs(subtitle = "Gap statistic method")  
 
 
 
 
 
 
  #kclust5 <- kmeans(dataHMm, 6)
#silhouette plot
distK<-daisy(dataHMm)
plot(silhouette(kclust5$cluster, distK), col=1:6, border=NA)  
 
 
   
 
 
 
 
 
 3. K-means clustering of means 
 
 
 
  #split <- paste0("Cluster\n", kclust5$cluster)
split <- factor(paste0("Cluster\n", kclust5$cluster), levels=c("Cluster\n3","Cluster\n2","Cluster\n4","Cluster\n5","Cluster\n6","Cluster\n1"))
hmap_k <- Heatmap(dataHMm, split=split, cluster_row_slices = FALSE,
                  cluster_columns = FALSE,
                  show_row_names = FALSE,
                  name = "Expression",
                  col = col_fun,
                  width = unit(20, "mm"),
                  column_title = "means", 
                  column_title_gp = gpar(fontsize = 16, fontface = "bold")
                  
                        )#top_annotation=colAnn)
hmap_hier_factors1 <- Heatmap(
  dataHi,  name = "ExpressionI",
  col = col_fun,
  column_title = paste0("individual samples"), 
  column_title_gp = gpar(fontsize = 16, fontface = "bold"),
  width = unit(60, "mm"),
  cluster_rows = FALSE,
  cluster_columns = FALSE,
  show_row_names = FALSE)
hmap_k  
 
 
   
 
 
 
 
 
 K-means clustering of means (with cluster annotation and individual samples added for inspection) 
 
 
 
  Response_Time<-data.frame(kclust5$cluster)
Response_Time = mutate(Response_Time, Response=
                   ifelse(Response_Time$kclust5.cluster==3, "late", 
                          ifelse(Response_Time$kclust5.cluster==4, "transient",
                                 ifelse(Response_Time$kclust5.cluster==1, "late",
                                        ifelse(Response_Time$kclust5.cluster==5, "transient",
                                               ifelse(Response_Time$kclust5.cluster==2, "transient",
                                                      ifelse(Response_Time$kclust5.cluster==6, "transient",
                                                                       "out")))))))
Response_Time<-Response_Time[c(2)]
rownames(Response_Time) <- NULL
ha = HeatmapAnnotation(df = Response_Time, which = "row", width = unit(1, "cm"),col = list(Response = c("early" =  "green3", "late" = "brown", "transient" = "violet")))
hmap_k+ha+hmap_hier_factors1  
 
 
   
 
 
 
 Mean profiles of clusters 
 
 
 
  clustercount<-data.frame(kclust5$cluster)
clustersizes<-table(clustercount$kclust5.cluster)
clusterMeans<-data.frame(kclust5$centers)
clusterMeans1<-data.frame(t(clusterMeans))
clusterMeans1 <- cbind(rownames(clusterMeans1), clusterMeans1)
orderN<-c("RBC_0h","RBC_2h","RBC_6h","RBC_20h")#### manual
rownames(clusterMeans1) <- NULL
names(clusterMeans1)[names(clusterMeans1)=="rownames(clusterMeans1)"] <- "Sample"
####clusterMeans1
pX1<-ggplot(data=clusterMeans1, aes(x=Sample, y=X1,group=1)) +
  geom_line()+  geom_point()+ggtitle(paste("Cluster X1 Profile ",clustersizes[1]," genes"))+  scale_x_discrete(limits=orderN)+
  theme(axis.title.x = element_blank(),axis.title.y = element_blank())
pX2<-ggplot(data=clusterMeans1, aes(x=Sample, y=X2,group=1)) +
  geom_line()+  geom_point()+ggtitle(paste("Cluster X2 Profile ",clustersizes[2]," genes"))+  scale_x_discrete(limits=orderN)+
  theme(axis.title.x = element_blank(),axis.title.y = element_blank())
pX3<-ggplot(data=clusterMeans1, aes(x=Sample, y=X3,group=1)) +
  geom_line()+  geom_point()+ggtitle(paste("Cluster X3 Profile ",clustersizes[3]," genes"))+  scale_x_discrete(limits=orderN)+
  theme(axis.title.x = element_blank(),axis.title.y = element_blank())
pX4<-ggplot(data=clusterMeans1, aes(x=Sample, y=X4,group=1)) +
  geom_line()+  geom_point()+ggtitle(paste("Cluster X4 Profile ",clustersizes[4]," genes"))+  scale_x_discrete(limits=orderN)+
  theme(axis.title.x = element_blank(),axis.title.y = element_blank())
pX5<-ggplot(data=clusterMeans1, aes(x=Sample, y=X5,group=1)) +
  geom_line()+  geom_point()+ggtitle(paste("Cluster X5 Profile ",clustersizes[5]," genes"))+  scale_x_discrete(limits=orderN)+
  theme(axis.title.x = element_blank(),axis.title.y = element_blank())
pX6<-ggplot(data=clusterMeans1, aes(x=Sample, y=X6,group=1)) +
  geom_line()+  geom_point()+ggtitle(paste("Cluster X6 Profile ",clustersizes[6]," genes"))+  scale_x_discrete(limits=orderN)+
  theme(axis.title.x = element_blank(),axis.title.y = element_blank())
#plot
multiplot(pX1, pX2, pX3, pX4,pX5, pX6, cols=2)  
 
 
   
 
 
 
 
 
 K-means clustering of means (other treatment means added for inspection) 
 
 
 
  hmap_k <- Heatmap(dataHMm, split=split, cluster_row_slices = FALSE,
                  cluster_columns = FALSE,
                  show_row_names = FALSE,
                  name = "Expression",
                  col = col_fun,
                  width = unit(25, "mm"),
                  column_title = "RBCnoTNF", 
                  column_title_gp = gpar(fontsize = 10, fontface = "bold"))
                  
                  
baseMeansHmTemp <-countsTable[,c(48:50)]
colnames(baseMeansHmTemp)<-c("Var37TNF_0h","Var37TNF_6h","Var37TNF_20h")
dataHMmR1_37Y<-baseMeansHmTemp[ topDEgenes, ]
dataHMmR1_37Y <- log2(dataHMmR1_37Y+1)
dataHMmR1_37Y<- t(as.matrix(dataHMmR1_37Y))
dataHMmR1_37Y <- t(scale(dataHMmR1_37Y))
baseMeansHmTemp <-countsTable[,c(60:63)]
colnames(baseMeansHmTemp)<-c("Var14_0h","Var14_2h","Var14_6h","Var14_20h")
dataHMmR2_14<-baseMeansHmTemp[ topDEgenes, ]
dataHMmR2_14 <- log2(dataHMmR2_14+1)
dataHMmR2_14<- t(as.matrix(dataHMmR2_14))
dataHMmR2_14 <- t(scale(dataHMmR2_14))
baseMeansHmTemp <-countsTable[,c(79:82)]
colnames(baseMeansHmTemp)<-c("RBC_0h","RBC_2h","RBC_6h","RBC_20h")
dataHMmR2_R<-baseMeansHmTemp[ topDEgenes, ]
dataHMmR2_R <- log2(dataHMmR2_R+1)
dataHMmR2_R<- t(as.matrix(dataHMmR2_R))
dataHMmR2_R <- t(scale(dataHMmR2_R))
baseMeansHmTemp <-countsTable[,c(110:113)]
colnames(baseMeansHmTemp)<-c("Var14TNF_0h","Var14TNF_2h","Var14TNF_6h","Var14TNF_20h")
dataHMmR4_14T<-baseMeansHmTemp[ topDEgenes, ]
dataHMmR4_14T <- log2(dataHMmR4_14T+1)
dataHMmR4_14T<- t(as.matrix(dataHMmR4_14T))
dataHMmR4_14T <- t(scale(dataHMmR4_14T))
baseMeansHmTemp <-countsTable[,c(129:132)]
colnames(baseMeansHmTemp)<-c("RBC_TNF_0h","RBC_TNF_2h","RBC_TNF_6h","RBC_TNF_20h")
dataHMmR4_RT<-baseMeansHmTemp[ topDEgenes, ]
dataHMmR4_RT <- log2(dataHMmR4_RT+1)
dataHMmR4_RT<- t(as.matrix(dataHMmR4_RT))
dataHMmR4_RT <- t(scale(dataHMmR4_RT))
hmap_37T <- Heatmap(dataHMmR1_37Y, split=split, 
                  name = "Expression37T",  
                  column_title = "VAR37TNF", 
                  cluster_columns = FALSE,  show_row_names = FALSE,col = col_fun,width = unit(20, "mm"),
                  column_title_gp = gpar(fontsize = 10, fontface = "bold"))
hmap_k14 <- Heatmap(dataHMmR2_14, split=split, 
                  name = "Expression14",  
                  column_title = "VAR14noTNF", 
                  cluster_columns = FALSE,  show_row_names = FALSE,col = col_fun,width = unit(25, "mm"),
                  column_title_gp = gpar(fontsize = 10, fontface = "bold"))
hmap_R <- Heatmap(dataHMmR2_R, split=split, 
                  name = "ExpressionR",  
                  column_title = "RBCnoTNF", 
                  cluster_columns = FALSE,  show_row_names = FALSE,col = col_fun,width = unit(25, "mm"),
                  column_title_gp = gpar(fontsize = 10, fontface = "bold"))
hmap_k14T <- Heatmap(dataHMmR4_14T, split=split, 
                  name = "Expression14T",  
                  column_title = "VAR14TNF", 
                  cluster_columns = FALSE,  show_row_names = FALSE,col = col_fun,width = unit(25, "mm"),
                  column_title_gp = gpar(fontsize = 10, fontface = "bold"))
hmap_RT <- Heatmap(dataHMmR4_RT, split=split, 
                  name = "ExpressionRT",  
                  column_title = "RBCTNF", 
                  cluster_columns = FALSE,  show_row_names = FALSE,col = col_fun,width = unit(25, "mm"),
                  column_title_gp = gpar(fontsize = 10, fontface = "bold"))
hmap_k+ha+hmap_k14+hmap_RT+hmap_k14T+hmap_37T  
 
 
   
 
 
 
 
 
 
  topDEgenes <- which(tempA$Include=="in")####find indexes
tempAkm<-tempA[ topDEgenes, ]
SymbolsKm<-dplyr::pull(tempAkm, Gene_Symbol)
#### export the gene expression data for the clusters
write.table(clusterMeans,paste0("ClusterMeansKm_",groupsName,".txt"),  sep = "\t")
ClusteredGenes<-data.frame(kclust5$cluster,SymbolsKm,dataHMm)
write.table(ClusteredGenes,paste0("ScaledDataInClustersKm_",groupsName,".txt"),  sep = "\t")
#head(ClusteredGenes)  
 
 
 
 
 
 
  bottomDEgenes<-which(tempA$Include=="out")####find indexes 
bottomG<-tempA[ bottomDEgenes, ]
bottomG<-dplyr::pull(bottomG, Gene_Symbol)
write.table(bottomG,paste0("ipaBottomKmeans_",groupsName,".txt"),  sep = "\t")
                         
topDEgenes <- which(tempA$Include=="in")####find indexes 
tempAkm<-tempA[ topDEgenes, ]
SymbolsKm<-dplyr::pull(tempAkm, Gene_Symbol)
ipaKmeans<-ClusteredGenes
#countsTable <-countsTable[,c(1:15)]####if samples need removing
ipaKmeans<-ipaKmeans[,c(1:2)]
ipaKmeans$name2<-rownames(ipaKmeans)
#ipaKmeans%>% rownames_to_column(var = "rowname")
#ipaKmeans
#rowid_to_column(ipaKmeans)
ipaKmeans = mutate(ipaKmeans, x1= ifelse(ipaKmeans$kclust5.cluster==1, "1", "0"))
ipaKmeans = mutate(ipaKmeans, x2= ifelse(ipaKmeans$kclust5.cluster==2, "1", "0"))
ipaKmeans = mutate(ipaKmeans, x3= ifelse(ipaKmeans$kclust5.cluster==3, "1", "0"))
ipaKmeans = mutate(ipaKmeans, x4= ifelse(ipaKmeans$kclust5.cluster==4, "1", "0"))
ipaKmeans = mutate(ipaKmeans, x5= ifelse(ipaKmeans$kclust5.cluster==5, "1", "0"))
ipaKmeans = mutate(ipaKmeans, x6= ifelse(ipaKmeans$kclust5.cluster==6, "1", "0"))
#ipaKmeans
write.table(ipaKmeans,paste0("ipaKmeans_",groupsName,".txt"),  sep = "\t")
#head(ipaKmeans)  
 
 
 
 
 
 
  ClusteredGenes2<-ClusteredGenes[c(1)]
#ClusteredGenes2
listAll<-list()
for(i in 1:6) {
  clusterName<-paste0("x",i)
  #clusterName<-row.names(subset(ClusteredGenes,ClusteredGenes==i))
  clusterName<-(subset(ClusteredGenes$SymbolsKm,ClusteredGenes==i))
  listAll[[i]]<-clusterName
}
#need to name the vectors in the list, example here is for 8 clusters
names(listAll)<-c("X1", "X2", "X3", "X4","X5", "X6")
#if you want to rearrange the order
#listAll<-listAll[c("x3", "x7", "x8", "x2", "x6", "x5", "x4", "x1")]
#lapply(listAll, head)  
 
 
 
 
 
 4. Annotation of K-means clusters 
 
 CC cellular compartment 
 BP biological process 
 MF molecular function 
 
 The simplify function has been used to cut down on GO redundancy 
 
 
 
  #str(AllGeneNames)  
 
 
 
 
 
 
  ####CC
cgoCC <- compareCluster(geneCluster = listAll, 
                      universe = AllGeneNames,
                      fun = "enrichGO",
                      OrgDb=org.Hs.eg.db, 
                      ####OrgDb=org.Mm.eg.db,
                      keyType="SYMBOL",
                      ont = "CC", 
                      pvalueCutoff=0.05,
                      qvalueCutoff = 0.10)
cgoCC2 <- simplify(cgoCC, cutoff=0.7, by="p.adjust", select_fun=min)
####write as spreadsheet
write.csv(as.data.frame(cgoCC2),paste0("GO_CC_",groupsName,".csv"))
dotplot(cgoCC2,showCategory = 30,
        title = paste0("GO Cellular Compartment ",groupsName))+
  theme(axis.text.x = element_text(angle = 90, vjust = 0.5, hjust=1))  
 
 
   
 
 
 
 
 
 
  png(paste0("GO_CC_",groupsName,".png"), width = 1224, height = 824)
dotplot(cgoCC2,showCategory = 30,
        title = paste0("GO Cellular Compartment ",groupsName))+
  theme(axis.text.x = element_text(angle = 90, vjust = 0.5, hjust=1))
dev.off()  
 
 
  null device 
          1   
 
 
 
 GO BP 
 
 
 
  ####CC
cgoBP <- compareCluster(geneCluster = listAll, 
                      universe = AllGeneNames,
                      fun = "enrichGO",
                      OrgDb=org.Hs.eg.db,
                      keyType="SYMBOL",
                      ont = "BP", 
                      pvalueCutoff=0.05,
                      qvalueCutoff = 0.10)
cgoBP2 <- simplify(cgoBP, cutoff=0.7, by="p.adjust", select_fun=min)
####write as spreadsheet
write.csv(as.data.frame(cgoBP2),paste0("GO_BP_",groupsName,".csv"))
dotplot(cgoBP2,showCategory = 30,
        title = paste0("GO Biological Process ",groupsName))+
  theme(axis.text.x = element_text(angle = 90, vjust = 0.5, hjust=1))  
 
 
   
 
 
 
 Plots and GO data were written to files 
 
 
 
  png(paste0("GO_BP_",groupsName,".png"), width = 1024, height = 1624)
dotplot(cgoBP2,showCategory = 30,
        title = paste0("GO Biological Process ",groupsName))+
  theme(axis.text.x = element_text(angle = 90, vjust = 0.5, hjust=1))
dev.off()  
 
 
  null device 
          1   
 
 
 
 GO MF 
 
 
 
  ####MF
cgoMF <- compareCluster(geneCluster = listAll, 
                      universe = AllGeneNames,
                      fun = "enrichGO",
                      OrgDb=org.Hs.eg.db, 
                      keyType="SYMBOL",
                      ont = "MF", 
                      pvalueCutoff=0.05,
                      qvalueCutoff = 0.10)
cgoMF2 <- simplify(cgoMF, cutoff=0.7, by="p.adjust", select_fun=min)
####write as spreadsheet
write.csv(as.data.frame(cgoMF2),paste0("GO_MF_",groupsName,".csv"))
dotplot(cgoMF2,showCategory = 30,
        title = paste0("GO Molecular Function  ",groupsName))+
  theme(axis.text.x = element_text(angle = 90, vjust = 0.5, hjust=1))  
 
 
   
 
 
 
 
 
 
  png(paste0("GO_MF_",groupsName,".png"), width = 1424, height = 1224)
dotplot(cgoMF2,showCategory = 30,
        title = paste0("GO Molecular Function  ",groupsName))+
  theme(axis.text.x = element_text(angle = 90, vjust = 0.5, hjust=1))
dev.off()  
 
 
  null device 
          1   
 
 
 
 
 
 
 R2 RBC no TNF k-means p0.05fc2 
 
 1. Genelist Selection 
 
 
 
  groupsName<-"R2_RBC_kmeans_p0.05fc2"  
 
 
 
 
 
 
  countsTable<-read.delim("RNAseq2019July_5.txt", header = TRUE, sep = "\t",check.names=FALSE,row.names=1)
head(countsTable)  
 
 
 
 
 
 
 
 
 
 
 
  AllGeneNames<-countsTable$Gene_Symbol
#head(AllGeneNames)  
 
 
 
 
 
 
  grid.arrange(gTree(children=vennp), gTree(children=vennpq) , ncol=2,top="R2 RBC no TNF")  
 
 
   
 
 
 
 
 
 
  #tempA<-resAll[-c(10:30) ]
tempA<-countsTable
#rownames(tempA)
rownames(tempA) <- NULL
tempA = mutate(tempA, Include=
                   ifelse(tempA$pvalue_R2RBCnoTNF_Hours_2h_vs_0h<0.05&abs(tempA$log2FoldChange_R2RBCnoTNF_Hours_2h_vs_0h)>1&!is.na(tempA$pvalue_R2RBCnoTNF_Hours_2h_vs_0h), "in",
                          ifelse(tempA$pvalue_R2RBCnoTNF_Hours_6h_vs_0h<0.05&abs(tempA$log2FoldChange_R2RBCnoTNF_Hours_6h_vs_0h)>1&!is.na(tempA$pvalue_R2RBCnoTNF_Hours_6h_vs_0h), "in",
                                 ifelse(tempA$pvalue_R2RBCnoTNF_Hours_20h_vs_0h<0.05&abs(tempA$log2FoldChange_R2RBCnoTNF_Hours_20h_vs_0h)>1&!is.na(tempA$pvalue_R2RBCnoTNF_Hours_20h_vs_0h), "in",
                                        ifelse(tempA$pvalue_R2RBCnoTNF_Hours_6h_vs_2h<0.05&abs(tempA$log2FoldChange_R2RBCnoTNF_Hours_6h_vs_2h)>1&!is.na(tempA$pvalue_R2RBCnoTNF_Hours_6h_vs_2h), "in",
                                               ifelse(tempA$pvalue_R2RBCnoTNF_Hours_20h_vs_6h<0.05&abs(tempA$log2FoldChange_R2RBCnoTNF_Hours_20h_vs_6h)>1&!is.na(tempA$pvalue_R2RBCnoTNF_Hours_20h_vs_6h), "in",
                                                                       "out"))))))
tempA  
 
 
 
 
 
 
 
  ####library(dplyr)
tempA %>%
     group_by(Include) %>% 
     tally()  
 
 
 
 
 
 
 
 
 
 
 
  topDEgenes <- which(tempA$Include=="in")####find indexes   
 
 
 
 
 
 NB Please check columns used and renamed for plots 
 
 
 
  baseMeansHm <-countsTable[,c(79:82)]
head(baseMeansHm)  
 
 
 
 
 
 
 
  colnames(baseMeansHm)<-c("RBC_0h","RBC_2h","RBC_6h","RBC_20h")
head(baseMeansHm)  
 
 
 
 
 
 
 
 
 
 
 
  dataHi <-countsTable[,c(10:17)]
head(dataHi)  
 
 
 
 
 
 
 
 
 
 
 
  dataHi <-countsTable[,c(10:17)]
colnames(dataHi)<-c("RBC_0h_2","RBC_0h_3","RBC_2h_2","RBC_2h_3","RBC_6h_2","RBC_6h_3","RBC_20h_2","RBC_20h_3")
head(dataHi)  
 
 
 
 
 
 
 
  dataHi<-dataHi[ topDEgenes, ]
dataHi <- log2(dataHi+1)
dataHi<- t(as.matrix(dataHi))
dataHi <- t(scale(dataHi))
####str(dataHi)  
 
 
 
 
 
 
  topDEgenes <- which(tempA$Include=="in")####find indexes   
 
 
 
 
 
 2. Hierachical clustering of means (individual samples added for inspection) 
 
 
 
  hmap_hier_factors1 <- Heatmap(
  dataHi,  name = "ExpressionI",
  column_title = paste0("Individual Samples"), 
  column_title_gp = gpar(fontsize = 16, fontface = "bold"),
  width = unit(300, "mm"),
  col = col_fun,
  cluster_rows = FALSE,
  cluster_columns = FALSE,
  show_row_names = FALSE)
  #top_annotation=colAnn  )
####means
dataHMm<-baseMeansHm[ topDEgenes, ]
dataHMm <- log2(dataHMm+1)
dataHMm<- t(as.matrix(dataHMm))
dataHMm <- t(scale(dataHMm))
#colAnnm <- HeatmapAnnotation(df=annm, which="col", col=coloursm, annotation_width=unit(c(2, 4), "cm"), gap=unit(1, "mm"))
hmap_hier_factors4 <- Heatmap(
  dataHMm,  name = "Expression",
  row_labels = paste0(rownames(dataHMm)," ",(tempA[ topDEgenes, ])$Gene_Symbol),
  column_title = paste0("Means"), 
  col = col_fun,
  column_title_gp = gpar(fontsize = 16, fontface = "bold"),
  width = unit(50, "mm"),
  cluster_columns = FALSE,
  show_row_names = FALSE)
  #top_annotation=colAnnm  )
hmap_hier_factors4+hmap_hier_factors1  
 
 
   
 
 
 
 
 
 
  par(mfrow=c(1,2))
#### Silhouette method
fviz_nbclust(dataHMm, kmeans, method = "silhouette",k.max = 16)+
  labs(subtitle = "Silhouette method")  
 
 
   
 
 
  #### Elbow method
fviz_nbclust(dataHMm, kmeans, method = "wss",k.max = 16) +
  labs(subtitle = "Elbow method")  
 
 
   
 
 
 
 
 
 
  ####gap stat slow!!!
####set.seed(123)
####fviz_nbclust(dataHMm, kmeans, nstart = 25,  method = "gap_stat", nboot = 100,k.max = 16)+
####  labs(subtitle = "Gap statistic method")  
 
 
 
 
 
 
  #kclust6 <- kmeans(dataHMm, 6)
#silhouette plot
distK<-daisy(dataHMm)
plot(silhouette(kclust6$cluster, distK), col=1:6, border=NA)  
 
 
   
 
 
 
 
 
 3. K-means clustering of means 
 
 
 
  #split <- paste0("Cluster\n", kclust6$cluster)
split <- factor(paste0("Cluster\n", kclust6$cluster), levels=c("Cluster\n5","Cluster\n1","Cluster\n2","Cluster\n3","Cluster\n4","Cluster\n6"))
hmap_k <- Heatmap(dataHMm, split=split, cluster_row_slices = FALSE,
                  cluster_columns = FALSE,
                  show_row_names = FALSE,
                  name = "Expression",
                  col = col_fun,
                  width = unit(20, "mm"),
                  column_title = "means", 
                  column_title_gp = gpar(fontsize = 16, fontface = "bold")
                  
                        )#top_annotation=colAnn)
hmap_hier_factors1 <- Heatmap(
  dataHi,  name = "ExpressionI",
  col = col_fun,
  column_title = paste0("individual samples"), 
  column_title_gp = gpar(fontsize = 16, fontface = "bold"),
  width = unit(60, "mm"),
  cluster_rows = FALSE,
  cluster_columns = FALSE,
  show_row_names = FALSE)
hmap_k  
 
 
   
 
 
 
 
 
 K-means clustering of means (with cluster annotation and individual samples added for inspection) 
 
 
 
  Response_Time<-data.frame(kclust6$cluster)
Response_Time = mutate(Response_Time, Response=
                   ifelse(Response_Time$kclust6.cluster==1, "transient", 
                          ifelse(Response_Time$kclust6.cluster==5, "late",
                                 ifelse(Response_Time$kclust6.cluster==6, "late",
                                        ifelse(Response_Time$kclust6.cluster==2, "transient",
                                               ifelse(Response_Time$kclust6.cluster==3, "transient",
                                                      ifelse(Response_Time$kclust6.cluster==4, "transient",
                                                                       "out")))))))
Response_Time<-Response_Time[c(2)]
rownames(Response_Time) <- NULL
ha = HeatmapAnnotation(df = Response_Time, which = "row", width = unit(1, "cm"),col = list(Response = c("early" =  "green3", "late" = "brown", "transient" = "violet")))
hmap_k+ha+hmap_hier_factors1  
 
 
   
 
 
 
 Mean profiles of clusters 
 
 
 
  clustercount<-data.frame(kclust6$cluster)
clustersizes<-table(clustercount$kclust6.cluster)
clusterMeans<-data.frame(kclust6$centers)
clusterMeans1<-data.frame(t(clusterMeans))
clusterMeans1 <- cbind(rownames(clusterMeans1), clusterMeans1)
orderN<-c("RBC_0h","RBC_2h","RBC_6h","RBC_20h")#### manual
rownames(clusterMeans1) <- NULL
names(clusterMeans1)[names(clusterMeans1)=="rownames(clusterMeans1)"] <- "Sample"
####clusterMeans1
pX1<-ggplot(data=clusterMeans1, aes(x=Sample, y=X1,group=1)) +
  geom_line()+  geom_point()+ggtitle(paste("Cluster X1 Profile ",clustersizes[1]," genes"))+  scale_x_discrete(limits=orderN)+
  theme(axis.title.x = element_blank(),axis.title.y = element_blank())
pX2<-ggplot(data=clusterMeans1, aes(x=Sample, y=X2,group=1)) +
  geom_line()+  geom_point()+ggtitle(paste("Cluster X2 Profile ",clustersizes[2]," genes"))+  scale_x_discrete(limits=orderN)+
  theme(axis.title.x = element_blank(),axis.title.y = element_blank())
pX3<-ggplot(data=clusterMeans1, aes(x=Sample, y=X3,group=1)) +
  geom_line()+  geom_point()+ggtitle(paste("Cluster X3 Profile ",clustersizes[3]," genes"))+  scale_x_discrete(limits=orderN)+
  theme(axis.title.x = element_blank(),axis.title.y = element_blank())
pX4<-ggplot(data=clusterMeans1, aes(x=Sample, y=X4,group=1)) +
  geom_line()+  geom_point()+ggtitle(paste("Cluster X4 Profile ",clustersizes[4]," genes"))+  scale_x_discrete(limits=orderN)+
  theme(axis.title.x = element_blank(),axis.title.y = element_blank())
pX5<-ggplot(data=clusterMeans1, aes(x=Sample, y=X5,group=1)) +
  geom_line()+  geom_point()+ggtitle(paste("Cluster X5 Profile ",clustersizes[5]," genes"))+  scale_x_discrete(limits=orderN)+
  theme(axis.title.x = element_blank(),axis.title.y = element_blank())
pX6<-ggplot(data=clusterMeans1, aes(x=Sample, y=X6,group=1)) +
  geom_line()+  geom_point()+ggtitle(paste("Cluster X6 Profile ",clustersizes[6]," genes"))+  scale_x_discrete(limits=orderN)+
  theme(axis.title.x = element_blank(),axis.title.y = element_blank())
#plot
multiplot(pX1, pX2, pX3, pX4,pX5, pX6, cols=2)  
 
 
   
 
 
 
 
 
 K-means clustering of means (other treatment means added for inspection) 
 
 
 
  hmap_k <- Heatmap(dataHMm, split=split, cluster_row_slices = FALSE,
                  cluster_columns = FALSE,
                  show_row_names = FALSE,
                  name = "Expression",
                  col = col_fun,
                  width = unit(25, "mm"),
                  column_title = "RBCnoTNF", 
                  column_title_gp = gpar(fontsize = 10, fontface = "bold"))
                  
                  
baseMeansHmTemp <-countsTable[,c(48:50)]
colnames(baseMeansHmTemp)<-c("Var37TNF_0h","Var37TNF_6h","Var37TNF_20h")
dataHMmR1_37Y<-baseMeansHmTemp[ topDEgenes, ]
dataHMmR1_37Y <- log2(dataHMmR1_37Y+1)
dataHMmR1_37Y<- t(as.matrix(dataHMmR1_37Y))
dataHMmR1_37Y <- t(scale(dataHMmR1_37Y))
baseMeansHmTemp <-countsTable[,c(60:63)]
colnames(baseMeansHmTemp)<-c("Var14_0h","Var14_2h","Var14_6h","Var14_20h")
dataHMmR2_14<-baseMeansHmTemp[ topDEgenes, ]
dataHMmR2_14 <- log2(dataHMmR2_14+1)
dataHMmR2_14<- t(as.matrix(dataHMmR2_14))
dataHMmR2_14 <- t(scale(dataHMmR2_14))
baseMeansHmTemp <-countsTable[,c(79:82)]
colnames(baseMeansHmTemp)<-c("RBC_0h","RBC_2h","RBC_6h","RBC_20h")
dataHMmR2_R<-baseMeansHmTemp[ topDEgenes, ]
dataHMmR2_R <- log2(dataHMmR2_R+1)
dataHMmR2_R<- t(as.matrix(dataHMmR2_R))
dataHMmR2_R <- t(scale(dataHMmR2_R))
baseMeansHmTemp <-countsTable[,c(110:113)]
colnames(baseMeansHmTemp)<-c("Var14TNF_0h","Var14TNF_2h","Var14TNF_6h","Var14TNF_20h")
dataHMmR4_14T<-baseMeansHmTemp[ topDEgenes, ]
dataHMmR4_14T <- log2(dataHMmR4_14T+1)
dataHMmR4_14T<- t(as.matrix(dataHMmR4_14T))
dataHMmR4_14T <- t(scale(dataHMmR4_14T))
baseMeansHmTemp <-countsTable[,c(129:132)]
colnames(baseMeansHmTemp)<-c("RBC_TNF_0h","RBC_TNF_2h","RBC_TNF_6h","RBC_TNF_20h")
dataHMmR4_RT<-baseMeansHmTemp[ topDEgenes, ]
dataHMmR4_RT <- log2(dataHMmR4_RT+1)
dataHMmR4_RT<- t(as.matrix(dataHMmR4_RT))
dataHMmR4_RT <- t(scale(dataHMmR4_RT))
hmap_37T <- Heatmap(dataHMmR1_37Y, split=split, 
                  name = "Expression37T",  
                  column_title = "VAR37TNF", 
                  cluster_columns = FALSE,  show_row_names = FALSE,col = col_fun,width = unit(20, "mm"),
                  column_title_gp = gpar(fontsize = 10, fontface = "bold"))
hmap_k14 <- Heatmap(dataHMmR2_14, split=split, 
                  name = "Expression14",  
                  column_title = "VAR14noTNF", 
                  cluster_columns = FALSE,  show_row_names = FALSE,col = col_fun,width = unit(25, "mm"),
                  column_title_gp = gpar(fontsize = 10, fontface = "bold"))
hmap_R <- Heatmap(dataHMmR2_R, split=split, 
                  name = "ExpressionR",  
                  column_title = "RBCnoTNF", 
                  cluster_columns = FALSE,  show_row_names = FALSE,col = col_fun,width = unit(25, "mm"),
                  column_title_gp = gpar(fontsize = 10, fontface = "bold"))
hmap_k14T <- Heatmap(dataHMmR4_14T, split=split, 
                  name = "Expression14T",  
                  column_title = "VAR14TNF", 
                  cluster_columns = FALSE,  show_row_names = FALSE,col = col_fun,width = unit(25, "mm"),
                  column_title_gp = gpar(fontsize = 10, fontface = "bold"))
hmap_RT <- Heatmap(dataHMmR4_RT, split=split, 
                  name = "ExpressionRT",  
                  column_title = "RBCTNF", 
                  cluster_columns = FALSE,  show_row_names = FALSE,col = col_fun,width = unit(25, "mm"),
                  column_title_gp = gpar(fontsize = 10, fontface = "bold"))
hmap_k+ha+hmap_k14+hmap_RT+hmap_k14T+hmap_37T  
 
 
   
 
 
 
 
 
 
  topDEgenes <- which(tempA$Include=="in")####find indexes
tempAkm<-tempA[ topDEgenes, ]
SymbolsKm<-dplyr::pull(tempAkm, Gene_Symbol)
#### export the gene expression data for the clusters
write.table(clusterMeans,paste0("ClusterMeansKm_",groupsName,".txt"),  sep = "\t")
ClusteredGenes<-data.frame(kclust6$cluster,SymbolsKm,dataHMm)
write.table(ClusteredGenes,paste0("ScaledDataInClustersKm_",groupsName,".txt"),  sep = "\t")
#head(ClusteredGenes)  
 
 
 
 
 
 
  bottomDEgenes<-which(tempA$Include=="out")####find indexes 
bottomG<-tempA[ bottomDEgenes, ]
bottomG<-dplyr::pull(bottomG, Gene_Symbol)
write.table(bottomG,paste0("ipaBottomKmeans_",groupsName,".txt"),  sep = "\t")
                         
topDEgenes <- which(tempA$Include=="in")####find indexes 
tempAkm<-tempA[ topDEgenes, ]
SymbolsKm<-dplyr::pull(tempAkm, Gene_Symbol)
ipaKmeans<-ClusteredGenes
#countsTable <-countsTable[,c(1:15)]####if samples need removing
ipaKmeans<-ipaKmeans[,c(1:2)]
ipaKmeans$name2<-rownames(ipaKmeans)
#ipaKmeans%>% rownames_to_column(var = "rowname")
#ipaKmeans
#rowid_to_column(ipaKmeans)
ipaKmeans = mutate(ipaKmeans, x1= ifelse(ipaKmeans$kclust6.cluster==1, "1", "0"))
ipaKmeans = mutate(ipaKmeans, x2= ifelse(ipaKmeans$kclust6.cluster==2, "1", "0"))
ipaKmeans = mutate(ipaKmeans, x3= ifelse(ipaKmeans$kclust6.cluster==3, "1", "0"))
ipaKmeans = mutate(ipaKmeans, x4= ifelse(ipaKmeans$kclust6.cluster==4, "1", "0"))
ipaKmeans = mutate(ipaKmeans, x5= ifelse(ipaKmeans$kclust6.cluster==5, "1", "0"))
ipaKmeans = mutate(ipaKmeans, x6= ifelse(ipaKmeans$kclust6.cluster==6, "1", "0"))
#ipaKmeans
write.table(ipaKmeans,paste0("ipaKmeans_",groupsName,".txt"),  sep = "\t")
#head(ipaKmeans)  
 
 
 
 
 
 
  ClusteredGenes2<-ClusteredGenes[c(1)]
#ClusteredGenes2
listAll<-list()
for(i in 1:6) {
  clusterName<-paste0("x",i)
  #clusterName<-row.names(subset(ClusteredGenes,ClusteredGenes==i))
  clusterName<-(subset(ClusteredGenes$SymbolsKm,ClusteredGenes==i))
  listAll[[i]]<-clusterName
}
#need to name the vectors in the list, example here is for 8 clusters
names(listAll)<-c("X1", "X2", "X3", "X4","X5", "X6")
#if you want to rearrange the order
#listAll<-listAll[c("x3", "x7", "x8", "x2", "x6", "x5", "x4", "x1")]
#lapply(listAll, head)  
 
 
 
 
 
 4. Annotation of K-means clusters 
 
 CC cellular compartment 
 BP biological process 
 MF molecular function 
 
 The simplify function has been used to cut down on GO redundancy 
 
 
 
  #str(AllGeneNames)  
 
 
 
 
 
 
  ####CC
cgoCC <- compareCluster(geneCluster = listAll, 
                      universe = AllGeneNames,
                      fun = "enrichGO",
                      OrgDb=org.Hs.eg.db, 
                      ####OrgDb=org.Mm.eg.db,
                      keyType="SYMBOL",
                      ont = "CC", 
                      pvalueCutoff=0.05,
                      qvalueCutoff = 0.10)
cgoCC2 <- simplify(cgoCC, cutoff=0.7, by="p.adjust", select_fun=min)
####write as spreadsheet
write.csv(as.data.frame(cgoCC2),paste0("GO_CC_",groupsName,".csv"))
dotplot(cgoCC2,showCategory = 30,
        title = paste0("GO Cellular Compartment ",groupsName))+
  theme(axis.text.x = element_text(angle = 90, vjust = 0.5, hjust=1))  
 
 
   
 
 
 
 Plots and GO data were written to files 
 
 
 
  png(paste0("GO_CC_",groupsName,".png"), width = 1224, height = 824)
dotplot(cgoCC2,showCategory = 30,
        title = paste0("GO Cellular Compartment ",groupsName))+
  theme(axis.text.x = element_text(angle = 90, vjust = 0.5, hjust=1))
dev.off()  
 
 
  null device 
          1   
 
 
 
 GO BP 
 
 
 
  ####CC
cgoBP <- compareCluster(geneCluster = listAll, 
                      universe = AllGeneNames,
                      fun = "enrichGO",
                      OrgDb=org.Hs.eg.db,
                      keyType="SYMBOL",
                      ont = "BP", 
                      pvalueCutoff=0.05,
                      qvalueCutoff = 0.10)
cgoBP2 <- simplify(cgoBP, cutoff=0.7, by="p.adjust", select_fun=min)
####write as spreadsheet
write.csv(as.data.frame(cgoBP2),paste0("GO_BP_",groupsName,".csv"))
dotplot(cgoBP2,showCategory = 30,
        title = paste0("GO Biological Process ",groupsName))+
  theme(axis.text.x = element_text(angle = 90, vjust = 0.5, hjust=1))  
 
 
   
 
 
 
 
 
 
  png(paste0("GO_BP_",groupsName,".png"), width = 1024, height = 1224)
dotplot(cgoBP2,showCategory = 30,
        title = paste0("GO Biological Process ",groupsName))+
  theme(axis.text.x = element_text(angle = 90, vjust = 0.5, hjust=1))
dev.off()  
 
 
  null device 
          1   
 
 
 
 GO MF 
 
 
 
  ####MF
cgoMF <- compareCluster(geneCluster = listAll, 
                      universe = AllGeneNames,
                      fun = "enrichGO",
                      OrgDb=org.Hs.eg.db, 
                      keyType="SYMBOL",
                      ont = "MF", 
                      pvalueCutoff=0.05,
                      qvalueCutoff = 0.10)
cgoMF2 <- simplify(cgoMF, cutoff=0.7, by="p.adjust", select_fun=min)
####write as spreadsheet
write.csv(as.data.frame(cgoMF2),paste0("GO_MF_",groupsName,".csv"))
dotplot(cgoMF2,showCategory = 30,
        title = paste0("GO Molecular Function  ",groupsName))+
  theme(axis.text.x = element_text(angle = 90, vjust = 0.5, hjust=1))  
 
 
   
 
 
 
 
 
 
  png(paste0("GO_MF_",groupsName,".png"), width = 1424, height = 824)
dotplot(cgoMF2,showCategory = 30,
        title = paste0("GO Molecular Function  ",groupsName))+
  theme(axis.text.x = element_text(angle = 90, vjust = 0.5, hjust=1))
dev.off()  
 
 
  null device 
          1   
 
 
 
 
 
 
 R4 VAR14 TNF k-means q0.05 
 
 1. Genelist Selection 
 
 
 
  groupsName<-"R4_VAR14_TNF_kmeans_q0.05"  
 
 
 
 
 
 
 
 
 
 
 
 
 
 
  tempA<-countsTable  
 
 
 
 
 
 
  topDEgenes <- which(tempA$padj_R4Var14TNF_Hours_2h_vs_0h<0.05&!is.na(tempA$padj_R4Var14TNF_Hours_2h_vs_0h))####find indexes 
listA<-tempA[ topDEgenes, ]$Gene_Symbol
topDEgenes <- which(tempA$padj_R4Var14TNF_Hours_6h_vs_0h<0.05&!is.na(tempA$padj_R4Var14TNF_Hours_6h_vs_0h))####find indexes 
listB<-tempA[ topDEgenes, ]$Gene_Symbol
topDEgenes <- which(tempA$padj_R4Var14TNF_Hours_20h_vs_0h<0.05&!is.na(tempA$padj_R4Var14TNF_Hours_20h_vs_0h))####find indexes 
listC<-tempA[ topDEgenes, ]$Gene_Symbol
topDEgenes <- which(tempA$padj_R4Var14TNF_Hours_6h_vs_2h<0.05&!is.na(tempA$padj_R4Var14TNF_Hours_6h_vs_2h))####find indexes 
listD<-tempA[ topDEgenes, ]$Gene_Symbol
topDEgenes <- which(tempA$padj_R4Var14TNF_Hours_20h_vs_6h<0.05&!is.na(tempA$padj_R4Var14TNF_Hours_20h_vs_6h))####find indexes 
listE<-tempA[ topDEgenes, ]$Gene_Symbol
vennq<-venn.diagram(x = list(listA,listB,listC,listD,listE) ,
            category.names = c("Var14TNF_2h_vs_0h","Var14TNF_6h_vs_0h","Var14TNF_20h_vs_0h","Var14TNF_6h_vs_2h","Var14TNF_20h_vs_6h"),
            main="padj<0.05",
            filename = NULL,  scaled = FALSE, fill = colorsV5, cat.col = colorsV5, cat.cex = 1, cat.dist=0.3,  margin = 0.3)
topDEgenes <- which(tempA$pvalue_R4Var14TNF_Hours_2h_vs_0h<0.05&abs(tempA$log2FoldChange_R4Var14TNF_Hours_2h_vs_0h)>1&!is.na(tempA$pvalue_R4Var14TNF_Hours_2h_vs_0h))####find indexes 
listA<-tempA[ topDEgenes, ]$Gene_Symbol
topDEgenes <- which(tempA$pvalue_R4Var14TNF_Hours_6h_vs_0h<0.05&abs(tempA$log2FoldChange_R4Var14TNF_Hours_6h_vs_0h)>1&!is.na(tempA$pvalue_R4Var14TNF_Hours_6h_vs_0h))####find indexes 
listB<-tempA[ topDEgenes, ]$Gene_Symbol
topDEgenes <- which(tempA$pvalue_R4Var14TNF_Hours_20h_vs_0h<0.05&abs(tempA$log2FoldChange_R4Var14TNF_Hours_20h_vs_0h)>1&!is.na(tempA$pvalue_R4Var14TNF_Hours_20h_vs_0h))####find indexes 
listC<-tempA[ topDEgenes, ]$Gene_Symbol
topDEgenes <- which(tempA$pvalue_R4Var14TNF_Hours_6h_vs_2h<0.05&abs(tempA$log2FoldChange_R4Var14TNF_Hours_6h_vs_2h)>1&!is.na(tempA$pvalue_R4Var14TNF_Hours_6h_vs_2h))####find indexes 
listD<-tempA[ topDEgenes, ]$Gene_Symbol
topDEgenes <- which(tempA$pvalue_R4Var14TNF_Hours_20h_vs_6h<0.05&abs(tempA$log2FoldChange_R4Var14TNF_Hours_20h_vs_6h)>1&!is.na(tempA$pvalue_R4Var14TNF_Hours_20h_vs_6h))####find indexes 
listE<-tempA[ topDEgenes, ]$Gene_Symbol
vennp<-venn.diagram(x = list(listA,listB,listC,listD,listE) ,
            category.names = c("Var14TNF_2h_vs_0h","Var14TNF_6h_vs_0h","Var14TNF_20h_vs_0h","Var14TNF_6h_vs_2h","Var14TNF_20h_vs_6h"),
            main="pvalue<0.05&fold change>2",
            filename = NULL,  scaled = FALSE, fill = colorsV5, cat.col = colorsV5, cat.cex = 1, cat.dist=0.3,  margin = 0.3)  
 
 
 
 
 
 
  topDEgenes <- which((tempA$padj_R4Var14TNF_Hours_2h_vs_0h<0.05&!is.na(tempA$padj_R4Var14TNF_Hours_2h_vs_0h))| 
(tempA$padj_R4Var14TNF_Hours_6h_vs_0h<0.05&!is.na(tempA$padj_R4Var14TNF_Hours_6h_vs_0h))|
(tempA$padj_R4Var14TNF_Hours_20h_vs_0h<0.05&!is.na(tempA$padj_R4Var14TNF_Hours_20h_vs_0h))| 
(tempA$padj_R4Var14TNF_Hours_6h_vs_2h<0.05&!is.na(tempA$padj_R4Var14TNF_Hours_6h_vs_2h))|
(tempA$padj_R4Var14TNF_Hours_20h_vs_6h<0.05&!is.na(tempA$padj_R4Var14TNF_Hours_20h_vs_6h)) 
)
listA<-tempA[ topDEgenes, ]$Gene_Symbol
topDEgenes <- which((tempA$pvalue_R4Var14TNF_Hours_2h_vs_0h<0.05&abs(tempA$log2FoldChange_R4Var14TNF_Hours_2h_vs_0h)>1&!is.na(tempA$pvalue_R4Var14TNF_Hours_2h_vs_0h))| 
(tempA$pvalue_R4Var14TNF_Hours_6h_vs_0h<0.05&abs(tempA$log2FoldChange_R4Var14TNF_Hours_6h_vs_0h)>1&!is.na(tempA$pvalue_R4Var14TNF_Hours_6h_vs_0h))| 
(tempA$pvalue_R4Var14TNF_Hours_20h_vs_0h<0.05&abs(tempA$log2FoldChange_R4Var14TNF_Hours_20h_vs_0h)>1&!is.na(tempA$pvalue_R4Var14TNF_Hours_20h_vs_0h))| 
(tempA$pvalue_R4Var14TNF_Hours_6h_vs_2h<0.05&abs(tempA$log2FoldChange_R4Var14TNF_Hours_6h_vs_2h)>1&!is.na(tempA$pvalue_R4Var14TNF_Hours_6h_vs_2h))| 
(tempA$pvalue_R4Var14TNF_Hours_20h_vs_6h<0.05&abs(tempA$log2FoldChange_R4Var14TNF_Hours_20h_vs_6h)>1&!is.na(tempA$pvalue_R4Var14TNF_Hours_20h_vs_6h))
 )####find indexes 
listC<-tempA[ topDEgenes, ]$Gene_Symbol
vennpq<-venn.diagram(x = list(listA,listC) ,
            category.names = c("padj<0.05","p<0.05&fc>2"),
            main="padj compared to pvalue",
            filename = NULL,  scaled = FALSE, fill = colorsV2, cat.col = colorsV2, cat.cex = 1, cat.dist=0.1,  margin = 0.15)  
 
 
 
 
 
 
  grid.arrange(gTree(children=vennq), gTree(children=vennpq) , ncol=2,top="R2 Var14 TNF")  
 
 
   
 
 
 
 
 
 
  #tempA<-resAll[-c(10:30) ]
tempA<-countsTable
#rownames(tempA)
rownames(tempA) <- NULL
tempA = mutate(tempA, Include=
                   ifelse(tempA$padj_R4Var14TNF_Hours_2h_vs_0h<0.05&!is.na(tempA$padj_R4Var14TNF_Hours_2h_vs_0h), "in",
                          ifelse(tempA$padj_R4Var14TNF_Hours_6h_vs_0h<0.05&!is.na(tempA$padj_R4Var14TNF_Hours_6h_vs_0h), "in",
                                 ifelse(tempA$padj_R4Var14TNF_Hours_20h_vs_0h<0.05&!is.na(tempA$padj_R4Var14TNF_Hours_20h_vs_0h), "in",
                                        ifelse(tempA$padj_R4Var14TNF_Hours_6h_vs_2h<0.05&!is.na(tempA$padj_R4Var14TNF_Hours_6h_vs_2h), "in",
                                               ifelse(tempA$padj_R4Var14TNF_Hours_20h_vs_6h<0.05&!is.na(tempA$padj_R4Var14TNF_Hours_20h_vs_6h), "in",
                                                                       "out"))))))
tempA  
 
 
 
 
 
 
 
  ####library(dplyr)
tempA %>%
     group_by(Include) %>% 
     tally()  
 
 
 
 
 
 
 
 
 
 
 
  topDEgenes <- which(tempA$Include=="in")####find indexes   
 
 
 
 
 
 NB Please check columns used and renamed for plots 
 
 
 
  baseMeansHm <-countsTable[,c(110:113)]
head(baseMeansHm)  
 
 
 
 
 
 
 
  colnames(baseMeansHm)<-c("Var14TNF_0h","Var14TNF_2h","Var14TNF_6h","Var14TNF_20h")
head(baseMeansHm)  
 
 
 
 
 
 
 
 
 
 
 NB Please check columns used and renamed for plots 
 
 
 
  dataHi <-countsTable[,c(37:47)]
head(dataHi)  
 
 
 
 
 
 
 
 
 
 
 
  dataHi <-countsTable[,c(37:47)]
colnames(dataHi)<-c("Var14TNF_0h_32","Var14TNF_2h_33","Var14TNF_6h_34","Var14TNF_20h_35","Var14TNF_2h_41","Var14TNF_6h_42","Var14TNF_20h_43","Var14TNF_0h_48","Var14TNF_2h_49","Var14TNF_6h_50","Var14TNF_20h_51")
head(dataHi)  
 
 
 
 
 
 
 
  dataHi<-dataHi[,c(1,8,2,5,9,3,6,10,4,7,11)]
head(dataHi)  
 
 
 
 
 
 
 
  dataHi<-dataHi[ topDEgenes, ]
dataHi <- log2(dataHi+1)
dataHi<- t(as.matrix(dataHi))
dataHi <- t(scale(dataHi))
####str(dataHi)  
 
 
 
 
 
 
  topDEgenes <- which(tempA$Include=="in")####find indexes   
 
 
 
 
 
 2. Hierachical clustering of means (individual samples added for inspection) 
 
 
 
  hmap_hier_factors1 <- Heatmap(
  dataHi,  name = "ExpressionI",
  column_title = paste0("Individual Samples"), 
  column_title_gp = gpar(fontsize = 16, fontface = "bold"),
  width = unit(300, "mm"),
  col = col_fun,
  cluster_rows = FALSE,
  cluster_columns = FALSE,
  show_row_names = FALSE)  
 
 
  `use_raster` is automatically set to TRUE for a matrix with more than 2000 rows. You can control `use_raster` arugment by explicitly setting TRUE/FALSE to it. Set
`ht_opt$message = FALSE` to turn off this message.  
 
 
    #top_annotation=colAnn  )
####means
dataHMm<-baseMeansHm[ topDEgenes, ]
dataHMm <- log2(dataHMm+1)
dataHMm<- t(as.matrix(dataHMm))
dataHMm <- t(scale(dataHMm))
#colAnnm <- HeatmapAnnotation(df=annm, which="col", col=coloursm, annotation_width=unit(c(2, 4), "cm"), gap=unit(1, "mm"))
hmap_hier_factors4 <- Heatmap(
  dataHMm,  name = "Expression",
  row_labels = paste0(rownames(dataHMm)," ",(tempA[ topDEgenes, ])$Gene_Symbol),
  column_title = paste0("Means"), 
  col = col_fun,
  column_title_gp = gpar(fontsize = 16, fontface = "bold"),
  width = unit(50, "mm"),
  cluster_columns = FALSE,
  show_row_names = FALSE)
  #top_annotation=colAnnm  )
hmap_hier_factors4+hmap_hier_factors1  
 
 
   
 
 
 
 
 
 
  par(mfrow=c(1,2))
#### Silhouette method
fviz_nbclust(dataHMm, kmeans, method = "silhouette",k.max = 16)+
  labs(subtitle = "Silhouette method")  
 
 
   
 
 
  #### Elbow method
fviz_nbclust(dataHMm, kmeans, method = "wss",k.max = 16) +
  labs(subtitle = "Elbow method")  
 
 
   
 
 
 
 
 
 
  ####gap stat slow!!!
####set.seed(123)
####fviz_nbclust(dataHMm, kmeans, nstart = 25,  method = "gap_stat", nboot = 100,k.max = 16)+
####  labs(subtitle = "Gap statistic method")  
 
 
 
 
 
 
  #kclust7 <- kmeans(dataHMm, 6)
#silhouette plot
distK<-daisy(dataHMm)
plot(silhouette(kclust7$cluster, distK), col=1:6, border=NA)  
 
 
   
 
 
 
 
 
 3. K-means clustering of means 
 
 
 
  split <- paste0("Cluster\n", kclust7$cluster)
#split <- factor(paste0("Cluster\n", kclust7$cluster), levels=c("Cluster\n5","Cluster\n6","Cluster\n2","Cluster\n1","Cluster\n4","Cluster\n3"))
hmap_k <- Heatmap(dataHMm, split=split, #cluster_row_slices = FALSE,
                  cluster_columns = FALSE,
                  show_row_names = FALSE,
                  name = "Expression",
                  col = col_fun,
                  width = unit(20, "mm"),
                  column_title = "means", 
                  column_title_gp = gpar(fontsize = 16, fontface = "bold")
                  
                        )#top_annotation=colAnn)
hmap_hier_factors1 <- Heatmap(
  dataHi,  name = "ExpressionI",
  col = col_fun,
  column_title = paste0("individual samples"), 
  column_title_gp = gpar(fontsize = 16, fontface = "bold"),
  width = unit(60, "mm"),
  cluster_rows = FALSE,
  cluster_columns = FALSE,
  show_row_names = FALSE)  
 
 
  `use_raster` is automatically set to TRUE for a matrix with more than 2000 rows. You can control `use_raster` arugment by explicitly setting TRUE/FALSE to it. Set
`ht_opt$message = FALSE` to turn off this message.  
 
 
  hmap_k  
 
 
   
 
 
 
 
 
 K-means clustering of means (with cluster annotation and individual samples added for inspection) 
 
 
 
  Response_Time<-data.frame(kclust7$cluster)
Response_Time = mutate(Response_Time, Response=
                   ifelse(Response_Time$kclust7.cluster==6, "transient", 
                          ifelse(Response_Time$kclust7.cluster==5, "transient",
                                 ifelse(Response_Time$kclust7.cluster==1, "transient",
                                        ifelse(Response_Time$kclust7.cluster==3, "transient",
                                               ifelse(Response_Time$kclust7.cluster==2, "transient",
                                                      ifelse(Response_Time$kclust7.cluster==4, "transient",
                                                                       "out")))))))
Response_Time<-Response_Time[c(2)]
rownames(Response_Time) <- NULL
ha = HeatmapAnnotation(df = Response_Time, which = "row", width = unit(1, "cm"),col = list(Response = c("early" =  "green3", "late" = "brown", "transient" = "violet")))
hmap_k+ha+hmap_hier_factors1  
 
 
   
 
 
 
 Mean profiles of clusters 
 
 
 
  clustercount<-data.frame(kclust7$cluster)
clustersizes<-table(clustercount$kclust7.cluster)
clusterMeans<-data.frame(kclust7$centers)
clusterMeans1<-data.frame(t(clusterMeans))
clusterMeans1 <- cbind(rownames(clusterMeans1), clusterMeans1)
orderN<-c("Var14TNF_0h","Var14TNF_2h","Var14TNF_6h","Var14TNF_20h")#### manual
rownames(clusterMeans1) <- NULL
names(clusterMeans1)[names(clusterMeans1)=="rownames(clusterMeans1)"] <- "Sample"
pX1<-ggplot(data=clusterMeans1, aes(x=Sample, y=X1,group=1)) +
  geom_line()+  geom_point()+ggtitle(paste("Cluster X1 Profile ",clustersizes[1]," genes"))+  scale_x_discrete(limits=orderN)+
  theme(axis.title.x = element_blank(),axis.title.y = element_blank())
pX2<-ggplot(data=clusterMeans1, aes(x=Sample, y=X2,group=1)) +
  geom_line()+  geom_point()+ggtitle(paste("Cluster X2 Profile ",clustersizes[2]," genes"))+  scale_x_discrete(limits=orderN)+
  theme(axis.title.x = element_blank(),axis.title.y = element_blank())
pX3<-ggplot(data=clusterMeans1, aes(x=Sample, y=X3,group=1)) +
  geom_line()+  geom_point()+ggtitle(paste("Cluster X3 Profile ",clustersizes[3]," genes"))+  scale_x_discrete(limits=orderN)+
  theme(axis.title.x = element_blank(),axis.title.y = element_blank())
pX4<-ggplot(data=clusterMeans1, aes(x=Sample, y=X4,group=1)) +
  geom_line()+  geom_point()+ggtitle(paste("Cluster X4 Profile ",clustersizes[4]," genes"))+  scale_x_discrete(limits=orderN)+
  theme(axis.title.x = element_blank(),axis.title.y = element_blank())
pX5<-ggplot(data=clusterMeans1, aes(x=Sample, y=X5,group=1)) +
  geom_line()+  geom_point()+ggtitle(paste("Cluster X5 Profile ",clustersizes[5]," genes"))+  scale_x_discrete(limits=orderN)+
  theme(axis.title.x = element_blank(),axis.title.y = element_blank())
pX6<-ggplot(data=clusterMeans1, aes(x=Sample, y=X6,group=1)) +
  geom_line()+  geom_point()+ggtitle(paste("Cluster X6 Profile ",clustersizes[6]," genes"))+  scale_x_discrete(limits=orderN)+
  theme(axis.title.x = element_blank(),axis.title.y = element_blank())
#plot
multiplot(pX1, pX2, pX3, pX4,pX5, pX6, cols=2)  
 
 
   
 
 
 
 
 
 K-means clustering of means (other treatment means added for inspection) 
 
 
 
  hmap_k <- Heatmap(dataHMm, split=split, cluster_row_slices = FALSE,
                  cluster_columns = FALSE,
                  show_row_names = FALSE,
                  name = "Expression",
                  col = col_fun,
                  width = unit(25, "mm"),
                  column_title = "Var14TNF", 
                  column_title_gp = gpar(fontsize = 10, fontface = "bold"))
                  
                  
baseMeansHmTemp <-countsTable[,c(48:50)]
colnames(baseMeansHmTemp)<-c("Var37TNF_0h","Var37TNF_6h","Var37TNF_20h")
dataHMmR1_37Y<-baseMeansHmTemp[ topDEgenes, ]
dataHMmR1_37Y <- log2(dataHMmR1_37Y+1)
dataHMmR1_37Y<- t(as.matrix(dataHMmR1_37Y))
dataHMmR1_37Y <- t(scale(dataHMmR1_37Y))
baseMeansHmTemp <-countsTable[,c(60:63)]
colnames(baseMeansHmTemp)<-c("Var14_0h","Var14_2h","Var14_6h","Var14_20h")
dataHMmR2_14<-baseMeansHmTemp[ topDEgenes, ]
dataHMmR2_14 <- log2(dataHMmR2_14+1)
dataHMmR2_14<- t(as.matrix(dataHMmR2_14))
dataHMmR2_14 <- t(scale(dataHMmR2_14))
baseMeansHmTemp <-countsTable[,c(79:82)]
colnames(baseMeansHmTemp)<-c("RBC_0h","RBC_2h","RBC_6h","RBC_20h")
dataHMmR2_R<-baseMeansHmTemp[ topDEgenes, ]
dataHMmR2_R <- log2(dataHMmR2_R+1)
dataHMmR2_R<- t(as.matrix(dataHMmR2_R))
dataHMmR2_R <- t(scale(dataHMmR2_R))
baseMeansHmTemp <-countsTable[,c(110:113)]
colnames(baseMeansHmTemp)<-c("Var14TNF_0h","Var14TNF_2h","Var14TNF_6h","Var14TNF_20h")
dataHMmR4_14T<-baseMeansHmTemp[ topDEgenes, ]
dataHMmR4_14T <- log2(dataHMmR4_14T+1)
dataHMmR4_14T<- t(as.matrix(dataHMmR4_14T))
dataHMmR4_14T <- t(scale(dataHMmR4_14T))
baseMeansHmTemp <-countsTable[,c(129:132)]
colnames(baseMeansHmTemp)<-c("RBC_TNF_0h","RBC_TNF_2h","RBC_TNF_6h","RBC_TNF_20h")
dataHMmR4_RT<-baseMeansHmTemp[ topDEgenes, ]
dataHMmR4_RT <- log2(dataHMmR4_RT+1)
dataHMmR4_RT<- t(as.matrix(dataHMmR4_RT))
dataHMmR4_RT <- t(scale(dataHMmR4_RT))
hmap_37T <- Heatmap(dataHMmR1_37Y, split=split, 
                  name = "Expression37T",  
                  column_title = "VAR37TNF", 
                  cluster_columns = FALSE,  show_row_names = FALSE,col = col_fun,width = unit(20, "mm"),
                  column_title_gp = gpar(fontsize = 10, fontface = "bold"))
hmap_k14 <- Heatmap(dataHMmR2_14, split=split, 
                  name = "Expression14",  
                  column_title = "VAR14noTNF", 
                  cluster_columns = FALSE,  show_row_names = FALSE,col = col_fun,width = unit(25, "mm"),
                  column_title_gp = gpar(fontsize = 10, fontface = "bold"))
hmap_R <- Heatmap(dataHMmR2_R, split=split, 
                  name = "ExpressionR",  
                  column_title = "RBCnoTNF", 
                  cluster_columns = FALSE,  show_row_names = FALSE,col = col_fun,width = unit(25, "mm"),
                  column_title_gp = gpar(fontsize = 10, fontface = "bold"))
hmap_k14T <- Heatmap(dataHMmR4_14T, split=split, 
                  name = "Expression14T",  
                  column_title = "VAR14TNF", 
                  cluster_columns = FALSE,  show_row_names = FALSE,col = col_fun,width = unit(25, "mm"),
                  column_title_gp = gpar(fontsize = 10, fontface = "bold"))
hmap_RT <- Heatmap(dataHMmR4_RT, split=split, 
                  name = "ExpressionRT",  
                  column_title = "RBCTNF", 
                  cluster_columns = FALSE,  show_row_names = FALSE,col = col_fun,width = unit(25, "mm"),
                  column_title_gp = gpar(fontsize = 10, fontface = "bold"))
hmap_k+ha+hmap_RT+hmap_37T+hmap_k14+hmap_R  
 
 
   
 
 
 
 
 
 
  topDEgenes <- which(tempA$Include=="in")####find indexes
tempAkm<-tempA[ topDEgenes, ]
SymbolsKm<-dplyr::pull(tempAkm, Gene_Symbol)
#### export the gene expression data for the clusters
write.table(clusterMeans,paste0("ClusterMeansKm_",groupsName,".txt"),  sep = "\t")
ClusteredGenes<-data.frame(kclust7$cluster,SymbolsKm,dataHMm)
write.table(ClusteredGenes,paste0("ScaledDataInClustersKm_",groupsName,".txt"),  sep = "\t")
#head(ClusteredGenes)  
 
 
 
 
 
 
  bottomDEgenes<-which(tempA$Include=="out")####find indexes 
bottomG<-tempA[ bottomDEgenes, ]
bottomG<-dplyr::pull(bottomG, Gene_Symbol)
write.table(bottomG,paste0("ipaBottomKmeans_",groupsName,".txt"),  sep = "\t")
                         
topDEgenes <- which(tempA$Include=="in")####find indexes 
tempAkm<-tempA[ topDEgenes, ]
SymbolsKm<-dplyr::pull(tempAkm, Gene_Symbol)
ipaKmeans<-ClusteredGenes
#countsTable <-countsTable[,c(1:15)]####if samples need removing
ipaKmeans<-ipaKmeans[,c(1:2)]
ipaKmeans$name2<-rownames(ipaKmeans)
#ipaKmeans%>% rownames_to_column(var = "rowname")
#ipaKmeans
#rowid_to_column(ipaKmeans)
ipaKmeans = mutate(ipaKmeans, x1= ifelse(ipaKmeans$kclust7.cluster==1, "1", "0"))
ipaKmeans = mutate(ipaKmeans, x2= ifelse(ipaKmeans$kclust7.cluster==2, "1", "0"))
ipaKmeans = mutate(ipaKmeans, x3= ifelse(ipaKmeans$kclust7.cluster==3, "1", "0"))
ipaKmeans = mutate(ipaKmeans, x4= ifelse(ipaKmeans$kclust7.cluster==4, "1", "0"))
ipaKmeans = mutate(ipaKmeans, x5= ifelse(ipaKmeans$kclust7.cluster==5, "1", "0"))
ipaKmeans = mutate(ipaKmeans, x6= ifelse(ipaKmeans$kclust7.cluster==6, "1", "0"))
#ipaKmeans
write.table(ipaKmeans,paste0("ipaKmeans_",groupsName,".txt"),  sep = "\t")
#head(ipaKmeans)  
 
 
 
 
 
 
  ClusteredGenes2<-ClusteredGenes[c(1)]
#ClusteredGenes2
listAll<-list()
for(i in 1:6) {
  clusterName<-paste0("x",i)
  #clusterName<-row.names(subset(ClusteredGenes,ClusteredGenes==i))
  clusterName<-(subset(ClusteredGenes$SymbolsKm,ClusteredGenes==i))
  listAll[[i]]<-clusterName
}
#need to name the vectors in the list, example here is for 8 clusters
names(listAll)<-c("X1", "X2", "X3", "X4","X5", "X6")
#if you want to rearrange the order
#listAll<-listAll[c("x3", "x7", "x8", "x2", "x6", "x5", "x4", "x1")]
#lapply(listAll, head)  
 
 
 
 
 
 4. Annotation of K-means clusters 
 
 CC cellular compartment 
 BP biological process 
 MF molecular function 
 
 The simplify function has been used to cut down on GO redundancy 
 
 
 
  #str(AllGeneNames)  
 
 
 
 
 
 
  ####CC
cgoCC <- compareCluster(geneCluster = listAll, 
                      universe = AllGeneNames,
                      fun = "enrichGO",
                      OrgDb=org.Hs.eg.db, 
                      ####OrgDb=org.Mm.eg.db,
                      keyType="SYMBOL",
                      ont = "CC", 
                      pvalueCutoff=0.05,
                      qvalueCutoff = 0.10)
cgoCC2 <- simplify(cgoCC, cutoff=0.7, by="p.adjust", select_fun=min)
####write as spreadsheet
write.csv(as.data.frame(cgoCC2),paste0("GO_CC_",groupsName,".csv"))
dotplot(cgoCC2,showCategory = 30,
        title = paste0("GO Cellular Compartment ",groupsName))+
  theme(axis.text.x = element_text(angle = 90, vjust = 0.5, hjust=1))  
 
 
   
 
 
 
 Plots and GO data were written to files 
 
 
 
  png(paste0("GO_CC_",groupsName,".png"), width = 1224, height = 824)
dotplot(cgoCC2,showCategory = 30,
        title = paste0("GO Cellular Compartment ",groupsName))+
  theme(axis.text.x = element_text(angle = 90, vjust = 0.5, hjust=1))
dev.off()  
 
 
  null device 
          1   
 
 
 
 GO BP 
 
 
 
  ####CC
cgoBP <- compareCluster(geneCluster = listAll, 
                      universe = AllGeneNames,
                      fun = "enrichGO",
                      OrgDb=org.Hs.eg.db,
                      keyType="SYMBOL",
                      ont = "BP", 
                      pvalueCutoff=0.05,
                      qvalueCutoff = 0.10)
cgoBP2 <- simplify(cgoBP, cutoff=0.7, by="p.adjust", select_fun=min)
####write as spreadsheet
write.csv(as.data.frame(cgoBP2),paste0("GO_BP_",groupsName,".csv"))
dotplot(cgoBP2,showCategory = 30,
        title = paste0("GO Biological Process ",groupsName))+
  theme(axis.text.x = element_text(angle = 90, vjust = 0.5, hjust=1))  
 
 
   
 
 
 
 
 
 
  png(paste0("GO_BP_",groupsName,".png"), width = 1024, height = 1224)
dotplot(cgoBP2,showCategory = 30,
        title = paste0("GO Biological Process ",groupsName))+
  theme(axis.text.x = element_text(angle = 90, vjust = 0.5, hjust=1))
dev.off()  
 
 
  null device 
          1   
 
 
 
 GO MF 
 
 
 
  ####MF
cgoMF <- compareCluster(geneCluster = listAll, 
                      universe = AllGeneNames,
                      fun = "enrichGO",
                      OrgDb=org.Hs.eg.db, 
                      keyType="SYMBOL",
                      ont = "MF", 
                      pvalueCutoff=0.05,
                      qvalueCutoff = 0.10)
cgoMF2 <- simplify(cgoMF, cutoff=0.7, by="p.adjust", select_fun=min)
####write as spreadsheet
write.csv(as.data.frame(cgoMF2),paste0("GO_MF_",groupsName,".csv"))
dotplot(cgoMF2,showCategory = 30,
        title = paste0("GO Molecular Function  ",groupsName))+
  theme(axis.text.x = element_text(angle = 90, vjust = 0.5, hjust=1))  
 
 
   
 
 
 
 
 
 
  png(paste0("GO_MF_",groupsName,".png"), width = 1424, height = 624)
dotplot(cgoMF2,showCategory = 30,
        title = paste0("GO Molecular Function  ",groupsName))+
  theme(axis.text.x = element_text(angle = 90, vjust = 0.5, hjust=1))
dev.off()  
 
 
  null device 
          1   
 
 
 
 
 
 
 R4 VAR14 TNF k-means p0.05fc2 
 
 1. Genelist Selection 
 
 
 
  groupsName<-"R4_Var14TNF_kmeans_p0.05fc2"  
 
 
 
 
 
 
  countsTable<-read.delim("RNAseq2019July_5.txt", header = TRUE, sep = "\t",check.names=FALSE,row.names=1)
head(countsTable)  
 
 
 
 
 
 
 
 
 
 
 
  AllGeneNames<-countsTable$Gene_Symbol
#head(AllGeneNames)  
 
 
 
 
 
 
  grid.arrange(gTree(children=vennp), gTree(children=vennpq) , ncol=2,top="R4 Var14 TNF")  
 
 
   
 
 
 
 
 
 
  #tempA<-resAll[-c(10:30) ]
tempA<-countsTable
#rownames(tempA)
rownames(tempA) <- NULL
tempA = mutate(tempA, Include=
                   ifelse(tempA$pvalue_R4Var14TNF_Hours_2h_vs_0h<0.05&abs(tempA$log2FoldChange_R4Var14TNF_Hours_2h_vs_0h)>1&!is.na(tempA$pvalue_R4Var14TNF_Hours_2h_vs_0h), "in",
                          ifelse(tempA$pvalue_R4Var14TNF_Hours_6h_vs_0h<0.05&abs(tempA$log2FoldChange_R4Var14TNF_Hours_6h_vs_0h)>1&!is.na(tempA$pvalue_R4Var14TNF_Hours_6h_vs_0h), "in",
                                 ifelse(tempA$pvalue_R4Var14TNF_Hours_20h_vs_0h<0.05&abs(tempA$log2FoldChange_R4Var14TNF_Hours_20h_vs_0h)>1&!is.na(tempA$pvalue_R4Var14TNF_Hours_20h_vs_0h), "in",
                                        ifelse(tempA$pvalue_R4Var14TNF_Hours_6h_vs_2h<0.05&abs(tempA$log2FoldChange_R4Var14TNF_Hours_6h_vs_2h)>1&!is.na(tempA$pvalue_R4Var14TNF_Hours_6h_vs_2h), "in",
                                               ifelse(tempA$pvalue_R4Var14TNF_Hours_20h_vs_6h<0.05&abs(tempA$log2FoldChange_R4Var14TNF_Hours_20h_vs_6h)>1&!is.na(tempA$pvalue_R4Var14TNF_Hours_20h_vs_6h), "in",
                                                                       "out"))))))
tempA  
 
 
 
 
 
 
 
  ####library(dplyr)
tempA %>%
     group_by(Include) %>% 
     tally()  
 
 
 
 
 
 
 
 
 
 
 
  topDEgenes <- which(tempA$Include=="in")####find indexes   
 
 
 
 
 
 NB Please check columns used and renamed for plots 
 
 
 
  baseMeansHm <-countsTable[,c(110:113)]
head(baseMeansHm)  
 
 
 
 
 
 
 
  colnames(baseMeansHm)<-c("Var14TNF_0h","Var14TNF_2h","Var14TNF_6h","Var14TNF_20h")
head(baseMeansHm)  
 
 
 
 
 
 
 
 
 
 
 
  dataHi <-countsTable[,c(37:47)]
head(dataHi)  
 
 
 
 
 
 
 
 
 
 
 
  dataHi <-countsTable[,c(37:47)]
colnames(dataHi)<-c("Var14TNF_0h_32","Var14TNF_2h_33","Var14TNF_6h_34","Var14TNF_20h_35","Var14TNF_2h_41","Var14TNF_6h_42","Var14TNF_20h_43","Var14TNF_0h_48","Var14TNF_2h_49","Var14TNF_6h_50","Var14TNF_20h_51")
head(dataHi)  
 
 
 
 
 
 
 
  dataHi<-dataHi[,c(1,8,2,5,9,3,6,10,4,7,11)]
head(dataHi)  
 
 
 
 
 
 
 
  dataHi<-dataHi[ topDEgenes, ]
dataHi <- log2(dataHi+1)
dataHi<- t(as.matrix(dataHi))
dataHi <- t(scale(dataHi))
####str(dataHi)  
 
 
 
 
 
 
  topDEgenes <- which(tempA$Include=="in")####find indexes   
 
 
 
 
 
 2. Hierachical clustering of means 
 
 
 
  hmap_hier_factors1 <- Heatmap(
  dataHi,  name = "ExpressionI",
  column_title = paste0("Individual Samples"), 
  column_title_gp = gpar(fontsize = 16, fontface = "bold"),
  width = unit(300, "mm"),
  col = col_fun,
  cluster_rows = FALSE,
  cluster_columns = FALSE,
  show_row_names = FALSE)  
 
 
  `use_raster` is automatically set to TRUE for a matrix with more than 2000 rows. You can control `use_raster` arugment by explicitly setting TRUE/FALSE to it. Set
`ht_opt$message = FALSE` to turn off this message.  
 
 
    #top_annotation=colAnn  )
####means
dataHMm<-baseMeansHm[ topDEgenes, ]
dataHMm <- log2(dataHMm+1)
dataHMm<- t(as.matrix(dataHMm))
dataHMm <- t(scale(dataHMm))
#colAnnm <- HeatmapAnnotation(df=annm, which="col", col=coloursm, annotation_width=unit(c(2, 4), "cm"), gap=unit(1, "mm"))
hmap_hier_factors4 <- Heatmap(
  dataHMm,  name = "Expression",
  row_labels = paste0(rownames(dataHMm)," ",(tempA[ topDEgenes, ])$Gene_Symbol),
  column_title = paste0("Means"), 
  col = col_fun,
  column_title_gp = gpar(fontsize = 16, fontface = "bold"),
  width = unit(50, "mm"),
  cluster_columns = FALSE,
  show_row_names = FALSE)
  #top_annotation=colAnnm  )
hmap_hier_factors4+hmap_hier_factors1  
 
 
   
 
 
 
 
 
 
  par(mfrow=c(1,2))
#### Silhouette method
fviz_nbclust(dataHMm, kmeans, method = "silhouette",k.max = 16)+
  labs(subtitle = "Silhouette method")  
 
 
  did not converge in 10 iterations  
 
 
   
 
 
  #### Elbow method
fviz_nbclust(dataHMm, kmeans, method = "wss",k.max = 16) +
  labs(subtitle = "Elbow method")  
 
 
   
 
 
 
 
 
 
  ####gap stat slow!!!
####set.seed(123)
####fviz_nbclust(dataHMm, kmeans, nstart = 25,  method = "gap_stat", nboot = 100,k.max = 16)+
####  labs(subtitle = "Gap statistic method")  
 
 
 
 
 
 
  #kclust8 <- kmeans(dataHMm, 6)
#silhouette plot
distK<-daisy(dataHMm)
plot(silhouette(kclust8$cluster, distK), col=1:6, border=NA)  
 
 
   
 
 
 
 
 
 3. K-means clustering of means 
 
 
 
  split <- paste0("Cluster\n", kclust8$cluster)
#split <- factor(paste0("Cluster\n", kclust8$cluster), levels=c("Cluster\n5","Cluster\n4","Cluster\n3","Cluster\n1","Cluster\n6","Cluster\n2"))
hmap_k <- Heatmap(dataHMm, split=split, #cluster_row_slices = FALSE,
                  cluster_columns = FALSE,
                  show_row_names = FALSE,
                  name = "Expression",
                  col = col_fun,
                  width = unit(20, "mm"),
                  column_title = "means", 
                  column_title_gp = gpar(fontsize = 16, fontface = "bold")
                  
                        )#top_annotation=colAnn)
hmap_hier_factors1 <- Heatmap(
  dataHi,  name = "ExpressionI",
  col = col_fun,
  column_title = paste0("individual samples"), 
  column_title_gp = gpar(fontsize = 16, fontface = "bold"),
  width = unit(60, "mm"),
  cluster_rows = FALSE,
  cluster_columns = FALSE,
  show_row_names = FALSE)  
 
 
  `use_raster` is automatically set to TRUE for a matrix with more than 2000 rows. You can control `use_raster` arugment by explicitly setting TRUE/FALSE to it. Set
`ht_opt$message = FALSE` to turn off this message.  
 
 
  hmap_k  
 
 
   
 
 
 
 
 
 K-means clustering of means (with cluster annotation and individual samples added for inspection) 
 
 
 
  Response_Time<-data.frame(kclust8$cluster)
Response_Time = mutate(Response_Time, Response=
                   ifelse(Response_Time$kclust8.cluster==3, "transient", 
                          ifelse(Response_Time$kclust8.cluster==1, "transient",
                                 ifelse(Response_Time$kclust8.cluster==4, "transient",
                                        ifelse(Response_Time$kclust8.cluster==5, "transient",
                                               ifelse(Response_Time$kclust8.cluster==2, "transient",
                                                      ifelse(Response_Time$kclust8.cluster==6, "transient",
                                                                       "out")))))))
Response_Time<-Response_Time[c(2)]
rownames(Response_Time) <- NULL
ha = HeatmapAnnotation(df = Response_Time, which = "row", width = unit(1, "cm"),col = list(Response = c("early" =  "green3", "late" = "brown", "transient" = "violet")))
hmap_k+ha+hmap_hier_factors1  
 
 
   
 
 
 
 Mean profiles of clusters 
 
 
 
  clustercount<-data.frame(kclust8$cluster)
clustersizes<-table(clustercount$kclust8.cluster)
clusterMeans<-data.frame(kclust8$centers)
clusterMeans1<-data.frame(t(clusterMeans))
clusterMeans1 <- cbind(rownames(clusterMeans1), clusterMeans1)
orderN<-c("Var14TNF_0h","Var14TNF_2h","Var14TNF_6h","Var14TNF_20h")#### manual
rownames(clusterMeans1) <- NULL
names(clusterMeans1)[names(clusterMeans1)=="rownames(clusterMeans1)"] <- "Sample"
####clusterMeans1
pX1<-ggplot(data=clusterMeans1, aes(x=Sample, y=X1,group=1)) +
  geom_line()+  geom_point()+ggtitle(paste("Cluster X1 Profile ",clustersizes[1]," genes"))+  scale_x_discrete(limits=orderN)+
  theme(axis.title.x = element_blank(),axis.title.y = element_blank())
pX2<-ggplot(data=clusterMeans1, aes(x=Sample, y=X2,group=1)) +
  geom_line()+  geom_point()+ggtitle(paste("Cluster X2 Profile ",clustersizes[2]," genes"))+  scale_x_discrete(limits=orderN)+
  theme(axis.title.x = element_blank(),axis.title.y = element_blank())
pX3<-ggplot(data=clusterMeans1, aes(x=Sample, y=X3,group=1)) +
  geom_line()+  geom_point()+ggtitle(paste("Cluster X3 Profile ",clustersizes[3]," genes"))+  scale_x_discrete(limits=orderN)+
  theme(axis.title.x = element_blank(),axis.title.y = element_blank())
pX4<-ggplot(data=clusterMeans1, aes(x=Sample, y=X4,group=1)) +
  geom_line()+  geom_point()+ggtitle(paste("Cluster X4 Profile ",clustersizes[4]," genes"))+  scale_x_discrete(limits=orderN)+
  theme(axis.title.x = element_blank(),axis.title.y = element_blank())
pX5<-ggplot(data=clusterMeans1, aes(x=Sample, y=X5,group=1)) +
  geom_line()+  geom_point()+ggtitle(paste("Cluster X5 Profile ",clustersizes[5]," genes"))+  scale_x_discrete(limits=orderN)+
  theme(axis.title.x = element_blank(),axis.title.y = element_blank())
pX6<-ggplot(data=clusterMeans1, aes(x=Sample, y=X6,group=1)) +
  geom_line()+  geom_point()+ggtitle(paste("Cluster X6 Profile ",clustersizes[6]," genes"))+  scale_x_discrete(limits=orderN)+
  theme(axis.title.x = element_blank(),axis.title.y = element_blank())
#plot
multiplot(pX1, pX2, pX3, pX4,pX5, pX6, cols=2)  
 
 
   
 
 
 
 
 
 K-means clustering of means (other treatment means added for inspection) 
 
 
 
  hmap_k <- Heatmap(dataHMm, split=split, cluster_row_slices = FALSE,
                  cluster_columns = FALSE,
                  show_row_names = FALSE,
                  name = "Expression",
                  col = col_fun,
                  width = unit(25, "mm"),
                  column_title = "Var14TNF", 
                  column_title_gp = gpar(fontsize = 10, fontface = "bold"))
                  
                  
baseMeansHmTemp <-countsTable[,c(48:50)]
colnames(baseMeansHmTemp)<-c("Var37TNF_0h","Var37TNF_6h","Var37TNF_20h")
dataHMmR1_37Y<-baseMeansHmTemp[ topDEgenes, ]
dataHMmR1_37Y <- log2(dataHMmR1_37Y+1)
dataHMmR1_37Y<- t(as.matrix(dataHMmR1_37Y))
dataHMmR1_37Y <- t(scale(dataHMmR1_37Y))
baseMeansHmTemp <-countsTable[,c(60:63)]
colnames(baseMeansHmTemp)<-c("Var14_0h","Var14_2h","Var14_6h","Var14_20h")
dataHMmR2_14<-baseMeansHmTemp[ topDEgenes, ]
dataHMmR2_14 <- log2(dataHMmR2_14+1)
dataHMmR2_14<- t(as.matrix(dataHMmR2_14))
dataHMmR2_14 <- t(scale(dataHMmR2_14))
baseMeansHmTemp <-countsTable[,c(79:82)]
colnames(baseMeansHmTemp)<-c("RBC_0h","RBC_2h","RBC_6h","RBC_20h")
dataHMmR2_R<-baseMeansHmTemp[ topDEgenes, ]
dataHMmR2_R <- log2(dataHMmR2_R+1)
dataHMmR2_R<- t(as.matrix(dataHMmR2_R))
dataHMmR2_R <- t(scale(dataHMmR2_R))
baseMeansHmTemp <-countsTable[,c(110:113)]
colnames(baseMeansHmTemp)<-c("Var14TNF_0h","Var14TNF_2h","Var14TNF_6h","Var14TNF_20h")
dataHMmR4_14T<-baseMeansHmTemp[ topDEgenes, ]
dataHMmR4_14T <- log2(dataHMmR4_14T+1)
dataHMmR4_14T<- t(as.matrix(dataHMmR4_14T))
dataHMmR4_14T <- t(scale(dataHMmR4_14T))
baseMeansHmTemp <-countsTable[,c(129:132)]
colnames(baseMeansHmTemp)<-c("RBC_TNF_0h","RBC_TNF_2h","RBC_TNF_6h","RBC_TNF_20h")
dataHMmR4_RT<-baseMeansHmTemp[ topDEgenes, ]
dataHMmR4_RT <- log2(dataHMmR4_RT+1)
dataHMmR4_RT<- t(as.matrix(dataHMmR4_RT))
dataHMmR4_RT <- t(scale(dataHMmR4_RT))
hmap_37T <- Heatmap(dataHMmR1_37Y, split=split, 
                  name = "Expression37T",  
                  column_title = "VAR37TNF", 
                  cluster_columns = FALSE,  show_row_names = FALSE,col = col_fun,width = unit(20, "mm"),
                  column_title_gp = gpar(fontsize = 10, fontface = "bold"))
hmap_k14 <- Heatmap(dataHMmR2_14, split=split, 
                  name = "Expression14",  
                  column_title = "VAR14noTNF", 
                  cluster_columns = FALSE,  show_row_names = FALSE,col = col_fun,width = unit(25, "mm"),
                  column_title_gp = gpar(fontsize = 10, fontface = "bold"))
hmap_R <- Heatmap(dataHMmR2_R, split=split, 
                  name = "ExpressionR",  
                  column_title = "RBCnoTNF", 
                  cluster_columns = FALSE,  show_row_names = FALSE,col = col_fun,width = unit(25, "mm"),
                  column_title_gp = gpar(fontsize = 10, fontface = "bold"))
hmap_k14T <- Heatmap(dataHMmR4_14T, split=split, 
                  name = "Expression14T",  
                  column_title = "VAR14TNF", 
                  cluster_columns = FALSE,  show_row_names = FALSE,col = col_fun,width = unit(25, "mm"),
                  column_title_gp = gpar(fontsize = 10, fontface = "bold"))
hmap_RT <- Heatmap(dataHMmR4_RT, split=split, 
                  name = "ExpressionRT",  
                  column_title = "RBCTNF", 
                  cluster_columns = FALSE,  show_row_names = FALSE,col = col_fun,width = unit(25, "mm"),
                  column_title_gp = gpar(fontsize = 10, fontface = "bold"))
hmap_k+ha+hmap_RT+hmap_37T+hmap_k14+hmap_R  
 
 
   
 
 
 
 
 
 
  topDEgenes <- which(tempA$Include=="in")####find indexes
tempAkm<-tempA[ topDEgenes, ]
SymbolsKm<-dplyr::pull(tempAkm, Gene_Symbol)
#### export the gene expression data for the clusters
write.table(clusterMeans,paste0("ClusterMeansKm_",groupsName,".txt"),  sep = "\t")
ClusteredGenes<-data.frame(kclust8$cluster,SymbolsKm,dataHMm)
write.table(ClusteredGenes,paste0("ScaledDataInClustersKm_",groupsName,".txt"),  sep = "\t")
#head(ClusteredGenes)  
 
 
 
 
 
 
  bottomDEgenes<-which(tempA$Include=="out")####find indexes 
bottomG<-tempA[ bottomDEgenes, ]
bottomG<-dplyr::pull(bottomG, Gene_Symbol)
write.table(bottomG,paste0("ipaBottomKmeans_",groupsName,".txt"),  sep = "\t")
                         
topDEgenes <- which(tempA$Include=="in")####find indexes 
tempAkm<-tempA[ topDEgenes, ]
SymbolsKm<-dplyr::pull(tempAkm, Gene_Symbol)
ipaKmeans<-ClusteredGenes
#countsTable <-countsTable[,c(1:15)]####if samples need removing
ipaKmeans<-ipaKmeans[,c(1:2)]
ipaKmeans$name2<-rownames(ipaKmeans)
#ipaKmeans%>% rownames_to_column(var = "rowname")
#ipaKmeans
#rowid_to_column(ipaKmeans)
ipaKmeans = mutate(ipaKmeans, x1= ifelse(ipaKmeans$kclust8.cluster==1, "1", "0"))
ipaKmeans = mutate(ipaKmeans, x2= ifelse(ipaKmeans$kclust8.cluster==2, "1", "0"))
ipaKmeans = mutate(ipaKmeans, x3= ifelse(ipaKmeans$kclust8.cluster==3, "1", "0"))
ipaKmeans = mutate(ipaKmeans, x4= ifelse(ipaKmeans$kclust8.cluster==4, "1", "0"))
ipaKmeans = mutate(ipaKmeans, x5= ifelse(ipaKmeans$kclust8.cluster==5, "1", "0"))
ipaKmeans = mutate(ipaKmeans, x6= ifelse(ipaKmeans$kclust8.cluster==6, "1", "0"))
#ipaKmeans
write.table(ipaKmeans,paste0("ipaKmeans_",groupsName,".txt"),  sep = "\t")
#head(ipaKmeans)  
 
 
 
 
 
 
  ClusteredGenes2<-ClusteredGenes[c(1)]
#ClusteredGenes2
listAll<-list()
for(i in 1:6) {
  clusterName<-paste0("x",i)
  #clusterName<-row.names(subset(ClusteredGenes,ClusteredGenes==i))
  clusterName<-(subset(ClusteredGenes$SymbolsKm,ClusteredGenes==i))
  listAll[[i]]<-clusterName
}
#need to name the vectors in the list, example here is for 8 clusters
names(listAll)<-c("X1", "X2", "X3", "X4","X5", "X6")
#if you want to rearrange the order
#listAll<-listAll[c("x3", "x7", "x8", "x2", "x6", "x5", "x4", "x1")]
#lapply(listAll, head)  
 
 
 
 
 
 4. Annotation of K-means clusters 
 
 CC cellular compartment 
 BP biological process 
 MF molecular function 
 
 The simplify function has been used to cut down on GO redundancy 
 
 
 
  #str(AllGeneNames)  
 
 
 
 
 
 
  ####CC
cgoCC <- compareCluster(geneCluster = listAll, 
                      universe = AllGeneNames,
                      fun = "enrichGO",
                      OrgDb=org.Hs.eg.db, 
                      ####OrgDb=org.Mm.eg.db,
                      keyType="SYMBOL",
                      ont = "CC", 
                      pvalueCutoff=0.05,
                      qvalueCutoff = 0.10)
cgoCC2 <- simplify(cgoCC, cutoff=0.7, by="p.adjust", select_fun=min)
####write as spreadsheet
write.csv(as.data.frame(cgoCC2),paste0("GO_CC_",groupsName,".csv"))
dotplot(cgoCC2,showCategory = 30,
        title = paste0("GO Cellular Compartment ",groupsName))+
  theme(axis.text.x = element_text(angle = 90, vjust = 0.5, hjust=1))  
 
 
   
 
 
 
 Plots and GO data were written to files 
 
 
 
  png(paste0("GO_CC_",groupsName,".png"), width = 1224, height = 824)
dotplot(cgoCC2,showCategory = 30,
        title = paste0("GO Cellular Compartment ",groupsName))+
  theme(axis.text.x = element_text(angle = 90, vjust = 0.5, hjust=1))
dev.off()  
 
 
  null device 
          1   
 
 
 
 GO BP 
 
 
 
  ####CC
cgoBP <- compareCluster(geneCluster = listAll, 
                      universe = AllGeneNames,
                      fun = "enrichGO",
                      OrgDb=org.Hs.eg.db,
                      keyType="SYMBOL",
                      ont = "BP", 
                      pvalueCutoff=0.05,
                      qvalueCutoff = 0.10)
cgoBP2 <- simplify(cgoBP, cutoff=0.7, by="p.adjust", select_fun=min)
####write as spreadsheet
write.csv(as.data.frame(cgoBP2),paste0("GO_BP_",groupsName,".csv"))
dotplot(cgoBP2,showCategory = 30,
        title = paste0("GO Biological Process ",groupsName))+
  theme(axis.text.x = element_text(angle = 90, vjust = 0.5, hjust=1))  
 
 
   
 
 
 
 
 
 
  png(paste0("GO_BP_",groupsName,".png"), width = 1024, height = 1224)
dotplot(cgoBP2,showCategory = 30,
        title = paste0("GO Biological Process ",groupsName))+
  theme(axis.text.x = element_text(angle = 90, vjust = 0.5, hjust=1))
dev.off()  
 
 
  null device 
          1   
 
 
 
 GO MF 
 
 
 
  ####MF
cgoMF <- compareCluster(geneCluster = listAll, 
                      universe = AllGeneNames,
                      fun = "enrichGO",
                      OrgDb=org.Hs.eg.db, 
                      keyType="SYMBOL",
                      ont = "MF", 
                      pvalueCutoff=0.05,
                      qvalueCutoff = 0.10)
cgoMF2 <- simplify(cgoMF, cutoff=0.7, by="p.adjust", select_fun=min)
####write as spreadsheet
write.csv(as.data.frame(cgoMF2),paste0("GO_MF_",groupsName,".csv"))
dotplot(cgoMF2,showCategory = 30,
        title = paste0("GO Molecular Function  ",groupsName))+
  theme(axis.text.x = element_text(angle = 90, vjust = 0.5, hjust=1))  
 
 
   
 
 
 
 
 
 
  png(paste0("GO_MF_",groupsName,".png"), width = 1424, height = 824)
dotplot(cgoMF2,showCategory = 30,
        title = paste0("GO Molecular Function  ",groupsName))+
  theme(axis.text.x = element_text(angle = 90, vjust = 0.5, hjust=1))
dev.off()  
 
 
  null device 
          1   
 
 
 
 
 
 
 R4 RBC TNF k-means q0.05 
 
 1. Genelist Selection 
 
 
 
  groupsName<-"R4_RBC_TNF_kmeans_q0.05"  
 
 
 
 
 
 
 
 
 
 
 
 
 
 
  tempA<-countsTable  
 
 
 
 
 
 
  topDEgenes <- which(tempA$padj_R4RBC_TNF_Hours_2h_vs_0h<0.05&!is.na(tempA$padj_R4RBC_TNF_Hours_2h_vs_0h))####find indexes 
listA<-tempA[ topDEgenes, ]$Gene_Symbol
topDEgenes <- which(tempA$padj_R4RBC_TNF_Hours_6h_vs_0h<0.05&!is.na(tempA$padj_R4RBC_TNF_Hours_6h_vs_0h))####find indexes 
listB<-tempA[ topDEgenes, ]$Gene_Symbol
topDEgenes <- which(tempA$padj_R4RBC_TNF_Hours_20h_vs_0h<0.05&!is.na(tempA$padj_R4RBC_TNF_Hours_20h_vs_0h))####find indexes 
listC<-tempA[ topDEgenes, ]$Gene_Symbol
topDEgenes <- which(tempA$padj_R4RBC_TNF_Hours_6h_vs_2h<0.05&!is.na(tempA$padj_R4RBC_TNF_Hours_6h_vs_2h))####find indexes 
listD<-tempA[ topDEgenes, ]$Gene_Symbol
topDEgenes <- which(tempA$padj_R4RBC_TNF_Hours_20h_vs_6h<0.05&!is.na(tempA$padj_R4RBC_TNF_Hours_20h_vs_6h))####find indexes 
listE<-tempA[ topDEgenes, ]$Gene_Symbol
vennq<-venn.diagram(x = list(listA,listB,listC,listD,listE) ,
            category.names = c("RBC_TNF_2h_vs_0h","RBC_TNF_6h_vs_0h","RBC_TNF_20h_vs_0h","RBC_TNF_6h_vs_2h","RBC_TNF_20h_vs_6h"),
            main="padj<0.05",
            filename = NULL,  scaled = FALSE, fill = colorsV5, cat.col = colorsV5, cat.cex = 1, cat.dist=0.3,  margin = 0.3)
topDEgenes <- which(tempA$pvalue_R4RBC_TNF_Hours_2h_vs_0h<0.05&abs(tempA$log2FoldChange_R4RBC_TNF_Hours_2h_vs_0h)>1&!is.na(tempA$pvalue_R4RBC_TNF_Hours_2h_vs_0h))####find indexes 
listA<-tempA[ topDEgenes, ]$Gene_Symbol
topDEgenes <- which(tempA$pvalue_R4RBC_TNF_Hours_6h_vs_0h<0.05&abs(tempA$log2FoldChange_R4RBC_TNF_Hours_6h_vs_0h)>1&!is.na(tempA$pvalue_R4RBC_TNF_Hours_6h_vs_0h))####find indexes 
listB<-tempA[ topDEgenes, ]$Gene_Symbol
topDEgenes <- which(tempA$pvalue_R4RBC_TNF_Hours_20h_vs_0h<0.05&abs(tempA$log2FoldChange_R4RBC_TNF_Hours_20h_vs_0h)>1&!is.na(tempA$pvalue_R4RBC_TNF_Hours_20h_vs_0h))####find indexes 
listC<-tempA[ topDEgenes, ]$Gene_Symbol
topDEgenes <- which(tempA$pvalue_R4RBC_TNF_Hours_6h_vs_2h<0.05&abs(tempA$log2FoldChange_R4RBC_TNF_Hours_6h_vs_2h)>1&!is.na(tempA$pvalue_R4RBC_TNF_Hours_6h_vs_2h))####find indexes 
listD<-tempA[ topDEgenes, ]$Gene_Symbol
topDEgenes <- which(tempA$pvalue_R4RBC_TNF_Hours_20h_vs_6h<0.05&abs(tempA$log2FoldChange_R4RBC_TNF_Hours_20h_vs_6h)>1&!is.na(tempA$pvalue_R4RBC_TNF_Hours_20h_vs_6h))####find indexes 
listE<-tempA[ topDEgenes, ]$Gene_Symbol
vennp<-venn.diagram(x = list(listA,listB,listC,listD,listE) ,
            category.names = c("RBC_TNF_2h_vs_0h","RBC_TNF_6h_vs_0h","RBC_TNF_20h_vs_0h","RBC_TNF_6h_vs_2h","RBC_TNF_20h_vs_6h"),
            main="pvalue<0.05&fold change>2",
            filename = NULL,  scaled = FALSE, fill = colorsV5, cat.col = colorsV5, cat.cex = 1, cat.dist=0.3,  margin = 0.3)  
 
 
 
 
 
 
  topDEgenes <- which((tempA$padj_R4RBC_TNF_Hours_2h_vs_0h<0.05&!is.na(tempA$padj_R4RBC_TNF_Hours_2h_vs_0h))| 
(tempA$padj_R4RBC_TNF_Hours_6h_vs_0h<0.05&!is.na(tempA$padj_R4RBC_TNF_Hours_6h_vs_0h))|
(tempA$padj_R4RBC_TNF_Hours_20h_vs_0h<0.05&!is.na(tempA$padj_R4RBC_TNF_Hours_20h_vs_0h))| 
(tempA$padj_R4RBC_TNF_Hours_6h_vs_2h<0.05&!is.na(tempA$padj_R4RBC_TNF_Hours_6h_vs_2h))|
(tempA$padj_R4RBC_TNF_Hours_20h_vs_6h<0.05&!is.na(tempA$padj_R4RBC_TNF_Hours_20h_vs_6h)) 
)
listA<-tempA[ topDEgenes, ]$Gene_Symbol
topDEgenes <- which((tempA$pvalue_R4RBC_TNF_Hours_2h_vs_0h<0.05&abs(tempA$log2FoldChange_R4RBC_TNF_Hours_2h_vs_0h)>1&!is.na(tempA$pvalue_R4RBC_TNF_Hours_2h_vs_0h))| 
(tempA$pvalue_R4RBC_TNF_Hours_6h_vs_0h<0.05&abs(tempA$log2FoldChange_R4RBC_TNF_Hours_6h_vs_0h)>1&!is.na(tempA$pvalue_R4RBC_TNF_Hours_6h_vs_0h))| 
(tempA$pvalue_R4RBC_TNF_Hours_20h_vs_0h<0.05&abs(tempA$log2FoldChange_R4RBC_TNF_Hours_20h_vs_0h)>1&!is.na(tempA$pvalue_R4RBC_TNF_Hours_20h_vs_0h))| 
(tempA$pvalue_R4RBC_TNF_Hours_6h_vs_2h<0.05&abs(tempA$log2FoldChange_R4RBC_TNF_Hours_6h_vs_2h)>1&!is.na(tempA$pvalue_R4RBC_TNF_Hours_6h_vs_2h))| 
(tempA$pvalue_R4RBC_TNF_Hours_20h_vs_6h<0.05&abs(tempA$log2FoldChange_R4RBC_TNF_Hours_20h_vs_6h)>1&!is.na(tempA$pvalue_R4RBC_TNF_Hours_20h_vs_6h))
 )####find indexes 
listC<-tempA[ topDEgenes, ]$Gene_Symbol
vennpq<-venn.diagram(x = list(listA,listC) ,
            category.names = c("padj<0.05","p<0.05&fc>2"),
            main="padj compared to pvalue",
            filename = NULL,  scaled = FALSE, fill = colorsV2, cat.col = colorsV2, cat.cex = 1, cat.dist=0.1,  margin = 0.15)  
 
 
 
 
 
 
  grid.arrange(gTree(children=vennq), gTree(children=vennpq) , ncol=2,top="R2 RBC TNF")  
 
 
   
 
 
 
 
 
 
  tempA<-countsTable  
 
 
 
 
 
 
  #tempA<-resAll[-c(10:30) ]
tempA<-countsTable
#rownames(tempA)
rownames(tempA) <- NULL
tempA = mutate(tempA, Include=
                   ifelse(tempA$padj_R4RBC_TNF_Hours_2h_vs_0h<0.05&!is.na(tempA$padj_R4RBC_TNF_Hours_2h_vs_0h), "in",
                          ifelse(tempA$padj_R4RBC_TNF_Hours_6h_vs_0h<0.05&!is.na(tempA$padj_R4RBC_TNF_Hours_6h_vs_0h), "in",
                                 ifelse(tempA$padj_R4RBC_TNF_Hours_20h_vs_0h<0.05&!is.na(tempA$padj_R4RBC_TNF_Hours_20h_vs_0h), "in",
                                        ifelse(tempA$padj_R4RBC_TNF_Hours_6h_vs_2h<0.05&!is.na(tempA$padj_R4RBC_TNF_Hours_6h_vs_2h), "in",
                                               ifelse(tempA$padj_R4RBC_TNF_Hours_20h_vs_6h<0.05&!is.na(tempA$padj_R4RBC_TNF_Hours_20h_vs_6h), "in",
                                                                       "out"))))))
tempA  
 
 
 
 
 
 
 
  ####library(dplyr)
tempA %>%
     group_by(Include) %>% 
     tally()  
 
 
 
 
 
 
 
 
 
 
 
  topDEgenes <- which(tempA$Include=="in")####find indexes   
 
 
 
 
 
 NB Please check columns used and renamed for plots 
 
 
 
  baseMeansHm <-countsTable[,c(129:132)]
head(baseMeansHm)  
 
 
 
 
 
 
 
  colnames(baseMeansHm)<-c("RBC_TNF_0h","RBC_TNF_2h","RBC_TNF_6h","RBC_TNF_20h")
head(baseMeansHm)  
 
 
 
 
 
 
 
 
 
 
 
  dataHi <-countsTable[,c(27:36)]
head(dataHi)  
 
 
 
 
 
 
 
  colnames(dataHi)<-c("RBC_TNF_0h_28","RBC_TNF_2h_29","RBC_TNF_6h_30","RBC_TNF_20h_31","RBC_TNF_6h_38","RBC_TNF_20h_39","RBC_TNF_0h_44","RBC_TNF_2h_45","RBC_TNF_6h_46","RBC_TNF_20h_47")
head(dataHi)  
 
 
 
 
 
 
 
  dataHi<-dataHi[,c(1,7,2,8,3,5,9,4,6,10)]
head(dataHi)  
 
 
 
 
 
 
 
  dataHi<-dataHi[ topDEgenes, ]
dataHi <- log2(dataHi+1)
dataHi<- t(as.matrix(dataHi))
dataHi <- t(scale(dataHi))
####str(dataHi)  
 
 
 
 
 
 
  topDEgenes <- which(tempA$Include=="in")####find indexes   
 
 
 
 
 
 2. Hierachical clustering of means (individual samples added for inspection) 
 
 
 
  hmap_hier_factors1 <- Heatmap(
  dataHi,  name = "ExpressionI",
  column_title = paste0("Individual Samples"), 
  column_title_gp = gpar(fontsize = 16, fontface = "bold"),
  width = unit(300, "mm"),
  col = col_fun,
  cluster_rows = FALSE,
  cluster_columns = FALSE,
  show_row_names = FALSE)
  #top_annotation=colAnn  )
####means
dataHMm<-baseMeansHm[ topDEgenes, ]
dataHMm <- log2(dataHMm+1)
dataHMm<- t(as.matrix(dataHMm))
dataHMm <- t(scale(dataHMm))
#colAnnm <- HeatmapAnnotation(df=annm, which="col", col=coloursm, annotation_width=unit(c(2, 4), "cm"), gap=unit(1, "mm"))
hmap_hier_factors4 <- Heatmap(
  dataHMm,  name = "Expression",
  row_labels = paste0(rownames(dataHMm)," ",(tempA[ topDEgenes, ])$Gene_Symbol),
  column_title = paste0("Means"), 
  col = col_fun,
  column_title_gp = gpar(fontsize = 16, fontface = "bold"),
  width = unit(50, "mm"),
  cluster_columns = FALSE,
  show_row_names = FALSE)
  #top_annotation=colAnnm  )
hmap_hier_factors4+hmap_hier_factors1  
 
 
   
 
 
 
 
 
 
  par(mfrow=c(1,2))
#### Silhouette method
fviz_nbclust(dataHMm, kmeans, method = "silhouette",k.max = 16)+
  labs(subtitle = "Silhouette method")  
 
 
   
 
 
  #### Elbow method
fviz_nbclust(dataHMm, kmeans, method = "wss",k.max = 16) +
  labs(subtitle = "Elbow method")  
 
 
   
 
 
 
 
 
 
  ####gap stat slow!!!
####set.seed(123)
####fviz_nbclust(dataHMm, kmeans, nstart = 25,  method = "gap_stat", nboot = 100,k.max = 16)+
####  labs(subtitle = "Gap statistic method")  
 
 
 
 
 
 
  #kclust9 <- kmeans(dataHMm, 6)
#silhouette plot
distK<-daisy(dataHMm)
plot(silhouette(kclust9$cluster, distK), col=1:6, border=NA)  
 
 
   
 
 
 
 
 
 3. K-means clustering of means 
 
 
 
  split <- paste0("Cluster\n", kclust9$cluster)
#split <- factor(paste0("Cluster\n", kclust9$cluster), levels=c("Cluster\n1","Cluster\n6","Cluster\n3","Cluster\n5","Cluster\n6","Cluster\n1"))
hmap_k <- Heatmap(dataHMm, split=split,# cluster_row_slices = FALSE,
                  cluster_columns = FALSE,
                  show_row_names = FALSE,
                  name = "Expression",
                  col = col_fun,
                  width = unit(20, "mm"),
                  column_title = "means", 
                  column_title_gp = gpar(fontsize = 16, fontface = "bold")
                  
                        )#top_annotation=colAnn)
hmap_hier_factors1 <- Heatmap(
  dataHi,  name = "ExpressionI",
  col = col_fun,
  column_title = paste0("individual samples"), 
  column_title_gp = gpar(fontsize = 16, fontface = "bold"),
  width = unit(60, "mm"),
  cluster_rows = FALSE,
  cluster_columns = FALSE,
  show_row_names = FALSE)
hmap_k  
 
 
   
 
 
 
 
 
 K-means clustering of means (with cluster annotation and individual samples added for inspection) 
 
 
 
  Response_Time<-data.frame(kclust9$cluster)
Response_Time = mutate(Response_Time, Response=
                   ifelse(Response_Time$kclust9.cluster==3, "transient", 
                          ifelse(Response_Time$kclust9.cluster==4, "transient",
                                 ifelse(Response_Time$kclust9.cluster==1, "transient",
                                        ifelse(Response_Time$kclust9.cluster==5, "transient",
                                               ifelse(Response_Time$kclust9.cluster==2, "transient",
                                                      ifelse(Response_Time$kclust9.cluster==6, "transient",
                                                                       "out")))))))
Response_Time<-Response_Time[c(2)]
rownames(Response_Time) <- NULL
ha = HeatmapAnnotation(df = Response_Time, which = "row", width = unit(1, "cm"),col = list(Response = c("early" =  "green3", "late" = "brown", "transient" = "violet")))
hmap_k+ha+hmap_hier_factors1  
 
 
   
 
 
 
 Mean profiles of clusters 
 
 
 
  clustercount<-data.frame(kclust9$cluster)
clustersizes<-table(clustercount$kclust9.cluster)
clusterMeans<-data.frame(kclust9$centers)
clusterMeans1<-data.frame(t(clusterMeans))
clusterMeans1 <- cbind(rownames(clusterMeans1), clusterMeans1)
orderN<-c("RBC_TNF_0h","RBC_TNF_2h","RBC_TNF_6h","RBC_TNF_20h")#### manual
rownames(clusterMeans1) <- NULL
names(clusterMeans1)[names(clusterMeans1)=="rownames(clusterMeans1)"] <- "Sample"
####clusterMeans1
pX1<-ggplot(data=clusterMeans1, aes(x=Sample, y=X1,group=1)) +
  geom_line()+  geom_point()+ggtitle(paste("Cluster X1 Profile ",clustersizes[1]," genes"))+  scale_x_discrete(limits=orderN)+
  theme(axis.title.x = element_blank(),axis.title.y = element_blank())
pX2<-ggplot(data=clusterMeans1, aes(x=Sample, y=X2,group=1)) +
  geom_line()+  geom_point()+ggtitle(paste("Cluster X2 Profile ",clustersizes[2]," genes"))+  scale_x_discrete(limits=orderN)+
  theme(axis.title.x = element_blank(),axis.title.y = element_blank())
pX3<-ggplot(data=clusterMeans1, aes(x=Sample, y=X3,group=1)) +
  geom_line()+  geom_point()+ggtitle(paste("Cluster X3 Profile ",clustersizes[3]," genes"))+  scale_x_discrete(limits=orderN)+
  theme(axis.title.x = element_blank(),axis.title.y = element_blank())
pX4<-ggplot(data=clusterMeans1, aes(x=Sample, y=X4,group=1)) +
  geom_line()+  geom_point()+ggtitle(paste("Cluster X4 Profile ",clustersizes[4]," genes"))+  scale_x_discrete(limits=orderN)+
  theme(axis.title.x = element_blank(),axis.title.y = element_blank())
pX5<-ggplot(data=clusterMeans1, aes(x=Sample, y=X5,group=1)) +
  geom_line()+  geom_point()+ggtitle(paste("Cluster X5 Profile ",clustersizes[5]," genes"))+  scale_x_discrete(limits=orderN)+
  theme(axis.title.x = element_blank(),axis.title.y = element_blank())
pX6<-ggplot(data=clusterMeans1, aes(x=Sample, y=X6,group=1)) +
  geom_line()+  geom_point()+ggtitle(paste("Cluster X6 Profile ",clustersizes[6]," genes"))+  scale_x_discrete(limits=orderN)+
  theme(axis.title.x = element_blank(),axis.title.y = element_blank())
#plot
multiplot(pX1, pX2, pX3, pX4,pX5, pX6, cols=2)  
 
 
   
 
 
 
 
 
 K-means clustering of means (other treatment means added for inspection) 
 
 
 
  hmap_k <- Heatmap(dataHMm, split=split, cluster_row_slices = FALSE,
                  cluster_columns = FALSE,
                  show_row_names = FALSE,
                  name = "Expression",
                  col = col_fun,
                  width = unit(25, "mm"),
                  column_title = "RBCTNF", 
                  column_title_gp = gpar(fontsize = 10, fontface = "bold"))
                  
                  
baseMeansHmTemp <-countsTable[,c(48:50)]
colnames(baseMeansHmTemp)<-c("Var37TNF_0h","Var37TNF_6h","Var37TNF_20h")
dataHMmR1_37Y<-baseMeansHmTemp[ topDEgenes, ]
dataHMmR1_37Y <- log2(dataHMmR1_37Y+1)
dataHMmR1_37Y<- t(as.matrix(dataHMmR1_37Y))
dataHMmR1_37Y <- t(scale(dataHMmR1_37Y))
baseMeansHmTemp <-countsTable[,c(60:63)]
colnames(baseMeansHmTemp)<-c("Var14_0h","Var14_2h","Var14_6h","Var14_20h")
dataHMmR2_14<-baseMeansHmTemp[ topDEgenes, ]
dataHMmR2_14 <- log2(dataHMmR2_14+1)
dataHMmR2_14<- t(as.matrix(dataHMmR2_14))
dataHMmR2_14 <- t(scale(dataHMmR2_14))
baseMeansHmTemp <-countsTable[,c(79:82)]
colnames(baseMeansHmTemp)<-c("RBC_0h","RBC_2h","RBC_6h","RBC_20h")
dataHMmR2_R<-baseMeansHmTemp[ topDEgenes, ]
dataHMmR2_R <- log2(dataHMmR2_R+1)
dataHMmR2_R<- t(as.matrix(dataHMmR2_R))
dataHMmR2_R <- t(scale(dataHMmR2_R))
baseMeansHmTemp <-countsTable[,c(110:113)]
colnames(baseMeansHmTemp)<-c("Var14TNF_0h","Var14TNF_2h","Var14TNF_6h","Var14TNF_20h")
dataHMmR4_14T<-baseMeansHmTemp[ topDEgenes, ]
dataHMmR4_14T <- log2(dataHMmR4_14T+1)
dataHMmR4_14T<- t(as.matrix(dataHMmR4_14T))
dataHMmR4_14T <- t(scale(dataHMmR4_14T))
baseMeansHmTemp <-countsTable[,c(129:132)]
colnames(baseMeansHmTemp)<-c("RBC_TNF_0h","RBC_TNF_2h","RBC_TNF_6h","RBC_TNF_20h")
dataHMmR4_RT<-baseMeansHmTemp[ topDEgenes, ]
dataHMmR4_RT <- log2(dataHMmR4_RT+1)
dataHMmR4_RT<- t(as.matrix(dataHMmR4_RT))
dataHMmR4_RT <- t(scale(dataHMmR4_RT))
hmap_37T <- Heatmap(dataHMmR1_37Y, split=split, 
                  name = "Expression37T",  
                  column_title = "VAR37TNF", 
                  cluster_columns = FALSE,  show_row_names = FALSE,col = col_fun,width = unit(20, "mm"),
                  column_title_gp = gpar(fontsize = 10, fontface = "bold"))
hmap_k14 <- Heatmap(dataHMmR2_14, split=split, 
                  name = "Expression14",  
                  column_title = "VAR14noTNF", 
                  cluster_columns = FALSE,  show_row_names = FALSE,col = col_fun,width = unit(25, "mm"),
                  column_title_gp = gpar(fontsize = 10, fontface = "bold"))
hmap_R <- Heatmap(dataHMmR2_R, split=split, 
                  name = "ExpressionR",  
                  column_title = "RBCnoTNF", 
                  cluster_columns = FALSE,  show_row_names = FALSE,col = col_fun,width = unit(25, "mm"),
                  column_title_gp = gpar(fontsize = 10, fontface = "bold"))
hmap_k14T <- Heatmap(dataHMmR4_14T, split=split, 
                  name = "Expression14T",  
                  column_title = "VAR14TNF", 
                  cluster_columns = FALSE,  show_row_names = FALSE,col = col_fun,width = unit(25, "mm"),
                  column_title_gp = gpar(fontsize = 10, fontface = "bold"))
hmap_RT <- Heatmap(dataHMmR4_RT, split=split, 
                  name = "ExpressionRT",  
                  column_title = "RBCTNF", 
                  cluster_columns = FALSE,  show_row_names = FALSE,col = col_fun,width = unit(25, "mm"),
                  column_title_gp = gpar(fontsize = 10, fontface = "bold"))
hmap_k+ha+hmap_k14T+hmap_37T+hmap_k14+hmap_R  
 
 
   
 
 
 
 
 
 
  topDEgenes <- which(tempA$Include=="in")####find indexes
tempAkm<-tempA[ topDEgenes, ]
SymbolsKm<-dplyr::pull(tempAkm, Gene_Symbol)
#### export the gene expression data for the clusters
write.table(clusterMeans,paste0("ClusterMeansKm_",groupsName,".txt"),  sep = "\t")
ClusteredGenes<-data.frame(kclust9$cluster,SymbolsKm,dataHMm)
write.table(ClusteredGenes,paste0("ScaledDataInClustersKm_",groupsName,".txt"),  sep = "\t")
#head(ClusteredGenes)  
 
 
 
 
 
 
  bottomDEgenes<-which(tempA$Include=="out")####find indexes 
bottomG<-tempA[ bottomDEgenes, ]
bottomG<-dplyr::pull(bottomG, Gene_Symbol)
write.table(bottomG,paste0("ipaBottomKmeans_",groupsName,".txt"),  sep = "\t")
                         
topDEgenes <- which(tempA$Include=="in")####find indexes 
tempAkm<-tempA[ topDEgenes, ]
SymbolsKm<-dplyr::pull(tempAkm, Gene_Symbol)
ipaKmeans<-ClusteredGenes
#countsTable <-countsTable[,c(1:15)]####if samples need removing
ipaKmeans<-ipaKmeans[,c(1:2)]
ipaKmeans$name2<-rownames(ipaKmeans)
#ipaKmeans%>% rownames_to_column(var = "rowname")
#ipaKmeans
#rowid_to_column(ipaKmeans)
ipaKmeans = mutate(ipaKmeans, x1= ifelse(ipaKmeans$kclust9.cluster==1, "1", "0"))
ipaKmeans = mutate(ipaKmeans, x2= ifelse(ipaKmeans$kclust9.cluster==2, "1", "0"))
ipaKmeans = mutate(ipaKmeans, x3= ifelse(ipaKmeans$kclust9.cluster==3, "1", "0"))
ipaKmeans = mutate(ipaKmeans, x4= ifelse(ipaKmeans$kclust9.cluster==4, "1", "0"))
ipaKmeans = mutate(ipaKmeans, x5= ifelse(ipaKmeans$kclust9.cluster==5, "1", "0"))
ipaKmeans = mutate(ipaKmeans, x6= ifelse(ipaKmeans$kclust9.cluster==6, "1", "0"))
#ipaKmeans
write.table(ipaKmeans,paste0("ipaKmeans_",groupsName,".txt"),  sep = "\t")
#head(ipaKmeans)  
 
 
 
 
 
 
  ClusteredGenes2<-ClusteredGenes[c(1)]
ClusteredGenes2  
 
 
 
 
 
 
 
  listAll<-list()
for(i in 1:6) {
  clusterName<-paste0("x",i)
  #clusterName<-row.names(subset(ClusteredGenes,ClusteredGenes==i))
  clusterName<-(subset(ClusteredGenes$SymbolsKm,ClusteredGenes==i))
  listAll[[i]]<-clusterName
}
#need to name the vectors in the list, example here is for 8 clusters
names(listAll)<-c("X1", "X2", "X3", "X4","X5", "X6")
#if you want to rearrange the order
#listAll<-listAll[c("x3", "x7", "x8", "x2", "x6", "x5", "x4", "x1")]
#lapply(listAll, head)  
 
 
 
 
 
 4. Annotation of K-means clusters 
 
 CC cellular compartment 
 BP biological process 
 MF molecular function 
 
 The simplify function has been used to cut down on GO redundancy 
 
 
 
  #str(AllGeneNames)  
 
 
 
 
 
 
  ####CC
cgoCC <- compareCluster(geneCluster = listAll, 
                      universe = AllGeneNames,
                      fun = "enrichGO",
                      OrgDb=org.Hs.eg.db, 
                      ####OrgDb=org.Mm.eg.db,
                      keyType="SYMBOL",
                      ont = "CC", 
                      pvalueCutoff=0.05,
                      qvalueCutoff = 0.10)
cgoCC2 <- simplify(cgoCC, cutoff=0.7, by="p.adjust", select_fun=min)
####write as spreadsheet
write.csv(as.data.frame(cgoCC2),paste0("GO_CC_",groupsName,".csv"))
dotplot(cgoCC2,showCategory = 30,
        title = paste0("GO Cellular Compartment ",groupsName))+
  theme(axis.text.x = element_text(angle = 90, vjust = 0.5, hjust=1))  
 
 
   
 
 
 
 Plots and GO data were written to files 
 
 
 
  png(paste0("GO_CC_",groupsName,".png"), width = 1224, height = 624)
dotplot(cgoCC2,showCategory = 30,
        title = paste0("GO Cellular Compartment ",groupsName))+
  theme(axis.text.x = element_text(angle = 90, vjust = 0.5, hjust=1))
dev.off()  
 
 
  null device 
          1   
 
 
 
 GO BP 
 
 
 
  ####CC
cgoBP <- compareCluster(geneCluster = listAll, 
                      universe = AllGeneNames,
                      fun = "enrichGO",
                      OrgDb=org.Hs.eg.db,
                      keyType="SYMBOL",
                      ont = "BP", 
                      pvalueCutoff=0.05,
                      qvalueCutoff = 0.10)
cgoBP2 <- simplify(cgoBP, cutoff=0.7, by="p.adjust", select_fun=min)
####write as spreadsheet
write.csv(as.data.frame(cgoBP2),paste0("GO_BP_",groupsName,".csv"))
dotplot(cgoBP2,showCategory = 30,
        title = paste0("GO Biological Process ",groupsName))+
  theme(axis.text.x = element_text(angle = 90, vjust = 0.5, hjust=1))  
 
 
   
 
 
 
 
 
 
  png(paste0("GO_BP_",groupsName,".png"), width = 1024, height = 1624)
dotplot(cgoBP2,showCategory = 30,
        title = paste0("GO Biological Process ",groupsName))+
  theme(axis.text.x = element_text(angle = 90, vjust = 0.5, hjust=1))
dev.off()  
 
 
  null device 
          1   
 
 
 
 GO MF 
 
 
 
  ####MF
cgoMF <- compareCluster(geneCluster = listAll, 
                      universe = AllGeneNames,
                      fun = "enrichGO",
                      OrgDb=org.Hs.eg.db, 
                      keyType="SYMBOL",
                      ont = "MF", 
                      pvalueCutoff=0.05,
                      qvalueCutoff = 0.10)
cgoMF2 <- simplify(cgoMF, cutoff=0.7, by="p.adjust", select_fun=min)
####write as spreadsheet
write.csv(as.data.frame(cgoMF2),paste0("GO_MF_",groupsName,".csv"))
dotplot(cgoMF2,showCategory = 30,
        title = paste0("GO Molecular Function  ",groupsName))+
  theme(axis.text.x = element_text(angle = 90, vjust = 0.5, hjust=1))  
 
 
   
 
 
 
 
 
 
  png(paste0("GO_MF_",groupsName,".png"), width = 1424, height = 824)
dotplot(cgoMF2,showCategory = 30,
        title = paste0("GO Molecular Function  ",groupsName))+
  theme(axis.text.x = element_text(angle = 90, vjust = 0.5, hjust=1))
dev.off()  
 
 
  null device 
          1   
 
 
 
 
 
 
 R4 RBC TNF k-means p0.05fc2 
 
 1. Genelist Selection 
 
 
 
  groupsName<-"R4_RBC_TNF_kmeans_p0.05fc2"  
 
 
 
 
 
 
  countsTable<-read.delim("RNAseq2019July_5.txt", header = TRUE, sep = "\t",check.names=FALSE,row.names=1)
head(countsTable)  
 
 
 
 
 
 
 
 
 
 
 
  AllGeneNames<-countsTable$Gene_Symbol
#head(AllGeneNames)  
 
 
 
 
 
 
  grid.arrange(gTree(children=vennp), gTree(children=vennpq) , ncol=2,top="R4 RBC TNF")  
 
 
   
 
 
 
 
 
 
  #tempA<-resAll[-c(10:30) ]
tempA<-countsTable
#rownames(tempA)
rownames(tempA) <- NULL
tempA = mutate(tempA, Include=
                   ifelse(tempA$pvalue_R4RBC_TNF_Hours_2h_vs_0h<0.05&abs(tempA$log2FoldChange_R4RBC_TNF_Hours_2h_vs_0h)>1&!is.na(tempA$pvalue_R4RBC_TNF_Hours_2h_vs_0h), "in",
                          ifelse(tempA$pvalue_R4RBC_TNF_Hours_6h_vs_0h<0.05&abs(tempA$log2FoldChange_R4RBC_TNF_Hours_6h_vs_0h)>1&!is.na(tempA$pvalue_R4RBC_TNF_Hours_6h_vs_0h), "in",
                                 ifelse(tempA$pvalue_R4RBC_TNF_Hours_20h_vs_0h<0.05&abs(tempA$log2FoldChange_R4RBC_TNF_Hours_20h_vs_0h)>1&!is.na(tempA$pvalue_R4RBC_TNF_Hours_20h_vs_0h), "in",
                                        ifelse(tempA$pvalue_R4RBC_TNF_Hours_6h_vs_2h<0.05&abs(tempA$log2FoldChange_R4RBC_TNF_Hours_6h_vs_2h)>1&!is.na(tempA$pvalue_R4RBC_TNF_Hours_6h_vs_2h), "in",
                                               ifelse(tempA$pvalue_R4RBC_TNF_Hours_20h_vs_6h<0.05&abs(tempA$log2FoldChange_R4RBC_TNF_Hours_20h_vs_6h)>1&!is.na(tempA$pvalue_R4RBC_TNF_Hours_20h_vs_6h), "in",
                                                                       "out"))))))
tempA  
 
 
 
 
 
 
 
  ####library(dplyr)
tempA %>%
     group_by(Include) %>% 
     tally()  
 
 
 
 
 
 
 
 
 
 
 
  topDEgenes <- which(tempA$Include=="in")####find indexes   
 
 
 
 
 
 NB Please check columns used and renamed for plots 
 
 
 
  baseMeansHm <-countsTable[,c(129:132)]
head(baseMeansHm)  
 
 
 
 
 
 
 
  colnames(baseMeansHm)<-c("RBC_TNF_0h","RBC_TNF_2h","RBC_TNF_6h","RBC_TNF_20h")
head(baseMeansHm)  
 
 
 
 
 
 
 
 
 
 
 
  dataHi <-countsTable[,c(27:36)]
head(dataHi)  
 
 
 
 
 
 
 
  colnames(dataHi)<-c("RBC_TNF_0h_28","RBC_TNF_2h_29","RBC_TNF_6h_30","RBC_TNF_20h_31","RBC_TNF_6h_38","RBC_TNF_20h_39","RBC_TNF_0h_44","RBC_TNF_2h_45","RBC_TNF_6h_46","RBC_TNF_20h_47")
head(dataHi)  
 
 
 
 
 
 
 
  dataHi<-dataHi[,c(1,7,2,8,3,5,9,4,6,10)]
head(dataHi)  
 
 
 
 
 
 
 
  dataHi<-dataHi[ topDEgenes, ]
dataHi <- log2(dataHi+1)
dataHi<- t(as.matrix(dataHi))
dataHi <- t(scale(dataHi))
####str(dataHi)  
 
 
 
 
 
 
  topDEgenes <- which(tempA$Include=="in")####find indexes   
 
 
 
 
 
 2. Hierachical clustering of means (individual samples added for inspection) 
 
 
 
  hmap_hier_factors1 <- Heatmap(
  dataHi,  name = "ExpressionI",
  column_title = paste0("Individual Samples"), 
  column_title_gp = gpar(fontsize = 16, fontface = "bold"),
  width = unit(300, "mm"),
  col = col_fun,
  cluster_rows = FALSE,
  cluster_columns = FALSE,
  show_row_names = FALSE)
  #top_annotation=colAnn  )
####means
dataHMm<-baseMeansHm[ topDEgenes, ]
dataHMm <- log2(dataHMm+1)
dataHMm<- t(as.matrix(dataHMm))
dataHMm <- t(scale(dataHMm))
#colAnnm <- HeatmapAnnotation(df=annm, which="col", col=coloursm, annotation_width=unit(c(2, 4), "cm"), gap=unit(1, "mm"))
hmap_hier_factors4 <- Heatmap(
  dataHMm,  name = "Expression",
  row_labels = paste0(rownames(dataHMm)," ",(tempA[ topDEgenes, ])$Gene_Symbol),
  column_title = paste0("Means"), 
  col = col_fun,
  column_title_gp = gpar(fontsize = 16, fontface = "bold"),
  width = unit(50, "mm"),
  cluster_columns = FALSE,
  show_row_names = FALSE)
  #top_annotation=colAnnm  )
hmap_hier_factors4+hmap_hier_factors1  
 
 
   
 
 
 
 
 
 
  par(mfrow=c(1,2))
#### Silhouette method
fviz_nbclust(dataHMm, kmeans, method = "silhouette",k.max = 16)+
  labs(subtitle = "Silhouette method")  
 
 
   
 
 
  #### Elbow method
fviz_nbclust(dataHMm, kmeans, method = "wss",k.max = 16) +
  labs(subtitle = "Elbow method")  
 
 
   
 
 
 
 
 
 
  ####gap stat slow!!!
####set.seed(123)
####fviz_nbclust(dataHMm, kmeans, nstart = 25,  method = "gap_stat", nboot = 100,k.max = 16)+
####  labs(subtitle = "Gap statistic method")  
 
 
 
 
 
 
  #kclust10 <- kmeans(dataHMm, 6)
#silhouette plot
distK<-daisy(dataHMm)
plot(silhouette(kclust10$cluster, distK), col=1:6, border=NA)  
 
 
   
 
 
 
 
 
 3. K-means clustering of means 
 
 
 
  split <- paste0("Cluster\n", kclust10$cluster)
####split <- factor(paste0("Cluster\n", kclust10$cluster), levels=c("Cluster\n5","Cluster\n1","Cluster\n2","Cluster\n3","Cluster\n4","Cluster\n6"))
hmap_k <- Heatmap(dataHMm, split=split, #cluster_row_slices = FALSE,
                  cluster_columns = FALSE,
                  show_row_names = FALSE,
                  name = "Expression",
                  col = col_fun,
                  width = unit(20, "mm"),
                  column_title = "means", 
                  column_title_gp = gpar(fontsize = 16, fontface = "bold")
                  
                        )#top_annotation=colAnn)
hmap_hier_factors1 <- Heatmap(
  dataHi,  name = "ExpressionI",
  col = col_fun,
  column_title = paste0("individual samples"), 
  column_title_gp = gpar(fontsize = 16, fontface = "bold"),
  width = unit(60, "mm"),
  cluster_rows = FALSE,
  cluster_columns = FALSE,
  show_row_names = FALSE)
hmap_k  
 
 
   
 
 
 
 
 
 K-means clustering of means (with cluster annotation and individual samples added for inspection) 
 
 
 
  Response_Time<-data.frame(kclust10$cluster)
Response_Time = mutate(Response_Time, Response=
                   ifelse(Response_Time$kclust10.cluster==1, "transient", 
                          ifelse(Response_Time$kclust10.cluster==5, "transient",
                                 ifelse(Response_Time$kclust10.cluster==6, "transient",
                                        ifelse(Response_Time$kclust10.cluster==2, "transient",
                                               ifelse(Response_Time$kclust10.cluster==3, "transient",
                                                      ifelse(Response_Time$kclust10.cluster==4, "transient",
                                                                       "out")))))))
Response_Time<-Response_Time[c(2)]
rownames(Response_Time) <- NULL
ha = HeatmapAnnotation(df = Response_Time, which = "row", width = unit(1, "cm"),col = list(Response = c("early" =  "green3", "late" = "brown", "transient" = "violet")))
hmap_k+ha+hmap_hier_factors1  
 
 
   
 
 
 
 Mean profiles of clusters 
 
 
 
  clustercount<-data.frame(kclust10$cluster)
clustersizes<-table(clustercount$kclust10.cluster)
clusterMeans<-data.frame(kclust10$centers)
clusterMeans1<-data.frame(t(clusterMeans))
clusterMeans1 <- cbind(rownames(clusterMeans1), clusterMeans1)
orderN<-c("RBC_TNF_0h","RBC_TNF_2h","RBC_TNF_6h","RBC_TNF_20h")#### manual
rownames(clusterMeans1) <- NULL
names(clusterMeans1)[names(clusterMeans1)=="rownames(clusterMeans1)"] <- "Sample"
####clusterMeans1
pX1<-ggplot(data=clusterMeans1, aes(x=Sample, y=X1,group=1)) +
  geom_line()+  geom_point()+ggtitle(paste("Cluster X1 Profile ",clustersizes[1]," genes"))+  scale_x_discrete(limits=orderN)+
  theme(axis.title.x = element_blank(),axis.title.y = element_blank())
pX2<-ggplot(data=clusterMeans1, aes(x=Sample, y=X2,group=1)) +
  geom_line()+  geom_point()+ggtitle(paste("Cluster X2 Profile ",clustersizes[2]," genes"))+  scale_x_discrete(limits=orderN)+
  theme(axis.title.x = element_blank(),axis.title.y = element_blank())
pX3<-ggplot(data=clusterMeans1, aes(x=Sample, y=X3,group=1)) +
  geom_line()+  geom_point()+ggtitle(paste("Cluster X3 Profile ",clustersizes[3]," genes"))+  scale_x_discrete(limits=orderN)+
  theme(axis.title.x = element_blank(),axis.title.y = element_blank())
pX4<-ggplot(data=clusterMeans1, aes(x=Sample, y=X4,group=1)) +
  geom_line()+  geom_point()+ggtitle(paste("Cluster X4 Profile ",clustersizes[4]," genes"))+  scale_x_discrete(limits=orderN)+
  theme(axis.title.x = element_blank(),axis.title.y = element_blank())
pX5<-ggplot(data=clusterMeans1, aes(x=Sample, y=X5,group=1)) +
  geom_line()+  geom_point()+ggtitle(paste("Cluster X5 Profile ",clustersizes[5]," genes"))+  scale_x_discrete(limits=orderN)+
  theme(axis.title.x = element_blank(),axis.title.y = element_blank())
pX6<-ggplot(data=clusterMeans1, aes(x=Sample, y=X6,group=1)) +
  geom_line()+  geom_point()+ggtitle(paste("Cluster X6 Profile ",clustersizes[6]," genes"))+  scale_x_discrete(limits=orderN)+
  theme(axis.title.x = element_blank(),axis.title.y = element_blank())
#plot
multiplot(pX1, pX2, pX3, pX4,pX5, pX6, cols=2)  
 
 
   
 
 
 
 
 
 K-means clustering of means (other treatment means added for inspection) 
 
 
 
  hmap_k <- Heatmap(dataHMm, split=split, cluster_row_slices = FALSE,
                  cluster_columns = FALSE,
                  show_row_names = FALSE,
                  name = "Expression",
                  col = col_fun,
                  width = unit(25, "mm"),
                  column_title = "RBCTNF", 
                  column_title_gp = gpar(fontsize = 10, fontface = "bold"))
                  
                  
baseMeansHmTemp <-countsTable[,c(48:50)]
colnames(baseMeansHmTemp)<-c("Var37TNF_0h","Var37TNF_6h","Var37TNF_20h")
dataHMmR1_37Y<-baseMeansHmTemp[ topDEgenes, ]
dataHMmR1_37Y <- log2(dataHMmR1_37Y+1)
dataHMmR1_37Y<- t(as.matrix(dataHMmR1_37Y))
dataHMmR1_37Y <- t(scale(dataHMmR1_37Y))
baseMeansHmTemp <-countsTable[,c(60:63)]
colnames(baseMeansHmTemp)<-c("Var14_0h","Var14_2h","Var14_6h","Var14_20h")
dataHMmR2_14<-baseMeansHmTemp[ topDEgenes, ]
dataHMmR2_14 <- log2(dataHMmR2_14+1)
dataHMmR2_14<- t(as.matrix(dataHMmR2_14))
dataHMmR2_14 <- t(scale(dataHMmR2_14))
baseMeansHmTemp <-countsTable[,c(79:82)]
colnames(baseMeansHmTemp)<-c("RBC_0h","RBC_2h","RBC_6h","RBC_20h")
dataHMmR2_R<-baseMeansHmTemp[ topDEgenes, ]
dataHMmR2_R <- log2(dataHMmR2_R+1)
dataHMmR2_R<- t(as.matrix(dataHMmR2_R))
dataHMmR2_R <- t(scale(dataHMmR2_R))
baseMeansHmTemp <-countsTable[,c(110:113)]
colnames(baseMeansHmTemp)<-c("Var14TNF_0h","Var14TNF_2h","Var14TNF_6h","Var14TNF_20h")
dataHMmR4_14T<-baseMeansHmTemp[ topDEgenes, ]
dataHMmR4_14T <- log2(dataHMmR4_14T+1)
dataHMmR4_14T<- t(as.matrix(dataHMmR4_14T))
dataHMmR4_14T <- t(scale(dataHMmR4_14T))
baseMeansHmTemp <-countsTable[,c(129:132)]
colnames(baseMeansHmTemp)<-c("RBC_TNF_0h","RBC_TNF_2h","RBC_TNF_6h","RBC_TNF_20h")
dataHMmR4_RT<-baseMeansHmTemp[ topDEgenes, ]
dataHMmR4_RT <- log2(dataHMmR4_RT+1)
dataHMmR4_RT<- t(as.matrix(dataHMmR4_RT))
dataHMmR4_RT <- t(scale(dataHMmR4_RT))
hmap_37T <- Heatmap(dataHMmR1_37Y, split=split, 
                  name = "Expression37T",  
                  column_title = "VAR37TNF", 
                  cluster_columns = FALSE,  show_row_names = FALSE,col = col_fun,width = unit(20, "mm"),
                  column_title_gp = gpar(fontsize = 10, fontface = "bold"))
hmap_k14 <- Heatmap(dataHMmR2_14, split=split, 
                  name = "Expression14",  
                  column_title = "VAR14noTNF", 
                  cluster_columns = FALSE,  show_row_names = FALSE,col = col_fun,width = unit(25, "mm"),
                  column_title_gp = gpar(fontsize = 10, fontface = "bold"))
hmap_R <- Heatmap(dataHMmR2_R, split=split, 
                  name = "ExpressionR",  
                  column_title = "RBCnoTNF", 
                  cluster_columns = FALSE,  show_row_names = FALSE,col = col_fun,width = unit(25, "mm"),
                  column_title_gp = gpar(fontsize = 10, fontface = "bold"))
hmap_k14T <- Heatmap(dataHMmR4_14T, split=split, 
                  name = "Expression14T",  
                  column_title = "VAR14TNF", 
                  cluster_columns = FALSE,  show_row_names = FALSE,col = col_fun,width = unit(25, "mm"),
                  column_title_gp = gpar(fontsize = 10, fontface = "bold"))
hmap_RT <- Heatmap(dataHMmR4_RT, split=split, 
                  name = "ExpressionRT",  
                  column_title = "RBCTNF", 
                  cluster_columns = FALSE,  show_row_names = FALSE,col = col_fun,width = unit(25, "mm"),
                  column_title_gp = gpar(fontsize = 10, fontface = "bold"))
hmap_k+ha+hmap_k14T+hmap_37T+hmap_k14+hmap_R  
 
 
   
 
 
 
 
 
 
  topDEgenes <- which(tempA$Include=="in")####find indexes
tempAkm<-tempA[ topDEgenes, ]
SymbolsKm<-dplyr::pull(tempAkm, Gene_Symbol)
#### export the gene expression data for the clusters
write.table(clusterMeans,paste0("ClusterMeansKm_",groupsName,".txt"),  sep = "\t")
ClusteredGenes<-data.frame(kclust10$cluster,SymbolsKm,dataHMm)
write.table(ClusteredGenes,paste0("ScaledDataInClustersKm_",groupsName,".txt"),  sep = "\t")
#head(ClusteredGenes)  
 
 
 
 
 
 
  bottomDEgenes<-which(tempA$Include=="out")####find indexes 
bottomG<-tempA[ bottomDEgenes, ]
bottomG<-dplyr::pull(bottomG, Gene_Symbol)
write.table(bottomG,paste0("ipaBottomKmeans_",groupsName,".txt"),  sep = "\t")
                         
topDEgenes <- which(tempA$Include=="in")####find indexes 
tempAkm<-tempA[ topDEgenes, ]
SymbolsKm<-dplyr::pull(tempAkm, Gene_Symbol)
ipaKmeans<-ClusteredGenes
#countsTable <-countsTable[,c(1:15)]####if samples need removing
ipaKmeans<-ipaKmeans[,c(1:2)]
ipaKmeans$name2<-rownames(ipaKmeans)
#ipaKmeans%>% rownames_to_column(var = "rowname")
#ipaKmeans
#rowid_to_column(ipaKmeans)
ipaKmeans = mutate(ipaKmeans, x1= ifelse(ipaKmeans$kclust10.cluster==1, "1", "0"))
ipaKmeans = mutate(ipaKmeans, x2= ifelse(ipaKmeans$kclust10.cluster==2, "1", "0"))
ipaKmeans = mutate(ipaKmeans, x3= ifelse(ipaKmeans$kclust10.cluster==3, "1", "0"))
ipaKmeans = mutate(ipaKmeans, x4= ifelse(ipaKmeans$kclust10.cluster==4, "1", "0"))
ipaKmeans = mutate(ipaKmeans, x5= ifelse(ipaKmeans$kclust10.cluster==5, "1", "0"))
ipaKmeans = mutate(ipaKmeans, x6= ifelse(ipaKmeans$kclust10.cluster==6, "1", "0"))
#ipaKmeans
write.table(ipaKmeans,paste0("ipaKmeans_",groupsName,".txt"),  sep = "\t")
#head(ipaKmeans)  
 
 
 
 
 
 
  ClusteredGenes2<-ClusteredGenes[c(1)]
#ClusteredGenes2
listAll<-list()
for(i in 1:6) {
  clusterName<-paste0("x",i)
  #clusterName<-row.names(subset(ClusteredGenes,ClusteredGenes==i))
  clusterName<-(subset(ClusteredGenes$SymbolsKm,ClusteredGenes==i))
  listAll[[i]]<-clusterName
}
#need to name the vectors in the list, example here is for 8 clusters
names(listAll)<-c("X1", "X2", "X3", "X4","X5", "X6")
#if you want to rearrange the order
#listAll<-listAll[c("x3", "x7", "x8", "x2", "x6", "x5", "x4", "x1")]
#lapply(listAll, head)  
 
 
 
 
 
 4. Annotation of K-means clusters 
 
 CC cellular compartment 
 BP biological process 
 MF molecular function 
 
 The simplify function has been used to cut down on GO redundancy 
 
 
 
  #str(AllGeneNames)  
 
 
 
 
 
 
  ####CC
cgoCC <- compareCluster(geneCluster = listAll, 
                      universe = AllGeneNames,
                      fun = "enrichGO",
                      OrgDb=org.Hs.eg.db, 
                      ####OrgDb=org.Mm.eg.db,
                      keyType="SYMBOL",
                      ont = "CC", 
                      pvalueCutoff=0.05,
                      qvalueCutoff = 0.10)
cgoCC2 <- simplify(cgoCC, cutoff=0.7, by="p.adjust", select_fun=min)
####write as spreadsheet
write.csv(as.data.frame(cgoCC2),paste0("GO_CC_",groupsName,".csv"))
dotplot(cgoCC2,showCategory = 30,
        title = paste0("GO Cellular Compartment ",groupsName))+
  theme(axis.text.x = element_text(angle = 90, vjust = 0.5, hjust=1))  
 
 
   
 
 
 
 Plots and GO data were written to files 
 
 
 
  png(paste0("GO_CC_",groupsName,".png"), width = 1224, height = 824)
dotplot(cgoCC2,showCategory = 30,
        title = paste0("GO Cellular Compartment ",groupsName))+
  theme(axis.text.x = element_text(angle = 90, vjust = 0.5, hjust=1))
dev.off()  
 
 
  null device 
          1   
 
 
 
 GO BP 
 
 
 
  ####CC
cgoBP <- compareCluster(geneCluster = listAll, 
                      universe = AllGeneNames,
                      fun = "enrichGO",
                      OrgDb=org.Hs.eg.db,
                      keyType="SYMBOL",
                      ont = "BP", 
                      pvalueCutoff=0.05,
                      qvalueCutoff = 0.10)
cgoBP2 <- simplify(cgoBP, cutoff=0.7, by="p.adjust", select_fun=min)
####write as spreadsheet
write.csv(as.data.frame(cgoBP2),paste0("GO_BP_",groupsName,".csv"))
dotplot(cgoBP2,showCategory = 30,
        title = paste0("GO Biological Process ",groupsName))+
  theme(axis.text.x = element_text(angle = 90, vjust = 0.5, hjust=1))  
 
 
   
 
 
 
 
 
 
  png(paste0("GO_BP_",groupsName,".png"), width = 1024, height = 1224)
dotplot(cgoBP2,showCategory = 30,
        title = paste0("GO Biological Process ",groupsName))+
  theme(axis.text.x = element_text(angle = 90, vjust = 0.5, hjust=1))
dev.off()  
 
 
  null device 
          1   
 
 
 
 GO MF 
 
 
 
  ####MF
cgoMF <- compareCluster(geneCluster = listAll, 
                      universe = AllGeneNames,
                      fun = "enrichGO",
                      OrgDb=org.Hs.eg.db, 
                      keyType="SYMBOL",
                      ont = "MF", 
                      pvalueCutoff=0.05,
                      qvalueCutoff = 0.10)
cgoMF2 <- simplify(cgoMF, cutoff=0.7, by="p.adjust", select_fun=min)
####write as spreadsheet
write.csv(as.data.frame(cgoMF2),paste0("GO_MF_",groupsName,".csv"))
dotplot(cgoMF2,showCategory = 30,
        title = paste0("GO Molecular Function  ",groupsName))+
  theme(axis.text.x = element_text(angle = 90, vjust = 0.5, hjust=1))  
 
 
   
 
 
 
 
 
 
  png(paste0("GO_MF_",groupsName,".png"), width = 1424, height = 824)
dotplot(cgoMF2,showCategory = 30,
        title = paste0("GO Molecular Function  ",groupsName))+
  theme(axis.text.x = element_text(angle = 90, vjust = 0.5, hjust=1))
dev.off()  
 
 
  null device 
          1   
 
 
 
 save: once happy with clustering save workspace so that it can be recalled 
 
 
 
  save.image(file="Km.RData")  
 
 
 
 Add a new chunk by clicking the  Insert Chunk  button on the toolbar or by pressing  Ctrl+Alt+I . 
 When you save the notebook, an HTML file containing the code and output will be saved alongside it (click the  Preview  button or press  Ctrl+Shift+K  to preview the HTML file). 
 The preview shows you a rendered HTML copy of the contents of the editor. Consequently, unlike  Knit ,  Preview  does not run any R code chunks. Instead, the output of the chunk when it was last run in the editor is displayed.
[truncated: 269,101 more chars]
