# Supplementary material for: Different PfEMP1-expressing Plasmodium falciparum variants induce divergent endothelial transcriptional responses during co-culture
Source: PLoS One. 2023 Nov 30;18(11):e0295053. doi: 10.1371/journal.pone.0295053 (PMC10688957; doi:10.1371/journal.pone.0295053)
Supplement: S4 File — (HTML) [file pone.0295053.s013.html]

KCouper/Liverpool K-means RNAseq Analysis November 2020


Code 

- Show All Code
- Hide All Code
- Download Rmd

# KCouper/Liverpool K-means RNAseq Analysis November 2020

#### Leo Zeef

## Analysis Sections

Viewing is better if Code is hidden (Top Right drop down list)


```
sink(file="RsessionInfoDESeq2.txt")
library('DESeq2')
library("ggplot2")
library(reshape2)
####library(tidyverse)
####library(splitstackshape)
####library(data.table)
library("RColorBrewer")
library("gplots")
####library('ggdendro')
library('ggrepel')
library("dplyr")
library("ComplexHeatmap")
library("clusterProfiler")
library(VennDiagram) ######
library(UpSetR)
library(gridExtra)
library(cluster)
library(circlize)
library(factoextra)
library(NbClust)
library("biomaRt")
library("org.Hs.eg.db")####human
library("org.Mm.eg.db")####mouse
library(venn)
####library(org.At.tair.db)####arabidopsis
sessionInfo()
sink()
#########################################
####multiplot
#########################################
#### Multiple plot function
####
#### ggplot objects can be passed in ..., or to plotlist (as a list of ggplot objects)
#### - cols:   Number of columns in layout
#### - layout: A matrix specifying the layout. If present, 'cols' is ignored.
#### If the layout is something like matrix(c(1,2,3,3), nrow=2, byrow=TRUE),
#### then plot 1 will go in the upper left, 2 will go in the upper right, and
#### 3 will go all the way across the bottom.
multiplot <- function(..., plotlist=NULL, file, cols=1, layout=NULL) {
  library(grid)
  
  #### Make a list from the ... arguments and plotlist
  plots <- c(list(...), plotlist)
  
  numPlots = length(plots)
  
  #### If layout is NULL, then use 'cols' to determine layout
  if (is.null(layout)) {
    #### Make the panel
    #### ncol: Number of columns of plots
    #### nrow: Number of rows needed, calculated from #### of cols
    layout <- matrix(seq(1, cols * ceiling(numPlots/cols)),
                     ncol = cols, nrow = ceiling(numPlots/cols))
  }
  
  if (numPlots==1) {
    print(plots[[1]])
    
  } else {
    #### Set up the page
    grid.newpage()
    pushViewport(viewport(layout = grid.layout(nrow(layout), ncol(layout))))
    
    #### Make each plot, in the correct location
    for (i in 1:numPlots) {
      #### Get the i,j matrix positions of the regions that contain this subplot
      matchidx <- as.data.frame(which(layout == i, arr.ind = TRUE))
      
      print(plots[[i]], vp = viewport(layout.pos.row = matchidx$row,
                                      layout.pos.col = matchidx$col))
    }
  }
}
####function my code edit of plotPCA
####################################
plotPCALeo<-function (x, intgroup = "Treatment", ntop = 500, returnData = FALSE, PCx=1, PCy=2)
{
  ####rv <- rowVars(assay(x))
  rv = apply((assay(x)), 1, var)
  select <- order(rv, decreasing = TRUE)[seq_len(min(ntop, 
                                                     length(rv)))]
  pca <- prcomp(t(assay(x)[select, ]))
  percentVar <- pca$sdev^2/sum(pca$sdev^2)
  if (!all(intgroup %in% names(colData(x)))) {
    stop("the argument 'intgroup' should specify columns of colData(dds)")
  }
  intgroup.df <- as.data.frame(colData(x)[, intgroup, drop = FALSE])
  group <- factor(apply(intgroup.df, 1, paste, collapse = " : "))
  d <- data.frame(PCX = pca$x[, PCx], PCY = pca$x[, PCy], group = group, 
                  intgroup.df, names = colnames(x))
  if (returnData) {
    attr(d, "percentVar") <- percentVar[PCx:PCy]
    return(d)
  }
  ggplot(data = d, aes_string(x = "PCX", y = "PCY", color = "group")) + 
    ####ggplot(data = d, aes_string(x = "PCX", y = "PCY", color=Tgfb1, shape=Treatment)) + 
    geom_point(size = 3) + xlab(paste0("PC",PCx,": ", round(percentVar[1] * 
                                                              100), "% variance")) + ylab(paste0("PC",PCy,": ", round(percentVar[2] * 
                                                                                                                        100), "% variance"))
}
```


```
col_fun = colorRamp2(c(-1,-0.2, 0,0.2, 1), c("blue","cyan", "grey90","orange", "red"))#heatmap colours
col_funGR = colorRamp2(c(-1.5, 0, 1.5), c("green", "black", "red"))
col_funGR2 = colorRamp2(c(-2, 0, 2), c("green", "black", "red"))
colorsV3 <- c("cornflowerblue",  "brown1","orange2")#Venn colours
colorsV2 <- c("mediumorchid1",  "chartreuse3")#Venn colours
colorsV4<-c("cornflowerblue", "orange2", "green3","red")#Venn colours
colorsV5<-c("cornflowerblue", "orange2", "green3","purple","red")#Venn colours
#col_fun(seq(-3, 3))
```


### R3 VAR14 vs RBC no TNF k-means q0.05

#### 1. Genelist Selection


```
groupsName<-"R3_VAR14_kmeans_q0.05"
```


```
countsTable<-read.delim("RNAseq2019July_5.txt", header = TRUE, sep = "\t",check.names=FALSE,row.names=1)
head(countsTable)
```


```
AllGeneNames<-countsTable$Gene_Symbol
#head(AllGeneNames)
```


```
tempA<-countsTable
```


```
topDEgenes <- which(tempA$padj_R3noTNF_var14_vs_RBC_0h<0.05&!is.na(tempA$padj_R3noTNF_var14_vs_RBC_0h))####find indexes 
listA<-tempA[ topDEgenes, ]$Gene_Symbol
topDEgenes <- which(tempA$padj_R3noTNF_var14_vs_RBC_2h<0.05&!is.na(tempA$padj_R3noTNF_var14_vs_RBC_2h))####find indexes 
listB<-tempA[ topDEgenes, ]$Gene_Symbol
topDEgenes <- which(tempA$padj_R3noTNF_var14_vs_RBC_6h<0.05&!is.na(tempA$padj_R3noTNF_var14_vs_RBC_6h))####find indexes 
listC<-tempA[ topDEgenes, ]$Gene_Symbol
topDEgenes <- which(tempA$padj_R3noTNF_var14_vs_RBC_20h<0.05&!is.na(tempA$padj_R3noTNF_var14_vs_RBC_20h))####find indexes 
listD<-tempA[ topDEgenes, ]$Gene_Symbol
vennq<-venn.diagram(x = list(listA,listB,listC,listD) ,
            category.names = c("Var14noTNF_0h","Var14noTNF_2h","Var14noTNF_6h","Var14noTNF_20h"),
            main="padj<0.05",
            filename = NULL,  scaled = FALSE, fill = colorsV4, cat.col = colorsV4, cat.cex = 1, cat.dist=0.3,  margin = 0.3)
topDEgenes <- which(tempA$pvalue_R3noTNF_var14_vs_RBC_0h<0.05&abs(tempA$log2FoldChange_R3noTNF_var14_vs_RBC_0h)>1&!is.na(tempA$pvalue_R3noTNF_var14_vs_RBC_0h))####find indexes 
listA<-tempA[ topDEgenes, ]$Gene_Symbol
topDEgenes <- which(tempA$pvalue_R3noTNF_var14_vs_RBC_2h<0.05&abs(tempA$log2FoldChange_R3noTNF_var14_vs_RBC_2h)>1&!is.na(tempA$pvalue_R3noTNF_var14_vs_RBC_2h))####find indexes 
listB<-tempA[ topDEgenes, ]$Gene_Symbol
topDEgenes <- which(tempA$pvalue_R3noTNF_var14_vs_RBC_6h<0.05&abs(tempA$log2FoldChange_R3noTNF_var14_vs_RBC_6h)>1&!is.na(tempA$pvalue_R3noTNF_var14_vs_RBC_6h))####find indexes 
listC<-tempA[ topDEgenes, ]$Gene_Symbol
topDEgenes <- which(tempA$pvalue_R3noTNF_var14_vs_RBC_20h<0.05&abs(tempA$log2FoldChange_R3noTNF_var14_vs_RBC_20h)>1&!is.na(tempA$pvalue_R3noTNF_var14_vs_RBC_20h))####find indexes 
listD<-tempA[ topDEgenes, ]$Gene_Symbol
vennp<-venn.diagram(x = list(listA,listB,listC,listD) ,
            category.names = c("Var14noTNF_0h","Var14noTNF_2h","Var14noTNF_6h","Var14noTNF_20h"),
            main="pvalue<0.05&fold change>2",
            filename = NULL,  scaled = FALSE, fill = colorsV4, cat.col = colorsV4, cat.cex = 1, cat.dist=0.3,  margin = 0.3)
topDEgenes <- which(tempA$padj_R3noTNF_var14_vs_RBC_0h<0.1&abs(tempA$log2FoldChange_R3noTNF_var14_vs_RBC_0h)>1&!is.na(tempA$padj_R3noTNF_var14_vs_RBC_0h))####find indexes 
listA<-tempA[ topDEgenes, ]$Gene_Symbol
topDEgenes <- which(tempA$padj_R3noTNF_var14_vs_RBC_2h<0.1&abs(tempA$log2FoldChange_R3noTNF_var14_vs_RBC_2h)>1&!is.na(tempA$padj_R3noTNF_var14_vs_RBC_2h))####find indexes 
listB<-tempA[ topDEgenes, ]$Gene_Symbol
topDEgenes <- which(tempA$padj_R3noTNF_var14_vs_RBC_6h<0.1&abs(tempA$log2FoldChange_R3noTNF_var14_vs_RBC_6h)>1&!is.na(tempA$padj_R3noTNF_var14_vs_RBC_6h))####find indexes 
listC<-tempA[ topDEgenes, ]$Gene_Symbol
topDEgenes <- which(tempA$padj_R3noTNF_var14_vs_RBC_20h<0.1&abs(tempA$log2FoldChange_R3noTNF_var14_vs_RBC_20h)>1&!is.na(tempA$padj_R3noTNF_var14_vs_RBC_20h))####find indexes 
listD<-tempA[ topDEgenes, ]$Gene_Symbol
vennq2<-venn.diagram(x = list(listA,listB,listC,listD) ,
            category.names = c("Var14noTNF_0h","Var14noTNF_2h","Var14noTNF_6h","Var14noTNF_20h"),
            main="padj0.1&fold change>2",
            filename = NULL,  scaled = FALSE, fill = colorsV4, cat.col = colorsV4, cat.cex = 1, cat.dist=0.3,  margin = 0.3)
```


```
topDEgenes <- which((tempA$padj_R3noTNF_var14_vs_RBC_0h<0.05&!is.na(tempA$padj_R3noTNF_var14_vs_RBC_0h))| 
(tempA$padj_R3noTNF_var14_vs_RBC_2h<0.05&!is.na(tempA$padj_R3noTNF_var14_vs_RBC_2h))|
(tempA$padj_R3noTNF_var14_vs_RBC_6h<0.05&!is.na(tempA$padj_R3noTNF_var14_vs_RBC_6h))| 
(tempA$padj_R3noTNF_var14_vs_RBC_20h<0.05&!is.na(tempA$padj_R3noTNF_var14_vs_RBC_20h)) 
)
listA<-tempA[ topDEgenes, ]$Gene_Symbol
topDEgenes <- which((tempA$pvalue_R3noTNF_var14_vs_RBC_0h<0.05&abs(tempA$log2FoldChange_R3noTNF_var14_vs_RBC_0h)>1&!is.na(tempA$pvalue_R3noTNF_var14_vs_RBC_0h))| 
(tempA$pvalue_R3noTNF_var14_vs_RBC_2h<0.05&abs(tempA$log2FoldChange_R3noTNF_var14_vs_RBC_2h)>1&!is.na(tempA$pvalue_R3noTNF_var14_vs_RBC_2h))| 
(tempA$pvalue_R3noTNF_var14_vs_RBC_6h<0.05&abs(tempA$log2FoldChange_R3noTNF_var14_vs_RBC_6h)>1&!is.na(tempA$pvalue_R3noTNF_var14_vs_RBC_6h))| 
(tempA$pvalue_R3noTNF_var14_vs_RBC_20h<0.05&abs(tempA$log2FoldChange_R3noTNF_var14_vs_RBC_20h)>1&!is.na(tempA$pvalue_R3noTNF_var14_vs_RBC_20h))
 )####find indexes 
listC<-tempA[ topDEgenes, ]$Gene_Symbol
topDEgenes <- which((tempA$padj_R3noTNF_var14_vs_RBC_0h<0.1&abs(tempA$log2FoldChange_R3noTNF_var14_vs_RBC_0h)>1&!is.na(tempA$padj_R3noTNF_var14_vs_RBC_0h))| 
(tempA$padj_R3noTNF_var14_vs_RBC_2h<0.1&abs(tempA$log2FoldChange_R3noTNF_var14_vs_RBC_2h)>1&!is.na(tempA$padj_R3noTNF_var14_vs_RBC_2h))| 
(tempA$padj_R3noTNF_var14_vs_RBC_6h<0.1&abs(tempA$log2FoldChange_R3noTNF_var14_vs_RBC_6h)>1&!is.na(tempA$padj_R3noTNF_var14_vs_RBC_6h))| 
(tempA$padj_R3noTNF_var14_vs_RBC_20h<0.1&abs(tempA$log2FoldChange_R3noTNF_var14_vs_RBC_20h)>1&!is.na(tempA$padj_R3noTNF_var14_vs_RBC_20h))
 )####find indexes 
listB<-tempA[ topDEgenes, ]$Gene_Symbol
vennpq<-venn.diagram(x = list(listA,listB,listC) ,
            category.names = c("padj<0.05","padj<0.1&fc>2","p<0.05&fc>2"),
            main="padj compared to pvalue",
            filename = NULL,  scaled = FALSE, fill = colorsV3, cat.col = colorsV3, cat.cex = 1, cat.dist=0.1,  margin = 0.15)
```


```
grid.arrange(gTree(children=vennq), gTree(children=vennpq), ncol=2,top="R2 Var14 no TNF")
```


```
#tempA<-resAll[-c(10:30) ]
tempA<-countsTable
#rownames(tempA)
rownames(tempA) <- NULL
tempA = mutate(tempA, Include=
                   ifelse(tempA$padj_R3noTNF_var14_vs_RBC_0h<0.05&!is.na(tempA$padj_R3noTNF_var14_vs_RBC_0h), "in",
                          ifelse(tempA$padj_R3noTNF_var14_vs_RBC_2h<0.05&!is.na(tempA$padj_R3noTNF_var14_vs_RBC_2h), "in",
                                 ifelse(tempA$padj_R3noTNF_var14_vs_RBC_6h<0.05&!is.na(tempA$padj_R3noTNF_var14_vs_RBC_6h), "in",
                                        ifelse(tempA$padj_R3noTNF_var14_vs_RBC_20h<0.05&!is.na(tempA$padj_R3noTNF_var14_vs_RBC_20h), "in",
                                                 "out")))))
#tempA
####library(dplyr)
tempA %>%
     group_by(Include) %>% 
     tally()
```


```
topDEgenes <- which(tempA$Include=="in")####find indexes
```


```
head(countsTable)
```

#### NB Please check columns used and renamed for plots


```
#baseMeansHm <-countsTable[,c(60:63)]
baseMeansHm <-countsTable[,c(60:63,79:82)]
head(baseMeansHm)
```


```
baseMeansHm2 <- log2(baseMeansHm+1)
baseMeansHm2$Var14_RBC_0h<-baseMeansHm2$Var14noTNF_0h_mean-baseMeansHm2$RBCnoTNF_0h_mean
baseMeansHm2$Var14_RBC_2h<-baseMeansHm2$Var14noTNF_2h_mean-baseMeansHm2$RBCnoTNF_2h_mean
baseMeansHm2$Var14_RBC_6h<-baseMeansHm2$Var14noTNF_6h_mean-baseMeansHm2$RBCnoTNF_6h_mean
baseMeansHm2$Var14_RBC_20h<-baseMeansHm2$Var14noTNF_20h_mean-baseMeansHm2$RBCnoTNF_20h_mean
baseMeansHm <-baseMeansHm2[,c(9:12)]
head(baseMeansHm)
```


```
baseMeansHmM <-baseMeansHm2[,c(1:8)]
head(baseMeansHmM)
```


```
topDEgenes <- which(tempA$Include=="in")####find indexes
```

#### 2. Hierachical clustering of means (individual samples added for inspection)


```
####mean logfc
dataHMm<-baseMeansHm[ topDEgenes, ]
#dataHMm <- log2(dataHMm+1)
dataHMm<- t(as.matrix(dataHMm))
dataHMm <- t(scale(dataHMm))
hmap_hier_factors4 <- Heatmap(
  dataHMm,  name = "logfc",
  row_labels = paste0(rownames(dataHMm)," ",(tempA[ topDEgenes, ])$Gene_Symbol),
  column_title = paste0("Means logfc"), 
  col = col_funGR,
  column_title_gp = gpar(fontsize = 16, fontface = "bold"),
  width = unit(50, "mm"),
  cluster_columns = FALSE,
  show_row_names = FALSE)
dataHMmPlot<-as.data.frame(dataHMm)
dataHMmPlot$Var14_RBC_2h<-dataHMmPlot$Var14_RBC_2h-dataHMmPlot$Var14_RBC_0h
dataHMmPlot$Var14_RBC_6h<-dataHMmPlot$Var14_RBC_6h-dataHMmPlot$Var14_RBC_0h
dataHMmPlot$Var14_RBC_20h<-dataHMmPlot$Var14_RBC_20h-dataHMmPlot$Var14_RBC_0h
dataHMmPlot$Var14_RBC_0h<-dataHMmPlot$Var14_RBC_0h-dataHMmPlot$Var14_RBC_0h
dataHMmPlot<-as.matrix(dataHMmPlot)
  
hmap_hier_factors6 <- Heatmap(
  dataHMmPlot,  name = "Normalised logfc",
  row_labels = paste0(rownames(dataHMm)," ",(tempA[ topDEgenes, ])$Gene_Symbol),
  column_title = paste0("0h Normalised logfc"), 
  col = col_funGR2,
  column_title_gp = gpar(fontsize = 16, fontface = "bold"),
  width = unit(50, "mm"),
  cluster_columns = FALSE,
  show_row_names = FALSE)
####means
dataHMm3<-baseMeansHmM[ topDEgenes, ]
#dataHMm <- log2(dataHMm+1)
dataHMm3<- t(as.matrix(dataHMm3))
dataHMm3 <- t(scale(dataHMm3))
dataHMm3<-dataHMm3[,c(5,1,6,2,7,3,8,4)]
hmap_hier_factors5 <- Heatmap(
  dataHMm3,  name = "Expression",
  row_labels = paste0(rownames(dataHMm)," ",(tempA[ topDEgenes, ])$Gene_Symbol),
  column_title = paste0("Means"), 
  col = col_fun,
  column_title_gp = gpar(fontsize = 16, fontface = "bold"),
  width = unit(100, "mm"),
  cluster_columns = FALSE,
  show_row_names = FALSE)
  
hmap_hier_factors4+hmap_hier_factors6+hmap_hier_factors5
```


```
par(mfrow=c(1,2))
#### Silhouette method
fviz_nbclust(dataHMm, kmeans, method = "silhouette",k.max = 16)+
  labs(subtitle = "Silhouette method")
```


```
#### Elbow method
fviz_nbclust(dataHMm, kmeans, method = "wss",k.max = 16) +
  labs(subtitle = "Elbow method")
```


```
####gap stat slow!!!
####set.seed(123)
####fviz_nbclust(dataHMm, kmeans, nstart = 25,  method = "gap_stat", nboot = 100,k.max = 16)+
####  labs(subtitle = "Gap statistic method")
```


```
kclust3 <- kmeans(dataHMm, 7)
#silhouette plot
distK<-daisy(dataHMm)
plot(silhouette(kclust3$cluster, distK), col=1:7, border=NA)
```

#### 3. K-means clustering of means


```
split <- paste0("Cluster\n", kclust3$cluster)
#split <- factor(paste0("Cluster\n", kclust3$cluster), levels=c("Cluster\n3","Cluster\n1","Cluster\n4","Cluster\n5","Cluster\n2","Cluster\n6"))
hmap_k <- Heatmap(dataHMm, split=split, cluster_row_slices = FALSE,
                  cluster_columns = FALSE,
                  show_row_names = FALSE,
                  name = "logfc",
                  col = col_funGR,
                  width = unit(50, "mm"),
                  column_title = "means logfc", 
                  column_title_gp = gpar(fontsize = 16, fontface = "bold"))
hmap_k+hmap_hier_factors6+hmap_hier_factors5
```


Mean profiles of clusters


```
clustercount<-data.frame(kclust3$cluster)
clustersizes<-table(clustercount$kclust3.cluster)
clusterMeans<-data.frame(kclust3$centers)
clusterMeans1<-data.frame(t(clusterMeans))
clusterMeans1 <- cbind(rownames(clusterMeans1), clusterMeans1)
orderN<-c("Var14_RBC_0h","Var14_RBC_2h","Var14_RBC_6h","Var14_RBC_20h")#### manual
rownames(clusterMeans1) <- NULL
names(clusterMeans1)[names(clusterMeans1)=="rownames(clusterMeans1)"] <- "Sample"
####clusterMeans1
pX1<-ggplot(data=clusterMeans1, aes(x=Sample, y=X1,group=1)) +
  geom_line()+  geom_point()+ggtitle(paste("Cluster X1 Profile ",clustersizes[1]," genes"))+  scale_x_discrete(limits=orderN)+
  theme(axis.title.x = element_blank(),axis.title.y = element_blank())
pX2<-ggplot(data=clusterMeans1, aes(x=Sample, y=X2,group=1)) +
  geom_line()+  geom_point()+ggtitle(paste("Cluster X2 Profile ",clustersizes[2]," genes"))+  scale_x_discrete(limits=orderN)+
  theme(axis.title.x = element_blank(),axis.title.y = element_blank())
pX3<-ggplot(data=clusterMeans1, aes(x=Sample, y=X3,group=1)) +
  geom_line()+  geom_point()+ggtitle(paste("Cluster X3 Profile ",clustersizes[3]," genes"))+  scale_x_discrete(limits=orderN)+
  theme(axis.title.x = element_blank(),axis.title.y = element_blank())
pX4<-ggplot(data=clusterMeans1, aes(x=Sample, y=X4,group=1)) +
  geom_line()+  geom_point()+ggtitle(paste("Cluster X4 Profile ",clustersizes[4]," genes"))+  scale_x_discrete(limits=orderN)+
  theme(axis.title.x = element_blank(),axis.title.y = element_blank())
pX5<-ggplot(data=clusterMeans1, aes(x=Sample, y=X5,group=1)) +
  geom_line()+  geom_point()+ggtitle(paste("Cluster X5 Profile ",clustersizes[5]," genes"))+  scale_x_discrete(limits=orderN)+
  theme(axis.title.x = element_blank(),axis.title.y = element_blank())
pX6<-ggplot(data=clusterMeans1, aes(x=Sample, y=X6,group=1)) +
  geom_line()+  geom_point()+ggtitle(paste("Cluster X6 Profile ",clustersizes[6]," genes"))+  scale_x_discrete(limits=orderN)+
  theme(axis.title.x = element_blank(),axis.title.y = element_blank())
pX7<-ggplot(data=clusterMeans1, aes(x=Sample, y=X7,group=1)) +
  geom_line()+  geom_point()+ggtitle(paste("Cluster X7 Profile ",clustersizes[5]," genes"))+  scale_x_discrete(limits=orderN)+
  theme(axis.title.x = element_blank(),axis.title.y = element_blank())
#pX8<-ggplot(data=clusterMeans1, aes(x=Sample, y=X8,group=1)) +
#  geom_line()+  geom_point()+ggtitle(paste("Cluster X8 Profile ",clustersizes[6]," genes"))+  scale_x_discrete(limits=orderN)+
#  theme(axis.title.x = element_blank(),axis.title.y = element_blank())
#plot
multiplot(pX1, pX2, pX3, pX4,pX5, pX6,pX7,  cols=2)
```


```
topDEgenes <- which(tempA$Include=="in")####find indexes
tempAkm<-tempA[ topDEgenes, ]
SymbolsKm<-dplyr::pull(tempAkm, Gene_Symbol)
#### export the gene expression data for the clusters
write.table(clusterMeans,paste0("ClusterMeansKm_",groupsName,".txt"),  sep = "\t")
ClusteredGenes<-data.frame(kclust3$cluster,SymbolsKm,dataHMm)
write.table(ClusteredGenes,paste0("ScaledDataInClustersKm_",groupsName,".txt"),  sep = "\t")
#head(ClusteredGenes)
```


```
bottomDEgenes<-which(tempA$Include=="out")####find indexes 
bottomG<-tempA[ bottomDEgenes, ]
bottomG<-dplyr::pull(bottomG, Gene_Symbol)
write.table(bottomG,paste0("ipaBottomKmeans_",groupsName,".txt"),  sep = "\t")
                         
topDEgenes <- which(tempA$Include=="in")####find indexes 
tempAkm<-tempA[ topDEgenes, ]
SymbolsKm<-dplyr::pull(tempAkm, Gene_Symbol)
ipaKmeans<-ClusteredGenes
#countsTable <-countsTable[,c(1:15)]####if samples need removing
ipaKmeans<-ipaKmeans[,c(1:2)]
ipaKmeans$name2<-rownames(ipaKmeans)
#ipaKmeans%>% rownames_to_column(var = "rowname")
#ipaKmeans
#rowid_to_column(ipaKmeans)
ipaKmeans = mutate(ipaKmeans, x1= ifelse(ipaKmeans$kclust3.cluster==1, "1", "0"))
ipaKmeans = mutate(ipaKmeans, x2= ifelse(ipaKmeans$kclust3.cluster==2, "1", "0"))
ipaKmeans = mutate(ipaKmeans, x3= ifelse(ipaKmeans$kclust3.cluster==3, "1", "0"))
ipaKmeans = mutate(ipaKmeans, x4= ifelse(ipaKmeans$kclust3.cluster==4, "1", "0"))
ipaKmeans = mutate(ipaKmeans, x5= ifelse(ipaKmeans$kclust3.cluster==5, "1", "0"))
ipaKmeans = mutate(ipaKmeans, x6= ifelse(ipaKmeans$kclust3.cluster==6, "1", "0"))
ipaKmeans = mutate(ipaKmeans, x7= ifelse(ipaKmeans$kclust3.cluster==7, "1", "0"))
#ipaKmeans
write.table(ipaKmeans,paste0("ipaKmeans_",groupsName,".txt"),  sep = "\t")
#head(ipaKmeans)
```


```
ClusteredGenes2<-ClusteredGenes[c(1)]
#ClusteredGenes2
listAll<-list()
for(i in 1:7) {
  clusterName<-paste0("x",i)
  #clusterName<-row.names(subset(ClusteredGenes,ClusteredGenes==i))
  clusterName<-(subset(ClusteredGenes$SymbolsKm,ClusteredGenes==i))
  listAll[[i]]<-clusterName
}
#need to name the vectors in the list, example here is for 8 clusters
names(listAll)<-c("X1", "X2", "X3", "X4","X5", "X6", "X7")
#if you want to rearrange the order
#listAll<-listAll[c("x3", "x7", "x8", "x2", "x6", "x5", "x4", "x1")]
#lapply(listAll, head)
```

#### 4. Annotation of K-means clusters

- CC cellular compartment
- BP biological process
- MF molecular function

The simplify function has been used to cut down on GO redundancy


```
#str(AllGeneNames)
```


```
####CC
cgoCC <- compareCluster(geneCluster = listAll, 
                      universe = AllGeneNames,
                      fun = "enrichGO",
                      OrgDb=org.Hs.eg.db, 
                      ####OrgDb=org.Mm.eg.db,
                      keyType="SYMBOL",
                      ont = "CC", 
                      pvalueCutoff=0.05,
                      qvalueCutoff = 0.10)
cgoCC2 <- simplify(cgoCC, cutoff=0.7, by="p.adjust", select_fun=min)
####write as spreadsheet
write.csv(as.data.frame(cgoCC2),paste0("GO_CC_",groupsName,".csv"))
dotplot(cgoCC2,showCategory = 30,
        title = paste0("GO Cellular Compartment ",groupsName))+
  theme(axis.text.x = element_text(angle = 90, vjust = 0.5, hjust=1))
```


Plots and GO data were written to files


```
png(paste0("GO_CC_",groupsName,".png"), width = 1224, height = 824)
dotplot(cgoCC2,showCategory = 30,
        title = paste0("GO Cellular Compartment ",groupsName))+
  theme(axis.text.x = element_text(angle = 90, vjust = 0.5, hjust=1))
dev.off()
```


```
null device 
          1
```


GO BP


```
####CC
cgoBP <- compareCluster(geneCluster = listAll, 
                      universe = AllGeneNames,
                      fun = "enrichGO",
                      OrgDb=org.Hs.eg.db,
                      keyType="SYMBOL",
                      ont = "BP", 
                      pvalueCutoff=0.05,
                      qvalueCutoff = 0.10)
cgoBP2 <- simplify(cgoBP, cutoff=0.7, by="p.adjust", select_fun=min)
####write as spreadsheet
write.csv(as.data.frame(cgoBP2),paste0("GO_BP_",groupsName,".csv"))
dotplot(cgoBP2,showCategory = 30,
        title = paste0("GO Biological Process ",groupsName))+
  theme(axis.text.x = element_text(angle = 90, vjust = 0.5, hjust=1))
```


```
png(paste0("GO_BP_",groupsName,".png"), width = 1024, height = 1224)
dotplot(cgoBP2,showCategory = 30,
        title = paste0("GO Biological Process ",groupsName))+
  theme(axis.text.x = element_text(angle = 90, vjust = 0.5, hjust=1))
dev.off()
```


```
null device 
          1
```


GO MF


```
####MF
cgoMF <- compareCluster(geneCluster = listAll, 
                      universe = AllGeneNames,
                      fun = "enrichGO",
                      OrgDb=org.Hs.eg.db, 
                      keyType="SYMBOL",
                      ont = "MF", 
                      pvalueCutoff=0.05,
                      qvalueCutoff = 0.10)
cgoMF2 <- simplify(cgoMF, cutoff=0.7, by="p.adjust", select_fun=min)
####write as spreadsheet
write.csv(as.data.frame(cgoMF2),paste0("GO_MF_",groupsName,".csv"))
dotplot(cgoMF2,showCategory = 30,
        title = paste0("GO Molecular Function  ",groupsName))+
  theme(axis.text.x = element_text(angle = 90, vjust = 0.5, hjust=1))
```


```
png(paste0("GO_MF_",groupsName,".png"), width = 1424, height = 824)
dotplot(cgoMF2,showCategory = 30,
        title = paste0("GO Molecular Function  ",groupsName))+
  theme(axis.text.x = element_text(angle = 90, vjust = 0.5, hjust=1))
dev.off()
```


```
null device 
          1
```

### R3 VAR14 vs RBC no TNF k-means p0.05fc2

#### 1. Genelist Selection


```
groupsName<-"R3_VAR14_kmeans_p0.05fc2"
```


```
countsTable<-read.delim("RNAseq2019July_5.txt", header = TRUE, sep = "\t",check.names=FALSE,row.names=1)
head(countsTable)
```


```
AllGeneNames<-countsTable$Gene_Symbol
#head(AllGeneNames)
```


```
grid.arrange(gTree(children=vennp), gTree(children=vennpq) , ncol=2,top="R3 Var14 no TNF")
```


```
#tempA<-resAll[-c(10:30) ]
tempA<-countsTable
#rownames(tempA)
rownames(tempA) <- NULL
tempA = mutate(tempA, Include=
                   ifelse(tempA$pvalue_R3noTNF_var14_vs_RBC_0h<0.05&abs(tempA$log2FoldChange_R3noTNF_var14_vs_RBC_0h)>1&!is.na(tempA$pvalue_R3noTNF_var14_vs_RBC_0h), "in",
                          ifelse(tempA$pvalue_R3noTNF_var14_vs_RBC_2h<0.05&abs(tempA$log2FoldChange_R3noTNF_var14_vs_RBC_2h)>1&!is.na(tempA$pvalue_R3noTNF_var14_vs_RBC_2h), "in",
                                 ifelse(tempA$pvalue_R3noTNF_var14_vs_RBC_6h<0.05&abs(tempA$log2FoldChange_R3noTNF_var14_vs_RBC_6h)>1&!is.na(tempA$pvalue_R3noTNF_var14_vs_RBC_6h), "in",
                                        ifelse(tempA$pvalue_R3noTNF_var14_vs_RBC_20h<0.05&abs(tempA$log2FoldChange_R3noTNF_var14_vs_RBC_20h)>1&!is.na(tempA$pvalue_R3noTNF_var14_vs_RBC_20h), "in",
                                                                       "out")))))
#tempA
####library(dplyr)
tempA %>%
     group_by(Include) %>% 
     tally()
```


```
topDEgenes <- which(tempA$Include=="in")####find indexes
```

#### NB Please check columns used and renamed for plots


```
baseMeansHm <-countsTable[,c(60:63,79:82)]
head(baseMeansHm)
```


```
baseMeansHm2 <- log2(baseMeansHm+1)
baseMeansHm2$Var14_RBC_0h<-baseMeansHm2$Var14noTNF_0h_mean-baseMeansHm2$RBCnoTNF_0h_mean
baseMeansHm2$Var14_RBC_2h<-baseMeansHm2$Var14noTNF_2h_mean-baseMeansHm2$RBCnoTNF_2h_mean
baseMeansHm2$Var14_RBC_6h<-baseMeansHm2$Var14noTNF_6h_mean-baseMeansHm2$RBCnoTNF_6h_mean
baseMeansHm2$Var14_RBC_20h<-baseMeansHm2$Var14noTNF_20h_mean-baseMeansHm2$RBCnoTNF_20h_mean
baseMeansHm <-baseMeansHm2[,c(9:12)]
head(baseMeansHm)
```


```
topDEgenes <- which(tempA$Include=="in")####find indexes
```

#### 2. Hierachical clustering of means (individual samples added for inspection)


```
####mean logfc
dataHMm<-baseMeansHm[ topDEgenes, ]
#dataHMm <- log2(dataHMm+1)
dataHMm<- t(as.matrix(dataHMm))
dataHMm <- t(scale(dataHMm))
hmap_hier_factors4 <- Heatmap(
  dataHMm,  name = "logfc",
  row_labels = paste0(rownames(dataHMm)," ",(tempA[ topDEgenes, ])$Gene_Symbol),
  column_title = paste0("Means logfc"), 
  col = col_funGR,
  column_title_gp = gpar(fontsize = 16, fontface = "bold"),
  width = unit(50, "mm"),
  cluster_columns = FALSE,
  show_row_names = FALSE)
dataHMmPlot<-as.data.frame(dataHMm)
dataHMmPlot$Var14_RBC_2h<-dataHMmPlot$Var14_RBC_2h-dataHMmPlot$Var14_RBC_0h
dataHMmPlot$Var14_RBC_6h<-dataHMmPlot$Var14_RBC_6h-dataHMmPlot$Var14_RBC_0h
dataHMmPlot$Var14_RBC_20h<-dataHMmPlot$Var14_RBC_20h-dataHMmPlot$Var14_RBC_0h
dataHMmPlot$Var14_RBC_0h<-dataHMmPlot$Var14_RBC_0h-dataHMmPlot$Var14_RBC_0h
dataHMmPlot<-as.matrix(dataHMmPlot)
  
hmap_hier_factors6 <- Heatmap(
  dataHMmPlot,  name = "Normalised logfc",
  row_labels = paste0(rownames(dataHMm)," ",(tempA[ topDEgenes, ])$Gene_Symbol),
  column_title = paste0("0h Normalised logfc"), 
  col = col_funGR2,
  column_title_gp = gpar(fontsize = 16, fontface = "bold"),
  width = unit(50, "mm"),
  cluster_columns = FALSE,
  show_row_names = FALSE)
####means
dataHMm3<-baseMeansHmM[ topDEgenes, ]
#dataHMm <- log2(dataHMm+1)
dataHMm3<- t(as.matrix(dataHMm3))
dataHMm3 <- t(scale(dataHMm3))
dataHMm3<-dataHMm3[,c(5,1,6,2,7,3,8,4)]
hmap_hier_factors5 <- Heatmap(
  dataHMm3,  name = "Expression",
  row_labels = paste0(rownames(dataHMm)," ",(tempA[ topDEgenes, ])$Gene_Symbol),
  column_title = paste0("Means"), 
  col = col_fun,
  column_title_gp = gpar(fontsize = 16, fontface = "bold"),
  width = unit(100, "mm"),
  cluster_columns = FALSE,
  show_row_names = FALSE)
  
hmap_hier_factors4+hmap_hier_factors6+hmap_hier_factors5
```


```
par(mfrow=c(1,2))
#### Silhouette method
fviz_nbclust(dataHMm, kmeans, method = "silhouette",k.max = 16)+
  labs(subtitle = "Silhouette method")
```


```
#### Elbow method
fviz_nbclust(dataHMm, kmeans, method = "wss",k.max = 16) +
  labs(subtitle = "Elbow method")
```


```
####gap stat slow!!!
####set.seed(123)
####fviz_nbclust(dataHMm, kmeans, nstart = 25,  method = "gap_stat", nboot = 100,k.max = 16)+
####  labs(subtitle = "Gap statistic method")
```


```
#kclust4 <- kmeans(dataHMm, 5)
#silhouette plot
distK<-daisy(dataHMm)
plot(silhouette(kclust4$cluster, distK), col=1:5, border=NA)
```

#### 3. K-means clustering of means


```
split <- paste0("Cluster\n", kclust4$cluster)
#split <- factor(paste0("Cluster\n", kclust3$cluster), levels=c("Cluster\n3","Cluster\n1","Cluster\n4","Cluster\n5","Cluster\n2","Cluster\n6"))
hmap_k <- Heatmap(dataHMm, split=split, cluster_row_slices = FALSE,
                  cluster_columns = FALSE,
                  show_row_names = FALSE,
                  name = "logfc",
                  col = col_funGR,
                  width = unit(50, "mm"),
                  column_title = "means logfc", 
                  column_title_gp = gpar(fontsize = 16, fontface = "bold"))
hmap_k+hmap_hier_factors6+hmap_hier_factors5
```


Mean profiles of clusters


```
clustercount<-data.frame(kclust4$cluster)
clustersizes<-table(clustercount$kclust4.cluster)
clusterMeans<-data.frame(kclust4$centers)
clusterMeans1<-data.frame(t(clusterMeans))
clusterMeans1 <- cbind(rownames(clusterMeans1), clusterMeans1)
orderN<-c("Var14_RBC_0h","Var14_RBC_2h","Var14_RBC_6h","Var14_RBC_20h")#### manual
rownames(clusterMeans1) <- NULL
names(clusterMeans1)[names(clusterMeans1)=="rownames(clusterMeans1)"] <- "Sample"
####clusterMeans1
pX1<-ggplot(data=clusterMeans1, aes(x=Sample, y=X1,group=1)) +
  geom_line()+  geom_point()+ggtitle(paste("Cluster X1 Profile ",clustersizes[1]," genes"))+  scale_x_discrete(limits=orderN)+
  theme(axis.title.x = element_blank(),axis.title.y = element_blank())
pX2<-ggplot(data=clusterMeans1, aes(x=Sample, y=X2,group=1)) +
  geom_line()+  geom_point()+ggtitle(paste("Cluster X2 Profile ",clustersizes[2]," genes"))+  scale_x_discrete(limits=orderN)+
  theme(axis.title.x = element_blank(),axis.title.y = element_blank())
pX3<-ggplot(data=clusterMeans1, aes(x=Sample, y=X3,group=1)) +
  geom_line()+  geom_point()+ggtitle(paste("Cluster X3 Profile ",clustersizes[3]," genes"))+  scale_x_discrete(limits=orderN)+
  theme(axis.title.x = element_blank(),axis.title.y = element_blank())
pX4<-ggplot(data=clusterMeans1, aes(x=Sample, y=X4,group=1)) +
  geom_line()+  geom_point()+ggtitle(paste("Cluster X4 Profile ",clustersizes[4]," genes"))+  scale_x_discrete(limits=orderN)+
  theme(axis.title.x = element_blank(),axis.title.y = element_blank())
pX5<-ggplot(data=clusterMeans1, aes(x=Sample, y=X5,group=1)) +
  geom_line()+  geom_point()+ggtitle(paste("Cluster X5 Profile ",clustersizes[5]," genes"))+  scale_x_discrete(limits=orderN)+
  theme(axis.title.x = element_blank(),axis.title.y = element_blank())
#pX6<-ggplot(data=clusterMeans1, aes(x=Sample, y=X6,group=1)) +
#  geom_line()+  geom_point()+ggtitle(paste("Cluster X6 Profile ",clustersizes[6]," genes"))+  scale_x_discrete(limits=orderN)+
#  theme(axis.title.x = element_blank(),axis.title.y = element_blank())
#plot
multiplot(pX1, pX2, pX3,pX4, pX5,  cols=2)
```


```
topDEgenes <- which(tempA$Include=="in")####find indexes
tempAkm<-tempA[ topDEgenes, ]
SymbolsKm<-dplyr::pull(tempAkm, Gene_Symbol)
#### export the gene expression data for the clusters
write.table(clusterMeans,paste0("ClusterMeansKm_",groupsName,".txt"),  sep = "\t")
ClusteredGenes<-data.frame(kclust4$cluster,SymbolsKm,dataHMm)
write.table(ClusteredGenes,paste0("ScaledDataInClustersKm_",groupsName,".txt"),  sep = "\t")
#head(ClusteredGenes)
```


```
bottomDEgenes<-which(tempA$Include=="out")####find indexes 
bottomG<-tempA[ bottomDEgenes, ]
bottomG<-dplyr::pull(bottomG, Gene_Symbol)
write.table(bottomG,paste0("ipaBottomKmeans_",groupsName,".txt"),  sep = "\t")
                         
topDEgenes <- which(tempA$Include=="in")####find indexes 
tempAkm<-tempA[ topDEgenes, ]
SymbolsKm<-dplyr::pull(tempAkm, Gene_Symbol)
ipaKmeans<-ClusteredGenes
#countsTable <-countsTable[,c(1:15)]####if samples need removing
ipaKmeans<-ipaKmeans[,c(1:2)]
ipaKmeans$name2<-rownames(ipaKmeans)
#ipaKmeans%>% rownames_to_column(var = "rowname")
#ipaKmeans
#rowid_to_column(ipaKmeans)
ipaKmeans = mutate(ipaKmeans, x1= ifelse(ipaKmeans$kclust4.cluster==1, "1", "0"))
ipaKmeans = mutate(ipaKmeans, x2= ifelse(ipaKmeans$kclust4.cluster==2, "1", "0"))
ipaKmeans = mutate(ipaKmeans, x3= ifelse(ipaKmeans$kclust4.cluster==3, "1", "0"))
ipaKmeans = mutate(ipaKmeans, x4= ifelse(ipaKmeans$kclust4.cluster==4, "1", "0"))
ipaKmeans = mutate(ipaKmeans, x5= ifelse(ipaKmeans$kclust4.cluster==5, "1", "0"))
#ipaKmeans = mutate(ipaKmeans, x6= ifelse(ipaKmeans$kclust4.cluster==6, "1", "0"))
#ipaKmeans
write.table(ipaKmeans,paste0("ipaKmeans_",groupsName,".txt"),  sep = "\t")
#head(ipaKmeans)
```


```
ClusteredGenes2<-ClusteredGenes[c(1)]
#ClusteredGenes2
listAll<-list()
for(i in 1:5) {
  clusterName<-paste0("x",i)
  #clusterName<-row.names(subset(ClusteredGenes,ClusteredGenes==i))
  clusterName<-(subset(ClusteredGenes$SymbolsKm,ClusteredGenes==i))
  listAll[[i]]<-clusterName
}
#need to name the vectors in the list, example here is for 8 clusters
names(listAll)<-c("X1", "X2", "X3","X4", "X5")
#if you want to rearrange the order
#listAll<-listAll[c("x3", "x7", "x8", "x2", "x6", "x5", "x4", "x1")]
lapply(listAll, head)
```


```
$X1
[1] "CDH11"  "KCTD12" "DNAJB4" "NFKBIZ" "SAMD9"  "PMAIP1"

$X2
[1] "ABCB1"     "CLEC10A"   "PCDH17"    "RPS7P11"   "FTH1P23"   "EEF1A1P12"

$X3
[1] "PSMD6-AS2" "RGS7BP"    "SNHG26"    "NCR3LG1"   "ANGPTL4"   "KRT7"     

$X4
[1] "CYP1A1" "TXNIP"  "TTC39A" "CCDC68" "KLF4"   "FOSB"  

$X5
[1] "F2RL3"      "FCN3"       "B9D2"       "RAB11FIP1"  "AC139530.1" "PRR29"
```

#### 4. Annotation of K-means clusters

- CC cellular compartment
- BP biological process
- MF molecular function

The simplify function has been used to cut down on GO redundancy


```
#str(AllGeneNames)
```


```
####CC
cgoCC <- compareCluster(geneCluster = listAll, 
                      universe = AllGeneNames,
                      fun = "enrichGO",
                      OrgDb=org.Hs.eg.db, 
                      ####OrgDb=org.Mm.eg.db,
                      keyType="SYMBOL",
                      ont = "CC", 
                      pvalueCutoff=0.05,
                      qvalueCutoff = 0.10)
cgoCC2 <- simplify(cgoCC, cutoff=0.7, by="p.adjust", select_fun=min)
####write as spreadsheet
write.csv(as.data.frame(cgoCC2),paste0("GO_CC_",groupsName,".csv"))

dotplot(cgoCC2,showCategory = 30,
        title = paste0("GO Cellular Compartment ",groupsName))+
  theme(axis.text.x = element_text(angle = 90, vjust = 0.5, hjust=1))
```


Plots and GO data were written to files


```
png(paste0("GO_CC_",groupsName,".png"), width = 1224, height = 824)
dotplot(cgoCC2,showCategory = 30,
        title = paste0("GO Cellular Compartment ",groupsName))+
  theme(axis.text.x = element_text(angle = 90, vjust = 0.5, hjust=1))
dev.off()
```


GO BP


```
####CC
cgoBP <- compareCluster(geneCluster = listAll, 
                      universe = AllGeneNames,
                      fun = "enrichGO",
                      OrgDb=org.Hs.eg.db,
                      keyType="SYMBOL",
                      ont = "BP", 
                      pvalueCutoff=0.05,
                      qvalueCutoff = 0.10)
cgoBP2 <- simplify(cgoBP, cutoff=0.7, by="p.adjust", select_fun=min)
####write as spreadsheet
write.csv(as.data.frame(cgoBP2),paste0("GO_BP_",groupsName,".csv"))
dotplot(cgoBP2,showCategory = 30,
        title = paste0("GO Biological Process ",groupsName))+
  theme(axis.text.x = element_text(angle = 90, vjust = 0.5, hjust=1))
```


```
png(paste0("GO_BP_",groupsName,".png"), width = 1024, height = 1224)
dotplot(cgoBP2,showCategory = 30,
        title = paste0("GO Biological Process ",groupsName))+
  theme(axis.text.x = element_text(angle = 90, vjust = 0.5, hjust=1))
dev.off()
```


```
null device 
          1
```


GO MF


```
####MF
cgoMF <- compareCluster(geneCluster = listAll, 
                      universe = AllGeneNames,
                      fun = "enrichGO",
                      OrgDb=org.Hs.eg.db, 
                      keyType="SYMBOL",
                      ont = "MF", 
                      pvalueCutoff=0.05,
                      qvalueCutoff = 0.10)
cgoMF2 <- simplify(cgoMF, cutoff=0.7, by="p.adjust", select_fun=min)
####write as spreadsheet
write.csv(as.data.frame(cgoMF2),paste0("GO_MF_",groupsName,".csv"))

dotplot(cgoMF2,showCategory = 30,
        title = paste0("GO Molecular Function  ",groupsName))+
  theme(axis.text.x = element_text(angle = 90, vjust = 0.5, hjust=1))
```


```
png(paste0("GO_MF_",groupsName,".png"), width = 1424, height = 824)
dotplot(cgoMF2,showCategory = 30,
        title = paste0("GO Molecular Function  ",groupsName))+
  theme(axis.text.x = element_text(angle = 90, vjust = 0.5, hjust=1))
dev.off()
```

### R3 VAR14 vs RBC no TNF k-means padj0.1fc2

#### 1. Genelist Selection


```
groupsName<-"R3_VAR14_kmeans_padj0.1fc2"
```


```
countsTable<-read.delim("RNAseq2019July_5.txt", header = TRUE, sep = "\t",check.names=FALSE,row.names=1)
head(countsTable)
```


```
AllGeneNames<-countsTable$Gene_Symbol
#head(AllGeneNames)
```


```
grid.arrange(gTree(children=vennp), gTree(children=vennpq) , ncol=2,top="R3 Var14 no TNF")
```


```
#tempA<-resAll[-c(10:30) ]
tempA<-countsTable
#rownames(tempA)
rownames(tempA) <- NULL
tempA = mutate(tempA, Include=
                   ifelse(tempA$padj_R3noTNF_var14_vs_RBC_0h<0.1&abs(tempA$log2FoldChange_R3noTNF_var14_vs_RBC_0h)>1&!is.na(tempA$padj_R3noTNF_var14_vs_RBC_0h), "in",
                          ifelse(tempA$padj_R3noTNF_var14_vs_RBC_2h<0.1&abs(tempA$log2FoldChange_R3noTNF_var14_vs_RBC_2h)>1&!is.na(tempA$padj_R3noTNF_var14_vs_RBC_2h), "in",
                                 ifelse(tempA$padj_R3noTNF_var14_vs_RBC_6h<0.1&abs(tempA$log2FoldChange_R3noTNF_var14_vs_RBC_6h)>1&!is.na(tempA$padj_R3noTNF_var14_vs_RBC_6h), "in",
                                        ifelse(tempA$padj_R3noTNF_var14_vs_RBC_20h<0.1&abs(tempA$log2FoldChange_R3noTNF_var14_vs_RBC_20h)>1&!is.na(tempA$padj_R3noTNF_var14_vs_RBC_20h), "in",
                                                                       "out")))))
#tempA
####library(dplyr)
tempA %>%
     group_by(Include) %>% 
     tally()
```


```
topDEgenes <- which(tempA$Include=="in")####find indexes
```

#### NB Please check columns used and renamed for plots


```
baseMeansHm <-countsTable[,c(60:63,79:82)]
baseMeansHm2 <- log2(baseMeansHm+1)
baseMeansHm2$Var14_RBC_0h<-baseMeansHm2$Var14noTNF_0h_mean-baseMeansHm2$RBCnoTNF_0h_mean
baseMeansHm2$Var14_RBC_2h<-baseMeansHm2$Var14noTNF_2h_mean-baseMeansHm2$RBCnoTNF_2h_mean
baseMeansHm2$Var14_RBC_6h<-baseMeansHm2$Var14noTNF_6h_mean-baseMeansHm2$RBCnoTNF_6h_mean
baseMeansHm2$Var14_RBC_20h<-baseMeansHm2$Var14noTNF_20h_mean-baseMeansHm2$RBCnoTNF_20h_mean
baseMeansHm <-baseMeansHm2[,c(9:12)]
head(baseMeansHm)
```


```
baseMeansHmM <-baseMeansHm2[,c(1:8)]
head(baseMeansHmM)
```


```
topDEgenes <- which(tempA$Include=="in")####find indexes
```

#### 2. Hierachical clustering of means (individual samples added for inspection)


```
####mean logfc
dataHMm<-baseMeansHm[ topDEgenes, ]
#dataHMm <- log2(dataHMm+1)
dataHMm<- t(as.matrix(dataHMm))
dataHMm <- t(scale(dataHMm))
hmap_hier_factors4 <- Heatmap(
  dataHMm,  name = "logfc",
  row_labels = paste0(rownames(dataHMm)," ",(tempA[ topDEgenes, ])$Gene_Symbol),
  column_title = paste0("Means logfc"), 
  col = col_funGR,
  column_title_gp = gpar(fontsize = 16, fontface = "bold"),
  width = unit(50, "mm"),
  cluster_columns = FALSE,
  show_row_names = FALSE)
dataHMmPlot<-as.data.frame(dataHMm)
dataHMmPlot$Var14_RBC_2h<-dataHMmPlot$Var14_RBC_2h-dataHMmPlot$Var14_RBC_0h
dataHMmPlot$Var14_RBC_6h<-dataHMmPlot$Var14_RBC_6h-dataHMmPlot$Var14_RBC_0h
dataHMmPlot$Var14_RBC_20h<-dataHMmPlot$Var14_RBC_20h-dataHMmPlot$Var14_RBC_0h
dataHMmPlot$Var14_RBC_0h<-dataHMmPlot$Var14_RBC_0h-dataHMmPlot$Var14_RBC_0h
dataHMmPlot<-as.matrix(dataHMmPlot)
  
hmap_hier_factors6 <- Heatmap(
  dataHMmPlot,  name = "Normalised logfc",
  row_labels = paste0(rownames(dataHMm)," ",(tempA[ topDEgenes, ])$Gene_Symbol),
  column_title = paste0("0h Normalised logfc"), 
  col = col_funGR2,
  column_title_gp = gpar(fontsize = 16, fontface = "bold"),
  width = unit(50, "mm"),
  cluster_columns = FALSE,
  show_row_names = FALSE)
####means
dataHMm3<-baseMeansHmM[ topDEgenes, ]
#dataHMm <- log2(dataHMm+1)
dataHMm3<- t(as.matrix(dataHMm3))
dataHMm3 <- t(scale(dataHMm3))
dataHMm3<-dataHMm3[,c(5,1,6,2,7,3,8,4)]
hmap_hier_factors5 <- Heatmap(
  dataHMm3,  name = "Expression",
  row_labels = paste0(rownames(dataHMm)," ",(tempA[ topDEgenes, ])$Gene_Symbol),
  column_title = paste0("Means"), 
  col = col_fun,
  column_title_gp = gpar(fontsize = 16, fontface = "bold"),
  width = unit(100, "mm"),
  cluster_columns = FALSE,
  show_row_names = FALSE)
  
hmap_hier_factors4+hmap_hier_factors6+hmap_hier_factors5
```


```
par(mfrow=c(1,2))
#### Silhouette method
fviz_nbclust(dataHMm, kmeans, method = "silhouette",k.max = 16)+
  labs(subtitle = "Silhouette method")
```


```
#### Elbow method
fviz_nbclust(dataHMm, kmeans, method = "wss",k.max = 16) +
  labs(subtitle = "Elbow method")
```


```
####gap stat slow!!!
####set.seed(123)
####fviz_nbclust(dataHMm, kmeans, nstart = 25,  method = "gap_stat", nboot = 100,k.max = 16)+
####  labs(subtitle = "Gap statistic method")
```


```
kclust4b <- kmeans(dataHMm, 3)
#silhouette plot
distK<-daisy(dataHMm)
plot(silhouette(kclust4b$cluster, distK), col=1:3, border=NA)
```

#### 3. K-means clustering of means


```
split <- paste0("Cluster\n", kclust4b$cluster)
#split <- factor(paste0("Cluster\n", kclust3$cluster), levels=c("Cluster\n3","Cluster\n1","Cluster\n4","Cluster\n5","Cluster\n2","Cluster\n6"))
hmap_k <- Heatmap(dataHMm, split=split, cluster_row_slices = FALSE,
                  cluster_columns = FALSE,
                  show_row_names = FALSE,
                  name = "logfc",
                  col = col_funGR,
                  width = unit(50, "mm"),
                  column_title = "means logfc", 
                  column_title_gp = gpar(fontsize = 16, fontface = "bold"))
hmap_k+hmap_hier_factors6+hmap_hier_factors5
```


Mean profiles of clusters


```
clustercount<-data.frame(kclust4b$cluster)
clustersizes<-table(clustercount$kclust4b.cluster)
clusterMeans<-data.frame(kclust4b$centers)
clusterMeans1<-data.frame(t(clusterMeans))
clusterMeans1 <- cbind(rownames(clusterMeans1), clusterMeans1)
orderN<-c("Var14_RBC_0h","Var14_RBC_2h","Var14_RBC_6h","Var14_RBC_20h")#### manual
rownames(clusterMeans1) <- NULL
names(clusterMeans1)[names(clusterMeans1)=="rownames(clusterMeans1)"] <- "Sample"
####clusterMeans1
pX1<-ggplot(data=clusterMeans1, aes(x=Sample, y=X1,group=1)) +
  geom_line()+  geom_point()+ggtitle(paste("Cluster X1 Profile ",clustersizes[1]," genes"))+  scale_x_discrete(limits=orderN)+
  theme(axis.title.x = element_blank(),axis.title.y = element_blank())
pX2<-ggplot(data=clusterMeans1, aes(x=Sample, y=X2,group=1)) +
  geom_line()+  geom_point()+ggtitle(paste("Cluster X2 Profile ",clustersizes[2]," genes"))+  scale_x_discrete(limits=orderN)+
  theme(axis.title.x = element_blank(),axis.title.y = element_blank())
pX3<-ggplot(data=clusterMeans1, aes(x=Sample, y=X3,group=1)) +
  geom_line()+  geom_point()+ggtitle(paste("Cluster X3 Profile ",clustersizes[3]," genes"))+  scale_x_discrete(limits=orderN)+
  theme(axis.title.x = element_blank(),axis.title.y = element_blank())
#pX4<-ggplot(data=clusterMeans1, aes(x=Sample, y=X4,group=1)) +
#  geom_line()+  geom_point()+ggtitle(paste("Cluster X4 Profile ",clustersizes[4]," genes"))+  scale_x_discrete(limits=orderN)+
#  theme(axis.title.x = element_blank(),axis.title.y = element_blank())
#pX5<-ggplot(data=clusterMeans1, aes(x=Sample, y=X5,group=1)) +
#  geom_line()+  geom_point()+ggtitle(paste("Cluster X5 Profile ",clustersizes[5]," genes"))+  scale_x_discrete(limits=orderN)+
#  theme(axis.title.x = element_blank(),axis.title.y = element_blank())
#pX6<-ggplot(data=clusterMeans1, aes(x=Sample, y=X6,group=1)) +
#  geom_line()+  geom_point()+ggtitle(paste("Cluster X6 Profile ",clustersizes[6]," genes"))+  scale_x_discrete(limits=orderN)+
#  theme(axis.title.x = element_blank(),axis.title.y = element_blank())
#plot
multiplot(pX1, pX2, pX3,  cols=2)
```


```
topDEgenes <- which(tempA$Include=="in")####find indexes
tempAkm<-tempA[ topDEgenes, ]
SymbolsKm<-dplyr::pull(tempAkm, Gene_Symbol)
#### export the gene expression data for the clusters
write.table(clusterMeans,paste0("ClusterMeansKm_",groupsName,".txt"),  sep = "\t")
ClusteredGenes<-data.frame(kclust4b$cluster,SymbolsKm,dataHMm)
write.table(ClusteredGenes,paste0("ScaledDataInClustersKm_",groupsName,".txt"),  sep = "\t")
#head(ClusteredGenes)
```


```
bottomDEgenes<-which(tempA$Include=="out")####find indexes 
bottomG<-tempA[ bottomDEgenes, ]
bottomG<-dplyr::pull(bottomG, Gene_Symbol)
write.table(bottomG,paste0("ipaBottomKmeans_",groupsName,".txt"),  sep = "\t")
                         
topDEgenes <- which(tempA$Include=="in")####find indexes 
tempAkm<-tempA[ topDEgenes, ]
SymbolsKm<-dplyr::pull(tempAkm, Gene_Symbol)
ipaKmeans<-ClusteredGenes
#countsTable <-countsTable[,c(1:15)]####if samples need removing
ipaKmeans<-ipaKmeans[,c(1:2)]
ipaKmeans$name2<-rownames(ipaKmeans)
#ipaKmeans%>% rownames_to_column(var = "rowname")
#ipaKmeans
#rowid_to_column(ipaKmeans)
ipaKmeans = mutate(ipaKmeans, x1= ifelse(ipaKmeans$kclust4b.cluster==1, "1", "0"))
ipaKmeans = mutate(ipaKmeans, x2= ifelse(ipaKmeans$kclust4b.cluster==2, "1", "0"))
ipaKmeans = mutate(ipaKmeans, x3= ifelse(ipaKmeans$kclust4b.cluster==3, "1", "0"))
#ipaKmeans = mutate(ipaKmeans, x4= ifelse(ipaKmeans$kclust4b.cluster==4, "1", "0"))
#ipaKmeans = mutate(ipaKmeans, x5= ifelse(ipaKmeans$kclust4b.cluster==5, "1", "0"))
#ipaKmeans = mutate(ipaKmeans, x6= ifelse(ipaKmeans$kclust4b.cluster==6, "1", "0"))
#ipaKmeans
write.table(ipaKmeans,paste0("ipaKmeans_",groupsName,".txt"),  sep = "\t")
#head(ipaKmeans)
```


```
ClusteredGenes2<-ClusteredGenes[c(1)]
#ClusteredGenes2
listAll<-list()
for(i in 1:3) {
  clusterName<-paste0("x",i)
  #clusterName<-row.names(subset(ClusteredGenes,ClusteredGenes==i))
  clusterName<-(subset(ClusteredGenes$SymbolsKm,ClusteredGenes==i))
  listAll[[i]]<-clusterName
}
#need to name the vectors in the list, example here is for 8 clusters
names(listAll)<-c("X1", "X2", "X3")
#if you want to rearrange the order
#listAll<-listAll[c("x3", "x7", "x8", "x2", "x6", "x5", "x4", "x1")]
lapply(listAll, head)
```


```
$X1
[1] "CYP1A1" "KCTD12" "TXNIP"  "DNAJB4" "PMAIP1" "USP53" 

$X2
[1] "PRR29"    "CLDN5"    "AMH"      "TRMT61A"  "DUS3L"    "EEF1A1P4"

$X3
[1] "ANGPTL4"    "KRT7"       "STARD4-AS1" "ZNF770"     "CEP295"
```

#### 4. Annotation of K-means clusters

- CC cellular compartment
- BP biological process
- MF molecular function

The simplify function has been used to cut down on GO redundancy


```
#str(AllGeneNames)
```


```
####CC
cgoCC <- compareCluster(geneCluster = listAll, 
                      universe = AllGeneNames,
                      fun = "enrichGO",
                      OrgDb=org.Hs.eg.db, 
                      ####OrgDb=org.Mm.eg.db,
                      keyType="SYMBOL",
                      ont = "CC", 
                      pvalueCutoff=0.05,
                      qvalueCutoff = 0.10)
cgoCC2 <- simplify(cgoCC, cutoff=0.7, by="p.adjust", select_fun=min)
####write as spreadsheet
write.csv(as.data.frame(cgoCC2),paste0("GO_CC_",groupsName,".csv"))
dotplot(cgoCC2,showCategory = 30,
        title = paste0("GO Cellular Compartment ",groupsName))+
  theme(axis.text.x = element_text(angle = 90, vjust = 0.5, hjust=1))
```


Plots and GO data were written to files


```
png(paste0("GO_CC_",groupsName,".png"), width = 1224, height = 824)
dotplot(cgoCC2,showCategory = 30,
        title = paste0("GO Cellular Compartment ",groupsName))+
  theme(axis.text.x = element_text(angle = 90, vjust = 0.5, hjust=1))
dev.off()
```


```
null device 
          1
```


GO BP


```
####CC
cgoBP <- compareCluster(geneCluster = listAll, 
                      universe = AllGeneNames,
                      fun = "enrichGO",
                      OrgDb=org.Hs.eg.db,
                      keyType="SYMBOL",
                      ont = "BP", 
                      pvalueCutoff=0.05,
                      qvalueCutoff = 0.10)
cgoBP2 <- simplify(cgoBP, cutoff=0.7, by="p.adjust", select_fun=min)
####write as spreadsheet
write.csv(as.data.frame(cgoBP2),paste0("GO_BP_",groupsName,".csv"))
dotplot(cgoBP2,showCategory = 30,
        title = paste0("GO Biological Process ",groupsName))+
  theme(axis.text.x = element_text(angle = 90, vjust = 0.5, hjust=1))
```


```
png(paste0("GO_BP_",groupsName,".png"), width = 1024, height = 1224)
dotplot(cgoBP2,showCategory = 30,
        title = paste0("GO Biological Process ",groupsName))+
  theme(axis.text.x = element_text(angle = 90, vjust = 0.5, hjust=1))
dev.off()
```


```
null device 
          1
```


GO MF


```
####MF
cgoMF <- compareCluster(geneCluster = listAll, 
                      universe = AllGeneNames,
                      fun = "enrichGO",
                      OrgDb=org.Hs.eg.db, 
                      keyType="SYMBOL",
                      ont = "MF", 
                      pvalueCutoff=0.05,
                      qvalueCutoff = 0.10)
cgoMF2 <- simplify(cgoMF, cutoff=0.7, by="p.adjust", select_fun=min)
####write as spreadsheet
write.csv(as.data.frame(cgoMF2),paste0("GO_MF_",groupsName,".csv"))
dotplot(cgoMF2,showCategory = 30,
        title = paste0("GO Molecular Function  ",groupsName))+
  theme(axis.text.x = element_text(angle = 90, vjust = 0.5, hjust=1))
```


```
png(paste0("GO_MF_",groupsName,".png"), width = 1424, height = 824)
dotplot(cgoMF2,showCategory = 30,
        title = paste0("GO Molecular Function  ",groupsName))+
  theme(axis.text.x = element_text(angle = 90, vjust = 0.5, hjust=1))
dev.off()
```


```
null device 
          1
```

### R5 VAR14 vs RBC TNF k-means q0.05

#### 1. Genelist Selection


```
groupsName<-"R5_VAR14_RBC_TNF_kmeans_q0.05"
```


```
countsTable<-read.delim("RNAseq2019July_5.txt", header = TRUE, sep = "\t",check.names=FALSE,row.names=1)
head(countsTable)
```


```
AllGeneNames<-countsTable$Gene_Symbol
#head(AllGeneNames)
```


```
tempA<-countsTable
```


```
topDEgenes <- which(tempA$padj_R5_TNF_var14_vs_RBC_0h<0.05&!is.na(tempA$padj_R5_TNF_var14_vs_RBC_0h))####find indexes 
listA<-tempA[ topDEgenes, ]$Gene_Symbol
topDEgenes <- which(tempA$padj_R5_TNF_var14_vs_RBC_2h<0.05&!is.na(tempA$padj_R5_TNF_var14_vs_RBC_2h))####find indexes 
listB<-tempA[ topDEgenes, ]$Gene_Symbol
topDEgenes <- which(tempA$padj_R5_TNF_var14_vs_RBC_6h<0.05&!is.na(tempA$padj_R5_TNF_var14_vs_RBC_6h))####find indexes 
listC<-tempA[ topDEgenes, ]$Gene_Symbol
topDEgenes <- which(tempA$padj_R5_TNF_var14_vs_RBC_20h<0.05&!is.na(tempA$padj_R5_TNF_var14_vs_RBC_20h))####find indexes 
listD<-tempA[ topDEgenes, ]$Gene_Symbol
vennq<-venn.diagram(x = list(listA,listB,listC,listD) ,
            category.names = c("Var14nNF_0h","Var14TNF_2h","Var14TNF_6h","Var14TNF_20h"),
            main="padj<0.05",
            filename = NULL,  scaled = FALSE, fill = colorsV4, cat.col = colorsV4, cat.cex = 1, cat.dist=0.3,  margin = 0.3)
topDEgenes <- which(tempA$pvalue_R5_TNF_var14_vs_RBC_0h<0.05&abs(tempA$log2FoldChange_R5_TNF_var14_vs_RBC_0h)>1&!is.na(tempA$pvalue_R5_TNF_var14_vs_RBC_0h))####find indexes 
listA<-tempA[ topDEgenes, ]$Gene_Symbol
topDEgenes <- which(tempA$pvalue_R5_TNF_var14_vs_RBC_2h<0.05&abs(tempA$log2FoldChange_R5_TNF_var14_vs_RBC_2h)>1&!is.na(tempA$pvalue_R5_TNF_var14_vs_RBC_2h))####find indexes 
listB<-tempA[ topDEgenes, ]$Gene_Symbol
topDEgenes <- which(tempA$pvalue_R5_TNF_var14_vs_RBC_6h<0.05&abs(tempA$log2FoldChange_R5_TNF_var14_vs_RBC_6h)>1&!is.na(tempA$pvalue_R5_TNF_var14_vs_RBC_6h))####find indexes 
listC<-tempA[ topDEgenes, ]$Gene_Symbol
topDEgenes <- which(tempA$pvalue_R5_TNF_var14_vs_RBC_20h<0.05&abs(tempA$log2FoldChange_R5_TNF_var14_vs_RBC_20h)>1&!is.na(tempA$pvalue_R5_TNF_var14_vs_RBC_20h))####find indexes 
listD<-tempA[ topDEgenes, ]$Gene_Symbol
vennp<-venn.diagram(x = list(listA,listB,listC,listD) ,
            category.names = c("Var14TNF_0h","Var14TNF_2h","Var14TNF_6h","Var14TNF_20h"),
            main="pvalue<0.05&fold change>2",
            filename = NULL,  scaled = FALSE, fill = colorsV4, cat.col = colorsV4, cat.cex = 1, cat.dist=0.3,  margin = 0.3)
topDEgenes <- which(tempA$padj_R5_TNF_var14_vs_RBC_0h<0.1&abs(tempA$log2FoldChange_R5_TNF_var14_vs_RBC_0h)>1&!is.na(tempA$padj_R5_TNF_var14_vs_RBC_0h))####find indexes 
listA<-tempA[ topDEgenes, ]$Gene_Symbol
topDEgenes <- which(tempA$padj_R5_TNF_var14_vs_RBC_2h<0.1&abs(tempA$log2FoldChange_R5_TNF_var14_vs_RBC_2h)>1&!is.na(tempA$padj_R5_TNF_var14_vs_RBC_2h))####find indexes 
listB<-tempA[ topDEgenes, ]$Gene_Symbol
topDEgenes <- which(tempA$padj_R5_TNF_var14_vs_RBC_6h<0.1&abs(tempA$log2FoldChange_R5_TNF_var14_vs_RBC_6h)>1&!is.na(tempA$padj_R5_TNF_var14_vs_RBC_6h))####find indexes 
listC<-tempA[ topDEgenes, ]$Gene_Symbol
topDEgenes <- which(tempA$padj_R5_TNF_var14_vs_RBC_20h<0.1&abs(tempA$log2FoldChange_R5_TNF_var14_vs_RBC_20h)>1&!is.na(tempA$padj_R5_TNF_var14_vs_RBC_20h))####find indexes 
listD<-tempA[ topDEgenes, ]$Gene_Symbol
vennq2<-venn.diagram(x = list(listA,listB,listC,listD) ,
            category.names = c("Var14TNF_0h","Var14TNF_2h","Var14TNF_6h","Var14TNF_20h"),
            main="padj0.1&fold change>2",
            filename = NULL,  scaled = FALSE, fill = colorsV4, cat.col = colorsV4, cat.cex = 1, cat.dist=0.3,  margin = 0.3)
```


```
topDEgenes <- which((tempA$padj_R5_TNF_var14_vs_RBC_0h<0.05&!is.na(tempA$padj_R5_TNF_var14_vs_RBC_0h))| 
(tempA$padj_R5_TNF_var14_vs_RBC_2h<0.05&!is.na(tempA$padj_R5_TNF_var14_vs_RBC_2h))|
(tempA$padj_R5_TNF_var14_vs_RBC_6h<0.05&!is.na(tempA$padj_R5_TNF_var14_vs_RBC_6h))| 
(tempA$padj_R5_TNF_var14_vs_RBC_20h<0.05&!is.na(tempA$padj_R5_TNF_var14_vs_RBC_20h)) 
)
listA<-tempA[ topDEgenes, ]$Gene_Symbol
topDEgenes <- which((tempA$pvalue_R5_TNF_var14_vs_RBC_0h<0.05&abs(tempA$log2FoldChange_R5_TNF_var14_vs_RBC_0h)>1&!is.na(tempA$pvalue_R5_TNF_var14_vs_RBC_0h))| 
(tempA$pvalue_R5_TNF_var14_vs_RBC_2h<0.05&abs(tempA$log2FoldChange_R5_TNF_var14_vs_RBC_2h)>1&!is.na(tempA$pvalue_R5_TNF_var14_vs_RBC_2h))| 
(tempA$pvalue_R5_TNF_var14_vs_RBC_6h<0.05&abs(tempA$log2FoldChange_R5_TNF_var14_vs_RBC_6h)>1&!is.na(tempA$pvalue_R5_TNF_var14_vs_RBC_6h))| 
(tempA$pvalue_R5_TNF_var14_vs_RBC_20h<0.05&abs(tempA$log2FoldChange_R5_TNF_var14_vs_RBC_20h)>1&!is.na(tempA$pvalue_R5_TNF_var14_vs_RBC_20h))
 )####find indexes 
listC<-tempA[ topDEgenes, ]$Gene_Symbol
topDEgenes <- which((tempA$padj_R5_TNF_var14_vs_RBC_0h<0.1&abs(tempA$log2FoldChange_R5_TNF_var14_vs_RBC_0h)>1&!is.na(tempA$padj_R5_TNF_var14_vs_RBC_0h))| 
(tempA$padj_R5_TNF_var14_vs_RBC_2h<0.1&abs(tempA$log2FoldChange_R5_TNF_var14_vs_RBC_2h)>1&!is.na(tempA$padj_R5_TNF_var14_vs_RBC_2h))| 
(tempA$padj_R5_TNF_var14_vs_RBC_6h<0.1&abs(tempA$log2FoldChange_R5_TNF_var14_vs_RBC_6h)>1&!is.na(tempA$padj_R5_TNF_var14_vs_RBC_6h))| 
(tempA$padj_R5_TNF_var14_vs_RBC_20h<0.1&abs(tempA$log2FoldChange_R5_TNF_var14_vs_RBC_20h)>1&!is.na(tempA$padj_R5_TNF_var14_vs_RBC_20h))
 )####find indexes 
listB<-tempA[ topDEgenes, ]$Gene_Symbol
vennpq<-venn.diagram(x = list(listA,listB,listC) ,
            category.names = c("padj<0.05","padj<0.1&fc>2","p<0.05&fc>2"),
            main="padj compared to pvalue",
            filename = NULL,  scaled = FALSE, fill = colorsV3, cat.col = colorsV3, cat.cex = 1, cat.dist=0.1,  margin = 0.15)
```


```
grid.arrange(gTree(children=vennq), gTree(children=vennpq), ncol=2,top="R2 Var14 TNF")
```


```
#tempA<-resAll[-c(10:30) ]
tempA<-countsTable
#rownames(tempA)
rownames(tempA) <- NULL
tempA = mutate(tempA, Include=
                   ifelse(tempA$padj_R5_TNF_var14_vs_RBC_0h<0.05&!is.na(tempA$padj_R5_TNF_var14_vs_RBC_0h), "in",
                          ifelse(tempA$padj_R5_TNF_var14_vs_RBC_2h<0.05&!is.na(tempA$padj_R5_TNF_var14_vs_RBC_2h), "in",
                                 ifelse(tempA$padj_R5_TNF_var14_vs_RBC_6h<0.05&!is.na(tempA$padj_R5_TNF_var14_vs_RBC_6h), "in",
                                        ifelse(tempA$padj_R5_TNF_var14_vs_RBC_20h<0.05&!is.na(tempA$padj_R5_TNF_var14_vs_RBC_20h), "in",
                                                 "out")))))
#tempA
####library(dplyr)
tempA %>%
     group_by(Include) %>% 
     tally()
```


```
topDEgenes <- which(tempA$Include=="in")####find indexes
```


```
head(countsTable)
```

#### NB Please check columns used and renamed for plots


```
#baseMeansHm <-countsTable[,c(60:63)]
baseMeansHm <-countsTable[,c(110:113,129:132)]
head(baseMeansHm)
```


```
baseMeansHm2 <- log2(baseMeansHm+1)
baseMeansHm2$Var14_RBC_0h<-baseMeansHm2$Var14TNF_0h_mean-baseMeansHm2$RBC_TNF_0h_mean
baseMeansHm2$Var14_RBC_2h<-baseMeansHm2$Var14TNF_2h_mean-baseMeansHm2$RBC_TNF_2h_mean
baseMeansHm2$Var14_RBC_6h<-baseMeansHm2$Var14TNF_6h_mean-baseMeansHm2$RBC_TNF_6h_mean
baseMeansHm2$Var14_RBC_20h<-baseMeansHm2$Var14TNF_20h_mean-baseMeansHm2$RBC_TNF_20h_mean
baseMeansHm <-baseMeansHm2[,c(9:12)]
head(baseMeansHm)
```


```
baseMeansHmM <-baseMeansHm2[,c(1:8)]
head(baseMeansHmM)
```


```
topDEgenes <- which(tempA$Include=="in")####find indexes
```

#### 2. Hierachical clustering of means (individual samples added for inspection)


```
####mean logfc
dataHMm<-baseMeansHm[ topDEgenes, ]
#dataHMm <- log2(dataHMm+1)
dataHMm<- t(as.matrix(dataHMm))
dataHMm <- t(scale(dataHMm))
hmap_hier_factors4 <- Heatmap(
  dataHMm,  name = "logfc",
  row_labels = paste0(rownames(dataHMm)," ",(tempA[ topDEgenes, ])$Gene_Symbol),
  column_title = paste0("Means logfc"), 
  col = col_funGR,
  column_title_gp = gpar(fontsize = 16, fontface = "bold"),
  width = unit(50, "mm"),
  cluster_columns = FALSE,
  show_row_names = FALSE)
dataHMmPlot<-as.data.frame(dataHMm)
dataHMmPlot$Var14_RBC_2h<-dataHMmPlot$Var14_RBC_2h-dataHMmPlot$Var14_RBC_0h
dataHMmPlot$Var14_RBC_6h<-dataHMmPlot$Var14_RBC_6h-dataHMmPlot$Var14_RBC_0h
dataHMmPlot$Var14_RBC_20h<-dataHMmPlot$Var14_RBC_20h-dataHMmPlot$Var14_RBC_0h
dataHMmPlot$Var14_RBC_0h<-dataHMmPlot$Var14_RBC_0h-dataHMmPlot$Var14_RBC_0h
dataHMmPlot<-as.matrix(dataHMmPlot)
  
hmap_hier_factors6 <- Heatmap(
  dataHMmPlot,  name = "Normalised logfc",
  row_labels = paste0(rownames(dataHMm)," ",(tempA[ topDEgenes, ])$Gene_Symbol),
  column_title = paste0("0h Normalised logfc"), 
  col = col_funGR2,
  column_title_gp = gpar(fontsize = 16, fontface = "bold"),
  width = unit(50, "mm"),
  cluster_columns = FALSE,
  show_row_names = FALSE)
####means
dataHMm3<-baseMeansHmM[ topDEgenes, ]
#dataHMm <- log2(dataHMm+1)
dataHMm3<- t(as.matrix(dataHMm3))
dataHMm3 <- t(scale(dataHMm3))
dataHMm3<-dataHMm3[,c(5,1,6,2,7,3,8,4)]
hmap_hier_factors5 <- Heatmap(
  dataHMm3,  name = "Expression",
  row_labels = paste0(rownames(dataHMm)," ",(tempA[ topDEgenes, ])$Gene_Symbol),
  column_title = paste0("Means"), 
  col = col_fun,
  column_title_gp = gpar(fontsize = 16, fontface = "bold"),
  width = unit(100, "mm"),
  cluster_columns = FALSE,
  show_row_names = FALSE)
  
hmap_hier_factors4+hmap_hier_factors6+hmap_hier_factors5
```


```
par(mfrow=c(1,2))
#### Silhouette method
fviz_nbclust(dataHMm, kmeans, method = "silhouette",k.max = 16)+
  labs(subtitle = "Silhouette method")
```


```
#### Elbow method
fviz_nbclust(dataHMm, kmeans, method = "wss",k.max = 16) +
  labs(subtitle = "Elbow method")
```


```
####gap stat slow!!!
####set.seed(123)
####fviz_nbclust(dataHMm, kmeans, nstart = 25,  method = "gap_stat", nboot = 100,k.max = 16)+
####  labs(subtitle = "Gap statistic method")
```


```
kclust7 <- kmeans(dataHMm, 6)
#silhouette plot
distK<-daisy(dataHMm)
plot(silhouette(kclust7$cluster, distK), col=1:6, border=NA)
```

#### 3. K-means clustering of means


```
split <- paste0("Cluster\n", kclust7$cluster)
#split <- factor(paste0("Cluster\n", kclust3$cluster), levels=c("Cluster\n3","Cluster\n1","Cluster\n4","Cluster\n5","Cluster\n2","Cluster\n6"))
hmap_k <- Heatmap(dataHMm, split=split, cluster_row_slices = FALSE,
                  cluster_columns = FALSE,
                  show_row_names = FALSE,
                  name = "logfc",
                  col = col_funGR,
                  width = unit(50, "mm"),
                  column_title = "means logfc", 
                  column_title_gp = gpar(fontsize = 16, fontface = "bold"))
hmap_k+hmap_hier_factors6+hmap_hier_factors5
```


Mean profiles of clusters


```
clustercount<-data.frame(kclust7$cluster)
clustersizes<-table(clustercount$kclust7.cluster)
clusterMeans<-data.frame(kclust7$centers)
clusterMeans1<-data.frame(t(clusterMeans))
clusterMeans1 <- cbind(rownames(clusterMeans1), clusterMeans1)
orderN<-c("Var14_RBC_0h","Var14_RBC_2h","Var14_RBC_6h","Var14_RBC_20h")#### manual
rownames(clusterMeans1) <- NULL
names(clusterMeans1)[names(clusterMeans1)=="rownames(clusterMeans1)"] <- "Sample"
pX1<-ggplot(data=clusterMeans1, aes(x=Sample, y=X1,group=1)) +
  geom_line()+  geom_point()+ggtitle(paste("Cluster X1 Profile ",clustersizes[1]," genes"))+  scale_x_discrete(limits=orderN)+
  theme(axis.title.x = element_blank(),axis.title.y = element_blank())
pX2<-ggplot(data=clusterMeans1, aes(x=Sample, y=X2,group=1)) +
  geom_line()+  geom_point()+ggtitle(paste("Cluster X2 Profile ",clustersizes[2]," genes"))+  scale_x_discrete(limits=orderN)+
  theme(axis.title.x = element_blank(),axis.title.y = element_blank())
pX3<-ggplot(data=clusterMeans1, aes(x=Sample, y=X3,group=1)) +
  geom_line()+  geom_point()+ggtitle(paste("Cluster X3 Profile ",clustersizes[3]," genes"))+  scale_x_discrete(limits=orderN)+
  theme(axis.title.x = element_blank(),axis.title.y = element_blank())
pX4<-ggplot(data=clusterMeans1, aes(x=Sample, y=X4,group=1)) +
  geom_line()+  geom_point()+ggtitle(paste("Cluster X4 Profile ",clustersizes[4]," genes"))+  scale_x_discrete(limits=orderN)+
  theme(axis.title.x = element_blank(),axis.title.y = element_blank())
pX5<-ggplot(data=clusterMeans1, aes(x=Sample, y=X5,group=1)) +
  geom_line()+  geom_point()+ggtitle(paste("Cluster X5 Profile ",clustersizes[5]," genes"))+  scale_x_discrete(limits=orderN)+
  theme(axis.title.x = element_blank(),axis.title.y = element_blank())
pX6<-ggplot(data=clusterMeans1, aes(x=Sample, y=X6,group=1)) +
  geom_line()+  geom_point()+ggtitle(paste("Cluster X6 Profile ",clustersizes[6]," genes"))+  scale_x_discrete(limits=orderN)+
  theme(axis.title.x = element_blank(),axis.title.y = element_blank())
#plot
multiplot(pX1, pX2, pX3, pX4,pX5, pX6, cols=2)
```


```
topDEgenes <- which(tempA$Include=="in")####find indexes
tempAkm<-tempA[ topDEgenes, ]
SymbolsKm<-dplyr::pull(tempAkm, Gene_Symbol)
#### export the gene expression data for the clusters
write.table(clusterMeans,paste0("ClusterMeansKm_",groupsName,".txt"),  sep = "\t")
ClusteredGenes<-data.frame(kclust7$cluster,SymbolsKm,dataHMm)
write.table(ClusteredGenes,paste0("ScaledDataInClustersKm_",groupsName,".txt"),  sep = "\t")
#head(ClusteredGenes)
```


```
bottomDEgenes<-which(tempA$Include=="out")####find indexes 
bottomG<-tempA[ bottomDEgenes, ]
bottomG<-dplyr::pull(bottomG, Gene_Symbol)
write.table(bottomG,paste0("ipaBottomKmeans_",groupsName,".txt"),  sep = "\t")
                         
topDEgenes <- which(tempA$Include=="in")####find indexes 
tempAkm<-tempA[ topDEgenes, ]
SymbolsKm<-dplyr::pull(tempAkm, Gene_Symbol)
ipaKmeans<-ClusteredGenes
#countsTable <-countsTable[,c(1:15)]####if samples need removing
ipaKmeans<-ipaKmeans[,c(1:2)]
ipaKmeans$name2<-rownames(ipaKmeans)
#ipaKmeans%>% rownames_to_column(var = "rowname")
#ipaKmeans
#rowid_to_column(ipaKmeans)
ipaKmeans = mutate(ipaKmeans, x1= ifelse(ipaKmeans$kclust7.cluster==1, "1", "0"))
ipaKmeans = mutate(ipaKmeans, x2= ifelse(ipaKmeans$kclust7.cluster==2, "1", "0"))
ipaKmeans = mutate(ipaKmeans, x3= ifelse(ipaKmeans$kclust7.cluster==3, "1", "0"))
ipaKmeans = mutate(ipaKmeans, x4= ifelse(ipaKmeans$kclust7.cluster==4, "1", "0"))
ipaKmeans = mutate(ipaKmeans, x5= ifelse(ipaKmeans$kclust7.cluster==5, "1", "0"))
ipaKmeans = mutate(ipaKmeans, x6= ifelse(ipaKmeans$kclust7.cluster==6, "1", "0"))
#ipaKmeans
write.table(ipaKmeans,paste0("ipaKmeans_",groupsName,".txt"),  sep = "\t")
#head(ipaKmeans)
```


```
ClusteredGenes2<-ClusteredGenes[c(1)]
#ClusteredGenes2
listAll<-list()
for(i in 1:6) {
  clusterName<-paste0("x",i)
  #clusterName<-row.names(subset(ClusteredGenes,ClusteredGenes==i))
  clusterName<-(subset(ClusteredGenes$SymbolsKm,ClusteredGenes==i))
  listAll[[i]]<-clusterName
}
#need to name the vectors in the list, example here is for 8 clusters
names(listAll)<-c("X1", "X2", "X3", "X4","X5", "X6")
#if you want to rearrange the order
#listAll<-listAll[c("x3", "x7", "x8", "x2", "x6", "x5", "x4", "x1")]
#lapply(listAll, head)
```

#### 4. Annotation of K-means clusters

- CC cellular compartment
- BP biological process
- MF molecular function

The simplify function has been used to cut down on GO redundancy


```
#str(AllGeneNames)
```


```
####CC
cgoCC <- compareCluster(geneCluster = listAll, 
                      universe = AllGeneNames,
                      fun = "enrichGO",
                      OrgDb=org.Hs.eg.db, 
                      ####OrgDb=org.Mm.eg.db,
                      keyType="SYMBOL",
                      ont = "CC", 
                      pvalueCutoff=0.05,
                      qvalueCutoff = 0.10)
cgoCC2 <- simplify(cgoCC, cutoff=0.7, by="p.adjust", select_fun=min)
####write as spreadsheet
write.csv(as.data.frame(cgoCC2),paste0("GO_CC_",groupsName,".csv"))
dotplot(cgoCC2,showCategory = 30,
        title = paste0("GO Cellular Compartment ",groupsName))+
  theme(axis.text.x = element_text(angle = 90, vjust = 0.5, hjust=1))
```


Plots and GO data were written to files


```
png(paste0("GO_CC_",groupsName,".png"), width = 1224, height = 824)
dotplot(cgoCC2,showCategory = 30,
        title = paste0("GO Cellular Compartment ",groupsName))+
  theme(axis.text.x = element_text(angle = 90, vjust = 0.5, hjust=1))
dev.off()
```


```
null device 
          1
```


GO BP


```
####CC
cgoBP <- compareCluster(geneCluster = listAll, 
                      universe = AllGeneNames,
                      fun = "enrichGO",
                      OrgDb=org.Hs.eg.db,
                      keyType="SYMBOL",
                      ont = "BP", 
                      pvalueCutoff=0.05,
                      qvalueCutoff = 0.10)
cgoBP2 <- simplify(cgoBP, cutoff=0.7, by="p.adjust", select_fun=min)
####write as spreadsheet
write.csv(as.data.frame(cgoBP2),paste0("GO_BP_",groupsName,".csv"))
dotplot(cgoBP2,showCategory = 30,
        title = paste0("GO Biological Process ",groupsName))+
  theme(axis.text.x = element_text(angle = 90, vjust = 0.5, hjust=1))
```


```
png(paste0("GO_BP_",groupsName,".png"), width = 1024, height = 1224)
dotplot(cgoBP2,showCategory = 30,
        title = paste0("GO Biological Process ",groupsName))+
  theme(axis.text.x = element_text(angle = 90, vjust = 0.5, hjust=1))
dev.off()
```


```
null device 
          1
```


GO MF


```
####MF
cgoMF <- compareCluster(geneCluster = listAll, 
                      universe = AllGeneNames,
                      fun = "enrichGO",
                      OrgDb=org.Hs.eg.db, 
                      keyType="SYMBOL",
                      ont = "MF", 
                      pvalueCutoff=0.05,
                      qvalueCutoff = 0.10)
cgoMF2 <- simplify(cgoMF, cutoff=0.7, by="p.adjust", select_fun=min)
####write as spreadsheet
write.csv(as.data.frame(cgoMF2),paste0("GO_MF_",groupsName,".csv"))
dotplot(cgoMF2,showCategory = 30,
        title = paste0("GO Molecular Function  ",groupsName))+
  theme(axis.text.x = element_text(angle = 90, vjust = 0.5, hjust=1))
```


```
png(paste0("GO_MF_",groupsName,".png"), width = 1424, height = 624)
dotplot(cgoMF2,showCategory = 30,
        title = paste0("GO Molecular Function  ",groupsName))+
  theme(axis.text.x = element_text(angle = 90, vjust = 0.5, hjust=1))
dev.off()
```


```
null device 
          1
```

### R5 VAR14 vs RBC TNF k-means p0.05fc2

#### 1. Genelist Selection


```
groupsName<-"R5_Var14vRBC_TNF_kmeans_p0.05fc2"
```


```
countsTable<-read.delim("RNAseq2019July_5.txt", header = TRUE, sep = "\t",check.names=FALSE,row.names=1)
head(countsTable)
```


```
AllGeneNames<-countsTable$Gene_Symbol
#head(AllGeneNames)
```


```
grid.arrange(gTree(children=vennp), gTree(children=vennpq) , ncol=2,top="R5 Var14 TNF")
```


```
#tempA<-resAll[-c(10:30) ]
tempA<-countsTable
#rownames(tempA)
rownames(tempA) <- NULL
tempA = mutate(tempA, Include=
                   ifelse(tempA$pvalue_R5_TNF_var14_vs_RBC_0h<0.05&abs(tempA$log2FoldChange_R5_TNF_var14_vs_RBC_0h)>1&!is.na(tempA$pvalue_R5_TNF_var14_vs_RBC_0h), "in",
                          ifelse(tempA$pvalue_R5_TNF_var14_vs_RBC_2h<0.05&abs(tempA$log2FoldChange_R5_TNF_var14_vs_RBC_2h)>1&!is.na(tempA$pvalue_R5_TNF_var14_vs_RBC_2h), "in",
                                 ifelse(tempA$pvalue_R5_TNF_var14_vs_RBC_6h<0.05&abs(tempA$log2FoldChange_R5_TNF_var14_vs_RBC_6h)>1&!is.na(tempA$pvalue_R5_TNF_var14_vs_RBC_6h), "in",
                                        ifelse(tempA$pvalue_R5_TNF_var14_vs_RBC_20h<0.05&abs(tempA$log2FoldChange_R5_TNF_var14_vs_RBC_20h)>1&!is.na(tempA$pvalue_R5_TNF_var14_vs_RBC_20h), "in",
                                                                       "out")))))
#tempA
####library(dplyr)
tempA %>%
     group_by(Include) %>% 
     tally()
```


```
topDEgenes <- which(tempA$Include=="in")####find indexes
```

#### NB Please check columns used and renamed for plots


```
#baseMeansHm <-countsTable[,c(60:63)]
baseMeansHm <-countsTable[,c(110:113,129:132)]
head(baseMeansHm)
```


```
baseMeansHm2 <- log2(baseMeansHm+1)
baseMeansHm2$Var14_RBC_0h<-baseMeansHm2$Var14TNF_0h_mean-baseMeansHm2$RBC_TNF_0h_mean
baseMeansHm2$Var14_RBC_2h<-baseMeansHm2$Var14TNF_2h_mean-baseMeansHm2$RBC_TNF_2h_mean
baseMeansHm2$Var14_RBC_6h<-baseMeansHm2$Var14TNF_6h_mean-baseMeansHm2$RBC_TNF_6h_mean
baseMeansHm2$Var14_RBC_20h<-baseMeansHm2$Var14TNF_20h_mean-baseMeansHm2$RBC_TNF_20h_mean
baseMeansHm <-baseMeansHm2[,c(9:12)]
head(baseMeansHm)
```


```
baseMeansHmM <-baseMeansHm2[,c(1:8)]
head(baseMeansHmM)
```


```
topDEgenes <- which(tempA$Include=="in")####find indexes
```

#### 2. Hierachical clustering of means


```
####mean logfc
dataHMm<-baseMeansHm[ topDEgenes, ]
#dataHMm <- log2(dataHMm+1)
dataHMm<- t(as.matrix(dataHMm))
dataHMm <- t(scale(dataHMm))
hmap_hier_factors4 <- Heatmap(
  dataHMm,  name = "logfc",
  row_labels = paste0(rownames(dataHMm)," ",(tempA[ topDEgenes, ])$Gene_Symbol),
  column_title = paste0("Means logfc"), 
  col = col_funGR,
  column_title_gp = gpar(fontsize = 16, fontface = "bold"),
  width = unit(50, "mm"),
  cluster_columns = FALSE,
  show_row_names = FALSE)
dataHMmPlot<-as.data.frame(dataHMm)
dataHMmPlot$Var14_RBC_2h<-dataHMmPlot$Var14_RBC_2h-dataHMmPlot$Var14_RBC_0h
dataHMmPlot$Var14_RBC_6h<-dataHMmPlot$Var14_RBC_6h-dataHMmPlot$Var14_RBC_0h
dataHMmPlot$Var14_RBC_20h<-dataHMmPlot$Var14_RBC_20h-dataHMmPlot$Var14_RBC_0h
dataHMmPlot$Var14_RBC_0h<-dataHMmPlot$Var14_RBC_0h-dataHMmPlot$Var14_RBC_0h
dataHMmPlot<-as.matrix(dataHMmPlot)
  
hmap_hier_factors6 <- Heatmap(
  dataHMmPlot,  name = "Normalised logfc",
  row_labels = paste0(rownames(dataHMm)," ",(tempA[ topDEgenes, ])$Gene_Symbol),
  column_title = paste0("0h Normalised logfc"), 
  col = col_funGR2,
  column_title_gp = gpar(fontsize = 16, fontface = "bold"),
  width = unit(50, "mm"),
  cluster_columns = FALSE,
  show_row_names = FALSE)
####means
dataHMm3<-baseMeansHmM[ topDEgenes, ]
#dataHMm <- log2(dataHMm+1)
dataHMm3<- t(as.matrix(dataHMm3))
dataHMm3 <- t(scale(dataHMm3))
dataHMm3<-dataHMm3[,c(5,1,6,2,7,3,8,4)]
hmap_hier_factors5 <- Heatmap(
  dataHMm3,  name = "Expression",
  row_labels = paste0(rownames(dataHMm)," ",(tempA[ topDEgenes, ])$Gene_Symbol),
  column_title = paste0("Means"), 
  col = col_fun,
  column_title_gp = gpar(fontsize = 16, fontface = "bold"),
  width = unit(100, "mm"),
  cluster_columns = FALSE,
  show_row_names = FALSE)
  
hmap_hier_factors4+hmap_hier_factors6+hmap_hier_factors5
```


```
par(mfrow=c(1,2))
#### Silhouette method
fviz_nbclust(dataHMm, kmeans, method = "silhouette",k.max = 16)+
  labs(subtitle = "Silhouette method")
```


```
#### Elbow method
fviz_nbclust(dataHMm, kmeans, method = "wss",k.max = 16) +
  labs(subtitle = "Elbow method")
```


```
####gap stat slow!!!
####set.seed(123)
####fviz_nbclust(dataHMm, kmeans, nstart = 25,  method = "gap_stat", nboot = 100,k.max = 16)+
####  labs(subtitle = "Gap statistic method")
```


```
kclust8 <- kmeans(dataHMm, 6)
#silhouette plot
distK<-daisy(dataHMm)
plot(silhouette(kclust8$cluster, distK), col=1:6, border=NA)
```

#### 3. K-means clustering of means


```
split <- paste0("Cluster\n", kclust8$cluster)
#split <- factor(paste0("Cluster\n", kclust3$cluster), levels=c("Cluster\n3","Cluster\n1","Cluster\n4","Cluster\n5","Cluster\n2","Cluster\n6"))
hmap_k <- Heatmap(dataHMm, split=split, cluster_row_slices = FALSE,
                  cluster_columns = FALSE,
                  show_row_names = FALSE,
                  name = "logfc",
                  col = col_funGR,
                  width = unit(50, "mm"),
                  column_title = "means logfc", 
                  column_title_gp = gpar(fontsize = 16, fontface = "bold"))
hmap_k+hmap_hier_factors6+hmap_hier_factors5
```


Mean profiles of clusters


```
clustercount<-data.frame(kclust8$cluster)
clustersizes<-table(clustercount$kclust8.cluster)
clusterMeans<-data.frame(kclust8$centers)
clusterMeans1<-data.frame(t(clusterMeans))
clusterMeans1 <- cbind(rownames(clusterMeans1), clusterMeans1)
orderN<-c("Var14_RBC_0h","Var14_RBC_2h","Var14_RBC_6h","Var14_RBC_20h")#### manual
rownames(clusterMeans1) <- NULL
names(clusterMeans1)[names(clusterMeans1)=="rownames(clusterMeans1)"] <- "Sample"
####clusterMeans1
pX1<-ggplot(data=clusterMeans1, aes(x=Sample, y=X1,group=1)) +
  geom_line()+  geom_point()+ggtitle(paste("Cluster X1 Profile ",clustersizes[1]," genes"))+  scale_x_discrete(limits=orderN)+
  theme(axis.title.x = element_blank(),axis.title.y = element_blank())
pX2<-ggplot(data=clusterMeans1, aes(x=Sample, y=X2,group=1)) +
  geom_line()+  geom_point()+ggtitle(paste("Cluster X2 Profile ",clustersizes[2]," genes"))+  scale_x_discrete(limits=orderN)+
  theme(axis.title.x = element_blank(),axis.title.y = element_blank())
pX3<-ggplot(data=clusterMeans1, aes(x=Sample, y=X3,group=1)) +
  geom_line()+  geom_point()+ggtitle(paste("Cluster X3 Profile ",clustersizes[3]," genes"))+  scale_x_discrete(limits=orderN)+
  theme(axis.title.x = element_blank(),axis.title.y = element_blank())
pX4<-ggplot(data=clusterMeans1, aes(x=Sample, y=X4,group=1)) +
  geom_line()+  geom_point()+ggtitle(paste("Cluster X4 Profile ",clustersizes[4]," genes"))+  scale_x_discrete(limits=orderN)+
  theme(axis.title.x = element_blank(),axis.title.y = element_blank())
pX5<-ggplot(data=clusterMeans1, aes(x=Sample, y=X5,group=1)) +
  geom_line()+  geom_point()+ggtitle(paste("Cluster X5 Profile ",clustersizes[5]," genes"))+  scale_x_discrete(limits=orderN)+
  theme(axis.title.x = element_blank(),axis.title.y = element_blank())
pX6<-ggplot(data=clusterMeans1, aes(x=Sample, y=X6,group=1)) +
  geom_line()+  geom_point()+ggtitle(paste("Cluster X6 Profile ",clustersizes[6]," genes"))+  scale_x_discrete(limits=orderN)+
  theme(axis.title.x = element_blank(),axis.title.y = element_blank())
#plot
multiplot(pX1, pX2, pX3, pX4,pX5, pX6, cols=2)
```


```
topDEgenes <- which(tempA$Include=="in")####find indexes
tempAkm<-tempA[ topDEgenes, ]
SymbolsKm<-dplyr::pull(tempAkm, Gene_Symbol)
#### export the gene expression data for the clusters
write.table(clusterMeans,paste0("ClusterMeansKm_",groupsName,".txt"),  sep = "\t")
ClusteredGenes<-data.frame(kclust8$cluster,SymbolsKm,dataHMm)
write.table(ClusteredGenes,paste0("ScaledDataInClustersKm_",groupsName,".txt"),  sep = "\t")
#head(ClusteredGenes)
```


```
bottomDEgenes<-which(tempA$Include=="out")####find indexes 
bottomG<-tempA[ bottomDEgenes, ]
bottomG<-dplyr::pull(bottomG, Gene_Symbol)
write.table(bottomG,paste0("ipaBottomKmeans_",groupsName,".txt"),  sep = "\t")
                         
topDEgenes <- which(tempA$Include=="in")####find indexes 
tempAkm<-tempA[ topDEgenes, ]
SymbolsKm<-dplyr::pull(tempAkm, Gene_Symbol)
ipaKmeans<-ClusteredGenes
#countsTable <-countsTable[,c(1:15)]####if samples need removing
ipaKmeans<-ipaKmeans[,c(1:2)]
ipaKmeans$name2<-rownames(ipaKmeans)
#ipaKmeans%>% rownames_to_column(var = "rowname")
#ipaKmeans
#rowid_to_column(ipaKmeans)
ipaKmeans = mutate(ipaKmeans, x1= ifelse(ipaKmeans$kclust8.cluster==1, "1", "0"))
ipaKmeans = mutate(ipaKmeans, x2= ifelse(ipaKmeans$kclust8.cluster==2, "1", "0"))
ipaKmeans = mutate(ipaKmeans, x3= ifelse(ipaKmeans$kclust8.cluster==3, "1", "0"))
ipaKmeans = mutate(ipaKmeans, x4= ifelse(ipaKmeans$kclust8.cluster==4, "1", "0"))
ipaKmeans = mutate(ipaKmeans, x5= ifelse(ipaKmeans$kclust8.cluster==5, "1", "0"))
ipaKmeans = mutate(ipaKmeans, x6= ifelse(ipaKmeans$kclust8.cluster==6, "1", "0"))
#ipaKmeans
write.table(ipaKmeans,paste0("ipaKmeans_",groupsName,".txt"),  sep = "\t")
#head(ipaKmeans)
```


```
ClusteredGenes2<-ClusteredGenes[c(1)]
#ClusteredGenes2
listAll<-list()
for(i in 1:6) {
  clusterName<-paste0("x",i)
  #clusterName<-row.names(subset(ClusteredGenes,ClusteredGenes==i))
  clusterName<-(subset(ClusteredGenes$SymbolsKm,ClusteredGenes==i))
  listAll[[i]]<-clusterName
}
#need to name the vectors in the list, example here is for 8 clusters
names(listAll)<-c("X1", "X2", "X3", "X4","X5", "X6")
#if you want to rearrange the order
#listAll<-listAll[c("x3", "x7", "x8", "x2", "x6", "x5", "x4", "x1")]
#lapply(listAll, head)
```

#### 4. Annotation of K-means clusters

- CC cellular compartment
- BP biological process
- MF molecular function

The simplify function has been used to cut down on GO redundancy


```
#str(AllGeneNames)
```


```
####CC
cgoCC <- compareCluster(geneCluster = listAll, 
                      universe = AllGeneNames,
                      fun = "enrichGO",
                      OrgDb=org.Hs.eg.db, 
                      ####OrgDb=org.Mm.eg.db,
                      keyType="SYMBOL",
                      ont = "CC", 
                      pvalueCutoff=0.05,
                      qvalueCutoff = 0.10)
cgoCC2 <- simplify(cgoCC, cutoff=0.7, by="p.adjust", select_fun=min)
####write as spreadsheet
write.csv(as.data.frame(cgoCC2),paste0("GO_CC_",groupsName,".csv"))
dotplot(cgoCC2,showCategory = 30,
        title = paste0("GO Cellular Compartment ",groupsName))+
  theme(axis.text.x = element_text(angle = 90, vjust = 0.5, hjust=1))
```


Plots and GO data were written to files


```
png(paste0("GO_CC_",groupsName,".png"), width = 1224, height = 824)
dotplot(cgoCC2,showCategory = 30,
        title = paste0("GO Cellular Compartment ",groupsName))+
  theme(axis.text.x = element_text(angle = 90, vjust = 0.5, hjust=1))
dev.off()
```


```
null device 
          1
```


GO BP


```
####CC
cgoBP <- compareCluster(geneCluster = listAll, 
                      universe = AllGeneNames,
                      fun = "enrichGO",
                      OrgDb=org.Hs.eg.db,
                      keyType="SYMBOL",
                      ont = "BP", 
                      pvalueCutoff=0.05,
                      qvalueCutoff = 0.10)
cgoBP2 <- simplify(cgoBP, cutoff=0.7, by="p.adjust", select_fun=min)
####write as spreadsheet
write.csv(as.data.frame(cgoBP2),paste0("GO_BP_",groupsName,".csv"))
dotplot(cgoBP2,showCategory = 30,
        title = paste0("GO Biological Process ",groupsName))+
  theme(axis.text.x = element_text(angle = 90, vjust = 0.5, hjust=1))
```


```
png(paste0("GO_BP_",groupsName,".png"), width = 1024, height = 1224)
dotplot(cgoBP2,showCategory = 30,
        title = paste0("GO Biological Process ",groupsName))+
  theme(axis.text.x = element_text(angle = 90, vjust = 0.5, hjust=1))
dev.off()
```


```
null device 
          1
```


GO MF


```
####MF
cgoMF <- compareCluster(geneCluster = listAll, 
                      universe = AllGeneNames,
                      fun = "enrichGO",
                      OrgDb=org.Hs.eg.db, 
                      keyType="SYMBOL",
                      ont = "MF", 
                      pvalueCutoff=0.05,
                      qvalueCutoff = 0.10)
cgoMF2 <- simplify(cgoMF, cutoff=0.7, by="p.adjust", select_fun=min)
####write as spreadsheet
write.csv(as.data.frame(cgoMF2),paste0("GO_MF_",groupsName,".csv"))
dotplot(cgoMF2,showCategory = 30,
        title = paste0("GO Molecular Function  ",groupsName))+
  theme(axis.text.x = element_text(angle = 90, vjust = 0.5, hjust=1))
```


```
png(paste0("GO_MF_",groupsName,".png"), width = 1424, height = 824)
dotplot(cgoMF2,showCategory = 30,
        title = paste0("GO Molecular Function  ",groupsName))+
  theme(axis.text.x = element_text(angle = 90, vjust = 0.5, hjust=1))
dev.off()
```


```
null device 
          1
```

### R5 VAR14 vs RBC TNF k-means padj0.1fc2

#### 1. Genelist Selection


```
groupsName<-"R5_Var14vRBC_TNF_kmeans_padj0.1fc2"
```


```
countsTable<-read.delim("RNAseq2019July_5.txt", header = TRUE, sep = "\t",check.names=FALSE,row.names=1)
head(countsTable)
```


```
AllGeneNames<-countsTable$Gene_Symbol
#head(AllGeneNames)
```


```
grid.arrange(gTree(children=vennq2), gTree(children=vennpq) , ncol=2,top="R5 Var14 TNF")
```


```
#tempA<-resAll[-c(10:30) ]
tempA<-countsTable
#rownames(tempA)
rownames(tempA) <- NULL
tempA = mutate(tempA, Include=
                   ifelse(tempA$padj_R5_TNF_var14_vs_RBC_0h<0.1&abs(tempA$log2FoldChange_R5_TNF_var14_vs_RBC_0h)>1&!is.na(tempA$padj_R5_TNF_var14_vs_RBC_0h), "in",
                          ifelse(tempA$padj_R5_TNF_var14_vs_RBC_2h<0.1&abs(tempA$log2FoldChange_R5_TNF_var14_vs_RBC_2h)>1&!is.na(tempA$padj_R5_TNF_var14_vs_RBC_2h), "in",
                                 ifelse(tempA$padj_R5_TNF_var14_vs_RBC_6h<0.1&abs(tempA$log2FoldChange_R5_TNF_var14_vs_RBC_6h)>1&!is.na(tempA$padj_R5_TNF_var14_vs_RBC_6h), "in",
                                        ifelse(tempA$padj_R5_TNF_var14_vs_RBC_20h<0.1&abs(tempA$log2FoldChange_R5_TNF_var14_vs_RBC_20h)>1&!is.na(tempA$padj_R5_TNF_var14_vs_RBC_20h), "in",
                                                                       "out")))))
#tempA
####library(dplyr)
tempA %>%
     group_by(Include) %>% 
     tally()
```


```
topDEgenes <- which(tempA$Include=="in")####find indexes
```

#### NB Please check columns used and renamed for plots


```
#baseMeansHm <-countsTable[,c(60:63)]
baseMeansHm <-countsTable[,c(110:113,129:132)]
head(baseMeansHm)
```


```
baseMeansHm2 <- log2(baseMeansHm+1)
baseMeansHm2$Var14_RBC_0h<-baseMeansHm2$Var14TNF_0h_mean-baseMeansHm2$RBC_TNF_0h_mean
baseMeansHm2$Var14_RBC_2h<-baseMeansHm2$Var14TNF_2h_mean-baseMeansHm2$RBC_TNF_2h_mean
baseMeansHm2$Var14_RBC_6h<-baseMeansHm2$Var14TNF_6h_mean-baseMeansHm2$RBC_TNF_6h_mean
baseMeansHm2$Var14_RBC_20h<-baseMeansHm2$Var14TNF_20h_mean-baseMeansHm2$RBC_TNF_20h_mean
baseMeansHm <-baseMeansHm2[,c(9:12)]
head(baseMeansHm)
```


```
baseMeansHmM <-baseMeansHm2[,c(1:8)]
head(baseMeansHmM)
```


```
topDEgenes <- which(tempA$Include=="in")####find indexes
```

#### 2. Hierachical clustering of means


```
####mean logfc
dataHMm<-baseMeansHm[ topDEgenes, ]
#dataHMm <- log2(dataHMm+1)
dataHMm<- t(as.matrix(dataHMm))
dataHMm <- t(scale(dataHMm))
hmap_hier_factors4 <- Heatmap(
  dataHMm,  name = "logfc",
  row_labels = paste0(rownames(dataHMm)," ",(tempA[ topDEgenes, ])$Gene_Symbol),
  column_title = paste0("Means logfc"), 
  col = col_funGR,
  column_title_gp = gpar(fontsize = 16, fontface = "bold"),
  width = unit(50, "mm"),
  cluster_columns = FALSE,
  show_row_names = FALSE)
dataHMmPlot<-as.data.frame(dataHMm)
dataHMmPlot$Var14_RBC_2h<-dataHMmPlot$Var14_RBC_2h-dataHMmPlot$Var14_RBC_0h
dataHMmPlot$Var14_RBC_6h<-dataHMmPlot$Var14_RBC_6h-dataHMmPlot$Var14_RBC_0h
dataHMmPlot$Var14_RBC_20h<-dataHMmPlot$Var14_RBC_20h-dataHMmPlot$Var14_RBC_0h
dataHMmPlot$Var14_RBC_0h<-dataHMmPlot$Var14_RBC_0h-dataHMmPlot$Var14_RBC_0h
dataHMmPlot<-as.matrix(dataHMmPlot)
  
hmap_hier_factors6 <- Heatmap(
  dataHMmPlot,  name = "Normalised logfc",
  row_labels = paste0(rownames(dataHMm)," ",(tempA[ topDEgenes, ])$Gene_Symbol),
  column_title = paste0("0h Normalised logfc"), 
  col = col_funGR2,
  column_title_gp = gpar(fontsize = 16, fontface = "bold"),
  width = unit(50, "mm"),
  cluster_columns = FALSE,
  show_row_names = FALSE)
####means
dataHMm3<-baseMeansHmM[ topDEgenes, ]
#dataHMm <- log2(dataHMm+1)
dataHMm3<- t(as.matrix(dataHMm3))
dataHMm3 <- t(scale(dataHMm3))
dataHMm3<-dataHMm3[,c(5,1,6,2,7,3,8,4)]
hmap_hier_factors5 <- Heatmap(
  dataHMm3,  name = "Expression",
  row_labels = paste0(rownames(dataHMm)," ",(tempA[ topDEgenes, ])$Gene_Symbol),
  column_title = paste0("Means"), 
  col = col_fun,
  column_title_gp = gpar(fontsize = 16, fontface = "bold"),
  width = unit(100, "mm"),
  cluster_columns = FALSE,
  show_row_names = FALSE)
  
hmap_hier_factors4+hmap_hier_factors6+hmap_hier_factors5
```


```
par(mfrow=c(1,2))
#### Silhouette method
fviz_nbclust(dataHMm, kmeans, method = "silhouette",k.max = 16)+
  labs(subtitle = "Silhouette method")
```


```
#### Elbow method
fviz_nbclust(dataHMm, kmeans, method = "wss",k.max = 16) +
  labs(subtitle = "Elbow method")
```


```
####gap stat slow!!!
####set.seed(123)
####fviz_nbclust(dataHMm, kmeans, nstart = 25,  method = "gap_stat", nboot = 100,k.max = 16)+
####  labs(subtitle = "Gap statistic method")
```


```
kclust8b <- kmeans(dataHMm, 6)
#silhouette plot
distK<-daisy(dataHMm)
plot(silhouette(kclust8b$cluster, distK), col=1:6, border=NA)
```

#### 3. K-means clustering of means


```
split <- paste0("Cluster\n", kclust8b$cluster)
#split <- factor(paste0("Cluster\n", kclust3$cluster), levels=c("Cluster\n3","Cluster\n1","Cluster\n4","Cluster\n5","Cluster\n2","Cluster\n6"))
hmap_k <- Heatmap(dataHMm, split=split, cluster_row_slices = FALSE,
                  cluster_columns = FALSE,
                  show_row_names = FALSE,
                  name = "logfc",
                  col = col_funGR,
                  width = unit(50, "mm"),
                  column_title = "means logfc", 
                  column_title_gp = gpar(fontsize = 16, fontface = "bold"))
hmap_k+hmap_hier_factors6+hmap_hier_factors5
```


Mean profiles of clusters


```
clustercount<-data.frame(kclust8b$cluster)
clustersizes<-table(clustercount$kclust8b.cluster)
clusterMeans<-data.frame(kclust8b$centers)
clusterMeans1<-data.frame(t(clusterMeans))
clusterMeans1 <- cbind(rownames(clusterMeans1), clusterMeans1)
orderN<-c("Var14_RBC_0h","Var14_RBC_2h","Var14_RBC_6h","Var14_RBC_20h")#### manual
rownames(clusterMeans1) <- NULL
names(clusterMeans1)[names(clusterMeans1)=="rownames(clusterMeans1)"] <- "Sample"
####clusterMeans1
pX1<-ggplot(data=clusterMeans1, aes(x=Sample, y=X1,group=1)) +
  geom_line()+  geom_point()+ggtitle(paste("Cluster X1 Profile ",clustersizes[1]," genes"))+  scale_x_discrete(limits=orderN)+
  theme(axis.title.x = element_blank(),axis.title.y = element_blank())
pX2<-ggplot(data=clusterMeans1, aes(x=Sample, y=X2,group=1)) +
  geom_line()+  geom_point()+ggtitle(paste("Cluster X2 Profile ",clustersizes[2]," genes"))+  scale_x_discrete(limits=orderN)+
  theme(axis.title.x = element_blank(),axis.title.y = element_blank())
pX3<-ggplot(data=clusterMeans1, aes(x=Sample, y=X3,group=1)) +
  geom_line()+  geom_point()+ggtitle(paste("Cluster X3 Profile ",clustersizes[3]," genes"))+  scale_x_discrete(limits=orderN)+
  theme(axis.title.x = element_blank(),axis.title.y = element_blank())
pX4<-ggplot(data=clusterMeans1, aes(x=Sample, y=X4,group=1)) +
  geom_line()+  geom_point()+ggtitle(paste("Cluster X4 Profile ",clustersizes[4]," genes"))+  scale_x_discrete(limits=orderN)+
  theme(axis.title.x = element_blank(),axis.title.y = element_blank())
pX5<-ggplot(data=clusterMeans1, aes(x=Sample, y=X5,group=1)) +
  geom_line()+  geom_point()+ggtitle(paste("Cluster X5 Profile ",clustersizes[5]," genes"))+  scale_x_discrete(limits=orderN)+
  theme(axis.title.x = element_blank(),axis.title.y = element_blank())
pX6<-ggplot(data=clusterMeans1, aes(x=Sample, y=X6,group=1)) +
  geom_line()+  geom_point()+ggtitle(paste("Cluster X6 Profile ",clustersizes[6]," genes"))+  scale_x_discrete(limits=orderN)+
  theme(axis.title.x = element_blank(),axis.title.y = element_blank())
#plot
multiplot(pX1, pX2, pX3, pX4,pX5, pX6, cols=2)
```


```
topDEgenes <- which(tempA$Include=="in")####find indexes
tempAkm<-tempA[ topDEgenes, ]
SymbolsKm<-dplyr::pull(tempAkm, Gene_Symbol)
#### export the gene expression data for the clusters
write.table(clusterMeans,paste0("ClusterMeansKm_",groupsName,".txt"),  sep = "\t")
ClusteredGenes<-data.frame(kclust8b$cluster,SymbolsKm,dataHMm)
write.table(ClusteredGenes,paste0("ScaledDataInClustersKm_",groupsName,".txt"),  sep = "\t")
#head(ClusteredGenes)
```


```
bottomDEgenes<-which(tempA$Include=="out")####find indexes 
bottomG<-tempA[ bottomDEgenes, ]
bottomG<-dplyr::pull(bottomG, Gene_Symbol)
write.table(bottomG,paste0("ipaBottomKmeans_",groupsName,".txt"),  sep = "\t")
                         
topDEgenes <- which(tempA$Include=="in")####find indexes 
tempAkm<-tempA[ topDEgenes, ]
SymbolsKm<-dplyr::pull(tempAkm, Gene_Symbol)
ipaKmeans<-ClusteredGenes
#countsTable <-countsTable[,c(1:15)]####if samples need removing
ipaKmeans<-ipaKmeans[,c(1:2)]
ipaKmeans$name2<-rownames(ipaKmeans)
#ipaKmeans%>% rownames_to_column(var = "rowname")
#ipaKmeans
#rowid_to_column(ipaKmeans)
ipaKmeans = mutate(ipaKmeans, x1= ifelse(ipaKmeans$kclust8b.cluster==1, "1", "0"))
ipaKmeans = mutate(ipaKmeans, x2= ifelse(ipaKmeans$kclust8b.cluster==2, "1", "0"))
ipaKmeans = mutate(ipaKmeans, x3= ifelse(ipaKmeans$kclust8b.cluster==3, "1", "0"))
ipaKmeans = mutate(ipaKmeans, x4= ifelse(ipaKmeans$kclust8b.cluster==4, "1", "0"))
ipaKmeans = mutate(ipaKmeans, x5= ifelse(ipaKmeans$kclust8b.cluster==5, "1", "0"))
ipaKmeans = mutate(ipaKmeans, x6= ifelse(ipaKmeans$kclust8b.cluster==6, "1", "0"))
#ipaKmeans
write.table(ipaKmeans,paste0("ipaKmeans_",groupsName,".txt"),  sep = "\t")
#head(ipaKmeans)
```


```
ClusteredGenes2<-ClusteredGenes[c(1)]
#ClusteredGenes2
listAll<-list()
for(i in 1:6) {
  clusterName<-paste0("x",i)
  #clusterName<-row.names(subset(ClusteredGenes,ClusteredGenes==i))
  clusterName<-(subset(ClusteredGenes$SymbolsKm,ClusteredGenes==i))
  listAll[[i]]<-clusterName
}
#need to name the vectors in the list, example here is for 8 clusters
names(listAll)<-c("X1", "X2", "X3", "X4","X5", "X6")
#if you want to rearrange the order
#listAll<-listAll[c("x3", "x7", "x8", "x2", "x6", "x5", "x4", "x1")]
#lapply(listAll, head)
```

#### 4. Annotation of K-means clusters

- CC cellular compartment
- BP biological process
- MF molecular function

The simplify function has been used to cut down on GO redundancy


```
#str(AllGeneNames)
```


```
####CC
cgoCC <- compareCluster(geneCluster = listAll, 
                      universe = AllGeneNames,
                      fun = "enrichGO",
                      OrgDb=org.Hs.eg.db, 
                      ####OrgDb=org.Mm.eg.db,
                      keyType="SYMBOL",
                      ont = "CC", 
                      pvalueCutoff=0.05,
                      qvalueCutoff = 0.10)
cgoCC2 <- simplify(cgoCC, cutoff=0.7, by="p.adjust", select_fun=min)
####write as spreadsheet
write.csv(as.data.frame(cgoCC2),paste0("GO_CC_",groupsName,".csv"))
dotplot(cgoCC2,showCategory = 30,
        title = paste0("GO Cellular Compartment ",groupsName))+
  theme(axis.text.x = element_text(angle = 90, vjust = 0.5, hjust=1))
```


Plots and GO data were written to files


```
png(paste0("GO_CC_",groupsName,".png"), width = 1224, height = 824)
dotplot(cgoCC2,showCategory = 30,
        title = paste0("GO Cellular Compartment ",groupsName))+
  theme(axis.text.x = element_text(angle = 90, vjust = 0.5, hjust=1))
dev.off()
```


```
null device 
          1
```


GO BP


```
####CC
cgoBP <- compareCluster(geneCluster = listAll, 
                      universe = AllGeneNames,
                      fun = "enrichGO",
                      OrgDb=org.Hs.eg.db,
                      keyType="SYMBOL",
                      ont = "BP", 
                      pvalueCutoff=0.05,
                      qvalueCutoff = 0.10)
cgoBP2 <- simplify(cgoBP, cutoff=0.7, by="p.adjust", select_fun=min)
####write as spreadsheet
write.csv(as.data.frame(cgoBP2),paste0("GO_BP_",groupsName,".csv"))
dotplot(cgoBP2,showCategory = 30,
        title = paste0("GO Biological Process ",groupsName))+
  theme(axis.text.x = element_text(angle = 90, vjust = 0.5, hjust=1))
```


```
png(paste0("GO_BP_",groupsName,".png"), width = 1024, height = 1224)
dotplot(cgoBP2,showCategory = 30,
        title = paste0("GO Biological Process ",groupsName))+
  theme(axis.text.x = element_text(angle = 90, vjust = 0.5, hjust=1))
dev.off()
```


```
null device 
          1
```


GO MF


```
####MF
cgoMF <- compareCluster(geneCluster = listAll, 
                      universe = AllGeneNames,
                      fun = "enrichGO",
                      OrgDb=org.Hs.eg.db, 
                      keyType="SYMBOL",
                      ont = "MF", 
                      pvalueCutoff=0.05,
                      qvalueCutoff = 0.10)
cgoMF2 <- simplify(cgoMF, cutoff=0.7, by="p.adjust", select_fun=min)
####write as spreadsheet
write.csv(as.data.frame(cgoMF2),paste0("GO_MF_",groupsName,".csv"))
dotplot(cgoMF2,showCategory = 30,
        title = paste0("GO Molecular Function  ",groupsName))+
  theme(axis.text.x = element_text(angle = 90, vjust = 0.5, hjust=1))
```


```
png(paste0("GO_MF_",groupsName,".png"), width = 1424, height = 824)
dotplot(cgoMF2,showCategory = 30,
        title = paste0("GO Molecular Function  ",groupsName))+
  theme(axis.text.x = element_text(angle = 90, vjust = 0.5, hjust=1))
dev.off()
```


```
null device 
          1
```


save: once happy with clustering save workspace so that it can be recalled


```
save.image(file="KmDecember.RData")
```


Add a new chunk by clicking the *Insert Chunk* button on the toolbar or by pressing *Ctrl+Alt+I*.

When you save the notebook, an HTML file containing the code and output will be saved alongside it (click the *Preview* button or press *Ctrl+Shift+K* to preview the HTML file).

The preview shows you a rendered HTML copy of the contents of the editor. Consequently, unlike *Knit*, *Preview* does not run any R code chunks. Instead, the output of the chunk when it was last run in the editor is displayed.

LS0tCnRpdGxlOiAiS0NvdXBlci9MaXZlcnBvb2wgSy1tZWFucyBSTkFzZXEgQW5hbHlzaXMgTm92ZW1iZXIgMjAyMCIKYXV0aG9yOiBMZW8gWmVlZgp0aGVtZTogdW5pdGVkCm91dHB1dDogCiBodG1sX25vdGVib29rOgogdG9jOiB0cnVlCiB0b2NfZGVwdGg6IDIKIHRvY19mbG9hdDogdHJ1ZQogY29sbGFwc2VkOiBmYWxzZQogc21vb3RoX3Njcm9sbDogZmFsc2UKIGNvZGVfZm9sZGluZzogImhpZGUiCi0tLQojIyBBbmFseXNpcyBTZWN0aW9ucyB7LnRhYnNldCAudGFic2V0LXBpbGxzfQpWaWV3aW5nIGlzIGJldHRlciBpZiBDb2RlIGlzIGhpZGRlbiAoVG9wIFJpZ2h0IGRyb3AgZG93biBsaXN0KQoKCmBgYHtyfQpzaW5rKGZpbGU9IlJzZXNzaW9uSW5mb0RFU2VxMi50eHQiKQpsaWJyYXJ5KCdERVNlcTInKQpsaWJyYXJ5KCJnZ3Bsb3QyIikKbGlicmFyeShyZXNoYXBlMikKIyMjI2xpYnJhcnkodGlkeXZlcnNlKQojIyMjbGlicmFyeShzcGxpdHN0YWNrc2hhcGUpCiMjIyNsaWJyYXJ5KGRhdGEudGFibGUpCmxpYnJhcnkoIlJDb2xvckJyZXdlciIpCmxpYnJhcnkoImdwbG90cyIpCiMjIyNsaWJyYXJ5KCdnZ2RlbmRybycpCmxpYnJhcnkoJ2dncmVwZWwnKQpsaWJyYXJ5KCJkcGx5ciIpCmxpYnJhcnkoIkNvbXBsZXhIZWF0bWFwIikKbGlicmFyeSgiY2x1c3RlclByb2ZpbGVyIikKbGlicmFyeShWZW5uRGlhZ3JhbSkgIyMjIyMjCmxpYnJhcnkoVXBTZXRSKQpsaWJyYXJ5KGdyaWRFeHRyYSkKbGlicmFyeShjbHVzdGVyKQpsaWJyYXJ5KGNpcmNsaXplKQpsaWJyYXJ5KGZhY3RvZXh0cmEpCmxpYnJhcnkoTmJDbHVzdCkKbGlicmFyeSgiYmlvbWFSdCIpCmxpYnJhcnkoIm9yZy5Icy5lZy5kYiIpIyMjI2h1bWFuCmxpYnJhcnkoIm9yZy5NbS5lZy5kYiIpIyMjI21vdXNlCmxpYnJhcnkodmVubikKIyMjI2xpYnJhcnkob3JnLkF0LnRhaXIuZGIpIyMjI2FyYWJpZG9wc2lzCnNlc3Npb25JbmZvKCkKc2luaygpCgojIyMjIyMjIyMjIyMjIyMjIyMjIyMjIyMjIyMjIyMjIyMjIyMjIyMjIwojIyMjbXVsdGlwbG90CiMjIyMjIyMjIyMjIyMjIyMjIyMjIyMjIyMjIyMjIyMjIyMjIyMjIyMjCiMjIyMgTXVsdGlwbGUgcGxvdCBmdW5jdGlvbgojIyMjCiMjIyMgZ2dwbG90IG9iamVjdHMgY2FuIGJlIHBhc3NlZCBpbiAuLi4sIG9yIHRvIHBsb3RsaXN0IChhcyBhIGxpc3Qgb2YgZ2dwbG90IG9iamVjdHMpCiMjIyMgLSBjb2xzOiAgIE51bWJlciBvZiBjb2x1bW5zIGluIGxheW91dAojIyMjIC0gbGF5b3V0OiBBIG1hdHJpeCBzcGVjaWZ5aW5nIHRoZSBsYXlvdXQuIElmIHByZXNlbnQsICdjb2xzJyBpcyBpZ25vcmVkLgoKIyMjIyBJZiB0aGUgbGF5b3V0IGlzIHNvbWV0aGluZyBsaWtlIG1hdHJpeChjKDEsMiwzLDMpLCBucm93PTIsIGJ5cm93PVRSVUUpLAojIyMjIHRoZW4gcGxvdCAxIHdpbGwgZ28gaW4gdGhlIHVwcGVyIGxlZnQsIDIgd2lsbCBnbyBpbiB0aGUgdXBwZXIgcmlnaHQsIGFuZAojIyMjIDMgd2lsbCBnbyBhbGwgdGhlIHdheSBhY3Jvc3MgdGhlIGJvdHRvbS4KCm11bHRpcGxvdCA8LSBmdW5jdGlvbiguLi4sIHBsb3RsaXN0PU5VTEwsIGZpbGUsIGNvbHM9MSwgbGF5b3V0PU5VTEwpIHsKICBsaWJyYXJ5KGdyaWQpCiAgCiAgIyMjIyBNYWtlIGEgbGlzdCBmcm9tIHRoZSAuLi4gYXJndW1lbnRzIGFuZCBwbG90bGlzdAogIHBsb3RzIDwtIGMobGlzdCguLi4pLCBwbG90bGlzdCkKICAKICBudW1QbG90cyA9IGxlbmd0aChwbG90cykKICAKICAjIyMjIElmIGxheW91dCBpcyBOVUxMLCB0aGVuIHVzZSAnY29scycgdG8gZGV0ZXJtaW5lIGxheW91dAogIGlmIChpcy5udWxsKGxheW91dCkpIHsKICAgICMjIyMgTWFrZSB0aGUgcGFuZWwKICAgICMjIyMgbmNvbDogTnVtYmVyIG9mIGNvbHVtbnMgb2YgcGxvdHMKICAgICMjIyMgbnJvdzogTnVtYmVyIG9mIHJvd3MgbmVlZGVkLCBjYWxjdWxhdGVkIGZyb20gIyMjIyBvZiBjb2xzCiAgICBsYXlvdXQgPC0gbWF0cml4KHNlcSgxLCBjb2xzICogY2VpbGluZyhudW1QbG90cy9jb2xzKSksCiAgICAgICAgICAgICAgICAgICAgIG5jb2wgPSBjb2xzLCBucm93ID0gY2VpbGluZyhudW1QbG90cy9jb2xzKSkKICB9CiAgCiAgaWYgKG51bVBsb3RzPT0xKSB7CiAgICBwcmludChwbG90c1tbMV1dKQogICAgCiAgfSBlbHNlIHsKICAgICMjIyMgU2V0IHVwIHRoZSBwYWdlCiAgICBncmlkLm5ld3BhZ2UoKQogICAgcHVzaFZpZXdwb3J0KHZpZXdwb3J0KGxheW91dCA9IGdyaWQubGF5b3V0KG5yb3cobGF5b3V0KSwgbmNvbChsYXlvdXQpKSkpCiAgICAKICAgICMjIyMgTWFrZSBlYWNoIHBsb3QsIGluIHRoZSBjb3JyZWN0IGxvY2F0aW9uCiAgICBmb3IgKGkgaW4gMTpudW1QbG90cykgewogICAgICAjIyMjIEdldCB0aGUgaSxqIG1hdHJpeCBwb3NpdGlvbnMgb2YgdGhlIHJlZ2lvbnMgdGhhdCBjb250YWluIHRoaXMgc3VicGxvdAogICAgICBtYXRjaGlkeCA8LSBhcy5kYXRhLmZyYW1lKHdoaWNoKGxheW91dCA9PSBpLCBhcnIuaW5kID0gVFJVRSkpCiAgICAgIAogICAgICBwcmludChwbG90c1tbaV1dLCB2cCA9IHZpZXdwb3J0KGxheW91dC5wb3Mucm93ID0gbWF0Y2hpZHgkcm93LAogICAgICAgICAgICAgICAgICAgICAgICAgICAgICAgICAgICAgIGxheW91dC5wb3MuY29sID0gbWF0Y2hpZHgkY29sKSkKICAgIH0KICB9Cn0KIyMjI2Z1bmN0aW9uIG15IGNvZGUgZWRpdCBvZiBwbG90UENBCiMjIyMjIyMjIyMjIyMjIyMjIyMjIyMjIyMjIyMjIyMjIyMjIwpwbG90UENBTGVvPC1mdW5jdGlvbiAoeCwgaW50Z3JvdXAgPSAiVHJlYXRtZW50IiwgbnRvcCA9IDUwMCwgcmV0dXJuRGF0YSA9IEZBTFNFLCBQQ3g9MSwgUEN5PTIpCnsKICAjIyMjcnYgPC0gcm93VmFycyhhc3NheSh4KSkKICBydiA9IGFwcGx5KChhc3NheSh4KSksIDEsIHZhcikKICBzZWxlY3QgPC0gb3JkZXIocnYsIGRlY3JlYXNpbmcgPSBUUlVFKVtzZXFfbGVuKG1pbihudG9wLCAKICAgICAgICAgICAgICAgICAgICAgICAgICAgICAgICAgICAgICAgICAgICAgICAgICAgICBsZW5ndGgocnYpKSldCiAgcGNhIDwtIHByY29tcCh0KGFzc2F5KHgpW3NlbGVjdCwgXSkpCiAgcGVyY2VudFZhciA8LSBwY2Ekc2Rldl4yL3N1bShwY2Ekc2Rldl4yKQogIGlmICghYWxsKGludGdyb3VwICVpbiUgbmFtZXMoY29sRGF0YSh4KSkpKSB7CiAgICBzdG9wKCJ0aGUgYXJndW1lbnQgJ2ludGdyb3VwJyBzaG91bGQgc3BlY2lmeSBjb2x1bW5zIG9mIGNvbERhdGEoZGRzKSIpCiAgfQogIGludGdyb3VwLmRmIDwtIGFzLmRhdGEuZnJhbWUoY29sRGF0YSh4KVssIGludGdyb3VwLCBkcm9wID0gRkFMU0VdKQogIGdyb3VwIDwtIGZhY3RvcihhcHBseShpbnRncm91cC5kZiwgMSwgcGFzdGUsIGNvbGxhcHNlID0gIiA6ICIpKQogIGQgPC0gZGF0YS5mcmFtZShQQ1ggPSBwY2EkeFssIFBDeF0sIFBDWSA9IHBjYSR4WywgUEN5XSwgZ3JvdXAgPSBncm91cCwgCiAgICAgICAgICAgICAgICAgIGludGdyb3VwLmRmLCBuYW1lcyA9IGNvbG5hbWVzKHgpKQogIGlmIChyZXR1cm5EYXRhKSB7CiAgICBhdHRyKGQsICJwZXJjZW50VmFyIikgPC0gcGVyY2VudFZhcltQQ3g6UEN5XQogICAgcmV0dXJuKGQpCiAgfQogIGdncGxvdChkYXRhID0gZCwgYWVzX3N0cmluZyh4ID0gIlBDWCIsIHkgPSAiUENZIiwgY29sb3IgPSAiZ3JvdXAiKSkgKyAKICAgICMjIyNnZ3Bsb3QoZGF0YSA9IGQsIGFlc19zdHJpbmcoeCA9ICJQQ1giLCB5ID0gIlBDWSIsIGNvbG9yPVRnZmIxLCBzaGFwZT1UcmVhdG1lbnQpKSArIAogICAgZ2VvbV9wb2ludChzaXplID0gMykgKyB4bGFiKHBhc3RlMCgiUEMiLFBDeCwiOiAiLCByb3VuZChwZXJjZW50VmFyWzFdICogCiAgICAgICAgICAgICAgICAgICAgICAgICAgICAgICAgICAgICAgICAgICAgICAgICAgICAgICAgICAgICAgMTAwKSwgIiUgdmFyaWFuY2UiKSkgKyB5bGFiKHBhc3RlMCgiUEMiLFBDeSwiOiAiLCByb3VuZChwZXJjZW50VmFyWzJdICogCiAgICAgICAgICAgICAgICAgICAgICAgICAgICAgICAgICAgICAgICAgICAgICAgICAgICAgICAgICAgICAgICAgICAgICAgICAgICAgICAgICAgICAgICAgICAgICAgICAgICAgICAgICAgICAgICAgICAgICAgIDEwMCksICIlIHZhcmlhbmNlIikpCn0KYGBgCgoKYGBge3J9CmNvbF9mdW4gPSBjb2xvclJhbXAyKGMoLTEsLTAuMiwgMCwwLjIsIDEpLCBjKCJibHVlIiwiY3lhbiIsICJncmV5OTAiLCJvcmFuZ2UiLCAicmVkIikpI2hlYXRtYXAgY29sb3Vycwpjb2xfZnVuR1IgPSBjb2xvclJhbXAyKGMoLTEuNSwgMCwgMS41KSwgYygiZ3JlZW4iLCAiYmxhY2siLCAicmVkIikpCmNvbF9mdW5HUjIgPSBjb2xvclJhbXAyKGMoLTIsIDAsIDIpLCBjKCJncmVlbiIsICJibGFjayIsICJyZWQiKSkKY29sb3JzVjMgPC0gYygiY29ybmZsb3dlcmJsdWUiLCAgImJyb3duMSIsIm9yYW5nZTIiKSNWZW5uIGNvbG91cnMKY29sb3JzVjIgPC0gYygibWVkaXVtb3JjaGlkMSIsICAiY2hhcnRyZXVzZTMiKSNWZW5uIGNvbG91cnMKY29sb3JzVjQ8LWMoImNvcm5mbG93ZXJibHVlIiwgIm9yYW5nZTIiLCAiZ3JlZW4zIiwicmVkIikjVmVubiBjb2xvdXJzCmNvbG9yc1Y1PC1jKCJjb3JuZmxvd2VyYmx1ZSIsICJvcmFuZ2UyIiwgImdyZWVuMyIsInB1cnBsZSIsInJlZCIpI1Zlbm4gY29sb3VycwojY29sX2Z1bihzZXEoLTMsIDMpKQpgYGAKCgoKIyMjIFIzIFZBUjE0IHZzIFJCQyBubyBUTkYgay1tZWFucyBxMC4wNQoKIyMjIzEuIEdlbmVsaXN0IFNlbGVjdGlvbgoKYGBge3J9Cmdyb3Vwc05hbWU8LSJSM19WQVIxNF9rbWVhbnNfcTAuMDUiCmBgYAoKCmBgYHtyfQpjb3VudHNUYWJsZTwtcmVhZC5kZWxpbSgiUk5Bc2VxMjAxOUp1bHlfNS50eHQiLCBoZWFkZXIgPSBUUlVFLCBzZXAgPSAiXHQiLGNoZWNrLm5hbWVzPUZBTFNFLHJvdy5uYW1lcz0xKQpoZWFkKGNvdW50c1RhYmxlKQpgYGAKCmBgYHtyfQpBbGxHZW5lTmFtZXM8LWNvdW50c1RhYmxlJEdlbmVfU3ltYm9sCiNoZWFkKEFsbEdlbmVOYW1lcykKYGBgCgpgYGB7cn0KdGVtcEE8LWNvdW50c1RhYmxlCmBgYAoKYGBge3J9Cgp0b3BERWdlbmVzIDwtIHdoaWNoKHRlbXBBJHBhZGpfUjNub1RORl92YXIxNF92c19SQkNfMGg8MC4wNSYhaXMubmEodGVtcEEkcGFkal9SM25vVE5GX3ZhcjE0X3ZzX1JCQ18waCkpIyMjI2ZpbmQgaW5kZXhlcyAKbGlzdEE8LXRlbXBBWyB0b3BERWdlbmVzLCBdJEdlbmVfU3ltYm9sCnRvcERFZ2VuZXMgPC0gd2hpY2godGVtcEEkcGFkal9SM25vVE5GX3ZhcjE0X3ZzX1JCQ18yaDwwLjA1JiFpcy5uYSh0ZW1wQSRwYWRqX1Izbm9UTkZfdmFyMTRfdnNfUkJDXzJoKSkjIyMjZmluZCBpbmRleGVzIApsaXN0QjwtdGVtcEFbIHRvcERFZ2VuZXMsIF0kR2VuZV9TeW1ib2wKdG9wREVnZW5lcyA8LSB3aGljaCh0ZW1wQSRwYWRqX1Izbm9UTkZfdmFyMTRfdnNfUkJDXzZoPDAuMDUmIWlzLm5hKHRlbXBBJHBhZGpfUjNub1RORl92YXIxNF92c19SQkNfNmgpKSMjIyNmaW5kIGluZGV4ZXMgCmxpc3RDPC10ZW1wQVsgdG9wREVnZW5lcywgXSRHZW5lX1N5bWJvbAp0b3BERWdlbmVzIDwtIHdoaWNoKHRlbXBBJHBhZGpfUjNub1RORl92YXIxNF92c19SQkNfMjBoPDAuMDUmIWlzLm5hKHRlbXBBJHBhZGpfUjNub1RORl92YXIxNF92c19SQkNfMjBoKSkjIyMjZmluZCBpbmRleGVzIApsaXN0RDwtdGVtcEFbIHRvcERFZ2VuZXMsIF0kR2VuZV9TeW1ib2wKCgoKdmVubnE8LXZlbm4uZGlhZ3JhbSh4ID0gbGlzdChsaXN0QSxsaXN0QixsaXN0QyxsaXN0RCkgLAogICAgICAgICAgICBjYXRlZ29yeS5uYW1lcyA9IGMoIlZhcjE0bm9UTkZfMGgiLCJWYXIxNG5vVE5GXzJoIiwiVmFyMTRub1RORl82aCIsIlZhcjE0bm9UTkZfMjBoIiksCiAgICAgICAgICAgIG1haW49InBhZGo8MC4wNSIsCiAgICAgICAgICAgIGZpbGVuYW1lID0gTlVMTCwgIHNjYWxlZCA9IEZBTFNFLCBmaWxsID0gY29sb3JzVjQsIGNhdC5jb2wgPSBjb2xvcnNWNCwgY2F0LmNleCA9IDEsIGNhdC5kaXN0PTAuMywgIG1hcmdpbiA9IDAuMykKCnRvcERFZ2VuZXMgPC0gd2hpY2godGVtcEEkcHZhbHVlX1Izbm9UTkZfdmFyMTRfdnNfUkJDXzBoPDAuMDUmYWJzKHRlbXBBJGxvZzJGb2xkQ2hhbmdlX1Izbm9UTkZfdmFyMTRfdnNfUkJDXzBoKT4xJiFpcy5uYSh0ZW1wQSRwdmFsdWVfUjNub1RORl92YXIxNF92c19SQkNfMGgpKSMjIyNmaW5kIGluZGV4ZXMgCmxpc3RBPC10ZW1wQVsgdG9wREVnZW5lcywgXSRHZW5lX1N5bWJvbAp0b3BERWdlbmVzIDwtIHdoaWNoKHRlbXBBJHB2YWx1ZV9SM25vVE5GX3ZhcjE0X3ZzX1JCQ18yaDwwLjA1JmFicyh0ZW1wQSRsb2cyRm9sZENoYW5nZV9SM25vVE5GX3ZhcjE0X3ZzX1JCQ18yaCk+MSYhaXMubmEodGVtcEEkcHZhbHVlX1Izbm9UTkZfdmFyMTRfdnNfUkJDXzJoKSkjIyMjZmluZCBpbmRleGVzIApsaXN0QjwtdGVtcEFbIHRvcERFZ2VuZXMsIF0kR2VuZV9TeW1ib2wKdG9wREVnZW5lcyA8LSB3aGljaCh0ZW1wQSRwdmFsdWVfUjNub1RORl92YXIxNF92c19SQkNfNmg8MC4wNSZhYnModGVtcEEkbG9nMkZvbGRDaGFuZ2VfUjNub1RORl92YXIxNF92c19SQkNfNmgpPjEmIWlzLm5hKHRlbXBBJHB2YWx1ZV9SM25vVE5GX3ZhcjE0X3ZzX1JCQ182aCkpIyMjI2ZpbmQgaW5kZXhlcyAKbGlzdEM8LXRlbXBBWyB0b3BERWdlbmVzLCBdJEdlbmVfU3ltYm9sCnRvcERFZ2VuZXMgPC0gd2hpY2godGVtcEEkcHZhbHVlX1Izbm9UTkZfdmFyMTRfdnNfUkJDXzIwaDwwLjA1JmFicyh0ZW1wQSRsb2cyRm9sZENoYW5nZV9SM25vVE5GX3ZhcjE0X3ZzX1JCQ18yMGgpPjEmIWlzLm5hKHRlbXBBJHB2YWx1ZV9SM25vVE5GX3ZhcjE0X3ZzX1JCQ18yMGgpKSMjIyNmaW5kIGluZGV4ZXMgCmxpc3REPC10ZW1wQVsgdG9wREVnZW5lcywgXSRHZW5lX1N5bWJvbAoKdmVubnA8LXZlbm4uZGlhZ3JhbSh4ID0gbGlzdChsaXN0QSxsaXN0QixsaXN0QyxsaXN0RCkgLAogICAgICAgICAgICBjYXRlZ29yeS5uYW1lcyA9IGMoIlZhcjE0bm9UTkZfMGgiLCJWYXIxNG5vVE5GXzJoIiwiVmFyMTRub1RORl82aCIsIlZhcjE0bm9UTkZfMjBoIiksCiAgICAgICAgICAgIG1haW49InB2YWx1ZTwwLjA1JmZvbGQgY2hhbmdlPjIiLAogICAgICAgICAgICBmaWxlbmFtZSA9IE5VTEwsICBzY2FsZWQgPSBGQUxTRSwgZmlsbCA9IGNvbG9yc1Y0LCBjYXQuY29sID0gY29sb3JzVjQsIGNhdC5jZXggPSAxLCBjYXQuZGlzdD0wLjMsICBtYXJnaW4gPSAwLjMpCgp0b3BERWdlbmVzIDwtIHdoaWNoKHRlbXBBJHBhZGpfUjNub1RORl92YXIxNF92c19SQkNfMGg8MC4xJmFicyh0ZW1wQSRsb2cyRm9sZENoYW5nZV9SM25vVE5GX3ZhcjE0X3ZzX1JCQ18waCk+MSYhaXMubmEodGVtcEEkcGFkal9SM25vVE5GX3ZhcjE0X3ZzX1JCQ18waCkpIyMjI2ZpbmQgaW5kZXhlcyAKbGlzdEE8LXRlbXBBWyB0b3BERWdlbmVzLCBdJEdlbmVfU3ltYm9sCnRvcERFZ2VuZXMgPC0gd2hpY2godGVtcEEkcGFkal9SM25vVE5GX3ZhcjE0X3ZzX1JCQ18yaDwwLjEmYWJzKHRlbXBBJGxvZzJGb2xkQ2hhbmdlX1Izbm9UTkZfdmFyMTRfdnNfUkJDXzJoKT4xJiFpcy5uYSh0ZW1wQSRwYWRqX1Izbm9UTkZfdmFyMTRfdnNfUkJDXzJoKSkjIyMjZmluZCBpbmRleGVzIApsaXN0QjwtdGVtcEFbIHRvcERFZ2VuZXMsIF0kR2VuZV9TeW1ib2wKdG9wREVnZW5lcyA8LSB3aGljaCh0ZW1wQSRwYWRqX1Izbm9UTkZfdmFyMTRfdnNfUkJDXzZoPDAuMSZhYnModGVtcEEkbG9nMkZvbGRDaGFuZ2VfUjNub1RORl92YXIxNF92c19SQkNfNmgpPjEmIWlzLm5hKHRlbXBBJHBhZGpfUjNub1RORl92YXIxNF92c19SQkNfNmgpKSMjIyNmaW5kIGluZGV4ZXMgCmxpc3RDPC10ZW1wQVsgdG9wREVnZW5lcywgXSRHZW5lX1N5bWJvbAp0b3BERWdlbmVzIDwtIHdoaWNoKHRlbXBBJHBhZGpfUjNub1RORl92YXIxNF92c19SQkNfMjBoPDAuMSZhYnModGVtcEEkbG9nMkZvbGRDaGFuZ2VfUjNub1RORl92YXIxNF92c19SQkNfMjBoKT4xJiFpcy5uYSh0ZW1wQSRwYWRqX1Izbm9UTkZfdmFyMTRfdnNfUkJDXzIwaCkpIyMjI2ZpbmQgaW5kZXhlcyAKbGlzdEQ8LXRlbXBBWyB0b3BERWdlbmVzLCBdJEdlbmVfU3ltYm9sCgp2ZW5ucTI8LXZlbm4uZGlhZ3JhbSh4ID0gbGlzdChsaXN0QSxsaXN0QixsaXN0QyxsaXN0RCkgLAogICAgICAgICAgICBjYXRlZ29yeS5uYW1lcyA9IGMoIlZhcjE0bm9UTkZfMGgiLCJWYXIxNG5vVE5GXzJoIiwiVmFyMTRub1RORl82aCIsIlZhcjE0bm9UTkZfMjBoIiksCiAgICAgICAgICAgIG1haW49InBhZGowLjEmZm9sZCBjaGFuZ2U+MiIsCiAgICAgICAgICAgIGZpbGVuYW1lID0gTlVMTCwgIHNjYWxlZCA9IEZBTFNFLCBmaWxsID0gY29sb3JzVjQsIGNhdC5jb2wgPSBjb2xvcnNWNCwgY2F0LmNleCA9IDEsIGNhdC5kaXN0PTAuMywgIG1hcmdpbiA9IDAuMykKCgpgYGAKCmBgYHtyfQp0b3BERWdlbmVzIDwtIHdoaWNoKCh0ZW1wQSRwYWRqX1Izbm9UTkZfdmFyMTRfdnNfUkJDXzBoPDAuMDUmIWlzLm5hKHRlbXBBJHBhZGpfUjNub1RORl92YXIxNF92c19SQkNfMGgpKXwgCih0ZW1wQSRwYWRqX1Izbm9UTkZfdmFyMTRfdnNfUkJDXzJoPDAuMDUmIWlzLm5hKHRlbXBBJHBhZGpfUjNub1RORl92YXIxNF92c19SQkNfMmgpKXwKKHRlbXBBJHBhZGpfUjNub1RORl92YXIxNF92c19SQkNfNmg8MC4wNSYhaXMubmEodGVtcEEkcGFkal9SM25vVE5GX3ZhcjE0X3ZzX1JCQ182aCkpfCAKKHRlbXBBJHBhZGpfUjNub1RORl92YXIxNF92c19SQkNfMjBoPDAuMDUmIWlzLm5hKHRlbXBBJHBhZGpfUjNub1RORl92YXIxNF92c19SQkNfMjBoKSkgCikKbGlzdEE8LXRlbXBBWyB0b3BERWdlbmVzLCBdJEdlbmVfU3ltYm9sCgp0b3BERWdlbmVzIDwtIHdoaWNoKCh0ZW1wQSRwdmFsdWVfUjNub1RORl92YXIxNF92c19SQkNfMGg8MC4wNSZhYnModGVtcEEkbG9nMkZvbGRDaGFuZ2VfUjNub1RORl92YXIxNF92c19SQkNfMGgpPjEmIWlzLm5hKHRlbXBBJHB2YWx1ZV9SM25vVE5GX3ZhcjE0X3ZzX1JCQ18waCkpfCAKKHRlbXBBJHB2YWx1ZV9SM25vVE5GX3ZhcjE0X3ZzX1JCQ18yaDwwLjA1JmFicyh0ZW1wQSRsb2cyRm9sZENoYW5nZV9SM25vVE5GX3ZhcjE0X3ZzX1JCQ18yaCk+MSYhaXMubmEodGVtcEEkcHZhbHVlX1Izbm9UTkZfdmFyMTRfdnNfUkJDXzJoKSl8IAoodGVtcEEkcHZhbHVlX1Izbm9UTkZfdmFyMTRfdnNfUkJDXzZoPDAuMDUmYWJzKHRlbXBBJGxvZzJGb2xkQ2hhbmdlX1Izbm9UTkZfdmFyMTRfdnNfUkJDXzZoKT4xJiFpcy5uYSh0ZW1wQSRwdmFsdWVfUjNub1RORl92YXIxNF92c19SQkNfNmgpKXwgCih0ZW1wQSRwdmFsdWVfUjNub1RORl92YXIxNF92c19SQkNfMjBoPDAuMDUmYWJzKHRlbXBBJGxvZzJGb2xkQ2hhbmdlX1Izbm9UTkZfdmFyMTRfdnNfUkJDXzIwaCk+MSYhaXMubmEodGVtcEEkcHZhbHVlX1Izbm9UTkZfdmFyMTRfdnNfUkJDXzIwaCkpCiApIyMjI2ZpbmQgaW5kZXhlcyAKbGlzdEM8LXRlbXBBWyB0b3BERWdlbmVzLCBdJEdlbmVfU3ltYm9sCgp0b3BERWdlbmVzIDwtIHdoaWNoKCh0ZW1wQSRwYWRqX1Izbm9UTkZfdmFyMTRfdnNfUkJDXzBoPDAuMSZhYnModGVtcEEkbG9nMkZvbGRDaGFuZ2VfUjNub1RORl92YXIxNF92c19SQkNfMGgpPjEmIWlzLm5hKHRlbXBBJHBhZGpfUjNub1RORl92YXIxNF92c19SQkNfMGgpKXwgCih0ZW1wQSRwYWRqX1Izbm9UTkZfdmFyMTRfdnNfUkJDXzJoPDAuMSZhYnModGVtcEEkbG9nMkZvbGRDaGFuZ2VfUjNub1RORl92YXIxNF92c19SQkNfMmgpPjEmIWlzLm5hKHRlbXBBJHBhZGpfUjNub1RORl92YXIxNF92c19SQkNfMmgpKXwgCih0ZW1wQSRwYWRqX1Izbm9UTkZfdmFyMTRfdnNfUkJDXzZoPDAuMSZhYnModGVtcEEkbG9nMkZvbGRDaGFuZ2VfUjNub1RORl92YXIxNF92c19SQkNfNmgpPjEmIWlzLm5hKHRlbXBBJHBhZGpfUjNub1RORl92YXIxNF92c19SQkNfNmgpKXwgCih0ZW1wQSRwYWRqX1Izbm9UTkZfdmFyMTRfdnNfUkJDXzIwaDwwLjEmYWJzKHRlbXBBJGxvZzJGb2xkQ2hhbmdlX1Izbm9UTkZfdmFyMTRfdnNfUkJDXzIwaCk+MSYhaXMubmEodGVtcEEkcGFkal9SM25vVE5GX3ZhcjE0X3ZzX1JCQ18yMGgpKQogKSMjIyNmaW5kIGluZGV4ZXMgCmxpc3RCPC10ZW1wQVsgdG9wREVnZW5lcywgXSRHZW5lX1N5bWJvbAoKdmVubnBxPC12ZW5uLmRpYWdyYW0oeCA9IGxpc3QobGlzdEEsbGlzdEIsbGlzdEMpICwKICAgICAgICAgICAgY2F0ZWdvcnkubmFtZXMgPSBjKCJwYWRqPDAuMDUiLCJwYWRqPDAuMSZmYz4yIiwicDwwLjA1JmZjPjIiKSwKICAgICAgICAgICAgbWFpbj0icGFkaiBjb21wYXJlZCB0byBwdmFsdWUiLAogICAgICAgICAgICBmaWxlbmFtZSA9IE5VTEwsICBzY2FsZWQgPSBGQUxTRSwgZmlsbCA9IGNvbG9yc1YzLCBjYXQuY29sID0gY29sb3JzVjMsIGNhdC5jZXggPSAxLCBjYXQuZGlzdD0wLjEsICBtYXJnaW4gPSAwLjE1KQpgYGAKCmBgYHtyLGZpZy5oZWlnaHQgPSAxMCxmaWcud2lkdGggPSAyMH0KZ3JpZC5hcnJhbmdlKGdUcmVlKGNoaWxkcmVuPXZlbm5xKSwgZ1RyZWUoY2hpbGRyZW49dmVubnBxKSwgbmNvbD0yLHRvcD0iUjMgVmFyMTQgbm8gVE5GIikKYGBgCgpgYGB7cn0KI3RlbXBBPC1yZXNBbGxbLWMoMTA6MzApIF0KdGVtcEE8LWNvdW50c1RhYmxlCiNyb3duYW1lcyh0ZW1wQSkKcm93bmFtZXModGVtcEEpIDwtIE5VTEwKdGVtcEEgPSBtdXRhdGUodGVtcEEsIEluY2x1ZGU9CiAgICAgICAgICAgICAgICAgICBpZmVsc2UodGVtcEEkcGFkal9SM25vVE5GX3ZhcjE0X3ZzX1JCQ18waDwwLjA1JiFpcy5uYSh0ZW1wQSRwYWRqX1Izbm9UTkZfdmFyMTRfdnNfUkJDXzBoKSwgImluIiwKICAgICAgICAgICAgICAgICAgICAgICAgICBpZmVsc2UodGVtcEEkcGFkal9SM25vVE5GX3ZhcjE0X3ZzX1JCQ18yaDwwLjA1JiFpcy5uYSh0ZW1wQSRwYWRqX1Izbm9UTkZfdmFyMTRfdnNfUkJDXzJoKSwgImluIiwKICAgICAgICAgICAgICAgICAgICAgICAgICAgICAgICAgaWZlbHNlKHRlbXBBJHBhZGpfUjNub1RORl92YXIxNF92c19SQkNfNmg8MC4wNSYhaXMubmEodGVtcEEkcGFkal9SM25vVE5GX3ZhcjE0X3ZzX1JCQ182aCksICJpbiIsCiAgICAgICAgICAgICAgICAgICAgICAgICAgICAgICAgICAgICAgICBpZmVsc2UodGVtcEEkcGFkal9SM25vVE5GX3ZhcjE0X3ZzX1JCQ18yMGg8MC4wNSYhaXMubmEodGVtcEEkcGFkal9SM25vVE5GX3ZhcjE0X3ZzX1JCQ18yMGgpLCAiaW4iLAogICAgICAgICAgICAgICAgICAgICAgICAgICAgICAgICAgICAgICAgICAgICAgICAgIm91dCIpKSkpKQoKCiN0ZW1wQQojIyMjbGlicmFyeShkcGx5cikKdGVtcEEgJT4lCiAgICAgZ3JvdXBfYnkoSW5jbHVkZSkgJT4lIAogICAgIHRhbGx5KCkKYGBgCgpgYGB7cn0KdG9wREVnZW5lcyA8LSB3aGljaCh0ZW1wQSRJbmNsdWRlPT0iaW4iKSMjIyNmaW5kIGluZGV4ZXMgCmBgYAoKYGBge3J9CmhlYWQoY291bnRzVGFibGUpCmBgYAoKCiMjIyMgTkIgUGxlYXNlIGNoZWNrIGNvbHVtbnMgdXNlZCBhbmQgcmVuYW1lZCBmb3IgcGxvdHMKYGBge3J9CiNiYXNlTWVhbnNIbSA8LWNvdW50c1RhYmxlWyxjKDYwOjYzKV0KYmFzZU1lYW5zSG0gPC1jb3VudHNUYWJsZVssYyg2MDo2Myw3OTo4MildCmhlYWQoYmFzZU1lYW5zSG0pCmJhc2VNZWFuc0htMiA8LSBsb2cyKGJhc2VNZWFuc0htKzEpCmJhc2VNZWFuc0htMiRWYXIxNF9SQkNfMGg8LWJhc2VNZWFuc0htMiRWYXIxNG5vVE5GXzBoX21lYW4tYmFzZU1lYW5zSG0yJFJCQ25vVE5GXzBoX21lYW4KYmFzZU1lYW5zSG0yJFZhcjE0X1JCQ18yaDwtYmFzZU1lYW5zSG0yJFZhcjE0bm9UTkZfMmhfbWVhbi1iYXNlTWVhbnNIbTIkUkJDbm9UTkZfMmhfbWVhbgpiYXNlTWVhbnNIbTIkVmFyMTRfUkJDXzZoPC1iYXNlTWVhbnNIbTIkVmFyMTRub1RORl82aF9tZWFuLWJhc2VNZWFuc0htMiRSQkNub1RORl82aF9tZWFuCmJhc2VNZWFuc0htMiRWYXIxNF9SQkNfMjBoPC1iYXNlTWVhbnNIbTIkVmFyMTRub1RORl8yMGhfbWVhbi1iYXNlTWVhbnNIbTIkUkJDbm9UTkZfMjBoX21lYW4KYmFzZU1lYW5zSG0gPC1iYXNlTWVhbnNIbTJbLGMoOToxMildCmhlYWQoYmFzZU1lYW5zSG0pCmJhc2VNZWFuc0htTSA8LWJhc2VNZWFuc0htMlssYygxOjgpXQpoZWFkKGJhc2VNZWFuc0htTSkKYGBgCgpgYGB7cn0KdG9wREVnZW5lcyA8LSB3aGljaCh0ZW1wQSRJbmNsdWRlPT0iaW4iKSMjIyNmaW5kIGluZGV4ZXMgCmBgYAoKCiMjIyMyLiBIaWVyYWNoaWNhbCBjbHVzdGVyaW5nIG9mIG1lYW5zIChpbmRpdmlkdWFsIHNhbXBsZXMgYWRkZWQgZm9yIGluc3BlY3Rpb24pCmBgYHtyLGZpZy5oZWlnaHQgPSAxMixmaWcud2lkdGggPSAyMH0KIyMjI21lYW4gbG9nZmMKZGF0YUhNbTwtYmFzZU1lYW5zSG1bIHRvcERFZ2VuZXMsIF0KI2RhdGFITW0gPC0gbG9nMihkYXRhSE1tKzEpCmRhdGFITW08LSB0KGFzLm1hdHJpeChkYXRhSE1tKSkKZGF0YUhNbSA8LSB0KHNjYWxlKGRhdGFITW0pKQoKaG1hcF9oaWVyX2ZhY3RvcnM0IDwtIEhlYXRtYXAoCiAgZGF0YUhNbSwgIG5hbWUgPSAibG9nZmMiLAogIHJvd19sYWJlbHMgPSBwYXN0ZTAocm93bmFtZXMoZGF0YUhNbSksIiAiLCh0ZW1wQVsgdG9wREVnZW5lcywgXSkkR2VuZV9TeW1ib2wpLAogIGNvbHVtbl90aXRsZSA9IHBhc3RlMCgiTWVhbnMgbG9nZmMiKSwgCiAgY29sID0gY29sX2Z1bkdSLAogIGNvbHVtbl90aXRsZV9ncCA9IGdwYXIoZm9udHNpemUgPSAxNiwgZm9udGZhY2UgPSAiYm9sZCIpLAogIHdpZHRoID0gdW5pdCg1MCwgIm1tIiksCiAgY2x1c3Rlcl9jb2x1bW5zID0gRkFMU0UsCiAgc2hvd19yb3dfbmFtZXMgPSBGQUxTRSkKCmRhdGFITW1QbG90PC1hcy5kYXRhLmZyYW1lKGRhdGFITW0pCmRhdGFITW1QbG90JFZhcjE0X1JCQ18yaDwtZGF0YUhNbVBsb3QkVmFyMTRfUkJDXzJoLWRhdGFITW1QbG90JFZhcjE0X1JCQ18waApkYXRhSE1tUGxvdCRWYXIxNF9SQkNfNmg8LWRhdGFITW1QbG90JFZhcjE0X1JCQ182aC1kYXRhSE1tUGxvdCRWYXIxNF9SQkNfMGgKZGF0YUhNbVBsb3QkVmFyMTRfUkJDXzIwaDwtZGF0YUhNbVBsb3QkVmFyMTRfUkJDXzIwaC1kYXRhSE1tUGxvdCRWYXIxNF9SQkNfMGgKZGF0YUhNbVBsb3QkVmFyMTRfUkJDXzBoPC1kYXRhSE1tUGxvdCRWYXIxNF9SQkNfMGgtZGF0YUhNbVBsb3QkVmFyMTRfUkJDXzBoCgpkYXRhSE1tUGxvdDwtYXMubWF0cml4KGRhdGFITW1QbG90KQogIApobWFwX2hpZXJfZmFjdG9yczYgPC0gSGVhdG1hcCgKICBkYXRhSE1tUGxvdCwgIG5hbWUgPSAiTm9ybWFsaXNlZCBsb2dmYyIsCiAgcm93X2xhYmVscyA9IHBhc3RlMChyb3duYW1lcyhkYXRhSE1tKSwiICIsKHRlbXBBWyB0b3BERWdlbmVzLCBdKSRHZW5lX1N5bWJvbCksCiAgY29sdW1uX3RpdGxlID0gcGFzdGUwKCIwaCBOb3JtYWxpc2VkIGxvZ2ZjIiksIAogIGNvbCA9IGNvbF9mdW5HUjIsCiAgY29sdW1uX3RpdGxlX2dwID0gZ3Bhcihmb250c2l6ZSA9IDE2LCBmb250ZmFjZSA9ICJib2xkIiksCiAgd2lkdGggPSB1bml0KDUwLCAibW0iKSwKICBjbHVzdGVyX2NvbHVtbnMgPSBGQUxTRSwKICBzaG93X3Jvd19uYW1lcyA9IEZBTFNFKQoKIyMjI21lYW5zCmRhdGFITW0zPC1iYXNlTWVhbnNIbU1bIHRvcERFZ2VuZXMsIF0KI2RhdGFITW0gPC0gbG9nMihkYXRhSE1tKzEpCmRhdGFITW0zPC0gdChhcy5tYXRyaXgoZGF0YUhNbTMpKQpkYXRhSE1tMyA8LSB0KHNjYWxlKGRhdGFITW0zKSkKZGF0YUhNbTM8LWRhdGFITW0zWyxjKDUsMSw2LDIsNywzLDgsNCldCmhtYXBfaGllcl9mYWN0b3JzNSA8LSBIZWF0bWFwKAogIGRhdGFITW0zLCAgbmFtZSA9ICJFeHByZXNzaW9uIiwKICByb3dfbGFiZWxzID0gcGFzdGUwKHJvd25hbWVzKGRhdGFITW0pLCIgIiwodGVtcEFbIHRvcERFZ2VuZXMsIF0pJEdlbmVfU3ltYm9sKSwKICBjb2x1bW5fdGl0bGUgPSBwYXN0ZTAoIk1lYW5zIiksIAogIGNvbCA9IGNvbF9mdW4sCiAgY29sdW1uX3RpdGxlX2dwID0gZ3Bhcihmb250c2l6ZSA9IDE2LCBmb250ZmFjZSA9ICJib2xkIiksCiAgd2lkdGggPSB1bml0KDEwMCwgIm1tIiksCiAgY2x1c3Rlcl9jb2x1bW5zID0gRkFMU0UsCiAgc2hvd19yb3dfbmFtZXMgPSBGQUxTRSkKICAKaG1hcF9oaWVyX2ZhY3RvcnM0K2htYXBfaGllcl9mYWN0b3JzNitobWFwX2hpZXJfZmFjdG9yczUKYGBgCgoKYGBge3IsZmlnLmhlaWdodCA9IDcsZmlnLndpZHRoID0xMiB9CnBhcihtZnJvdz1jKDEsMikpCiMjIyMgU2lsaG91ZXR0ZSBtZXRob2QKZnZpel9uYmNsdXN0KGRhdGFITW0sIGttZWFucywgbWV0aG9kID0gInNpbGhvdWV0dGUiLGsubWF4ID0gMTYpKwogIGxhYnMoc3VidGl0bGUgPSAiU2lsaG91ZXR0ZSBtZXRob2QiKQoKIyMjIyBFbGJvdyBtZXRob2QKZnZpel9uYmNsdXN0KGRhdGFITW0sIGttZWFucywgbWV0aG9kID0gIndzcyIsay5tYXggPSAxNikgKwogIGxhYnMoc3VidGl0bGUgPSAiRWxib3cgbWV0aG9kIikKCmBgYAoKYGBge3J9CiMjIyNnYXAgc3RhdCBzbG93ISEhCiMjIyNzZXQuc2VlZCgxMjMpCiMjIyNmdml6X25iY2x1c3QoZGF0YUhNbSwga21lYW5zLCBuc3RhcnQgPSAyNSwgIG1ldGhvZCA9ICJnYXBfc3RhdCIsIG5ib290ID0gMTAwLGsubWF4ID0gMTYpKwojIyMjICBsYWJzKHN1YnRpdGxlID0gIkdhcCBzdGF0aXN0aWMgbWV0aG9kIikKYGBgCgoKYGBge3IsZmlnLmhlaWdodCA9IDd9CiNrY2x1c3QzIDwtIGttZWFucyhkYXRhSE1tLCA3KQojc2lsaG91ZXR0ZSBwbG90CmRpc3RLPC1kYWlzeShkYXRhSE1tKQpwbG90KHNpbGhvdWV0dGUoa2NsdXN0MyRjbHVzdGVyLCBkaXN0SyksIGNvbD0xOjcsIGJvcmRlcj1OQSkKCmBgYAoKIyMjIzMuIEstbWVhbnMgY2x1c3RlcmluZyBvZiBtZWFucyAKYGBge3IsZmlnLmhlaWdodCA9IDEwLGZpZy53aWR0aCA9IDIwfQpzcGxpdCA8LSBwYXN0ZTAoIkNsdXN0ZXJcbiIsIGtjbHVzdDMkY2x1c3RlcikKI3NwbGl0IDwtIGZhY3RvcihwYXN0ZTAoIkNsdXN0ZXJcbiIsIGtjbHVzdDMkY2x1c3RlciksIGxldmVscz1jKCJDbHVzdGVyXG4zIiwiQ2x1c3RlclxuMSIsIkNsdXN0ZXJcbjQiLCJDbHVzdGVyXG41IiwiQ2x1c3RlclxuMiIsIkNsdXN0ZXJcbjYiKSkKaG1hcF9rIDwtIEhlYXRtYXAoZGF0YUhNbSwgc3BsaXQ9c3BsaXQsIGNsdXN0ZXJfcm93X3NsaWNlcyA9IEZBTFNFLAogICAgICAgICAgICAgICAgICBjbHVzdGVyX2NvbHVtbnMgPSBGQUxTRSwKICAgICAgICAgICAgICAgICAgc2hvd19yb3dfbmFtZXMgPSBGQUxTRSwKICAgICAgICAgICAgICAgICAgbmFtZSA9ICJsb2dmYyIsCiAgICAgICAgICAgICAgICAgIGNvbCA9IGNvbF9mdW5HUiwKICAgICAgICAgICAgICAgICAgd2lkdGggPSB1bml0KDUwLCAibW0iKSwKICAgICAgICAgICAgICAgICAgY29sdW1uX3RpdGxlID0gIm1lYW5zIGxvZ2ZjIiwgCiAgICAgICAgICAgICAgICAgIGNvbHVtbl90aXRsZV9ncCA9IGdwYXIoZm9udHNpemUgPSAxNiwgZm9udGZhY2UgPSAiYm9sZCIpKQoKaG1hcF9rK2htYXBfaGllcl9mYWN0b3JzNitobWFwX2hpZXJfZmFjdG9yczUKYGBgCgpNZWFuIHByb2ZpbGVzIG9mIGNsdXN0ZXJzCgpgYGB7cixmaWcuaGVpZ2h0ID0gOCxmaWcud2lkdGggPSA4fQpjbHVzdGVyY291bnQ8LWRhdGEuZnJhbWUoa2NsdXN0MyRjbHVzdGVyKQpjbHVzdGVyc2l6ZXM8LXRhYmxlKGNsdXN0ZXJjb3VudCRrY2x1c3QzLmNsdXN0ZXIpCmNsdXN0ZXJNZWFuczwtZGF0YS5mcmFtZShrY2x1c3QzJGNlbnRlcnMpCmNsdXN0ZXJNZWFuczE8LWRhdGEuZnJhbWUodChjbHVzdGVyTWVhbnMpKQpjbHVzdGVyTWVhbnMxIDwtIGNiaW5kKHJvd25hbWVzKGNsdXN0ZXJNZWFuczEpLCBjbHVzdGVyTWVhbnMxKQpvcmRlck48LWMoIlZhcjE0X1JCQ18waCIsIlZhcjE0X1JCQ18yaCIsIlZhcjE0X1JCQ182aCIsIlZhcjE0X1JCQ18yMGgiKSMjIyMgbWFudWFsCgpyb3duYW1lcyhjbHVzdGVyTWVhbnMxKSA8LSBOVUxMCm5hbWVzKGNsdXN0ZXJNZWFuczEpW25hbWVzKGNsdXN0ZXJNZWFuczEpPT0icm93bmFtZXMoY2x1c3Rlck1lYW5zMSkiXSA8LSAiU2FtcGxlIgojIyMjY2x1c3Rlck1lYW5zMQoKcFgxPC1nZ3Bsb3QoZGF0YT1jbHVzdGVyTWVhbnMxLCBhZXMoeD1TYW1wbGUsIHk9WDEsZ3JvdXA9MSkpICsKICBnZW9tX2xpbmUoKSsgIGdlb21fcG9pbnQoKStnZ3RpdGxlKHBhc3RlKCJDbHVzdGVyIFgxIFByb2ZpbGUgIixjbHVzdGVyc2l6ZXNbMV0sIiBnZW5lcyIpKSsgIHNjYWxlX3hfZGlzY3JldGUobGltaXRzPW9yZGVyTikrCiAgdGhlbWUoYXhpcy50aXRsZS54ID0gZWxlbWVudF9ibGFuaygpLGF4aXMudGl0bGUueSA9IGVsZW1lbnRfYmxhbmsoKSkKcFgyPC1nZ3Bsb3QoZGF0YT1jbHVzdGVyTWVhbnMxLCBhZXMoeD1TYW1wbGUsIHk9WDIsZ3JvdXA9MSkpICsKICBnZW9tX2xpbmUoKSsgIGdlb21fcG9pbnQoKStnZ3RpdGxlKHBhc3RlKCJDbHVzdGVyIFgyIFByb2ZpbGUgIixjbHVzdGVyc2l6ZXNbMl0sIiBnZW5lcyIpKSsgIHNjYWxlX3hfZGlzY3JldGUobGltaXRzPW9yZGVyTikrCiAgdGhlbWUoYXhpcy50aXRsZS54ID0gZWxlbWVudF9ibGFuaygpLGF4aXMudGl0bGUueSA9IGVsZW1lbnRfYmxhbmsoKSkKcFgzPC1nZ3Bsb3QoZGF0YT1jbHVzdGVyTWVhbnMxLCBhZXMoeD1TYW1wbGUsIHk9WDMsZ3JvdXA9MSkpICsKICBnZW9tX2xpbmUoKSsgIGdlb21fcG9pbnQoKStnZ3RpdGxlKHBhc3RlKCJDbHVzdGVyIFgzIFByb2ZpbGUgIixjbHVzdGVyc2l6ZXNbM10sIiBnZW5lcyIpKSsgIHNjYWxlX3hfZGlzY3JldGUobGltaXRzPW9yZGVyTikrCiAgdGhlbWUoYXhpcy50aXRsZS54ID0gZWxlbWVudF9ibGFuaygpLGF4aXMudGl0bGUueSA9IGVsZW1lbnRfYmxhbmsoKSkKcFg0PC1nZ3Bsb3QoZGF0YT1jbHVzdGVyTWVhbnMxLCBhZXMoeD1TYW1wbGUsIHk9WDQsZ3JvdXA9MSkpICsKICBnZW9tX2xpbmUoKSsgIGdlb21fcG9pbnQoKStnZ3RpdGxlKHBhc3RlKCJDbHVzdGVyIFg0IFByb2ZpbGUgIixjbHVzdGVyc2l6ZXNbNF0sIiBnZW5lcyIpKSsgIHNjYWxlX3hfZGlzY3JldGUobGltaXRzPW9yZGVyTikrCiAgdGhlbWUoYXhpcy50aXRsZS54ID0gZWxlbWVudF9ibGFuaygpLGF4aXMudGl0bGUueSA9IGVsZW1lbnRfYmxhbmsoKSkKcFg1PC1nZ3Bsb3QoZGF0YT1jbHVzdGVyTWVhbnMxLCBhZXMoeD1TYW1wbGUsIHk9WDUsZ3JvdXA9MSkpICsKICBnZW9tX2xpbmUoKSsgIGdlb21fcG9pbnQoKStnZ3RpdGxlKHBhc3RlKCJDbHVzdGVyIFg1IFByb2ZpbGUgIixjbHVzdGVyc2l6ZXNbNV0sIiBnZW5lcyIpKSsgIHNjYWxlX3hfZGlzY3JldGUobGltaXRzPW9yZGVyTikrCiAgdGhlbWUoYXhpcy50aXRsZS54ID0gZWxlbWVudF9ibGFuaygpLGF4aXMudGl0bGUueSA9IGVsZW1lbnRfYmxhbmsoKSkKcFg2PC1nZ3Bsb3QoZGF0YT1jbHVzdGVyTWVhbnMxLCBhZXMoeD1TYW1wbGUsIHk9WDYsZ3JvdXA9MSkpICsKICBnZW9tX2xpbmUoKSsgIGdlb21fcG9pbnQoKStnZ3RpdGxlKHBhc3RlKCJDbHVzdGVyIFg2IFByb2ZpbGUgIixjbHVzdGVyc2l6ZXNbNl0sIiBnZW5lcyIpKSsgIHNjYWxlX3hfZGlzY3JldGUobGltaXRzPW9yZGVyTikrCiAgdGhlbWUoYXhpcy50aXRsZS54ID0gZWxlbWVudF9ibGFuaygpLGF4aXMudGl0bGUueSA9IGVsZW1lbnRfYmxhbmsoKSkKcFg3PC1nZ3Bsb3QoZGF0YT1jbHVzdGVyTWVhbnMxLCBhZXMoeD1TYW1wbGUsIHk9WDcsZ3JvdXA9MSkpICsKICBnZW9tX2xpbmUoKSsgIGdlb21fcG9pbnQoKStnZ3RpdGxlKHBhc3RlKCJDbHVzdGVyIFg3IFByb2ZpbGUgIixjbHVzdGVyc2l6ZXNbNV0sIiBnZW5lcyIpKSsgIHNjYWxlX3hfZGlzY3JldGUobGltaXRzPW9yZGVyTikrCiAgdGhlbWUoYXhpcy50aXRsZS54ID0gZWxlbWVudF9ibGFuaygpLGF4aXMudGl0bGUueSA9IGVsZW1lbnRfYmxhbmsoKSkKI3BYODwtZ2dwbG90KGRhdGE9Y2x1c3Rlck1lYW5zMSwgYWVzKHg9U2FtcGxlLCB5PVg4LGdyb3VwPTEpKSArCiMgIGdlb21fbGluZSgpKyAgZ2VvbV9wb2ludCgpK2dndGl0bGUocGFzdGUoIkNsdXN0ZXIgWDggUHJvZmlsZSAiLGNsdXN0ZXJzaXplc1s2XSwiIGdlbmVzIikpKyAgc2NhbGVfeF9kaXNjcmV0ZShsaW1pdHM9b3JkZXJOKSsKIyAgdGhlbWUoYXhpcy50aXRsZS54ID0gZWxlbWVudF9ibGFuaygpLGF4aXMudGl0bGUueSA9IGVsZW1lbnRfYmxhbmsoKSkKCgojcGxvdAptdWx0aXBsb3QocFgxLCBwWDIsIHBYMywgcFg0LHBYNSwgcFg2LHBYNywgIGNvbHM9MikKCmBgYAoKYGBge3J9CnRvcERFZ2VuZXMgPC0gd2hpY2godGVtcEEkSW5jbHVkZT09ImluIikjIyMjZmluZCBpbmRleGVzCnRlbXBBa208LXRlbXBBWyB0b3BERWdlbmVzLCBdClN5bWJvbHNLbTwtZHBseXI6OnB1bGwodGVtcEFrbSwgR2VuZV9TeW1ib2wpCiMjIyMgZXhwb3J0IHRoZSBnZW5lIGV4cHJlc3Npb24gZGF0YSBmb3IgdGhlIGNsdXN0ZXJzCndyaXRlLnRhYmxlKGNsdXN0ZXJNZWFucyxwYXN0ZTAoIkNsdXN0ZXJNZWFuc0ttXyIsZ3JvdXBzTmFtZSwiLnR4dCIpLCAgc2VwID0gIlx0IikKQ2x1c3RlcmVkR2VuZXM8LWRhdGEuZnJhbWUoa2NsdXN0MyRjbHVzdGVyLFN5bWJvbHNLbSxkYXRhSE1tKQp3cml0ZS50YWJsZShDbHVzdGVyZWRHZW5lcyxwYXN0ZTAoIlNjYWxlZERhdGFJbkNsdXN0ZXJzS21fIixncm91cHNOYW1lLCIudHh0IiksICBzZXAgPSAiXHQiKQojaGVhZChDbHVzdGVyZWRHZW5lcykKYGBgCgoKYGBge3J9CmJvdHRvbURFZ2VuZXM8LXdoaWNoKHRlbXBBJEluY2x1ZGU9PSJvdXQiKSMjIyNmaW5kIGluZGV4ZXMgCmJvdHRvbUc8LXRlbXBBWyBib3R0b21ERWdlbmVzLCBdCmJvdHRvbUc8LWRwbHlyOjpwdWxsKGJvdHRvbUcsIEdlbmVfU3ltYm9sKQp3cml0ZS50YWJsZShib3R0b21HLHBhc3RlMCgiaXBhQm90dG9tS21lYW5zXyIsZ3JvdXBzTmFtZSwiLnR4dCIpLCAgc2VwID0gIlx0IikKICAgICAgICAgICAgICAgICAgICAgICAgIAoKdG9wREVnZW5lcyA8LSB3aGljaCh0ZW1wQSRJbmNsdWRlPT0iaW4iKSMjIyNmaW5kIGluZGV4ZXMgCnRlbXBBa208LXRlbXBBWyB0b3BERWdlbmVzLCBdClN5bWJvbHNLbTwtZHBseXI6OnB1bGwodGVtcEFrbSwgR2VuZV9TeW1ib2wpCgppcGFLbWVhbnM8LUNsdXN0ZXJlZEdlbmVzCiNjb3VudHNUYWJsZSA8LWNvdW50c1RhYmxlWyxjKDE6MTUpXSMjIyNpZiBzYW1wbGVzIG5lZWQgcmVtb3ZpbmcKaXBhS21lYW5zPC1pcGFLbWVhbnNbLGMoMToyKV0KaXBhS21lYW5zJG5hbWUyPC1yb3duYW1lcyhpcGFLbWVhbnMpCiNpcGFLbWVhbnMlPiUgcm93bmFtZXNfdG9fY29sdW1uKHZhciA9ICJyb3duYW1lIikKI2lwYUttZWFucwojcm93aWRfdG9fY29sdW1uKGlwYUttZWFucykKaXBhS21lYW5zID0gbXV0YXRlKGlwYUttZWFucywgeDE9IGlmZWxzZShpcGFLbWVhbnMka2NsdXN0My5jbHVzdGVyPT0xLCAiMSIsICIwIikpCmlwYUttZWFucyA9IG11dGF0ZShpcGFLbWVhbnMsIHgyPSBpZmVsc2UoaXBhS21lYW5zJGtjbHVzdDMuY2x1c3Rlcj09MiwgIjEiLCAiMCIpKQppcGFLbWVhbnMgPSBtdXRhdGUoaXBhS21lYW5zLCB4Mz0gaWZlbHNlKGlwYUttZWFucyRrY2x1c3QzLmNsdXN0ZXI9PTMsICIxIiwgIjAiKSkKaXBhS21lYW5zID0gbXV0YXRlKGlwYUttZWFucywgeDQ9IGlmZWxzZShpcGFLbWVhbnMka2NsdXN0My5jbHVzdGVyPT00LCAiMSIsICIwIikpCmlwYUttZWFucyA9IG11dGF0ZShpcGFLbWVhbnMsIHg1PSBpZmVsc2UoaXBhS21lYW5zJGtjbHVzdDMuY2x1c3Rlcj09NSwgIjEiLCAiMCIpKQppcGFLbWVhbnMgPSBtdXRhdGUoaXBhS21lYW5zLCB4Nj0gaWZlbHNlKGlwYUttZWFucyRrY2x1c3QzLmNsdXN0ZXI9PTYsICIxIiwgIjAiKSkKaXBhS21lYW5zID0gbXV0YXRlKGlwYUttZWFucywgeDc9IGlmZWxzZShpcGFLbWVhbnMka2NsdXN0My5jbHVzdGVyPT03LCAiMSIsICIwIikpCiNpcGFLbWVhbnMKd3JpdGUudGFibGUoaXBhS21lYW5zLHBhc3RlMCgiaXBhS21lYW5zXyIsZ3JvdXBzTmFtZSwiLnR4dCIpLCAgc2VwID0gIlx0IikKI2hlYWQoaXBhS21lYW5zKQoKYGBgCgoKYGBge3J9CkNsdXN0ZXJlZEdlbmVzMjwtQ2x1c3RlcmVkR2VuZXNbYygxKV0KI0NsdXN0ZXJlZEdlbmVzMgpsaXN0QWxsPC1saXN0KCkKZm9yKGkgaW4gMTo3KSB7CiAgY2x1c3Rlck5hbWU8LXBhc3RlMCgieCIsaSkKICAjY2x1c3Rlck5hbWU8LXJvdy5uYW1lcyhzdWJzZXQoQ2x1c3RlcmVkR2VuZXMsQ2x1c3RlcmVkR2VuZXM9PWkpKQogIGNsdXN0ZXJOYW1lPC0oc3Vic2V0KENsdXN0ZXJlZEdlbmVzJFN5bWJvbHNLbSxDbHVzdGVyZWRHZW5lcz09aSkpCiAgbGlzdEFsbFtbaV1dPC1jbHVzdGVyTmFtZQp9CiNuZWVkIHRvIG5hbWUgdGhlIHZlY3RvcnMgaW4gdGhlIGxpc3QsIGV4YW1wbGUgaGVyZSBpcyBmb3IgOCBjbHVzdGVycwpuYW1lcyhsaXN0QWxsKTwtYygiWDEiLCAiWDIiLCAiWDMiLCAiWDQiLCJYNSIsICJYNiIsICJYNyIpCgojaWYgeW91IHdhbnQgdG8gcmVhcnJhbmdlIHRoZSBvcmRlcgojbGlzdEFsbDwtbGlzdEFsbFtjKCJ4MyIsICJ4NyIsICJ4OCIsICJ4MiIsICJ4NiIsICJ4NSIsICJ4NCIsICJ4MSIpXQoKI2xhcHBseShsaXN0QWxsLCBoZWFkKQoKYGBgCgojIyMjNC4gQW5ub3RhdGlvbiBvZiBLLW1lYW5zIGNsdXN0ZXJzCi0gQ0MgY2VsbHVsYXIgY29tcGFydG1lbnQKLSBCUCBiaW9sb2dpY2FsIHByb2Nlc3MKLSBNRiBtb2xlY3VsYXIgZnVuY3Rpb24KClRoZSBzaW1wbGlmeSBmdW5jdGlvbiBoYXMgYmVlbiB1c2VkIHRvIGN1dCBkb3duIG9uIEdPIHJlZHVuZGFuY3kKCmBgYHtyfQojc3RyKEFsbEdlbmVOYW1lcykKYGBgCgoKYGBge3IsZmlnLmhlaWdodCA9IDgsZmlnLndpZHRoID0gMTJ9CiMjIyNDQwpjZ29DQyA8LSBjb21wYXJlQ2x1c3RlcihnZW5lQ2x1c3RlciA9IGxpc3RBbGwsIAogICAgICAgICAgICAgICAgICAgICAgdW5pdmVyc2UgPSBBbGxHZW5lTmFtZXMsCiAgICAgICAgICAgICAgICAgICAgICBmdW4gPSAiZW5yaWNoR08iLAogICAgICAgICAgICAgICAgICAgICAgT3JnRGI9b3JnLkhzLmVnLmRiLCAKICAgICAgICAgICAgICAgICAgICAgICMjIyNPcmdEYj1vcmcuTW0uZWcuZGIsCiAgICAgICAgICAgICAgICAgICAgICBrZXlUeXBlPSJTWU1CT0wiLAogICAgICAgICAgICAgICAgICAgICAgb250ID0gIkNDIiwgCiAgICAgICAgICAgICAgICAgICAgICBwdmFsdWVDdXRvZmY9MC4wNSwKICAgICAgICAgICAgICAgICAgICAgIHF2YWx1ZUN1dG9mZiA9IDAuMTApCmNnb0NDMiA8LSBzaW1wbGlmeShjZ29DQywgY3V0b2ZmPTAuNywgYnk9InAuYWRqdXN0Iiwgc2VsZWN0X2Z1bj1taW4pCiMjIyN3cml0ZSBhcyBzcHJlYWRzaGVldAp3cml0ZS5jc3YoYXMuZGF0YS5mcmFtZShjZ29DQzIpLHBhc3RlMCgiR09fQ0NfIixncm91cHNOYW1lLCIuY3N2IikpCgpkb3RwbG90KGNnb0NDMixzaG93Q2F0ZWdvcnkgPSAzMCwKICAgICAgICB0aXRsZSA9IHBhc3RlMCgiR08gQ2VsbHVsYXIgQ29tcGFydG1lbnQgIixncm91cHNOYW1lKSkrCiAgdGhlbWUoYXhpcy50ZXh0LnggPSBlbGVtZW50X3RleHQoYW5nbGUgPSA5MCwgdmp1c3QgPSAwLjUsIGhqdXN0PTEpKQoKYGBgClBsb3RzIGFuZCBHTyBkYXRhIHdlcmUgd3JpdHRlbiB0byBmaWxlcwpgYGB7cn0KcG5nKHBhc3RlMCgiR09fQ0NfIixncm91cHNOYW1lLCIucG5nIiksIHdpZHRoID0gMTIyNCwgaGVpZ2h0ID0gODI0KQpkb3RwbG90KGNnb0NDMixzaG93Q2F0ZWdvcnkgPSAzMCwKICAgICAgICB0aXRsZSA9IHBhc3RlMCgiR08gQ2VsbHVsYXIgQ29tcGFydG1lbnQgIixncm91cHNOYW1lKSkrCiAgdGhlbWUoYXhpcy50ZXh0LnggPSBlbGVtZW50X3RleHQoYW5nbGUgPSA5MCwgdmp1c3QgPSAwLjUsIGhqdXN0PTEpKQpkZXYub2ZmKCkKYGBgCgpHTyBCUApgYGB7cixmaWcuaGVpZ2h0ID0gMTIsZmlnLndpZHRoID0gMTJ9CiMjIyNDQwpjZ29CUCA8LSBjb21wYXJlQ2x1c3RlcihnZW5lQ2x1c3RlciA9IGxpc3RBbGwsIAogICAgICAgICAgICAgICAgICAgICAgdW5pdmVyc2UgPSBBbGxHZW5lTmFtZXMsCiAgICAgICAgICAgICAgICAgICAgICBmdW4gPSAiZW5yaWNoR08iLAogICAgICAgICAgICAgICAgICAgICAgT3JnRGI9b3JnLkhzLmVnLmRiLAogICAgICAgICAgICAgICAgICAgICAga2V5VHlwZT0iU1lNQk9MIiwKICAgICAgICAgICAgICAgICAgICAgIG9udCA9ICJCUCIsIAogICAgICAgICAgICAgICAgICAgICAgcHZhbHVlQ3V0b2ZmPTAuMDUsCiAgICAgICAgICAgICAgICAgICAgICBxdmFsdWVDdXRvZmYgPSAwLjEwKQpjZ29CUDIgPC0gc2ltcGxpZnkoY2dvQlAsIGN1dG9mZj0wLjcsIGJ5PSJwLmFkanVzdCIsIHNlbGVjdF9mdW49bWluKQojIyMjd3JpdGUgYXMgc3ByZWFkc2hlZXQKd3JpdGUuY3N2KGFzLmRhdGEuZnJhbWUoY2dvQlAyKSxwYXN0ZTAoIkdPX0JQXyIsZ3JvdXBzTmFtZSwiLmNzdiIpKQoKZG90cGxvdChjZ29CUDIsc2hvd0NhdGVnb3J5ID0gMzAsCiAgICAgICAgdGl0bGUgPSBwYXN0ZTAoIkdPIEJpb2xvZ2ljYWwgUHJvY2VzcyAiLGdyb3Vwc05hbWUpKSsKICB0aGVtZShheGlzLnRleHQueCA9IGVsZW1lbnRfdGV4dChhbmdsZSA9IDkwLCB2anVzdCA9IDAuNSwgaGp1c3Q9MSkpCgpgYGAKCmBgYHtyfQpwbmcocGFzdGUwKCJHT19CUF8iLGdyb3Vwc05hbWUsIi5wbmciKSwgd2lkdGggPSAxMDI0LCBoZWlnaHQgPSAxMjI0KQpkb3RwbG90KGNnb0JQMixzaG93Q2F0ZWdvcnkgPSAzMCwKICAgICAgICB0aXRsZSA9IHBhc3RlMCgiR08gQmlvbG9naWNhbCBQcm9jZXNzICIsZ3JvdXBzTmFtZSkpKwogIHRoZW1lKGF4aXMudGV4dC54ID0gZWxlbWVudF90ZXh0KGFuZ2xlID0gOTAsIHZqdXN0ID0gMC41LCBoanVzdD0xKSkKZGV2Lm9mZigpCmBgYAoKCkdPIE1GCmBgYHtyLGZpZy5oZWlnaHQgPSAxMCxmaWcud2lkdGggPSAxMn0KIyMjI01GCmNnb01GIDwtIGNvbXBhcmVDbHVzdGVyKGdlbmVDbHVzdGVyID0gbGlzdEFsbCwgCiAgICAgICAgICAgICAgICAgICAgICB1bml2ZXJzZSA9IEFsbEdlbmVOYW1lcywKICAgICAgICAgICAgICAgICAgICAgIGZ1biA9ICJlbnJpY2hHTyIsCiAgICAgICAgICAgICAgICAgICAgICBPcmdEYj1vcmcuSHMuZWcuZGIsIAogICAgICAgICAgICAgICAgICAgICAga2V5VHlwZT0iU1lNQk9MIiwKICAgICAgICAgICAgICAgICAgICAgIG9udCA9ICJNRiIsIAogICAgICAgICAgICAgICAgICAgICAgcHZhbHVlQ3V0b2ZmPTAuMDUsCiAgICAgICAgICAgICAgICAgICAgICBxdmFsdWVDdXRvZmYgPSAwLjEwKQpjZ29NRjIgPC0gc2ltcGxpZnkoY2dvTUYsIGN1dG9mZj0wLjcsIGJ5PSJwLmFkanVzdCIsIHNlbGVjdF9mdW49bWluKQojIyMjd3JpdGUgYXMgc3ByZWFkc2hlZXQKd3JpdGUuY3N2KGFzLmRhdGEuZnJhbWUoY2dvTUYyKSxwYXN0ZTAoIkdPX01GXyIsZ3JvdXBzTmFtZSwiLmNzdiIpKQoKZG90cGxvdChjZ29NRjIsc2hvd0NhdGVnb3J5ID0gMzAsCiAgICAgICAgdGl0bGUgPSBwYXN0ZTAoIkdPIE1vbGVjdWxhciBGdW5jdGlvbiAgIixncm91cHNOYW1lKSkrCiAgdGhlbWUoYXhpcy50ZXh0LnggPSBlbGVtZW50X3RleHQoYW5nbGUgPSA5MCwgdmp1c3QgPSAwLjUsIGhqdXN0PTEpKQoKYGBgCgoKCmBgYHtyfQpwbmcocGFzdGUwKCJHT19NRl8iLGdyb3Vwc05hbWUsIi5wbmciKSwgd2lkdGggPSAxNDI0LCBoZWlnaHQgPSA4MjQpCmRvdHBsb3QoY2dvTUYyLHNob3dDYXRlZ29yeSA9IDMwLAogICAgICAgIHRpdGxlID0gcGFzdGUwKCJHTyBNb2xlY3VsYXIgRnVuY3Rpb24gICIsZ3JvdXBzTmFtZSkpKwogIHRoZW1lKGF4aXMudGV4dC54ID0gZWxlbWVudF90ZXh0KGFuZ2xlID0gOTAsIHZqdXN0ID0gMC41LCBoanVzdD0xKSkKZGV2Lm9mZigpCmBgYAoKCgoKIyMjIFIzIFZBUjE0IHZzIFJCQyBubyBUTkYgay1tZWFucyBwMC4wNWZjMgoKIyMjIzEuIEdlbmVsaXN0IFNlbGVjdGlvbgoKCmBgYHtyfQpncm91cHNOYW1lPC0iUjNfVkFSMTRfa21lYW5zX3AwLjA1ZmMyIgpgYGAKCgpgYGB7cn0KY291bnRzVGFibGU8LXJlYWQuZGVsaW0oIlJOQXNlcTIwMTlKdWx5XzUudHh0IiwgaGVhZGVyID0gVFJVRSwgc2VwID0gIlx0IixjaGVjay5uYW1lcz1GQUxTRSxyb3cubmFtZXM9MSkKaGVhZChjb3VudHNUYWJsZSkKYGBgCgpgYGB7cn0KQWxsR2VuZU5hbWVzPC1jb3VudHNUYWJsZSRHZW5lX1N5bWJvbAojaGVhZChBbGxHZW5lTmFtZXMpCmBgYAoKYGBge3IsZmlnLmhlaWdodCA9IDEwLGZpZy53aWR0aCA9IDIwfQpncmlkLmFycmFuZ2UoZ1RyZWUoY2hpbGRyZW49dmVubnApLCBnVHJlZShjaGlsZHJlbj12ZW5ucHEpICwgbmNvbD0yLHRvcD0iUjMgVmFyMTQgbm8gVE5GIikKYGBgCgpgYGB7cn0KI3RlbXBBPC1yZXNBbGxbLWMoMTA6MzApIF0KdGVtcEE8LWNvdW50c1RhYmxlCiNyb3duYW1lcyh0ZW1wQSkKcm93bmFtZXModGVtcEEpIDwtIE5VTEwKdGVtcEEgPSBtdXRhdGUodGVtcEEsIEluY2x1ZGU9CiAgICAgICAgICAgICAgICAgICBpZmVsc2UodGVtcEEkcHZhbHVlX1Izbm9UTkZfdmFyMTRfdnNfUkJDXzBoPDAuMDUmYWJzKHRlbXBBJGxvZzJGb2xkQ2hhbmdlX1Izbm9UTkZfdmFyMTRfdnNfUkJDXzBoKT4xJiFpcy5uYSh0ZW1wQSRwdmFsdWVfUjNub1RORl92YXIxNF92c19SQkNfMGgpLCAiaW4iLAogICAgICAgICAgICAgICAgICAgICAgICAgIGlmZWxzZSh0ZW1wQSRwdmFsdWVfUjNub1RORl92YXIxNF92c19SQkNfMmg8MC4wNSZhYnModGVtcEEkbG9nMkZvbGRDaGFuZ2VfUjNub1RORl92YXIxNF92c19SQkNfMmgpPjEmIWlzLm5hKHRlbXBBJHB2YWx1ZV9SM25vVE5GX3ZhcjE0X3ZzX1JCQ18yaCksICJpbiIsCiAgICAgICAgICAgICAgICAgICAgICAgICAgICAgICAgIGlmZWxzZSh0ZW1wQSRwdmFsdWVfUjNub1RORl92YXIxNF92c19SQkNfNmg8MC4wNSZhYnModGVtcEEkbG9nMkZvbGRDaGFuZ2VfUjNub1RORl92YXIxNF92c19SQkNfNmgpPjEmIWlzLm5hKHRlbXBBJHB2YWx1ZV9SM25vVE5GX3ZhcjE0X3ZzX1JCQ182aCksICJpbiIsCiAgICAgICAgICAgICAgICAgICAgICAgICAgICAgICAgICAgICAgICBpZmVsc2UodGVtcEEkcHZhbHVlX1Izbm9UTkZfdmFyMTRfdnNfUkJDXzIwaDwwLjA1JmFicyh0ZW1wQSRsb2cyRm9sZENoYW5nZV9SM25vVE5GX3ZhcjE0X3ZzX1JCQ18yMGgpPjEmIWlzLm5hKHRlbXBBJHB2YWx1ZV9SM25vVE5GX3ZhcjE0X3ZzX1JCQ18yMGgpLCAiaW4iLAogICAgICAgICAgICAgICAgICAgICAgICAgICAgICAgICAgICAgICAgICAgICAgICAgICAgICAgICAgICAgICAgICAgICAgICJvdXQiKSkpKSkKCgojdGVtcEEKIyMjI2xpYnJhcnkoZHBseXIpCnRlbXBBICU+JQogICAgIGdyb3VwX2J5KEluY2x1ZGUpICU+JSAKICAgICB0YWxseSgpCgpgYGAKCmBgYHtyfQp0b3BERWdlbmVzIDwtIHdoaWNoKHRlbXBBJEluY2x1ZGU9PSJpbiIpIyMjI2ZpbmQgaW5kZXhlcyAKYGBgCgoKCiMjIyMgTkIgUGxlYXNlIGNoZWNrIGNvbHVtbnMgdXNlZCBhbmQgcmVuYW1lZCBmb3IgcGxvdHMKYGBge3J9CmJhc2VNZWFuc0htIDwtY291bnRzVGFibGVbLGMoNjA6NjMsNzk6ODIpXQpiYXNlTWVhbnNIbTIgPC0gbG9nMihiYXNlTWVhbnNIbSsxKQpiYXNlTWVhbnNIbTIkVmFyMTRfUkJDXzBoPC1iYXNlTWVhbnNIbTIkVmFyMTRub1RORl8waF9tZWFuLWJhc2VNZWFuc0htMiRSQkNub1RORl8waF9tZWFuCmJhc2VNZWFuc0htMiRWYXIxNF9SQkNfMmg8LWJhc2VNZWFuc0htMiRWYXIxNG5vVE5GXzJoX21lYW4tYmFzZU1lYW5zSG0yJFJCQ25vVE5GXzJoX21lYW4KYmFzZU1lYW5zSG0yJFZhcjE0X1JCQ182aDwtYmFzZU1lYW5zSG0yJFZhcjE0bm9UTkZfNmhfbWVhbi1iYXNlTWVhbnNIbTIkUkJDbm9UTkZfNmhfbWVhbgpiYXNlTWVhbnNIbTIkVmFyMTRfUkJDXzIwaDwtYmFzZU1lYW5zSG0yJFZhcjE0bm9UTkZfMjBoX21lYW4tYmFzZU1lYW5zSG0yJFJCQ25vVE5GXzIwaF9tZWFuCmJhc2VNZWFuc0htIDwtYmFzZU1lYW5zSG0yWyxjKDk6MTIpXQpoZWFkKGJhc2VNZWFuc0htKQpiYXNlTWVhbnNIbU0gPC1iYXNlTWVhbnNIbTJbLGMoMTo4KV0KaGVhZChiYXNlTWVhbnNIbU0pCmBgYAoKCmBgYHtyfQp0b3BERWdlbmVzIDwtIHdoaWNoKHRlbXBBJEluY2x1ZGU9PSJpbiIpIyMjI2ZpbmQgaW5kZXhlcyAKYGBgCgoKIyMjIzIuIEhpZXJhY2hpY2FsIGNsdXN0ZXJpbmcgb2YgbWVhbnMgKGluZGl2aWR1YWwgc2FtcGxlcyBhZGRlZCBmb3IgaW5zcGVjdGlvbikKCmBgYHtyLGZpZy5oZWlnaHQgPSAxMCxmaWcud2lkdGggPSAyMH0KIyMjI21lYW4gbG9nZmMKZGF0YUhNbTwtYmFzZU1lYW5zSG1bIHRvcERFZ2VuZXMsIF0KI2RhdGFITW0gPC0gbG9nMihkYXRhSE1tKzEpCmRhdGFITW08LSB0KGFzLm1hdHJpeChkYXRhSE1tKSkKZGF0YUhNbSA8LSB0KHNjYWxlKGRhdGFITW0pKQoKaG1hcF9oaWVyX2ZhY3RvcnM0IDwtIEhlYXRtYXAoCiAgZGF0YUhNbSwgIG5hbWUgPSAibG9nZmMiLAogIHJvd19sYWJlbHMgPSBwYXN0ZTAocm93bmFtZXMoZGF0YUhNbSksIiAiLCh0ZW1wQVsgdG9wREVnZW5lcywgXSkkR2VuZV9TeW1ib2wpLAogIGNvbHVtbl90aXRsZSA9IHBhc3RlMCgiTWVhbnMgbG9nZmMiKSwgCiAgY29sID0gY29sX2Z1bkdSLAogIGNvbHVtbl90aXRsZV9ncCA9IGdwYXIoZm9udHNpemUgPSAxNiwgZm9udGZhY2UgPSAiYm9sZCIpLAogIHdpZHRoID0gdW5pdCg1MCwgIm1tIiksCiAgY2x1c3Rlcl9jb2x1bW5zID0gRkFMU0UsCiAgc2hvd19yb3dfbmFtZXMgPSBGQUxTRSkKCmRhdGFITW1QbG90PC1hcy5kYXRhLmZyYW1lKGRhdGFITW0pCmRhdGFITW1QbG90JFZhcjE0X1JCQ18yaDwtZGF0YUhNbVBsb3QkVmFyMTRfUkJDXzJoLWRhdGFITW1QbG90JFZhcjE0X1JCQ18waApkYXRhSE1tUGxvdCRWYXIxNF9SQkNfNmg8LWRhdGFITW1QbG90JFZhcjE0X1JCQ182aC1kYXRhSE1tUGxvdCRWYXIxNF9SQkNfMGgKZGF0YUhNbVBsb3QkVmFyMTRfUkJDXzIwaDwtZGF0YUhNbVBsb3QkVmFyMTRfUkJDXzIwaC1kYXRhSE1tUGxvdCRWYXIxNF9SQkNfMGgKZGF0YUhNbVBsb3QkVmFyMTRfUkJDXzBoPC1kYXRhSE1tUGxvdCRWYXIxNF9SQkNfMGgtZGF0YUhNbVBsb3QkVmFyMTRfUkJDXzBoCgpkYXRhSE1tUGxvdDwtYXMubWF0cml4KGRhdGFITW1QbG90KQogIApobWFwX2hpZXJfZmFjdG9yczYgPC0gSGVhdG1hcCgKICBkYXRhSE1tUGxvdCwgIG5hbWUgPSAiTm9ybWFsaXNlZCBsb2dmYyIsCiAgcm93X2xhYmVscyA9IHBhc3RlMChyb3duYW1lcyhkYXRhSE1tKSwiICIsKHRlbXBBWyB0b3BERWdlbmVzLCBdKSRHZW5lX1N5bWJvbCksCiAgY29sdW1uX3RpdGxlID0gcGFzdGUwKCIwaCBOb3JtYWxpc2VkIGxvZ2ZjIiksIAogIGNvbCA9IGNvbF9mdW5HUjIsCiAgY29sdW1uX3RpdGxlX2dwID0gZ3Bhcihmb250c2l6ZSA9IDE2LCBmb250ZmFjZSA9ICJib2xkIiksCiAgd2lkdGggPSB1bml0KDUwLCAibW0iKSwKICBjbHVzdGVyX2NvbHVtbnMgPSBGQUxTRSwKICBzaG93X3Jvd19uYW1lcyA9IEZBTFNFKQoKIyMjI21lYW5zCmRhdGFITW0zPC1iYXNlTWVhbnNIbU1bIHRvcERFZ2VuZXMsIF0KI2RhdGFITW0gPC0gbG9nMihkYXRhSE1tKzEpCmRhdGFITW0zPC0gdChhcy5tYXRyaXgoZGF0YUhNbTMpKQpkYXRhSE1tMyA8LSB0KHNjYWxlKGRhdGFITW0zKSkKZGF0YUhNbTM8LWRhdGFITW0zWyxjKDUsMSw2LDIsNywzLDgsNCldCmhtYXBfaGllcl9mYWN0b3JzNSA8LSBIZWF0bWFwKAogIGRhdGFITW0zLCAgbmFtZSA9ICJFeHByZXNzaW9uIiwKICByb3dfbGFiZWxzID0gcGFzdGUwKHJvd25hbWVzKGRhdGFITW0pLCIgIiwodGVtcEFbIHRvcERFZ2VuZXMsIF0pJEdlbmVfU3ltYm9sKSwKICBjb2x1bW5fdGl0bGUgPSBwYXN0ZTAoIk1lYW5zIiksIAogIGNvbCA9IGNvbF9mdW4sCiAgY29sdW1uX3RpdGxlX2dwID0gZ3Bhcihmb250c2l6ZSA9IDE2LCBmb250ZmFjZSA9ICJib2xkIiksCiAgd2lkdGggPSB1bml0KDEwMCwgIm1tIiksCiAgY2x1c3Rlcl9jb2x1bW5zID0gRkFMU0UsCiAgc2hvd19yb3dfbmFtZXMgPSBGQUxTRSkKICAKaG1hcF9oaWVyX2ZhY3RvcnM0K2htYXBfaGllcl9mYWN0b3JzNitobWFwX2hpZXJfZmFjdG9yczUKCmBgYAoKCmBgYHtyLGZpZy5oZWlnaHQgPSA3LGZpZy53aWR0aCA9MTIgfQpwYXIobWZyb3c9YygxLDIpKQojIyMjIFNpbGhvdWV0dGUgbWV0aG9kCmZ2aXpfbmJjbHVzdChkYXRhSE1tLCBrbWVhbnMsIG1ldGhvZCA9ICJzaWxob3VldHRlIixrLm1heCA9IDE2KSsKICBsYWJzKHN1YnRpdGxlID0gIlNpbGhvdWV0dGUgbWV0aG9kIikKCiMjIyMgRWxib3cgbWV0aG9kCmZ2aXpfbmJjbHVzdChkYXRhSE1tLCBrbWVhbnMsIG1ldGhvZCA9ICJ3c3MiLGsubWF4ID0gMTYpICsKICBsYWJzKHN1YnRpdGxlID0gIkVsYm93IG1ldGhvZCIpCgpgYGAKCmBgYHtyfQojIyMjZ2FwIHN0YXQgc2xvdyEhIQojIyMjc2V0LnNlZWQoMTIzKQojIyMjZnZpel9uYmNsdXN0KGRhdGFITW0sIGttZWFucywgbnN0YXJ0ID0gMjUsICBtZXRob2QgPSAiZ2FwX3N0YXQiLCBuYm9vdCA9IDEwMCxrLm1heCA9IDE2KSsKIyMjIyAgbGFicyhzdWJ0aXRsZSA9ICJHYXAgc3RhdGlzdGljIG1ldGhvZCIpCmBgYAoKCmBgYHtyLGZpZy5oZWlnaHQgPSA3fQoja2NsdXN0NCA8LSBrbWVhbnMoZGF0YUhNbSwgNSkKI3NpbGhvdWV0dGUgcGxvdApkaXN0SzwtZGFpc3koZGF0YUhNbSkKcGxvdChzaWxob3VldHRlKGtjbHVzdDQkY2x1c3RlciwgZGlzdEspLCBjb2w9MTo1LCBib3JkZXI9TkEpCgpgYGAKCiMjIyMzLiBLLW1lYW5zIGNsdXN0ZXJpbmcgb2YgbWVhbnMKCmBgYHtyLGZpZy5oZWlnaHQgPSAxMCxmaWcud2lkdGggPSAyMH0Kc3BsaXQgPC0gcGFzdGUwKCJDbHVzdGVyXG4iLCBrY2x1c3Q0JGNsdXN0ZXIpCiNzcGxpdCA8LSBmYWN0b3IocGFzdGUwKCJDbHVzdGVyXG4iLCBrY2x1c3QzJGNsdXN0ZXIpLCBsZXZlbHM9YygiQ2x1c3RlclxuMyIsIkNsdXN0ZXJcbjEiLCJDbHVzdGVyXG40IiwiQ2x1c3RlclxuNSIsIkNsdXN0ZXJcbjIiLCJDbHVzdGVyXG42IikpCmhtYXBfayA8LSBIZWF0bWFwKGRhdGFITW0sIHNwbGl0PXNwbGl0LCBjbHVzdGVyX3Jvd19zbGljZXMgPSBGQUxTRSwKICAgICAgICAgICAgICAgICAgY2x1c3Rlcl9jb2x1bW5zID0gRkFMU0UsCiAgICAgICAgICAgICAgICAgIHNob3dfcm93X25hbWVzID0gRkFMU0UsCiAgICAgICAgICAgICAgICAgIG5hbWUgPSAibG9nZmMiLAogICAgICAgICAgICAgICAgICBjb2wgPSBjb2xfZnVuR1IsCiAgICAgICAgICAgICAgICAgIHdpZHRoID0gdW5pdCg1MCwgIm1tIiksCiAgICAgICAgICAgICAgICAgIGNvbHVtbl90aXRsZSA9ICJtZWFucyBsb2dmYyIsIAogICAgICAgICAgICAgICAgICBjb2x1bW5fdGl0bGVfZ3AgPSBncGFyKGZvbnRzaXplID0gMTYsIGZvbnRmYWNlID0gImJvbGQiKSkKCmhtYXBfaytobWFwX2hpZXJfZmFjdG9yczYraG1hcF9oaWVyX2ZhY3RvcnM1CmBgYAoKCk1lYW4gcHJvZmlsZXMgb2YgY2x1c3RlcnMKCmBgYHtyLGZpZy5oZWlnaHQgPSA4LGZpZy53aWR0aCA9IDh9CmNsdXN0ZXJjb3VudDwtZGF0YS5mcmFtZShrY2x1c3Q0JGNsdXN0ZXIpCmNsdXN0ZXJzaXplczwtdGFibGUoY2x1c3RlcmNvdW50JGtjbHVzdDQuY2x1c3RlcikKY2x1c3Rlck1lYW5zPC1kYXRhLmZyYW1lKGtjbHVzdDQkY2VudGVycykKY2x1c3Rlck1lYW5zMTwtZGF0YS5mcmFtZSh0KGNsdXN0ZXJNZWFucykpCmNsdXN0ZXJNZWFuczEgPC0gY2JpbmQocm93bmFtZXMoY2x1c3Rlck1lYW5zMSksIGNsdXN0ZXJNZWFuczEpCm9yZGVyTjwtYygiVmFyMTRfUkJDXzBoIiwiVmFyMTRfUkJDXzJoIiwiVmFyMTRfUkJDXzZoIiwiVmFyMTRfUkJDXzIwaCIpIyMjIyBtYW51YWwKCnJvd25hbWVzKGNsdXN0ZXJNZWFuczEpIDwtIE5VTEwKbmFtZXMoY2x1c3Rlck1lYW5zMSlbbmFtZXMoY2x1c3Rlck1lYW5zMSk9PSJyb3duYW1lcyhjbHVzdGVyTWVhbnMxKSJdIDwtICJTYW1wbGUiCiMjIyNjbHVzdGVyTWVhbnMxCgpwWDE8LWdncGxvdChkYXRhPWNsdXN0ZXJNZWFuczEsIGFlcyh4PVNhbXBsZSwgeT1YMSxncm91cD0xKSkgKwogIGdlb21fbGluZSgpKyAgZ2VvbV9wb2ludCgpK2dndGl0bGUocGFzdGUoIkNsdXN0ZXIgWDEgUHJvZmlsZSAiLGNsdXN0ZXJzaXplc1sxXSwiIGdlbmVzIikpKyAgc2NhbGVfeF9kaXNjcmV0ZShsaW1pdHM9b3JkZXJOKSsKICB0aGVtZShheGlzLnRpdGxlLnggPSBlbGVtZW50X2JsYW5rKCksYXhpcy50aXRsZS55ID0gZWxlbWVudF9ibGFuaygpKQpwWDI8LWdncGxvdChkYXRhPWNsdXN0ZXJNZWFuczEsIGFlcyh4PVNhbXBsZSwgeT1YMixncm91cD0xKSkgKwogIGdlb21fbGluZSgpKyAgZ2VvbV9wb2ludCgpK2dndGl0bGUocGFzdGUoIkNsdXN0ZXIgWDIgUHJvZmlsZSAiLGNsdXN0ZXJzaXplc1syXSwiIGdlbmVzIikpKyAgc2NhbGVfeF9kaXNjcmV0ZShsaW1pdHM9b3JkZXJOKSsKICB0aGVtZShheGlzLnRpdGxlLnggPSBlbGVtZW50X2JsYW5rKCksYXhpcy50aXRsZS55ID0gZWxlbWVudF9ibGFuaygpKQpwWDM8LWdncGxvdChkYXRhPWNsdXN0ZXJNZWFuczEsIGFlcyh4PVNhbXBsZSwgeT1YMyxncm91cD0xKSkgKwogIGdlb21fbGluZSgpKyAgZ2VvbV9wb2ludCgpK2dndGl0bGUocGFzdGUoIkNsdXN0ZXIgWDMgUHJvZmlsZSAiLGNsdXN0ZXJzaXplc1szXSwiIGdlbmVzIikpKyAgc2NhbGVfeF9kaXNjcmV0ZShsaW1pdHM9b3JkZXJOKSsKICB0aGVtZShheGlzLnRpdGxlLnggPSBlbGVtZW50X2JsYW5rKCksYXhpcy50aXRsZS55ID0gZWxlbWVudF9ibGFuaygpKQpwWDQ8LWdncGxvdChkYXRhPWNsdXN0ZXJNZWFuczEsIGFlcyh4PVNhbXBsZSwgeT1YNCxncm91cD0xKSkgKwogIGdlb21fbGluZSgpKyAgZ2VvbV9wb2ludCgpK2dndGl0bGUocGFzdGUoIkNsdXN0ZXIgWDQgUHJvZmlsZSAiLGNsdXN0ZXJzaXplc1s0XSwiIGdlbmVzIikpKyAgc2NhbGVfeF9kaXNjcmV0ZShsaW1pdHM9b3JkZXJOKSsKICB0aGVtZShheGlzLnRpdGxlLnggPSBlbGVtZW50X2JsYW5rKCksYXhpcy50aXRsZS55ID0gZWxlbWVudF9ibGFuaygpKQpwWDU8LWdncGxvdChkYXRhPWNsdXN0ZXJNZWFuczEsIGFlcyh4PVNhbXBsZSwgeT1YNSxncm91cD0xKSkgKwogIGdlb21fbGluZSgpKyAgZ2VvbV9wb2ludCgpK2dndGl0bGUocGFzdGUoIkNsdXN0ZXIgWDUgUHJvZmlsZSAiLGNsdXN0ZXJzaXplc1s1XSwiIGdlbmVzIikpKyAgc2NhbGVfeF9kaXNjcmV0ZShsaW1pdHM9b3JkZXJOKSsKICB0aGVtZShheGlzLnRpdGxlLnggPSBlbGVtZW50X2JsYW5rKCksYXhpcy50aXRsZS55ID0gZWxlbWVudF9ibGFuaygpKQojcFg2PC1nZ3Bsb3QoZGF0YT1jbHVzdGVyTWVhbnMxLCBhZXMoeD1TYW1wbGUsIHk9WDYsZ3JvdXA9MSkpICsKIyAgZ2VvbV9saW5lKCkrICBnZW9tX3BvaW50KCkrZ2d0aXRsZShwYXN0ZSgiQ2x1c3RlciBYNiBQcm9maWxlICIsY2x1c3RlcnNpemVzWzZdLCIgZ2VuZXMiKSkrICBzY2FsZV94X2Rpc2NyZXRlKGxpbWl0cz1vcmRlck4pKwojICB0aGVtZShheGlzLnRpdGxlLnggPSBlbGVtZW50X2JsYW5rKCksYXhpcy50aXRsZS55ID0gZWxlbWVudF9ibGFuaygpKQoKCiNwbG90Cm11bHRpcGxvdChwWDEsIHBYMiwgcFgzLHBYNCwgcFg1LCAgY29scz0yKQoKYGBgCgoKYGBge3J9CnRvcERFZ2VuZXMgPC0gd2hpY2godGVtcEEkSW5jbHVkZT09ImluIikjIyMjZmluZCBpbmRleGVzCnRlbXBBa208LXRlbXBBWyB0b3BERWdlbmVzLCBdClN5bWJvbHNLbTwtZHBseXI6OnB1bGwodGVtcEFrbSwgR2VuZV9TeW1ib2wpCiMjIyMgZXhwb3J0IHRoZSBnZW5lIGV4cHJlc3Npb24gZGF0YSBmb3IgdGhlIGNsdXN0ZXJzCndyaXRlLnRhYmxlKGNsdXN0ZXJNZWFucyxwYXN0ZTAoIkNsdXN0ZXJNZWFuc0ttXyIsZ3JvdXBzTmFtZSwiLnR4dCIpLCAgc2VwID0gIlx0IikKQ2x1c3RlcmVkR2VuZXM8LWRhdGEuZnJhbWUoa2NsdXN0NCRjbHVzdGVyLFN5bWJvbHNLbSxkYXRhSE1tKQp3cml0ZS50YWJsZShDbHVzdGVyZWRHZW5lcyxwYXN0ZTAoIlNjYWxlZERhdGFJbkNsdXN0ZXJzS21fIixncm91cHNOYW1lLCIudHh0IiksICBzZXAgPSAiXHQiKQojaGVhZChDbHVzdGVyZWRHZW5lcykKYGBgCgoKYGBge3J9CmJvdHRvbURFZ2VuZXM8LXdoaWNoKHRlbXBBJEluY2x1ZGU9PSJvdXQiKSMjIyNmaW5kIGluZGV4ZXMgCmJvdHRvbUc8LXRlbXBBWyBib3R0b21ERWdlbmVzLCBdCmJvdHRvbUc8LWRwbHlyOjpwdWxsKGJvdHRvbUcsIEdlbmVfU3ltYm9sKQp3cml0ZS50YWJsZShib3R0b21HLHBhc3RlMCgiaXBhQm90dG9tS21lYW5zXyIsZ3JvdXBzTmFtZSwiLnR4dCIpLCAgc2VwID0gIlx0IikKICAgICAgICAgICAgICAgICAgICAgICAgIAoKdG9wREVnZW5lcyA8LSB3aGljaCh0ZW1wQSRJbmNsdWRlPT0iaW4iKSMjIyNmaW5kIGluZGV4ZXMgCnRlbXBBa208LXRlbXBBWyB0b3BERWdlbmVzLCBdClN5bWJvbHNLbTwtZHBseXI6OnB1bGwodGVtcEFrbSwgR2VuZV9TeW1ib2wpCgppcGFLbWVhbnM8LUNsdXN0ZXJlZEdlbmVzCiNjb3VudHNUYWJsZSA8LWNvdW50c1RhYmxlWyxjKDE6MTUpXSMjIyNpZiBzYW1wbGVzIG5lZWQgcmVtb3ZpbmcKaXBhS21lYW5zPC1pcGFLbWVhbnNbLGMoMToyKV0KaXBhS21lYW5zJG5hbWUyPC1yb3duYW1lcyhpcGFLbWVhbnMpCiNpcGFLbWVhbnMlPiUgcm93bmFtZXNfdG9fY29sdW1uKHZhciA9ICJyb3duYW1lIikKI2lwYUttZWFucwojcm93aWRfdG9fY29sdW1uKGlwYUttZWFucykKaXBhS21lYW5zID0gbXV0YXRlKGlwYUttZWFucywgeDE9IGlmZWxzZShpcGFLbWVhbnMka2NsdXN0NC5jbHVzdGVyPT0xLCAiMSIsICIwIikpCmlwYUttZWFucyA9IG11dGF0ZShpcGFLbWVhbnMsIHgyPSBpZmVsc2UoaXBhS21lYW5zJGtjbHVzdDQuY2x1c3Rlcj09MiwgIjEiLCAiMCIpKQppcGFLbWVhbnMgPSBtdXRhdGUoaXBhS21lYW5zLCB4Mz0gaWZlbHNlKGlwYUttZWFucyRrY2x1c3Q0LmNsdXN0ZXI9PTMsICIxIiwgIjAiKSkKaXBhS21lYW5zID0gbXV0YXRlKGlwYUttZWFucywgeDQ9IGlmZWxzZShpcGFLbWVhbnMka2NsdXN0NC5jbHVzdGVyPT00LCAiMSIsICIwIikpCmlwYUttZWFucyA9IG11dGF0ZShpcGFLbWVhbnMsIHg1PSBpZmVsc2UoaXBhS21lYW5zJGtjbHVzdDQuY2x1c3Rlcj09NSwgIjEiLCAiMCIpKQojaXBhS21lYW5zID0gbXV0YXRlKGlwYUttZWFucywgeDY9IGlmZWxzZShpcGFLbWVhbnMka2NsdXN0NC5jbHVzdGVyPT02LCAiMSIsICIwIikpCiNpcGFLbWVhbnMKd3JpdGUudGFibGUoaXBhS21lYW5zLHBhc3RlMCgiaXBhS21lYW5zXyIsZ3JvdXBzTmFtZSwiLnR4dCIpLCAgc2VwID0gIlx0IikKI2hlYWQoaXBhS21lYW5zKQoKYGBgCgoKYGBge3J9CkNsdXN0ZXJlZEdlbmVzMjwtQ2x1c3RlcmVkR2VuZXNbYygxKV0KI0NsdXN0ZXJlZEdlbmVzMgpsaXN0QWxsPC1saXN0KCkKZm9yKGkgaW4gMTo1KSB7CiAgY2x1c3Rlck5hbWU8LXBhc3RlMCgieCIsaSkKICAjY2x1c3Rlck5hbWU8LXJvdy5uYW1lcyhzdWJzZXQoQ2x1c3RlcmVkR2VuZXMsQ2x1c3RlcmVkR2VuZXM9PWkpKQogIGNsdXN0ZXJOYW1lPC0oc3Vic2V0KENsdXN0ZXJlZEdlbmVzJFN5bWJvbHNLbSxDbHVzdGVyZWRHZW5lcz09aSkpCiAgbGlzdEFsbFtbaV1dPC1jbHVzdGVyTmFtZQp9CiNuZWVkIHRvIG5hbWUgdGhlIHZlY3RvcnMgaW4gdGhlIGxpc3QsIGV4YW1wbGUgaGVyZSBpcyBmb3IgOCBjbHVzdGVycwpuYW1lcyhsaXN0QWxsKTwtYygiWDEiLCAiWDIiLCAiWDMiLCJYNCIsICJYNSIpCgojaWYgeW91IHdhbnQgdG8gcmVhcnJhbmdlIHRoZSBvcmRlcgojbGlzdEFsbDwtbGlzdEFsbFtjKCJ4MyIsICJ4NyIsICJ4OCIsICJ4MiIsICJ4NiIsICJ4NSIsICJ4NCIsICJ4MSIpXQoKbGFwcGx5KGxpc3RBbGwsIGhlYWQpCgpgYGAKCiMjIyM0LiBBbm5vdGF0aW9uIG9mIEstbWVhbnMgY2x1c3RlcnMKLSBDQyBjZWxsdWxhciBjb21wYXJ0bWVudAotIEJQIGJpb2xvZ2ljYWwgcHJvY2VzcwotIE1GIG1vbGVjdWxhciBmdW5jdGlvbgoKVGhlIHNpbXBsaWZ5IGZ1bmN0aW9uIGhhcyBiZWVuIHVzZWQgdG8gY3V0IGRvd24gb24gR08gcmVkdW5kYW5jeQoKYGBge3J9CiNzdHIoQWxsR2VuZU5hbWVzKQpgYGAKCgpgYGB7cixmaWcuaGVpZ2h0ID0gOCxmaWcud2lkdGggPSAxMn0KIyMjI0NDCmNnb0NDIDwtIGNvbXBhcmVDbHVzdGVyKGdlbmVDbHVzdGVyID0gbGlzdEFsbCwgCiAgICAgICAgICAgICAgICAgICAgICB1bml2ZXJzZSA9IEFsbEdlbmVOYW1lcywKICAgICAgICAgICAgICAgICAgICAgIGZ1biA9ICJlbnJpY2hHTyIsCiAgICAgICAgICAgICAgICAgICAgICBPcmdEYj1vcmcuSHMuZWcuZGIsIAogICAgICAgICAgICAgICAgICAgICAgIyMjI09yZ0RiPW9yZy5NbS5lZy5kYiwKICAgICAgICAgICAgICAgICAgICAgIGtleVR5cGU9IlNZTUJPTCIsCiAgICAgICAgICAgICAgICAgICAgICBvbnQgPSAiQ0MiLCAKICAgICAgICAgICAgICAgICAgICAgIHB2YWx1ZUN1dG9mZj0wLjA1LAogICAgICAgICAgICAgICAgICAgICAgcXZhbHVlQ3V0b2ZmID0gMC4xMCkKY2dvQ0MyIDwtIHNpbXBsaWZ5KGNnb0NDLCBjdXRvZmY9MC43LCBieT0icC5hZGp1c3QiLCBzZWxlY3RfZnVuPW1pbikKIyMjI3dyaXRlIGFzIHNwcmVhZHNoZWV0CndyaXRlLmNzdihhcy5kYXRhLmZyYW1lKGNnb0NDMikscGFzdGUwKCJHT19DQ18iLGdyb3Vwc05hbWUsIi5jc3YiKSkKCmRvdHBsb3QoY2dvQ0MyLHNob3dDYXRlZ29yeSA9IDMwLAogICAgICAgIHRpdGxlID0gcGFzdGUwKCJHTyBDZWxsdWxhciBDb21wYXJ0bWVudCAiLGdyb3Vwc05hbWUpKSsKICB0aGVtZShheGlzLnRleHQueCA9IGVsZW1lbnRfdGV4dChhbmdsZSA9IDkwLCB2anVzdCA9IDAuNSwgaGp1c3Q9MSkpCgpgYGAKUGxvdHMgYW5kIEdPIGRhdGEgd2VyZSB3cml0dGVuIHRvIGZpbGVzCmBgYHtyfQpwbmcocGFzdGUwKCJHT19DQ18iLGdyb3Vwc05hbWUsIi5wbmciKSwgd2lkdGggPSAxMjI0LCBoZWlnaHQgPSA4MjQpCmRvdHBsb3QoY2dvQ0MyLHNob3dDYXRlZ29yeSA9IDMwLAogICAgICAgIHRpdGxlID0gcGFzdGUwKCJHTyBDZWxsdWxhciBDb21wYXJ0bWVudCAiLGdyb3Vwc05hbWUpKSsKICB0aGVtZShheGlzLnRleHQueCA9IGVsZW1lbnRfdGV4dChhbmdsZSA9IDkwLCB2anVzdCA9IDAuNSwgaGp1c3Q9MSkpCmRldi5vZmYoKQpgYGAKCkdPIEJQCmBgYHtyLGZpZy5oZWlnaHQgPSAxMixmaWcud2lkdGggPSAxMn0KIyMjI0NDCmNnb0JQIDwtIGNvbXBhcmVDbHVzdGVyKGdlbmVDbHVzdGVyID0gbGlzdEFsbCwgCiAgICAgICAgICAgICAgICAgICAgICB1bml2ZXJzZSA9IEFsbEdlbmVOYW1lcywKICAgICAgICAgICAgICAgICAgICAgIGZ1biA9ICJlbnJpY2hHTyIsCiAgICAgICAgICAgICAgICAgICAgICBPcmdEYj1vcmcuSHMuZWcuZGIsCiAgICAgICAgICAgICAgICAgICAgICBrZXlUeXBlPSJTWU1CT0wiLAogICAgICAgICAgICAgICAgICAgICAgb250ID0gIkJQIiwgCiAgICAgICAgICAgICAgICAgICAgICBwdmFsdWVDdXRvZmY9MC4wNSwKICAgICAgICAgICAgICAgICAgICAgIHF2YWx1ZUN1dG9mZiA9IDAuMTApCmNnb0JQMiA8LSBzaW1wbGlmeShjZ29CUCwgY3V0b2ZmPTAuNywgYnk9InAuYWRqdXN0Iiwgc2VsZWN0X2Z1bj1taW4pCiMjIyN3cml0ZSBhcyBzcHJlYWRzaGVldAp3cml0ZS5jc3YoYXMuZGF0YS5mcmFtZShjZ29CUDIpLHBhc3RlMCgiR09fQlBfIixncm91cHNOYW1lLCIuY3N2IikpCgpkb3RwbG90KGNnb0JQMixzaG93Q2F0ZWdvcnkgPSAzMCwKICAgICAgICB0aXRsZSA9IHBhc3RlMCgiR08gQmlvbG9naWNhbCBQcm9jZXNzICIsZ3JvdXBzTmFtZSkpKwogIHRoZW1lKGF4aXMudGV4dC54ID0gZWxlbWVudF90ZXh0KGFuZ2xlID0gOTAsIHZqdXN0ID0gMC41LCBoanVzdD0xKSkKCmBgYAoKYGBge3J9CnBuZyhwYXN0ZTAoIkdPX0JQXyIsZ3JvdXBzTmFtZSwiLnBuZyIpLCB3aWR0aCA9IDEwMjQsIGhlaWdodCA9IDEyMjQpCmRvdHBsb3QoY2dvQlAyLHNob3dDYXRlZ29yeSA9IDMwLAogICAgICAgIHRpdGxlID0gcGFzdGUwKCJHTyBCaW9sb2dpY2FsIFByb2Nlc3MgIixncm91cHNOYW1lKSkrCiAgdGhlbWUoYXhpcy50ZXh0LnggPSBlbGVtZW50X3RleHQoYW5nbGUgPSA5MCwgdmp1c3QgPSAwLjUsIGhqdXN0PTEpKQpkZXYub2ZmKCkKYGBgCgoKR08gTUYKYGBge3IsZmlnLmhlaWdodCA9IDEwLGZpZy53aWR0aCA9IDEyfQojIyMjTUYKY2dvTUYgPC0gY29tcGFyZUNsdXN0ZXIoZ2VuZUNsdXN0ZXIgPSBsaXN0QWxsLCAKICAgICAgICAgICAgICAgICAgICAgIHVuaXZlcnNlID0gQWxsR2VuZU5hbWVzLAogICAgICAgICAgICAgICAgICAgICAgZnVuID0gImVucmljaEdPIiwKICAgICAgICAgICAgICAgICAgICAgIE9yZ0RiPW9yZy5Icy5lZy5kYiwgCiAgICAgICAgICAgICAgICAgICAgICBrZXlUeXBlPSJTWU1CT0wiLAogICAgICAgICAgICAgICAgICAgICAgb250ID0gIk1GIiwgCiAgICAgICAgICAgICAgICAgICAgICBwdmFsdWVDdXRvZmY9MC4wNSwKICAgICAgICAgICAgICAgICAgICAgIHF2YWx1ZUN1dG9mZiA9IDAuMTApCmNnb01GMiA8LSBzaW1wbGlmeShjZ29NRiwgY3V0b2ZmPTAuNywgYnk9InAuYWRqdXN0Iiwgc2VsZWN0X2Z1bj1taW4pCiMjIyN3cml0ZSBhcyBzcHJlYWRzaGVldAp3cml0ZS5jc3YoYXMuZGF0YS5mcmFtZShjZ29NRjIpLHBhc3RlMCgiR09fTUZfIixncm91cHNOYW1lLCIuY3N2IikpCgpkb3RwbG90KGNnb01GMixzaG93Q2F0ZWdvcnkgPSAzMCwKICAgICAgICB0aXRsZSA9IHBhc3RlMCgiR08gTW9sZWN1bGFyIEZ1bmN0aW9uICAiLGdyb3Vwc05hbWUpKSsKICB0aGVtZShheGlzLnRleHQueCA9IGVsZW1lbnRfdGV4dChhbmdsZSA9IDkwLCB2anVzdCA9IDAuNSwgaGp1c3Q9MSkpCgpgYGAKCgoKYGBge3J9CnBuZyhwYXN0ZTAoIkdPX01GXyIsZ3JvdXBzTmFtZSwiLnBuZyIpLCB3aWR0aCA9IDE0MjQsIGhlaWdodCA9IDgyNCkKZG90cGxvdChjZ29NRjIsc2hvd0NhdGVnb3J5ID0gMzAsCiAgICAgICAgdGl0bGUgPSBwYXN0ZTAoIkdPIE1vbGVjdWxhciBGdW5jdGlvbiAgIixncm91cHNOYW1lKSkrCiAgdGhlbWUoYXhpcy50ZXh0LnggPSBlbGVtZW50X3RleHQoYW5nbGUgPSA5MCwgdmp1c3QgPSAwLjUsIGhqdXN0PTEpKQpkZXYub2ZmKCkKYGBgCgoKIyMjIFIzIFZBUjE0IHZzIFJCQyBubyBUTkYgay1tZWFucyBwYWRqMC4xZmMyCgojIyMjMS4gR2VuZWxpc3QgU2VsZWN0aW9uCgoKYGBge3J9Cmdyb3Vwc05hbWU8LSJSM19WQVIxNF9rbWVhbnNfcGFkajAuMWZjMiIKYGBgCgoKYGBge3J9CmNvdW50c1RhYmxlPC1yZWFkLmRlbGltKCJSTkFzZXEyMDE5SnVseV81LnR4dCIsIGhlYWRlciA9IFRSVUUsIHNlcCA9ICJcdCIsY2hlY2submFtZXM9RkFMU0Uscm93Lm5hbWVzPTEpCmhlYWQoY291bnRzVGFibGUpCmBgYAoKYGBge3J9CkFsbEdlbmVOYW1lczwtY291bnRzVGFibGUkR2VuZV9TeW1ib2wKI2hlYWQoQWxsR2VuZU5hbWVzKQpgYGAKCmBgYHtyLGZpZy5oZWlnaHQgPSAxMCxmaWcud2lkdGggPSAyMH0KZ3JpZC5hcnJhbmdlKGdUcmVlKGNoaWxkcmVuPXZlbm5wKSwgZ1RyZWUoY2hpbGRyZW49dmVubnBxKSAsIG5jb2w9Mix0b3A9IlIzIFZhcjE0IG5vIFRORiIpCmBgYAoKYGBge3J9CiN0ZW1wQTwtcmVzQWxsWy1jKDEwOjMwKSBdCnRlbXBBPC1jb3VudHNUYWJsZQojcm93bmFtZXModGVtcEEpCnJvd25hbWVzKHRlbXBBKSA8LSBOVUxMCnRlbXBBID0gbXV0YXRlKHRlbXBBLCBJbmNsdWRlPQogICAgICAgICAgICAgICAgICAgaWZlbHNlKHRlbXBBJHBhZGpfUjNub1RORl92YXIxNF92c19SQkNfMGg8MC4xJmFicyh0ZW1wQSRsb2cyRm9sZENoYW5nZV9SM25vVE5GX3ZhcjE0X3ZzX1JCQ18waCk+MSYhaXMubmEodGVtcEEkcGFkal9SM25vVE5GX3ZhcjE0X3ZzX1JCQ18waCksICJpbiIsCiAgICAgICAgICAgICAgICAgICAgICAgICAgaWZlbHNlKHRlbXBBJHBhZGpfUjNub1RORl92YXIxNF92c19SQkNfMmg8MC4xJmFicyh0ZW1wQSRsb2cyRm9sZENoYW5nZV9SM25vVE5GX3ZhcjE0X3ZzX1JCQ18yaCk+MSYhaXMubmEodGVtcEEkcGFkal9SM25vVE5GX3ZhcjE0X3ZzX1JCQ18yaCksICJpbiIsCiAgICAgICAgICAgICAgICAgICAgICAgICAgICAgICAgIGlmZWxzZSh0ZW1wQSRwYWRqX1Izbm9UTkZfdmFyMTRfdnNfUkJDXzZoPDAuMSZhYnModGVtcEEkbG9nMkZvbGRDaGFuZ2VfUjNub1RORl92YXIxNF92c19SQkNfNmgpPjEmIWlzLm5hKHRlbXBBJHBhZGpfUjNub1RORl92YXIxNF92c19SQkNfNmgpLCAiaW4iLAogICAgICAgICAgICAgICAgICAgICAgICAgICAgICAgICAgICAgICAgaWZlbHNlKHRlbXBBJHBhZGpfUjNub1RORl92YXIxNF92c19SQkNfMjBoPDAuMSZhYnModGVtcEEkbG9nMkZvbGRDaGFuZ2VfUjNub1RORl92YXIxNF92c19SQkNfMjBoKT4xJiFpcy5uYSh0ZW1wQSRwYWRqX1Izbm9UTkZfdmFyMTRfdnNfUkJDXzIwaCksICJpbiIsCiAgICAgICAgICAgICAgICAgICAgICAgICAgICAgICAgICAgICAgICAgICAgICAgICAgICAgICAgICAgICAgICAgICAgICAgIm91dCIpKSkpKQoKCiN0ZW1wQQojIyMjbGlicmFyeShkcGx5cikKdGVtcEEgJT4lCiAgICAgZ3JvdXBfYnkoSW5jbHVkZSkgJT4lIAogICAgIHRhbGx5KCkKCmBgYAoKYGBge3J9CnRvcERFZ2VuZXMgPC0gd2hpY2godGVtcEEkSW5jbHVkZT09ImluIikjIyMjZmluZCBpbmRleGVzIApgYGAKCgoKIyMjIyBOQiBQbGVhc2UgY2hlY2sgY29sdW1ucyB1c2VkIGFuZCByZW5hbWVkIGZvciBwbG90cwpgYGB7cn0KYmFzZU1lYW5zSG0gPC1jb3VudHNUYWJsZVssYyg2MDo2Myw3OTo4MildCmJhc2VNZWFuc0htMiA8LSBsb2cyKGJhc2VNZWFuc0htKzEpCmJhc2VNZWFuc0htMiRWYXIxNF9SQkNfMGg8LWJhc2VNZWFuc0htMiRWYXIxNG5vVE5GXzBoX21lYW4tYmFzZU1lYW5zSG0yJFJCQ25vVE5GXzBoX21lYW4KYmFzZU1lYW5zSG0yJFZhcjE0X1JCQ18yaDwtYmFzZU1lYW5zSG0yJFZhcjE0bm9UTkZfMmhfbWVhbi1iYXNlTWVhbnNIbTIkUkJDbm9UTkZfMmhfbWVhbgpiYXNlTWVhbnNIbTIkVmFyMTRfUkJDXzZoPC1iYXNlTWVhbnNIbTIkVmFyMTRub1RORl82aF9tZWFuLWJhc2VNZWFuc0htMiRSQkNub1RORl82aF9tZWFuCmJhc2VNZWFuc0htMiRWYXIxNF9SQkNfMjBoPC1iYXNlTWVhbnNIbTIkVmFyMTRub1RORl8yMGhfbWVhbi1iYXNlTWVhbnNIbTIkUkJDbm9UTkZfMjBoX21lYW4KYmFzZU1lYW5zSG0gPC1iYXNlTWVhbnNIbTJbLGMoOToxMildCmhlYWQoYmFzZU1lYW5zSG0pCmJhc2VNZWFuc0htTSA8LWJhc2VNZWFuc0htMlssYygxOjgpXQpoZWFkKGJhc2VNZWFuc0htTSkKYGBgCgoKYGBge3J9CnRvcERFZ2VuZXMgPC0gd2hpY2godGVtcEEkSW5jbHVkZT09ImluIikjIyMjZmluZCBpbmRleGVzIApgYGAKCgojIyMjMi4gSGllcmFjaGljYWwgY2x1c3RlcmluZyBvZiBtZWFucyAoaW5kaXZpZHVhbCBzYW1wbGVzIGFkZGVkIGZvciBpbnNwZWN0aW9uKQoKYGBge3IsZmlnLmhlaWdodCA9IDEwLGZpZy53aWR0aCA9IDIwfQojIyMjbWVhbiBsb2dmYwpkYXRhSE1tPC1iYXNlTWVhbnNIbVsgdG9wREVnZW5lcywgXQojZGF0YUhNbSA8LSBsb2cyKGRhdGFITW0rMSkKZGF0YUhNbTwtIHQoYXMubWF0cml4KGRhdGFITW0pKQpkYXRhSE1tIDwtIHQoc2NhbGUoZGF0YUhNbSkpCgpobWFwX2hpZXJfZmFjdG9yczQgPC0gSGVhdG1hcCgKICBkYXRhSE1tLCAgbmFtZSA9ICJsb2dmYyIsCiAgcm93X2xhYmVscyA9IHBhc3RlMChyb3duYW1lcyhkYXRhSE1tKSwiICIsKHRlbXBBWyB0b3BERWdlbmVzLCBdKSRHZW5lX1N5bWJvbCksCiAgY29sdW1uX3RpdGxlID0gcGFzdGUwKCJNZWFucyBsb2dmYyIpLCAKICBjb2wgPSBjb2xfZnVuR1IsCiAgY29sdW1uX3RpdGxlX2dwID0gZ3Bhcihmb250c2l6ZSA9IDE2LCBmb250ZmFjZSA9ICJib2xkIiksCiAgd2lkdGggPSB1bml0KDUwLCAibW0iKSwKICBjbHVzdGVyX2NvbHVtbnMgPSBGQUxTRSwKICBzaG93X3Jvd19uYW1lcyA9IEZBTFNFKQoKZGF0YUhNbVBsb3Q8LWFzLmRhdGEuZnJhbWUoZGF0YUhNbSkKZGF0YUhNbVBsb3QkVmFyMTRfUkJDXzJoPC1kYXRhSE1tUGxvdCRWYXIxNF9SQkNfMmgtZGF0YUhNbVBsb3QkVmFyMTRfUkJDXzBoCmRhdGFITW1QbG90JFZhcjE0X1JCQ182aDwtZGF0YUhNbVBsb3QkVmFyMTRfUkJDXzZoLWRhdGFITW1QbG90JFZhcjE0X1JCQ18waApkYXRhSE1tUGxvdCRWYXIxNF9SQkNfMjBoPC1kYXRhSE1tUGxvdCRWYXIxNF9SQkNfMjBoLWRhdGFITW1QbG90JFZhcjE0X1JCQ18waApkYXRhSE1tUGxvdCRWYXIxNF9SQkNfMGg8LWRhdGFITW1QbG90JFZhcjE0X1JCQ18waC1kYXRhSE1tUGxvdCRWYXIxNF9SQkNfMGgKCmRhdGFITW1QbG90PC1hcy5tYXRyaXgoZGF0YUhNbVBsb3QpCiAgCmhtYXBfaGllcl9mYWN0b3JzNiA8LSBIZWF0bWFwKAogIGRhdGFITW1QbG90LCAgbmFtZSA9ICJOb3JtYWxpc2VkIGxvZ2ZjIiwKICByb3dfbGFiZWxzID0gcGFzdGUwKHJvd25hbWVzKGRhdGFITW0pLCIgIiwodGVtcEFbIHRvcERFZ2VuZXMsIF0pJEdlbmVfU3ltYm9sKSwKICBjb2x1bW5fdGl0bGUgPSBwYXN0ZTAoIjBoIE5vcm1hbGlzZWQgbG9nZmMiKSwgCiAgY29sID0gY29sX2Z1bkdSMiwKICBjb2x1bW5fdGl0bGVfZ3AgPSBncGFyKGZvbnRzaXplID0gMTYsIGZvbnRmYWNlID0gImJvbGQiKSwKICB3aWR0aCA9IHVuaXQoNTAsICJtbSIpLAogIGNsdXN0ZXJfY29sdW1ucyA9IEZBTFNFLAogIHNob3dfcm93X25hbWVzID0gRkFMU0UpCgojIyMjbWVhbnMKZGF0YUhNbTM8LWJhc2VNZWFuc0htTVsgdG9wREVnZW5lcywgXQojZGF0YUhNbSA8LSBsb2cyKGRhdGFITW0rMSkKZGF0YUhNbTM8LSB0KGFzLm1hdHJpeChkYXRhSE1tMykpCmRhdGFITW0zIDwtIHQoc2NhbGUoZGF0YUhNbTMpKQpkYXRhSE1tMzwtZGF0YUhNbTNbLGMoNSwxLDYsMiw3LDMsOCw0KV0KaG1hcF9oaWVyX2ZhY3RvcnM1IDwtIEhlYXRtYXAoCiAgZGF0YUhNbTMsICBuYW1lID0gIkV4cHJlc3Npb24iLAogIHJvd19sYWJlbHMgPSBwYXN0ZTAocm93bmFtZXMoZGF0YUhNbSksIiAiLCh0ZW1wQVsgdG9wREVnZW5lcywgXSkkR2VuZV9TeW1ib2wpLAogIGNvbHVtbl90aXRsZSA9IHBhc3RlMCgiTWVhbnMiKSwgCiAgY29sID0gY29sX2Z1biwKICBjb2x1bW5fdGl0bGVfZ3AgPSBncGFyKGZvbnRzaXplID0gMTYsIGZvbnRmYWNlID0gImJvbGQiKSwKICB3aWR0aCA9IHVuaXQoMTAwLCAibW0iKSwKICBjbHVzdGVyX2NvbHVtbnMgPSBGQUxTRSwKICBzaG93X3Jvd19uYW1lcyA9IEZBTFNFKQogIApobWFwX2hpZXJfZmFjdG9yczQraG1hcF9oaWVyX2ZhY3RvcnM2K2htYXBfaGllcl9mYWN0b3JzNQoKYGBgCgoKYGBge3IsZmlnLmhlaWdodCA9IDcsZmlnLndpZHRoID0xMiB9CnBhcihtZnJvdz1jKDEsMikpCiMjIyMgU2lsaG91ZXR0ZSBtZXRob2QKZnZpel9uYmNsdXN0KGRhdGFITW0sIGttZWFucywgbWV0aG9kID0gInNpbGhvdWV0dGUiLGsubWF4ID0gMTYpKwogIGxhYnMoc3VidGl0bGUgPSAiU2lsaG91ZXR0ZSBtZXRob2QiKQoKIyMjIyBFbGJvdyBtZXRob2QKZnZpel9uYmNsdXN0KGRhdGFITW0sIGttZWFucywgbWV0aG9kID0gIndzcyIsay5tYXggPSAxNikgKwogIGxhYnMoc3VidGl0bGUgPSAiRWxib3cgbWV0aG9kIikKCmBgYAoKYGBge3J9CiMjIyNnYXAgc3RhdCBzbG93ISEhCiMjIyNzZXQuc2VlZCgxMjMpCiMjIyNmdml6X25iY2x1c3QoZGF0YUhNbSwga21lYW5zLCBuc3RhcnQgPSAyNSwgIG1ldGhvZCA9ICJnYXBfc3RhdCIsIG5ib290ID0gMTAwLGsubWF4ID0gMTYpKwojIyMjICBsYWJzKHN1YnRpdGxlID0gIkdhcCBzdGF0aXN0aWMgbWV0aG9kIikKYGBgCgoKYGBge3IsZmlnLmhlaWdodCA9IDd9CiNrY2x1c3Q0YiA8LSBrbWVhbnMoZGF0YUhNbSwgMykKI3NpbGhvdWV0dGUgcGxvdApkaXN0SzwtZGFpc3koZGF0YUhNbSkKcGxvdChzaWxob3VldHRlKGtjbHVzdDRiJGNsdXN0ZXIsIGRpc3RLKSwgY29sPTE6MywgYm9yZGVyPU5BKQoKYGBgCgojIyMjMy4gSy1tZWFucyBjbHVzdGVyaW5nIG9mIG1lYW5zCgpgYGB7cixmaWcuaGVpZ2h0ID0gMTAsZmlnLndpZHRoID0gMjB9CnNwbGl0IDwtIHBhc3RlMCgiQ2x1c3RlclxuIiwga2NsdXN0NGIkY2x1c3RlcikKI3NwbGl0IDwtIGZhY3RvcihwYXN0ZTAoIkNsdXN0ZXJcbiIsIGtjbHVzdDMkY2x1c3RlciksIGxldmVscz1jKCJDbHVzdGVyXG4zIiwiQ2x1c3RlclxuMSIsIkNsdXN0ZXJcbjQiLCJDbHVzdGVyXG41IiwiQ2x1c3RlclxuMiIsIkNsdXN0ZXJcbjYiKSkKaG1hcF9rIDwtIEhlYXRtYXAoZGF0YUhNbSwgc3BsaXQ9c3BsaXQsIGNsdXN0ZXJfcm93X3NsaWNlcyA9IEZBTFNFLAogICAgICAgICAgICAgICAgICBjbHVzdGVyX2NvbHVtbnMgPSBGQUxTRSwKICAgICAgICAgICAgICAgICAgc2hvd19yb3dfbmFtZXMgPSBGQUxTRSwKICAgICAgICAgICAgICAgICAgbmFtZSA9ICJsb2dmYyIsCiAgICAgICAgICAgICAgICAgIGNvbCA9IGNvbF9mdW5HUiwKICAgICAgICAgICAgICAgICAgd2lkdGggPSB1bml0KDUwLCAibW0iKSwKICAgICAgICAgICAgICAgICAgY29sdW1uX3RpdGxlID0gIm1lYW5zIGxvZ2ZjIiwgCiAgICAgICAgICAgICAgICAgIGNvbHVtbl90aXRsZV9ncCA9IGdwYXIoZm9udHNpemUgPSAxNiwgZm9udGZhY2UgPSAiYm9sZCIpKQoKaG1hcF9rK2htYXBfaGllcl9mYWN0b3JzNitobWFwX2hpZXJfZmFjdG9yczUKYGBgCgoKTWVhbiBwcm9maWxlcyBvZiBjbHVzdGVycwoKYGBge3IsZmlnLmhlaWdodCA9IDgsZmlnLndpZHRoID0gOH0KY2x1c3RlcmNvdW50PC1kYXRhLmZyYW1lKGtjbHVzdDRiJGNsdXN0ZXIpCmNsdXN0ZXJzaXplczwtdGFibGUoY2x1c3RlcmNvdW50JGtjbHVzdDRiLmNsdXN0ZXIpCmNsdXN0ZXJNZWFuczwtZGF0YS5mcmFtZShrY2x1c3Q0YiRjZW50ZXJzKQpjbHVzdGVyTWVhbnMxPC1kYXRhLmZyYW1lKHQoY2x1c3Rlck1lYW5zKSkKY2x1c3Rlck1lYW5zMSA8LSBjYmluZChyb3duYW1lcyhjbHVzdGVyTWVhbnMxKSwgY2x1c3Rlck1lYW5zMSkKb3JkZXJOPC1jKCJWYXIxNF9SQkNfMGgiLCJWYXIxNF9SQkNfMmgiLCJWYXIxNF9SQkNfNmgiLCJWYXIxNF9SQkNfMjBoIikjIyMjIG1hbnVhbAoKcm93bmFtZXMoY2x1c3Rlck1lYW5zMSkgPC0gTlVMTApuYW1lcyhjbHVzdGVyTWVhbnMxKVtuYW1lcyhjbHVzdGVyTWVhbnMxKT09InJvd25hbWVzKGNsdXN0ZXJNZWFuczEpIl0gPC0gIlNhbXBsZSIKIyMjI2NsdXN0ZXJNZWFuczEKCnBYMTwtZ2dwbG90KGRhdGE9Y2x1c3Rlck1lYW5zMSwgYWVzKHg9U2FtcGxlLCB5PVgxLGdyb3VwPTEpKSArCiAgZ2VvbV9saW5lKCkrICBnZW9tX3BvaW50KCkrZ2d0aXRsZShwYXN0ZSgiQ2x1c3RlciBYMSBQcm9maWxlICIsY2x1c3RlcnNpemVzWzFdLCIgZ2VuZXMiKSkrICBzY2FsZV94X2Rpc2NyZXRlKGxpbWl0cz1vcmRlck4pKwogIHRoZW1lKGF4aXMudGl0bGUueCA9IGVsZW1lbnRfYmxhbmsoKSxheGlzLnRpdGxlLnkgPSBlbGVtZW50X2JsYW5rKCkpCnBYMjwtZ2dwbG90KGRhdGE9Y2x1c3Rlck1lYW5zMSwgYWVzKHg9U2FtcGxlLCB5PVgyLGdyb3VwPTEpKSArCiAgZ2VvbV9saW5lKCkrICBnZW9tX3BvaW50KCkrZ2d0aXRsZShwYXN0ZSgiQ2x1c3RlciBYMiBQcm9maWxlICIsY2x1c3RlcnNpemVzWzJdLCIgZ2VuZXMiKSkrICBzY2FsZV94X2Rpc2NyZXRlKGxpbWl0cz1vcmRlck4pKwogIHRoZW1lKGF4aXMudGl0bGUueCA9IGVsZW1lbnRfYmxhbmsoKSxheGlzLnRpdGxlLnkgPSBlbGVtZW50X2JsYW5rKCkpCnBYMzwtZ2dwbG90KGRhdGE9Y2x1c3Rlck1lYW5zMSwgYWVzKHg9U2FtcGxlLCB5PVgzLGdyb3VwPTEpKSArCiAgZ2VvbV9saW5lKCkrICBnZW9tX3BvaW50KCkrZ2d0aXRsZShwYXN0ZSgiQ2x1c3RlciBYMyBQcm9maWxlICIsY2x1c3RlcnNpemVzWzNdLCIgZ2VuZXMiKSkrICBzY2FsZV94X2Rpc2NyZXRlKGxpbWl0cz1vcmRlck4pKwogIHRoZW1lKGF4aXMudGl0bGUueCA9IGVsZW1lbnRfYmxhbmsoKSxheGlzLnRpdGxlLnkgPSBlbGVtZW50X2JsYW5rKCkpCiNwWDQ8LWdncGxvdChkYXRhPWNsdXN0ZXJNZWFuczEsIGFlcyh4PVNhbXBsZSwgeT1YNCxncm91cD0xKSkgKwojICBnZW9tX2xpbmUoKSsgIGdlb21fcG9pbnQoKStnZ3RpdGxlKHBhc3RlKCJDbHVzdGVyIFg0IFByb2ZpbGUgIixjbHVzdGVyc2l6ZXNbNF0sIiBnZW5lcyIpKSsgIHNjYWxlX3hfZGlzY3JldGUobGltaXRzPW9yZGVyTikrCiMgIHRoZW1lKGF4aXMudGl0bGUueCA9IGVsZW1lbnRfYmxhbmsoKSxheGlzLnRpdGxlLnkgPSBlbGVtZW50X2JsYW5rKCkpCiNwWDU8LWdncGxvdChkYXRhPWNsdXN0ZXJNZWFuczEsIGFlcyh4PVNhbXBsZSwgeT1YNSxncm91cD0xKSkgKwojICBnZW9tX2xpbmUoKSsgIGdlb21fcG9pbnQoKStnZ3RpdGxlKHBhc3RlKCJDbHVzdGVyIFg1IFByb2ZpbGUgIixjbHVzdGVyc2l6ZXNbNV0sIiBnZW5lcyIpKSsgIHNjYWxlX3hfZGlzY3JldGUobGltaXRzPW9yZGVyTikrCiMgIHRoZW1lKGF4aXMudGl0bGUueCA9IGVsZW1lbnRfYmxhbmsoKSxheGlzLnRpdGxlLnkgPSBlbGVtZW50X2JsYW5rKCkpCiNwWDY8LWdncGxvdChkYXRhPWNsdXN0ZXJNZWFuczEsIGFlcyh4PVNhbXBsZSwgeT1YNixncm91cD0xKSkgKwojICBnZW9tX2xpbmUoKSsgIGdlb21fcG9pbnQoKStnZ3RpdGxlKHBhc3RlKCJDbHVzdGVyIFg2IFByb2ZpbGUgIixjbHVzdGVyc2l6ZXNbNl0sIiBnZW5lcyIpKSsgIHNjYWxlX3hfZGlzY3JldGUobGltaXRzPW9yZGVyTikrCiMgIHRoZW1lKGF4aXMudGl0bGUueCA9IGVsZW1lbnRfYmxhbmsoKSxheGlzLnRpdGxlLnkgPSBlbGVtZW50X2JsYW5rKCkpCgoKI3Bsb3QKbXVsdGlwbG90KHBYMSwgcFgyLCBwWDMsICBjb2xzPTIpCgpgYGAKCgpgYGB7cn0KdG9wREVnZW5lcyA8LSB3aGljaCh0ZW1wQSRJbmNsdWRlPT0iaW4iKSMjIyNmaW5kIGluZGV4ZXMKdGVtcEFrbTwtdGVtcEFbIHRvcERFZ2VuZXMsIF0KU3ltYm9sc0ttPC1kcGx5cjo6cHVsbCh0ZW1wQWttLCBHZW5lX1N5bWJvbCkKIyMjIyBleHBvcnQgdGhlIGdlbmUgZXhwcmVzc2lvbiBkYXRhIGZvciB0aGUgY2x1c3RlcnMKd3JpdGUudGFibGUoY2x1c3Rlck1lYW5zLHBhc3RlMCgiQ2x1c3Rlck1lYW5zS21fIixncm91cHNOYW1lLCIudHh0IiksICBzZXAgPSAiXHQiKQpDbHVzdGVyZWRHZW5lczwtZGF0YS5mcmFtZShrY2x1c3Q0YiRjbHVzdGVyLFN5bWJvbHNLbSxkYXRhSE1tKQp3cml0ZS50YWJsZShDbHVzdGVyZWRHZW5lcyxwYXN0ZTAoIlNjYWxlZERhdGFJbkNsdXN0ZXJzS21fIixncm91cHNOYW1lLCIudHh0IiksICBzZXAgPSAiXHQiKQojaGVhZChDbHVzdGVyZWRHZW5lcykKYGBgCgoKYGBge3J9CmJvdHRvbURFZ2VuZXM8LXdoaWNoKHRlbXBBJEluY2x1ZGU9PSJvdXQiKSMjIyNmaW5kIGluZGV4ZXMgCmJvdHRvbUc8LXRlbXBBWyBib3R0b21ERWdlbmVzLCBdCmJvdHRvbUc8LWRwbHlyOjpwdWxsKGJvdHRvbUcsIEdlbmVfU3ltYm9sKQp3cml0ZS50YWJsZShib3R0b21HLHBhc3RlMCgiaXBhQm90dG9tS21lYW5zXyIsZ3JvdXBzTmFtZSwiLnR4dCIpLCAgc2VwID0gIlx0IikKICAgICAgICAgICAgICAgICAgICAgICAgIAoKdG9wREVnZW5lcyA8LSB3aGljaCh0ZW1wQSRJbmNsdWRlPT0iaW4iKSMjIyNmaW5kIGluZGV4ZXMgCnRlbXBBa208LXRlbXBBWyB0b3BERWdlbmVzLCBdClN5bWJvbHNLbTwtZHBseXI6OnB1bGwodGVtcEFrbSwgR2VuZV9TeW1ib2wpCgppcGFLbWVhbnM8LUNsdXN0ZXJlZEdlbmVzCiNjb3VudHNUYWJsZSA8LWNvdW50c1RhYmxlWyxjKDE6MTUpXSMjIyNpZiBzYW1wbGVzIG5lZWQgcmVtb3ZpbmcKaXBhS21lYW5zPC1pcGFLbWVhbnNbLGMoMToyKV0KaXBhS21lYW5zJG5hbWUyPC1yb3duYW1lcyhpcGFLbWVhbnMpCiNpcGFLbWVhbnMlPiUgcm93bmFtZXNfdG9fY29sdW1uKHZhciA9ICJyb3duYW1lIikKI2lwYUttZWFucwojcm93aWRfdG9fY29sdW1uKGlwYUttZWFucykKaXBhS21lYW5zID0gbXV0YXRlKGlwYUttZWFucywgeDE9IGlmZWxzZShpcGFLbWVhbnMka2NsdXN0NGIuY2x1c3Rlcj09MSwgIjEiLCAiMCIpKQppcGFLbWVhbnMgPSBtdXRhdGUoaXBhS21lYW5zLCB4Mj0gaWZlbHNlKGlwYUttZWFucyRrY2x1c3Q0Yi5jbHVzdGVyPT0yLCAiMSIsICIwIikpCmlwYUttZWFucyA9IG11dGF0ZShpcGFLbWVhbnMsIHgzPSBpZmVsc2UoaXBhS21lYW5zJGtjbHVzdDRiLmNsdXN0ZXI9PTMsICIxIiwgIjAiKSkKI2lwYUttZWFucyA9IG11dGF0ZShpcGFLbWVhbnMsIHg0PSBpZmVsc2UoaXBhS21lYW5zJGtjbHVzdDRiLmNsdXN0ZXI9PTQsICIxIiwgIjAiKSkKI2lwYUttZWFucyA9IG11dGF0ZShpcGFLbWVhbnMsIHg1PSBpZmVsc2UoaXBhS21lYW5zJGtjbHVzdDRiLmNsdXN0ZXI9PTUsICIxIiwgIjAiKSkKI2lwYUttZWFucyA9IG11dGF0ZShpcGFLbWVhbnMsIHg2PSBpZmVsc2UoaXBhS21lYW5zJGtjbHVzdDRiLmNsdXN0ZXI9PTYsICIxIiwgIjAiKSkKI2lwYUttZWFucwp3cml0ZS50YWJsZShpcGFLbWVhbnMscGFzdGUwKCJpcGFLbWVhbnNfIixncm91cHNOYW1lLCIudHh0IiksICBzZXAgPSAiXHQiKQojaGVhZChpcGFLbWVhbnMpCgpgYGAKCgpgYGB7cn0KQ2x1c3RlcmVkR2VuZXMyPC1DbHVzdGVyZWRHZW5lc1tjKDEpXQojQ2x1c3RlcmVkR2VuZXMyCmxpc3RBbGw8LWxpc3QoKQpmb3IoaSBpbiAxOjMpIHsKICBjbHVzdGVyTmFtZTwtcGFzdGUwKCJ4IixpKQogICNjbHVzdGVyTmFtZTwtcm93Lm5hbWVzKHN1YnNldChDbHVzdGVyZWRHZW5lcyxDbHVzdGVyZWRHZW5lcz09aSkpCiAgY2x1c3Rlck5hbWU8LShzdWJzZXQoQ2x1c3RlcmVkR2VuZXMkU3ltYm9sc0ttLENsdXN0ZXJlZEdlbmVzPT1pKSkKICBsaXN0QWxsW1tpXV08LWNsdXN0ZXJOYW1lCn0KI25lZWQgdG8gbmFtZSB0aGUgdmVjdG9ycyBpbiB0aGUgbGlzdCwgZXhhbXBsZSBoZXJlIGlzIGZvciA4IGNsdXN0ZXJzCm5hbWVzKGxpc3RBbGwpPC1jKCJYMSIsICJYMiIsICJYMyIpCgojaWYgeW91IHdhbnQgdG8gcmVhcnJhbmdlIHRoZSBvcmRlcgojbGlzdEFsbDwtbGlzdEFsbFtjKCJ4MyIsICJ4NyIsICJ4OCIsICJ4MiIsICJ4NiIsICJ4NSIsICJ4NCIsICJ4MSIpXQoKbGFwcGx5KGxpc3RBbGwsIGhlYWQpCgpgYGAKCiMjIyM0LiBBbm5vdGF0aW9uIG9mIEstbWVhbnMgY2x1c3RlcnMKLSBDQyBjZWxsdWxhciBjb21wYXJ0bWVudAotIEJQIGJpb2xvZ2ljYWwgcHJvY2VzcwotIE1GIG1vbGVjdWxhciBmdW5jdGlvbgoKVGhlIHNpbXBsaWZ5IGZ1bmN0aW9uIGhhcyBiZWVuIHVzZWQgdG8gY3V0IGRvd24gb24gR08gcmVkdW5kYW5jeQoKYGBge3J9CiNzdHIoQWxsR2VuZU5hbWVzKQpgYGAKCgpgYGB7cixmaWcuaGVpZ2h0ID0gOCxmaWcud2lkdGggPSAxMn0KIyMjI0NDCmNnb0NDIDwtIGNvbXBhcmVDbHVzdGVyKGdlbmVDbHVzdGVyID0gbGlzdEFsbCwgCiAgICAgICAgICAgICAgICAgICAgICB1bml2ZXJzZSA9IEFsbEdlbmVOYW1lcywKICAgICAgICAgICAgICAgICAgICAgIGZ1biA9ICJlbnJpY2hHTyIsCiAgICAgICAgICAgICAgICAgICAgICBPcmdEYj1vcmcuSHMuZWcuZGIsIAogICAgICAgICAgICAgICAgICAgICAgIyMjI09yZ0RiPW9yZy5NbS5lZy5kYiwKICAgICAgICAgICAgICAgICAgICAgIGtleVR5cGU9IlNZTUJPTCIsCiAgICAgICAgICAgICAgICAgICAgICBvbnQgPSAiQ0MiLCAKICAgICAgICAgICAgICAgICAgICAgIHB2YWx1ZUN1dG9mZj0wLjA1LAogICAgICAgICAgICAgICAgICAgICAgcXZhbHVlQ3V0b2ZmID0gMC4xMCkKY2dvQ0MyIDwtIHNpbXBsaWZ5KGNnb0NDLCBjdXRvZmY9MC43LCBieT0icC5hZGp1c3QiLCBzZWxlY3RfZnVuPW1pbikKIyMjI3dyaXRlIGFzIHNwcmVhZHNoZWV0CndyaXRlLmNzdihhcy5kYXRhLmZyYW1lKGNnb0NDMikscGFzdGUwKCJHT19DQ18iLGdyb3Vwc05hbWUsIi5jc3YiKSkKCmRvdHBsb3QoY2dvQ0MyLHNob3dDYXRlZ29yeSA9IDMwLAogICAgICAgIHRpdGxlID0gcGFzdGUwKCJHTyBDZWxsdWxhciBDb21wYXJ0bWVudCAiLGdyb3Vwc05hbWUpKSsKICB0aGVtZShheGlzLnRleHQueCA9IGVsZW1lbnRfdGV4dChhbmdsZSA9IDkwLCB2anVzdCA9IDAuNSwgaGp1c3Q9MSkpCgpgYGAKUGxvdHMgYW5kIEdPIGRhdGEgd2VyZSB3cml0dGVuIHRvIGZpbGVzCmBgYHtyfQpwbmcocGFzdGUwKCJHT19DQ18iLGdyb3Vwc05hbWUsIi5wbmciKSwgd2lkdGggPSAxMjI0LCBoZWlnaHQgPSA4MjQpCmRvdHBsb3QoY2dvQ0MyLHNob3dDYXRlZ29yeSA9IDMwLAogICAgICAgIHRpdGxlID0gcGFzdGUwKCJHTyBDZWxsdWxhciBDb21wYXJ0bWVudCAiLGdyb3Vwc05hbWUpKSsKICB0aGVtZShheGlzLnRleHQueCA9IGVsZW1lbnRfdGV4dChhbmdsZSA9IDkwLCB2anVzdCA9IDAuNSwgaGp1c3Q9MSkpCmRldi5vZmYoKQpgYGAKCkdPIEJQCmBgYHtyLGZpZy5oZWlnaHQgPSAxMixmaWcud2lkdGggPSAxMn0KIyMjI0NDCmNnb0JQIDwtIGNvbXBhcmVDbHVzdGVyKGdlbmVDbHVzdGVyID0gbGlzdEFsbCwgCiAgICAgICAgICAgICAgICAgICAgICB1bml2ZXJzZSA9IEFsbEdlbmVOYW1lcywKICAgICAgICAgICAgICAgICAgICAgIGZ1biA9ICJlbnJpY2hHTyIsCiAgICAgICAgICAgICAgICAgICAgICBPcmdEYj1vcmcuSHMuZWcuZGIsCiAgICAgICAgICAgICAgICAgICAgICBrZXlUeXBlPSJTWU1CT0wiLAogICAgICAgICAgICAgICAgICAgICAgb250ID0gIkJQIiwgCiAgICAgICAgICAgICAgICAgICAgICBwdmFsdWVDdXRvZmY9MC4wNSwKICAgICAgICAgICAgICAgICAgICAgIHF2YWx1ZUN1dG9mZiA9IDAuMTApCmNnb0JQMiA8LSBzaW1wbGlmeShjZ29CUCwgY3V0b2ZmPTAuNywgYnk9InAuYWRqdXN0Iiwgc2VsZWN0X2Z1bj1taW4pCiMjIyN3cml0ZSBhcyBzcHJlYWRzaGVldAp3cml0ZS5jc3YoYXMuZGF0YS5mcmFtZShjZ29CUDIpLHBhc3RlMCgiR09fQlBfIixncm91cHNOYW1lLCIuY3N2IikpCgpkb3RwbG90KGNnb0JQMixzaG93Q2F0ZWdvcnkgPSAzMCwKICAgICAgICB0aXRsZSA9IHBhc3RlMCgiR08gQmlvbG9naWNhbCBQcm9jZXNzICIsZ3JvdXBzTmFtZSkpKwogIHRoZW1lKGF4aXMudGV4dC54ID0gZWxlbWVudF90ZXh0KGFuZ2xlID0gOTAsIHZqdXN0ID0gMC41LCBoanVzdD0xKSkKCmBgYAoKYGBge3J9CnBuZyhwYXN0ZTAoIkdPX0JQXyIsZ3JvdXBzTmFtZSwiLnBuZyIpLCB3aWR0aCA9IDEwMjQsIGhlaWdodCA9IDEyMjQpCmRvdHBsb3QoY2dvQlAyLHNob3dDYXRlZ29yeSA9IDMwLAogICAgICAgIHRpdGxlID0gcGFzdGUwKCJHTyBCaW9sb2dpY2FsIFByb2Nlc3MgIixncm91cHNOYW1lKSkrCiAgdGhlbWUoYXhpcy50ZXh0LnggPSBlbGVtZW50X3RleHQoYW5nbGUgPSA5MCwgdmp1c3QgPSAwLjUsIGhqdXN0PTEpKQpkZXYub2ZmKCkKYGBgCgoKR08gTUYKYGBge3IsZmlnLmhlaWdodCA9IDEwLGZpZy53aWR0aCA9IDEyfQojIyMjTUYKY2dvTUYgPC0gY29tcGFyZUNsdXN0ZXIoZ2VuZUNsdXN0ZXIgPSBsaXN0QWxsLCAKICAgICAgICAgICAgICAgICAgICAgIHVuaXZlcnNlID0gQWxsR2VuZU5hbWVzLAogICAgICAgICAgICAgICAgICAgICAgZnVuID0gImVucmljaEdPIiwKICAgICAgICAgICAgICAgICAgICAgIE9yZ0RiPW9yZy5Icy5lZy5kYiwgCiAgICAgICAgICAgICAgICAgICAgICBrZXlUeXBlPSJTWU1CT0wiLAogICAgICAgICAgICAgICAgICAgICAgb250ID0gIk1GIiwgCiAgICAgICAgICAgICAgICAgICAgICBwdmFsdWVDdXRvZmY9MC4wNSwKICAgICAgICAgICAgICAgICAgICAgIHF2YWx1ZUN1dG9mZiA9IDAuMTApCmNnb01GMiA8LSBzaW1wbGlmeShjZ29NRiwgY3V0b2ZmPTAuNywgYnk9InAuYWRqdXN0Iiwgc2VsZWN0X2Z1bj1taW4pCiMjIyN3cml0ZSBhcyBzcHJlYWRzaGVldAp3cml0ZS5jc3YoYXMuZGF0YS5mcmFtZShjZ29NRjIpLHBhc3RlMCgiR09fTUZfIixncm91cHNOYW1lLCIuY3N2IikpCgpkb3RwbG90KGNnb01GMixzaG93Q2F0ZWdvcnkgPSAzMCwKICAgICAgICB0aXRsZSA9IHBhc3RlMCgiR08gTW9sZWN1bGFyIEZ1bmN0aW9uICAiLGdyb3Vwc05hbWUpKSsKICB0aGVtZShheGlzLnRleHQueCA9IGVsZW1lbnRfdGV4dChhbmdsZSA9IDkwLCB2anVzdCA9IDAuNSwgaGp1c3Q9MSkpCgpgYGAKCgoKYGBge3J9CnBuZyhwYXN0ZTAoIkdPX01GXyIsZ3JvdXBzTmFtZSwiLnBuZyIpLCB3aWR0aCA9IDE0MjQsIGhlaWdodCA9IDgyNCkKZG90cGxvdChjZ29NRjIsc2hvd0NhdGVnb3J5ID0gMzAsCiAgICAgICAgdGl0bGUgPSBwYXN0ZTAoIkdPIE1vbGVjdWxhciBGdW5jdGlvbiAgIixncm91cHNOYW1lKSkrCiAgdGhlbWUoYXhpcy50ZXh0LnggPSBlbGVtZW50X3RleHQoYW5nbGUgPSA5MCwgdmp1c3QgPSAwLjUsIGhqdXN0PTEpKQpkZXYub2ZmKCkKYGBgCgoKIyMjIFI1IFZBUjE0IHZzIFJCQyBUTkYgay1tZWFucyBxMC4wNQoKIyMjIzEuIEdlbmVsaXN0IFNlbGVjdGlvbgoKYGBge3J9Cmdyb3Vwc05hbWU8LSJSNV9WQVIxNF9SQkNfVE5GX2ttZWFuc19xMC4wNSIKYGBgCgoKYGBge3J9CmNvdW50c1RhYmxlPC1yZWFkLmRlbGltKCJSTkFzZXEyMDE5SnVseV81LnR4dCIsIGhlYWRlciA9IFRSVUUsIHNlcCA9ICJcdCIsY2hlY2submFtZXM9RkFMU0Uscm93Lm5hbWVzPTEpCmhlYWQoY291bnRzVGFibGUpCmBgYAoKYGBge3J9CkFsbEdlbmVOYW1lczwtY291bnRzVGFibGUkR2VuZV9TeW1ib2wKI2hlYWQoQWxsR2VuZU5hbWVzKQpgYGAKCmBgYHtyfQp0ZW1wQTwtY291bnRzVGFibGUKYGBgCgoKYGBge3J9Cgp0b3BERWdlbmVzIDwtIHdoaWNoKHRlbXBBJHBhZGpfUjVfVE5GX3ZhcjE0X3ZzX1JCQ18waDwwLjA1JiFpcy5uYSh0ZW1wQSRwYWRqX1I1X1RORl92YXIxNF92c19SQkNfMGgpKSMjIyNmaW5kIGluZGV4ZXMgCmxpc3RBPC10ZW1wQVsgdG9wREVnZW5lcywgXSRHZW5lX1N5bWJvbAp0b3BERWdlbmVzIDwtIHdoaWNoKHRlbXBBJHBhZGpfUjVfVE5GX3ZhcjE0X3ZzX1JCQ18yaDwwLjA1JiFpcy5uYSh0ZW1wQSRwYWRqX1I1X1RORl92YXIxNF92c19SQkNfMmgpKSMjIyNmaW5kIGluZGV4ZXMgCmxpc3RCPC10ZW1wQVsgdG9wREVnZW5lcywgXSRHZW5lX1N5bWJvbAp0b3BERWdlbmVzIDwtIHdoaWNoKHRlbXBBJHBhZGpfUjVfVE5GX3ZhcjE0X3ZzX1JCQ182aDwwLjA1JiFpcy5uYSh0ZW1wQSRwYWRqX1I1X1RORl92YXIxNF92c19SQkNfNmgpKSMjIyNmaW5kIGluZGV4ZXMgCmxpc3RDPC10ZW1wQVsgdG9wREVnZW5lcywgXSRHZW5lX1N5bWJvbAp0b3BERWdlbmVzIDwtIHdoaWNoKHRlbXBBJHBhZGpfUjVfVE5GX3ZhcjE0X3ZzX1JCQ18yMGg8MC4wNSYhaXMubmEodGVtcEEkcGFkal9SNV9UTkZfdmFyMTRfdnNfUkJDXzIwaCkpIyMjI2ZpbmQgaW5kZXhlcyAKbGlzdEQ8LXRlbXBBWyB0b3BERWdlbmVzLCBdJEdlbmVfU3ltYm9sCgoKCnZlbm5xPC12ZW5uLmRpYWdyYW0oeCA9IGxpc3QobGlzdEEsbGlzdEIsbGlzdEMsbGlzdEQpICwKICAgICAgICAgICAgY2F0ZWdvcnkubmFtZXMgPSBjKCJWYXIxNG5ORl8waCIsIlZhcjE0VE5GXzJoIiwiVmFyMTRUTkZfNmgiLCJWYXIxNFRORl8yMGgiKSwKICAgICAgICAgICAgbWFpbj0icGFkajwwLjA1IiwKICAgICAgICAgICAgZmlsZW5hbWUgPSBOVUxMLCAgc2NhbGVkID0gRkFMU0UsIGZpbGwgPSBjb2xvcnNWNCwgY2F0LmNvbCA9IGNvbG9yc1Y0LCBjYXQuY2V4ID0gMSwgY2F0LmRpc3Q9MC4zLCAgbWFyZ2luID0gMC4zKQoKdG9wREVnZW5lcyA8LSB3aGljaCh0ZW1wQSRwdmFsdWVfUjVfVE5GX3ZhcjE0X3ZzX1JCQ18waDwwLjA1JmFicyh0ZW1wQSRsb2cyRm9sZENoYW5nZV9SNV9UTkZfdmFyMTRfdnNfUkJDXzBoKT4xJiFpcy5uYSh0ZW1wQSRwdmFsdWVfUjVfVE5GX3ZhcjE0X3ZzX1JCQ18waCkpIyMjI2ZpbmQgaW5kZXhlcyAKbGlzdEE8LXRlbXBBWyB0b3BERWdlbmVzLCBdJEdlbmVfU3ltYm9sCnRvcERFZ2VuZXMgPC0gd2hpY2godGVtcEEkcHZhbHVlX1I1X1RORl92YXIxNF92c19SQkNfMmg8MC4wNSZhYnModGVtcEEkbG9nMkZvbGRDaGFuZ2VfUjVfVE5GX3ZhcjE0X3ZzX1JCQ18yaCk+MSYhaXMubmEodGVtcEEkcHZhbHVlX1I1X1RORl92YXIxNF92c19SQkNfMmgpKSMjIyNmaW5kIGluZGV4ZXMgCmxpc3RCPC10ZW1wQVsgdG9wREVnZW5lcywgXSRHZW5lX1N5bWJvbAp0b3BERWdlbmVzIDwtIHdoaWNoKHRlbXBBJHB2YWx1ZV9SNV9UTkZfdmFyMTRfdnNfUkJDXzZoPDAuMDUmYWJzKHRlbXBBJGxvZzJGb2xkQ2hhbmdlX1I1X1RORl92YXIxNF92c19SQkNfNmgpPjEmIWlzLm5hKHRlbXBBJHB2YWx1ZV9SNV9UTkZfdmFyMTRfdnNfUkJDXzZoKSkjIyMjZmluZCBpbmRleGVzIApsaXN0QzwtdGVtcEFbIHRvcERFZ2VuZXMsIF0kR2VuZV9TeW1ib2wKdG9wREVnZW5lcyA8LSB3aGljaCh0ZW1wQSRwdmFsdWVfUjVfVE5GX3ZhcjE0X3ZzX1JCQ18yMGg8MC4wNSZhYnModGVtcEEkbG9nMkZvbGRDaGFuZ2VfUjVfVE5GX3ZhcjE0X3ZzX1JCQ18yMGgpPjEmIWlzLm5hKHRlbXBBJHB2YWx1ZV9SNV9UTkZfdmFyMTRfdnNfUkJDXzIwaCkpIyMjI2ZpbmQgaW5kZXhlcyAKbGlzdEQ8LXRlbXBBWyB0b3BERWdlbmVzLCBdJEdlbmVfU3ltYm9sCgp2ZW5ucDwtdmVubi5kaWFncmFtKHggPSBsaXN0KGxpc3RBLGxpc3RCLGxpc3RDLGxpc3REKSAsCiAgICAgICAgICAgIGNhdGVnb3J5Lm5hbWVzID0gYygiVmFyMTRUTkZfMGgiLCJWYXIxNFRORl8yaCIsIlZhcjE0VE5GXzZoIiwiVmFyMTRUTkZfMjBoIiksCiAgICAgICAgICAgIG1haW49InB2YWx1ZTwwLjA1JmZvbGQgY2hhbmdlPjIiLAogICAgICAgICAgICBmaWxlbmFtZSA9IE5VTEwsICBzY2FsZWQgPSBGQUxTRSwgZmlsbCA9IGNvbG9yc1Y0LCBjYXQuY29sID0gY29sb3JzVjQsIGNhdC5jZXggPSAxLCBjYXQuZGlzdD0wLjMsICBtYXJnaW4gPSAwLjMpCgp0b3BERWdlbmVzIDwtIHdoaWNoKHRlbXBBJHBhZGpfUjVfVE5GX3ZhcjE0X3ZzX1JCQ18waDwwLjEmYWJzKHRlbXBBJGxvZzJGb2xkQ2hhbmdlX1I1X1RORl92YXIxNF92c19SQkNfMGgpPjEmIWlzLm5hKHRlbXBBJHBhZGpfUjVfVE5GX3ZhcjE0X3ZzX1JCQ18waCkpIyMjI2ZpbmQgaW5kZXhlcyAKbGlzdEE8LXRlbXBBWyB0b3BERWdlbmVzLCBdJEdlbmVfU3ltYm9sCnRvcERFZ2VuZXMgPC0gd2hpY2godGVtcEEkcGFkal9SNV9UTkZfdmFyMTRfdnNfUkJDXzJoPDAuMSZhYnModGVtcEEkbG9nMkZvbGRDaGFuZ2VfUjVfVE5GX3ZhcjE0X3ZzX1JCQ18yaCk+MSYhaXMubmEodGVtcEEkcGFkal9SNV9UTkZfdmFyMTRfdnNfUkJDXzJoKSkjIyMjZmluZCBpbmRleGVzIApsaXN0QjwtdGVtcEFbIHRvcERFZ2VuZXMsIF0kR2VuZV9TeW1ib2wKdG9wREVnZW5lcyA8LSB3aGljaCh0ZW1wQSRwYWRqX1I1X1RORl92YXIxNF92c19SQkNfNmg8MC4xJmFicyh0ZW1wQSRsb2cyRm9sZENoYW5nZV9SNV9UTkZfdmFyMTRfdnNfUkJDXzZoKT4xJiFpcy5uYSh0ZW1wQSRwYWRqX1I1X1RORl92YXIxNF92c19SQkNfNmgpKSMjIyNmaW5kIGluZGV4ZXMgCmxpc3RDPC10ZW1wQVsgdG9wREVnZW5lcywgXSRHZW5lX1N5bWJvbAp0b3BERWdlbmVzIDwtIHdoaWNoKHRlbXBBJHBhZGpfUjVfVE5GX3ZhcjE0X3ZzX1JCQ18yMGg8MC4xJmFicyh0ZW1wQSRsb2cyRm9sZENoYW5nZV9SNV9UTkZfdmFyMTRfdnNfUkJDXzIwaCk+MSYhaXMubmEodGVtcEEkcGFkal9SNV9UTkZfdmFyMTRfdnNfUkJDXzIwaCkpIyMjI2ZpbmQgaW5kZXhlcyAKbGlzdEQ8LXRlbXBBWyB0b3BERWdlbmVzLCBdJEdlbmVfU3ltYm9sCgp2ZW5ucTI8LXZlbm4uZGlhZ3JhbSh4ID0gbGlzdChsaXN0QSxsaXN0QixsaXN0QyxsaXN0RCkgLAogICAgICAgICAgICBjYXRlZ29yeS5uYW1lcyA9IGMoIlZhcjE0VE5GXzBoIiwiVmFyMTRUTkZfMmgiLCJWYXIxNFRORl82aCIsIlZhcjE0VE5GXzIwaCIpLAogICAgICAgICAgICBtYWluPSJwYWRqMC4xJmZvbGQgY2hhbmdlPjIiLAogICAgICAgICAgICBmaWxlbmFtZSA9IE5VTEwsICBzY2FsZWQgPSBGQUxTRSwgZmlsbCA9IGNvbG9yc1Y0LCBjYXQuY29sID0gY29sb3JzVjQsIGNhdC5jZXggPSAxLCBjYXQuZGlzdD0wLjMsICBtYXJnaW4gPSAwLjMpCgpgYGAKCmBgYHtyfQp0b3BERWdlbmVzIDwtIHdoaWNoKCh0ZW1wQSRwYWRqX1I1X1RORl92YXIxNF92c19SQkNfMGg8MC4wNSYhaXMubmEodGVtcEEkcGFkal9SNV9UTkZfdmFyMTRfdnNfUkJDXzBoKSl8IAoodGVtcEEkcGFkal9SNV9UTkZfdmFyMTRfdnNfUkJDXzJoPDAuMDUmIWlzLm5hKHRlbXBBJHBhZGpfUjVfVE5GX3ZhcjE0X3ZzX1JCQ18yaCkpfAoodGVtcEEkcGFkal9SNV9UTkZfdmFyMTRfdnNfUkJDXzZoPDAuMDUmIWlzLm5hKHRlbXBBJHBhZGpfUjVfVE5GX3ZhcjE0X3ZzX1JCQ182aCkpfCAKKHRlbXBBJHBhZGpfUjVfVE5GX3ZhcjE0X3ZzX1JCQ18yMGg8MC4wNSYhaXMubmEodGVtcEEkcGFkal9SNV9UTkZfdmFyMTRfdnNfUkJDXzIwaCkpIAopCmxpc3RBPC10ZW1wQVsgdG9wREVnZW5lcywgXSRHZW5lX1N5bWJvbAoKdG9wREVnZW5lcyA8LSB3aGljaCgodGVtcEEkcHZhbHVlX1I1X1RORl92YXIxNF92c19SQkNfMGg8MC4wNSZhYnModGVtcEEkbG9nMkZvbGRDaGFuZ2VfUjVfVE5GX3ZhcjE0X3ZzX1JCQ18waCk+MSYhaXMubmEodGVtcEEkcHZhbHVlX1I1X1RORl92YXIxNF92c19SQkNfMGgpKXwgCih0ZW1wQSRwdmFsdWVfUjVfVE5GX3ZhcjE0X3ZzX1JCQ18yaDwwLjA1JmFicyh0ZW1wQSRsb2cyRm9sZENoYW5nZV9SNV9UTkZfdmFyMTRfdnNfUkJDXzJoKT4xJiFpcy5uYSh0ZW1wQSRwdmFsdWVfUjVfVE5GX3ZhcjE0X3ZzX1JCQ18yaCkpfCAKKHRlbXBBJHB2YWx1ZV9SNV9UTkZfdmFyMTRfdnNfUkJDXzZoPDAuMDUmYWJzKHRlbXBBJGxvZzJGb2xkQ2hhbmdlX1I1X1RORl92YXIxNF92c19SQkNfNmgpPjEmIWlzLm5hKHRlbXBBJHB2YWx1ZV9SNV9UTkZfdmFyMTRfdnNfUkJDXzZoKSl8IAoodGVtcEEkcHZhbHVlX1I1X1RORl92YXIxNF92c19SQkNfMjBoPDAuMDUmYWJzKHRlbXBBJGxvZzJGb2xkQ2hhbmdlX1I1X1RORl92YXIxNF92c19SQkNfMjBoKT4xJiFpcy5uYSh0ZW1wQSRwdmFsdWVfUjVfVE5GX3ZhcjE0X3ZzX1JCQ18yMGgpKQogKSMjIyNmaW5kIGluZGV4ZXMgCmxpc3RDPC10ZW1wQVsgdG9wREVnZW5lcywgXSRHZW5lX1N5bWJvbAp0b3BERWdlbmVzIDwtIHdoaWNoKCh0ZW1wQSRwYWRqX1I1X1RORl92YXIxNF92c19SQkNfMGg8MC4xJmFicyh0ZW1wQSRsb2cyRm9sZENoYW5nZV9SNV9UTkZfdmFyMTRfdnNfUkJDXzBoKT4xJiFpcy5uYSh0ZW1wQSRwYWRqX1I1X1RORl92YXIxNF92c19SQkNfMGgpKXwgCih0ZW1wQSRwYWRqX1I1X1RORl92YXIxNF92c19SQkNfMmg8MC4xJmFicyh0ZW1wQSRsb2cyRm9sZENoYW5nZV9SNV9UTkZfdmFyMTRfdnNfUkJDXzJoKT4xJiFpcy5uYSh0ZW1wQSRwYWRqX1I1X1RORl92YXIxNF92c19SQkNfMmgpKXwgCih0ZW1wQSRwYWRqX1I1X1RORl92YXIxNF92c19SQkNfNmg8MC4xJmFicyh0ZW1wQSRsb2cyRm9sZENoYW5nZV9SNV9UTkZfdmFyMTRfdnNfUkJDXzZoKT4xJiFpcy5uYSh0ZW1wQSRwYWRqX1I1X1RORl92YXIxNF92c19SQkNfNmgpKXwgCih0ZW1wQSRwYWRqX1I1X1RORl92YXIxNF92c19SQkNfMjBoPDAuMSZhYnModGVtcEEkbG9nMkZvbGRDaGFuZ2VfUjVfVE5GX3ZhcjE0X3ZzX1JCQ18yMGgpPjEmIWlzLm5hKHRlbXBBJHBhZGpfUjVfVE5GX3ZhcjE0X3ZzX1JCQ18yMGgpKQogKSMjIyNmaW5kIGluZGV4ZXMgCmxpc3RCPC10ZW1wQVsgdG9wREVnZW5lcywgXSRHZW5lX1N5bWJvbAoKdmVubnBxPC12ZW5uLmRpYWdyYW0oeCA9IGxpc3QobGlzdEEsbGlzdEIsbGlzdEMpICwKICAgICAgICAgICAgY2F0ZWdvcnkubmFtZXMgPSBjKCJwYWRqPDAuMDUiLCJwYWRqPDAuMSZmYz4yIiwicDwwLjA1JmZjPjIiKSwKICAgICAgICAgICAgbWFpbj0icGFkaiBjb21wYXJlZCB0byBwdmFsdWUiLAogICAgICAgICAgICBmaWxlbmFtZSA9IE5VTEwsICBzY2FsZWQgPSBGQUxTRSwgZmlsbCA9IGNvbG9yc1YzLCBjYXQuY29sID0gY29sb3JzVjMsIGNhdC5jZXggPSAxLCBjYXQuZGlzdD0wLjEsICBtYXJnaW4gPSAwLjE1KQpgYGAKCmBgYHtyLGZpZy5oZWlnaHQgPSAxMCxmaWcud2lkdGggPSAyMH0KZ3JpZC5hcnJhbmdlKGdUcmVlKGNoaWxkcmVuPXZlbm5xKSwgZ1RyZWUoY2hpbGRyZW49dmVubnBxKSwgbmNvbD0yLHRvcD0iUjIgVmFyMTQgVE5GIikKYGBgCgpgYGB7cn0KI3RlbXBBPC1yZXNBbGxbLWMoMTA6MzApIF0KdGVtcEE8LWNvdW50c1RhYmxlCiNyb3duYW1lcyh0ZW1wQSkKcm93bmFtZXModGVtcEEpIDwtIE5VTEwKdGVtcEEgPSBtdXRhdGUodGVtcEEsIEluY2x1ZGU9CiAgICAgICAgICAgICAgICAgICBpZmVsc2UodGVtcEEkcGFkal9SNV9UTkZfdmFyMTRfdnNfUkJDXzBoPDAuMDUmIWlzLm5hKHRlbXBBJHBhZGpfUjVfVE5GX3ZhcjE0X3ZzX1JCQ18waCksICJpbiIsCiAgICAgICAgICAgICAgICAgICAgICAgICAgaWZlbHNlKHRlbXBBJHBhZGpfUjVfVE5GX3ZhcjE0X3ZzX1JCQ18yaDwwLjA1JiFpcy5uYSh0ZW1wQSRwYWRqX1I1X1RORl92YXIxNF92c19SQkNfMmgpLCAiaW4iLAogICAgICAgICAgICAgICAgICAgICAgICAgICAgICAgICBpZmVsc2UodGVtcEEkcGFkal9SNV9UTkZfdmFyMTRfdnNfUkJDXzZoPDAuMDUmIWlzLm5hKHRlbXBBJHBhZGpfUjVfVE5GX3ZhcjE0X3ZzX1JCQ182aCksICJpbiIsCiAgICAgICAgICAgICAgICAgICAgICAgICAgICAgICAgICAgICAgICBpZmVsc2UodGVtcEEkcGFkal9SNV9UTkZfdmFyMTRfdnNfUkJDXzIwaDwwLjA1JiFpcy5uYSh0ZW1wQSRwYWRqX1I1X1RORl92YXIxNF92c19SQkNfMjBoKSwgImluIiwKICAgICAgICAgICAgICAgICAgICAgICAgICAgICAgICAgICAgICAgICAgICAgICAgICJvdXQiKSkpKSkKCgojdGVtcEEKIyMjI2xpYnJhcnkoZHBseXIpCnRlbXBBICU+JQogICAgIGdyb3VwX2J5KEluY2x1ZGUpICU+JSAKICAgICB0YWxseSgpCmBgYAoKYGBge3J9CnRvcERFZ2VuZXMgPC0gd2hpY2godGVtcEEkSW5jbHVkZT09ImluIikjIyMjZmluZCBpbmRleGVzIApgYGAKCmBgYHtyfQpoZWFkKGNvdW50c1RhYmxlKQpgYGAKCiMjIyMgTkIgUGxlYXNlIGNoZWNrIGNvbHVtbnMgdXNlZCBhbmQgcmVuYW1lZCBmb3IgcGxvdHMKYGBge3J9CiNiYXNlTWVhbnNIbSA8LWNvdW50c1RhYmxlWyxjKDYwOjYzKV0KYmFzZU1lYW5zSG0gPC1jb3VudHNUYWJsZVssYygxMTA6MTEzLDEyOToxMzIpXQpoZWFkKGJhc2VNZWFuc0htKQpiYXNlTWVhbnNIbTIgPC0gbG9nMihiYXNlTWVhbnNIbSsxKQpiYXNlTWVhbnNIbTIkVmFyMTRfUkJDXzBoPC1iYXNlTWVhbnNIbTIkVmFyMTRUTkZfMGhfbWVhbi1iYXNlTWVhbnNIbTIkUkJDX1RORl8waF9tZWFuCmJhc2VNZWFuc0htMiRWYXIxNF9SQkNfMmg8LWJhc2VNZWFuc0htMiRWYXIxNFRORl8yaF9tZWFuLWJhc2VNZWFuc0htMiRSQkNfVE5GXzJoX21lYW4KYmFzZU1lYW5zSG0yJFZhcjE0X1JCQ182aDwtYmFzZU1lYW5zSG0yJFZhcjE0VE5GXzZoX21lYW4tYmFzZU1lYW5zSG0yJFJCQ19UTkZfNmhfbWVhbgpiYXNlTWVhbnNIbTIkVmFyMTRfUkJDXzIwaDwtYmFzZU1lYW5zSG0yJFZhcjE0VE5GXzIwaF9tZWFuLWJhc2VNZWFuc0htMiRSQkNfVE5GXzIwaF9tZWFuCmJhc2VNZWFuc0htIDwtYmFzZU1lYW5zSG0yWyxjKDk6MTIpXQpoZWFkKGJhc2VNZWFuc0htKQpiYXNlTWVhbnNIbU0gPC1iYXNlTWVhbnNIbTJbLGMoMTo4KV0KaGVhZChiYXNlTWVhbnNIbU0pCmBgYAoKCmBgYHtyfQp0b3BERWdlbmVzIDwtIHdoaWNoKHRlbXBBJEluY2x1ZGU9PSJpbiIpIyMjI2ZpbmQgaW5kZXhlcyAKYGBgCgoKIyMjIzIuIEhpZXJhY2hpY2FsIGNsdXN0ZXJpbmcgb2YgbWVhbnMgKGluZGl2aWR1YWwgc2FtcGxlcyBhZGRlZCBmb3IgaW5zcGVjdGlvbikKYGBge3IsZmlnLmhlaWdodCA9IDEyLGZpZy53aWR0aCA9IDIwfQojIyMjbWVhbiBsb2dmYwpkYXRhSE1tPC1iYXNlTWVhbnNIbVsgdG9wREVnZW5lcywgXQojZGF0YUhNbSA8LSBsb2cyKGRhdGFITW0rMSkKZGF0YUhNbTwtIHQoYXMubWF0cml4KGRhdGFITW0pKQpkYXRhSE1tIDwtIHQoc2NhbGUoZGF0YUhNbSkpCgpobWFwX2hpZXJfZmFjdG9yczQgPC0gSGVhdG1hcCgKICBkYXRhSE1tLCAgbmFtZSA9ICJsb2dmYyIsCiAgcm93X2xhYmVscyA9IHBhc3RlMChyb3duYW1lcyhkYXRhSE1tKSwiICIsKHRlbXBBWyB0b3BERWdlbmVzLCBdKSRHZW5lX1N5bWJvbCksCiAgY29sdW1uX3RpdGxlID0gcGFzdGUwKCJNZWFucyBsb2dmYyIpLCAKICBjb2wgPSBjb2xfZnVuR1IsCiAgY29sdW1uX3RpdGxlX2dwID0gZ3Bhcihmb250c2l6ZSA9IDE2LCBmb250ZmFjZSA9ICJib2xkIiksCiAgd2lkdGggPSB1bml0KDUwLCAibW0iKSwKICBjbHVzdGVyX2NvbHVtbnMgPSBGQUxTRSwKICBzaG93X3Jvd19uYW1lcyA9IEZBTFNFKQoKZGF0YUhNbVBsb3Q8LWFzLmRhdGEuZnJhbWUoZGF0YUhNbSkKZGF0YUhNbVBsb3QkVmFyMTRfUkJDXzJoPC1kYXRhSE1tUGxvdCRWYXIxNF9SQkNfMmgtZGF0YUhNbVBsb3QkVmFyMTRfUkJDXzBoCmRhdGFITW1QbG90JFZhcjE0X1JCQ182aDwtZGF0YUhNbVBsb3QkVmFyMTRfUkJDXzZoLWRhdGFITW1QbG90JFZhcjE0X1JCQ18waApkYXRhSE1tUGxvdCRWYXIxNF9SQkNfMjBoPC1kYXRhSE1tUGxvdCRWYXIxNF9SQkNfMjBoLWRhdGFITW1QbG90JFZhcjE0X1JCQ18waApkYXRhSE1tUGxvdCRWYXIxNF9SQkNfMGg8LWRhdGFITW1QbG90JFZhcjE0X1JCQ18waC1kYXRhSE1tUGxvdCRWYXIxNF9SQkNfMGgKCmRhdGFITW1QbG90PC1hcy5tYXRyaXgoZGF0YUhNbVBsb3QpCiAgCmhtYXBfaGllcl9mYWN0b3JzNiA8LSBIZWF0bWFwKAogIGRhdGFITW1QbG90LCAgbmFtZSA9ICJOb3JtYWxpc2VkIGxvZ2ZjIiwKICByb3dfbGFiZWxzID0gcGFzdGUwKHJvd25hbWVzKGRhdGFITW0pLCIgIiwodGVtcEFbIHRvcERFZ2VuZXMsIF0pJEdlbmVfU3ltYm9sKSwKICBjb2x1bW5fdGl0bGUgPSBwYXN0ZTAoIjBoIE5vcm1hbGlzZWQgbG9nZmMiKSwgCiAgY29sID0gY29sX2Z1bkdSMiwKICBjb2x1bW5fdGl0bGVfZ3AgPSBncGFyKGZvbnRzaXplID0gMTYsIGZvbnRmYWNlID0gImJvbGQiKSwKICB3aWR0aCA9IHVuaXQoNTAsICJtbSIpLAogIGNsdXN0ZXJfY29sdW1ucyA9IEZBTFNFLAogIHNob3dfcm93X25hbWVzID0gRkFMU0UpCgojIyMjbWVhbnMKZGF0YUhNbTM8LWJhc2VNZWFuc0htTVsgdG9wREVnZW5lcywgXQojZGF0YUhNbSA8LSBsb2cyKGRhdGFITW0rMSkKZGF0YUhNbTM8LSB0KGFzLm1hdHJpeChkYXRhSE1tMykpCmRhdGFITW0zIDwtIHQoc2NhbGUoZGF0YUhNbTMpKQpkYXRhSE1tMzwtZGF0YUhNbTNbLGMoNSwxLDYsMiw3LDMsOCw0KV0KaG1hcF9oaWVyX2ZhY3RvcnM1IDwtIEhlYXRtYXAoCiAgZGF0YUhNbTMsICBuYW1lID0gIkV4cHJlc3Npb24iLAogIHJvd19sYWJlbHMgPSBwYXN0ZTAocm93bmFtZXMoZGF0YUhNbSksIiAiLCh0ZW1wQVsgdG9wREVnZW5lcywgXSkkR2VuZV9TeW1ib2wpLAogIGNvbHVtbl90aXRsZSA9IHBhc3RlMCgiTWVhbnMiKSwgCiAgY29sID0gY29sX2Z1biwKICBjb2x1bW5fdGl0bGVfZ3AgPSBncGFyKGZvbnRzaXplID0gMTYsIGZvbnRmYWNlID0gImJvbGQiKSwKICB3aWR0aCA9IHVuaXQoMTAwLCAibW0iKSwKICBjbHVzdGVyX2NvbHVtbnMgPSBGQUxTRSwKICBzaG93X3Jvd19uYW1lcyA9IEZBTFNFKQogIApobWFwX2hpZXJfZmFjdG9yczQraG1hcF9oaWVyX2ZhY3RvcnM2K2htYXBfaGllcl9mYWN0b3JzNQpgYGAKCgpgYGB7cixmaWcuaGVpZ2h0ID0gNyxmaWcud2lkdGggPTEyIH0KcGFyKG1mcm93PWMoMSwyKSkKIyMjIyBTaWxob3VldHRlIG1ldGhvZApmdml6X25iY2x1c3QoZGF0YUhNbSwga21lYW5zLCBtZXRob2QgPSAic2lsaG91ZXR0ZSIsay5tYXggPSAxNikrCiAgbGFicyhzdWJ0aXRsZSA9ICJTaWxob3VldHRlIG1ldGhvZCIpCgojIyMjIEVsYm93IG1ldGhvZApmdml6X25iY2x1c3QoZGF0YUhNbSwga21lYW5zLCBtZXRob2QgPSAid3NzIixrLm1heCA9IDE2KSArCiAgbGFicyhzdWJ0aXRsZSA9ICJFbGJvdyBtZXRob2QiKQoKYGBgCgpgYGB7cn0KIyMjI2dhcCBzdGF0IHNsb3chISEKIyMjI3NldC5zZWVkKDEyMykKIyMjI2Z2aXpfbmJjbHVzdChkYXRhSE1tLCBrbWVhbnMsIG5zdGFydCA9IDI1LCAgbWV0aG9kID0gImdhcF9zdGF0IiwgbmJvb3QgPSAxMDAsay5tYXggPSAxNikrCiMjIyMgIGxhYnMoc3VidGl0bGUgPSAiR2FwIHN0YXRpc3RpYyBtZXRob2QiKQpgYGAKCgpgYGB7cixmaWcuaGVpZ2h0ID0gN30KI2tjbHVzdDcgPC0ga21lYW5zKGRhdGFITW0sIDYpCiNzaWxob3VldHRlIHBsb3QKZGlzdEs8LWRhaXN5KGRhdGFITW0pCnBsb3Qoc2lsaG91ZXR0ZShrY2x1c3Q3JGNsdXN0ZXIsIGRpc3RLKSwgY29sPTE6NiwgYm9yZGVyPU5BKQoKYGBgCgoKIyMjIzMuIEstbWVhbnMgY2x1c3RlcmluZyBvZiBtZWFucyAKCmBgYHtyLGZpZy5oZWlnaHQgPSAxMCxmaWcud2lkdGggPSAyMH0Kc3BsaXQgPC0gcGFzdGUwKCJDbHVzdGVyXG4iLCBrY2x1c3Q3JGNsdXN0ZXIpCiNzcGxpdCA8LSBmYWN0b3IocGFzdGUwKCJDbHVzdGVyXG4iLCBrY2x1c3QzJGNsdXN0ZXIpLCBsZXZlbHM9YygiQ2x1c3RlclxuMyIsIkNsdXN0ZXJcbjEiLCJDbHVzdGVyXG40IiwiQ2x1c3RlclxuNSIsIkNsdXN0ZXJcbjIiLCJDbHVzdGVyXG42IikpCmhtYXBfayA8LSBIZWF0bWFwKGRhdGFITW0sIHNwbGl0PXNwbGl0LCBjbHVzdGVyX3Jvd19zbGljZXMgPSBGQUxTRSwKICAgICAgICAgICAgICAgICAgY2x1c3Rlcl9jb2x1bW5zID0gRkFMU0UsCiAgICAgICAgICAgICAgICAgIHNob3dfcm93X25hbWVzID0gRkFMU0UsCiAgICAgICAgICAgICAgICAgIG5hbWUgPSAibG9nZmMiLAogICAgICAgICAgICAgICAgICBjb2wgPSBjb2xfZnVuR1IsCiAgICAgICAgICAgICAgICAgIHdpZHRoID0gdW5pdCg1MCwgIm1tIiksCiAgICAgICAgICAgICAgICAgIGNvbHVtbl90aXRsZSA9ICJtZWFucyBsb2dmYyIsIAogICAgICAgICAgICAgICAgICBjb2x1bW5fdGl0bGVfZ3AgPSBncGFyKGZvbnRzaXplID0gMTYsIGZvbnRmYWNlID0gImJvbGQiKSkKCmhtYXBfaytobWFwX2hpZXJfZmFjdG9yczYraG1hcF9oaWVyX2ZhY3RvcnM1CmBgYAoKCk1lYW4gcHJvZmlsZXMgb2YgY2x1c3RlcnMKCmBgYHtyLGZpZy5oZWlnaHQgPSA4LGZpZy53aWR0aCA9IDh9CmNsdXN0ZXJjb3VudDwtZGF0YS5mcmFtZShrY2x1c3Q3JGNsdXN0ZXIpCmNsdXN0ZXJzaXplczwtdGFibGUoY2x1c3RlcmNvdW50JGtjbHVzdDcuY2x1c3RlcikKY2x1c3Rlck1lYW5zPC1kYXRhLmZyYW1lKGtjbHVzdDckY2VudGVycykKY2x1c3Rlck1lYW5zMTwtZGF0YS5mcmFtZSh0KGNsdXN0ZXJNZWFucykpCmNsdXN0ZXJNZWFuczEgPC0gY2JpbmQocm93bmFtZXMoY2x1c3Rlck1lYW5zMSksIGNsdXN0ZXJNZWFuczEpCm9yZGVyTjwtYygiVmFyMTRfUkJDXzBoIiwiVmFyMTRfUkJDXzJoIiwiVmFyMTRfUkJDXzZoIiwiVmFyMTRfUkJDXzIwaCIpIyMjIyBtYW51YWwKcm93bmFtZXMoY2x1c3Rlck1lYW5zMSkgPC0gTlVMTApuYW1lcyhjbHVzdGVyTWVhbnMxKVtuYW1lcyhjbHVzdGVyTWVhbnMxKT09InJvd25hbWVzKGNsdXN0ZXJNZWFuczEpIl0gPC0gIlNhbXBsZSIKCnBYMTwtZ2dwbG90KGRhdGE9Y2x1c3Rlck1lYW5zMSwgYWVzKHg9U2FtcGxlLCB5PVgxLGdyb3VwPTEpKSArCiAgZ2VvbV9saW5lKCkrICBnZW9tX3BvaW50KCkrZ2d0aXRsZShwYXN0ZSgiQ2x1c3RlciBYMSBQcm9maWxlICIsY2x1c3RlcnNpemVzWzFdLCIgZ2VuZXMiKSkrICBzY2FsZV94X2Rpc2NyZXRlKGxpbWl0cz1vcmRlck4pKwogIHRoZW1lKGF4aXMudGl0bGUueCA9IGVsZW1lbnRfYmxhbmsoKSxheGlzLnRpdGxlLnkgPSBlbGVtZW50X2JsYW5rKCkpCnBYMjwtZ2dwbG90KGRhdGE9Y2x1c3Rlck1lYW5zMSwgYWVzKHg9U2FtcGxlLCB5PVgyLGdyb3VwPTEpKSArCiAgZ2VvbV9saW5lKCkrICBnZW9tX3BvaW50KCkrZ2d0aXRsZShwYXN0ZSgiQ2x1c3RlciBYMiBQcm9maWxlICIsY2x1c3RlcnNpemVzWzJdLCIgZ2VuZXMiKSkrICBzY2FsZV94X2Rpc2NyZXRlKGxpbWl0cz1vcmRlck4pKwogIHRoZW1lKGF4aXMudGl0bGUueCA9IGVsZW1lbnRfYmxhbmsoKSxheGlzLnRpdGxlLnkgPSBlbGVtZW50X2JsYW5rKCkpCnBYMzwtZ2dwbG90KGRhdGE9Y2x1c3Rlck1lYW5zMSwgYWVzKHg9U2FtcGxlLCB5PVgzLGdyb3VwPTEpKSArCiAgZ2VvbV9saW5lKCkrICBnZW9tX3BvaW50KCkrZ2d0aXRsZShwYXN0ZSgiQ2x1c3RlciBYMyBQcm9maWxlICIsY2x1c3RlcnNpemVzWzNdLCIgZ2VuZXMiKSkrICBzY2FsZV94X2Rpc2NyZXRlKGxpbWl0cz1vcmRlck4pKwogIHRoZW1lKGF4aXMudGl0bGUueCA9IGVsZW1lbnRfYmxhbmsoKSxheGlzLnRpdGxlLnkgPSBlbGVtZW50X2JsYW5rKCkpCnBYNDwtZ2dwbG90KGRhdGE9Y2x1c3Rlck1lYW5zMSwgYWVzKHg9U2FtcGxlLCB5PVg0LGdyb3VwPTEpKSArCiAgZ2VvbV9saW5lKCkrICBnZW9tX3BvaW50KCkrZ2d0aXRsZShwYXN0ZSgiQ2x1c3RlciBYNCBQcm9maWxlICIsY2x1c3RlcnNpemVzWzRdLCIgZ2VuZXMiKSkrICBzY2FsZV94X2Rpc2NyZXRlKGxpbWl0cz1vcmRlck4pKwogIHRoZW1lKGF4aXMudGl0bGUueCA9IGVsZW1lbnRfYmxhbmsoKSxheGlzLnRpdGxlLnkgPSBlbGVtZW50X2JsYW5rKCkpCnBYNTwtZ2dwbG90KGRhdGE9Y2x1c3Rlck1lYW5zMSwgYWVzKHg9U2FtcGxlLCB5PVg1LGdyb3VwPTEpKSArCiAgZ2VvbV9saW5lKCkrICBnZW9tX3BvaW50KCkrZ2d0aXRsZShwYXN0ZSgiQ2x1c3RlciBYNSBQcm9maWxlICIsY2x1c3RlcnNpemVzWzVdLCIgZ2VuZXMiKSkrICBzY2FsZV94X2Rpc2NyZXRlKGxpbWl0cz1vcmRlck4pKwogIHRoZW1lKGF4aXMudGl0bGUueCA9IGVsZW1lbnRfYmxhbmsoKSxheGlzLnRpdGxlLnkgPSBlbGVtZW50X2JsYW5rKCkpCnBYNjwtZ2dwbG90KGRhdGE9Y2x1c3Rlck1lYW5zMSwgYWVzKHg9U2FtcGxlLCB5PVg2LGdyb3VwPTEpKSArCiAgZ2VvbV9saW5lKCkrICBnZW9tX3BvaW50KCkrZ2d0aXRsZShwYXN0ZSgiQ2x1c3RlciBYNiBQcm9maWxlICIsY2x1c3RlcnNpemVzWzZdLCIgZ2VuZXMiKSkrICBzY2FsZV94X2Rpc2NyZXRlKGxpbWl0cz1vcmRlck4pKwogIHRoZW1lKGF4aXMudGl0bGUueCA9IGVsZW1lbnRfYmxhbmsoKSxheGlzLnRpdGxlLnkgPSBlbGVtZW50X2JsYW5rKCkpCiNwbG90Cm11bHRpcGxvdChwWDEsIHBYMiwgcFgzLCBwWDQscFg1LCBwWDYsIGNvbHM9MikKCmBgYAoKCmBgYHtyfQp0b3BERWdlbmVzIDwtIHdoaWNoKHRlbXBBJEluY2x1ZGU9PSJpbiIpIyMjI2ZpbmQgaW5kZXhlcwp0ZW1wQWttPC10ZW1wQVsgdG9wREVnZW5lcywgXQpTeW1ib2xzS208LWRwbHlyOjpwdWxsKHRlbXBBa20sIEdlbmVfU3ltYm9sKQojIyMjIGV4cG9ydCB0aGUgZ2VuZSBleHByZXNzaW9uIGRhdGEgZm9yIHRoZSBjbHVzdGVycwp3cml0ZS50YWJsZShjbHVzdGVyTWVhbnMscGFzdGUwKCJDbHVzdGVyTWVhbnNLbV8iLGdyb3Vwc05hbWUsIi50eHQiKSwgIHNlcCA9ICJcdCIpCkNsdXN0ZXJlZEdlbmVzPC1kYXRhLmZyYW1lKGtjbHVzdDckY2x1c3RlcixTeW1ib2xzS20sZGF0YUhNbSkKd3JpdGUudGFibGUoQ2x1c3RlcmVkR2VuZXMscGFzdGUwKCJTY2FsZWREYXRhSW5DbHVzdGVyc0ttXyIsZ3JvdXBzTmFtZSwiLnR4dCIpLCAgc2VwID0gIlx0IikKI2hlYWQoQ2x1c3RlcmVkR2VuZXMpCmBgYAoKCmBgYHtyfQpib3R0b21ERWdlbmVzPC13aGljaCh0ZW1wQSRJbmNsdWRlPT0ib3V0IikjIyMjZmluZCBpbmRleGVzIApib3R0b21HPC10ZW1wQVsgYm90dG9tREVnZW5lcywgXQpib3R0b21HPC1kcGx5cjo6cHVsbChib3R0b21HLCBHZW5lX1N5bWJvbCkKd3JpdGUudGFibGUoYm90dG9tRyxwYXN0ZTAoImlwYUJvdHRvbUttZWFuc18iLGdyb3Vwc05hbWUsIi50eHQiKSwgIHNlcCA9ICJcdCIpCiAgICAgICAgICAgICAgICAgICAgICAgICAKCnRvcERFZ2VuZXMgPC0gd2hpY2godGVtcEEkSW5jbHVkZT09ImluIikjIyMjZmluZCBpbmRleGVzIAp0ZW1wQWttPC10ZW1wQVsgdG9wREVnZW5lcywgXQpTeW1ib2xzS208LWRwbHlyOjpwdWxsKHRlbXBBa20sIEdlbmVfU3ltYm9sKQoKaXBhS21lYW5zPC1DbHVzdGVyZWRHZW5lcwojY291bnRzVGFibGUgPC1jb3VudHNUYWJsZVssYygxOjE1KV0jIyMjaWYgc2FtcGxlcyBuZWVkIHJlbW92aW5nCmlwYUttZWFuczwtaXBhS21lYW5zWyxjKDE6MildCmlwYUttZWFucyRuYW1lMjwtcm93bmFtZXMoaXBhS21lYW5zKQojaXBhS21lYW5zJT4lIHJvd25hbWVzX3RvX2NvbHVtbih2YXIgPSAicm93bmFtZSIpCiNpcGFLbWVhbnMKI3Jvd2lkX3RvX2NvbHVtbihpcGFLbWVhbnMpCmlwYUttZWFucyA9IG11dGF0ZShpcGFLbWVhbnMsIHgxPSBpZmVsc2UoaXBhS21lYW5zJGtjbHVzdDcuY2x1c3Rlcj09MSwgIjEiLCAiMCIpKQppcGFLbWVhbnMgPSBtdXRhdGUoaXBhS21lYW5zLCB4Mj0gaWZlbHNlKGlwYUttZWFucyRrY2x1c3Q3LmNsdXN0ZXI9PTIsICIxIiwgIjAiKSkKaXBhS21lYW5zID0gbXV0YXRlKGlwYUttZWFucywgeDM9IGlmZWxzZShpcGFLbWVhbnMka2NsdXN0Ny5jbHVzdGVyPT0zLCAiMSIsICIwIikpCmlwYUttZWFucyA9IG11dGF0ZShpcGFLbWVhbnMsIHg0PSBpZmVsc2UoaXBhS21lYW5zJGtjbHVzdDcuY2x1c3Rlcj09NCwgIjEiLCAiMCIpKQppcGFLbWVhbnMgPSBtdXRhdGUoaXBhS21lYW5zLCB4NT0gaWZlbHNlKGlwYUttZWFucyRrY2x1c3Q3LmNsdXN0ZXI9PTUsICIxIiwgIjAiKSkKaXBhS21lYW5zID0gbXV0YXRlKGlwYUttZWFucywgeDY9IGlmZWxzZShpcGFLbWVhbnMka2NsdXN0Ny5jbHVzdGVyPT02LCAiMSIsICIwIikpCiNpcGFLbWVhbnMKd3JpdGUudGFibGUoaXBhS21lYW5zLHBhc3RlMCgiaXBhS21lYW5zXyIsZ3JvdXBzTmFtZSwiLnR4dCIpLCAgc2VwID0gIlx0IikKI2hlYWQoaXBhS21lYW5zKQoKYGBgCgoKYGBge3J9CkNsdXN0ZXJlZEdlbmVzMjwtQ2x1c3RlcmVkR2VuZXNbYygxKV0KI0NsdXN0ZXJlZEdlbmVzMgpsaXN0QWxsPC1saXN0KCkKZm9yKGkgaW4gMTo2KSB7CiAgY2x1c3Rlck5hbWU8LXBhc3RlMCgieCIsaSkKICAjY2x1c3Rlck5hbWU8LXJvdy5uYW1lcyhzdWJzZXQoQ2x1c3RlcmVkR2VuZXMsQ2x1c3RlcmVkR2VuZXM9PWkpKQogIGNsdXN0ZXJOYW1lPC0oc3Vic2V0KENsdXN0ZXJlZEdlbmVzJFN5bWJvbHNLbSxDbHVzdGVyZWRHZW5lcz09aSkpCiAgbGlzdEFsbFtbaV1dPC1jbHVzdGVyTmFtZQp9CiNuZWVkIHRvIG5hbWUgdGhlIHZlY3RvcnMgaW4gdGhlIGxpc3QsIGV4YW1wbGUgaGVyZSBpcyBmb3IgOCBjbHVzdGVycwpuYW1lcyhsaXN0QWxsKTwtYygiWDEiLCAiWDIiLCAiWDMiLCAiWDQiLCJYNSIsICJYNiIpCgojaWYgeW91IHdhbnQgdG8gcmVhcnJhbmdlIHRoZSBvcmRlcgojbGlzdEFsbDwtbGlzdEFsbFtjKCJ4MyIsICJ4NyIsICJ4OCIsICJ4MiIsICJ4NiIsICJ4NSIsICJ4NCIsICJ4MSIpXQoKI2xhcHBseShsaXN0QWxsLCBoZWFkKQoKYGBgCgojIyMjNC4gQW5ub3RhdGlvbiBvZiBLLW1lYW5zIGNsdXN0ZXJzCi0gQ0MgY2VsbHVsYXIgY29tcGFydG1lbnQKLSBCUCBiaW9sb2dpY2FsIHByb2Nlc3MKLSBNRiBtb2xlY3VsYXIgZnVuY3Rpb24KClRoZSBzaW1wbGlmeSBmdW5jdGlvbiBoYXMgYmVlbiB1c2VkIHRvIGN1dCBkb3duIG9uIEdPIHJlZHVuZGFuY3kKCmBgYHtyfQojc3RyKEFsbEdlbmVOYW1lcykKYGBgCgoKYGBge3IsZmlnLmhlaWdodCA9IDgsZmlnLndpZHRoID0gMTJ9CiMjIyNDQwpjZ29DQyA8LSBjb21wYXJlQ2x1c3RlcihnZW5lQ2x1c3RlciA9IGxpc3RBbGwsIAogICAgICAgICAgICAgICAgICAgICAgdW5pdmVyc2UgPSBBbGxHZW5lTmFtZXMsCiAgICAgICAgICAgICAgICAgICAgICBmdW4gPSAiZW5yaWNoR08iLAogICAgICAgICAgICAgICAgICAgICAgT3JnRGI9b3JnLkhzLmVnLmRiLCAKICAgICAgICAgICAgICAgICAgICAgICMjIyNPcmdEYj1vcmcuTW0uZWcuZGIsCiAgICAgICAgICAgICAgICAgICAgICBrZXlUeXBlPSJTWU1CT0wiLAogICAgICAgICAgICAgICAgICAgICAgb250ID0gIkNDIiwgCiAgICAgICAgICAgICAgICAgICAgICBwdmFsdWVDdXRvZmY9MC4wNSwKICAgICAgICAgICAgICAgICAgICAgIHF2YWx1ZUN1dG9mZiA9IDAuMTApCmNnb0NDMiA8LSBzaW1wbGlmeShjZ29DQywgY3V0b2ZmPTAuNywgYnk9InAuYWRqdXN0Iiwgc2VsZWN0X2Z1bj1taW4pCiMjIyN3cml0ZSBhcyBzcHJlYWRzaGVldAp3cml0ZS5jc3YoYXMuZGF0YS5mcmFtZShjZ29DQzIpLHBhc3RlMCgiR09fQ0NfIixncm91cHNOYW1lLCIuY3N2IikpCgpkb3RwbG90KGNnb0NDMixzaG93Q2F0ZWdvcnkgPSAzMCwKICAgICAgICB0aXRsZSA9IHBhc3RlMCgiR08gQ2VsbHVsYXIgQ29tcGFydG1lbnQgIixncm91cHNOYW1lKSkrCiAgdGhlbWUoYXhpcy50ZXh0LnggPSBlbGVtZW50X3RleHQoYW5nbGUgPSA5MCwgdmp1c3QgPSAwLjUsIGhqdXN0PTEpKQoKYGBgClBsb3RzIGFuZCBHTyBkYXRhIHdlcmUgd3JpdHRlbiB0byBmaWxlcwpgYGB7cn0KcG5nKHBhc3RlMCgiR09fQ0NfIixncm91cHNOYW1lLCIucG5nIiksIHdpZHRoID0gMTIyNCwgaGVpZ2h0ID0gODI0KQpkb3RwbG90KGNnb0NDMixzaG93Q2F0ZWdvcnkgPSAzMCwKICAgICAgICB0aXRsZSA9IHBhc3RlMCgiR08gQ2VsbHVsYXIgQ29tcGFydG1lbnQgIixncm91cHNOYW1lKSkrCiAgdGhlbWUoYXhpcy50ZXh0LnggPSBlbGVtZW50X3RleHQoYW5nbGUgPSA5MCwgdmp1c3QgPSAwLjUsIGhqdXN0PTEpKQpkZXYub2ZmKCkKYGBgCgpHTyBCUApgYGB7cixmaWcuaGVpZ2h0ID0gMTIsZmlnLndpZHRoID0gMTJ9CiMjIyNDQwpjZ29CUCA8LSBjb21wYXJlQ2x1c3RlcihnZW5lQ2x1c3RlciA9IGxpc3RBbGwsIAogICAgICAgICAgICAgICAgICAgICAgdW5pdmVyc2UgPSBBbGxHZW5lTmFtZXMsCiAgICAgICAgICAgICAgICAgICAgICBmdW4gPSAiZW5yaWNoR08iLAogICAgICAgICAgICAgICAgICAgICAgT3JnRGI9b3JnLkhzLmVnLmRiLAogICAgICAgICAgICAgICAgICAgICAga2V5VHlwZT0iU1lNQk9MIiwKICAgICAgICAgICAgICAgICAgICAgIG9udCA9ICJCUCIsIAogICAgICAgICAgICAgICAgICAgICAgcHZhbHVlQ3V0b2ZmPTAuMDUsCiAgICAgICAgICAgICAgICAgICAgICBxdmFsdWVDdXRvZmYgPSAwLjEwKQpjZ29CUDIgPC0gc2ltcGxpZnkoY2dvQlAsIGN1dG9mZj0wLjcsIGJ5PSJwLmFkanVzdCIsIHNlbGVjdF9mdW49bWluKQojIyMjd3JpdGUgYXMgc3ByZWFkc2hlZXQKd3JpdGUuY3N2KGFzLmRhdGEuZnJhbWUoY2dvQlAyKSxwYXN0ZTAoIkdPX0JQXyIsZ3JvdXBzTmFtZSwiLmNzdiIpKQoKZG90cGxvdChjZ29CUDIsc2hvd0NhdGVnb3J5ID0gMzAsCiAgICAgICAgdGl0bGUgPSBwYXN0ZTAoIkdPIEJpb2xvZ2ljYWwgUHJvY2VzcyAiLGdyb3Vwc05hbWUpKSsKICB0aGVtZShheGlzLnRleHQueCA9IGVsZW1lbnRfdGV4dChhbmdsZSA9IDkwLCB2anVzdCA9IDAuNSwgaGp1c3Q9MSkpCgpgYGAKCmBgYHtyfQpwbmcocGFzdGUwKCJHT19CUF8iLGdyb3Vwc05hbWUsIi5wbmciKSwgd2lkdGggPSAxMDI0LCBoZWlnaHQgPSAxMjI0KQpkb3RwbG90KGNnb0JQMixzaG93Q2F0ZWdvcnkgPSAzMCwKICAgICAgICB0aXRsZSA9IHBhc3RlMCgiR08gQmlvbG9naWNhbCBQcm9jZXNzICIsZ3JvdXBzTmFtZSkpKwogIHRoZW1lKGF4aXMudGV4dC54ID0gZWxlbWVudF90ZXh0KGFuZ2xlID0gOTAsIHZqdXN0ID0gMC41LCBoanVzdD0xKSkKZGV2Lm9mZigpCmBgYAoKCkdPIE1GCmBgYHtyLGZpZy5oZWlnaHQgPSAxMCxmaWcud2lkdGggPSAxMn0KIyMjI01GCmNnb01GIDwtIGNvbXBhcmVDbHVzdGVyKGdlbmVDbHVzdGVyID0gbGlzdEFsbCwgCiAgICAgICAgICAgICAgICAgICAgICB1bml2ZXJzZSA9IEFsbEdlbmVOYW1lcywKICAgICAgICAgICAgICAgICAgICAgIGZ1biA9ICJlbnJpY2hHTyIsCiAgICAgICAgICAgICAgICAgICAgICBPcmdEYj1vcmcuSHMuZWcuZGIsIAogICAgICAgICAgICAgICAgICAgICAga2V5VHlwZT0iU1lNQk9MIiwKICAgICAgICAgICAgICAgICAgICAgIG9udCA9ICJNRiIsIAogICAgICAgICAgICAgICAgICAgICAgcHZhbHVlQ3V0b2ZmPTAuMDUsCiAgICAgICAgICAgICAgICAgICAgICBxdmFsdWVDdXRvZmYgPSAwLjEwKQpjZ29NRjIgPC0gc2ltcGxpZnkoY2dvTUYsIGN1dG9mZj0wLjcsIGJ5PSJwLmFkanVzdCIsIHNlbGVjdF9mdW49bWluKQojIyMjd3JpdGUgYXMgc3ByZWFkc2hlZXQKd3JpdGUuY3N2KGFzLmRhdGEuZnJhbWUoY2dvTUYyKSxwYXN0ZTAoIkdPX01GXyIsZ3JvdXBzTmFtZSwiLmNzdiIpKQoKZG90cGxvdChjZ29NRjIsc2hvd0NhdGVnb3J5ID0gMzAsCiAgICAgICAgdGl0bGUgPSBwYXN0ZTAoIkdPIE1vbGVjdWxhciBGdW5jdGlvbiAgIixncm91cHNOYW1lKSkrCiAgdGhlbWUoYXhpcy50ZXh0LnggPSBlbGVtZW50X3RleHQoYW5nbGUgPSA5MCwgdmp1c3QgPSAwLjUsIGhqdXN0PTEpKQoKYGBgCgoKCmBgYHtyfQpwbmcocGFzdGUwKCJHT19NRl8iLGdyb3Vwc05hbWUsIi5wbmciKSwgd2lkdGggPSAxNDI0LCBoZWlnaHQgPSA2MjQpCmRvdHBsb3QoY2dvTUYyLHNob3dDYXRlZ29yeSA9IDMwLAogICAgICAgIHRpdGxlID0gcGFzdGUwKCJHTyBNb2xlY3VsYXIgRnVuY3Rpb24gICIsZ3JvdXBzTmFtZSkpKwogIHRoZW1lKGF4aXMudGV4dC54ID0gZWxlbWVudF90ZXh0KGFuZ2xlID0gOTAsIHZqdXN0ID0gMC41LCBoanVzdD0xKSkKZGV2Lm9mZigpCmBgYAoKCgoKCiMjIyBSNSBWQVIxNCB2cyBSQkMgVE5GIGstbWVhbnMgcDAuMDVmYzIKCiMjIyMxLiBHZW5lbGlzdCBTZWxlY3Rpb24KCmBgYHtyfQpncm91cHNOYW1lPC0iUjVfVmFyMTR2UkJDX1RORl9rbWVhbnNfcDAuMDVmYzIiCmBgYAoKCmBgYHtyfQpjb3VudHNUYWJsZTwtcmVhZC5kZWxpbSgiUk5Bc2VxMjAxOUp1bHlfNS50eHQiLCBoZWFkZXIgPSBUUlVFLCBzZXAgPSAiXHQiLGNoZWNrLm5hbWVzPUZBTFNFLHJvdy5uYW1lcz0xKQpoZWFkKGNvdW50c1RhYmxlKQpgYGAKCmBgYHtyfQpBbGxHZW5lTmFtZXM8LWNvdW50c1RhYmxlJEdlbmVfU3ltYm9sCiNoZWFkKEFsbEdlbmVOYW1lcykKYGBgCgpgYGB7cixmaWcuaGVpZ2h0ID0gMTAsZmlnLndpZHRoID0gMjB9CmdyaWQuYXJyYW5nZShnVHJlZShjaGlsZHJlbj12ZW5ucCksIGdUcmVlKGNoaWxkcmVuPXZlbm5wcSkgLCBuY29sPTIsdG9wPSJSNSBWYXIxNCBUTkYiKQpgYGAKCmBgYHtyfQojdGVtcEE8LXJlc0FsbFstYygxMDozMCkgXQp0ZW1wQTwtY291bnRzVGFibGUKI3Jvd25hbWVzKHRlbXBBKQpyb3duYW1lcyh0ZW1wQSkgPC0gTlVMTAp0ZW1wQSA9IG11dGF0ZSh0ZW1wQSwgSW5jbHVkZT0KICAgICAgICAgICAgICAgICAgIGlmZWxzZSh0ZW1wQSRwdmFsdWVfUjVfVE5GX3ZhcjE0X3ZzX1JCQ18waDwwLjA1JmFicyh0ZW1wQSRsb2cyRm9sZENoYW5nZV9SNV9UTkZfdmFyMTRfdnNfUkJDXzBoKT4xJiFpcy5uYSh0ZW1wQSRwdmFsdWVfUjVfVE5GX3ZhcjE0X3ZzX1JCQ18waCksICJpbiIsCiAgICAgICAgICAgICAgICAgICAgICAgICAgaWZlbHNlKHRlbXBBJHB2YWx1ZV9SNV9UTkZfdmFyMTRfdnNfUkJDXzJoPDAuMDUmYWJzKHRlbXBBJGxvZzJGb2xkQ2hhbmdlX1I1X1RORl92YXIxNF92c19SQkNfMmgpPjEmIWlzLm5hKHRlbXBBJHB2YWx1ZV9SNV9UTkZfdmFyMTRfdnNfUkJDXzJoKSwgImluIiwKICAgICAgICAgICAgICAgICAgICAgICAgICAgICAgICAgaWZlbHNlKHRlbXBBJHB2YWx1ZV9SNV9UTkZfdmFyMTRfdnNfUkJDXzZoPDAuMDUmYWJzKHRlbXBBJGxvZzJGb2xkQ2hhbmdlX1I1X1RORl92YXIxNF92c19SQkNfNmgpPjEmIWlzLm5hKHRlbXBBJHB2YWx1ZV9SNV9UTkZfdmFyMTRfdnNfUkJDXzZoKSwgImluIiwKICAgICAgICAgICAgICAgICAgICAgICAgICAgICAgICAgICAgICAgIGlmZWxzZSh0ZW1wQSRwdmFsdWVfUjVfVE5GX3ZhcjE0X3ZzX1JCQ18yMGg8MC4wNSZhYnModGVtcEEkbG9nMkZvbGRDaGFuZ2VfUjVfVE5GX3ZhcjE0X3ZzX1JCQ18yMGgpPjEmIWlzLm5hKHRlbXBBJHB2YWx1ZV9SNV9UTkZfdmFyMTRfdnNfUkJDXzIwaCksICJpbiIsCiAgICAgICAgICAgICAgICAgICAgICAgICAgICAgICAgICAgICAgICAgICAgICAgICAgICAgICAgICAgICAgICAgICAgICAgIm91dCIpKSkpKQoKCiN0ZW1wQQojIyMjbGlicmFyeShkcGx5cikKdGVtcEEgJT4lCiAgICAgZ3JvdXBfYnkoSW5jbHVkZSkgJT4lIAogICAgIHRhbGx5KCkKCmBgYAoKYGBge3J9CnRvcERFZ2VuZXMgPC0gd2hpY2godGVtcEEkSW5jbHVkZT09ImluIikjIyMjZmluZCBpbmRleGVzIApgYGAKCgojIyMjIE5CIFBsZWFzZSBjaGVjayBjb2x1bW5zIHVzZWQgYW5kIHJlbmFtZWQgZm9yIHBsb3RzCmBgYHtyfQojYmFzZU1lYW5zSG0gPC1jb3VudHNUYWJsZVssYyg2MDo2MyldCmJhc2VNZWFuc0htIDwtY291bnRzVGFibGVbLGMoMTEwOjExMywxMjk6MTMyKV0KaGVhZChiYXNlTWVhbnNIbSkKYmFzZU1lYW5zSG0yIDwtIGxvZzIoYmFzZU1lYW5zSG0rMSkKYmFzZU1lYW5zSG0yJFZhcjE0X1JCQ18waDwtYmFzZU1lYW5zSG0yJFZhcjE0VE5GXzBoX21lYW4tYmFzZU1lYW5zSG0yJFJCQ19UTkZfMGhfbWVhbgpiYXNlTWVhbnNIbTIkVmFyMTRfUkJDXzJoPC1iYXNlTWVhbnNIbTIkVmFyMTRUTkZfMmhfbWVhbi1iYXNlTWVhbnNIbTIkUkJDX1RORl8yaF9tZWFuCmJhc2VNZWFuc0htMiRWYXIxNF9SQkNfNmg8LWJhc2VNZWFuc0htMiRWYXIxNFRORl82aF9tZWFuLWJhc2VNZWFuc0htMiRSQkNfVE5GXzZoX21lYW4KYmFzZU1lYW5zSG0yJFZhcjE0X1JCQ18yMGg8LWJhc2VNZWFuc0htMiRWYXIxNFRORl8yMGhfbWVhbi1iYXNlTWVhbnNIbTIkUkJDX1RORl8yMGhfbWVhbgpiYXNlTWVhbnNIbSA8LWJhc2VNZWFuc0htMlssYyg5OjEyKV0KaGVhZChiYXNlTWVhbnNIbSkKYmFzZU1lYW5zSG1NIDwtYmFzZU1lYW5zSG0yWyxjKDE6OCldCmhlYWQoYmFzZU1lYW5zSG1NKQpgYGAKCgoKYGBge3J9CnRvcERFZ2VuZXMgPC0gd2hpY2godGVtcEEkSW5jbHVkZT09ImluIikjIyMjZmluZCBpbmRleGVzIApgYGAKCgojIyMjMi4gSGllcmFjaGljYWwgY2x1c3RlcmluZyBvZiBtZWFucyAKYGBge3IsZmlnLmhlaWdodCA9IDEyLGZpZy53aWR0aCA9IDIwfQojIyMjbWVhbiBsb2dmYwpkYXRhSE1tPC1iYXNlTWVhbnNIbVsgdG9wREVnZW5lcywgXQojZGF0YUhNbSA8LSBsb2cyKGRhdGFITW0rMSkKZGF0YUhNbTwtIHQoYXMubWF0cml4KGRhdGFITW0pKQpkYXRhSE1tIDwtIHQoc2NhbGUoZGF0YUhNbSkpCgpobWFwX2hpZXJfZmFjdG9yczQgPC0gSGVhdG1hcCgKICBkYXRhSE1tLCAgbmFtZSA9ICJsb2dmYyIsCiAgcm93X2xhYmVscyA9IHBhc3RlMChyb3duYW1lcyhkYXRhSE1tKSwiICIsKHRlbXBBWyB0b3BERWdlbmVzLCBdKSRHZW5lX1N5bWJvbCksCiAgY29sdW1uX3RpdGxlID0gcGFzdGUwKCJNZWFucyBsb2dmYyIpLCAKICBjb2wgPSBjb2xfZnVuR1IsCiAgY29sdW1uX3RpdGxlX2dwID0gZ3Bhcihmb250c2l6ZSA9IDE2LCBmb250ZmFjZSA9ICJib2xkIiksCiAgd2lkdGggPSB1bml0KDUwLCAibW0iKSwKICBjbHVzdGVyX2NvbHVtbnMgPSBGQUxTRSwKICBzaG93X3Jvd19uYW1lcyA9IEZBTFNFKQoKZGF0YUhNbVBsb3Q8LWFzLmRhdGEuZnJhbWUoZGF0YUhNbSkKZGF0YUhNbVBsb3QkVmFyMTRfUkJDXzJoPC1kYXRhSE1tUGxvdCRWYXIxNF9SQkNfMmgtZGF0YUhNbVBsb3QkVmFyMTRfUkJDXzBoCmRhdGFITW1QbG90JFZhcjE0X1JCQ182aDwtZGF0YUhNbVBsb3QkVmFyMTRfUkJDXzZoLWRhdGFITW1QbG90JFZhcjE0X1JCQ18waApkYXRhSE1tUGxvdCRWYXIxNF9SQkNfMjBoPC1kYXRhSE1tUGxvdCRWYXIxNF9SQkNfMjBoLWRhdGFITW1QbG90JFZhcjE0X1JCQ18waApkYXRhSE1tUGxvdCRWYXIxNF9SQkNfMGg8LWRhdGFITW1QbG90JFZhcjE0X1JCQ18waC1kYXRhSE1tUGxvdCRWYXIxNF9SQkNfMGgKCmRhdGFITW1QbG90PC1hcy5tYXRyaXgoZGF0YUhNbVBsb3QpCiAgCmhtYXBfaGllcl9mYWN0b3JzNiA8LSBIZWF0bWFwKAogIGRhdGFITW1QbG90LCAgbmFtZSA9ICJOb3JtYWxpc2VkIGxvZ2ZjIiwKICByb3dfbGFiZWxzID0gcGFzdGUwKHJvd25hbWVzKGRhdGFITW0pLCIgIiwodGVtcEFbIHRvcERFZ2VuZXMsIF0pJEdlbmVfU3ltYm9sKSwKICBjb2x1bW5fdGl0bGUgPSBwYXN0ZTAoIjBoIE5vcm1hbGlzZWQgbG9nZmMiKSwgCiAgY29sID0gY29sX2Z1bkdSMiwKICBjb2x1bW5fdGl0bGVfZ3AgPSBncGFyKGZvbnRzaXplID0gMTYsIGZvbnRmYWNlID0gImJvbGQiKSwKICB3aWR0aCA9IHVuaXQoNTAsICJtbSIpLAogIGNsdXN0ZXJfY29sdW1ucyA9IEZBTFNFLAogIHNob3dfcm93X25hbWVzID0gRkFMU0UpCgojIyMjbWVhbnMKZGF0YUhNbTM8LWJhc2VNZWFuc0htTVsgdG9wREVnZW5lcywgXQojZGF0YUhNbSA8LSBsb2cyKGRhdGFITW0rMSkKZGF0YUhNbTM8LSB0KGFzLm1hdHJpeChkYXRhSE1tMykpCmRhdGFITW0zIDwtIHQoc2NhbGUoZGF0YUhNbTMpKQpkYXRhSE1tMzwtZGF0YUhNbTNbLGMoNSwxLDYsMiw3LDMsOCw0KV0KaG1hcF9oaWVyX2ZhY3RvcnM1IDwtIEhlYXRtYXAoCiAgZGF0YUhNbTMsICBuYW1lID0gIkV4cHJlc3Npb24iLAogIHJvd19sYWJlbHMgPSBwYXN0ZTAocm93bmFtZXMoZGF0YUhNbSksIiAiLCh0ZW1wQVsgdG9wREVnZW5lcywgXSkkR2VuZV9TeW1ib2wpLAogIGNvbHVtbl90aXRsZSA9IHBhc3RlMCgiTWVhbnMiKSwgCiAgY29sID0gY29sX2Z1biwKICBjb2x1bW5fdGl0bGVfZ3AgPSBncGFyKGZvbnRzaXplID0gMTYsIGZvbnRmYWNlID0gImJvbGQiKSwKICB3aWR0aCA9IHVuaXQoMTAwLCAibW0iKSwKICBjbHVzdGVyX2NvbHVtbnMgPSBGQUxTRSwKICBzaG93X3Jvd19uYW1lcyA9IEZBTFNFKQogIApobWFwX2hpZXJfZmFjdG9yczQraG1hcF9oaWVyX2ZhY3RvcnM2K2htYXBfaGllcl9mYWN0b3JzNQpgYGAKCgpgYGB7cixmaWcuaGVpZ2h0ID0gNyxmaWcud2lkdGggPTEyIH0KcGFyKG1mcm93PWMoMSwyKSkKIyMjIyBTaWxob3VldHRlIG1ldGhvZApmdml6X25iY2x1c3QoZGF0YUhNbSwga21lYW5zLCBtZXRob2QgPSAic2lsaG91ZXR0ZSIsay5tYXggPSAxNikrCiAgbGFicyhzdWJ0aXRsZSA9ICJTaWxob3VldHRlIG1ldGhvZCIpCgojIyMjIEVsYm93IG1ldGhvZApmdml6X25iY2x1c3QoZGF0YUhNbSwga21lYW5zLCBtZXRob2QgPSAid3NzIixrLm1heCA9IDE2KSArCiAgbGFicyhzdWJ0aXRsZSA9ICJFbGJvdyBtZXRob2QiKQoKYGBgCgpgYGB7cn0KIyMjI2dhcCBzdGF0IHNsb3chISEKIyMjI3NldC5zZWVkKDEyMykKIyMjI2Z2aXpfbmJjbHVzdChkYXRhSE1tLCBrbWVhbnMsIG5zdGFydCA9IDI1LCAgbWV0aG9kID0gImdhcF9zdGF0IiwgbmJvb3QgPSAxMDAsay5tYXggPSAxNikrCiMjIyMgIGxhYnMoc3VidGl0bGUgPSAiR2FwIHN0YXRpc3RpYyBtZXRob2QiKQpgYGAKCgpgYGB7cixmaWcuaGVpZ2h0ID0gN30KI2tjbHVzdDggPC0ga21lYW5zKGRhdGFITW0sIDYpCiNzaWxob3VldHRlIHBsb3QKZGlzdEs8LWRhaXN5KGRhdGFITW0pCnBsb3Qoc2lsaG91ZXR0ZShrY2x1c3Q4JGNsdXN0ZXIsIGRpc3RLKSwgY29sPTE6NiwgYm9yZGVyPU5BKQoKYGBgCgoKIyMjIzMuIEstbWVhbnMgY2x1c3RlcmluZyBvZiBtZWFucyAKCmBgYHtyLGZpZy5oZWlnaHQgPSAxMCxmaWcud2lkdGggPSAyMH0Kc3BsaXQgPC0gcGFzdGUwKCJDbHVzdGVyXG4iLCBrY2x1c3Q4JGNsdXN0ZXIpCiNzcGxpdCA8LSBmYWN0b3IocGFzdGUwKCJDbHVzdGVyXG4iLCBrY2x1c3QzJGNsdXN0ZXIpLCBsZXZlbHM9YygiQ2x1c3RlclxuMyIsIkNsdXN0ZXJcbjEiLCJDbHVzdGVyXG40IiwiQ2x1c3RlclxuNSIsIkNsdXN0ZXJcbjIiLCJDbHVzdGVyXG42IikpCmhtYXBfayA8LSBIZWF0bWFwKGRhdGFITW0sIHNwbGl0PXNwbGl0LCBjbHVzdGVyX3Jvd19zbGljZXMgPSBGQUxTRSwKICAgICAgICAgICAgICAgICAgY2x1c3Rlcl9jb2x1bW5zID0gRkFMU0UsCiAgICAgICAgICAgICAgICAgIHNob3dfcm93X25hbWVzID0gRkFMU0UsCiAgICAgICAgICAgICAgICAgIG5hbWUgPSAibG9nZmMiLAogICAgICAgICAgICAgICAgICBjb2wgPSBjb2xfZnVuR1IsCiAgICAgICAgICAgICAgICAgIHdpZHRoID0gdW5pdCg1MCwgIm1tIiksCiAgICAgICAgICAgICAgICAgIGNvbHVtbl90aXRsZSA9ICJtZWFucyBsb2dmYyIsIAogICAgICAgICAgICAgICAgICBjb2x1bW5fdGl0bGVfZ3AgPSBncGFyKGZvbnRzaXplID0gMTYsIGZvbnRmYWNlID0gImJvbGQiKSkKCmhtYXBfaytobWFwX2hpZXJfZmFjdG9yczYraG1hcF9oaWVyX2ZhY3RvcnM1CmBgYAoKCk1lYW4gcHJvZmlsZXMgb2YgY2x1c3RlcnMKCmBgYHtyLGZpZy5oZWlnaHQgPSA4LGZpZy53aWR0aCA9IDh9CmNsdXN0ZXJjb3VudDwtZGF0YS5mcmFtZShrY2x1c3Q4JGNsdXN0ZXIpCmNsdXN0ZXJzaXplczwtdGFibGUoY2x1c3RlcmNvdW50JGtjbHVzdDguY2x1c3RlcikKY2x1c3Rlck1lYW5zPC1kYXRhLmZyYW1lKGtjbHVzdDgkY2VudGVycykKY2x1c3Rlck1lYW5zMTwtZGF0YS5mcmFtZSh0KGNsdXN0ZXJNZWFucykpCmNsdXN0ZXJNZWFuczEgPC0gY2JpbmQocm93bmFtZXMoY2x1c3Rlck1lYW5zMSksIGNsdXN0ZXJNZWFuczEpCm9yZGVyTjwtYygiVmFyMTRfUkJDXzBoIiwiVmFyMTRfUkJDXzJoIiwiVmFyMTRfUkJDXzZoIiwiVmFyMTRfUkJDXzIwaCIpIyMjIyBtYW51YWwKCnJvd25hbWVzKGNsdXN0ZXJNZWFuczEpIDwtIE5VTEwKbmFtZXMoY2x1c3Rlck1lYW5zMSlbbmFtZXMoY2x1c3Rlck1lYW5zMSk9PSJyb3duYW1lcyhjbHVzdGVyTWVhbnMxKSJdIDwtICJTYW1wbGUiCiMjIyNjbHVzdGVyTWVhbnMxCgpwWDE8LWdncGxvdChkYXRhPWNsdXN0ZXJNZWFuczEsIGFlcyh4PVNhbXBsZSwgeT1YMSxncm91cD0xKSkgKwogIGdlb21fbGluZSgpKyAgZ2VvbV9wb2ludCgpK2dndGl0bGUocGFzdGUoIkNsdXN0ZXIgWDEgUHJvZmlsZSAiLGNsdXN0ZXJzaXplc1sxXSwiIGdlbmVzIikpKyAgc2NhbGVfeF9kaXNjcmV0ZShsaW1pdHM9b3JkZXJOKSsKICB0aGVtZShheGlzLnRpdGxlLnggPSBlbGVtZW50X2JsYW5rKCksYXhpcy50aXRsZS55ID0gZWxlbWVudF9ibGFuaygpKQpwWDI8LWdncGxvdChkYXRhPWNsdXN0ZXJNZWFuczEsIGFlcyh4PVNhbXBsZSwgeT1YMixncm91cD0xKSkgKwogIGdlb21fbGluZSgpKyAgZ2VvbV9wb2ludCgpK2dndGl0bGUocGFzdGUoIkNsdXN0ZXIgWDIgUHJvZmlsZSAiLGNsdXN0ZXJzaXplc1syXSwiIGdlbmVzIikpKyAgc2NhbGVfeF9kaXNjcmV0ZShsaW1pdHM9b3JkZXJOKSsKICB0aGVtZShheGlzLnRpdGxlLnggPSBlbGVtZW50X2JsYW5rKCksYXhpcy50aXRsZS55ID0gZWxlbWVudF9ibGFuaygpKQpwWDM8LWdncGxvdChkYXRhPWNsdXN0ZXJNZWFuczEsIGFlcyh4PVNhbXBsZSwgeT1YMyxncm91cD0xKSkgKwogIGdlb21fbGluZSgpKyAgZ2VvbV9wb2ludCgpK2dndGl0bGUocGFzdGUoIkNsdXN0ZXIgWDMgUHJvZmlsZSAiLGNsdXN0ZXJzaXplc1szXSwiIGdlbmVzIikpKyAgc2NhbGVfeF9kaXNjcmV0ZShsaW1pdHM9b3JkZXJOKSsKICB0aGVtZShheGlzLnRpdGxlLnggPSBlbGVtZW50X2JsYW5rKCksYXhpcy50aXRsZS55ID0gZWxlbWVudF9ibGFuaygpKQpwWDQ8LWdncGxvdChkYXRhPWNsdXN0ZXJNZWFuczEsIGFlcyh4PVNhbXBsZSwgeT1YNCxncm91cD0xKSkgKwogIGdlb21fbGluZSgpKyAgZ2VvbV9wb2ludCgpK2dndGl0bGUocGFzdGUoIkNsdXN0ZXIgWDQgUHJvZmlsZSAiLGNsdXN0ZXJzaXplc1s0XSwiIGdlbmVzIikpKyAgc2NhbGVfeF9kaXNjcmV0ZShsaW1pdHM9b3JkZXJOKSsKICB0aGVtZShheGlzLnRpdGxlLnggPSBlbGVtZW50X2JsYW5rKCksYXhpcy50aXRsZS55ID0gZWxlbWVudF9ibGFuaygpKQpwWDU8LWdncGxvdChkYXRhPWNsdXN0ZXJNZWFuczEsIGFlcyh4PVNhbXBsZSwgeT1YNSxncm91cD0xKSkgKwogIGdlb21fbGluZSgpKyAgZ2VvbV9wb2ludCgpK2dndGl0bGUocGFzdGUoIkNsdXN0ZXIgWDUgUHJvZmlsZSAiLGNsdXN0ZXJzaXplc1s1XSwiIGdlbmVzIikpKyAgc2NhbGVfeF9kaXNjcmV0ZShsaW1pdHM9b3JkZXJOKSsKICB0aGVtZShheGlzLnRpdGxlLnggPSBlbGVtZW50X2JsYW5rKCksYXhpcy50aXRsZS55ID0gZWxlbWVudF9ibGFuaygpKQpwWDY8LWdncGxvdChkYXRhPWNsdXN0ZXJNZWFuczEsIGFlcyh4PVNhbXBsZSwgeT1YNixncm91cD0xKSkgKwogIGdlb21fbGluZSgpKyAgZ2VvbV9wb2ludCgpK2dndGl0bGUocGFzdGUoIkNsdXN0ZXIgWDYgUHJvZmlsZSAiLGNsdXN0ZXJzaXplc1s2XSwiIGdlbmVzIikpKyAgc2NhbGVfeF9kaXNjcmV0ZShsaW1pdHM9b3JkZXJOKSsKICB0aGVtZShheGlzLnRpdGxlLnggPSBlbGVtZW50X2JsYW5rKCksYXhpcy50aXRsZS55ID0gZWxlbWVudF9ibGFuaygpKQoKCiNwbG90Cm11bHRpcGxvdChwWDEsIHBYMiwgcFgzLCBwWDQscFg1LCBwWDYsIGNvbHM9MikKCgoKYGBgCgoKCmBgYHtyfQp0b3BERWdlbmVzIDwtIHdoaWNoKHRlbXBBJEluY2x1ZGU9PSJpbiIpIyMjI2ZpbmQgaW5kZXhlcwp0ZW1wQWttPC10ZW1wQVsgdG9wREVnZW5lcywgXQpTeW1ib2xzS208LWRwbHlyOjpwdWxsKHRlbXBBa20sIEdlbmVfU3ltYm9sKQojIyMjIGV4cG9ydCB0aGUgZ2VuZSBleHByZXNzaW9uIGRhdGEgZm9yIHRoZSBjbHVzdGVycwp3cml0ZS50YWJsZShjbHVzdGVyTWVhbnMscGFzdGUwKCJDbHVzdGVyTWVhbnNLbV8iLGdyb3Vwc05hbWUsIi50eHQiKSwgIHNlcCA9ICJcdCIpCkNsdXN0ZXJlZEdlbmVzPC1kYXRhLmZyYW1lKGtjbHVzdDgkY2x1c3RlcixTeW1ib2xzS20sZGF0YUhNbSkKd3JpdGUudGFibGUoQ2x1c3RlcmVkR2VuZXMscGFzdGUwKCJTY2FsZWREYXRhSW5DbHVzdGVyc0ttXyIsZ3JvdXBzTmFtZSwiLnR4dCIpLCAgc2VwID0gIlx0IikKI2hlYWQoQ2x1c3RlcmVkR2VuZXMpCmBgYAoKCmBgYHtyfQpib3R0b21ERWdlbmVzPC13aGljaCh0ZW1wQSRJbmNsdWRlPT0ib3V0IikjIyMjZmluZCBpbmRleGVzIApib3R0b21HPC10ZW1wQVsgYm90dG9tREVnZW5lcywgXQpib3R0b21HPC1kcGx5cjo6cHVsbChib3R0b21HLCBHZW5lX1N5bWJvbCkKd3JpdGUudGFibGUoYm90dG9tRyxwYXN0ZTAoImlwYUJvdHRvbUttZWFuc18iLGdyb3Vwc05hbWUsIi50eHQiKSwgIHNlcCA9ICJcdCIpCiAgICAgICAgICAgICAgICAgICAgICAgICAKCnRvcERFZ2VuZXMgPC0gd2hpY2godGVtcEEkSW5jbHVkZT09ImluIikjIyMjZmluZCBpbmRleGVzIAp0ZW1wQWttPC10ZW1wQVsgdG9wREVnZW5lcywgXQpTeW1ib2xzS208LWRwbHlyOjpwdWxsKHRlbXBBa20sIEdlbmVfU3ltYm9sKQoKaXBhS21lYW5zPC1DbHVzdGVyZWRHZW5lcwojY291bnRzVGFibGUgPC1jb3VudHNUYWJsZVssYygxOjE1KV0jIyMjaWYgc2FtcGxlcyBuZWVkIHJlbW92aW5nCmlwYUttZWFuczwtaXBhS21lYW5zWyxjKDE6MildCmlwYUttZWFucyRuYW1lMjwtcm93bmFtZXMoaXBhS21lYW5zKQojaXBhS21lYW5zJT4lIHJvd25hbWVzX3RvX2NvbHVtbih2YXIgPSAicm93bmFtZSIpCiNpcGFLbWVhbnMKI3Jvd2lkX3RvX2NvbHVtbihpcGFLbWVhbnMpCmlwYUttZWFucyA9IG11dGF0ZShpcGFLbWVhbnMsIHgxPSBpZmVsc2UoaXBhS21lYW5zJGtjbHVzdDguY2x1c3Rlcj09MSwgIjEiLCAiMCIpKQppcGFLbWVhbnMgPSBtdXRhdGUoaXBhS21lYW5zLCB4Mj0gaWZlbHNlKGlwYUttZWFucyRrY2x1c3Q4LmNsdXN0ZXI9PTIsICIxIiwgIjAiKSkKaXBhS21lYW5zID0gbXV0YXRlKGlwYUttZWFucywgeDM9IGlmZWxzZShpcGFLbWVhbnMka2NsdXN0OC5jbHVzdGVyPT0zLCAiMSIsICIwIikpCmlwYUttZWFucyA9IG11dGF0ZShpcGFLbWVhbnMsIHg0PSBpZmVsc2UoaXBhS21lYW5zJGtjbHVzdDguY2x1c3Rlcj09NCwgIjEiLCAiMCIpKQppcGFLbWVhbnMgPSBtdXRhdGUoaXBhS21lYW5zLCB4NT0gaWZlbHNlKGlwYUttZWFucyRrY2x1c3Q4LmNsdXN0ZXI9PTUsICIxIiwgIjAiKSkKaXBhS21lYW5zID0gbXV0YXRlKGlwYUttZWFucywgeDY9IGlmZWxzZShpcGFLbWVhbnMka2NsdXN0OC5jbHVzdGVyPT02LCAiMSIsICIwIikpCiNpcGFLbWVhbnMKd3JpdGUudGFibGUoaXBhS21lYW5zLHBhc3RlMCgiaXBhS21lYW5zXyIsZ3JvdXBzTmFtZSwiLnR4dCIpLCAgc2VwID0gIlx0IikKI2hlYWQoaXBhS21lYW5zKQoKYGBgCgoKYGBge3J9CkNsdXN0ZXJlZEdlbmVzMjwtQ2x1c3RlcmVkR2VuZXNbYygxKV0KI0NsdXN0ZXJlZEdlbmVzMgpsaXN0QWxsPC1saXN0KCkKZm9yKGkgaW4gMTo2KSB7CiAgY2x1c3Rlck5hbWU8LXBhc3RlMCgieCIsaSkKICAjY2x1c3Rlck5hbWU8LXJvdy5uYW1lcyhzdWJzZXQoQ2x1c3RlcmVkR2VuZXMsQ2x1c3RlcmVkR2VuZXM9PWkpKQogIGNsdXN0ZXJOYW1lPC0oc3Vic2V0KENsdXN0ZXJlZEdlbmVzJFN5bWJvbHNLbSxDbHVzdGVyZWRHZW5lcz09aSkpCiAgbGlzdEFsbFtbaV1dPC1jbHVzdGVyTmFtZQp9CiNuZWVkIHRvIG5hbWUgdGhlIHZlY3RvcnMgaW4gdGhlIGxpc3QsIGV4YW1wbGUgaGVyZSBpcyBmb3IgOCBjbHVzdGVycwpuYW1lcyhsaXN0QWxsKTwtYygiWDEiLCAiWDIiLCAiWDMiLCAiWDQiLCJYNSIsICJYNiIpCgojaWYgeW91IHdhbnQgdG8gcmVhcnJhbmdlIHRoZSBvcmRlcgojbGlzdEFsbDwtbGlzdEFsbFtjKCJ4MyIsICJ4NyIsICJ4OCIsICJ4MiIsICJ4NiIsICJ4NSIsICJ4NCIsICJ4MSIpXQoKI2xhcHBseShsaXN0QWxsLCBoZWFkKQoKYGBgCgojIyMjNC4gQW5ub3RhdGlvbiBvZiBLLW1lYW5zIGNsdXN0ZXJzCi0gQ0MgY2VsbHVsYXIgY29tcGFydG1lbnQKLSBCUCBiaW9sb2dpY2FsIHByb2Nlc3MKLSBNRiBtb2xlY3VsYXIgZnVuY3Rpb24KClRoZSBzaW1wbGlmeSBmdW5jdGlvbiBoYXMgYmVlbiB1c2VkIHRvIGN1dCBkb3duIG9uIEdPIHJlZHVuZGFuY3kKCmBgYHtyfQojc3RyKEFsbEdlbmVOYW1lcykKYGBgCgoKYGBge3IsZmlnLmhlaWdodCA9IDgsZmlnLndpZHRoID0gMTJ9CiMjIyNDQwpjZ29DQyA8LSBjb21wYXJlQ2x1c3RlcihnZW5lQ2x1c3RlciA9IGxpc3RBbGwsIAogICAgICAgICAgICAgICAgICAgICAgdW5pdmVyc2UgPSBBbGxHZW5lTmFtZXMsCiAgICAgICAgICAgICAgICAgICAgICBmdW4gPSAiZW5yaWNoR08iLAogICAgICAgICAgICAgICAgICAgICAgT3JnRGI9b3JnLkhzLmVnLmRiLCAKICAgICAgICAgICAgICAgICAgICAgICMjIyNPcmdEYj1vcmcuTW0uZWcuZGIsCiAgICAgICAgICAgICAgICAgICAgICBrZXlUeXBlPSJTWU1CT0wiLAogICAgICAgICAgICAgICAgICAgICAgb250ID0gIkNDIiwgCiAgICAgICAgICAgICAgICAgICAgICBwdmFsdWVDdXRvZmY9MC4wNSwKICAgICAgICAgICAgICAgICAgICAgIHF2YWx1ZUN1dG9mZiA9IDAuMTApCmNnb0NDMiA8LSBzaW1wbGlmeShjZ29DQywgY3V0b2ZmPTAuNywgYnk9InAuYWRqdXN0Iiwgc2VsZWN0X2Z1bj1taW4pCiMjIyN3cml0ZSBhcyBzcHJlYWRzaGVldAp3cml0ZS5jc3YoYXMuZGF0YS5mcmFtZShjZ29DQzIpLHBhc3RlMCgiR09fQ0NfIixncm91cHNOYW1lLCIuY3N2IikpCgpkb3RwbG90KGNnb0NDMixzaG93Q2F0ZWdvcnkgPSAzMCwKICAgICAgICB0aXRsZSA9IHBhc3RlMCgiR08gQ2VsbHVsYXIgQ29tcGFydG1lbnQgIixncm91cHNOYW1lKSkrCiAgdGhlbWUoYXhpcy50ZXh0LnggPSBlbGVtZW50X3RleHQoYW5nbGUgPSA5MCwgdmp1c3QgPSAwLjUsIGhqdXN0PTEpKQoKYGBgClBsb3RzIGFuZCBHTyBkYXRhIHdlcmUgd3JpdHRlbiB0byBmaWxlcwpgYGB7cn0KcG5nKHBhc3RlMCgiR09fQ0NfIixncm91cHNOYW1lLCIucG5nIiksIHdpZHRoID0gMTIyNCwgaGVpZ2h0ID0gODI0KQpkb3RwbG90KGNnb0NDMixzaG93Q2F0ZWdvcnkgPSAzMCwKICAgICAgICB0aXRsZSA9IHBhc3RlMCgiR08gQ2VsbHVsYXIgQ29tcGFydG1lbnQgIixncm91cHNOYW1lKSkrCiAgdGhlbWUoYXhpcy50ZXh0LnggPSBlbGVtZW50X3RleHQoYW5nbGUgPSA5MCwgdmp1c3QgPSAwLjUsIGhqdXN0PTEpKQpkZXYub2ZmKCkKYGBgCgpHTyBCUApgYGB7cixmaWcuaGVpZ2h0ID0gMTIsZmlnLndpZHRoID0gMTJ9CiMjIyNDQwpjZ29CUCA8LSBjb21wYXJlQ2x1c3RlcihnZW5lQ2x1c3RlciA9IGxpc3RBbGwsIAogICAgICAgICAgICAgICAgICAgICAgdW5pdmVyc2UgPSBBbGxHZW5lTmFtZXMsCiAgICAgICAgICAgICAgICAgICAgICBmdW4gPSAiZW5yaWNoR08iLAogICAgICAgICAgICAgICAgICAgICAgT3JnRGI9b3JnLkhzLmVnLmRiLAogICAgICAgICAgICAgICAgICAgICAga2V5VHlwZT0iU1lNQk9MIiwKICAgICAgICAgICAgICAgICAgICAgIG9udCA9ICJCUCIsIAogICAgICAgICAgICAgICAgICAgICAgcHZhbHVlQ3V0b2ZmPTAuMDUsCiAgICAgICAgICAgICAgICAgICAgICBxdmFsdWVDdXRvZmYgPSAwLjEwKQpjZ29CUDIgPC0gc2ltcGxpZnkoY2dvQlAsIGN1dG9mZj0wLjcsIGJ5PSJwLmFkanVzdCIsIHNlbGVjdF9mdW49bWluKQojIyMjd3JpdGUgYXMgc3ByZWFkc2hlZXQKd3JpdGUuY3N2KGFzLmRhdGEuZnJhbWUoY2dvQlAyKSxwYXN0ZTAoIkdPX0JQXyIsZ3JvdXBzTmFtZSwiLmNzdiIpKQoKZG90cGxvdChjZ29CUDIsc2hvd0NhdGVnb3J5ID0gMzAsCiAgICAgICAgdGl0bGUgPSBwYXN0ZTAoIkdPIEJpb2xvZ2ljYWwgUHJvY2VzcyAiLGdyb3Vwc05hbWUpKSsKICB0aGVtZShheGlzLnRleHQueCA9IGVsZW1lbnRfdGV4dChhbmdsZSA9IDkwLCB2anVzdCA9IDAuNSwgaGp1c3Q9MSkpCgpgYGAKCmBgYHtyfQpwbmcocGFzdGUwKCJHT19CUF8iLGdyb3Vwc05hbWUsIi5wbmciKSwgd2lkdGggPSAxMDI0LCBoZWlnaHQgPSAxMjI0KQpkb3RwbG90KGNnb0JQMixzaG93Q2F0ZWdvcnkgPSAzMCwKICAgICAgICB0aXRsZSA9IHBhc3RlMCgiR08gQmlvbG9naWNhbCBQcm9jZXNzICIsZ3JvdXBzTmFtZSkpKwogIHRoZW1lKGF4aXMudGV4dC54ID0gZWxlbWVudF90ZXh0KGFuZ2xlID0gOTAsIHZqdXN0ID0gMC41LCBoanVzdD0xKSkKZGV2Lm9mZigpCmBgYAoKCkdPIE1GCmBgYHtyLGZpZy5oZWlnaHQgPSAxMCxmaWcud2lkdGggPSAxMn0KIyMjI01GCmNnb01GIDwtIGNvbXBhcmVDbHVzdGVyKGdlbmVDbHVzdGVyID0gbGlzdEFsbCwgCiAgICAgICAgICAgICAgICAgICAgICB1bml2ZXJzZSA9IEFsbEdlbmVOYW1lcywKICAgICAgICAgICAgICAgICAgICAgIGZ1biA9ICJlbnJpY2hHTyIsCiAgICAgICAgICAgICAgICAgICAgICBPcmdEYj1vcmcuSHMuZWcuZGIsIAogICAgICAgICAgICAgICAgICAgICAga2V5VHlwZT0iU1lNQk9MIiwKICAgICAgICAgICAgICAgICAgICAgIG9udCA9ICJNRiIsIAogICAgICAgICAgICAgICAgICAgICAgcHZhbHVlQ3V0b2ZmPTAuMDUsCiAgICAgICAgICAgICAgICAgICAgICBxdmFsdWVDdXRvZmYgPSAwLjEwKQpjZ29NRjIgPC0gc2ltcGxpZnkoY2dvTUYsIGN1dG9mZj0wLjcsIGJ5PSJwLmFkanVzdCIsIHNlbGVjdF9mdW49bWluKQojIyMjd3JpdGUgYXMgc3ByZWFkc2hlZXQKd3JpdGUuY3N2KGFzLmRhdGEuZnJhbWUoY2dvTUYyKSxwYXN0ZTAoIkdPX01GXyIsZ3JvdXBzTmFtZSwiLmNzdiIpKQoKZG90cGxvdChjZ29NRjIsc2hvd0NhdGVnb3J5ID0gMzAsCiAgICAgICAgdGl0bGUgPSBwYXN0ZTAoIkdPIE1vbGVjdWxhciBGdW5jdGlvbiAgIixncm91cHNOYW1lKSkrCiAgdGhlbWUoYXhpcy50ZXh0LnggPSBlbGVtZW50X3RleHQoYW5nbGUgPSA5MCwgdmp1c3QgPSAwLjUsIGhqdXN0PTEpKQoKYGBgCgoKCmBgYHtyfQpwbmcocGFzdGUwKCJHT19NRl8iLGdyb3Vwc05hbWUsIi5wbmciKSwgd2lkdGggPSAxNDI0LCBoZWlnaHQgPSA4MjQpCmRvdHBsb3QoY2dvTUYyLHNob3dDYXRlZ29yeSA9IDMwLAogICAgICAgIHRpdGxlID0gcGFzdGUwKCJHTyBNb2xlY3VsYXIgRnVuY3Rpb24gICIsZ3JvdXBzTmFtZSkpKwogIHRoZW1lKGF4aXMudGV4dC54ID0gZWxlbWVudF90ZXh0KGFuZ2xlID0gOTAsIHZqdXN0ID0gMC41LCBoanVzdD0xKSkKZGV2Lm9mZigpCmBgYAoKIyMjIFI1IFZBUjE0IHZzIFJCQyBUTkYgay1tZWFucyBwYWRqMC4xZmMyCgojIyMjMS4gR2VuZWxpc3QgU2VsZWN0aW9uCgpgYGB7cn0KZ3JvdXBzTmFtZTwtIlI1X1ZhcjE0dlJCQ19UTkZfa21lYW5zX3BhZGowLjFmYzIiCmBgYAoKCmBgYHtyfQpjb3VudHNUYWJsZTwtcmVhZC5kZWxpbSgiUk5Bc2VxMjAxOUp1bHlfNS50eHQiLCBoZWFkZXIgPSBUUlVFLCBzZXAgPSAiXHQiLGNoZWNrLm5hbWVzPUZBTFNFLHJvdy5uYW1lcz0xKQpoZWFkKGNvdW50c1RhYmxlKQpgYGAKCmBgYHtyfQpBbGxHZW5lTmFtZXM8LWNvdW50c1RhYmxlJEdlbmVfU3ltYm9sCiNoZWFkKEFsbEdlbmVOYW1lcykKYGBgCgpgYGB7cixmaWcuaGVpZ2h0ID0gMTAsZmlnLndpZHRoID0gMjB9CmdyaWQuYXJyYW5nZShnVHJlZShjaGlsZHJlbj12ZW5ucTIpLCBnVHJlZShjaGlsZHJlbj12ZW5ucHEpICwgbmNvbD0yLHRvcD0iUjUgVmFyMTQgVE5GIikKYGBgCgpgYGB7cn0KI3RlbXBBPC1yZXNBbGxbLWMoMTA6MzApIF0KdGVtcEE8LWNvdW50c1RhYmxlCiNyb3duYW1lcyh0ZW1wQSkKcm93bmFtZXModGVtcEEpIDwtIE5VTEwKdGVtcEEgPSBtdXRhdGUodGVtcEEsIEluY2x1ZGU9CiAgICAgICAgICAgICAgICAgICBpZmVsc2UodGVtcEEkcGFkal9SNV9UTkZfdmFyMTRfdnNfUkJDXzBoPDAuMSZhYnModGVtcEEkbG9nMkZvbGRDaGFuZ2VfUjVfVE5GX3ZhcjE0X3ZzX1JCQ18waCk+MSYhaXMubmEodGVtcEEkcGFkal9SNV9UTkZfdmFyMTRfdnNfUkJDXzBoKSwgImluIiwKICAgICAgICAgICAgICAgICAgICAgICAgICBpZmVsc2UodGVtcEEkcGFkal9SNV9UTkZfdmFyMTRfdnNfUkJDXzJoPDAuMSZhYnModGVtcEEkbG9nMkZvbGRDaGFuZ2VfUjVfVE5GX3ZhcjE0X3ZzX1JCQ18yaCk+MSYhaXMubmEodGVtcEEkcGFkal9SNV9UTkZfdmFyMTRfdnNfUkJDXzJoKSwgImluIiwKICAgICAgICAgICAgICAgICAgICAgICAgICAgICAgICAgaWZlbHNlKHRlbXBBJHBhZGpfUjVfVE5GX3ZhcjE0X3ZzX1JCQ182aDwwLjEmYWJzKHRlbXBBJGxvZzJGb2xkQ2hhbmdlX1I1X1RORl92YXIxNF92c19SQkNfNmgpPjEmIWlzLm5hKHRlbXBBJHBhZGpfUjVfVE5GX3ZhcjE0X3ZzX1JCQ182aCksICJpbiIsCiAgICAgICAgICAgICAgICAgICAgICAgICAgICAgICAgICAgICAgICBpZmVsc2UodGVtcEEkcGFkal9SNV9UTkZfdmFyMTRfdnNfUkJDXzIwaDwwLjEmYWJzKHRlbXBBJGxvZzJGb2xkQ2hhbmdlX1I1X1RORl92YXIxNF92c19SQkNfMjBoKT4xJiFpcy5uYSh0ZW1wQSRwYWRqX1I1X1RORl92YXIxNF92c19SQkNfMjBoKSwgImluIiwKICAgICAgICAgICAgICAgICAgICAgICAgICAgICAgICAgICAgICAgICAgICAgICAgICAgICAgICAgICAgICAgICAgICAgICAib3V0IikpKSkpCgoKI3RlbXBBCiMjIyNsaWJyYXJ5KGRwbHlyKQp0ZW1wQSAlPiUKICAgICBncm91cF9ieShJbmNsdWRlKSAlPiUgCiAgICAgdGFsbHkoKQoKYGBgCgpgYGB7cn0KdG9wREVnZW5lcyA8LSB3aGljaCh0ZW1wQSRJbmNsdWRlPT0iaW4iKSMjIyNmaW5kIGluZGV4ZXMgCmBgYAoKCiMjIyMgTkIgUGxlYXNlIGNoZWNrIGNvbHVtbnMgdXNlZCBhbmQgcmVuYW1lZCBmb3IgcGxvdHMKYGBge3J9CiNiYXNlTWVhbnNIbSA8LWNvdW50c1RhYmxlWyxjKDYwOjYzKV0KYmFzZU1lYW5zSG0gPC1jb3VudHNUYWJsZVssYygxMTA6MTEzLDEyOToxMzIpXQpoZWFkKGJhc2VNZWFuc0htKQpiYXNlTWVhbnNIbTIgPC0gbG9nMihiYXNlTWVhbnNIbSsxKQpiYXNlTWVhbnNIbTIkVmFyMTRfUkJDXzBoPC1iYXNlTWVhbnNIbTIkVmFyMTRUTkZfMGhfbWVhbi1iYXNlTWVhbnNIbTIkUkJDX1RORl8waF9tZWFuCmJhc2VNZWFuc0htMiRWYXIxNF9SQkNfMmg8LWJhc2VNZWFuc0htMiRWYXIxNFRORl8yaF9tZWFuLWJhc2VNZWFuc0htMiRSQkNfVE5GXzJoX21lYW4KYmFzZU1lYW5zSG0yJFZhcjE0X1JCQ182aDwtYmFzZU1lYW5zSG0yJFZhcjE0VE5GXzZoX21lYW4tYmFzZU1lYW5zSG0yJFJCQ19UTkZfNmhfbWVhbgpiYXNlTWVhbnNIbTIkVmFyMTRfUkJDXzIwaDwtYmFzZU1lYW5zSG0yJFZhcjE0VE5GXzIwaF9tZWFuLWJhc2VNZWFuc0htMiRSQkNfVE5GXzIwaF9tZWFuCmJhc2VNZWFuc0htIDwtYmFzZU1lYW5zSG0yWyxjKDk6MTIpXQpoZWFkKGJhc2VNZWFuc0htKQpiYXNlTWVhbnNIbU0gPC1iYXNlTWVhbnNIbTJbLGMoMTo4KV0KaGVhZChiYXNlTWVhbnNIbU0pCmBgYAoKCgpgYGB7cn0KdG9wREVnZW5lcyA8LSB3aGljaCh0ZW1wQSRJbmNsdWRlPT0iaW4iKSMjIyNmaW5kIGluZGV4ZXMgCmBgYAoKCiMjIyMyLiBIaWVyYWNoaWNhbCBjbHVzdGVyaW5nIG9mIG1lYW5zIApgYGB7cixmaWcuaGVpZ2h0ID0gMTIsZmlnLndpZHRoID0gMjB9CiMjIyNtZWFuIGxvZ2ZjCmRhdGFITW08LWJhc2VNZWFuc0htWyB0b3BERWdlbmVzLCBdCiNkYXRhSE1tIDwtIGxvZzIoZGF0YUhNbSsxKQpkYXRhSE1tPC0gdChhcy5tYXRyaXgoZGF0YUhNbSkpCmRhdGFITW0gPC0gdChzY2FsZShkYXRhSE1tKSkKCmhtYXBfaGllcl9mYWN0b3JzNCA8LSBIZWF0bWFwKAogIGRhdGFITW0sICBuYW1lID0gImxvZ2ZjIiwKICByb3dfbGFiZWxzID0gcGFzdGUwKHJvd25hbWVzKGRhdGFITW0pLCIgIiwodGVtcEFbIHRvcERFZ2VuZXMsIF0pJEdlbmVfU3ltYm9sKSwKICBjb2x1bW5fdGl0bGUgPSBwYXN0ZTAoIk1lYW5zIGxvZ2ZjIiksIAogIGNvbCA9IGNvbF9mdW5HUiwKICBjb2x1bW5fdGl0bGVfZ3AgPSBncGFyKGZvbnRzaXplID0gMTYsIGZvbnRmYWNlID0gImJvbGQiKSwKICB3aWR0aCA9IHVuaXQoNTAsICJtbSIpLAogIGNsdXN0ZXJfY29sdW1ucyA9IEZBTFNFLAogIHNob3dfcm93X25hbWVzID0gRkFMU0UpCgpkYXRhSE1tUGxvdDwtYXMuZGF0YS5mcmFtZShkYXRhSE1tKQpkYXRhSE1tUGxvdCRWYXIxNF9SQkNfMmg8LWRhdGFITW1QbG90JFZhcjE0X1JCQ18yaC1kYXRhSE1tUGxvdCRWYXIxNF9SQkNfMGgKZGF0YUhNbVBsb3QkVmFyMTRfUkJDXzZoPC1kYXRhSE1tUGxvdCRWYXIxNF9SQkNfNmgtZGF0YUhNbVBsb3QkVmFyMTRfUkJDXzBoCmRhdGFITW1QbG90JFZhcjE0X1JCQ18yMGg8LWRhdGFITW1QbG90JFZhcjE0X1JCQ18yMGgtZGF0YUhNbVBsb3QkVmFyMTRfUkJDXzBoCmRhdGFITW1QbG90JFZhcjE0X1JCQ18waDwtZGF0YUhNbVBsb3QkVmFyMTRfUkJDXzBoLWRhdGFITW1QbG90JFZhcjE0X1JCQ18waAoKZGF0YUhNbVBsb3Q8LWFzLm1hdHJpeChkYXRhSE1tUGxvdCkKICAKaG1hcF9oaWVyX2ZhY3RvcnM2IDwtIEhlYXRtYXAoCiAgZGF0YUhNbVBsb3QsICBuYW1lID0gIk5vcm1hbGlzZWQgbG9nZmMiLAogIHJvd19sYWJlbHMgPSBwYXN0ZTAocm93bmFtZXMoZGF0YUhNbSksIiAiLCh0ZW1wQVsgdG9wREVnZW5lcywgXSkkR2VuZV9TeW1ib2wpLAogIGNvbHVtbl90aXRsZSA9IHBhc3RlMCgiMGggTm9ybWFsaXNlZCBsb2dmYyIpLCAKICBjb2wgPSBjb2xfZnVuR1IyLAogIGNvbHVtbl90aXRsZV9ncCA9IGdwYXIoZm9udHNpemUgPSAxNiwgZm9udGZhY2UgPSAiYm9sZCIpLAogIHdpZHRoID0gdW5pdCg1MCwgIm1tIiksCiAgY2x1c3Rlcl9jb2x1bW5zID0gRkFMU0UsCiAgc2hvd19yb3dfbmFtZXMgPSBGQUxTRSkKCiMjIyNtZWFucwpkYXRhSE1tMzwtYmFzZU1lYW5zSG1NWyB0b3BERWdlbmVzLCBdCiNkYXRhSE1tIDwtIGxvZzIoZGF0YUhNbSsxKQpkYXRhSE1tMzwtIHQoYXMubWF0cml4KGRhdGFITW0zKSkKZGF0YUhNbTMgPC0gdChzY2FsZShkYXRhSE1tMykpCmRhdGFITW0zPC1kYXRhSE1tM1ssYyg1LDEsNiwyLDcsMyw4LDQpXQpobWFwX2hpZXJfZmFjdG9yczUgPC0gSGVhdG1hcCgKICBkYXRhSE1tMywgIG5hbWUgPSAiRXhwcmVzc2lvbiIsCiAgcm93X2xhYmVscyA9IHBhc3RlMChyb3duYW1lcyhkYXRhSE1tKSwiICIsKHRlbXBBWyB0b3BERWdlbmVzLCBdKSRHZW5lX1N5bWJvbCksCiAgY29sdW1uX3RpdGxlID0gcGFzdGUwKCJNZWFucyIpLCAKICBjb2wgPSBjb2xfZnVuLAogIGNvbHVtbl90aXRsZV9ncCA9IGdwYXIoZm9udHNpemUgPSAxNiwgZm9udGZhY2UgPSAiYm9sZCIpLAogIHdpZHRoID0gdW5pdCgxMDAsICJtbSIpLAogIGNsdXN0ZXJfY29sdW1ucyA9IEZBTFNFLAogIHNob3dfcm93X25hbWVzID0gRkFMU0UpCiAgCmhtYXBfaGllcl9mYWN0b3JzNCtobWFwX2hpZXJfZmFjdG9yczYraG1hcF9oaWVyX2ZhY3RvcnM1CmBgYAoKCmBgYHtyLGZpZy5oZWlnaHQgPSA3LGZpZy53aWR0aCA9MTIgfQpwYXIobWZyb3c9YygxLDIpKQojIyMjIFNpbGhvdWV0dGUgbWV0aG9kCmZ2aXpfbmJjbHVzdChkYXRhSE1tLCBrbWVhbnMsIG1ldGhvZCA9ICJzaWxob3VldHRlIixrLm1heCA9IDE2KSsKICBsYWJzKHN1YnRpdGxlID0gIlNpbGhvdWV0dGUgbWV0aG9kIikKCiMjIyMgRWxib3cgbWV0aG9kCmZ2aXpfbmJjbHVzdChkYXRhSE1tLCBrbWVhbnMsIG1ldGhvZCA9ICJ3c3MiLGsubWF4ID0gMTYpICsKICBsYWJzKHN1YnRpdGxlID0gIkVsYm93IG1ldGhvZCIpCgpgYGAKCmBgYHtyfQojIyMjZ2FwIHN0YXQgc2xvdyEhIQojIyMjc2V0LnNlZWQoMTIzKQojIyMjZnZpel9uYmNsdXN0KGRhdGFITW0sIGttZWFucywgbnN0YXJ0ID0gMjUsICBtZXRob2QgPSAiZ2FwX3N0YXQiLCBuYm9vdCA9IDEwMCxrLm1heCA9IDE2KSsKIyMjIyAgbGFicyhzdWJ0aXRsZSA9ICJHYXAgc3RhdGlzdGljIG1ldGhvZCIpCmBgYAoKCmBgYHtyLGZpZy5oZWlnaHQgPSA3fQoja2NsdXN0OGIgPC0ga21lYW5zKGRhdGFITW0sIDYpCiNzaWxob3VldHRlIHBsb3QKZGlzdEs8LWRhaXN5KGRhdGFITW0pCnBsb3Qoc2lsaG91ZXR0ZShrY2x1c3Q4YiRjbHVzdGVyLCBkaXN0SyksIGNvbD0xOjYsIGJvcmRlcj1OQSkKCmBgYAoKCiMjIyMzLiBLLW1lYW5zIGNsdXN0ZXJpbmcgb2YgbWVhbnMgCgpgYGB7cixmaWcuaGVpZ2h0ID0gMTAsZmlnLndpZHRoID0gMjB9CnNwbGl0IDwtIHBhc3RlMCgiQ2x1c3RlclxuIiwga2NsdXN0OGIkY2x1c3RlcikKI3NwbGl0IDwtIGZhY3RvcihwYXN0ZTAoIkNsdXN0ZXJcbiIsIGtjbHVzdDMkY2x1c3RlciksIGxldmVscz1jKCJDbHVzdGVyXG4zIiwiQ2x1c3RlclxuMSIsIkNsdXN0ZXJcbjQiLCJDbHVzdGVyXG41IiwiQ2x1c3RlclxuMiIsIkNsdXN0ZXJcbjYiKSkKaG1hcF9rIDwtIEhlYXRtYXAoZGF0YUhNbSwgc3BsaXQ9c3BsaXQsIGNsdXN0ZXJfcm93X3NsaWNlcyA9IEZBTFNFLAogICAgICAgICAgICAgICAgICBjbHVzdGVyX2NvbHVtbnMgPSBGQUxTRSwKICAgICAgICAgICAgICAgICAgc2hvd19yb3dfbmFtZXMgPSBGQUxTRSwKICAgICAgICAgICAgICAgICAgbmFtZSA9ICJsb2dmYyIsCiAgICAgICAgICAgICAgICAgIGNvbCA9IGNvbF9mdW5HUiwKICAgICAgICAgICAgICAgICAgd2lkdGggPSB1bml0KDUwLCAibW0iKSwKICAgICAgICAgICAgICAgICAgY29sdW1uX3RpdGxlID0gIm1lYW5zIGxvZ2ZjIiwgCiAgICAgICAgICAgICAgICAgIGNvbHVtbl90aXRsZV9ncCA9IGdwYXIoZm9udHNpemUgPSAxNiwgZm9udGZhY2UgPSAiYm9sZCIpKQoKaG1hcF9rK2htYXBfaGllcl9mYWN0b3JzNitobWFwX2hpZXJfZmFjdG9yczUKYGBgCgoKTWVhbiBwcm9maWxlcyBvZiBjbHVzdGVycwoKYGBge3IsZmlnLmhlaWdodCA9IDgsZmlnLndpZHRoID0gOH0KY2x1c3RlcmNvdW50PC1kYXRhLmZyYW1lKGtjbHVzdDhiJGNsdXN0ZXIpCmNsdXN0ZXJzaXplczwtdGFibGUoY2x1c3RlcmNvdW50JGtjbHVzdDhiLmNsdXN0ZXIpCmNsdXN0ZXJNZWFuczwtZGF0YS5mcmFtZShrY2x1c3Q4YiRjZW50ZXJzKQpjbHVzdGVyTWVhbnMxPC1kYXRhLmZyYW1lKHQoY2x1c3Rlck1lYW5zKSkKY2x1c3Rlck1lYW5zMSA8LSBjYmluZChyb3duYW1lcyhjbHVzdGVyTWVhbnMxKSwgY2x1c3Rlck1lYW5zMSkKb3JkZXJOPC1jKCJWYXIxNF9SQkNfMGgiLCJWYXIxNF9SQkNfMmgiLCJWYXIxNF9SQkNfNmgiLCJWYXIxNF9SQkNfMjBoIikjIyMjIG1hbnVhbAoKcm93bmFtZXMoY2x1c3Rlck1lYW5zMSkgPC0gTlVMTApuYW1lcyhjbHVzdGVyTWVhbnMxKVtuYW1lcyhjbHVzdGVyTWVhbnMxKT09InJvd25hbWVzKGNsdXN0ZXJNZWFuczEpIl0gPC0gIlNhbXBsZSIKIyMjI2NsdXN0ZXJNZWFuczEKCnBYMTwtZ2dwbG90KGRhdGE9Y2x1c3Rlck1lYW5zMSwgYWVzKHg9U2FtcGxlLCB5PVgxLGdyb3VwPTEpKSArCiAgZ2VvbV9saW5lKCkrICBnZW9tX3BvaW50KCkrZ2d0aXRsZShwYXN0ZSgiQ2x1c3RlciBYMSBQcm9maWxlICIsY2x1c3RlcnNpemVzWzFdLCIgZ2VuZXMiKSkrICBzY2FsZV94X2Rpc2NyZXRlKGxpbWl0cz1vcmRlck4pKwogIHRoZW1lKGF4aXMudGl0bGUueCA9IGVsZW1lbnRfYmxhbmsoKSxheGlzLnRpdGxlLnkgPSBlbGVtZW50X2JsYW5rKCkpCnBYMjwtZ2dwbG90KGRhdGE9Y2x1c3Rlck1lYW5zMSwgYWVzKHg9U2FtcGxlLCB5PVgyLGdyb3VwPTEpKSArCiAgZ2VvbV9saW5lKCkrICBnZW9tX3BvaW50KCkrZ2d0aXRsZShwYXN0ZSgiQ2x1c3RlciBYMiBQcm9maWxlICIsY2x1c3RlcnNpemVzWzJdLCIgZ2VuZXMiKSkrICBzY2FsZV94X2Rpc2NyZXRlKGxpbWl0cz1vcmRlck4pKwogIHRoZW1lKGF4aXMudGl0bGUueCA9IGVsZW1lbnRfYmxhbmsoKSxheGlzLnRpdGxlLnkgPSBlbGVtZW50X2JsYW5rKCkpCnBYMzwtZ2dwbG90KGRhdGE9Y2x1c3Rlck1lYW5zMSwgYWVzKHg9U2FtcGxlLCB5PVgzLGdyb3VwPTEpKSArCiAgZ2VvbV9saW5lKCkrICBnZW9tX3BvaW50KCkrZ2d0aXRsZShwYXN0ZSgiQ2x1c3RlciBYMyBQcm9maWxlICIsY2x1c3RlcnNpemVzWzNdLCIgZ2VuZXMiKSkrICBzY2FsZV94X2Rpc2NyZXRlKGxpbWl0cz1vcmRlck4pKwogIHRoZW1lKGF4aXMudGl0bGUueCA9IGVsZW1lbnRfYmxhbmsoKSxheGlzLnRpdGxlLnkgPSBlbGVtZW50X2JsYW5rKCkpCnBYNDwtZ2dwbG90KGRhdGE9Y2x1c3Rlck1lYW5zMSwgYWVzKHg9U2FtcGxlLCB5PVg0LGdyb3VwPTEpKSArCiAgZ2VvbV9saW5lKCkrICBnZW9tX3BvaW50KCkrZ2d0aXRsZShwYXN0ZSgiQ2x1c3RlciBYNCBQcm9maWxlICIsY2x1c3RlcnNpemVzWzRdLCIgZ2VuZXMiKSkrICBzY2FsZV94X2Rpc2NyZXRlKGxpbWl0cz1vcmRlck4pKwogIHRoZW1lKGF4aXMudGl0bGUueCA9IGVsZW1lbnRfYmxhbmsoKSxheGlzLnRpdGxlLnkgPSBlbGVtZW50X2JsYW5rKCkpCnBYNTwtZ2dwbG90KGRhdGE9Y2x1c3Rlck1lYW5zMSwgYWVzKHg9U2FtcGxlLCB5PVg1LGdyb3VwPTEpKSArCiAgZ2VvbV9saW5lKCkrICBnZW9tX3BvaW50KCkrZ2d0aXRsZShwYXN0ZSgiQ2x1c3RlciBYNSBQcm9maWxlICIsY2x1c3RlcnNpemVzWzVdLCIgZ2VuZXMiKSkrICBzY2FsZV94X2Rpc2NyZXRlKGxpbWl0cz1vcmRlck4pKwogIHRoZW1lKGF4aXMudGl0bGUueCA9IGVsZW1lbnRfYmxhbmsoKSxheGlzLnRpdGxlLnkgPSBlbGVtZW50X2JsYW5rKCkpCnBYNjwtZ2dwbG90KGRhdGE9Y2x1c3Rlck1lYW5zMSwgYWVzKHg9U2FtcGxlLCB5PVg2LGdyb3VwPTEpKSArCiAgZ2VvbV9saW5lKCkrICBnZW9tX3BvaW50KCkrZ2d0aXRsZShwYXN0ZSgiQ2x1c3RlciBYNiBQcm9maWxlICIsY2x1c3RlcnNpemVzWzZdLCIgZ2VuZXMiKSkrICBzY2FsZV94X2Rpc2NyZXRlKGxpbWl0cz1vcmRlck4pKwogIHRoZW1lKGF4aXMudGl0bGUueCA9IGVsZW1lbnRfYmxhbmsoKSxheGlzLnRpdGxlLnkgPSBlbGVtZW50X2JsYW5rKCkpCgoKI3Bsb3QKbXVsdGlwbG90KHBYMSwgcFgyLCBwWDMsIHBYNCxwWDUsIHBYNiwgY29scz0yKQoKCgpgYGAKCgpgYGB7cn0KdG9wREVnZW5lcyA8LSB3aGljaCh0ZW1wQSRJbmNsdWRlPT0iaW4iKSMjIyNmaW5kIGluZGV4ZXMKdGVtcEFrbTwtdGVtcEFbIHRvcERFZ2VuZXMsIF0KU3ltYm9sc0ttPC1kcGx5cjo6cHVsbCh0ZW1wQWttLCBHZW5lX1N5bWJvbCkKIyMjIyBleHBvcnQgdGhlIGdlbmUgZXhwcmVzc2lvbiBkYXRhIGZvciB0aGUgY2x1c3RlcnMKd3JpdGUudGFibGUoY2x1c3Rlck1lYW5zLHBhc3RlMCgiQ2x1c3Rlck1lYW5zS21fIixncm91cHNOYW1lLCIudHh0IiksICBzZXAgPSAiXHQiKQpDbHVzdGVyZWRHZW5lczwtZGF0YS5mcmFtZShrY2x1c3Q4YiRjbHVzdGVyLFN5bWJvbHNLbSxkYXRhSE1tKQp3cml0ZS50YWJsZShDbHVzdGVyZWRHZW5lcyxwYXN0ZTAoIlNjYWxlZERhdGFJbkNsdXN0ZXJzS21fIixncm91cHNOYW1lLCIudHh0IiksICBzZXAgPSAiXHQiKQojaGVhZChDbHVzdGVyZWRHZW5lcykKYGBgCgoKYGBge3J9CmJvdHRvbURFZ2VuZXM8LXdoaWNoKHRlbXBBJEluY2x1ZGU9PSJvdXQiKSMjIyNmaW5kIGluZGV4ZXMgCmJvdHRvbUc8LXRlbXBBWyBib3R0b21ERWdlbmVzLCBdCmJvdHRvbUc8LWRwbHlyOjpwdWxsKGJvdHRvbUcsIEdlbmVfU3ltYm9sKQp3cml0ZS50YWJsZShib3R0b21HLHBhc3RlMCgiaXBhQm90dG9tS21lYW5zXyIsZ3JvdXBzTmFtZSwiLnR4dCIpLCAgc2VwID0gIlx0IikKICAgICAgICAgICAgICAgICAgICAgICAgIAoKdG9wREVnZW5lcyA8LSB3aGljaCh0ZW1wQSRJbmNsdWRlPT0iaW4iKSMjIyNmaW5kIGluZGV4ZXMgCnRlbXBBa208LXRlbXBBWyB0b3BERWdlbmVzLCBdClN5bWJvbHNLbTwtZHBseXI6OnB1bGwodGVtcEFrbSwgR2VuZV9TeW1ib2wpCgppcGFLbWVhbnM8LUNsdXN0ZXJlZEdlbmVzCiNjb3VudHNUYWJsZSA8LWNvdW50c1RhYmxlWyxjKDE6MTUpXSMjIyNpZiBzYW1wbGVzIG5lZWQgcmVtb3ZpbmcKaXBhS21lYW5zPC1pcGFLbWVhbnNbLGMoMToyKV0KaXBhS21lYW5zJG5hbWUyPC1yb3duYW1lcyhpcGFLbWVhbnMpCiNpcGFLbWVhbnMlPiUgcm93bmFtZXNfdG9fY29sdW1uKHZhciA9ICJyb3duYW1lIikKI2lwYUttZWFucwojcm93aWRfdG9fY29sdW1uKGlwYUttZWFucykKaXBhS21lYW5zID0gbXV0YXRlKGlwYUttZWFucywgeDE9IGlmZWxzZShpcGFLbWVhbnMka2NsdXN0OGIuY2x1c3Rlcj09MSwgIjEiLCAiMCIpKQppcGFLbWVhbnMgPSBtdXRhdGUoaXBhS21lYW5zLCB4Mj0gaWZlbHNlKGlwYUttZWFucyRrY2x1c3Q4Yi5jbHVzdGVyPT0yLCAiMSIsICIwIikpCmlwYUttZWFucyA9IG11dGF0ZShpcGFLbWVhbnMsIHgzPSBpZmVsc2UoaXBhS21lYW5zJGtjbHVzdDhiLmNsdXN0ZXI9PTMsICIxIiwgIjAiKSkKaXBhS21lYW5zID0gbXV0YXRlKGlwYUttZWFucywgeDQ9IGlmZWxzZShpcGFLbWVhbnMka2NsdXN0OGIuY2x1c3Rlcj09NCwgIjEiLCAiMCIpKQppcGFLbWVhbnMgPSBtdXRhdGUoaXBhS21lYW5zLCB4NT0gaWZlbHNlKGlwYUttZWFucyRrY2x1c3Q4Yi5jbHVzdGVyPT01LCAiMSIsICIwIikpCmlwYUttZWFucyA9IG11dGF0ZShpcGFLbWVhbnMsIHg2PSBpZmVsc2UoaXBhS21lYW5zJGtjbHVzdDhiLmNsdXN0ZXI9PTYsICIxIiwgIjAiKSkKI2lwYUttZWFucwp3cml0ZS50YWJsZShpcGFLbWVhbnMscGFzdGUwKCJpcGFLbWVhbnNfIixncm91cHNOYW1lLCIudHh0IiksICBzZXAgPSAiXHQiKQojaGVhZChpcGFLbWVhbnMpCgpgYGAKCgpgYGB7cn0KQ2x1c3RlcmVkR2VuZXMyPC1DbHVzdGVyZWRHZW5lc1tjKDEpXQojQ2x1c3RlcmVkR2VuZXMyCmxpc3RBbGw8LWxpc3QoKQpmb3IoaSBpbiAxOjYpIHsKICBjbHVzdGVyTmFtZTwtcGFzdGUwKCJ4IixpKQogICNjbHVzdGVyTmFtZTwtcm93Lm5hbWVzKHN1YnNldChDbHVzdGVyZWRHZW5lcyxDbHVzdGVyZWRHZW5lcz09aSkpCiAgY2x1c3Rlck5hbWU8LShzdWJzZXQoQ2x1c3RlcmVkR2VuZXMkU3ltYm9sc0ttLENsdXN0ZXJlZEdlbmVzPT1pKSkKICBsaXN0QWxsW1tpXV08LWNsdXN0ZXJOYW1lCn0KI25lZWQgdG8gbmFtZSB0aGUgdmVjdG9ycyBpbiB0aGUgbGlzdCwgZXhhbXBsZSBoZXJlIGlzIGZvciA4IGNsdXN0ZXJzCm5hbWVzKGxpc3RBbGwpPC1jKCJYMSIsICJYMiIsICJYMyIsICJYNCIsIlg1IiwgIlg2IikKCiNpZiB5b3Ugd2FudCB0byByZWFycmFuZ2UgdGhlIG9yZGVyCiNsaXN0QWxsPC1saXN0QWxsW2MoIngzIiwgIng3IiwgIng4IiwgIngyIiwgIng2IiwgIng1IiwgIng0IiwgIngxIildCgojbGFwcGx5KGxpc3RBbGwsIGhlYWQpCgpgYGAKCiMjIyM0LiBBbm5vdGF0aW9uIG9mIEstbWVhbnMgY2x1c3RlcnMKLSBDQyBjZWxsdWxhciBjb21wYXJ0bWVudAotIEJQIGJpb2xvZ2ljYWwgcHJvY2VzcwotIE1GIG1vbGVjdWxhciBmdW5jdGlvbgoKVGhlIHNpbXBsaWZ5IGZ1bmN0aW9uIGhhcyBiZWVuIHVzZWQgdG8gY3V0IGRvd24gb24gR08gcmVkdW5kYW5jeQoKYGBge3J9CiNzdHIoQWxsR2VuZU5hbWVzKQpgYGAKCgpgYGB7cixmaWcuaGVpZ2h0ID0gOCxmaWcud2lkdGggPSAxMn0KIyMjI0NDCmNnb0NDIDwtIGNvbXBhcmVDbHVzdGVyKGdlbmVDbHVzdGVyID0gbGlzdEFsbCwgCiAgICAgICAgICAgICAgICAgICAgICB1bml2ZXJzZSA9IEFsbEdlbmVOYW1lcywKICAgICAgICAgICAgICAgICAgICAgIGZ1biA9ICJlbnJpY2hHTyIsCiAgICAgICAgICAgICAgICAgICAgICBPcmdEYj1vcmcuSHMuZWcuZGIsIAogICAgICAgICAgICAgICAgICAgICAgIyMjI09yZ0RiPW9yZy5NbS5lZy5kYiwKICAgICAgICAgICAgICAgICAgICAgIGtleVR5cGU9IlNZTUJPTCIsCiAgICAgICAgICAgICAgICAgICAgICBvbnQgPSAiQ0MiLCAKICAgICAgICAgICAgICAgICAgICAgIHB2YWx1ZUN1dG9mZj0wLjA1LAogICAgICAgICAgICAgICAgICAgICAgcXZhbHVlQ3V0b2ZmID0gMC4xMCkKY2dvQ0MyIDwtIHNpbXBsaWZ5KGNnb0NDLCBjdXRvZmY9MC43LCBieT0icC5hZGp1c3QiLCBzZWxlY3RfZnVuPW1pbikKIyMjI3dyaXRlIGFzIHNwcmVhZHNoZWV0CndyaXRlLmNzdihhcy5kYXRhLmZyYW1lKGNnb0NDMikscGFzdGUwKCJHT19DQ18iLGdyb3Vwc05hbWUsIi5jc3YiKSkKCmRvdHBsb3QoY2dvQ0MyLHNob3dDYXRlZ29yeSA9IDMwLAogICAgICAgIHRpdGxlID0gcGFzdGUwKCJHTyBDZWxsdWxhciBDb21wYXJ0bWVudCAiLGdyb3Vwc05hbWUpKSsKICB0aGVtZShheGlzLnRleHQueCA9IGVsZW1lbnRfdGV4dChhbmdsZSA9IDkwLCB2anVzdCA9IDAuNSwgaGp1c3Q9MSkpCgpgYGAKUGxvdHMgYW5kIEdPIGRhdGEgd2VyZSB3cml0dGVuIHRvIGZpbGVzCmBgYHtyfQpwbmcocGFzdGUwKCJHT19DQ18iLGdyb3Vwc05hbWUsIi5wbmciKSwgd2lkdGggPSAxMjI0LCBoZWlnaHQgPSA4MjQpCmRvdHBsb3QoY2dvQ0MyLHNob3dDYXRlZ29yeSA9IDMwLAogICAgICAgIHRpdGxlID0gcGFzdGUwKCJHTyBDZWxsdWxhciBDb21wYXJ0bWVudCAiLGdyb3Vwc05hbWUpKSsKICB0aGVtZShheGlzLnRleHQueCA9IGVsZW1lbnRfdGV4dChhbmdsZSA9IDkwLCB2anVzdCA9IDAuNSwgaGp1c3Q9MSkpCmRldi5vZmYoKQpgYGAKCkdPIEJQCmBgYHtyLGZpZy5oZWlnaHQgPSAxMixmaWcud2lkdGggPSAxMn0KIyMjI0NDCmNnb0JQIDwtIGNvbXBhcmVDbHVzdGVyKGdlbmVDbHVzdGVyID0gbGlzdEFsbCwgCiAgICAgICAgICAgICAgICAgICAgICB1bml2ZXJzZSA9IEFsbEdlbmVOYW1lcywKICAgICAgICAgICAgICAgICAgICAgIGZ1biA9ICJlbnJpY2hHTyIsCiAgICAgICAgICAgICAgICAgICAgICBPcmdEYj1vcmcuSHMuZWcuZGIsCiAgICAgICAgICAgICAgICAgICAgICBrZXlUeXBlPSJTWU1CT0wiLAogICAgICAgICAgICAgICAgICAgICAgb250ID0gIkJQIiwgCiAgICAgICAgICAgICAgICAgICAgICBwdmFsdWVDdXRvZmY9MC4wNSwKICAgICAgICAgICAgICAgICAgICAgIHF2YWx1ZUN1dG9mZiA9IDAuMTApCmNnb0JQMiA8LSBzaW1wbGlmeShjZ29CUCwgY3V0b2ZmPTAuNywgYnk9InAuYWRqdXN0Iiwgc2VsZWN0X2Z1bj1taW4pCiMjIyN3cml0ZSBhcyBzcHJlYWRzaGVldAp3cml0ZS5jc3YoYXMuZGF0YS5mcmFtZShjZ29CUDIpLHBhc3RlMCgiR09fQlBfIixncm91cHNOYW1lLCIuY3N2IikpCgpkb3RwbG90KGNnb0JQMixzaG93Q2F0ZWdvcnkgPSAzMCwKICAgICAgICB0aXRsZSA9IHBhc3RlMCgiR08gQmlvbG9naWNhbCBQcm9jZXNzICIsZ3JvdXBzTmFtZSkpKwogIHRoZW1lKGF4aXMudGV4dC54ID0gZWxlbWVudF90ZXh0KGFuZ2xlID0gOTAsIHZqdXN0ID0gMC41LCBoanVzdD0xKSkKCmBgYAoKYGBge3J9CnBuZyhwYXN0ZTAoIkdPX0JQXyIsZ3JvdXBzTmFtZSwiLnBuZyIpLCB3aWR0aCA9IDEwMjQsIGhlaWdodCA9IDEyMjQpCmRvdHBsb3QoY2dvQlAyLHNob3dDYXRlZ29yeSA9IDMwLAogICAgICAgIHRpdGxlID0gcGFzdGUwKCJHTyBCaW9sb2dpY2FsIFByb2Nlc3MgIixncm91cHNOYW1lKSkrCiAgdGhlbWUoYXhpcy50ZXh0LnggPSBlbGVtZW50X3RleHQoYW5nbGUgPSA5MCwgdmp1c3QgPSAwLjUsIGhqdXN0PTEpKQpkZXYub2ZmKCkKYGBgCgoKR08gTUYKYGBge3IsZmlnLmhlaWdodCA9IDEwLGZpZy53aWR0aCA9IDEyfQojIyMjTUYKY2dvTUYgPC0gY29tcGFyZUNsdXN0ZXIoZ2VuZUNsdXN0ZXIgPSBsaXN0QWxsLCAKICAgICAgICAgICAgICAgICAgICAgIHVuaXZlcnNlID0gQWxsR2VuZU5hbWVzLAogICAgICAgICAgICAgICAgICAgICAgZnVuID0gImVucmljaEdPIiwKICAgICAgICAgICAgICAgICAgICAgIE9yZ0RiPW9yZy5Icy5lZy5kYiwgCiAgICAgICAgICAgICAgICAgICAgICBrZXlUeXBlPSJTWU1CT0wiLAogICAgICAgICAgICAgICAgICAgICAgb250ID0gIk1GIiwgCiAgICAgICAgICAgICAgICAgICAgICBwdmFsdWVDdXRvZmY9MC4wNSwKICAgICAgICAgICAgICAgICAgICAgIHF2YWx1ZUN1dG9mZiA9IDAuMTApCmNnb01GMiA8LSBzaW1wbGlmeShjZ29NRiwgY3V0b2ZmPTAuNywgYnk9InAuYWRqdXN0Iiwgc2VsZWN0X2Z1bj1taW4pCiMjIyN3cml0ZSBhcyBzcHJlYWRzaGVldAp3cml0ZS5jc3YoYXMuZGF0YS5mcmFtZShjZ29NRjIpLHBhc3RlMCgiR09fTUZfIixncm91cHNOYW1lLCIuY3N2IikpCgpkb3RwbG90KGNnb01GMixzaG93Q2F0ZWdvcnkgPSAzMCwKICAgICAgICB0aXRsZSA9IHBhc3RlMCgiR08gTW9sZWN1bGFyIEZ1bmN0aW9uICAiLGdyb3Vwc05hbWUpKSsKICB0aGVtZShheGlzLnRleHQueCA9IGVsZW1lbnRfdGV4dChhbmdsZSA9IDkwLCB2anVzdCA9IDAuNSwgaGp1c3Q9MSkpCgpgYGAKCgoKYGBge3J9CnBuZyhwYXN0ZTAoIkdPX01GXyIsZ3JvdXBzTmFtZSwiLnBuZyIpLCB3aWR0aCA9IDE0MjQsIGhlaWdodCA9IDgyNCkKZG90cGxvdChjZ29NRjIsc2hvd0NhdGVnb3J5ID0gMzAsCiAgICAgICAgdGl0bGUgPSBwYXN0ZTAoIkdPIE1vbGVjdWxhciBGdW5jdGlvbiAgIixncm91cHNOYW1lKSkrCiAgdGhlbWUoYXhpcy50ZXh0LnggPSBlbGVtZW50X3RleHQoYW5nbGUgPSA5MCwgdmp1c3QgPSAwLjUsIGhqdXN0PTEpKQpkZXYub2ZmKCkKYGBgCgoKCgoKCnNhdmU6IG9uY2UgaGFwcHkgd2l0aCBjbHVzdGVyaW5nIHNhdmUgd29ya3NwYWNlIHNvIHRoYXQgaXQgY2FuIGJlIHJlY2FsbGVkCmBgYHtyfQpzYXZlLmltYWdlKGZpbGU9IkttRGVjZW1iZXIuUkRhdGEiKQpgYGAKCgoKCkFkZCBhIG5ldyBjaHVuayBieSBjbGlja2luZyB0aGUgKkluc2VydCBDaHVuayogYnV0dG9uIG9uIHRoZSB0b29sYmFyIG9yIGJ5IHByZXNzaW5nICpDdHJsK0FsdCtJKi4KCldoZW4geW91IHNhdmUgdGhlIG5vdGVib29rLCBhbiBIVE1MIGZpbGUgY29udGFpbmluZyB0aGUgY29kZSBhbmQgb3V0cHV0IHdpbGwgYmUgc2F2ZWQgYWxvbmdzaWRlIGl0IChjbGljayB0aGUgKlByZXZpZXcqIGJ1dHRvbiBvciBwcmVzcyAqQ3RybCtTaGlmdCtLKiB0byBwcmV2aWV3IHRoZSBIVE1MIGZpbGUpLgoKVGhlIHByZXZpZXcgc2hvd3MgeW91IGEgcmVuZGVyZWQgSFRNTCBjb3B5IG9mIHRoZSBjb250ZW50cyBvZiB0aGUgZWRpdG9yLiBDb25zZXF1ZW50bHksIHVubGlrZSAqS25pdCosICpQcmV2aWV3KiBkb2VzIG5vdCBydW4gYW55IFIgY29kZSBjaHVua3MuIEluc3RlYWQsIHRoZSBvdXRwdXQgb2YgdGhlIGNodW5rIHdoZW4gaXQgd2FzIGxhc3QgcnVuIGluIHRoZSBlZGl0b3IgaXMgZGlzcGxheWVkLgo=
