# Supplementary material for: Different PfEMP1-expressing Plasmodium falciparum variants induce divergent endothelial transcriptional responses during co-culture
Source: PLoS One. 2023 Nov 30;18(11):e0295053. doi: 10.1371/journal.pone.0295053 (PMC10688957; doi:10.1371/journal.pone.0295053)
Supplement: S8 File — (HTML) [file pone.0295053.s017.html]

KCouper/Liverpool K-means RNAseq Analysis November 2020


Code 

- Show All Code
- Hide All Code
- Download Rmd

# KCouper/Liverpool K-means RNAseq Analysis November 2020

#### Leo Zeef

## Analysis Sections

Viewing is better if Code is hidden (Top Right drop down list)


```
sink(file="RsessionInfoDESeq2.txt")
library('DESeq2')
library("ggplot2")
library(reshape2)
####library(tidyverse)
####library(splitstackshape)
####library(data.table)
library("RColorBrewer")
library("gplots")
####library('ggdendro')
library('ggrepel')
library("dplyr")
library("ComplexHeatmap")
library("clusterProfiler")
library(VennDiagram) ######
library(UpSetR)
library(gridExtra)
library(cluster)
library(circlize)
library(factoextra)
library(NbClust)
library("biomaRt")
library("org.Hs.eg.db")####human
library("org.Mm.eg.db")####mouse
library(venn)
library(enrichR)
library(ReactomePA)
```


```
Error in library(ReactomePA) : there is no package called ‘ReactomePA’
```


```
col_fun = colorRamp2(c(-1,-0.2, 0,0.2, 1), c("blue","cyan", "grey90","orange", "red"))#heatmap colours
col_funGR = colorRamp2(c(-1.5, 0, 1.5), c("green", "black", "red"))
col_funGR2 = colorRamp2(c(-2, 0, 2), c("green", "black", "red"))
colorsV3 <- c("cornflowerblue",  "brown1","orange2")#Venn colours
colorsV2 <- c("mediumorchid1",  "chartreuse3")#Venn colours
colorsV4<-c("cornflowerblue", "orange2", "green3","red")#Venn colours
colorsV5<-c("cornflowerblue", "orange2", "green3","purple","red")#Venn colours
#col_fun(seq(-3, 3))
```


### DE Gene Selection

#### 1. Genelist Selection


```
groupsName<-"R1_R4_kmeans_q0.05"
```


```
countsTable<-read.delim("RNAseq2019July_5.txt", header = TRUE, sep = "\t",check.names=FALSE,row.names=1)
head(countsTable)
```


```
AllGeneNames<-countsTable$Gene_Symbol
#head(AllGeneNames)
```


```
tempA<-countsTable
```


```
topDEgenes <- which(tempA$padj_R1_Var37_Hours_6h_vs_0h<0.05&((tempA$Var37TNF_0h_mean>10)|(tempA$Var37TNF_6h_mean>10))&!is.na(tempA$padj_R1_Var37_Hours_6h_vs_0h))####find indexes 
listA<-tempA[ topDEgenes, ]$Gene_Symbol
topDEgenes <- which(tempA$padj_R1_Var37_Hours_20h_vs_0h<0.05&((tempA$Var37TNF_0h_mean>10)|(tempA$Var37TNF_20h_mean>10))&!is.na(tempA$padj_R1_Var37_Hours_20h_vs_0h))####find indexes 
listB<-tempA[ topDEgenes, ]$Gene_Symbol
topDEgenes <- which(tempA$padj_R1_Var37_Hours_20h_vs_6h<0.05&((tempA$Var37TNF_20h_mean>10)|(tempA$Var37TNF_6h_mean>10))&!is.na(tempA$padj_R1_Var37_Hours_20h_vs_6h))####find indexes 
listC<-tempA[ topDEgenes, ]$Gene_Symbol
vennq<-venn.diagram(x = list(listA,listB,listC),#,listD) ,
            category.names = c("Var37_6hv0h","Var37_20hv0h","Var37_20hv6h"),
            main="padj<0.05",
            filename = NULL,  scaled = FALSE, fill = colorsV3, cat.col = colorsV3, cat.cex = 1, cat.dist=0.1,  margin = 0.3)
topDEgenes <- which(tempA$pvalue_R1_Var37_Hours_6h_vs_0h<0.05&abs(tempA$log2FoldChange_R1_Var37_Hours_6h_vs_0h)>1&((tempA$Var37TNF_6h_mean>10)|(tempA$Var37TNF_0h_mean>10))&!is.na(tempA$pvalue_R1_Var37_Hours_6h_vs_0h))####find indexes 
listA<-tempA[ topDEgenes, ]$Gene_Symbol
topDEgenes <- which(tempA$pvalue_R1_Var37_Hours_20h_vs_0h<0.05&abs(tempA$log2FoldChange_R1_Var37_Hours_20h_vs_0h)>1&((tempA$Var37TNF_0h_mean>10)|(tempA$Var37TNF_20h_mean>10))&!is.na(tempA$pvalue_R1_Var37_Hours_20h_vs_0h))####find indexes 
listB<-tempA[ topDEgenes, ]$Gene_Symbol
topDEgenes <- which(tempA$pvalue_R1_Var37_Hours_20h_vs_6h<0.05&abs(tempA$log2FoldChange_R1_Var37_Hours_20h_vs_6h)>1&((tempA$Var37TNF_6h_mean>10)|(tempA$Var37TNF_20h_mean>10))&!is.na(tempA$pvalue_R1_Var37_Hours_20h_vs_6h))####find indexes 
listC<-tempA[ topDEgenes, ]$Gene_Symbol
vennp<-venn.diagram(x = list(listA,listB,listC) ,
            category.names = c("Var37_6hv0h","Var37_20hv0h","Var37_20hv6h"),
            main="pvalue<0.05&fold change>2",
            filename = NULL,  scaled = FALSE, fill = colorsV3, cat.col = colorsV3, cat.cex = 1, cat.dist=0.1,  margin = 0.3)
topDEgenes <- which(tempA$padj_R4Var14TNF_Hours_6h_vs_0h<0.05&((tempA$Var14TNF_0h_mean>10)|(tempA$Var14TNF_6h_mean>10))&!is.na(tempA$padj_R4Var14TNF_Hours_6h_vs_0h))####find indexes 
listA<-tempA[ topDEgenes, ]$Gene_Symbol
topDEgenes <- which(tempA$padj_R4Var14TNF_Hours_20h_vs_0h<0.05&((tempA$Var14TNF_20h_mean>10)|(tempA$Var14TNF_0h_mean>10))&!is.na(tempA$padj_R4Var14TNF_Hours_20h_vs_0h))####find indexes 
listB<-tempA[ topDEgenes, ]$Gene_Symbol
topDEgenes <- which(tempA$padj_R1_Var37_Hours_20h_vs_6h<0.05&((tempA$Var14TNF_20h_mean>10)|(tempA$Var14TNF_6h_mean>10))&!is.na(tempA$padj_R1_Var37_Hours_20h_vs_6h))####find indexes 
listC<-tempA[ topDEgenes, ]$Gene_Symbol
vennq2<-venn.diagram(x = list(listA,listB,listC),#,listD) ,
            category.names = c("Var14_6hv0h","Var14_20hv0h","Var14_20hv6h"),
            main="padj<0.05",
            filename = NULL,  scaled = FALSE, fill = colorsV3, cat.col = colorsV3, cat.cex = 1, cat.dist=0.1,  margin = 0.3)
topDEgenes <- which(tempA$pvalue_R4Var14TNF_Hours_6h_vs_0h<0.05&abs(tempA$log2FoldChange_R4Var14TNF_Hours_6h_vs_0h)>1&((tempA$Var14TNF_0h_mean>10)|(tempA$Var14TNF_6h_mean>10))&!is.na(tempA$pvalue_R4Var14TNF_Hours_6h_vs_0h))####find indexes 
listA<-tempA[ topDEgenes, ]$Gene_Symbol
topDEgenes <- which(tempA$pvalue_R4Var14TNF_Hours_20h_vs_0h<0.05&abs(tempA$log2FoldChange_R4Var14TNF_Hours_20h_vs_0h)>1&((tempA$Var14TNF_20h_mean>10)|(tempA$Var14TNF_0h_mean>10))&!is.na(tempA$pvalue_R4Var14TNF_Hours_20h_vs_0h))####find indexes 
listB<-tempA[ topDEgenes, ]$Gene_Symbol
topDEgenes <- which(tempA$pvalue_R4Var14TNF_Hours_20h_vs_6h<0.05&abs(tempA$log2FoldChange_R4Var14TNF_Hours_20h_vs_6h)>1&((tempA$Var14TNF_20h_mean>10)|(tempA$Var14TNF_6h_mean>10))&!is.na(tempA$pvalue_R4Var14TNF_Hours_20h_vs_6h))####find indexes 
listC<-tempA[ topDEgenes, ]$Gene_Symbol
vennp2<-venn.diagram(x = list(listA,listB,listC) ,
            category.names = c("Var14_6hv0h","Var14_20hv0h","Var14_20hv6h"),
            main="pvalue<0.05&fold change>2",
            filename = NULL,  scaled = FALSE, fill = colorsV3, cat.col = colorsV3, cat.cex = 1, cat.dist=0.1,  margin = 0.3)
```


```
topDEgenes <- which((tempA$padj_R1_Var37_Hours_6h_vs_0h<0.05&((tempA$Var37TNF_6h_mean>10)|(tempA$Var37TNF_0h_mean>10))&!is.na(tempA$padj_R1_Var37_Hours_6h_vs_0h))| 
(tempA$padj_R1_Var37_Hours_20h_vs_0h<0.05&((tempA$Var37TNF_0h_mean>10)|(tempA$Var37TNF_20h_mean>10))&!is.na(tempA$padj_R1_Var37_Hours_20h_vs_0h))|
(tempA$padj_R1_Var37_Hours_20h_vs_6h<0.05&((tempA$Var37TNF_6h_mean>10)|(tempA$Var37TNF_20h_mean>10))&!is.na(tempA$padj_R1_Var37_Hours_20h_vs_6h))
)
listA<-tempA[ topDEgenes, ]$Gene_Symbol
topDEgenes <- which((tempA$pvalue_R1_Var37_Hours_6h_vs_0h<0.05&abs(tempA$log2FoldChange_R1_Var37_Hours_6h_vs_0h)>1&((tempA$Var37TNF_6h_mean>10)|(tempA$Var37TNF_0h_mean>10))&!is.na(tempA$pvalue_R1_Var37_Hours_6h_vs_0h))| 
(tempA$pvalue_R1_Var37_Hours_20h_vs_0h<0.05&abs(tempA$log2FoldChange_R1_Var37_Hours_20h_vs_0h)>1&((tempA$Var37TNF_0h_mean>10)|(tempA$Var37TNF_20h_mean>10))&!is.na(tempA$pvalue_R1_Var37_Hours_20h_vs_0h))| 
(tempA$pvalue_R1_Var37_Hours_20h_vs_6h<0.05&abs(tempA$log2FoldChange_R1_Var37_Hours_20h_vs_6h)>1&((tempA$Var37TNF_6h_mean>10)|(tempA$Var37TNF_20h_mean>10))&!is.na(tempA$pvalue_R1_Var37_Hours_20h_vs_6h)) 
 )####find indexes 
listB<-tempA[ topDEgenes, ]$Gene_Symbol
topDEgenes <- which((tempA$padj_R4Var14TNF_Hours_6h_vs_0h<0.05&((tempA$Var14TNF_0h_mean>10)|(tempA$Var14TNF_6h_mean>10))&!is.na(tempA$padj_R4Var14TNF_Hours_6h_vs_0h))| 
(tempA$padj_R4Var14TNF_Hours_20h_vs_0h<0.05&((tempA$Var14TNF_20h_mean>10)|(tempA$Var14TNF_0h_mean>10))&!is.na(tempA$padj_R4Var14TNF_Hours_20h_vs_0h))|
(tempA$padj_R4Var14TNF_Hours_20h_vs_6h<0.05&((tempA$Var14TNF_20h_mean>10)|(tempA$Var14TNF_6h_mean>10))&!is.na(tempA$padj_R4Var14TNF_Hours_20h_vs_6h))
)
listA2<-tempA[ topDEgenes, ]$Gene_Symbol
topDEgenes <- which((tempA$pvalue_R4Var14TNF_Hours_6h_vs_0h<0.05&abs(tempA$log2FoldChange_R4Var14TNF_Hours_6h_vs_0h)>1&((tempA$Var14TNF_0h_mean>10)|(tempA$Var14TNF_6h_mean>10))&!is.na(tempA$pvalue_R4Var14TNF_Hours_6h_vs_0h))| 
(tempA$pvalue_R4Var14TNF_Hours_20h_vs_0h<0.05&abs(tempA$log2FoldChange_R4Var14TNF_Hours_20h_vs_0h)>1&((tempA$Var14TNF_20h_mean>10)|(tempA$Var14TNF_0h_mean>10))&!is.na(tempA$pvalue_R4Var14TNF_Hours_20h_vs_0h))| 
(tempA$pvalue_R4Var14TNF_Hours_20h_vs_6h<0.05&abs(tempA$log2FoldChange_R4Var14TNF_Hours_20h_vs_6h)>1&((tempA$Var14TNF_20h_mean>10)|(tempA$Var14TNF_6h_mean>10))&!is.na(tempA$pvalue_R4Var14TNF_Hours_20h_vs_6h))
 )####find indexes 
listB2<-tempA[ topDEgenes, ]$Gene_Symbol
vennpq<-venn.diagram(x = list(listA,listB,listA2,listB2) ,
            category.names = c("V37padj<0.05","V37p<0.05&fc>2","V14padj<0.05","V14p<0.05&fc>2"),
            main="padj compared to pvalue",
            filename = NULL,  scaled = FALSE, fill = colorsV4, cat.col = colorsV4, cat.cex = 1, cat.dist=0.3,  margin = 0.15)
```


```
grid.arrange(gTree(children=vennq), gTree(children=vennp), ncol=2,top="R1 Var37 TNF")
```


```
grid.arrange(gTree(children=vennq2), gTree(children=vennp2), ncol=2,top="R4 Var14 TNF")
```


```
grid.arrange(gTree(children=vennpq), ncol=1,top="R4 Var14 TNF")
```

### R1 & R4 VAR37 VAR14 TNF k-means q0.05

Design


```
#tempA<-resAll[-c(10:30) ]
tempA<-countsTable
#rownames(tempA)
rownames(tempA) <- NULL
tempA = mutate(tempA, Include=
                   ifelse(tempA$padj_R1_Var37_Hours_6h_vs_0h<0.05&((tempA$Var37TNF_6h_mean>10)|(tempA$Var37TNF_0h_mean>10))&!is.na(tempA$padj_R1_Var37_Hours_6h_vs_0h), "in",
                          ifelse(tempA$padj_R1_Var37_Hours_20h_vs_0h<0.05&((tempA$Var37TNF_0h_mean>10)|(tempA$Var37TNF_20h_mean>10))&!is.na(tempA$padj_R1_Var37_Hours_20h_vs_0h), "in",
                                 ifelse(tempA$padj_R1_Var37_Hours_20h_vs_6h<0.05&((tempA$Var37TNF_6h_mean>10)|(tempA$Var37TNF_20h_mean>10))&!is.na(tempA$padj_R1_Var37_Hours_20h_vs_6h), "in",
                                        ifelse(tempA$padj_R4Var14TNF_Hours_6h_vs_0h<0.05&((tempA$Var14TNF_0h_mean>10)|(tempA$Var14TNF_6h_mean>10))&!is.na(tempA$padj_R4Var14TNF_Hours_6h_vs_0h), "in",
                                               ifelse(tempA$padj_R4Var14TNF_Hours_20h_vs_0h<0.05&((tempA$Var14TNF_20h_mean>10)|(tempA$Var14TNF_0h_mean>10))&!is.na(tempA$padj_R4Var14TNF_Hours_20h_vs_0h),"in",
                                                      ifelse(tempA$padj_R4Var14TNF_Hours_20h_vs_6h<0.05&((tempA$Var14TNF_20h_mean>10)|(tempA$Var14TNF_6h_mean>10))&!is.na(tempA$padj_R4Var14TNF_Hours_20h_vs_6h),"in",
                                                 "out")))))))
#tempA
####library(dplyr)
tempA %>%
     group_by(Include) %>% 
     tally()
```


```
topDEgenes <- which(tempA$Include=="in")####find indexes
```


```
head(countsTable)
```


```
baseMeansHm <-countsTable[,c(48:50,110,112,113)]
head(baseMeansHm)
```


#### NB Please check columns used and renamed for plots


```
#baseMeansHm <-countsTable[,c(60:63)]
baseMeansHm <-countsTable[,c(48:50,110,112,113)]
head(baseMeansHm)
```


```
tail(baseMeansHm[ topDEgenes, ])
```


```
baseMeansHm$Var37TNF_0h<-baseMeansHm$Var37TNF_0h_mean
baseMeansHm$Var37TNF_6h<-baseMeansHm$Var37TNF_6h_mean
baseMeansHm$Var37TNF_20h<-baseMeansHm$Var37TNF_20h_mean
baseMeansHm$Var14TNF_0h<-baseMeansHm$Var14TNF_0h_mean
baseMeansHm$Var14TNF_6h<-baseMeansHm$Var14TNF_6h_mean
baseMeansHm$Var14TNF_20h<-baseMeansHm$Var14TNF_20h_mean
baseMeansHm <-baseMeansHm[,c(7:12)]
#replace low values with 0
baseMeansHm$Var37TNF_0h[baseMeansHm$Var37TNF_0h<10]<-0
baseMeansHm$Var37TNF_6h[baseMeansHm$Var37TNF_6h<10]<-0
baseMeansHm$Var37TNF_20h[baseMeansHm$Var37TNF_20h<10]<-0
baseMeansHm$Var14TNF_0h[baseMeansHm$Var14TNF_0h<10]<-0
baseMeansHm$Var14TNF_6h[baseMeansHm$Var14TNF_6h<10]<-0
baseMeansHm$Var14TNF_20h[baseMeansHm$Var14TNF_20h<10]<-0
tail(baseMeansHm)
```


```
baseMeansHm <- log2(baseMeansHm+1)
tail(baseMeansHm)
```


```
#baseMeansHmM <-baseMeansHm2[,c(1:8)]
#head(baseMeansHmM)
```


```
topDEgenes <- which(tempA$Include=="in")####find indexes
```


```
#scale Var35 and Var14 separately
var14mn<-baseMeansHm[,c(4:6)]
var14mn<- t(as.matrix(var14mn))
var14mn <- t(scale(var14mn))
#head(var14mn)
baseMeansHm2<-baseMeansHm[,c(1:3)]
baseMeansHm2<- t(as.matrix(baseMeansHm2))
baseMeansHm2 <- t(scale(baseMeansHm2))
baseMeansHm2 <- as.data.frame(cbind(baseMeansHm2, var14mn))
baseMeansHm2[is.na(baseMeansHm2)] <- 0
#head(baseMeansHm2)
baseMeansHm2$Var37TNF_0h_lfc<-baseMeansHm2$Var37TNF_0h-baseMeansHm2$Var37TNF_0h
baseMeansHm2$Var37TNF_6h_lfc<-baseMeansHm2$Var37TNF_6h-baseMeansHm2$Var37TNF_0h
baseMeansHm2$Var37TNF_20h_lfc<-baseMeansHm2$Var37TNF_20h-baseMeansHm2$Var37TNF_0h
baseMeansHm2$Var14TNF_0h_lfc<-baseMeansHm2$Var14TNF_0h-baseMeansHm2$Var14TNF_0h
baseMeansHm2$Var14TNF_6h_lfc<-baseMeansHm2$Var14TNF_6h-baseMeansHm2$Var14TNF_0h
baseMeansHm2$Var14TNF_20h_lfc<-baseMeansHm2$Var14TNF_20h-baseMeansHm2$Var14TNF_0h
#baseMeansHm1<-baseMeansHm2[,c(1:6)]
baseMeansHm3<-baseMeansHm2[,c(7:12)]
head(baseMeansHm3)
```


```
baseMeansHm2<-baseMeansHm2[,c(1:6)]
head(baseMeansHm2)
```


```
dataHMm2<-as.matrix(baseMeansHm2[ topDEgenes, ])
tail(dataHMm2)
```


```
                   Var37TNF_0h Var37TNF_6h Var37TNF_20h Var14TNF_0h Var14TNF_6h Var14TNF_20h
ENSG00000120805.13  -0.5782822  -0.5764178     1.154700  -1.0855261   0.2018455    0.8836806
ENSG00000184307.14   0.5771031   0.5775974    -1.154701   0.1224345   0.9331455   -1.0555800
ENSG00000109920.12  -0.5767641  -0.5779363     1.154700  -1.0632535   0.1416024    0.9216511
ENSG00000088280.18  -0.5815036  -0.5731870     1.154691   0.8191921  -1.1143607    0.2951686
ENSG00000007171.16   0.0000000   0.0000000     0.000000  -0.5773503  -0.5773503    1.1547005
ENSG00000132185.16   0.0000000   0.0000000     0.000000  -0.5773503   1.1547005   -0.5773503
```

#### 2. Hierachical clustering of means (individual samples added for inspection)


```
####mean
dataHMm2<-as.matrix(baseMeansHm2[ topDEgenes, ])
dataHMm2_37<-dataHMm2[,c(1,2,3)]
dataHMm2_14<-dataHMm2[,c(4,5,6)]
hmap_hier_factors37 <- Heatmap(
  dataHMm2_37,  name = "mean37",
  row_labels = paste0(rownames(dataHMm2_37)," ",(tempA[ topDEgenes, ])$Gene_Symbol),
  column_title = paste0("MeansV37"), 
  col = col_funGR,
  column_title_gp = gpar(fontsize = 14, fontface = "bold"),
  width = unit(25, "mm"),
  cluster_columns = FALSE,
  #cluster_rows = FALSE,
  show_row_names = FALSE)
hmap_hier_factors37b <- Heatmap(
  dataHMm2_37,  name = "mean37b",
  row_labels = paste0(rownames(dataHMm2_37)," ",(tempA[ topDEgenes, ])$Gene_Symbol),
  column_title = paste0("MeansV37"), 
  col = col_funGR,
  column_title_gp = gpar(fontsize = 14, fontface = "bold"),
  width = unit(25, "mm"),
  #cluster_columns = FALSE,
  #cluster_rows = FALSE,
  show_row_names = FALSE)
hmap_hier_factors14 <- Heatmap(
  dataHMm2_14,  name = "mean14",
  row_labels = paste0(rownames(dataHMm2_14)," ",(tempA[ topDEgenes, ])$Gene_Symbol),
  column_title = paste0("MeansV14"), 
  col = col_funGR,
  column_title_gp = gpar(fontsize = 14, fontface = "bold"),
  width = unit(25, "mm"),
  cluster_columns = FALSE,
  #cluster_rows = FALSE,
  show_row_names = FALSE)
hmap_hier_factors14b <- Heatmap(
  dataHMm2_14,  name = "mean14b",
  row_labels = paste0(rownames(dataHMm2_14)," ",(tempA[ topDEgenes, ])$Gene_Symbol),
  column_title = paste0("MeansV14"), 
  col = col_funGR,
  column_title_gp = gpar(fontsize = 14, fontface = "bold"),
  width = unit(25, "mm"),
  #cluster_columns = FALSE,
  #cluster_rows = FALSE,
  show_row_names = FALSE)
#write.table(dataHMm2,"dataHMm2.txt",  sep = "\t")
hmap_hier_factors4 <- Heatmap(
  dataHMm2,  name = "mean1",
  row_labels = paste0(rownames(dataHMm2)," ",(tempA[ topDEgenes, ])$Gene_Symbol),
  column_title = paste0("Means"), 
  col = col_funGR,
  column_title_gp = gpar(fontsize = 14, fontface = "bold"),
  width = unit(50, "mm"),
  cluster_columns = FALSE,
  #cluster_rows = FALSE,
  show_row_names = FALSE)
dataHMm2b<-dataHMm2[,c(1,4,2,5,3,6)]
hmap_hier_factors4a <- Heatmap(
  dataHMm2b,  name = "mean2",
  row_labels = paste0(rownames(dataHMm2b)," ",(tempA[ topDEgenes, ])$Gene_Symbol),
  column_title = paste0("Means Rearranged"), 
  col = col_funGR,
  column_title_gp = gpar(fontsize = 14, fontface = "bold"),
  width = unit(50, "mm"),
  cluster_columns = FALSE,
  #cluster_rows = FALSE,
  show_row_names = FALSE)
hmap_hier_factors4b <- Heatmap(
  dataHMm2,  name = "mean3",
  row_labels = paste0(rownames(dataHMm2)," ",(tempA[ topDEgenes, ])$Gene_Symbol),
  column_title = paste0("Means Clustered"), 
  col = col_funGR,
  column_title_gp = gpar(fontsize = 14, fontface = "bold"),
  width = unit(50, "mm"),
  #cluster_columns = FALSE,
  #cluster_rows = FALSE,
  show_row_names = FALSE)
dataHMm3<-as.matrix(baseMeansHm3[ topDEgenes, ])
write.table(dataHMm3,"dataHMm3.txt",  sep = "\t")
#baseMeansHm2<-as.matrix(baseMeansHm2)
  
hmap_hier_factors6 <- Heatmap(
  dataHMm3,  name = "logfc1",
  row_labels = paste0(rownames(dataHMm3)," ",(tempA[ topDEgenes, ])$Gene_Symbol),
  column_title = paste0("vs 0h"), 
  col = col_funGR2,
  column_title_gp = gpar(fontsize = 14, fontface = "bold"),
  width = unit(50, "mm"),
  cluster_columns = FALSE,
  show_row_names = FALSE)
dataHMm3b<-dataHMm3[,c(1,4,2,5,3,6)]
hmap_hier_factors6b <- Heatmap(
  dataHMm3b,  name = "logfc2",
  row_labels = paste0(rownames(dataHMm3b)," ",(tempA[ topDEgenes, ])$Gene_Symbol),
  column_title = paste0("vs 0h Rearranged"), 
  col = col_funGR2,
  column_title_gp = gpar(fontsize = 14, fontface = "bold"),
  width = unit(50, "mm"),
  cluster_columns = FALSE,
  show_row_names = FALSE)
hmap_hier_factors6c <- Heatmap(
  dataHMm3,  name = "logfc3",
  row_labels = paste0(rownames(dataHMm3)," ",(tempA[ topDEgenes, ])$Gene_Symbol),
  column_title = paste0("vs 0h"), 
  col = col_funGR2,
  column_title_gp = gpar(fontsize = 14, fontface = "bold"),
  width = unit(50, "mm"),
  #cluster_columns = FALSE,
  show_row_names = FALSE)
hmlist1=hmap_hier_factors37+hmap_hier_factors14+hmap_hier_factors37b+hmap_hier_factors14b
draw(hmlist1, column_title = "Heatmaps on Means (scaled per strain). Genelists combined from VAR37 and VAR14 timecourses padj<0.05", column_title_gp = gpar(fontsize = 22))
```


```
hmlist2=hmap_hier_factors4+hmap_hier_factors4a+hmap_hier_factors4b
draw(hmlist2, column_title = "Heatmaps on Means (scaled per strain)", column_title_gp = gpar(fontsize = 22))
```


```
hmlist3=hmap_hier_factors4+hmap_hier_factors4a+hmap_hier_factors4b+hmap_hier_factors6+hmap_hier_factors6b+hmap_hier_factors6c
draw(hmlist3, column_title = "Heatmaps on Means (scaled per strain) and logfc Means vs Strain 0h", column_title_gp = gpar(fontsize = 22))
```


```
par(mfrow=c(1,2))
#### Silhouette method
fviz_nbclust(dataHMm3, kmeans, method = "silhouette",k.max = 16)+
  labs(subtitle = "Silhouette method")
```


```
#### Elbow method
fviz_nbclust(dataHMm3, kmeans, method = "wss",k.max = 16) +
  labs(subtitle = "Elbow method")
```


```
####gap stat slow!!!
####set.seed(123)
####fviz_nbclust(dataHMm, kmeans, nstart = 25,  method = "gap_stat", nboot = 100,k.max = 16)+
####  labs(subtitle = "Gap statistic method")
```


```
#kclust7 <- kmeans(dataHMm3, 4)
#silhouette plot
distK<-daisy(dataHMm3)
plot(silhouette(kclust7$cluster, distK), col=1:4, border=NA)
```

#### 3. K-means clustering of means


```
split <- paste0("Cluster\n", kclust7$cluster)
#split <- factor(paste0("Cluster\n", kclust3$cluster), levels=c("Cluster\n3","Cluster\n1","Cluster\n4","Cluster\n5","Cluster\n2","Cluster\n6"))
hmap_k <- Heatmap(dataHMm3, split=split, cluster_row_slices = FALSE,
                  cluster_columns = FALSE,
                  show_row_names = FALSE,
                  name = "Means (scaled per strain",
                  col = col_funGR2,
                  width = unit(50, "mm"),
                  column_title = "Means", 
                  column_title_gp = gpar(fontsize = 16, fontface = "bold"))
hmap_k#+hmap_hier_factors6+hmap_hier_factors5
```


Mean profiles of clusters


```
clustercount<-data.frame(kclust7$cluster)
clustersizes<-table(clustercount$kclust7.cluster)
clusterMeans<-data.frame(kclust7$centers)
clusterMeans1<-data.frame(t(clusterMeans))
clusterMeans1 <- cbind(rownames(clusterMeans1), clusterMeans1)
orderN<-c("Var37TNF_0h_lfc","Var37TNF_6h_lfc","Var37TNF_20h_lfc","Var14TNF_0h_lfc","Var14TNF_6h_lfc","Var14TNF_20h_lfc")#### manual
rownames(clusterMeans1) <- NULL
names(clusterMeans1)[names(clusterMeans1)=="rownames(clusterMeans1)"] <- "Sample"
####clusterMeans1
Strain<-factor(c(rep("VAR37",3),rep("VAR14",3)))####note names
#p1=ggplot(data=dataHmt, aes(x=row.names(dataHmt), y=ENSG00000162551.14),group=Run) + ggtitle("ALPL") +geom_point() +  scale_x_discrete(limits=limitsPlot)+  ylab(ylabPlot)+xlab(xlabPlot)+geom_line(aes(group = Run)) 
pX1<-ggplot(data=clusterMeans1, aes(x=Sample, y=X1,group=1)) +
  geom_line(aes(group = Strain))+  geom_point()+ggtitle(paste("Cluster X1 Profile ",clustersizes[1]," genes"))+  scale_x_discrete(limits=orderN)+
  theme(axis.title.x = element_blank(),axis.text.x = element_text(angle = 90, vjust = 0.5, hjust=1),axis.title.y = element_blank())+ylim (-1.8,1.8)
pX2<-ggplot(data=clusterMeans1, aes(x=Sample, y=X2,group=1)) +
  geom_line(aes(group = Strain))+  geom_point()+ggtitle(paste("Cluster X2 Profile ",clustersizes[2]," genes"))+  scale_x_discrete(limits=orderN)+
  theme(axis.title.x = element_blank(),axis.text.x = element_text(angle = 90, vjust = 0.5, hjust=1),axis.title.y = element_blank())+ylim (-1.8,1.8)
pX3<-ggplot(data=clusterMeans1, aes(x=Sample, y=X3,group=1)) +
  geom_line(aes(group = Strain))+  geom_point()+ggtitle(paste("Cluster X3 Profile ",clustersizes[3]," genes"))+  scale_x_discrete(limits=orderN)+
  theme(axis.title.x = element_blank(),axis.text.x = element_text(angle = 90, vjust = 0.5, hjust=1),axis.title.y = element_blank())+ylim (-1.8,1.8)
pX4<-ggplot(data=clusterMeans1, aes(x=Sample, y=X4,group=1)) +
  geom_line(aes(group = Strain))+  geom_point()+ggtitle(paste("Cluster X4 Profile ",clustersizes[4]," genes"))+  scale_x_discrete(limits=orderN)+
  theme(axis.title.x = element_blank(),axis.text.x = element_text(angle = 90, vjust = 0.5, hjust=1),axis.title.y = element_blank())+ylim (-1.8,1.8)
#pX5<-ggplot(data=clusterMeans1, aes(x=Sample, y=X5,group=1)) +
#  geom_line(aes(group = Strain))+  geom_point()+ggtitle(paste("Cluster X5 Profile ",clustersizes[5]," genes"))+  scale_x_discrete(limits=orderN)+
#  theme(axis.title.x = element_blank(),axis.text.x = element_text(angle = 90, vjust = 0.5, hjust=1),axis.title.y = element_blank())+ylim (-1.8,1.8)
#pX8<-ggplot(data=clusterMeans1, aes(x=Sample, y=X8,group=1)) +
#  geom_line()+  geom_point()+ggtitle(paste("Cluster X8 Profile ",clustersizes[6]," genes"))+  scale_x_discrete(limits=orderN)+
#  theme(axis.title.x = element_blank(),axis.title.y = element_blank())
#plot
multiplot(pX1,pX2,pX3,pX4,     cols=2)
```


```
topDEgenes <- which(tempA$Include=="in")####find indexes
tempAkm<-tempA[ topDEgenes, ]
SymbolsKm<-dplyr::pull(tempAkm, Gene_Symbol)
#### export the gene expression data for the clusters
write.table(clusterMeans,paste0("ClusterMeansKm_",groupsName,".txt"),  sep = "\t")
ClusteredGenes<-data.frame(kclust7$cluster,SymbolsKm,dataHMm3)
write.table(ClusteredGenes,paste0("ScaledDataInClustersKm_",groupsName,".txt"),  sep = "\t")
#head(ClusteredGenes)
```


```
bottomDEgenes<-which(tempA$Include=="out")####find indexes 
bottomG<-tempA[ bottomDEgenes, ]
bottomG<-dplyr::pull(bottomG, Gene_Symbol)
write.table(bottomG,paste0("ipaBottomKmeans_",groupsName,".txt"),  sep = "\t")
                         
topDEgenes <- which(tempA$Include=="in")####find indexes 
tempAkm<-tempA[ topDEgenes, ]
SymbolsKm<-dplyr::pull(tempAkm, Gene_Symbol)
ipaKmeans<-ClusteredGenes
#countsTable <-countsTable[,c(1:15)]####if samples need removing
ipaKmeans<-ipaKmeans[,c(1:2)]
ipaKmeans$name2<-rownames(ipaKmeans)
#ipaKmeans%>% rownames_to_column(var = "rowname")
#ipaKmeans
#rowid_to_column(ipaKmeans)
ipaKmeans = mutate(ipaKmeans, x1= ifelse(ipaKmeans$kclust7.cluster==1, "1", "0"))
ipaKmeans = mutate(ipaKmeans, x2= ifelse(ipaKmeans$kclust7.cluster==2, "1", "0"))
ipaKmeans = mutate(ipaKmeans, x3= ifelse(ipaKmeans$kclust7.cluster==3, "1", "0"))
ipaKmeans = mutate(ipaKmeans, x4= ifelse(ipaKmeans$kclust7.cluster==4, "1", "0"))
#ipaKmeans = mutate(ipaKmeans, x5= ifelse(ipaKmeans$kclust3.cluster==5, "1", "0"))
#ipaKmeans = mutate(ipaKmeans, x6= ifelse(ipaKmeans$kclust3.cluster==6, "1", "0"))
#ipaKmeans = mutate(ipaKmeans, x7= ifelse(ipaKmeans$kclust3.cluster==7, "1", "0"))
#ipaKmeans
write.table(ipaKmeans,paste0("ipaKmeans_",groupsName,".txt"),  sep = "\t")
#head(ipaKmeans)
```


```
ClusteredGenes2<-ClusteredGenes[c(1)]
#ClusteredGenes2
listAll<-list()
for(i in 1:4) {
  clusterName<-paste0("x",i)
  #clusterName<-row.names(subset(ClusteredGenes,ClusteredGenes==i))
  clusterName<-(subset(ClusteredGenes$SymbolsKm,ClusteredGenes==i))
  listAll[[i]]<-clusterName
}
#need to name the vectors in the list, example here is for 8 clusters
names(listAll)<-c("X1", "X2", "X3", "X4")#,"X5", "X6", "X7")
#if you want to rearrange the order
#listAll<-listAll[c("x3", "x7", "x8", "x2", "x6", "x5", "x4", "x1")]
lapply(listAll, head)
```


```
$X1
[1] "SOD2"    "RIPK2"   "CCL20"   "SLC12A2" "SELE"    "NFKB1"  

$X2
[1] "CYP1A1" "PLXNA4" "NPTX1"  "EMCN"   "PALMD"  "CTGF"  

$X3
[1] "CSF2"    "SLC41A1" "GPRC5B"  "CXCL3"   "SDC4"    "PLAU"   

$X4
[1] "FRY"     "ELMOD1"  "SLC1A1"  "SLC7A8"  "GALNT15" "MS4A6A"
```


```
(subset(ClusteredGenes$SymbolsKm,ClusteredGenes==1))
```

#### Erichr


```
setEnrichrSite("Enrichr") # Human genes
```


```
Connection changed to https://maayanlab.cloud/Enrichr/
Connection is Live!
```


```
websiteLive <- TRUE
dbs <- listEnrichrDbs()
if (is.null(dbs)) websiteLive <- FALSE
if (websiteLive) head(dbs)
```

#### 4. Annotation of K-means clusters

- CC cellular compartment
- BP biological process
- MF molecular function

The simplify function has been used to cut down on GO redundancy


```
#str(AllGeneNames)
```


```
####CC
cgoCC <- compareCluster(geneCluster = listAll, 
                      universe = AllGeneNames,
                      fun = "enrichGO",
                      OrgDb=org.Hs.eg.db, 
                      ####OrgDb=org.Mm.eg.db,
                      keyType="SYMBOL",
                      ont = "CC", 
                      pvalueCutoff=0.05,
                      qvalueCutoff = 0.10)
cgoCC2 <- simplify(cgoCC, cutoff=0.7, by="p.adjust", select_fun=min)
####write as spreadsheet
write.csv(as.data.frame(cgoCC2),paste0("GO_CC_",groupsName,".csv"))
dotplot(cgoCC2,showCategory = 30,
        title = paste0("GO Cellular Compartment ",groupsName))+
  theme(axis.text.x = element_text(angle = 90, vjust = 0.5, hjust=1))
```


Plots and GO data were written to files


```
png(paste0("GO_CC_",groupsName,".png"), width = 1224, height = 824)
dotplot(cgoCC2,showCategory = 30,
        title = paste0("GO Cellular Compartment ",groupsName))+
  theme(axis.text.x = element_text(angle = 90, vjust = 0.5, hjust=1))
dev.off()
```


```
null device 
          1
```


GO BP


```
####CC
cgoBP <- compareCluster(geneCluster = listAll, 
                      universe = AllGeneNames,
                      fun = "enrichGO",
                      OrgDb=org.Hs.eg.db,
                      keyType="SYMBOL",
                      ont = "BP", 
                      pvalueCutoff=0.05,
                      qvalueCutoff = 0.10)
cgoBP2 <- simplify(cgoBP, cutoff=0.7, by="p.adjust", select_fun=min)
####write as spreadsheet
write.csv(as.data.frame(cgoBP2),paste0("GO_BP_",groupsName,".csv"))
dotplot(cgoBP2,showCategory = 30,
        title = paste0("GO Biological Process ",groupsName))+
  theme(axis.text.x = element_text(angle = 90, vjust = 0.5, hjust=1))
```


```
png(paste0("GO_BP_",groupsName,".png"), width = 1024, height = 1224)
dotplot(cgoBP2,showCategory = 30,
        title = paste0("GO Biological Process ",groupsName))+
  theme(axis.text.x = element_text(angle = 90, vjust = 0.5, hjust=1))
dev.off()
```


```
null device 
          1
```


GO MF


```
####MF
cgoMF <- compareCluster(geneCluster = listAll, 
                      universe = AllGeneNames,
                      fun = "enrichGO",
                      OrgDb=org.Hs.eg.db, 
                      keyType="SYMBOL",
                      ont = "MF", 
                      pvalueCutoff=0.05,
                      qvalueCutoff = 0.10)
cgoMF2 <- simplify(cgoMF, cutoff=0.7, by="p.adjust", select_fun=min)
####write as spreadsheet
write.csv(as.data.frame(cgoMF2),paste0("GO_MF_",groupsName,".csv"))
dotplot(cgoMF2,showCategory = 30,
        title = paste0("GO Molecular Function  ",groupsName))+
  theme(axis.text.x = element_text(angle = 90, vjust = 0.5, hjust=1))
```


```
#dbs <- c("GO_Molecular_Function_2018", "GO_Cellular_Component_2018", "GO_Biological_Process_2018")
dbs <- c("Reactome_2016","WikiPathways_2019_Mouse")
if (websiteLive) {    enriched1 <- enrichr((subset(ClusteredGenes$SymbolsKm,ClusteredGenes==1)), dbs)}
if (websiteLive) plotEnrich(enriched1[[1]], showTerms = 30, numChar = 80, y = "Count", orderBy = "P.value", title ="Reactome Enrichment Analysis Cluster 1")
```


```
if (websiteLive) {    enriched2 <- enrichr((subset(ClusteredGenes$SymbolsKm,ClusteredGenes==2)), dbs)}
if (websiteLive) plotEnrich(enriched2[[1]], showTerms = 30, numChar = 80, y = "Count", orderBy = "P.value", title ="Reactome Enrichment Analysis Cluster 2")
```


```
if (websiteLive) {    enriched3 <- enrichr((subset(ClusteredGenes$SymbolsKm,ClusteredGenes==3)), dbs)}
if (websiteLive) plotEnrich(enriched3[[1]], showTerms = 30, numChar = 80, y = "Count", orderBy = "P.value", title ="Reactome Enrichment Analysis Cluster 3")
```


```
if (websiteLive) {    enriched4 <- enrichr((subset(ClusteredGenes$SymbolsKm,ClusteredGenes==4)), dbs)}
if (websiteLive) plotEnrich(enriched4[[1]], showTerms = 30, numChar = 80, y = "Count", orderBy = "P.value", title ="Reactome Enrichment Analysis Cluster 4")
```


```
if (websiteLive) plotEnrich(enriched1[[2]], showTerms = 30, numChar = 80, y = "Count", orderBy = "P.value", title ="WikiPathways Enrichment Analysis Cluster 1")
```


```
if (websiteLive) plotEnrich(enriched2[[2]], showTerms = 30, numChar = 80, y = "Count", orderBy = "P.value", title ="WikiPathways Enrichment Analysis Cluster 2")
```


```
if (websiteLive) plotEnrich(enriched3[[2]], showTerms = 30, numChar = 80, y = "Count", orderBy = "P.value", title ="WikiPathways Enrichment Analysis Cluster 3")
```


```
if (websiteLive) plotEnrich(enriched4[[2]], showTerms = 30, numChar = 80, y = "Count", orderBy = "P.value", title ="WikiPathways Enrichment Analysis Cluster 4")
```


```
png(paste0("GO_MF_",groupsName,".png"), width = 1424, height = 824)
dotplot(cgoMF2,showCategory = 30,
        title = paste0("GO Molecular Function  ",groupsName))+
  theme(axis.text.x = element_text(angle = 90, vjust = 0.5, hjust=1))
dev.off()
```


```
null device 
          1
```

### R1 & R4 VAR37 VAR14 TNF k-means p0.05 lfc 1

Design

#### 1. Genelist Selection


```
groupsName<-"R1_R4_kmeans_p0.05lfc1"
```


```
countsTable<-read.delim("RNAseq2019July_5.txt", header = TRUE, sep = "\t",check.names=FALSE,row.names=1)
head(countsTable)
```


```
AllGeneNames<-countsTable$Gene_Symbol
#head(AllGeneNames)
```


```
tempA<-countsTable
```


```
#tempA<-resAll[-c(10:30) ]
tempA<-countsTable
#rownames(tempA)
rownames(tempA) <- NULL
tempA = mutate(tempA, Include=
                   ifelse(tempA$pvalue_R1_Var37_Hours_6h_vs_0h<0.05&abs(tempA$log2FoldChange_R1_Var37_Hours_6h_vs_0h)>1&((tempA$Var37TNF_0h_mean>10)|(tempA$Var37TNF_6h_mean>10))&!is.na(tempA$pvalue_R1_Var37_Hours_6h_vs_0h), "in",
                          ifelse(tempA$pvalue_R1_Var37_Hours_20h_vs_0h<0.05&abs(tempA$log2FoldChange_R1_Var37_Hours_20h_vs_0h)>1&((tempA$Var37TNF_0h_mean>10)|(tempA$Var37TNF_20h_mean>10))&!is.na(tempA$pvalue_R1_Var37_Hours_20h_vs_0h), "in",
                                 ifelse(tempA$pvalue_R1_Var37_Hours_20h_vs_6h<0.05&abs(tempA$log2FoldChange_R1_Var37_Hours_20h_vs_6h)>1&((tempA$Var37TNF_6h_mean>10)|(tempA$Var37TNF_20h_mean>10))&!is.na(tempA$pvalue_R1_Var37_Hours_20h_vs_6h), "in",
                                        ifelse(tempA$pvalue_R4Var14TNF_Hours_6h_vs_0h<0.05&abs(tempA$log2FoldChange_R4Var14TNF_Hours_6h_vs_0h)>1&((tempA$Var14TNF_0h_mean>10)|(tempA$Var14TNF_6h_mean>10))&!is.na(tempA$pvalue_R4Var14TNF_Hours_6h_vs_0h), "in",
                                               ifelse(tempA$pvalue_R4Var14TNF_Hours_20h_vs_0h<0.05&abs(tempA$log2FoldChange_R4Var14TNF_Hours_20h_vs_0h)>1&((tempA$Var14TNF_0h_mean>10)|(tempA$Var14TNF_20h_mean>10))&!is.na(tempA$pvalue_R4Var14TNF_Hours_20h_vs_0h),"in",
                                                      ifelse(tempA$pvalue_R4Var14TNF_Hours_20h_vs_6h<0.05&abs(tempA$log2FoldChange_R4Var14TNF_Hours_20h_vs_6h)>1&((tempA$Var14TNF_20h_mean>10)|(tempA$Var14TNF_6h_mean>10))&!is.na(tempA$pvalue_R4Var14TNF_Hours_20h_vs_6h),"in",
                                                 "out")))))))
#tempA
####library(dplyr)
tempA %>%
     group_by(Include) %>% 
     tally()
```


```
topDEgenes <- which(tempA$Include=="in")####find indexes
```


```
head(countsTable)
```


```
baseMeansHm <-countsTable[,c(48:50,110,112,113)]
head(baseMeansHm)
```

#### NB Please check columns used and renamed for plots


```
#baseMeansHm <-countsTable[,c(60:63)]
baseMeansHm <-countsTable[,c(48:50,110,112,113)]
head(baseMeansHm)
```


```
tail(baseMeansHm[ topDEgenes, ])
```


```
baseMeansHm$Var37TNF_0h<-baseMeansHm$Var37TNF_0h_mean
baseMeansHm$Var37TNF_6h<-baseMeansHm$Var37TNF_6h_mean
baseMeansHm$Var37TNF_20h<-baseMeansHm$Var37TNF_20h_mean
baseMeansHm$Var14TNF_0h<-baseMeansHm$Var14TNF_0h_mean
baseMeansHm$Var14TNF_6h<-baseMeansHm$Var14TNF_6h_mean
baseMeansHm$Var14TNF_20h<-baseMeansHm$Var14TNF_20h_mean
baseMeansHm <-baseMeansHm[,c(7:12)]
#replace low values with 0
baseMeansHm$Var37TNF_0h[baseMeansHm$Var37TNF_0h<10]<-0
baseMeansHm$Var37TNF_6h[baseMeansHm$Var37TNF_6h<10]<-0
baseMeansHm$Var37TNF_20h[baseMeansHm$Var37TNF_20h<10]<-0
baseMeansHm$Var14TNF_0h[baseMeansHm$Var14TNF_0h<10]<-0
baseMeansHm$Var14TNF_6h[baseMeansHm$Var14TNF_6h<10]<-0
baseMeansHm$Var14TNF_20h[baseMeansHm$Var14TNF_20h<10]<-0
tail(baseMeansHm)
```


```
baseMeansHm <- log2(baseMeansHm+1)
tail(baseMeansHm)
```


```
#baseMeansHmM <-baseMeansHm2[,c(1:8)]
#head(baseMeansHmM)
```


```
topDEgenes <- which(tempA$Include=="in")####find indexes
```


```
#scale Var35 and Var14 separately
var14mn<-baseMeansHm[,c(4:6)]
var14mn<- t(as.matrix(var14mn))
var14mn <- t(scale(var14mn))
#head(var14mn)
baseMeansHm2<-baseMeansHm[,c(1:3)]
baseMeansHm2<- t(as.matrix(baseMeansHm2))
baseMeansHm2 <- t(scale(baseMeansHm2))
baseMeansHm2 <- as.data.frame(cbind(baseMeansHm2, var14mn))
baseMeansHm2[is.na(baseMeansHm2)] <- 0
#head(baseMeansHm2)
baseMeansHm2$Var37TNF_0h_lfc<-baseMeansHm2$Var37TNF_0h-baseMeansHm2$Var37TNF_0h
baseMeansHm2$Var37TNF_6h_lfc<-baseMeansHm2$Var37TNF_6h-baseMeansHm2$Var37TNF_0h
baseMeansHm2$Var37TNF_20h_lfc<-baseMeansHm2$Var37TNF_20h-baseMeansHm2$Var37TNF_0h
baseMeansHm2$Var14TNF_0h_lfc<-baseMeansHm2$Var14TNF_0h-baseMeansHm2$Var14TNF_0h
baseMeansHm2$Var14TNF_6h_lfc<-baseMeansHm2$Var14TNF_6h-baseMeansHm2$Var14TNF_0h
baseMeansHm2$Var14TNF_20h_lfc<-baseMeansHm2$Var14TNF_20h-baseMeansHm2$Var14TNF_0h
#baseMeansHm1<-baseMeansHm2[,c(1:6)]
baseMeansHm3<-baseMeansHm2[,c(7:12)]
head(baseMeansHm3)
```


```
baseMeansHm2<-baseMeansHm2[,c(1:6)]
head(baseMeansHm2)
```


```
dataHMm2<-as.matrix(baseMeansHm2[ topDEgenes, ])
tail(dataHMm2)
```

#### 2. Hierachical clustering of means (individual samples added for inspection)


```
####mean
dataHMm2<-as.matrix(baseMeansHm2[ topDEgenes, ])
dataHMm2_37<-dataHMm2[,c(1,2,3)]
dataHMm2_14<-dataHMm2[,c(4,5,6)]
hmap_hier_factors37 <- Heatmap(
  dataHMm2_37,  name = "mean37",
  row_labels = paste0(rownames(dataHMm2_37)," ",(tempA[ topDEgenes, ])$Gene_Symbol),
  column_title = paste0("MeansV37"), 
  col = col_funGR,
  column_title_gp = gpar(fontsize = 14, fontface = "bold"),
  width = unit(25, "mm"),
  cluster_columns = FALSE,
  #cluster_rows = FALSE,
  show_row_names = FALSE)
hmap_hier_factors37b <- Heatmap(
  dataHMm2_37,  name = "mean37b",
  row_labels = paste0(rownames(dataHMm2_37)," ",(tempA[ topDEgenes, ])$Gene_Symbol),
  column_title = paste0("MeansV37"), 
  col = col_funGR,
  column_title_gp = gpar(fontsize = 14, fontface = "bold"),
  width = unit(25, "mm"),
  #cluster_columns = FALSE,
  #cluster_rows = FALSE,
  show_row_names = FALSE)
hmap_hier_factors14 <- Heatmap(
  dataHMm2_14,  name = "mean14",
  row_labels = paste0(rownames(dataHMm2_14)," ",(tempA[ topDEgenes, ])$Gene_Symbol),
  column_title = paste0("MeansV14"), 
  col = col_funGR,
  column_title_gp = gpar(fontsize = 14, fontface = "bold"),
  width = unit(25, "mm"),
  cluster_columns = FALSE,
  #cluster_rows = FALSE,
  show_row_names = FALSE)
hmap_hier_factors14b <- Heatmap(
  dataHMm2_14,  name = "mean14b",
  row_labels = paste0(rownames(dataHMm2_14)," ",(tempA[ topDEgenes, ])$Gene_Symbol),
  column_title = paste0("MeansV14"), 
  col = col_funGR,
  column_title_gp = gpar(fontsize = 14, fontface = "bold"),
  width = unit(25, "mm"),
  #cluster_columns = FALSE,
  #cluster_rows = FALSE,
  show_row_names = FALSE)
#write.table(dataHMm2,"dataHMm2.txt",  sep = "\t")
hmap_hier_factors4 <- Heatmap(
  dataHMm2,  name = "mean1",
  row_labels = paste0(rownames(dataHMm2)," ",(tempA[ topDEgenes, ])$Gene_Symbol),
  column_title = paste0("Means"), 
  col = col_funGR,
  column_title_gp = gpar(fontsize = 14, fontface = "bold"),
  width = unit(50, "mm"),
  cluster_columns = FALSE,
  #cluster_rows = FALSE,
  show_row_names = FALSE)
dataHMm2b<-dataHMm2[,c(1,4,2,5,3,6)]
hmap_hier_factors4a <- Heatmap(
  dataHMm2b,  name = "mean2",
  row_labels = paste0(rownames(dataHMm2b)," ",(tempA[ topDEgenes, ])$Gene_Symbol),
  column_title = paste0("Means Rearranged"), 
  col = col_funGR,
  column_title_gp = gpar(fontsize = 14, fontface = "bold"),
  width = unit(50, "mm"),
  cluster_columns = FALSE,
  #cluster_rows = FALSE,
  show_row_names = FALSE)
hmap_hier_factors4b <- Heatmap(
  dataHMm2,  name = "mean3",
  row_labels = paste0(rownames(dataHMm2)," ",(tempA[ topDEgenes, ])$Gene_Symbol),
  column_title = paste0("Means Clustered"), 
  col = col_funGR,
  column_title_gp = gpar(fontsize = 14, fontface = "bold"),
  width = unit(50, "mm"),
  #cluster_columns = FALSE,
  #cluster_rows = FALSE,
  show_row_names = FALSE)
dataHMm3<-as.matrix(baseMeansHm3[ topDEgenes, ])
write.table(dataHMm3,"dataHMm3.txt",  sep = "\t")
#baseMeansHm2<-as.matrix(baseMeansHm2)
  
hmap_hier_factors6 <- Heatmap(
  dataHMm3,  name = "logfc1",
  row_labels = paste0(rownames(dataHMm3)," ",(tempA[ topDEgenes, ])$Gene_Symbol),
  column_title = paste0("vs 0h"), 
  col = col_funGR2,
  column_title_gp = gpar(fontsize = 14, fontface = "bold"),
  width = unit(50, "mm"),
  cluster_columns = FALSE,
  show_row_names = FALSE)
dataHMm3b<-dataHMm3[,c(1,4,2,5,3,6)]
hmap_hier_factors6b <- Heatmap(
  dataHMm3b,  name = "logfc2",
  row_labels = paste0(rownames(dataHMm3b)," ",(tempA[ topDEgenes, ])$Gene_Symbol),
  column_title = paste0("vs 0h Rearranged"), 
  col = col_funGR2,
  column_title_gp = gpar(fontsize = 14, fontface = "bold"),
  width = unit(50, "mm"),
  cluster_columns = FALSE,
  show_row_names = FALSE)
hmap_hier_factors6c <- Heatmap(
  dataHMm3,  name = "logfc3",
  row_labels = paste0(rownames(dataHMm3)," ",(tempA[ topDEgenes, ])$Gene_Symbol),
  column_title = paste0("vs 0h"), 
  col = col_funGR2,
  column_title_gp = gpar(fontsize = 14, fontface = "bold"),
  width = unit(50, "mm"),
  #cluster_columns = FALSE,
  show_row_names = FALSE)
hmlist1=hmap_hier_factors37+hmap_hier_factors14+hmap_hier_factors37b+hmap_hier_factors14b
draw(hmlist1, column_title = "Heatmaps on Means (scaled per strain). Genelists combined from VAR37 and VAR14 timecourses p<0.05 lfc1", column_title_gp = gpar(fontsize = 22))
```


```
hmlist2=hmap_hier_factors4+hmap_hier_factors4a+hmap_hier_factors4b
draw(hmlist2, column_title = "Heatmaps on Means (scaled per strain)", column_title_gp = gpar(fontsize = 22))
```


```
hmlist3=hmap_hier_factors4+hmap_hier_factors4a+hmap_hier_factors4b+hmap_hier_factors6+hmap_hier_factors6b+hmap_hier_factors6c
draw(hmlist3, column_title = "Heatmaps on Means (scaled per strain) and logfc Means vs Strain 0h", column_title_gp = gpar(fontsize = 22))
```


```
par(mfrow=c(1,2))
#### Silhouette method
fviz_nbclust(dataHMm3, kmeans, method = "silhouette",k.max = 16)+
  labs(subtitle = "Silhouette method")
```


```
#### Elbow method
fviz_nbclust(dataHMm3, kmeans, method = "wss",k.max = 16) +
  labs(subtitle = "Elbow method")
```


```
####gap stat slow!!!
####set.seed(123)
####fviz_nbclust(dataHMm, kmeans, nstart = 25,  method = "gap_stat", nboot = 100,k.max = 16)+
####  labs(subtitle = "Gap statistic method")
```


```
#kclust8 <- kmeans(dataHMm3, 4)
#silhouette plot
distK<-daisy(dataHMm3)
plot(silhouette(kclust8$cluster, distK), col=1:4, border=NA)
```

#### 3. K-means clustering of means


```
split <- paste0("Cluster\n", kclust8$cluster)
#split <- factor(paste0("Cluster\n", kclust3$cluster), levels=c("Cluster\n3","Cluster\n1","Cluster\n4","Cluster\n5","Cluster\n2","Cluster\n6"))
hmap_k <- Heatmap(dataHMm3, split=split, cluster_row_slices = FALSE,
                  cluster_columns = FALSE,
                  show_row_names = FALSE,
                  name = "Means (scaled per strain",
                  col = col_funGR2,
                  width = unit(50, "mm"),
                  column_title = "Means", 
                  column_title_gp = gpar(fontsize = 16, fontface = "bold"))
hmap_k#+hmap_hier_factors6+hmap_hier_factors5
```


Mean profiles of clusters


```
clustercount<-data.frame(kclust8$cluster)
clustersizes<-table(clustercount$kclust8.cluster)
clusterMeans<-data.frame(kclust8$centers)
clusterMeans1<-data.frame(t(clusterMeans))
clusterMeans1 <- cbind(rownames(clusterMeans1), clusterMeans1)
orderN<-c("Var37TNF_0h_lfc","Var37TNF_6h_lfc","Var37TNF_20h_lfc","Var14TNF_0h_lfc","Var14TNF_6h_lfc","Var14TNF_20h_lfc")#### manual
rownames(clusterMeans1) <- NULL
names(clusterMeans1)[names(clusterMeans1)=="rownames(clusterMeans1)"] <- "Sample"
####clusterMeans1
Strain<-factor(c(rep("VAR37",3),rep("VAR14",3)))####note names
#p1=ggplot(data=dataHmt, aes(x=row.names(dataHmt), y=ENSG00000162551.14),group=Run) + ggtitle("ALPL") +geom_point() +  scale_x_discrete(limits=limitsPlot)+  ylab(ylabPlot)+xlab(xlabPlot)+geom_line(aes(group = Run)) 
pX1<-ggplot(data=clusterMeans1, aes(x=Sample, y=X1,group=1)) +
  geom_line(aes(group = Strain))+  geom_point()+ggtitle(paste("Cluster X1 Profile ",clustersizes[1]," genes"))+  scale_x_discrete(limits=orderN)+
  theme(axis.title.x = element_blank(),axis.text.x = element_text(angle = 90, vjust = 0.5, hjust=1),axis.title.y = element_blank())+ylim (-1.8,1.8)
pX2<-ggplot(data=clusterMeans1, aes(x=Sample, y=X2,group=1)) +
  geom_line(aes(group = Strain))+  geom_point()+ggtitle(paste("Cluster X2 Profile ",clustersizes[2]," genes"))+  scale_x_discrete(limits=orderN)+
  theme(axis.title.x = element_blank(),axis.text.x = element_text(angle = 90, vjust = 0.5, hjust=1),axis.title.y = element_blank())+ylim (-1.8,1.8)
pX3<-ggplot(data=clusterMeans1, aes(x=Sample, y=X3,group=1)) +
  geom_line(aes(group = Strain))+  geom_point()+ggtitle(paste("Cluster X3 Profile ",clustersizes[3]," genes"))+  scale_x_discrete(limits=orderN)+
  theme(axis.title.x = element_blank(),axis.text.x = element_text(angle = 90, vjust = 0.5, hjust=1),axis.title.y = element_blank())+ylim (-1.8,1.8)
pX4<-ggplot(data=clusterMeans1, aes(x=Sample, y=X4,group=1)) +
  geom_line(aes(group = Strain))+  geom_point()+ggtitle(paste("Cluster X4 Profile ",clustersizes[4]," genes"))+  scale_x_discrete(limits=orderN)+
  theme(axis.title.x = element_blank(),axis.text.x = element_text(angle = 90, vjust = 0.5, hjust=1),axis.title.y = element_blank())+ylim (-1.8,1.8)
#pX5<-ggplot(data=clusterMeans1, aes(x=Sample, y=X5,group=1)) +
#  geom_line(aes(group = Strain))+  geom_point()+ggtitle(paste("Cluster X5 Profile ",clustersizes[5]," genes"))+  scale_x_discrete(limits=orderN)+
#  theme(axis.title.x = element_blank(),axis.text.x = element_text(angle = 90, vjust = 0.5, hjust=1),axis.title.y = element_blank())+ylim (-1.8,1.8)
#pX8<-ggplot(data=clusterMeans1, aes(x=Sample, y=X8,group=1)) +
#  geom_line()+  geom_point()+ggtitle(paste("Cluster X8 Profile ",clustersizes[6]," genes"))+  scale_x_discrete(limits=orderN)+
#  theme(axis.title.x = element_blank(),axis.title.y = element_blank())
#plot
multiplot(pX1,pX2,pX4,pX3,     cols=2)
```


```
topDEgenes <- which(tempA$Include=="in")####find indexes
tempAkm<-tempA[ topDEgenes, ]
SymbolsKm<-dplyr::pull(tempAkm, Gene_Symbol)
#### export the gene expression data for the clusters
write.table(clusterMeans,paste0("ClusterMeansKm_",groupsName,".txt"),  sep = "\t")
ClusteredGenes<-data.frame(kclust8$cluster,SymbolsKm,dataHMm3)
write.table(ClusteredGenes,paste0("ScaledDataInClustersKm_",groupsName,".txt"),  sep = "\t")
#head(ClusteredGenes)
```


```
bottomDEgenes<-which(tempA$Include=="out")####find indexes 
bottomG<-tempA[ bottomDEgenes, ]
bottomG<-dplyr::pull(bottomG, Gene_Symbol)
write.table(bottomG,paste0("ipaBottomKmeans_",groupsName,".txt"),  sep = "\t")
                         
topDEgenes <- which(tempA$Include=="in")####find indexes 
tempAkm<-tempA[ topDEgenes, ]
SymbolsKm<-dplyr::pull(tempAkm, Gene_Symbol)
ipaKmeans<-ClusteredGenes
#countsTable <-countsTable[,c(1:15)]####if samples need removing
ipaKmeans<-ipaKmeans[,c(1:2)]
ipaKmeans$name2<-rownames(ipaKmeans)
#ipaKmeans%>% rownames_to_column(var = "rowname")
#ipaKmeans
#rowid_to_column(ipaKmeans)
ipaKmeans = mutate(ipaKmeans, x1= ifelse(ipaKmeans$kclust8.cluster==1, "1", "0"))
ipaKmeans = mutate(ipaKmeans, x2= ifelse(ipaKmeans$kclust8.cluster==2, "1", "0"))
ipaKmeans = mutate(ipaKmeans, x3= ifelse(ipaKmeans$kclust8.cluster==3, "1", "0"))
ipaKmeans = mutate(ipaKmeans, x4= ifelse(ipaKmeans$kclust8.cluster==4, "1", "0"))
#ipaKmeans = mutate(ipaKmeans, x5= ifelse(ipaKmeans$kclust3.cluster==5, "1", "0"))
#ipaKmeans = mutate(ipaKmeans, x6= ifelse(ipaKmeans$kclust3.cluster==6, "1", "0"))
#ipaKmeans = mutate(ipaKmeans, x7= ifelse(ipaKmeans$kclust3.cluster==7, "1", "0"))
#ipaKmeans
write.table(ipaKmeans,paste0("ipaKmeans_",groupsName,".txt"),  sep = "\t")
#head(ipaKmeans)
```


```
ClusteredGenes2<-ClusteredGenes[c(1)]
#ClusteredGenes2
listAll<-list()
for(i in 1:4) {
  clusterName<-paste0("x",i)
  #clusterName<-row.names(subset(ClusteredGenes,ClusteredGenes==i))
  clusterName<-(subset(ClusteredGenes$SymbolsKm,ClusteredGenes==i))
  listAll[[i]]<-clusterName
}
#need to name the vectors in the list, example here is for 8 clusters
names(listAll)<-c("X1", "X2", "X3", "X4")#,"X5", "X6", "X7")
#if you want to rearrange the order
#listAll<-listAll[c("x3", "x7", "x8", "x2", "x6", "x5", "x4", "x1")]
#lapply(listAll, head)
```

#### 4. Annotation of K-means clusters

- CC cellular compartment
- BP biological process
- MF molecular function

The simplify function has been used to cut down on GO redundancy


```
#str(AllGeneNames)
```


```
xread
```


```
Error: object 'xread' not found
```


```
####CC
cgoCC <- compareCluster(geneCluster = listAll, 
                      universe = AllGeneNames,
                      fun = "enrichGO",
                      OrgDb=org.Hs.eg.db, 
                      ####OrgDb=org.Mm.eg.db,
                      keyType="SYMBOL",
                      ont = "CC", 
                      pvalueCutoff=0.05,
                      qvalueCutoff = 0.10)
cgoCC2 <- simplify(cgoCC, cutoff=0.7, by="p.adjust", select_fun=min)
####write as spreadsheet
write.csv(as.data.frame(cgoCC2),paste0("GO_CC_",groupsName,".csv"))
dotplot(cgoCC2,showCategory = 30,
        title = paste0("GO Cellular Compartment ",groupsName))+
  theme(axis.text.x = element_text(angle = 90, vjust = 0.5, hjust=1))
```


Plots and GO data were written to files


```
png(paste0("GO_CC_",groupsName,".png"), width = 1224, height = 824)
dotplot(cgoCC2,showCategory = 30,
        title = paste0("GO Cellular Compartment ",groupsName))+
  theme(axis.text.x = element_text(angle = 90, vjust = 0.5, hjust=1))
dev.off()
```


```
null device 
          1
```


GO BP


```
####CC
cgoBP <- compareCluster(geneCluster = listAll, 
                      universe = AllGeneNames,
                      fun = "enrichGO",
                      OrgDb=org.Hs.eg.db,
                      keyType="SYMBOL",
                      ont = "BP", 
                      pvalueCutoff=0.05,
                      qvalueCutoff = 0.10)
cgoBP2 <- simplify(cgoBP, cutoff=0.7, by="p.adjust", select_fun=min)
####write as spreadsheet
write.csv(as.data.frame(cgoBP2),paste0("GO_BP_",groupsName,".csv"))
dotplot(cgoBP2,showCategory = 30,
        title = paste0("GO Biological Process ",groupsName))+
  theme(axis.text.x = element_text(angle = 90, vjust = 0.5, hjust=1))
```


```
png(paste0("GO_BP_",groupsName,".png"), width = 1024, height = 1224)
dotplot(cgoBP2,showCategory = 30,
        title = paste0("GO Biological Process ",groupsName))+
  theme(axis.text.x = element_text(angle = 90, vjust = 0.5, hjust=1))
dev.off()
```


```
null device 
          1
```


GO MF


```
####MF
cgoMF <- compareCluster(geneCluster = listAll, 
                      universe = AllGeneNames,
                      fun = "enrichGO",
                      OrgDb=org.Hs.eg.db, 
                      keyType="SYMBOL",
                      ont = "MF", 
                      pvalueCutoff=0.05,
                      qvalueCutoff = 0.10)
cgoMF2 <- simplify(cgoMF, cutoff=0.7, by="p.adjust", select_fun=min)
####write as spreadsheet
write.csv(as.data.frame(cgoMF2),paste0("GO_MF_",groupsName,".csv"))
dotplot(cgoMF2,showCategory = 30,
        title = paste0("GO Molecular Function  ",groupsName))+
  theme(axis.text.x = element_text(angle = 90, vjust = 0.5, hjust=1))
```


```
png(paste0("GO_MF_",groupsName,".png"), width = 1424, height = 824)
dotplot(cgoMF2,showCategory = 30,
        title = paste0("GO Molecular Function  ",groupsName))+
  theme(axis.text.x = element_text(angle = 90, vjust = 0.5, hjust=1))
dev.off()
```


```
null device 
          1
```


```
#dbs <- c("GO_Molecular_Function_2018", "GO_Cellular_Component_2018", "GO_Biological_Process_2018")
dbs <- c("Reactome_2016","WikiPathways_2019_Mouse")
if (websiteLive) {    enriched1 <- enrichr((subset(ClusteredGenes$SymbolsKm,ClusteredGenes==1)), dbs)}
if (websiteLive) plotEnrich(enriched1[[1]], showTerms = 30, numChar = 80, y = "Count", orderBy = "P.value", title ="Reactome Enrichment Analysis Cluster 1")
```


```
if (websiteLive) {    enriched2 <- enrichr((subset(ClusteredGenes$SymbolsKm,ClusteredGenes==2)), dbs)}
if (websiteLive) plotEnrich(enriched2[[1]], showTerms = 30, numChar = 80, y = "Count", orderBy = "P.value", title ="Reactome Enrichment Analysis Cluster 2")
```


```
if (websiteLive) {    enriched3 <- enrichr((subset(ClusteredGenes$SymbolsKm,ClusteredGenes==3)), dbs)}
if (websiteLive) plotEnrich(enriched3[[1]], showTerms = 30, numChar = 80, y = "Count", orderBy = "P.value", title ="Reactome Enrichment Analysis Cluster 3")
```


```
if (websiteLive) {    enriched4 <- enrichr((subset(ClusteredGenes$SymbolsKm,ClusteredGenes==4)), dbs)}
if (websiteLive) plotEnrich(enriched4[[1]], showTerms = 30, numChar = 80, y = "Count", orderBy = "P.value", title ="Reactome Enrichment Analysis Cluster 4")
```


```
if (websiteLive) plotEnrich(enriched1[[2]], showTerms = 30, numChar = 80, y = "Count", orderBy = "P.value", title ="WikiPathways Enrichment Analysis Cluster 1")
```


```
if (websiteLive) plotEnrich(enriched2[[2]], showTerms = 30, numChar = 80, y = "Count", orderBy = "P.value", title ="WikiPathways Enrichment Analysis Cluster 2")
```


```
if (websiteLive) plotEnrich(enriched3[[2]], showTerms = 30, numChar = 80, y = "Count", orderBy = "P.value", title ="WikiPathways Enrichment Analysis Cluster 3")
```


```
if (websiteLive) plotEnrich(enriched4[[2]], showTerms = 30, numChar = 80, y = "Count", orderBy = "P.value", title ="WikiPathways Enrichment Analysis Cluster 4")
```


save: once happy with clustering save workspace so that it can be recalled


```
save.image(file="KmFeb2021.RData")
```


Add a new chunk by clicking the *Insert Chunk* button on the toolbar or by pressing *Ctrl+Alt+I*.

When you save the notebook, an HTML file containing the code and output will be saved alongside it (click the *Preview* button or press *Ctrl+Shift+K* to preview the HTML file).

The preview shows you a rendered HTML copy of the contents of the editor. Consequently, unlike *Knit*, *Preview* does not run any R code chunks. Instead, the output of the chunk when it was last run in the editor is displayed.

LS0tCnRpdGxlOiAiS0NvdXBlci9MaXZlcnBvb2wgSy1tZWFucyBSTkFzZXEgQW5hbHlzaXMgTm92ZW1iZXIgMjAyMCIKYXV0aG9yOiBMZW8gWmVlZgp0aGVtZTogdW5pdGVkCm91dHB1dDogCiBodG1sX25vdGVib29rOgogdG9jOiB0cnVlCiB0b2NfZGVwdGg6IDIKIHRvY19mbG9hdDogdHJ1ZQogY29sbGFwc2VkOiBmYWxzZQogc21vb3RoX3Njcm9sbDogZmFsc2UKIGNvZGVfZm9sZGluZzogImhpZGUiCi0tLQojIyBBbmFseXNpcyBTZWN0aW9ucyB7LnRhYnNldCAudGFic2V0LXBpbGxzfQpWaWV3aW5nIGlzIGJldHRlciBpZiBDb2RlIGlzIGhpZGRlbiAoVG9wIFJpZ2h0IGRyb3AgZG93biBsaXN0KQoKCmBgYHtyfQpzaW5rKGZpbGU9IlJzZXNzaW9uSW5mb0RFU2VxMi50eHQiKQpsaWJyYXJ5KCdERVNlcTInKQpsaWJyYXJ5KCJnZ3Bsb3QyIikKbGlicmFyeShyZXNoYXBlMikKIyMjI2xpYnJhcnkodGlkeXZlcnNlKQojIyMjbGlicmFyeShzcGxpdHN0YWNrc2hhcGUpCiMjIyNsaWJyYXJ5KGRhdGEudGFibGUpCmxpYnJhcnkoIlJDb2xvckJyZXdlciIpCmxpYnJhcnkoImdwbG90cyIpCiMjIyNsaWJyYXJ5KCdnZ2RlbmRybycpCmxpYnJhcnkoJ2dncmVwZWwnKQpsaWJyYXJ5KCJkcGx5ciIpCmxpYnJhcnkoIkNvbXBsZXhIZWF0bWFwIikKbGlicmFyeSgiY2x1c3RlclByb2ZpbGVyIikKbGlicmFyeShWZW5uRGlhZ3JhbSkgIyMjIyMjCmxpYnJhcnkoVXBTZXRSKQpsaWJyYXJ5KGdyaWRFeHRyYSkKbGlicmFyeShjbHVzdGVyKQpsaWJyYXJ5KGNpcmNsaXplKQpsaWJyYXJ5KGZhY3RvZXh0cmEpCmxpYnJhcnkoTmJDbHVzdCkKbGlicmFyeSgiYmlvbWFSdCIpCmxpYnJhcnkoIm9yZy5Icy5lZy5kYiIpIyMjI2h1bWFuCmxpYnJhcnkoIm9yZy5NbS5lZy5kYiIpIyMjI21vdXNlCmxpYnJhcnkodmVubikKbGlicmFyeShlbnJpY2hSKQojbGlicmFyeShSZWFjdG9tZVBBKQojIyMjbGlicmFyeShvcmcuQXQudGFpci5kYikjIyMjYXJhYmlkb3BzaXMKc2Vzc2lvbkluZm8oKQpzaW5rKCkKCiMjIyMjIyMjIyMjIyMjIyMjIyMjIyMjIyMjIyMjIyMjIyMjIyMjIyMjCiMjIyNtdWx0aXBsb3QKIyMjIyMjIyMjIyMjIyMjIyMjIyMjIyMjIyMjIyMjIyMjIyMjIyMjIyMKIyMjIyBNdWx0aXBsZSBwbG90IGZ1bmN0aW9uCiMjIyMKIyMjIyBnZ3Bsb3Qgb2JqZWN0cyBjYW4gYmUgcGFzc2VkIGluIC4uLiwgb3IgdG8gcGxvdGxpc3QgKGFzIGEgbGlzdCBvZiBnZ3Bsb3Qgb2JqZWN0cykKIyMjIyAtIGNvbHM6ICAgTnVtYmVyIG9mIGNvbHVtbnMgaW4gbGF5b3V0CiMjIyMgLSBsYXlvdXQ6IEEgbWF0cml4IHNwZWNpZnlpbmcgdGhlIGxheW91dC4gSWYgcHJlc2VudCwgJ2NvbHMnIGlzIGlnbm9yZWQuCgojIyMjIElmIHRoZSBsYXlvdXQgaXMgc29tZXRoaW5nIGxpa2UgbWF0cml4KGMoMSwyLDMsMyksIG5yb3c9MiwgYnlyb3c9VFJVRSksCiMjIyMgdGhlbiBwbG90IDEgd2lsbCBnbyBpbiB0aGUgdXBwZXIgbGVmdCwgMiB3aWxsIGdvIGluIHRoZSB1cHBlciByaWdodCwgYW5kCiMjIyMgMyB3aWxsIGdvIGFsbCB0aGUgd2F5IGFjcm9zcyB0aGUgYm90dG9tLgoKbXVsdGlwbG90IDwtIGZ1bmN0aW9uKC4uLiwgcGxvdGxpc3Q9TlVMTCwgZmlsZSwgY29scz0xLCBsYXlvdXQ9TlVMTCkgewogIGxpYnJhcnkoZ3JpZCkKICAKICAjIyMjIE1ha2UgYSBsaXN0IGZyb20gdGhlIC4uLiBhcmd1bWVudHMgYW5kIHBsb3RsaXN0CiAgcGxvdHMgPC0gYyhsaXN0KC4uLiksIHBsb3RsaXN0KQogIAogIG51bVBsb3RzID0gbGVuZ3RoKHBsb3RzKQogIAogICMjIyMgSWYgbGF5b3V0IGlzIE5VTEwsIHRoZW4gdXNlICdjb2xzJyB0byBkZXRlcm1pbmUgbGF5b3V0CiAgaWYgKGlzLm51bGwobGF5b3V0KSkgewogICAgIyMjIyBNYWtlIHRoZSBwYW5lbAogICAgIyMjIyBuY29sOiBOdW1iZXIgb2YgY29sdW1ucyBvZiBwbG90cwogICAgIyMjIyBucm93OiBOdW1iZXIgb2Ygcm93cyBuZWVkZWQsIGNhbGN1bGF0ZWQgZnJvbSAjIyMjIG9mIGNvbHMKICAgIGxheW91dCA8LSBtYXRyaXgoc2VxKDEsIGNvbHMgKiBjZWlsaW5nKG51bVBsb3RzL2NvbHMpKSwKICAgICAgICAgICAgICAgICAgICAgbmNvbCA9IGNvbHMsIG5yb3cgPSBjZWlsaW5nKG51bVBsb3RzL2NvbHMpKQogIH0KICAKICBpZiAobnVtUGxvdHM9PTEpIHsKICAgIHByaW50KHBsb3RzW1sxXV0pCiAgICAKICB9IGVsc2UgewogICAgIyMjIyBTZXQgdXAgdGhlIHBhZ2UKICAgIGdyaWQubmV3cGFnZSgpCiAgICBwdXNoVmlld3BvcnQodmlld3BvcnQobGF5b3V0ID0gZ3JpZC5sYXlvdXQobnJvdyhsYXlvdXQpLCBuY29sKGxheW91dCkpKSkKICAgIAogICAgIyMjIyBNYWtlIGVhY2ggcGxvdCwgaW4gdGhlIGNvcnJlY3QgbG9jYXRpb24KICAgIGZvciAoaSBpbiAxOm51bVBsb3RzKSB7CiAgICAgICMjIyMgR2V0IHRoZSBpLGogbWF0cml4IHBvc2l0aW9ucyBvZiB0aGUgcmVnaW9ucyB0aGF0IGNvbnRhaW4gdGhpcyBzdWJwbG90CiAgICAgIG1hdGNoaWR4IDwtIGFzLmRhdGEuZnJhbWUod2hpY2gobGF5b3V0ID09IGksIGFyci5pbmQgPSBUUlVFKSkKICAgICAgCiAgICAgIHByaW50KHBsb3RzW1tpXV0sIHZwID0gdmlld3BvcnQobGF5b3V0LnBvcy5yb3cgPSBtYXRjaGlkeCRyb3csCiAgICAgICAgICAgICAgICAgICAgICAgICAgICAgICAgICAgICAgbGF5b3V0LnBvcy5jb2wgPSBtYXRjaGlkeCRjb2wpKQogICAgfQogIH0KfQojIyMjZnVuY3Rpb24gbXkgY29kZSBlZGl0IG9mIHBsb3RQQ0EKIyMjIyMjIyMjIyMjIyMjIyMjIyMjIyMjIyMjIyMjIyMjIyMjCnBsb3RQQ0FMZW88LWZ1bmN0aW9uICh4LCBpbnRncm91cCA9ICJUcmVhdG1lbnQiLCBudG9wID0gNTAwLCByZXR1cm5EYXRhID0gRkFMU0UsIFBDeD0xLCBQQ3k9MikKewogICMjIyNydiA8LSByb3dWYXJzKGFzc2F5KHgpKQogIHJ2ID0gYXBwbHkoKGFzc2F5KHgpKSwgMSwgdmFyKQogIHNlbGVjdCA8LSBvcmRlcihydiwgZGVjcmVhc2luZyA9IFRSVUUpW3NlcV9sZW4obWluKG50b3AsIAogICAgICAgICAgICAgICAgICAgICAgICAgICAgICAgICAgICAgICAgICAgICAgICAgICAgIGxlbmd0aChydikpKV0KICBwY2EgPC0gcHJjb21wKHQoYXNzYXkoeClbc2VsZWN0LCBdKSkKICBwZXJjZW50VmFyIDwtIHBjYSRzZGV2XjIvc3VtKHBjYSRzZGV2XjIpCiAgaWYgKCFhbGwoaW50Z3JvdXAgJWluJSBuYW1lcyhjb2xEYXRhKHgpKSkpIHsKICAgIHN0b3AoInRoZSBhcmd1bWVudCAnaW50Z3JvdXAnIHNob3VsZCBzcGVjaWZ5IGNvbHVtbnMgb2YgY29sRGF0YShkZHMpIikKICB9CiAgaW50Z3JvdXAuZGYgPC0gYXMuZGF0YS5mcmFtZShjb2xEYXRhKHgpWywgaW50Z3JvdXAsIGRyb3AgPSBGQUxTRV0pCiAgZ3JvdXAgPC0gZmFjdG9yKGFwcGx5KGludGdyb3VwLmRmLCAxLCBwYXN0ZSwgY29sbGFwc2UgPSAiIDogIikpCiAgZCA8LSBkYXRhLmZyYW1lKFBDWCA9IHBjYSR4WywgUEN4XSwgUENZID0gcGNhJHhbLCBQQ3ldLCBncm91cCA9IGdyb3VwLCAKICAgICAgICAgICAgICAgICAgaW50Z3JvdXAuZGYsIG5hbWVzID0gY29sbmFtZXMoeCkpCiAgaWYgKHJldHVybkRhdGEpIHsKICAgIGF0dHIoZCwgInBlcmNlbnRWYXIiKSA8LSBwZXJjZW50VmFyW1BDeDpQQ3ldCiAgICByZXR1cm4oZCkKICB9CiAgZ2dwbG90KGRhdGEgPSBkLCBhZXNfc3RyaW5nKHggPSAiUENYIiwgeSA9ICJQQ1kiLCBjb2xvciA9ICJncm91cCIpKSArIAogICAgIyMjI2dncGxvdChkYXRhID0gZCwgYWVzX3N0cmluZyh4ID0gIlBDWCIsIHkgPSAiUENZIiwgY29sb3I9VGdmYjEsIHNoYXBlPVRyZWF0bWVudCkpICsgCiAgICBnZW9tX3BvaW50KHNpemUgPSAzKSArIHhsYWIocGFzdGUwKCJQQyIsUEN4LCI6ICIsIHJvdW5kKHBlcmNlbnRWYXJbMV0gKiAKICAgICAgICAgICAgICAgICAgICAgICAgICAgICAgICAgICAgICAgICAgICAgICAgICAgICAgICAgICAgICAxMDApLCAiJSB2YXJpYW5jZSIpKSArIHlsYWIocGFzdGUwKCJQQyIsUEN5LCI6ICIsIHJvdW5kKHBlcmNlbnRWYXJbMl0gKiAKICAgICAgICAgICAgICAgICAgICAgICAgICAgICAgICAgICAgICAgICAgICAgICAgICAgICAgICAgICAgICAgICAgICAgICAgICAgICAgICAgICAgICAgICAgICAgICAgICAgICAgICAgICAgICAgICAgICAgICAgMTAwKSwgIiUgdmFyaWFuY2UiKSkKfQpgYGAKCgpgYGB7cn0KY29sX2Z1biA9IGNvbG9yUmFtcDIoYygtMSwtMC4yLCAwLDAuMiwgMSksIGMoImJsdWUiLCJjeWFuIiwgImdyZXk5MCIsIm9yYW5nZSIsICJyZWQiKSkjaGVhdG1hcCBjb2xvdXJzCmNvbF9mdW5HUiA9IGNvbG9yUmFtcDIoYygtMS41LCAwLCAxLjUpLCBjKCJncmVlbiIsICJibGFjayIsICJyZWQiKSkKY29sX2Z1bkdSMiA9IGNvbG9yUmFtcDIoYygtMiwgMCwgMiksIGMoImdyZWVuIiwgImJsYWNrIiwgInJlZCIpKQpjb2xvcnNWMyA8LSBjKCJjb3JuZmxvd2VyYmx1ZSIsICAiYnJvd24xIiwib3JhbmdlMiIpI1Zlbm4gY29sb3Vycwpjb2xvcnNWMiA8LSBjKCJtZWRpdW1vcmNoaWQxIiwgICJjaGFydHJldXNlMyIpI1Zlbm4gY29sb3Vycwpjb2xvcnNWNDwtYygiY29ybmZsb3dlcmJsdWUiLCAib3JhbmdlMiIsICJncmVlbjMiLCJyZWQiKSNWZW5uIGNvbG91cnMKY29sb3JzVjU8LWMoImNvcm5mbG93ZXJibHVlIiwgIm9yYW5nZTIiLCAiZ3JlZW4zIiwicHVycGxlIiwicmVkIikjVmVubiBjb2xvdXJzCiNjb2xfZnVuKHNlcSgtMywgMykpCmBgYAoKCgojIyMgREUgR2VuZSBTZWxlY3Rpb24KCiMjIyMxLiBHZW5lbGlzdCBTZWxlY3Rpb24KCmBgYHtyfQpncm91cHNOYW1lPC0iUjFfUjRfa21lYW5zX3EwLjA1IgpgYGAKCgpgYGB7cn0KY291bnRzVGFibGU8LXJlYWQuZGVsaW0oIlJOQXNlcTIwMTlKdWx5XzUudHh0IiwgaGVhZGVyID0gVFJVRSwgc2VwID0gIlx0IixjaGVjay5uYW1lcz1GQUxTRSxyb3cubmFtZXM9MSkKaGVhZChjb3VudHNUYWJsZSkKYGBgCgpgYGB7cn0KQWxsR2VuZU5hbWVzPC1jb3VudHNUYWJsZSRHZW5lX1N5bWJvbAojaGVhZChBbGxHZW5lTmFtZXMpCmBgYAoKYGBge3J9CnRlbXBBPC1jb3VudHNUYWJsZQpgYGAKCmBgYHtyfQoKCnRvcERFZ2VuZXMgPC0gd2hpY2godGVtcEEkcGFkal9SMV9WYXIzN19Ib3Vyc182aF92c18waDwwLjA1JigodGVtcEEkVmFyMzdUTkZfMGhfbWVhbj4xMCl8KHRlbXBBJFZhcjM3VE5GXzZoX21lYW4+MTApKSYhaXMubmEodGVtcEEkcGFkal9SMV9WYXIzN19Ib3Vyc182aF92c18waCkpIyMjI2ZpbmQgaW5kZXhlcyAKbGlzdEE8LXRlbXBBWyB0b3BERWdlbmVzLCBdJEdlbmVfU3ltYm9sCnRvcERFZ2VuZXMgPC0gd2hpY2godGVtcEEkcGFkal9SMV9WYXIzN19Ib3Vyc18yMGhfdnNfMGg8MC4wNSYoKHRlbXBBJFZhcjM3VE5GXzBoX21lYW4+MTApfCh0ZW1wQSRWYXIzN1RORl8yMGhfbWVhbj4xMCkpJiFpcy5uYSh0ZW1wQSRwYWRqX1IxX1ZhcjM3X0hvdXJzXzIwaF92c18waCkpIyMjI2ZpbmQgaW5kZXhlcyAKbGlzdEI8LXRlbXBBWyB0b3BERWdlbmVzLCBdJEdlbmVfU3ltYm9sCnRvcERFZ2VuZXMgPC0gd2hpY2godGVtcEEkcGFkal9SMV9WYXIzN19Ib3Vyc18yMGhfdnNfNmg8MC4wNSYoKHRlbXBBJFZhcjM3VE5GXzIwaF9tZWFuPjEwKXwodGVtcEEkVmFyMzdUTkZfNmhfbWVhbj4xMCkpJiFpcy5uYSh0ZW1wQSRwYWRqX1IxX1ZhcjM3X0hvdXJzXzIwaF92c182aCkpIyMjI2ZpbmQgaW5kZXhlcyAKbGlzdEM8LXRlbXBBWyB0b3BERWdlbmVzLCBdJEdlbmVfU3ltYm9sCnZlbm5xPC12ZW5uLmRpYWdyYW0oeCA9IGxpc3QobGlzdEEsbGlzdEIsbGlzdEMpLCMsbGlzdEQpICwKICAgICAgICAgICAgY2F0ZWdvcnkubmFtZXMgPSBjKCJWYXIzN182aHYwaCIsIlZhcjM3XzIwaHYwaCIsIlZhcjM3XzIwaHY2aCIpLAogICAgICAgICAgICBtYWluPSJwYWRqPDAuMDUiLAogICAgICAgICAgICBmaWxlbmFtZSA9IE5VTEwsICBzY2FsZWQgPSBGQUxTRSwgZmlsbCA9IGNvbG9yc1YzLCBjYXQuY29sID0gY29sb3JzVjMsIGNhdC5jZXggPSAxLCBjYXQuZGlzdD0wLjEsICBtYXJnaW4gPSAwLjMpCgp0b3BERWdlbmVzIDwtIHdoaWNoKHRlbXBBJHB2YWx1ZV9SMV9WYXIzN19Ib3Vyc182aF92c18waDwwLjA1JmFicyh0ZW1wQSRsb2cyRm9sZENoYW5nZV9SMV9WYXIzN19Ib3Vyc182aF92c18waCk+MSYoKHRlbXBBJFZhcjM3VE5GXzZoX21lYW4+MTApfCh0ZW1wQSRWYXIzN1RORl8waF9tZWFuPjEwKSkmIWlzLm5hKHRlbXBBJHB2YWx1ZV9SMV9WYXIzN19Ib3Vyc182aF92c18waCkpIyMjI2ZpbmQgaW5kZXhlcyAKbGlzdEE8LXRlbXBBWyB0b3BERWdlbmVzLCBdJEdlbmVfU3ltYm9sCnRvcERFZ2VuZXMgPC0gd2hpY2godGVtcEEkcHZhbHVlX1IxX1ZhcjM3X0hvdXJzXzIwaF92c18waDwwLjA1JmFicyh0ZW1wQSRsb2cyRm9sZENoYW5nZV9SMV9WYXIzN19Ib3Vyc18yMGhfdnNfMGgpPjEmKCh0ZW1wQSRWYXIzN1RORl8waF9tZWFuPjEwKXwodGVtcEEkVmFyMzdUTkZfMjBoX21lYW4+MTApKSYhaXMubmEodGVtcEEkcHZhbHVlX1IxX1ZhcjM3X0hvdXJzXzIwaF92c18waCkpIyMjI2ZpbmQgaW5kZXhlcyAKbGlzdEI8LXRlbXBBWyB0b3BERWdlbmVzLCBdJEdlbmVfU3ltYm9sCnRvcERFZ2VuZXMgPC0gd2hpY2godGVtcEEkcHZhbHVlX1IxX1ZhcjM3X0hvdXJzXzIwaF92c182aDwwLjA1JmFicyh0ZW1wQSRsb2cyRm9sZENoYW5nZV9SMV9WYXIzN19Ib3Vyc18yMGhfdnNfNmgpPjEmKCh0ZW1wQSRWYXIzN1RORl82aF9tZWFuPjEwKXwodGVtcEEkVmFyMzdUTkZfMjBoX21lYW4+MTApKSYhaXMubmEodGVtcEEkcHZhbHVlX1IxX1ZhcjM3X0hvdXJzXzIwaF92c182aCkpIyMjI2ZpbmQgaW5kZXhlcyAKbGlzdEM8LXRlbXBBWyB0b3BERWdlbmVzLCBdJEdlbmVfU3ltYm9sCnZlbm5wPC12ZW5uLmRpYWdyYW0oeCA9IGxpc3QobGlzdEEsbGlzdEIsbGlzdEMpICwKICAgICAgICAgICAgY2F0ZWdvcnkubmFtZXMgPSBjKCJWYXIzN182aHYwaCIsIlZhcjM3XzIwaHYwaCIsIlZhcjM3XzIwaHY2aCIpLAogICAgICAgICAgICBtYWluPSJwdmFsdWU8MC4wNSZmb2xkIGNoYW5nZT4yIiwKICAgICAgICAgICAgZmlsZW5hbWUgPSBOVUxMLCAgc2NhbGVkID0gRkFMU0UsIGZpbGwgPSBjb2xvcnNWMywgY2F0LmNvbCA9IGNvbG9yc1YzLCBjYXQuY2V4ID0gMSwgY2F0LmRpc3Q9MC4xLCAgbWFyZ2luID0gMC4zKQoKdG9wREVnZW5lcyA8LSB3aGljaCh0ZW1wQSRwYWRqX1I0VmFyMTRUTkZfSG91cnNfNmhfdnNfMGg8MC4wNSYoKHRlbXBBJFZhcjE0VE5GXzBoX21lYW4+MTApfCh0ZW1wQSRWYXIxNFRORl82aF9tZWFuPjEwKSkmIWlzLm5hKHRlbXBBJHBhZGpfUjRWYXIxNFRORl9Ib3Vyc182aF92c18waCkpIyMjI2ZpbmQgaW5kZXhlcyAKbGlzdEE8LXRlbXBBWyB0b3BERWdlbmVzLCBdJEdlbmVfU3ltYm9sCnRvcERFZ2VuZXMgPC0gd2hpY2godGVtcEEkcGFkal9SNFZhcjE0VE5GX0hvdXJzXzIwaF92c18waDwwLjA1JigodGVtcEEkVmFyMTRUTkZfMjBoX21lYW4+MTApfCh0ZW1wQSRWYXIxNFRORl8waF9tZWFuPjEwKSkmIWlzLm5hKHRlbXBBJHBhZGpfUjRWYXIxNFRORl9Ib3Vyc18yMGhfdnNfMGgpKSMjIyNmaW5kIGluZGV4ZXMgCmxpc3RCPC10ZW1wQVsgdG9wREVnZW5lcywgXSRHZW5lX1N5bWJvbAp0b3BERWdlbmVzIDwtIHdoaWNoKHRlbXBBJHBhZGpfUjFfVmFyMzdfSG91cnNfMjBoX3ZzXzZoPDAuMDUmKCh0ZW1wQSRWYXIxNFRORl8yMGhfbWVhbj4xMCl8KHRlbXBBJFZhcjE0VE5GXzZoX21lYW4+MTApKSYhaXMubmEodGVtcEEkcGFkal9SMV9WYXIzN19Ib3Vyc18yMGhfdnNfNmgpKSMjIyNmaW5kIGluZGV4ZXMgCmxpc3RDPC10ZW1wQVsgdG9wREVnZW5lcywgXSRHZW5lX1N5bWJvbAp2ZW5ucTI8LXZlbm4uZGlhZ3JhbSh4ID0gbGlzdChsaXN0QSxsaXN0QixsaXN0QyksIyxsaXN0RCkgLAogICAgICAgICAgICBjYXRlZ29yeS5uYW1lcyA9IGMoIlZhcjE0XzZodjBoIiwiVmFyMTRfMjBodjBoIiwiVmFyMTRfMjBodjZoIiksCiAgICAgICAgICAgIG1haW49InBhZGo8MC4wNSIsCiAgICAgICAgICAgIGZpbGVuYW1lID0gTlVMTCwgIHNjYWxlZCA9IEZBTFNFLCBmaWxsID0gY29sb3JzVjMsIGNhdC5jb2wgPSBjb2xvcnNWMywgY2F0LmNleCA9IDEsIGNhdC5kaXN0PTAuMSwgIG1hcmdpbiA9IDAuMykKCnRvcERFZ2VuZXMgPC0gd2hpY2godGVtcEEkcHZhbHVlX1I0VmFyMTRUTkZfSG91cnNfNmhfdnNfMGg8MC4wNSZhYnModGVtcEEkbG9nMkZvbGRDaGFuZ2VfUjRWYXIxNFRORl9Ib3Vyc182aF92c18waCk+MSYoKHRlbXBBJFZhcjE0VE5GXzBoX21lYW4+MTApfCh0ZW1wQSRWYXIxNFRORl82aF9tZWFuPjEwKSkmIWlzLm5hKHRlbXBBJHB2YWx1ZV9SNFZhcjE0VE5GX0hvdXJzXzZoX3ZzXzBoKSkjIyMjZmluZCBpbmRleGVzIApsaXN0QTwtdGVtcEFbIHRvcERFZ2VuZXMsIF0kR2VuZV9TeW1ib2wKdG9wREVnZW5lcyA8LSB3aGljaCh0ZW1wQSRwdmFsdWVfUjRWYXIxNFRORl9Ib3Vyc18yMGhfdnNfMGg8MC4wNSZhYnModGVtcEEkbG9nMkZvbGRDaGFuZ2VfUjRWYXIxNFRORl9Ib3Vyc18yMGhfdnNfMGgpPjEmKCh0ZW1wQSRWYXIxNFRORl8yMGhfbWVhbj4xMCl8KHRlbXBBJFZhcjE0VE5GXzBoX21lYW4+MTApKSYhaXMubmEodGVtcEEkcHZhbHVlX1I0VmFyMTRUTkZfSG91cnNfMjBoX3ZzXzBoKSkjIyMjZmluZCBpbmRleGVzIApsaXN0QjwtdGVtcEFbIHRvcERFZ2VuZXMsIF0kR2VuZV9TeW1ib2wKdG9wREVnZW5lcyA8LSB3aGljaCh0ZW1wQSRwdmFsdWVfUjRWYXIxNFRORl9Ib3Vyc18yMGhfdnNfNmg8MC4wNSZhYnModGVtcEEkbG9nMkZvbGRDaGFuZ2VfUjRWYXIxNFRORl9Ib3Vyc18yMGhfdnNfNmgpPjEmKCh0ZW1wQSRWYXIxNFRORl8yMGhfbWVhbj4xMCl8KHRlbXBBJFZhcjE0VE5GXzZoX21lYW4+MTApKSYhaXMubmEodGVtcEEkcHZhbHVlX1I0VmFyMTRUTkZfSG91cnNfMjBoX3ZzXzZoKSkjIyMjZmluZCBpbmRleGVzIApsaXN0QzwtdGVtcEFbIHRvcERFZ2VuZXMsIF0kR2VuZV9TeW1ib2wKdmVubnAyPC12ZW5uLmRpYWdyYW0oeCA9IGxpc3QobGlzdEEsbGlzdEIsbGlzdEMpICwKICAgICAgICAgICAgY2F0ZWdvcnkubmFtZXMgPSBjKCJWYXIxNF82aHYwaCIsIlZhcjE0XzIwaHYwaCIsIlZhcjE0XzIwaHY2aCIpLAogICAgICAgICAgICBtYWluPSJwdmFsdWU8MC4wNSZmb2xkIGNoYW5nZT4yIiwKICAgICAgICAgICAgZmlsZW5hbWUgPSBOVUxMLCAgc2NhbGVkID0gRkFMU0UsIGZpbGwgPSBjb2xvcnNWMywgY2F0LmNvbCA9IGNvbG9yc1YzLCBjYXQuY2V4ID0gMSwgY2F0LmRpc3Q9MC4xLCAgbWFyZ2luID0gMC4zKQoKCmBgYAoKYGBge3J9Cgp0b3BERWdlbmVzIDwtIHdoaWNoKCh0ZW1wQSRwYWRqX1IxX1ZhcjM3X0hvdXJzXzZoX3ZzXzBoPDAuMDUmKCh0ZW1wQSRWYXIzN1RORl82aF9tZWFuPjEwKXwodGVtcEEkVmFyMzdUTkZfMGhfbWVhbj4xMCkpJiFpcy5uYSh0ZW1wQSRwYWRqX1IxX1ZhcjM3X0hvdXJzXzZoX3ZzXzBoKSl8IAoodGVtcEEkcGFkal9SMV9WYXIzN19Ib3Vyc18yMGhfdnNfMGg8MC4wNSYoKHRlbXBBJFZhcjM3VE5GXzBoX21lYW4+MTApfCh0ZW1wQSRWYXIzN1RORl8yMGhfbWVhbj4xMCkpJiFpcy5uYSh0ZW1wQSRwYWRqX1IxX1ZhcjM3X0hvdXJzXzIwaF92c18waCkpfAoodGVtcEEkcGFkal9SMV9WYXIzN19Ib3Vyc18yMGhfdnNfNmg8MC4wNSYoKHRlbXBBJFZhcjM3VE5GXzZoX21lYW4+MTApfCh0ZW1wQSRWYXIzN1RORl8yMGhfbWVhbj4xMCkpJiFpcy5uYSh0ZW1wQSRwYWRqX1IxX1ZhcjM3X0hvdXJzXzIwaF92c182aCkpCikKbGlzdEE8LXRlbXBBWyB0b3BERWdlbmVzLCBdJEdlbmVfU3ltYm9sCgp0b3BERWdlbmVzIDwtIHdoaWNoKCh0ZW1wQSRwdmFsdWVfUjFfVmFyMzdfSG91cnNfNmhfdnNfMGg8MC4wNSZhYnModGVtcEEkbG9nMkZvbGRDaGFuZ2VfUjFfVmFyMzdfSG91cnNfNmhfdnNfMGgpPjEmKCh0ZW1wQSRWYXIzN1RORl82aF9tZWFuPjEwKXwodGVtcEEkVmFyMzdUTkZfMGhfbWVhbj4xMCkpJiFpcy5uYSh0ZW1wQSRwdmFsdWVfUjFfVmFyMzdfSG91cnNfNmhfdnNfMGgpKXwgCih0ZW1wQSRwdmFsdWVfUjFfVmFyMzdfSG91cnNfMjBoX3ZzXzBoPDAuMDUmYWJzKHRlbXBBJGxvZzJGb2xkQ2hhbmdlX1IxX1ZhcjM3X0hvdXJzXzIwaF92c18waCk+MSYoKHRlbXBBJFZhcjM3VE5GXzBoX21lYW4+MTApfCh0ZW1wQSRWYXIzN1RORl8yMGhfbWVhbj4xMCkpJiFpcy5uYSh0ZW1wQSRwdmFsdWVfUjFfVmFyMzdfSG91cnNfMjBoX3ZzXzBoKSl8IAoodGVtcEEkcHZhbHVlX1IxX1ZhcjM3X0hvdXJzXzIwaF92c182aDwwLjA1JmFicyh0ZW1wQSRsb2cyRm9sZENoYW5nZV9SMV9WYXIzN19Ib3Vyc18yMGhfdnNfNmgpPjEmKCh0ZW1wQSRWYXIzN1RORl82aF9tZWFuPjEwKXwodGVtcEEkVmFyMzdUTkZfMjBoX21lYW4+MTApKSYhaXMubmEodGVtcEEkcHZhbHVlX1IxX1ZhcjM3X0hvdXJzXzIwaF92c182aCkpIAogKSMjIyNmaW5kIGluZGV4ZXMgCmxpc3RCPC10ZW1wQVsgdG9wREVnZW5lcywgXSRHZW5lX1N5bWJvbAoKdG9wREVnZW5lcyA8LSB3aGljaCgodGVtcEEkcGFkal9SNFZhcjE0VE5GX0hvdXJzXzZoX3ZzXzBoPDAuMDUmKCh0ZW1wQSRWYXIxNFRORl8waF9tZWFuPjEwKXwodGVtcEEkVmFyMTRUTkZfNmhfbWVhbj4xMCkpJiFpcy5uYSh0ZW1wQSRwYWRqX1I0VmFyMTRUTkZfSG91cnNfNmhfdnNfMGgpKXwgCih0ZW1wQSRwYWRqX1I0VmFyMTRUTkZfSG91cnNfMjBoX3ZzXzBoPDAuMDUmKCh0ZW1wQSRWYXIxNFRORl8yMGhfbWVhbj4xMCl8KHRlbXBBJFZhcjE0VE5GXzBoX21lYW4+MTApKSYhaXMubmEodGVtcEEkcGFkal9SNFZhcjE0VE5GX0hvdXJzXzIwaF92c18waCkpfAoodGVtcEEkcGFkal9SNFZhcjE0VE5GX0hvdXJzXzIwaF92c182aDwwLjA1JigodGVtcEEkVmFyMTRUTkZfMjBoX21lYW4+MTApfCh0ZW1wQSRWYXIxNFRORl82aF9tZWFuPjEwKSkmIWlzLm5hKHRlbXBBJHBhZGpfUjRWYXIxNFRORl9Ib3Vyc18yMGhfdnNfNmgpKQopCmxpc3RBMjwtdGVtcEFbIHRvcERFZ2VuZXMsIF0kR2VuZV9TeW1ib2wKCnRvcERFZ2VuZXMgPC0gd2hpY2goKHRlbXBBJHB2YWx1ZV9SNFZhcjE0VE5GX0hvdXJzXzZoX3ZzXzBoPDAuMDUmYWJzKHRlbXBBJGxvZzJGb2xkQ2hhbmdlX1I0VmFyMTRUTkZfSG91cnNfNmhfdnNfMGgpPjEmKCh0ZW1wQSRWYXIxNFRORl8waF9tZWFuPjEwKXwodGVtcEEkVmFyMTRUTkZfNmhfbWVhbj4xMCkpJiFpcy5uYSh0ZW1wQSRwdmFsdWVfUjRWYXIxNFRORl9Ib3Vyc182aF92c18waCkpfCAKKHRlbXBBJHB2YWx1ZV9SNFZhcjE0VE5GX0hvdXJzXzIwaF92c18waDwwLjA1JmFicyh0ZW1wQSRsb2cyRm9sZENoYW5nZV9SNFZhcjE0VE5GX0hvdXJzXzIwaF92c18waCk+MSYoKHRlbXBBJFZhcjE0VE5GXzIwaF9tZWFuPjEwKXwodGVtcEEkVmFyMTRUTkZfMGhfbWVhbj4xMCkpJiFpcy5uYSh0ZW1wQSRwdmFsdWVfUjRWYXIxNFRORl9Ib3Vyc18yMGhfdnNfMGgpKXwgCih0ZW1wQSRwdmFsdWVfUjRWYXIxNFRORl9Ib3Vyc18yMGhfdnNfNmg8MC4wNSZhYnModGVtcEEkbG9nMkZvbGRDaGFuZ2VfUjRWYXIxNFRORl9Ib3Vyc18yMGhfdnNfNmgpPjEmKCh0ZW1wQSRWYXIxNFRORl8yMGhfbWVhbj4xMCl8KHRlbXBBJFZhcjE0VE5GXzZoX21lYW4+MTApKSYhaXMubmEodGVtcEEkcHZhbHVlX1I0VmFyMTRUTkZfSG91cnNfMjBoX3ZzXzZoKSkKICkjIyMjZmluZCBpbmRleGVzIApsaXN0QjI8LXRlbXBBWyB0b3BERWdlbmVzLCBdJEdlbmVfU3ltYm9sCgoKCnZlbm5wcTwtdmVubi5kaWFncmFtKHggPSBsaXN0KGxpc3RBLGxpc3RCLGxpc3RBMixsaXN0QjIpICwKICAgICAgICAgICAgY2F0ZWdvcnkubmFtZXMgPSBjKCJWMzdwYWRqPDAuMDUiLCJWMzdwPDAuMDUmZmM+MiIsIlYxNHBhZGo8MC4wNSIsIlYxNHA8MC4wNSZmYz4yIiksCiAgICAgICAgICAgIG1haW49InBhZGogY29tcGFyZWQgdG8gcHZhbHVlIiwKICAgICAgICAgICAgZmlsZW5hbWUgPSBOVUxMLCAgc2NhbGVkID0gRkFMU0UsIGZpbGwgPSBjb2xvcnNWNCwgY2F0LmNvbCA9IGNvbG9yc1Y0LCBjYXQuY2V4ID0gMSwgY2F0LmRpc3Q9MC4zLCAgbWFyZ2luID0gMC4xNSkKYGBgCgpgYGB7cixmaWcuaGVpZ2h0ID0gMTAsZmlnLndpZHRoID0gMjB9CmdyaWQuYXJyYW5nZShnVHJlZShjaGlsZHJlbj12ZW5ucSksIGdUcmVlKGNoaWxkcmVuPXZlbm5wKSwgbmNvbD0yLHRvcD0iUjEgVmFyMzcgVE5GIikKYGBgCmBgYHtyLGZpZy5oZWlnaHQgPSAxMCxmaWcud2lkdGggPSAyMH0KZ3JpZC5hcnJhbmdlKGdUcmVlKGNoaWxkcmVuPXZlbm5xMiksIGdUcmVlKGNoaWxkcmVuPXZlbm5wMiksIG5jb2w9Mix0b3A9IlI0IFZhcjE0IFRORiIpCmBgYApgYGB7cixmaWcuaGVpZ2h0ID0gMTAsZmlnLndpZHRoID0gMTB9CmdyaWQuYXJyYW5nZShnVHJlZShjaGlsZHJlbj12ZW5ucHEpLCBuY29sPTEsdG9wPSJSNCBWYXIxNCBUTkYiKQpgYGAKCiMjIyBSMSAmIFI0IFZBUjM3IFZBUjE0IFRORiBrLW1lYW5zIHEwLjA1CiFbRGVzaWduXShkZXNpZ24ucG5nKQoKCmBgYHtyfQoKI3RlbXBBPC1yZXNBbGxbLWMoMTA6MzApIF0KdGVtcEE8LWNvdW50c1RhYmxlCiNyb3duYW1lcyh0ZW1wQSkKcm93bmFtZXModGVtcEEpIDwtIE5VTEwKdGVtcEEgPSBtdXRhdGUodGVtcEEsIEluY2x1ZGU9CiAgICAgICAgICAgICAgICAgICBpZmVsc2UodGVtcEEkcGFkal9SMV9WYXIzN19Ib3Vyc182aF92c18waDwwLjA1JigodGVtcEEkVmFyMzdUTkZfNmhfbWVhbj4xMCl8KHRlbXBBJFZhcjM3VE5GXzBoX21lYW4+MTApKSYhaXMubmEodGVtcEEkcGFkal9SMV9WYXIzN19Ib3Vyc182aF92c18waCksICJpbiIsCiAgICAgICAgICAgICAgICAgICAgICAgICAgaWZlbHNlKHRlbXBBJHBhZGpfUjFfVmFyMzdfSG91cnNfMjBoX3ZzXzBoPDAuMDUmKCh0ZW1wQSRWYXIzN1RORl8waF9tZWFuPjEwKXwodGVtcEEkVmFyMzdUTkZfMjBoX21lYW4+MTApKSYhaXMubmEodGVtcEEkcGFkal9SMV9WYXIzN19Ib3Vyc18yMGhfdnNfMGgpLCAiaW4iLAogICAgICAgICAgICAgICAgICAgICAgICAgICAgICAgICBpZmVsc2UodGVtcEEkcGFkal9SMV9WYXIzN19Ib3Vyc18yMGhfdnNfNmg8MC4wNSYoKHRlbXBBJFZhcjM3VE5GXzZoX21lYW4+MTApfCh0ZW1wQSRWYXIzN1RORl8yMGhfbWVhbj4xMCkpJiFpcy5uYSh0ZW1wQSRwYWRqX1IxX1ZhcjM3X0hvdXJzXzIwaF92c182aCksICJpbiIsCiAgICAgICAgICAgICAgICAgICAgICAgICAgICAgICAgICAgICAgICBpZmVsc2UodGVtcEEkcGFkal9SNFZhcjE0VE5GX0hvdXJzXzZoX3ZzXzBoPDAuMDUmKCh0ZW1wQSRWYXIxNFRORl8waF9tZWFuPjEwKXwodGVtcEEkVmFyMTRUTkZfNmhfbWVhbj4xMCkpJiFpcy5uYSh0ZW1wQSRwYWRqX1I0VmFyMTRUTkZfSG91cnNfNmhfdnNfMGgpLCAiaW4iLAogICAgICAgICAgICAgICAgICAgICAgICAgICAgICAgICAgICAgICAgICAgICAgIGlmZWxzZSh0ZW1wQSRwYWRqX1I0VmFyMTRUTkZfSG91cnNfMjBoX3ZzXzBoPDAuMDUmKCh0ZW1wQSRWYXIxNFRORl8yMGhfbWVhbj4xMCl8KHRlbXBBJFZhcjE0VE5GXzBoX21lYW4+MTApKSYhaXMubmEodGVtcEEkcGFkal9SNFZhcjE0VE5GX0hvdXJzXzIwaF92c18waCksImluIiwKICAgICAgICAgICAgICAgICAgICAgICAgICAgICAgICAgICAgICAgICAgICAgICAgICAgICAgaWZlbHNlKHRlbXBBJHBhZGpfUjRWYXIxNFRORl9Ib3Vyc18yMGhfdnNfNmg8MC4wNSYoKHRlbXBBJFZhcjE0VE5GXzIwaF9tZWFuPjEwKXwodGVtcEEkVmFyMTRUTkZfNmhfbWVhbj4xMCkpJiFpcy5uYSh0ZW1wQSRwYWRqX1I0VmFyMTRUTkZfSG91cnNfMjBoX3ZzXzZoKSwiaW4iLAogICAgICAgICAgICAgICAgICAgICAgICAgICAgICAgICAgICAgICAgICAgICAgICAgIm91dCIpKSkpKSkpCgoKI3RlbXBBCiMjIyNsaWJyYXJ5KGRwbHlyKQp0ZW1wQSAlPiUKICAgICBncm91cF9ieShJbmNsdWRlKSAlPiUgCiAgICAgdGFsbHkoKQpgYGAKCmBgYHtyfQp0b3BERWdlbmVzIDwtIHdoaWNoKHRlbXBBJEluY2x1ZGU9PSJpbiIpIyMjI2ZpbmQgaW5kZXhlcyAKYGBgCgpgYGB7cn0KaGVhZChjb3VudHNUYWJsZSkKYGBgCgpgYGB7cn0KYmFzZU1lYW5zSG0gPC1jb3VudHNUYWJsZVssYyg0ODo1MCwxMTAsMTEyLDExMyldCmhlYWQoYmFzZU1lYW5zSG0pCmBgYAoKIyMjIyBOQiBQbGVhc2UgY2hlY2sgY29sdW1ucyB1c2VkIGFuZCByZW5hbWVkIGZvciBwbG90cwpgYGB7cn0KI2Jhc2VNZWFuc0htIDwtY291bnRzVGFibGVbLGMoNjA6NjMpXQpiYXNlTWVhbnNIbSA8LWNvdW50c1RhYmxlWyxjKDQ4OjUwLDExMCwxMTIsMTEzKV0KCmhlYWQoYmFzZU1lYW5zSG0pCgoKdGFpbChiYXNlTWVhbnNIbVsgdG9wREVnZW5lcywgXSkKCgpiYXNlTWVhbnNIbSRWYXIzN1RORl8waDwtYmFzZU1lYW5zSG0kVmFyMzdUTkZfMGhfbWVhbgpiYXNlTWVhbnNIbSRWYXIzN1RORl82aDwtYmFzZU1lYW5zSG0kVmFyMzdUTkZfNmhfbWVhbgpiYXNlTWVhbnNIbSRWYXIzN1RORl8yMGg8LWJhc2VNZWFuc0htJFZhcjM3VE5GXzIwaF9tZWFuCgpiYXNlTWVhbnNIbSRWYXIxNFRORl8waDwtYmFzZU1lYW5zSG0kVmFyMTRUTkZfMGhfbWVhbgpiYXNlTWVhbnNIbSRWYXIxNFRORl82aDwtYmFzZU1lYW5zSG0kVmFyMTRUTkZfNmhfbWVhbgpiYXNlTWVhbnNIbSRWYXIxNFRORl8yMGg8LWJhc2VNZWFuc0htJFZhcjE0VE5GXzIwaF9tZWFuCgpiYXNlTWVhbnNIbSA8LWJhc2VNZWFuc0htWyxjKDc6MTIpXQoKI3JlcGxhY2UgbG93IHZhbHVlcyB3aXRoIDAKYmFzZU1lYW5zSG0kVmFyMzdUTkZfMGhbYmFzZU1lYW5zSG0kVmFyMzdUTkZfMGg8MTBdPC0wCmJhc2VNZWFuc0htJFZhcjM3VE5GXzZoW2Jhc2VNZWFuc0htJFZhcjM3VE5GXzZoPDEwXTwtMApiYXNlTWVhbnNIbSRWYXIzN1RORl8yMGhbYmFzZU1lYW5zSG0kVmFyMzdUTkZfMjBoPDEwXTwtMApiYXNlTWVhbnNIbSRWYXIxNFRORl8waFtiYXNlTWVhbnNIbSRWYXIxNFRORl8waDwxMF08LTAKYmFzZU1lYW5zSG0kVmFyMTRUTkZfNmhbYmFzZU1lYW5zSG0kVmFyMTRUTkZfNmg8MTBdPC0wCmJhc2VNZWFuc0htJFZhcjE0VE5GXzIwaFtiYXNlTWVhbnNIbSRWYXIxNFRORl8yMGg8MTBdPC0wCnRhaWwoYmFzZU1lYW5zSG0pCmJhc2VNZWFuc0htIDwtIGxvZzIoYmFzZU1lYW5zSG0rMSkKdGFpbChiYXNlTWVhbnNIbSkKI2Jhc2VNZWFuc0htTSA8LWJhc2VNZWFuc0htMlssYygxOjgpXQojaGVhZChiYXNlTWVhbnNIbU0pCmBgYAoKYGBge3J9CnRvcERFZ2VuZXMgPC0gd2hpY2godGVtcEEkSW5jbHVkZT09ImluIikjIyMjZmluZCBpbmRleGVzIApgYGAKCmBgYHtyfQojc2NhbGUgVmFyMzUgYW5kIFZhcjE0IHNlcGFyYXRlbHkKdmFyMTRtbjwtYmFzZU1lYW5zSG1bLGMoNDo2KV0KdmFyMTRtbjwtIHQoYXMubWF0cml4KHZhcjE0bW4pKQp2YXIxNG1uIDwtIHQoc2NhbGUodmFyMTRtbikpCiNoZWFkKHZhcjE0bW4pCmJhc2VNZWFuc0htMjwtYmFzZU1lYW5zSG1bLGMoMTozKV0KYmFzZU1lYW5zSG0yPC0gdChhcy5tYXRyaXgoYmFzZU1lYW5zSG0yKSkKYmFzZU1lYW5zSG0yIDwtIHQoc2NhbGUoYmFzZU1lYW5zSG0yKSkKYmFzZU1lYW5zSG0yIDwtIGFzLmRhdGEuZnJhbWUoY2JpbmQoYmFzZU1lYW5zSG0yLCB2YXIxNG1uKSkKYmFzZU1lYW5zSG0yW2lzLm5hKGJhc2VNZWFuc0htMildIDwtIDAKI2hlYWQoYmFzZU1lYW5zSG0yKQoKYmFzZU1lYW5zSG0yJFZhcjM3VE5GXzBoX2xmYzwtYmFzZU1lYW5zSG0yJFZhcjM3VE5GXzBoLWJhc2VNZWFuc0htMiRWYXIzN1RORl8waApiYXNlTWVhbnNIbTIkVmFyMzdUTkZfNmhfbGZjPC1iYXNlTWVhbnNIbTIkVmFyMzdUTkZfNmgtYmFzZU1lYW5zSG0yJFZhcjM3VE5GXzBoCmJhc2VNZWFuc0htMiRWYXIzN1RORl8yMGhfbGZjPC1iYXNlTWVhbnNIbTIkVmFyMzdUTkZfMjBoLWJhc2VNZWFuc0htMiRWYXIzN1RORl8waApiYXNlTWVhbnNIbTIkVmFyMTRUTkZfMGhfbGZjPC1iYXNlTWVhbnNIbTIkVmFyMTRUTkZfMGgtYmFzZU1lYW5zSG0yJFZhcjE0VE5GXzBoCmJhc2VNZWFuc0htMiRWYXIxNFRORl82aF9sZmM8LWJhc2VNZWFuc0htMiRWYXIxNFRORl82aC1iYXNlTWVhbnNIbTIkVmFyMTRUTkZfMGgKYmFzZU1lYW5zSG0yJFZhcjE0VE5GXzIwaF9sZmM8LWJhc2VNZWFuc0htMiRWYXIxNFRORl8yMGgtYmFzZU1lYW5zSG0yJFZhcjE0VE5GXzBoCiNiYXNlTWVhbnNIbTE8LWJhc2VNZWFuc0htMlssYygxOjYpXQpiYXNlTWVhbnNIbTM8LWJhc2VNZWFuc0htMlssYyg3OjEyKV0KaGVhZChiYXNlTWVhbnNIbTMpCmJhc2VNZWFuc0htMjwtYmFzZU1lYW5zSG0yWyxjKDE6NildCmhlYWQoYmFzZU1lYW5zSG0yKQoKYGBgCgpgYGB7cn0KZGF0YUhNbTI8LWFzLm1hdHJpeChiYXNlTWVhbnNIbTJbIHRvcERFZ2VuZXMsIF0pCnRhaWwoZGF0YUhNbTIpCmBgYAoKCiMjIyMyLiBIaWVyYWNoaWNhbCBjbHVzdGVyaW5nIG9mIG1lYW5zIChpbmRpdmlkdWFsIHNhbXBsZXMgYWRkZWQgZm9yIGluc3BlY3Rpb24pCmBgYHtyLGZpZy5oZWlnaHQgPSAxMixmaWcud2lkdGggPSAyMH0KIyMjI21lYW4KCmRhdGFITW0yPC1hcy5tYXRyaXgoYmFzZU1lYW5zSG0yWyB0b3BERWdlbmVzLCBdKQpkYXRhSE1tMl8zNzwtZGF0YUhNbTJbLGMoMSwyLDMpXQpkYXRhSE1tMl8xNDwtZGF0YUhNbTJbLGMoNCw1LDYpXQoKaG1hcF9oaWVyX2ZhY3RvcnMzNyA8LSBIZWF0bWFwKAogIGRhdGFITW0yXzM3LCAgbmFtZSA9ICJtZWFuMzciLAogIHJvd19sYWJlbHMgPSBwYXN0ZTAocm93bmFtZXMoZGF0YUhNbTJfMzcpLCIgIiwodGVtcEFbIHRvcERFZ2VuZXMsIF0pJEdlbmVfU3ltYm9sKSwKICBjb2x1bW5fdGl0bGUgPSBwYXN0ZTAoIk1lYW5zVjM3IiksIAogIGNvbCA9IGNvbF9mdW5HUiwKICBjb2x1bW5fdGl0bGVfZ3AgPSBncGFyKGZvbnRzaXplID0gMTQsIGZvbnRmYWNlID0gImJvbGQiKSwKICB3aWR0aCA9IHVuaXQoMjUsICJtbSIpLAogIGNsdXN0ZXJfY29sdW1ucyA9IEZBTFNFLAogICNjbHVzdGVyX3Jvd3MgPSBGQUxTRSwKICBzaG93X3Jvd19uYW1lcyA9IEZBTFNFKQoKaG1hcF9oaWVyX2ZhY3RvcnMzN2IgPC0gSGVhdG1hcCgKICBkYXRhSE1tMl8zNywgIG5hbWUgPSAibWVhbjM3YiIsCiAgcm93X2xhYmVscyA9IHBhc3RlMChyb3duYW1lcyhkYXRhSE1tMl8zNyksIiAiLCh0ZW1wQVsgdG9wREVnZW5lcywgXSkkR2VuZV9TeW1ib2wpLAogIGNvbHVtbl90aXRsZSA9IHBhc3RlMCgiTWVhbnNWMzciKSwgCiAgY29sID0gY29sX2Z1bkdSLAogIGNvbHVtbl90aXRsZV9ncCA9IGdwYXIoZm9udHNpemUgPSAxNCwgZm9udGZhY2UgPSAiYm9sZCIpLAogIHdpZHRoID0gdW5pdCgyNSwgIm1tIiksCiAgI2NsdXN0ZXJfY29sdW1ucyA9IEZBTFNFLAogICNjbHVzdGVyX3Jvd3MgPSBGQUxTRSwKICBzaG93X3Jvd19uYW1lcyA9IEZBTFNFKQoKaG1hcF9oaWVyX2ZhY3RvcnMxNCA8LSBIZWF0bWFwKAogIGRhdGFITW0yXzE0LCAgbmFtZSA9ICJtZWFuMTQiLAogIHJvd19sYWJlbHMgPSBwYXN0ZTAocm93bmFtZXMoZGF0YUhNbTJfMTQpLCIgIiwodGVtcEFbIHRvcERFZ2VuZXMsIF0pJEdlbmVfU3ltYm9sKSwKICBjb2x1bW5fdGl0bGUgPSBwYXN0ZTAoIk1lYW5zVjE0IiksIAogIGNvbCA9IGNvbF9mdW5HUiwKICBjb2x1bW5fdGl0bGVfZ3AgPSBncGFyKGZvbnRzaXplID0gMTQsIGZvbnRmYWNlID0gImJvbGQiKSwKICB3aWR0aCA9IHVuaXQoMjUsICJtbSIpLAogIGNsdXN0ZXJfY29sdW1ucyA9IEZBTFNFLAogICNjbHVzdGVyX3Jvd3MgPSBGQUxTRSwKICBzaG93X3Jvd19uYW1lcyA9IEZBTFNFKQoKaG1hcF9oaWVyX2ZhY3RvcnMxNGIgPC0gSGVhdG1hcCgKICBkYXRhSE1tMl8xNCwgIG5hbWUgPSAibWVhbjE0YiIsCiAgcm93X2xhYmVscyA9IHBhc3RlMChyb3duYW1lcyhkYXRhSE1tMl8xNCksIiAiLCh0ZW1wQVsgdG9wREVnZW5lcywgXSkkR2VuZV9TeW1ib2wpLAogIGNvbHVtbl90aXRsZSA9IHBhc3RlMCgiTWVhbnNWMTQiKSwgCiAgY29sID0gY29sX2Z1bkdSLAogIGNvbHVtbl90aXRsZV9ncCA9IGdwYXIoZm9udHNpemUgPSAxNCwgZm9udGZhY2UgPSAiYm9sZCIpLAogIHdpZHRoID0gdW5pdCgyNSwgIm1tIiksCiAgI2NsdXN0ZXJfY29sdW1ucyA9IEZBTFNFLAogICNjbHVzdGVyX3Jvd3MgPSBGQUxTRSwKICBzaG93X3Jvd19uYW1lcyA9IEZBTFNFKQoKI3dyaXRlLnRhYmxlKGRhdGFITW0yLCJkYXRhSE1tMi50eHQiLCAgc2VwID0gIlx0IikKCmhtYXBfaGllcl9mYWN0b3JzNCA8LSBIZWF0bWFwKAogIGRhdGFITW0yLCAgbmFtZSA9ICJtZWFuMSIsCiAgcm93X2xhYmVscyA9IHBhc3RlMChyb3duYW1lcyhkYXRhSE1tMiksIiAiLCh0ZW1wQVsgdG9wREVnZW5lcywgXSkkR2VuZV9TeW1ib2wpLAogIGNvbHVtbl90aXRsZSA9IHBhc3RlMCgiTWVhbnMiKSwgCiAgY29sID0gY29sX2Z1bkdSLAogIGNvbHVtbl90aXRsZV9ncCA9IGdwYXIoZm9udHNpemUgPSAxNCwgZm9udGZhY2UgPSAiYm9sZCIpLAogIHdpZHRoID0gdW5pdCg1MCwgIm1tIiksCiAgY2x1c3Rlcl9jb2x1bW5zID0gRkFMU0UsCiAgI2NsdXN0ZXJfcm93cyA9IEZBTFNFLAogIHNob3dfcm93X25hbWVzID0gRkFMU0UpCgpkYXRhSE1tMmI8LWRhdGFITW0yWyxjKDEsNCwyLDUsMyw2KV0KaG1hcF9oaWVyX2ZhY3RvcnM0YSA8LSBIZWF0bWFwKAogIGRhdGFITW0yYiwgIG5hbWUgPSAibWVhbjIiLAogIHJvd19sYWJlbHMgPSBwYXN0ZTAocm93bmFtZXMoZGF0YUhNbTJiKSwiICIsKHRlbXBBWyB0b3BERWdlbmVzLCBdKSRHZW5lX1N5bWJvbCksCiAgY29sdW1uX3RpdGxlID0gcGFzdGUwKCJNZWFucyBSZWFycmFuZ2VkIiksIAogIGNvbCA9IGNvbF9mdW5HUiwKICBjb2x1bW5fdGl0bGVfZ3AgPSBncGFyKGZvbnRzaXplID0gMTQsIGZvbnRmYWNlID0gImJvbGQiKSwKICB3aWR0aCA9IHVuaXQoNTAsICJtbSIpLAogIGNsdXN0ZXJfY29sdW1ucyA9IEZBTFNFLAogICNjbHVzdGVyX3Jvd3MgPSBGQUxTRSwKICBzaG93X3Jvd19uYW1lcyA9IEZBTFNFKQoKaG1hcF9oaWVyX2ZhY3RvcnM0YiA8LSBIZWF0bWFwKAogIGRhdGFITW0yLCAgbmFtZSA9ICJtZWFuMyIsCiAgcm93X2xhYmVscyA9IHBhc3RlMChyb3duYW1lcyhkYXRhSE1tMiksIiAiLCh0ZW1wQVsgdG9wREVnZW5lcywgXSkkR2VuZV9TeW1ib2wpLAogIGNvbHVtbl90aXRsZSA9IHBhc3RlMCgiTWVhbnMgQ2x1c3RlcmVkIiksIAogIGNvbCA9IGNvbF9mdW5HUiwKICBjb2x1bW5fdGl0bGVfZ3AgPSBncGFyKGZvbnRzaXplID0gMTQsIGZvbnRmYWNlID0gImJvbGQiKSwKICB3aWR0aCA9IHVuaXQoNTAsICJtbSIpLAogICNjbHVzdGVyX2NvbHVtbnMgPSBGQUxTRSwKICAjY2x1c3Rlcl9yb3dzID0gRkFMU0UsCiAgc2hvd19yb3dfbmFtZXMgPSBGQUxTRSkKCmRhdGFITW0zPC1hcy5tYXRyaXgoYmFzZU1lYW5zSG0zWyB0b3BERWdlbmVzLCBdKQp3cml0ZS50YWJsZShkYXRhSE1tMywiZGF0YUhNbTMudHh0IiwgIHNlcCA9ICJcdCIpCiNiYXNlTWVhbnNIbTI8LWFzLm1hdHJpeChiYXNlTWVhbnNIbTIpCiAgCmhtYXBfaGllcl9mYWN0b3JzNiA8LSBIZWF0bWFwKAogIGRhdGFITW0zLCAgbmFtZSA9ICJsb2dmYzEiLAogIHJvd19sYWJlbHMgPSBwYXN0ZTAocm93bmFtZXMoZGF0YUhNbTMpLCIgIiwodGVtcEFbIHRvcERFZ2VuZXMsIF0pJEdlbmVfU3ltYm9sKSwKICBjb2x1bW5fdGl0bGUgPSBwYXN0ZTAoInZzIDBoIiksIAogIGNvbCA9IGNvbF9mdW5HUjIsCiAgY29sdW1uX3RpdGxlX2dwID0gZ3Bhcihmb250c2l6ZSA9IDE0LCBmb250ZmFjZSA9ICJib2xkIiksCiAgd2lkdGggPSB1bml0KDUwLCAibW0iKSwKICBjbHVzdGVyX2NvbHVtbnMgPSBGQUxTRSwKICBzaG93X3Jvd19uYW1lcyA9IEZBTFNFKQoKZGF0YUhNbTNiPC1kYXRhSE1tM1ssYygxLDQsMiw1LDMsNildCmhtYXBfaGllcl9mYWN0b3JzNmIgPC0gSGVhdG1hcCgKICBkYXRhSE1tM2IsICBuYW1lID0gImxvZ2ZjMiIsCiAgcm93X2xhYmVscyA9IHBhc3RlMChyb3duYW1lcyhkYXRhSE1tM2IpLCIgIiwodGVtcEFbIHRvcERFZ2VuZXMsIF0pJEdlbmVfU3ltYm9sKSwKICBjb2x1bW5fdGl0bGUgPSBwYXN0ZTAoInZzIDBoIFJlYXJyYW5nZWQiKSwgCiAgY29sID0gY29sX2Z1bkdSMiwKICBjb2x1bW5fdGl0bGVfZ3AgPSBncGFyKGZvbnRzaXplID0gMTQsIGZvbnRmYWNlID0gImJvbGQiKSwKICB3aWR0aCA9IHVuaXQoNTAsICJtbSIpLAogIGNsdXN0ZXJfY29sdW1ucyA9IEZBTFNFLAogIHNob3dfcm93X25hbWVzID0gRkFMU0UpCgpobWFwX2hpZXJfZmFjdG9yczZjIDwtIEhlYXRtYXAoCiAgZGF0YUhNbTMsICBuYW1lID0gImxvZ2ZjMyIsCiAgcm93X2xhYmVscyA9IHBhc3RlMChyb3duYW1lcyhkYXRhSE1tMyksIiAiLCh0ZW1wQVsgdG9wREVnZW5lcywgXSkkR2VuZV9TeW1ib2wpLAogIGNvbHVtbl90aXRsZSA9IHBhc3RlMCgidnMgMGgiKSwgCiAgY29sID0gY29sX2Z1bkdSMiwKICBjb2x1bW5fdGl0bGVfZ3AgPSBncGFyKGZvbnRzaXplID0gMTQsIGZvbnRmYWNlID0gImJvbGQiKSwKICB3aWR0aCA9IHVuaXQoNTAsICJtbSIpLAogICNjbHVzdGVyX2NvbHVtbnMgPSBGQUxTRSwKICBzaG93X3Jvd19uYW1lcyA9IEZBTFNFKQoKaG1saXN0MT1obWFwX2hpZXJfZmFjdG9yczM3K2htYXBfaGllcl9mYWN0b3JzMTQraG1hcF9oaWVyX2ZhY3RvcnMzN2IraG1hcF9oaWVyX2ZhY3RvcnMxNGIKZHJhdyhobWxpc3QxLCBjb2x1bW5fdGl0bGUgPSAiSGVhdG1hcHMgb24gTWVhbnMgKHNjYWxlZCBwZXIgc3RyYWluKS4gR2VuZWxpc3RzIGNvbWJpbmVkIGZyb20gVkFSMzcgYW5kIFZBUjE0IHRpbWVjb3Vyc2VzIHBhZGo8MC4wNSIsIGNvbHVtbl90aXRsZV9ncCA9IGdwYXIoZm9udHNpemUgPSAyMikpCgpobWxpc3QyPWhtYXBfaGllcl9mYWN0b3JzNCtobWFwX2hpZXJfZmFjdG9yczRhK2htYXBfaGllcl9mYWN0b3JzNGIKZHJhdyhobWxpc3QyLCBjb2x1bW5fdGl0bGUgPSAiSGVhdG1hcHMgb24gTWVhbnMgKHNjYWxlZCBwZXIgc3RyYWluKSIsIGNvbHVtbl90aXRsZV9ncCA9IGdwYXIoZm9udHNpemUgPSAyMikpCgpobWxpc3QzPWhtYXBfaGllcl9mYWN0b3JzNCtobWFwX2hpZXJfZmFjdG9yczRhK2htYXBfaGllcl9mYWN0b3JzNGIraG1hcF9oaWVyX2ZhY3RvcnM2K2htYXBfaGllcl9mYWN0b3JzNmIraG1hcF9oaWVyX2ZhY3RvcnM2YwpkcmF3KGhtbGlzdDMsIGNvbHVtbl90aXRsZSA9ICJIZWF0bWFwcyBvbiBNZWFucyAoc2NhbGVkIHBlciBzdHJhaW4pIGFuZCBsb2dmYyBNZWFucyB2cyBTdHJhaW4gMGgiLCBjb2x1bW5fdGl0bGVfZ3AgPSBncGFyKGZvbnRzaXplID0gMjIpKQpgYGAKCgoKCgoKCmBgYHtyLGZpZy5oZWlnaHQgPSA3LGZpZy53aWR0aCA9MTIgfQpwYXIobWZyb3c9YygxLDIpKQojIyMjIFNpbGhvdWV0dGUgbWV0aG9kCmZ2aXpfbmJjbHVzdChkYXRhSE1tMywga21lYW5zLCBtZXRob2QgPSAic2lsaG91ZXR0ZSIsay5tYXggPSAxNikrCiAgbGFicyhzdWJ0aXRsZSA9ICJTaWxob3VldHRlIG1ldGhvZCIpCgojIyMjIEVsYm93IG1ldGhvZApmdml6X25iY2x1c3QoZGF0YUhNbTMsIGttZWFucywgbWV0aG9kID0gIndzcyIsay5tYXggPSAxNikgKwogIGxhYnMoc3VidGl0bGUgPSAiRWxib3cgbWV0aG9kIikKCmBgYAoKYGBge3J9CiMjIyNnYXAgc3RhdCBzbG93ISEhCiMjIyNzZXQuc2VlZCgxMjMpCiMjIyNmdml6X25iY2x1c3QoZGF0YUhNbSwga21lYW5zLCBuc3RhcnQgPSAyNSwgIG1ldGhvZCA9ICJnYXBfc3RhdCIsIG5ib290ID0gMTAwLGsubWF4ID0gMTYpKwojIyMjICBsYWJzKHN1YnRpdGxlID0gIkdhcCBzdGF0aXN0aWMgbWV0aG9kIikKYGBgCgoKYGBge3IsZmlnLmhlaWdodCA9IDd9CiNrY2x1c3Q3IDwtIGttZWFucyhkYXRhSE1tMywgNCkKI3NpbGhvdWV0dGUgcGxvdApkaXN0SzwtZGFpc3koZGF0YUhNbTMpCnBsb3Qoc2lsaG91ZXR0ZShrY2x1c3Q3JGNsdXN0ZXIsIGRpc3RLKSwgY29sPTE6NCwgYm9yZGVyPU5BKQoKYGBgCgojIyMjMy4gSy1tZWFucyBjbHVzdGVyaW5nIG9mIG1lYW5zIApgYGB7cixmaWcuaGVpZ2h0ID0gMTAsZmlnLndpZHRoID0gMjB9CnNwbGl0IDwtIHBhc3RlMCgiQ2x1c3RlclxuIiwga2NsdXN0NyRjbHVzdGVyKQojc3BsaXQgPC0gZmFjdG9yKHBhc3RlMCgiQ2x1c3RlclxuIiwga2NsdXN0MyRjbHVzdGVyKSwgbGV2ZWxzPWMoIkNsdXN0ZXJcbjMiLCJDbHVzdGVyXG4xIiwiQ2x1c3RlclxuNCIsIkNsdXN0ZXJcbjUiLCJDbHVzdGVyXG4yIiwiQ2x1c3RlclxuNiIpKQpobWFwX2sgPC0gSGVhdG1hcChkYXRhSE1tMywgc3BsaXQ9c3BsaXQsIGNsdXN0ZXJfcm93X3NsaWNlcyA9IEZBTFNFLAogICAgICAgICAgICAgICAgICBjbHVzdGVyX2NvbHVtbnMgPSBGQUxTRSwKICAgICAgICAgICAgICAgICAgc2hvd19yb3dfbmFtZXMgPSBGQUxTRSwKICAgICAgICAgICAgICAgICAgbmFtZSA9ICJNZWFucyAoc2NhbGVkIHBlciBzdHJhaW4iLAogICAgICAgICAgICAgICAgICBjb2wgPSBjb2xfZnVuR1IyLAogICAgICAgICAgICAgICAgICB3aWR0aCA9IHVuaXQoNTAsICJtbSIpLAogICAgICAgICAgICAgICAgICBjb2x1bW5fdGl0bGUgPSAiTWVhbnMiLCAKICAgICAgICAgICAgICAgICAgY29sdW1uX3RpdGxlX2dwID0gZ3Bhcihmb250c2l6ZSA9IDE2LCBmb250ZmFjZSA9ICJib2xkIikpCgpobWFwX2sjK2htYXBfaGllcl9mYWN0b3JzNitobWFwX2hpZXJfZmFjdG9yczUKYGBgCgpNZWFuIHByb2ZpbGVzIG9mIGNsdXN0ZXJzCgpgYGB7cixmaWcuaGVpZ2h0ID0gOSxmaWcud2lkdGggPSA4fQpjbHVzdGVyY291bnQ8LWRhdGEuZnJhbWUoa2NsdXN0NyRjbHVzdGVyKQpjbHVzdGVyc2l6ZXM8LXRhYmxlKGNsdXN0ZXJjb3VudCRrY2x1c3Q3LmNsdXN0ZXIpCmNsdXN0ZXJNZWFuczwtZGF0YS5mcmFtZShrY2x1c3Q3JGNlbnRlcnMpCmNsdXN0ZXJNZWFuczE8LWRhdGEuZnJhbWUodChjbHVzdGVyTWVhbnMpKQpjbHVzdGVyTWVhbnMxIDwtIGNiaW5kKHJvd25hbWVzKGNsdXN0ZXJNZWFuczEpLCBjbHVzdGVyTWVhbnMxKQpvcmRlck48LWMoIlZhcjM3VE5GXzBoX2xmYyIsIlZhcjM3VE5GXzZoX2xmYyIsIlZhcjM3VE5GXzIwaF9sZmMiLCJWYXIxNFRORl8waF9sZmMiLCJWYXIxNFRORl82aF9sZmMiLCJWYXIxNFRORl8yMGhfbGZjIikjIyMjIG1hbnVhbAoKcm93bmFtZXMoY2x1c3Rlck1lYW5zMSkgPC0gTlVMTApuYW1lcyhjbHVzdGVyTWVhbnMxKVtuYW1lcyhjbHVzdGVyTWVhbnMxKT09InJvd25hbWVzKGNsdXN0ZXJNZWFuczEpIl0gPC0gIlNhbXBsZSIKIyMjI2NsdXN0ZXJNZWFuczEKU3RyYWluPC1mYWN0b3IoYyhyZXAoIlZBUjM3IiwzKSxyZXAoIlZBUjE0IiwzKSkpIyMjI25vdGUgbmFtZXMKI3AxPWdncGxvdChkYXRhPWRhdGFIbXQsIGFlcyh4PXJvdy5uYW1lcyhkYXRhSG10KSwgeT1FTlNHMDAwMDAxNjI1NTEuMTQpLGdyb3VwPVJ1bikgKyBnZ3RpdGxlKCJBTFBMIikgK2dlb21fcG9pbnQoKSArICBzY2FsZV94X2Rpc2NyZXRlKGxpbWl0cz1saW1pdHNQbG90KSsgIHlsYWIoeWxhYlBsb3QpK3hsYWIoeGxhYlBsb3QpK2dlb21fbGluZShhZXMoZ3JvdXAgPSBSdW4pKSAKCgpwWDE8LWdncGxvdChkYXRhPWNsdXN0ZXJNZWFuczEsIGFlcyh4PVNhbXBsZSwgeT1YMSxncm91cD0xKSkgKwogIGdlb21fbGluZShhZXMoZ3JvdXAgPSBTdHJhaW4pKSsgIGdlb21fcG9pbnQoKStnZ3RpdGxlKHBhc3RlKCJDbHVzdGVyIFgxIFByb2ZpbGUgIixjbHVzdGVyc2l6ZXNbMV0sIiBnZW5lcyIpKSsgIHNjYWxlX3hfZGlzY3JldGUobGltaXRzPW9yZGVyTikrCiAgdGhlbWUoYXhpcy50aXRsZS54ID0gZWxlbWVudF9ibGFuaygpLGF4aXMudGV4dC54ID0gZWxlbWVudF90ZXh0KGFuZ2xlID0gOTAsIHZqdXN0ID0gMC41LCBoanVzdD0xKSxheGlzLnRpdGxlLnkgPSBlbGVtZW50X2JsYW5rKCkpK3lsaW0gKC0xLjgsMS44KQpwWDI8LWdncGxvdChkYXRhPWNsdXN0ZXJNZWFuczEsIGFlcyh4PVNhbXBsZSwgeT1YMixncm91cD0xKSkgKwogIGdlb21fbGluZShhZXMoZ3JvdXAgPSBTdHJhaW4pKSsgIGdlb21fcG9pbnQoKStnZ3RpdGxlKHBhc3RlKCJDbHVzdGVyIFgyIFByb2ZpbGUgIixjbHVzdGVyc2l6ZXNbMl0sIiBnZW5lcyIpKSsgIHNjYWxlX3hfZGlzY3JldGUobGltaXRzPW9yZGVyTikrCiAgdGhlbWUoYXhpcy50aXRsZS54ID0gZWxlbWVudF9ibGFuaygpLGF4aXMudGV4dC54ID0gZWxlbWVudF90ZXh0KGFuZ2xlID0gOTAsIHZqdXN0ID0gMC41LCBoanVzdD0xKSxheGlzLnRpdGxlLnkgPSBlbGVtZW50X2JsYW5rKCkpK3lsaW0gKC0xLjgsMS44KQpwWDM8LWdncGxvdChkYXRhPWNsdXN0ZXJNZWFuczEsIGFlcyh4PVNhbXBsZSwgeT1YMyxncm91cD0xKSkgKwogIGdlb21fbGluZShhZXMoZ3JvdXAgPSBTdHJhaW4pKSsgIGdlb21fcG9pbnQoKStnZ3RpdGxlKHBhc3RlKCJDbHVzdGVyIFgzIFByb2ZpbGUgIixjbHVzdGVyc2l6ZXNbM10sIiBnZW5lcyIpKSsgIHNjYWxlX3hfZGlzY3JldGUobGltaXRzPW9yZGVyTikrCiAgdGhlbWUoYXhpcy50aXRsZS54ID0gZWxlbWVudF9ibGFuaygpLGF4aXMudGV4dC54ID0gZWxlbWVudF90ZXh0KGFuZ2xlID0gOTAsIHZqdXN0ID0gMC41LCBoanVzdD0xKSxheGlzLnRpdGxlLnkgPSBlbGVtZW50X2JsYW5rKCkpK3lsaW0gKC0xLjgsMS44KQpwWDQ8LWdncGxvdChkYXRhPWNsdXN0ZXJNZWFuczEsIGFlcyh4PVNhbXBsZSwgeT1YNCxncm91cD0xKSkgKwogIGdlb21fbGluZShhZXMoZ3JvdXAgPSBTdHJhaW4pKSsgIGdlb21fcG9pbnQoKStnZ3RpdGxlKHBhc3RlKCJDbHVzdGVyIFg0IFByb2ZpbGUgIixjbHVzdGVyc2l6ZXNbNF0sIiBnZW5lcyIpKSsgIHNjYWxlX3hfZGlzY3JldGUobGltaXRzPW9yZGVyTikrCiAgdGhlbWUoYXhpcy50aXRsZS54ID0gZWxlbWVudF9ibGFuaygpLGF4aXMudGV4dC54ID0gZWxlbWVudF90ZXh0KGFuZ2xlID0gOTAsIHZqdXN0ID0gMC41LCBoanVzdD0xKSxheGlzLnRpdGxlLnkgPSBlbGVtZW50X2JsYW5rKCkpK3lsaW0gKC0xLjgsMS44KQojcFg1PC1nZ3Bsb3QoZGF0YT1jbHVzdGVyTWVhbnMxLCBhZXMoeD1TYW1wbGUsIHk9WDUsZ3JvdXA9MSkpICsKIyAgZ2VvbV9saW5lKGFlcyhncm91cCA9IFN0cmFpbikpKyAgZ2VvbV9wb2ludCgpK2dndGl0bGUocGFzdGUoIkNsdXN0ZXIgWDUgUHJvZmlsZSAiLGNsdXN0ZXJzaXplc1s1XSwiIGdlbmVzIikpKyAgc2NhbGVfeF9kaXNjcmV0ZShsaW1pdHM9b3JkZXJOKSsKIyAgdGhlbWUoYXhpcy50aXRsZS54ID0gZWxlbWVudF9ibGFuaygpLGF4aXMudGV4dC54ID0gZWxlbWVudF90ZXh0KGFuZ2xlID0gOTAsIHZqdXN0ID0gMC41LCBoanVzdD0xKSxheGlzLnRpdGxlLnkgPSBlbGVtZW50X2JsYW5rKCkpK3lsaW0gKC0xLjgsMS44KQojcFg4PC1nZ3Bsb3QoZGF0YT1jbHVzdGVyTWVhbnMxLCBhZXMoeD1TYW1wbGUsIHk9WDgsZ3JvdXA9MSkpICsKIyAgZ2VvbV9saW5lKCkrICBnZW9tX3BvaW50KCkrZ2d0aXRsZShwYXN0ZSgiQ2x1c3RlciBYOCBQcm9maWxlICIsY2x1c3RlcnNpemVzWzZdLCIgZ2VuZXMiKSkrICBzY2FsZV94X2Rpc2NyZXRlKGxpbWl0cz1vcmRlck4pKwojICB0aGVtZShheGlzLnRpdGxlLnggPSBlbGVtZW50X2JsYW5rKCksYXhpcy50aXRsZS55ID0gZWxlbWVudF9ibGFuaygpKQoKCiNwbG90Cm11bHRpcGxvdChwWDEscFgyLHBYMyxwWDQsICAgICBjb2xzPTIpCgpgYGAKCgpgYGB7cn0KdG9wREVnZW5lcyA8LSB3aGljaCh0ZW1wQSRJbmNsdWRlPT0iaW4iKSMjIyNmaW5kIGluZGV4ZXMKdGVtcEFrbTwtdGVtcEFbIHRvcERFZ2VuZXMsIF0KU3ltYm9sc0ttPC1kcGx5cjo6cHVsbCh0ZW1wQWttLCBHZW5lX1N5bWJvbCkKIyMjIyBleHBvcnQgdGhlIGdlbmUgZXhwcmVzc2lvbiBkYXRhIGZvciB0aGUgY2x1c3RlcnMKd3JpdGUudGFibGUoY2x1c3Rlck1lYW5zLHBhc3RlMCgiQ2x1c3Rlck1lYW5zS21fIixncm91cHNOYW1lLCIudHh0IiksICBzZXAgPSAiXHQiKQpDbHVzdGVyZWRHZW5lczwtZGF0YS5mcmFtZShrY2x1c3Q3JGNsdXN0ZXIsU3ltYm9sc0ttLGRhdGFITW0zKQp3cml0ZS50YWJsZShDbHVzdGVyZWRHZW5lcyxwYXN0ZTAoIlNjYWxlZERhdGFJbkNsdXN0ZXJzS21fIixncm91cHNOYW1lLCIudHh0IiksICBzZXAgPSAiXHQiKQojaGVhZChDbHVzdGVyZWRHZW5lcykKYGBgCgoKYGBge3J9CmJvdHRvbURFZ2VuZXM8LXdoaWNoKHRlbXBBJEluY2x1ZGU9PSJvdXQiKSMjIyNmaW5kIGluZGV4ZXMgCmJvdHRvbUc8LXRlbXBBWyBib3R0b21ERWdlbmVzLCBdCmJvdHRvbUc8LWRwbHlyOjpwdWxsKGJvdHRvbUcsIEdlbmVfU3ltYm9sKQp3cml0ZS50YWJsZShib3R0b21HLHBhc3RlMCgiaXBhQm90dG9tS21lYW5zXyIsZ3JvdXBzTmFtZSwiLnR4dCIpLCAgc2VwID0gIlx0IikKICAgICAgICAgICAgICAgICAgICAgICAgIAoKdG9wREVnZW5lcyA8LSB3aGljaCh0ZW1wQSRJbmNsdWRlPT0iaW4iKSMjIyNmaW5kIGluZGV4ZXMgCnRlbXBBa208LXRlbXBBWyB0b3BERWdlbmVzLCBdClN5bWJvbHNLbTwtZHBseXI6OnB1bGwodGVtcEFrbSwgR2VuZV9TeW1ib2wpCgppcGFLbWVhbnM8LUNsdXN0ZXJlZEdlbmVzCiNjb3VudHNUYWJsZSA8LWNvdW50c1RhYmxlWyxjKDE6MTUpXSMjIyNpZiBzYW1wbGVzIG5lZWQgcmVtb3ZpbmcKaXBhS21lYW5zPC1pcGFLbWVhbnNbLGMoMToyKV0KaXBhS21lYW5zJG5hbWUyPC1yb3duYW1lcyhpcGFLbWVhbnMpCiNpcGFLbWVhbnMlPiUgcm93bmFtZXNfdG9fY29sdW1uKHZhciA9ICJyb3duYW1lIikKI2lwYUttZWFucwojcm93aWRfdG9fY29sdW1uKGlwYUttZWFucykKaXBhS21lYW5zID0gbXV0YXRlKGlwYUttZWFucywgeDE9IGlmZWxzZShpcGFLbWVhbnMka2NsdXN0Ny5jbHVzdGVyPT0xLCAiMSIsICIwIikpCmlwYUttZWFucyA9IG11dGF0ZShpcGFLbWVhbnMsIHgyPSBpZmVsc2UoaXBhS21lYW5zJGtjbHVzdDcuY2x1c3Rlcj09MiwgIjEiLCAiMCIpKQppcGFLbWVhbnMgPSBtdXRhdGUoaXBhS21lYW5zLCB4Mz0gaWZlbHNlKGlwYUttZWFucyRrY2x1c3Q3LmNsdXN0ZXI9PTMsICIxIiwgIjAiKSkKaXBhS21lYW5zID0gbXV0YXRlKGlwYUttZWFucywgeDQ9IGlmZWxzZShpcGFLbWVhbnMka2NsdXN0Ny5jbHVzdGVyPT00LCAiMSIsICIwIikpCiNpcGFLbWVhbnMgPSBtdXRhdGUoaXBhS21lYW5zLCB4NT0gaWZlbHNlKGlwYUttZWFucyRrY2x1c3QzLmNsdXN0ZXI9PTUsICIxIiwgIjAiKSkKI2lwYUttZWFucyA9IG11dGF0ZShpcGFLbWVhbnMsIHg2PSBpZmVsc2UoaXBhS21lYW5zJGtjbHVzdDMuY2x1c3Rlcj09NiwgIjEiLCAiMCIpKQojaXBhS21lYW5zID0gbXV0YXRlKGlwYUttZWFucywgeDc9IGlmZWxzZShpcGFLbWVhbnMka2NsdXN0My5jbHVzdGVyPT03LCAiMSIsICIwIikpCiNpcGFLbWVhbnMKd3JpdGUudGFibGUoaXBhS21lYW5zLHBhc3RlMCgiaXBhS21lYW5zXyIsZ3JvdXBzTmFtZSwiLnR4dCIpLCAgc2VwID0gIlx0IikKI2hlYWQoaXBhS21lYW5zKQoKYGBgCgoKYGBge3J9CkNsdXN0ZXJlZEdlbmVzMjwtQ2x1c3RlcmVkR2VuZXNbYygxKV0KI0NsdXN0ZXJlZEdlbmVzMgpsaXN0QWxsPC1saXN0KCkKZm9yKGkgaW4gMTo0KSB7CiAgY2x1c3Rlck5hbWU8LXBhc3RlMCgieCIsaSkKICAjY2x1c3Rlck5hbWU8LXJvdy5uYW1lcyhzdWJzZXQoQ2x1c3RlcmVkR2VuZXMsQ2x1c3RlcmVkR2VuZXM9PWkpKQogIGNsdXN0ZXJOYW1lPC0oc3Vic2V0KENsdXN0ZXJlZEdlbmVzJFN5bWJvbHNLbSxDbHVzdGVyZWRHZW5lcz09aSkpCiAgbGlzdEFsbFtbaV1dPC1jbHVzdGVyTmFtZQp9CiNuZWVkIHRvIG5hbWUgdGhlIHZlY3RvcnMgaW4gdGhlIGxpc3QsIGV4YW1wbGUgaGVyZSBpcyBmb3IgOCBjbHVzdGVycwpuYW1lcyhsaXN0QWxsKTwtYygiWDEiLCAiWDIiLCAiWDMiLCAiWDQiKSMsIlg1IiwgIlg2IiwgIlg3IikKCiNpZiB5b3Ugd2FudCB0byByZWFycmFuZ2UgdGhlIG9yZGVyCiNsaXN0QWxsPC1saXN0QWxsW2MoIngzIiwgIng3IiwgIng4IiwgIngyIiwgIng2IiwgIng1IiwgIng0IiwgIngxIildCgpsYXBwbHkobGlzdEFsbCwgaGVhZCkKCmBgYAoKYGBge3J9CihzdWJzZXQoQ2x1c3RlcmVkR2VuZXMkU3ltYm9sc0ttLENsdXN0ZXJlZEdlbmVzPT0xKSkKYGBgCgoKIyMjI0VyaWNocgpgYGB7cn0Kc2V0RW5yaWNoclNpdGUoIkVucmljaHIiKSAjIEh1bWFuIGdlbmVzCmBgYApgYGB7cn0Kd2Vic2l0ZUxpdmUgPC0gVFJVRQpkYnMgPC0gbGlzdEVucmljaHJEYnMoKQppZiAoaXMubnVsbChkYnMpKSB3ZWJzaXRlTGl2ZSA8LSBGQUxTRQppZiAod2Vic2l0ZUxpdmUpIGhlYWQoZGJzKQpgYGAKCgoKCiMjIyM0LiBBbm5vdGF0aW9uIG9mIEstbWVhbnMgY2x1c3RlcnMKLSBDQyBjZWxsdWxhciBjb21wYXJ0bWVudAotIEJQIGJpb2xvZ2ljYWwgcHJvY2VzcwotIE1GIG1vbGVjdWxhciBmdW5jdGlvbgoKVGhlIHNpbXBsaWZ5IGZ1bmN0aW9uIGhhcyBiZWVuIHVzZWQgdG8gY3V0IGRvd24gb24gR08gcmVkdW5kYW5jeQoKYGBge3J9CiNzdHIoQWxsR2VuZU5hbWVzKQpgYGAKCgpgYGB7cixmaWcuaGVpZ2h0ID0gOCxmaWcud2lkdGggPSAxMn0KIyMjI0NDCmNnb0NDIDwtIGNvbXBhcmVDbHVzdGVyKGdlbmVDbHVzdGVyID0gbGlzdEFsbCwgCiAgICAgICAgICAgICAgICAgICAgICB1bml2ZXJzZSA9IEFsbEdlbmVOYW1lcywKICAgICAgICAgICAgICAgICAgICAgIGZ1biA9ICJlbnJpY2hHTyIsCiAgICAgICAgICAgICAgICAgICAgICBPcmdEYj1vcmcuSHMuZWcuZGIsIAogICAgICAgICAgICAgICAgICAgICAgIyMjI09yZ0RiPW9yZy5NbS5lZy5kYiwKICAgICAgICAgICAgICAgICAgICAgIGtleVR5cGU9IlNZTUJPTCIsCiAgICAgICAgICAgICAgICAgICAgICBvbnQgPSAiQ0MiLCAKICAgICAgICAgICAgICAgICAgICAgIHB2YWx1ZUN1dG9mZj0wLjA1LAogICAgICAgICAgICAgICAgICAgICAgcXZhbHVlQ3V0b2ZmID0gMC4xMCkKY2dvQ0MyIDwtIHNpbXBsaWZ5KGNnb0NDLCBjdXRvZmY9MC43LCBieT0icC5hZGp1c3QiLCBzZWxlY3RfZnVuPW1pbikKIyMjI3dyaXRlIGFzIHNwcmVhZHNoZWV0CndyaXRlLmNzdihhcy5kYXRhLmZyYW1lKGNnb0NDMikscGFzdGUwKCJHT19DQ18iLGdyb3Vwc05hbWUsIi5jc3YiKSkKCmRvdHBsb3QoY2dvQ0MyLHNob3dDYXRlZ29yeSA9IDMwLAogICAgICAgIHRpdGxlID0gcGFzdGUwKCJHTyBDZWxsdWxhciBDb21wYXJ0bWVudCAiLGdyb3Vwc05hbWUpKSsKICB0aGVtZShheGlzLnRleHQueCA9IGVsZW1lbnRfdGV4dChhbmdsZSA9IDkwLCB2anVzdCA9IDAuNSwgaGp1c3Q9MSkpCgpgYGAKUGxvdHMgYW5kIEdPIGRhdGEgd2VyZSB3cml0dGVuIHRvIGZpbGVzCmBgYHtyfQpwbmcocGFzdGUwKCJHT19DQ18iLGdyb3Vwc05hbWUsIi5wbmciKSwgd2lkdGggPSAxMjI0LCBoZWlnaHQgPSA4MjQpCmRvdHBsb3QoY2dvQ0MyLHNob3dDYXRlZ29yeSA9IDMwLAogICAgICAgIHRpdGxlID0gcGFzdGUwKCJHTyBDZWxsdWxhciBDb21wYXJ0bWVudCAiLGdyb3Vwc05hbWUpKSsKICB0aGVtZShheGlzLnRleHQueCA9IGVsZW1lbnRfdGV4dChhbmdsZSA9IDkwLCB2anVzdCA9IDAuNSwgaGp1c3Q9MSkpCmRldi5vZmYoKQpgYGAKCkdPIEJQCmBgYHtyLGZpZy5oZWlnaHQgPSAxMixmaWcud2lkdGggPSAxMn0KIyMjI0NDCmNnb0JQIDwtIGNvbXBhcmVDbHVzdGVyKGdlbmVDbHVzdGVyID0gbGlzdEFsbCwgCiAgICAgICAgICAgICAgICAgICAgICB1bml2ZXJzZSA9IEFsbEdlbmVOYW1lcywKICAgICAgICAgICAgICAgICAgICAgIGZ1biA9ICJlbnJpY2hHTyIsCiAgICAgICAgICAgICAgICAgICAgICBPcmdEYj1vcmcuSHMuZWcuZGIsCiAgICAgICAgICAgICAgICAgICAgICBrZXlUeXBlPSJTWU1CT0wiLAogICAgICAgICAgICAgICAgICAgICAgb250ID0gIkJQIiwgCiAgICAgICAgICAgICAgICAgICAgICBwdmFsdWVDdXRvZmY9MC4wNSwKICAgICAgICAgICAgICAgICAgICAgIHF2YWx1ZUN1dG9mZiA9IDAuMTApCmNnb0JQMiA8LSBzaW1wbGlmeShjZ29CUCwgY3V0b2ZmPTAuNywgYnk9InAuYWRqdXN0Iiwgc2VsZWN0X2Z1bj1taW4pCiMjIyN3cml0ZSBhcyBzcHJlYWRzaGVldAp3cml0ZS5jc3YoYXMuZGF0YS5mcmFtZShjZ29CUDIpLHBhc3RlMCgiR09fQlBfIixncm91cHNOYW1lLCIuY3N2IikpCgpkb3RwbG90KGNnb0JQMixzaG93Q2F0ZWdvcnkgPSAzMCwKICAgICAgICB0aXRsZSA9IHBhc3RlMCgiR08gQmlvbG9naWNhbCBQcm9jZXNzICIsZ3JvdXBzTmFtZSkpKwogIHRoZW1lKGF4aXMudGV4dC54ID0gZWxlbWVudF90ZXh0KGFuZ2xlID0gOTAsIHZqdXN0ID0gMC41LCBoanVzdD0xKSkKCmBgYAoKYGBge3J9CnBuZyhwYXN0ZTAoIkdPX0JQXyIsZ3JvdXBzTmFtZSwiLnBuZyIpLCB3aWR0aCA9IDEwMjQsIGhlaWdodCA9IDEyMjQpCmRvdHBsb3QoY2dvQlAyLHNob3dDYXRlZ29yeSA9IDMwLAogICAgICAgIHRpdGxlID0gcGFzdGUwKCJHTyBCaW9sb2dpY2FsIFByb2Nlc3MgIixncm91cHNOYW1lKSkrCiAgdGhlbWUoYXhpcy50ZXh0LnggPSBlbGVtZW50X3RleHQoYW5nbGUgPSA5MCwgdmp1c3QgPSAwLjUsIGhqdXN0PTEpKQpkZXYub2ZmKCkKYGBgCgoKR08gTUYKYGBge3IsZmlnLmhlaWdodCA9IDEwLGZpZy53aWR0aCA9IDEyfQojIyMjTUYKY2dvTUYgPC0gY29tcGFyZUNsdXN0ZXIoZ2VuZUNsdXN0ZXIgPSBsaXN0QWxsLCAKICAgICAgICAgICAgICAgICAgICAgIHVuaXZlcnNlID0gQWxsR2VuZU5hbWVzLAogICAgICAgICAgICAgICAgICAgICAgZnVuID0gImVucmljaEdPIiwKICAgICAgICAgICAgICAgICAgICAgIE9yZ0RiPW9yZy5Icy5lZy5kYiwgCiAgICAgICAgICAgICAgICAgICAgICBrZXlUeXBlPSJTWU1CT0wiLAogICAgICAgICAgICAgICAgICAgICAgb250ID0gIk1GIiwgCiAgICAgICAgICAgICAgICAgICAgICBwdmFsdWVDdXRvZmY9MC4wNSwKICAgICAgICAgICAgICAgICAgICAgIHF2YWx1ZUN1dG9mZiA9IDAuMTApCmNnb01GMiA8LSBzaW1wbGlmeShjZ29NRiwgY3V0b2ZmPTAuNywgYnk9InAuYWRqdXN0Iiwgc2VsZWN0X2Z1bj1taW4pCiMjIyN3cml0ZSBhcyBzcHJlYWRzaGVldAp3cml0ZS5jc3YoYXMuZGF0YS5mcmFtZShjZ29NRjIpLHBhc3RlMCgiR09fTUZfIixncm91cHNOYW1lLCIuY3N2IikpCgpkb3RwbG90KGNnb01GMixzaG93Q2F0ZWdvcnkgPSAzMCwKICAgICAgICB0aXRsZSA9IHBhc3RlMCgiR08gTW9sZWN1bGFyIEZ1bmN0aW9uICAiLGdyb3Vwc05hbWUpKSsKICB0aGVtZShheGlzLnRleHQueCA9IGVsZW1lbnRfdGV4dChhbmdsZSA9IDkwLCB2anVzdCA9IDAuNSwgaGp1c3Q9MSkpCgpgYGAKYGBge3IsZmlnLmhlaWdodCA9IDcsZmlnLndpZHRoID0gMTJ9CiNkYnMgPC0gYygiR09fTW9sZWN1bGFyX0Z1bmN0aW9uXzIwMTgiLCAiR09fQ2VsbHVsYXJfQ29tcG9uZW50XzIwMTgiLCAiR09fQmlvbG9naWNhbF9Qcm9jZXNzXzIwMTgiKQpkYnMgPC0gYygiUmVhY3RvbWVfMjAxNiIsIldpa2lQYXRod2F5c18yMDE5X01vdXNlIikKCmlmICh3ZWJzaXRlTGl2ZSkgeyAgICBlbnJpY2hlZDEgPC0gZW5yaWNocigoc3Vic2V0KENsdXN0ZXJlZEdlbmVzJFN5bWJvbHNLbSxDbHVzdGVyZWRHZW5lcz09MSkpLCBkYnMpfQppZiAod2Vic2l0ZUxpdmUpIHBsb3RFbnJpY2goZW5yaWNoZWQxW1sxXV0sIHNob3dUZXJtcyA9IDMwLCBudW1DaGFyID0gODAsIHkgPSAiQ291bnQiLCBvcmRlckJ5ID0gIlAudmFsdWUiLCB0aXRsZSA9IlJlYWN0b21lIEVucmljaG1lbnQgQW5hbHlzaXMgQ2x1c3RlciAxIikKaWYgKHdlYnNpdGVMaXZlKSB7ICAgIGVucmljaGVkMiA8LSBlbnJpY2hyKChzdWJzZXQoQ2x1c3RlcmVkR2VuZXMkU3ltYm9sc0ttLENsdXN0ZXJlZEdlbmVzPT0yKSksIGRicyl9CmlmICh3ZWJzaXRlTGl2ZSkgcGxvdEVucmljaChlbnJpY2hlZDJbWzFdXSwgc2hvd1Rlcm1zID0gMzAsIG51bUNoYXIgPSA4MCwgeSA9ICJDb3VudCIsIG9yZGVyQnkgPSAiUC52YWx1ZSIsIHRpdGxlID0iUmVhY3RvbWUgRW5yaWNobWVudCBBbmFseXNpcyBDbHVzdGVyIDIiKQppZiAod2Vic2l0ZUxpdmUpIHsgICAgZW5yaWNoZWQzIDwtIGVucmljaHIoKHN1YnNldChDbHVzdGVyZWRHZW5lcyRTeW1ib2xzS20sQ2x1c3RlcmVkR2VuZXM9PTMpKSwgZGJzKX0KaWYgKHdlYnNpdGVMaXZlKSBwbG90RW5yaWNoKGVucmljaGVkM1tbMV1dLCBzaG93VGVybXMgPSAzMCwgbnVtQ2hhciA9IDgwLCB5ID0gIkNvdW50Iiwgb3JkZXJCeSA9ICJQLnZhbHVlIiwgdGl0bGUgPSJSZWFjdG9tZSBFbnJpY2htZW50IEFuYWx5c2lzIENsdXN0ZXIgMyIpCmlmICh3ZWJzaXRlTGl2ZSkgeyAgICBlbnJpY2hlZDQgPC0gZW5yaWNocigoc3Vic2V0KENsdXN0ZXJlZEdlbmVzJFN5bWJvbHNLbSxDbHVzdGVyZWRHZW5lcz09NCkpLCBkYnMpfQppZiAod2Vic2l0ZUxpdmUpIHBsb3RFbnJpY2goZW5yaWNoZWQ0W1sxXV0sIHNob3dUZXJtcyA9IDMwLCBudW1DaGFyID0gODAsIHkgPSAiQ291bnQiLCBvcmRlckJ5ID0gIlAudmFsdWUiLCB0aXRsZSA9IlJlYWN0b21lIEVucmljaG1lbnQgQW5hbHlzaXMgQ2x1c3RlciA0IikKCmlmICh3ZWJzaXRlTGl2ZSkgcGxvdEVucmljaChlbnJpY2hlZDFbWzJdXSwgc2hvd1Rlcm1zID0gMzAsIG51bUNoYXIgPSA4MCwgeSA9ICJDb3VudCIsIG9yZGVyQnkgPSAiUC52YWx1ZSIsIHRpdGxlID0iV2lraVBhdGh3YXlzIEVucmljaG1lbnQgQW5hbHlzaXMgQ2x1c3RlciAxIikKaWYgKHdlYnNpdGVMaXZlKSBwbG90RW5yaWNoKGVucmljaGVkMltbMl1dLCBzaG93VGVybXMgPSAzMCwgbnVtQ2hhciA9IDgwLCB5ID0gIkNvdW50Iiwgb3JkZXJCeSA9ICJQLnZhbHVlIiwgdGl0bGUgPSJXaWtpUGF0aHdheXMgRW5yaWNobWVudCBBbmFseXNpcyBDbHVzdGVyIDIiKQppZiAod2Vic2l0ZUxpdmUpIHBsb3RFbnJpY2goZW5yaWNoZWQzW1syXV0sIHNob3dUZXJtcyA9IDMwLCBudW1DaGFyID0gODAsIHkgPSAiQ291bnQiLCBvcmRlckJ5ID0gIlAudmFsdWUiLCB0aXRsZSA9Ildpa2lQYXRod2F5cyBFbnJpY2htZW50IEFuYWx5c2lzIENsdXN0ZXIgMyIpCmlmICh3ZWJzaXRlTGl2ZSkgcGxvdEVucmljaChlbnJpY2hlZDRbWzJdXSwgc2hvd1Rlcm1zID0gMzAsIG51bUNoYXIgPSA4MCwgeSA9ICJDb3VudCIsIG9yZGVyQnkgPSAiUC52YWx1ZSIsIHRpdGxlID0iV2lraVBhdGh3YXlzIEVucmljaG1lbnQgQW5hbHlzaXMgQ2x1c3RlciA0IikKYGBgCgoKYGBge3J9CnBuZyhwYXN0ZTAoIkdPX01GXyIsZ3JvdXBzTmFtZSwiLnBuZyIpLCB3aWR0aCA9IDE0MjQsIGhlaWdodCA9IDgyNCkKZG90cGxvdChjZ29NRjIsc2hvd0NhdGVnb3J5ID0gMzAsCiAgICAgICAgdGl0bGUgPSBwYXN0ZTAoIkdPIE1vbGVjdWxhciBGdW5jdGlvbiAgIixncm91cHNOYW1lKSkrCiAgdGhlbWUoYXhpcy50ZXh0LnggPSBlbGVtZW50X3RleHQoYW5nbGUgPSA5MCwgdmp1c3QgPSAwLjUsIGhqdXN0PTEpKQpkZXYub2ZmKCkKYGBgCgoKCiMjIyBSMSAmIFI0IFZBUjM3IFZBUjE0IFRORiBrLW1lYW5zIHAwLjA1IGxmYyAxCgohW0Rlc2lnbl0oZGVzaWduLnBuZykKCiMjIyMxLiBHZW5lbGlzdCBTZWxlY3Rpb24KCmBgYHtyfQpncm91cHNOYW1lPC0iUjFfUjRfa21lYW5zX3AwLjA1bGZjMSIKYGBgCgoKYGBge3J9CmNvdW50c1RhYmxlPC1yZWFkLmRlbGltKCJSTkFzZXEyMDE5SnVseV81LnR4dCIsIGhlYWRlciA9IFRSVUUsIHNlcCA9ICJcdCIsY2hlY2submFtZXM9RkFMU0Uscm93Lm5hbWVzPTEpCmhlYWQoY291bnRzVGFibGUpCmBgYAoKYGBge3J9CkFsbEdlbmVOYW1lczwtY291bnRzVGFibGUkR2VuZV9TeW1ib2wKI2hlYWQoQWxsR2VuZU5hbWVzKQpgYGAKCmBgYHtyfQp0ZW1wQTwtY291bnRzVGFibGUKYGBgCgpgYGB7cn0KI3RlbXBBPC1yZXNBbGxbLWMoMTA6MzApIF0KdGVtcEE8LWNvdW50c1RhYmxlCiNyb3duYW1lcyh0ZW1wQSkKcm93bmFtZXModGVtcEEpIDwtIE5VTEwKdGVtcEEgPSBtdXRhdGUodGVtcEEsIEluY2x1ZGU9CiAgICAgICAgICAgICAgICAgICBpZmVsc2UodGVtcEEkcHZhbHVlX1IxX1ZhcjM3X0hvdXJzXzZoX3ZzXzBoPDAuMDUmYWJzKHRlbXBBJGxvZzJGb2xkQ2hhbmdlX1IxX1ZhcjM3X0hvdXJzXzZoX3ZzXzBoKT4xJigodGVtcEEkVmFyMzdUTkZfMGhfbWVhbj4xMCl8KHRlbXBBJFZhcjM3VE5GXzZoX21lYW4+MTApKSYhaXMubmEodGVtcEEkcHZhbHVlX1IxX1ZhcjM3X0hvdXJzXzZoX3ZzXzBoKSwgImluIiwKICAgICAgICAgICAgICAgICAgICAgICAgICBpZmVsc2UodGVtcEEkcHZhbHVlX1IxX1ZhcjM3X0hvdXJzXzIwaF92c18waDwwLjA1JmFicyh0ZW1wQSRsb2cyRm9sZENoYW5nZV9SMV9WYXIzN19Ib3Vyc18yMGhfdnNfMGgpPjEmKCh0ZW1wQSRWYXIzN1RORl8waF9tZWFuPjEwKXwodGVtcEEkVmFyMzdUTkZfMjBoX21lYW4+MTApKSYhaXMubmEodGVtcEEkcHZhbHVlX1IxX1ZhcjM3X0hvdXJzXzIwaF92c18waCksICJpbiIsCiAgICAgICAgICAgICAgICAgICAgICAgICAgICAgICAgIGlmZWxzZSh0ZW1wQSRwdmFsdWVfUjFfVmFyMzdfSG91cnNfMjBoX3ZzXzZoPDAuMDUmYWJzKHRlbXBBJGxvZzJGb2xkQ2hhbmdlX1IxX1ZhcjM3X0hvdXJzXzIwaF92c182aCk+MSYoKHRlbXBBJFZhcjM3VE5GXzZoX21lYW4+MTApfCh0ZW1wQSRWYXIzN1RORl8yMGhfbWVhbj4xMCkpJiFpcy5uYSh0ZW1wQSRwdmFsdWVfUjFfVmFyMzdfSG91cnNfMjBoX3ZzXzZoKSwgImluIiwKICAgICAgICAgICAgICAgICAgICAgICAgICAgICAgICAgICAgICAgIGlmZWxzZSh0ZW1wQSRwdmFsdWVfUjRWYXIxNFRORl9Ib3Vyc182aF92c18waDwwLjA1JmFicyh0ZW1wQSRsb2cyRm9sZENoYW5nZV9SNFZhcjE0VE5GX0hvdXJzXzZoX3ZzXzBoKT4xJigodGVtcEEkVmFyMTRUTkZfMGhfbWVhbj4xMCl8KHRlbXBBJFZhcjE0VE5GXzZoX21lYW4+MTApKSYhaXMubmEodGVtcEEkcHZhbHVlX1I0VmFyMTRUTkZfSG91cnNfNmhfdnNfMGgpLCAiaW4iLAogICAgICAgICAgICAgICAgICAgICAgICAgICAgICAgICAgICAgICAgICAgICAgIGlmZWxzZSh0ZW1wQSRwdmFsdWVfUjRWYXIxNFRORl9Ib3Vyc18yMGhfdnNfMGg8MC4wNSZhYnModGVtcEEkbG9nMkZvbGRDaGFuZ2VfUjRWYXIxNFRORl9Ib3Vyc18yMGhfdnNfMGgpPjEmKCh0ZW1wQSRWYXIxNFRORl8waF9tZWFuPjEwKXwodGVtcEEkVmFyMTRUTkZfMjBoX21lYW4+MTApKSYhaXMubmEodGVtcEEkcHZhbHVlX1I0VmFyMTRUTkZfSG91cnNfMjBoX3ZzXzBoKSwiaW4iLAogICAgICAgICAgICAgICAgICAgICAgICAgICAgICAgICAgICAgICAgICAgICAgICAgICAgICBpZmVsc2UodGVtcEEkcHZhbHVlX1I0VmFyMTRUTkZfSG91cnNfMjBoX3ZzXzZoPDAuMDUmYWJzKHRlbXBBJGxvZzJGb2xkQ2hhbmdlX1I0VmFyMTRUTkZfSG91cnNfMjBoX3ZzXzZoKT4xJigodGVtcEEkVmFyMTRUTkZfMjBoX21lYW4+MTApfCh0ZW1wQSRWYXIxNFRORl82aF9tZWFuPjEwKSkmIWlzLm5hKHRlbXBBJHB2YWx1ZV9SNFZhcjE0VE5GX0hvdXJzXzIwaF92c182aCksImluIiwKICAgICAgICAgICAgICAgICAgICAgICAgICAgICAgICAgICAgICAgICAgICAgICAgICJvdXQiKSkpKSkpKQoKCiN0ZW1wQQojIyMjbGlicmFyeShkcGx5cikKdGVtcEEgJT4lCiAgICAgZ3JvdXBfYnkoSW5jbHVkZSkgJT4lIAogICAgIHRhbGx5KCkKYGBgCgpgYGB7cn0KdG9wREVnZW5lcyA8LSB3aGljaCh0ZW1wQSRJbmNsdWRlPT0iaW4iKSMjIyNmaW5kIGluZGV4ZXMgCmBgYAoKYGBge3J9CmhlYWQoY291bnRzVGFibGUpCmBgYAoKYGBge3J9CmJhc2VNZWFuc0htIDwtY291bnRzVGFibGVbLGMoNDg6NTAsMTEwLDExMiwxMTMpXQpoZWFkKGJhc2VNZWFuc0htKQpgYGAKCiMjIyMgTkIgUGxlYXNlIGNoZWNrIGNvbHVtbnMgdXNlZCBhbmQgcmVuYW1lZCBmb3IgcGxvdHMKYGBge3J9CiNiYXNlTWVhbnNIbSA8LWNvdW50c1RhYmxlWyxjKDYwOjYzKV0KYmFzZU1lYW5zSG0gPC1jb3VudHNUYWJsZVssYyg0ODo1MCwxMTAsMTEyLDExMyldCgpoZWFkKGJhc2VNZWFuc0htKQoKCnRhaWwoYmFzZU1lYW5zSG1bIHRvcERFZ2VuZXMsIF0pCgoKYmFzZU1lYW5zSG0kVmFyMzdUTkZfMGg8LWJhc2VNZWFuc0htJFZhcjM3VE5GXzBoX21lYW4KYmFzZU1lYW5zSG0kVmFyMzdUTkZfNmg8LWJhc2VNZWFuc0htJFZhcjM3VE5GXzZoX21lYW4KYmFzZU1lYW5zSG0kVmFyMzdUTkZfMjBoPC1iYXNlTWVhbnNIbSRWYXIzN1RORl8yMGhfbWVhbgoKYmFzZU1lYW5zSG0kVmFyMTRUTkZfMGg8LWJhc2VNZWFuc0htJFZhcjE0VE5GXzBoX21lYW4KYmFzZU1lYW5zSG0kVmFyMTRUTkZfNmg8LWJhc2VNZWFuc0htJFZhcjE0VE5GXzZoX21lYW4KYmFzZU1lYW5zSG0kVmFyMTRUTkZfMjBoPC1iYXNlTWVhbnNIbSRWYXIxNFRORl8yMGhfbWVhbgoKYmFzZU1lYW5zSG0gPC1iYXNlTWVhbnNIbVssYyg3OjEyKV0KCiNyZXBsYWNlIGxvdyB2YWx1ZXMgd2l0aCAwCmJhc2VNZWFuc0htJFZhcjM3VE5GXzBoW2Jhc2VNZWFuc0htJFZhcjM3VE5GXzBoPDEwXTwtMApiYXNlTWVhbnNIbSRWYXIzN1RORl82aFtiYXNlTWVhbnNIbSRWYXIzN1RORl82aDwxMF08LTAKYmFzZU1lYW5zSG0kVmFyMzdUTkZfMjBoW2Jhc2VNZWFuc0htJFZhcjM3VE5GXzIwaDwxMF08LTAKYmFzZU1lYW5zSG0kVmFyMTRUTkZfMGhbYmFzZU1lYW5zSG0kVmFyMTRUTkZfMGg8MTBdPC0wCmJhc2VNZWFuc0htJFZhcjE0VE5GXzZoW2Jhc2VNZWFuc0htJFZhcjE0VE5GXzZoPDEwXTwtMApiYXNlTWVhbnNIbSRWYXIxNFRORl8yMGhbYmFzZU1lYW5zSG0kVmFyMTRUTkZfMjBoPDEwXTwtMAp0YWlsKGJhc2VNZWFuc0htKQpiYXNlTWVhbnNIbSA8LSBsb2cyKGJhc2VNZWFuc0htKzEpCnRhaWwoYmFzZU1lYW5zSG0pCiNiYXNlTWVhbnNIbU0gPC1iYXNlTWVhbnNIbTJbLGMoMTo4KV0KI2hlYWQoYmFzZU1lYW5zSG1NKQpgYGAKCmBgYHtyfQp0b3BERWdlbmVzIDwtIHdoaWNoKHRlbXBBJEluY2x1ZGU9PSJpbiIpIyMjI2ZpbmQgaW5kZXhlcyAKYGBgCgpgYGB7cn0KI3NjYWxlIFZhcjM1IGFuZCBWYXIxNCBzZXBhcmF0ZWx5CnZhcjE0bW48LWJhc2VNZWFuc0htWyxjKDQ6NildCnZhcjE0bW48LSB0KGFzLm1hdHJpeCh2YXIxNG1uKSkKdmFyMTRtbiA8LSB0KHNjYWxlKHZhcjE0bW4pKQojaGVhZCh2YXIxNG1uKQpiYXNlTWVhbnNIbTI8LWJhc2VNZWFuc0htWyxjKDE6MyldCmJhc2VNZWFuc0htMjwtIHQoYXMubWF0cml4KGJhc2VNZWFuc0htMikpCmJhc2VNZWFuc0htMiA8LSB0KHNjYWxlKGJhc2VNZWFuc0htMikpCmJhc2VNZWFuc0htMiA8LSBhcy5kYXRhLmZyYW1lKGNiaW5kKGJhc2VNZWFuc0htMiwgdmFyMTRtbikpCmJhc2VNZWFuc0htMltpcy5uYShiYXNlTWVhbnNIbTIpXSA8LSAwCiNoZWFkKGJhc2VNZWFuc0htMikKCmJhc2VNZWFuc0htMiRWYXIzN1RORl8waF9sZmM8LWJhc2VNZWFuc0htMiRWYXIzN1RORl8waC1iYXNlTWVhbnNIbTIkVmFyMzdUTkZfMGgKYmFzZU1lYW5zSG0yJFZhcjM3VE5GXzZoX2xmYzwtYmFzZU1lYW5zSG0yJFZhcjM3VE5GXzZoLWJhc2VNZWFuc0htMiRWYXIzN1RORl8waApiYXNlTWVhbnNIbTIkVmFyMzdUTkZfMjBoX2xmYzwtYmFzZU1lYW5zSG0yJFZhcjM3VE5GXzIwaC1iYXNlTWVhbnNIbTIkVmFyMzdUTkZfMGgKYmFzZU1lYW5zSG0yJFZhcjE0VE5GXzBoX2xmYzwtYmFzZU1lYW5zSG0yJFZhcjE0VE5GXzBoLWJhc2VNZWFuc0htMiRWYXIxNFRORl8waApiYXNlTWVhbnNIbTIkVmFyMTRUTkZfNmhfbGZjPC1iYXNlTWVhbnNIbTIkVmFyMTRUTkZfNmgtYmFzZU1lYW5zSG0yJFZhcjE0VE5GXzBoCmJhc2VNZWFuc0htMiRWYXIxNFRORl8yMGhfbGZjPC1iYXNlTWVhbnNIbTIkVmFyMTRUTkZfMjBoLWJhc2VNZWFuc0htMiRWYXIxNFRORl8waAojYmFzZU1lYW5zSG0xPC1iYXNlTWVhbnNIbTJbLGMoMTo2KV0KYmFzZU1lYW5zSG0zPC1iYXNlTWVhbnNIbTJbLGMoNzoxMildCmhlYWQoYmFzZU1lYW5zSG0zKQpiYXNlTWVhbnNIbTI8LWJhc2VNZWFuc0htMlssYygxOjYpXQpoZWFkKGJhc2VNZWFuc0htMikKCmBgYAoKYGBge3J9CmRhdGFITW0yPC1hcy5tYXRyaXgoYmFzZU1lYW5zSG0yWyB0b3BERWdlbmVzLCBdKQp0YWlsKGRhdGFITW0yKQpgYGAKCgojIyMjMi4gSGllcmFjaGljYWwgY2x1c3RlcmluZyBvZiBtZWFucyAoaW5kaXZpZHVhbCBzYW1wbGVzIGFkZGVkIGZvciBpbnNwZWN0aW9uKQpgYGB7cixmaWcuaGVpZ2h0ID0gMTIsZmlnLndpZHRoID0gMjB9CiMjIyNtZWFuCgpkYXRhSE1tMjwtYXMubWF0cml4KGJhc2VNZWFuc0htMlsgdG9wREVnZW5lcywgXSkKZGF0YUhNbTJfMzc8LWRhdGFITW0yWyxjKDEsMiwzKV0KZGF0YUhNbTJfMTQ8LWRhdGFITW0yWyxjKDQsNSw2KV0KCmhtYXBfaGllcl9mYWN0b3JzMzcgPC0gSGVhdG1hcCgKICBkYXRhSE1tMl8zNywgIG5hbWUgPSAibWVhbjM3IiwKICByb3dfbGFiZWxzID0gcGFzdGUwKHJvd25hbWVzKGRhdGFITW0yXzM3KSwiICIsKHRlbXBBWyB0b3BERWdlbmVzLCBdKSRHZW5lX1N5bWJvbCksCiAgY29sdW1uX3RpdGxlID0gcGFzdGUwKCJNZWFuc1YzNyIpLCAKICBjb2wgPSBjb2xfZnVuR1IsCiAgY29sdW1uX3RpdGxlX2dwID0gZ3Bhcihmb250c2l6ZSA9IDE0LCBmb250ZmFjZSA9ICJib2xkIiksCiAgd2lkdGggPSB1bml0KDI1LCAibW0iKSwKICBjbHVzdGVyX2NvbHVtbnMgPSBGQUxTRSwKICAjY2x1c3Rlcl9yb3dzID0gRkFMU0UsCiAgc2hvd19yb3dfbmFtZXMgPSBGQUxTRSkKCmhtYXBfaGllcl9mYWN0b3JzMzdiIDwtIEhlYXRtYXAoCiAgZGF0YUhNbTJfMzcsICBuYW1lID0gIm1lYW4zN2IiLAogIHJvd19sYWJlbHMgPSBwYXN0ZTAocm93bmFtZXMoZGF0YUhNbTJfMzcpLCIgIiwodGVtcEFbIHRvcERFZ2VuZXMsIF0pJEdlbmVfU3ltYm9sKSwKICBjb2x1bW5fdGl0bGUgPSBwYXN0ZTAoIk1lYW5zVjM3IiksIAogIGNvbCA9IGNvbF9mdW5HUiwKICBjb2x1bW5fdGl0bGVfZ3AgPSBncGFyKGZvbnRzaXplID0gMTQsIGZvbnRmYWNlID0gImJvbGQiKSwKICB3aWR0aCA9IHVuaXQoMjUsICJtbSIpLAogICNjbHVzdGVyX2NvbHVtbnMgPSBGQUxTRSwKICAjY2x1c3Rlcl9yb3dzID0gRkFMU0UsCiAgc2hvd19yb3dfbmFtZXMgPSBGQUxTRSkKCmhtYXBfaGllcl9mYWN0b3JzMTQgPC0gSGVhdG1hcCgKICBkYXRhSE1tMl8xNCwgIG5hbWUgPSAibWVhbjE0IiwKICByb3dfbGFiZWxzID0gcGFzdGUwKHJvd25hbWVzKGRhdGFITW0yXzE0KSwiICIsKHRlbXBBWyB0b3BERWdlbmVzLCBdKSRHZW5lX1N5bWJvbCksCiAgY29sdW1uX3RpdGxlID0gcGFzdGUwKCJNZWFuc1YxNCIpLCAKICBjb2wgPSBjb2xfZnVuR1IsCiAgY29sdW1uX3RpdGxlX2dwID0gZ3Bhcihmb250c2l6ZSA9IDE0LCBmb250ZmFjZSA9ICJib2xkIiksCiAgd2lkdGggPSB1bml0KDI1LCAibW0iKSwKICBjbHVzdGVyX2NvbHVtbnMgPSBGQUxTRSwKICAjY2x1c3Rlcl9yb3dzID0gRkFMU0UsCiAgc2hvd19yb3dfbmFtZXMgPSBGQUxTRSkKCmhtYXBfaGllcl9mYWN0b3JzMTRiIDwtIEhlYXRtYXAoCiAgZGF0YUhNbTJfMTQsICBuYW1lID0gIm1lYW4xNGIiLAogIHJvd19sYWJlbHMgPSBwYXN0ZTAocm93bmFtZXMoZGF0YUhNbTJfMTQpLCIgIiwodGVtcEFbIHRvcERFZ2VuZXMsIF0pJEdlbmVfU3ltYm9sKSwKICBjb2x1bW5fdGl0bGUgPSBwYXN0ZTAoIk1lYW5zVjE0IiksIAogIGNvbCA9IGNvbF9mdW5HUiwKICBjb2x1bW5fdGl0bGVfZ3AgPSBncGFyKGZvbnRzaXplID0gMTQsIGZvbnRmYWNlID0gImJvbGQiKSwKICB3aWR0aCA9IHVuaXQoMjUsICJtbSIpLAogICNjbHVzdGVyX2NvbHVtbnMgPSBGQUxTRSwKICAjY2x1c3Rlcl9yb3dzID0gRkFMU0UsCiAgc2hvd19yb3dfbmFtZXMgPSBGQUxTRSkKCiN3cml0ZS50YWJsZShkYXRhSE1tMiwiZGF0YUhNbTIudHh0IiwgIHNlcCA9ICJcdCIpCgpobWFwX2hpZXJfZmFjdG9yczQgPC0gSGVhdG1hcCgKICBkYXRhSE1tMiwgIG5hbWUgPSAibWVhbjEiLAogIHJvd19sYWJlbHMgPSBwYXN0ZTAocm93bmFtZXMoZGF0YUhNbTIpLCIgIiwodGVtcEFbIHRvcERFZ2VuZXMsIF0pJEdlbmVfU3ltYm9sKSwKICBjb2x1bW5fdGl0bGUgPSBwYXN0ZTAoIk1lYW5zIiksIAogIGNvbCA9IGNvbF9mdW5HUiwKICBjb2x1bW5fdGl0bGVfZ3AgPSBncGFyKGZvbnRzaXplID0gMTQsIGZvbnRmYWNlID0gImJvbGQiKSwKICB3aWR0aCA9IHVuaXQoNTAsICJtbSIpLAogIGNsdXN0ZXJfY29sdW1ucyA9IEZBTFNFLAogICNjbHVzdGVyX3Jvd3MgPSBGQUxTRSwKICBzaG93X3Jvd19uYW1lcyA9IEZBTFNFKQoKZGF0YUhNbTJiPC1kYXRhSE1tMlssYygxLDQsMiw1LDMsNildCmhtYXBfaGllcl9mYWN0b3JzNGEgPC0gSGVhdG1hcCgKICBkYXRhSE1tMmIsICBuYW1lID0gIm1lYW4yIiwKICByb3dfbGFiZWxzID0gcGFzdGUwKHJvd25hbWVzKGRhdGFITW0yYiksIiAiLCh0ZW1wQVsgdG9wREVnZW5lcywgXSkkR2VuZV9TeW1ib2wpLAogIGNvbHVtbl90aXRsZSA9IHBhc3RlMCgiTWVhbnMgUmVhcnJhbmdlZCIpLCAKICBjb2wgPSBjb2xfZnVuR1IsCiAgY29sdW1uX3RpdGxlX2dwID0gZ3Bhcihmb250c2l6ZSA9IDE0LCBmb250ZmFjZSA9ICJib2xkIiksCiAgd2lkdGggPSB1bml0KDUwLCAibW0iKSwKICBjbHVzdGVyX2NvbHVtbnMgPSBGQUxTRSwKICAjY2x1c3Rlcl9yb3dzID0gRkFMU0UsCiAgc2hvd19yb3dfbmFtZXMgPSBGQUxTRSkKCmhtYXBfaGllcl9mYWN0b3JzNGIgPC0gSGVhdG1hcCgKICBkYXRhSE1tMiwgIG5hbWUgPSAibWVhbjMiLAogIHJvd19sYWJlbHMgPSBwYXN0ZTAocm93bmFtZXMoZGF0YUhNbTIpLCIgIiwodGVtcEFbIHRvcERFZ2VuZXMsIF0pJEdlbmVfU3ltYm9sKSwKICBjb2x1bW5fdGl0bGUgPSBwYXN0ZTAoIk1lYW5zIENsdXN0ZXJlZCIpLCAKICBjb2wgPSBjb2xfZnVuR1IsCiAgY29sdW1uX3RpdGxlX2dwID0gZ3Bhcihmb250c2l6ZSA9IDE0LCBmb250ZmFjZSA9ICJib2xkIiksCiAgd2lkdGggPSB1bml0KDUwLCAibW0iKSwKICAjY2x1c3Rlcl9jb2x1bW5zID0gRkFMU0UsCiAgI2NsdXN0ZXJfcm93cyA9IEZBTFNFLAogIHNob3dfcm93X25hbWVzID0gRkFMU0UpCgpkYXRhSE1tMzwtYXMubWF0cml4KGJhc2VNZWFuc0htM1sgdG9wREVnZW5lcywgXSkKd3JpdGUudGFibGUoZGF0YUhNbTMsImRhdGFITW0zLnR4dCIsICBzZXAgPSAiXHQiKQojYmFzZU1lYW5zSG0yPC1hcy5tYXRyaXgoYmFzZU1lYW5zSG0yKQogIApobWFwX2hpZXJfZmFjdG9yczYgPC0gSGVhdG1hcCgKICBkYXRhSE1tMywgIG5hbWUgPSAibG9nZmMxIiwKICByb3dfbGFiZWxzID0gcGFzdGUwKHJvd25hbWVzKGRhdGFITW0zKSwiICIsKHRlbXBBWyB0b3BERWdlbmVzLCBdKSRHZW5lX1N5bWJvbCksCiAgY29sdW1uX3RpdGxlID0gcGFzdGUwKCJ2cyAwaCIpLCAKICBjb2wgPSBjb2xfZnVuR1IyLAogIGNvbHVtbl90aXRsZV9ncCA9IGdwYXIoZm9udHNpemUgPSAxNCwgZm9udGZhY2UgPSAiYm9sZCIpLAogIHdpZHRoID0gdW5pdCg1MCwgIm1tIiksCiAgY2x1c3Rlcl9jb2x1bW5zID0gRkFMU0UsCiAgc2hvd19yb3dfbmFtZXMgPSBGQUxTRSkKCmRhdGFITW0zYjwtZGF0YUhNbTNbLGMoMSw0LDIsNSwzLDYpXQpobWFwX2hpZXJfZmFjdG9yczZiIDwtIEhlYXRtYXAoCiAgZGF0YUhNbTNiLCAgbmFtZSA9ICJsb2dmYzIiLAogIHJvd19sYWJlbHMgPSBwYXN0ZTAocm93bmFtZXMoZGF0YUhNbTNiKSwiICIsKHRlbXBBWyB0b3BERWdlbmVzLCBdKSRHZW5lX1N5bWJvbCksCiAgY29sdW1uX3RpdGxlID0gcGFzdGUwKCJ2cyAwaCBSZWFycmFuZ2VkIiksIAogIGNvbCA9IGNvbF9mdW5HUjIsCiAgY29sdW1uX3RpdGxlX2dwID0gZ3Bhcihmb250c2l6ZSA9IDE0LCBmb250ZmFjZSA9ICJib2xkIiksCiAgd2lkdGggPSB1bml0KDUwLCAibW0iKSwKICBjbHVzdGVyX2NvbHVtbnMgPSBGQUxTRSwKICBzaG93X3Jvd19uYW1lcyA9IEZBTFNFKQoKaG1hcF9oaWVyX2ZhY3RvcnM2YyA8LSBIZWF0bWFwKAogIGRhdGFITW0zLCAgbmFtZSA9ICJsb2dmYzMiLAogIHJvd19sYWJlbHMgPSBwYXN0ZTAocm93bmFtZXMoZGF0YUhNbTMpLCIgIiwodGVtcEFbIHRvcERFZ2VuZXMsIF0pJEdlbmVfU3ltYm9sKSwKICBjb2x1bW5fdGl0bGUgPSBwYXN0ZTAoInZzIDBoIiksIAogIGNvbCA9IGNvbF9mdW5HUjIsCiAgY29sdW1uX3RpdGxlX2dwID0gZ3Bhcihmb250c2l6ZSA9IDE0LCBmb250ZmFjZSA9ICJib2xkIiksCiAgd2lkdGggPSB1bml0KDUwLCAibW0iKSwKICAjY2x1c3Rlcl9jb2x1bW5zID0gRkFMU0UsCiAgc2hvd19yb3dfbmFtZXMgPSBGQUxTRSkKCmhtbGlzdDE9aG1hcF9oaWVyX2ZhY3RvcnMzNytobWFwX2hpZXJfZmFjdG9yczE0K2htYXBfaGllcl9mYWN0b3JzMzdiK2htYXBfaGllcl9mYWN0b3JzMTRiCmRyYXcoaG1saXN0MSwgY29sdW1uX3RpdGxlID0gIkhlYXRtYXBzIG9uIE1lYW5zIChzY2FsZWQgcGVyIHN0cmFpbikuIEdlbmVsaXN0cyBjb21iaW5lZCBmcm9tIFZBUjM3IGFuZCBWQVIxNCB0aW1lY291cnNlcyBwPDAuMDUgbGZjMSIsIGNvbHVtbl90aXRsZV9ncCA9IGdwYXIoZm9udHNpemUgPSAyMikpCgpobWxpc3QyPWhtYXBfaGllcl9mYWN0b3JzNCtobWFwX2hpZXJfZmFjdG9yczRhK2htYXBfaGllcl9mYWN0b3JzNGIKZHJhdyhobWxpc3QyLCBjb2x1bW5fdGl0bGUgPSAiSGVhdG1hcHMgb24gTWVhbnMgKHNjYWxlZCBwZXIgc3RyYWluKSIsIGNvbHVtbl90aXRsZV9ncCA9IGdwYXIoZm9udHNpemUgPSAyMikpCgpobWxpc3QzPWhtYXBfaGllcl9mYWN0b3JzNCtobWFwX2hpZXJfZmFjdG9yczRhK2htYXBfaGllcl9mYWN0b3JzNGIraG1hcF9oaWVyX2ZhY3RvcnM2K2htYXBfaGllcl9mYWN0b3JzNmIraG1hcF9oaWVyX2ZhY3RvcnM2YwpkcmF3KGhtbGlzdDMsIGNvbHVtbl90aXRsZSA9ICJIZWF0bWFwcyBvbiBNZWFucyAoc2NhbGVkIHBlciBzdHJhaW4pIGFuZCBsb2dmYyBNZWFucyB2cyBTdHJhaW4gMGgiLCBjb2x1bW5fdGl0bGVfZ3AgPSBncGFyKGZvbnRzaXplID0gMjIpKQpgYGAKCgoKCgoKCmBgYHtyLGZpZy5oZWlnaHQgPSA3LGZpZy53aWR0aCA9MTIgfQpwYXIobWZyb3c9YygxLDIpKQojIyMjIFNpbGhvdWV0dGUgbWV0aG9kCmZ2aXpfbmJjbHVzdChkYXRhSE1tMywga21lYW5zLCBtZXRob2QgPSAic2lsaG91ZXR0ZSIsay5tYXggPSAxNikrCiAgbGFicyhzdWJ0aXRsZSA9ICJTaWxob3VldHRlIG1ldGhvZCIpCgojIyMjIEVsYm93IG1ldGhvZApmdml6X25iY2x1c3QoZGF0YUhNbTMsIGttZWFucywgbWV0aG9kID0gIndzcyIsay5tYXggPSAxNikgKwogIGxhYnMoc3VidGl0bGUgPSAiRWxib3cgbWV0aG9kIikKCmBgYAoKYGBge3J9CiMjIyNnYXAgc3RhdCBzbG93ISEhCiMjIyNzZXQuc2VlZCgxMjMpCiMjIyNmdml6X25iY2x1c3QoZGF0YUhNbSwga21lYW5zLCBuc3RhcnQgPSAyNSwgIG1ldGhvZCA9ICJnYXBfc3RhdCIsIG5ib290ID0gMTAwLGsubWF4ID0gMTYpKwojIyMjICBsYWJzKHN1YnRpdGxlID0gIkdhcCBzdGF0aXN0aWMgbWV0aG9kIikKYGBgCgoKYGBge3IsZmlnLmhlaWdodCA9IDd9CiNrY2x1c3Q4IDwtIGttZWFucyhkYXRhSE1tMywgNCkKI3NpbGhvdWV0dGUgcGxvdApkaXN0SzwtZGFpc3koZGF0YUhNbTMpCnBsb3Qoc2lsaG91ZXR0ZShrY2x1c3Q4JGNsdXN0ZXIsIGRpc3RLKSwgY29sPTE6NCwgYm9yZGVyPU5BKQoKYGBgCgojIyMjMy4gSy1tZWFucyBjbHVzdGVyaW5nIG9mIG1lYW5zIApgYGB7cixmaWcuaGVpZ2h0ID0gMTAsZmlnLndpZHRoID0gMjB9CnNwbGl0IDwtIHBhc3RlMCgiQ2x1c3RlclxuIiwga2NsdXN0OCRjbHVzdGVyKQojc3BsaXQgPC0gZmFjdG9yKHBhc3RlMCgiQ2x1c3RlclxuIiwga2NsdXN0MyRjbHVzdGVyKSwgbGV2ZWxzPWMoIkNsdXN0ZXJcbjMiLCJDbHVzdGVyXG4xIiwiQ2x1c3RlclxuNCIsIkNsdXN0ZXJcbjUiLCJDbHVzdGVyXG4yIiwiQ2x1c3RlclxuNiIpKQpobWFwX2sgPC0gSGVhdG1hcChkYXRhSE1tMywgc3BsaXQ9c3BsaXQsIGNsdXN0ZXJfcm93X3NsaWNlcyA9IEZBTFNFLAogICAgICAgICAgICAgICAgICBjbHVzdGVyX2NvbHVtbnMgPSBGQUxTRSwKICAgICAgICAgICAgICAgICAgc2hvd19yb3dfbmFtZXMgPSBGQUxTRSwKICAgICAgICAgICAgICAgICAgbmFtZSA9ICJNZWFucyAoc2NhbGVkIHBlciBzdHJhaW4iLAogICAgICAgICAgICAgICAgICBjb2wgPSBjb2xfZnVuR1IyLAogICAgICAgICAgICAgICAgICB3aWR0aCA9IHVuaXQoNTAsICJtbSIpLAogICAgICAgICAgICAgICAgICBjb2x1bW5fdGl0bGUgPSAiTWVhbnMiLCAKICAgICAgICAgICAgICAgICAgY29sdW1uX3RpdGxlX2dwID0gZ3Bhcihmb250c2l6ZSA9IDE2LCBmb250ZmFjZSA9ICJib2xkIikpCgpobWFwX2sjK2htYXBfaGllcl9mYWN0b3JzNitobWFwX2hpZXJfZmFjdG9yczUKYGBgCgpNZWFuIHByb2ZpbGVzIG9mIGNsdXN0ZXJzCgpgYGB7cixmaWcuaGVpZ2h0ID0gOSxmaWcud2lkdGggPSA4fQpjbHVzdGVyY291bnQ8LWRhdGEuZnJhbWUoa2NsdXN0OCRjbHVzdGVyKQpjbHVzdGVyc2l6ZXM8LXRhYmxlKGNsdXN0ZXJjb3VudCRrY2x1c3Q4LmNsdXN0ZXIpCmNsdXN0ZXJNZWFuczwtZGF0YS5mcmFtZShrY2x1c3Q4JGNlbnRlcnMpCmNsdXN0ZXJNZWFuczE8LWRhdGEuZnJhbWUodChjbHVzdGVyTWVhbnMpKQpjbHVzdGVyTWVhbnMxIDwtIGNiaW5kKHJvd25hbWVzKGNsdXN0ZXJNZWFuczEpLCBjbHVzdGVyTWVhbnMxKQpvcmRlck48LWMoIlZhcjM3VE5GXzBoX2xmYyIsIlZhcjM3VE5GXzZoX2xmYyIsIlZhcjM3VE5GXzIwaF9sZmMiLCJWYXIxNFRORl8waF9sZmMiLCJWYXIxNFRORl82aF9sZmMiLCJWYXIxNFRORl8yMGhfbGZjIikjIyMjIG1hbnVhbAoKcm93bmFtZXMoY2x1c3Rlck1lYW5zMSkgPC0gTlVMTApuYW1lcyhjbHVzdGVyTWVhbnMxKVtuYW1lcyhjbHVzdGVyTWVhbnMxKT09InJvd25hbWVzKGNsdXN0ZXJNZWFuczEpIl0gPC0gIlNhbXBsZSIKIyMjI2NsdXN0ZXJNZWFuczEKU3RyYWluPC1mYWN0b3IoYyhyZXAoIlZBUjM3IiwzKSxyZXAoIlZBUjE0IiwzKSkpIyMjI25vdGUgbmFtZXMKI3AxPWdncGxvdChkYXRhPWRhdGFIbXQsIGFlcyh4PXJvdy5uYW1lcyhkYXRhSG10KSwgeT1FTlNHMDAwMDAxNjI1NTEuMTQpLGdyb3VwPVJ1bikgKyBnZ3RpdGxlKCJBTFBMIikgK2dlb21fcG9pbnQoKSArICBzY2FsZV94X2Rpc2NyZXRlKGxpbWl0cz1saW1pdHNQbG90KSsgIHlsYWIoeWxhYlBsb3QpK3hsYWIoeGxhYlBsb3QpK2dlb21fbGluZShhZXMoZ3JvdXAgPSBSdW4pKSAKCgpwWDE8LWdncGxvdChkYXRhPWNsdXN0ZXJNZWFuczEsIGFlcyh4PVNhbXBsZSwgeT1YMSxncm91cD0xKSkgKwogIGdlb21fbGluZShhZXMoZ3JvdXAgPSBTdHJhaW4pKSsgIGdlb21fcG9pbnQoKStnZ3RpdGxlKHBhc3RlKCJDbHVzdGVyIFgxIFByb2ZpbGUgIixjbHVzdGVyc2l6ZXNbMV0sIiBnZW5lcyIpKSsgIHNjYWxlX3hfZGlzY3JldGUobGltaXRzPW9yZGVyTikrCiAgdGhlbWUoYXhpcy50aXRsZS54ID0gZWxlbWVudF9ibGFuaygpLGF4aXMudGV4dC54ID0gZWxlbWVudF90ZXh0KGFuZ2xlID0gOTAsIHZqdXN0ID0gMC41LCBoanVzdD0xKSxheGlzLnRpdGxlLnkgPSBlbGVtZW50X2JsYW5rKCkpK3lsaW0gKC0xLjgsMS44KQpwWDI8LWdncGxvdChkYXRhPWNsdXN0ZXJNZWFuczEsIGFlcyh4PVNhbXBsZSwgeT1YMixncm91cD0xKSkgKwogIGdlb21fbGluZShhZXMoZ3JvdXAgPSBTdHJhaW4pKSsgIGdlb21fcG9pbnQoKStnZ3RpdGxlKHBhc3RlKCJDbHVzdGVyIFgyIFByb2ZpbGUgIixjbHVzdGVyc2l6ZXNbMl0sIiBnZW5lcyIpKSsgIHNjYWxlX3hfZGlzY3JldGUobGltaXRzPW9yZGVyTikrCiAgdGhlbWUoYXhpcy50aXRsZS54ID0gZWxlbWVudF9ibGFuaygpLGF4aXMudGV4dC54ID0gZWxlbWVudF90ZXh0KGFuZ2xlID0gOTAsIHZqdXN0ID0gMC41LCBoanVzdD0xKSxheGlzLnRpdGxlLnkgPSBlbGVtZW50X2JsYW5rKCkpK3lsaW0gKC0xLjgsMS44KQpwWDM8LWdncGxvdChkYXRhPWNsdXN0ZXJNZWFuczEsIGFlcyh4PVNhbXBsZSwgeT1YMyxncm91cD0xKSkgKwogIGdlb21fbGluZShhZXMoZ3JvdXAgPSBTdHJhaW4pKSsgIGdlb21fcG9pbnQoKStnZ3RpdGxlKHBhc3RlKCJDbHVzdGVyIFgzIFByb2ZpbGUgIixjbHVzdGVyc2l6ZXNbM10sIiBnZW5lcyIpKSsgIHNjYWxlX3hfZGlzY3JldGUobGltaXRzPW9yZGVyTikrCiAgdGhlbWUoYXhpcy50aXRsZS54ID0gZWxlbWVudF9ibGFuaygpLGF4aXMudGV4dC54ID0gZWxlbWVudF90ZXh0KGFuZ2xlID0gOTAsIHZqdXN0ID0gMC41LCBoanVzdD0xKSxheGlzLnRpdGxlLnkgPSBlbGVtZW50X2JsYW5rKCkpK3lsaW0gKC0xLjgsMS44KQpwWDQ8LWdncGxvdChkYXRhPWNsdXN0ZXJNZWFuczEsIGFlcyh4PVNhbXBsZSwgeT1YNCxncm91cD0xKSkgKwogIGdlb21fbGluZShhZXMoZ3JvdXAgPSBTdHJhaW4pKSsgIGdlb21fcG9pbnQoKStnZ3RpdGxlKHBhc3RlKCJDbHVzdGVyIFg0IFByb2ZpbGUgIixjbHVzdGVyc2l6ZXNbNF0sIiBnZW5lcyIpKSsgIHNjYWxlX3hfZGlzY3JldGUobGltaXRzPW9yZGVyTikrCiAgdGhlbWUoYXhpcy50aXRsZS54ID0gZWxlbWVudF9ibGFuaygpLGF4aXMudGV4dC54ID0gZWxlbWVudF90ZXh0KGFuZ2xlID0gOTAsIHZqdXN0ID0gMC41LCBoanVzdD0xKSxheGlzLnRpdGxlLnkgPSBlbGVtZW50X2JsYW5rKCkpK3lsaW0gKC0xLjgsMS44KQojcFg1PC1nZ3Bsb3QoZGF0YT1jbHVzdGVyTWVhbnMxLCBhZXMoeD1TYW1wbGUsIHk9WDUsZ3JvdXA9MSkpICsKIyAgZ2VvbV9saW5lKGFlcyhncm91cCA9IFN0cmFpbikpKyAgZ2VvbV9wb2ludCgpK2dndGl0bGUocGFzdGUoIkNsdXN0ZXIgWDUgUHJvZmlsZSAiLGNsdXN0ZXJzaXplc1s1XSwiIGdlbmVzIikpKyAgc2NhbGVfeF9kaXNjcmV0ZShsaW1pdHM9b3JkZXJOKSsKIyAgdGhlbWUoYXhpcy50aXRsZS54ID0gZWxlbWVudF9ibGFuaygpLGF4aXMudGV4dC54ID0gZWxlbWVudF90ZXh0KGFuZ2xlID0gOTAsIHZqdXN0ID0gMC41LCBoanVzdD0xKSxheGlzLnRpdGxlLnkgPSBlbGVtZW50X2JsYW5rKCkpK3lsaW0gKC0xLjgsMS44KQojcFg4PC1nZ3Bsb3QoZGF0YT1jbHVzdGVyTWVhbnMxLCBhZXMoeD1TYW1wbGUsIHk9WDgsZ3JvdXA9MSkpICsKIyAgZ2VvbV9saW5lKCkrICBnZW9tX3BvaW50KCkrZ2d0aXRsZShwYXN0ZSgiQ2x1c3RlciBYOCBQcm9maWxlICIsY2x1c3RlcnNpemVzWzZdLCIgZ2VuZXMiKSkrICBzY2FsZV94X2Rpc2NyZXRlKGxpbWl0cz1vcmRlck4pKwojICB0aGVtZShheGlzLnRpdGxlLnggPSBlbGVtZW50X2JsYW5rKCksYXhpcy50aXRsZS55ID0gZWxlbWVudF9ibGFuaygpKQoKCiNwbG90Cm11bHRpcGxvdChwWDEscFgyLHBYNCxwWDMsICAgICBjb2xzPTIpCgpgYGAKCgpgYGB7cn0KdG9wREVnZW5lcyA8LSB3aGljaCh0ZW1wQSRJbmNsdWRlPT0iaW4iKSMjIyNmaW5kIGluZGV4ZXMKdGVtcEFrbTwtdGVtcEFbIHRvcERFZ2VuZXMsIF0KU3ltYm9sc0ttPC1kcGx5cjo6cHVsbCh0ZW1wQWttLCBHZW5lX1N5bWJvbCkKIyMjIyBleHBvcnQgdGhlIGdlbmUgZXhwcmVzc2lvbiBkYXRhIGZvciB0aGUgY2x1c3RlcnMKd3JpdGUudGFibGUoY2x1c3Rlck1lYW5zLHBhc3RlMCgiQ2x1c3Rlck1lYW5zS21fIixncm91cHNOYW1lLCIudHh0IiksICBzZXAgPSAiXHQiKQpDbHVzdGVyZWRHZW5lczwtZGF0YS5mcmFtZShrY2x1c3Q4JGNsdXN0ZXIsU3ltYm9sc0ttLGRhdGFITW0zKQp3cml0ZS50YWJsZShDbHVzdGVyZWRHZW5lcyxwYXN0ZTAoIlNjYWxlZERhdGFJbkNsdXN0ZXJzS21fIixncm91cHNOYW1lLCIudHh0IiksICBzZXAgPSAiXHQiKQojaGVhZChDbHVzdGVyZWRHZW5lcykKYGBgCgoKYGBge3J9CmJvdHRvbURFZ2VuZXM8LXdoaWNoKHRlbXBBJEluY2x1ZGU9PSJvdXQiKSMjIyNmaW5kIGluZGV4ZXMgCmJvdHRvbUc8LXRlbXBBWyBib3R0b21ERWdlbmVzLCBdCmJvdHRvbUc8LWRwbHlyOjpwdWxsKGJvdHRvbUcsIEdlbmVfU3ltYm9sKQp3cml0ZS50YWJsZShib3R0b21HLHBhc3RlMCgiaXBhQm90dG9tS21lYW5zXyIsZ3JvdXBzTmFtZSwiLnR4dCIpLCAgc2VwID0gIlx0IikKICAgICAgICAgICAgICAgICAgICAgICAgIAoKdG9wREVnZW5lcyA8LSB3aGljaCh0ZW1wQSRJbmNsdWRlPT0iaW4iKSMjIyNmaW5kIGluZGV4ZXMgCnRlbXBBa208LXRlbXBBWyB0b3BERWdlbmVzLCBdClN5bWJvbHNLbTwtZHBseXI6OnB1bGwodGVtcEFrbSwgR2VuZV9TeW1ib2wpCgppcGFLbWVhbnM8LUNsdXN0ZXJlZEdlbmVzCiNjb3VudHNUYWJsZSA8LWNvdW50c1RhYmxlWyxjKDE6MTUpXSMjIyNpZiBzYW1wbGVzIG5lZWQgcmVtb3ZpbmcKaXBhS21lYW5zPC1pcGFLbWVhbnNbLGMoMToyKV0KaXBhS21lYW5zJG5hbWUyPC1yb3duYW1lcyhpcGFLbWVhbnMpCiNpcGFLbWVhbnMlPiUgcm93bmFtZXNfdG9fY29sdW1uKHZhciA9ICJyb3duYW1lIikKI2lwYUttZWFucwojcm93aWRfdG9fY29sdW1uKGlwYUttZWFucykKaXBhS21lYW5zID0gbXV0YXRlKGlwYUttZWFucywgeDE9IGlmZWxzZShpcGFLbWVhbnMka2NsdXN0OC5jbHVzdGVyPT0xLCAiMSIsICIwIikpCmlwYUttZWFucyA9IG11dGF0ZShpcGFLbWVhbnMsIHgyPSBpZmVsc2UoaXBhS21lYW5zJGtjbHVzdDguY2x1c3Rlcj09MiwgIjEiLCAiMCIpKQppcGFLbWVhbnMgPSBtdXRhdGUoaXBhS21lYW5zLCB4Mz0gaWZlbHNlKGlwYUttZWFucyRrY2x1c3Q4LmNsdXN0ZXI9PTMsICIxIiwgIjAiKSkKaXBhS21lYW5zID0gbXV0YXRlKGlwYUttZWFucywgeDQ9IGlmZWxzZShpcGFLbWVhbnMka2NsdXN0OC5jbHVzdGVyPT00LCAiMSIsICIwIikpCiNpcGFLbWVhbnMgPSBtdXRhdGUoaXBhS21lYW5zLCB4NT0gaWZlbHNlKGlwYUttZWFucyRrY2x1c3QzLmNsdXN0ZXI9PTUsICIxIiwgIjAiKSkKI2lwYUttZWFucyA9IG11dGF0ZShpcGFLbWVhbnMsIHg2PSBpZmVsc2UoaXBhS21lYW5zJGtjbHVzdDMuY2x1c3Rlcj09NiwgIjEiLCAiMCIpKQojaXBhS21lYW5zID0gbXV0YXRlKGlwYUttZWFucywgeDc9IGlmZWxzZShpcGFLbWVhbnMka2NsdXN0My5jbHVzdGVyPT03LCAiMSIsICIwIikpCiNpcGFLbWVhbnMKd3JpdGUudGFibGUoaXBhS21lYW5zLHBhc3RlMCgiaXBhS21lYW5zXyIsZ3JvdXBzTmFtZSwiLnR4dCIpLCAgc2VwID0gIlx0IikKI2hlYWQoaXBhS21lYW5zKQoKYGBgCgoKYGBge3J9CkNsdXN0ZXJlZEdlbmVzMjwtQ2x1c3RlcmVkR2VuZXNbYygxKV0KI0NsdXN0ZXJlZEdlbmVzMgpsaXN0QWxsPC1saXN0KCkKZm9yKGkgaW4gMTo0KSB7CiAgY2x1c3Rlck5hbWU8LXBhc3RlMCgieCIsaSkKICAjY2x1c3Rlck5hbWU8LXJvdy5uYW1lcyhzdWJzZXQoQ2x1c3RlcmVkR2VuZXMsQ2x1c3RlcmVkR2VuZXM9PWkpKQogIGNsdXN0ZXJOYW1lPC0oc3Vic2V0KENsdXN0ZXJlZEdlbmVzJFN5bWJvbHNLbSxDbHVzdGVyZWRHZW5lcz09aSkpCiAgbGlzdEFsbFtbaV1dPC1jbHVzdGVyTmFtZQp9CiNuZWVkIHRvIG5hbWUgdGhlIHZlY3RvcnMgaW4gdGhlIGxpc3QsIGV4YW1wbGUgaGVyZSBpcyBmb3IgOCBjbHVzdGVycwpuYW1lcyhsaXN0QWxsKTwtYygiWDEiLCAiWDIiLCAiWDMiLCAiWDQiKSMsIlg1IiwgIlg2IiwgIlg3IikKCiNpZiB5b3Ugd2FudCB0byByZWFycmFuZ2UgdGhlIG9yZGVyCiNsaXN0QWxsPC1saXN0QWxsW2MoIngzIiwgIng3IiwgIng4IiwgIngyIiwgIng2IiwgIng1IiwgIng0IiwgIngxIildCgojbGFwcGx5KGxpc3RBbGwsIGhlYWQpCgpgYGAKCiMjIyM0LiBBbm5vdGF0aW9uIG9mIEstbWVhbnMgY2x1c3RlcnMKLSBDQyBjZWxsdWxhciBjb21wYXJ0bWVudAotIEJQIGJpb2xvZ2ljYWwgcHJvY2VzcwotIE1GIG1vbGVjdWxhciBmdW5jdGlvbgoKVGhlIHNpbXBsaWZ5IGZ1bmN0aW9uIGhhcyBiZWVuIHVzZWQgdG8gY3V0IGRvd24gb24gR08gcmVkdW5kYW5jeQoKYGBge3J9CiNzdHIoQWxsR2VuZU5hbWVzKQpgYGAKCmBgYHtyfQp4cmVhZApgYGAKCmBgYHtyLGZpZy5oZWlnaHQgPSA4LGZpZy53aWR0aCA9IDEyfQojIyMjQ0MKY2dvQ0MgPC0gY29tcGFyZUNsdXN0ZXIoZ2VuZUNsdXN0ZXIgPSBsaXN0QWxsLCAKICAgICAgICAgICAgICAgICAgICAgIHVuaXZlcnNlID0gQWxsR2VuZU5hbWVzLAogICAgICAgICAgICAgICAgICAgICAgZnVuID0gImVucmljaEdPIiwKICAgICAgICAgICAgICAgICAgICAgIE9yZ0RiPW9yZy5Icy5lZy5kYiwgCiAgICAgICAgICAgICAgICAgICAgICAjIyMjT3JnRGI9b3JnLk1tLmVnLmRiLAogICAgICAgICAgICAgICAgICAgICAga2V5VHlwZT0iU1lNQk9MIiwKICAgICAgICAgICAgICAgICAgICAgIG9udCA9ICJDQyIsIAogICAgICAgICAgICAgICAgICAgICAgcHZhbHVlQ3V0b2ZmPTAuMDUsCiAgICAgICAgICAgICAgICAgICAgICBxdmFsdWVDdXRvZmYgPSAwLjEwKQpjZ29DQzIgPC0gc2ltcGxpZnkoY2dvQ0MsIGN1dG9mZj0wLjcsIGJ5PSJwLmFkanVzdCIsIHNlbGVjdF9mdW49bWluKQojIyMjd3JpdGUgYXMgc3ByZWFkc2hlZXQKd3JpdGUuY3N2KGFzLmRhdGEuZnJhbWUoY2dvQ0MyKSxwYXN0ZTAoIkdPX0NDXyIsZ3JvdXBzTmFtZSwiLmNzdiIpKQoKZG90cGxvdChjZ29DQzIsc2hvd0NhdGVnb3J5ID0gMzAsCiAgICAgICAgdGl0bGUgPSBwYXN0ZTAoIkdPIENlbGx1bGFyIENvbXBhcnRtZW50ICIsZ3JvdXBzTmFtZSkpKwogIHRoZW1lKGF4aXMudGV4dC54ID0gZWxlbWVudF90ZXh0KGFuZ2xlID0gOTAsIHZqdXN0ID0gMC41LCBoanVzdD0xKSkKCmBgYApQbG90cyBhbmQgR08gZGF0YSB3ZXJlIHdyaXR0ZW4gdG8gZmlsZXMKYGBge3J9CnBuZyhwYXN0ZTAoIkdPX0NDXyIsZ3JvdXBzTmFtZSwiLnBuZyIpLCB3aWR0aCA9IDEyMjQsIGhlaWdodCA9IDgyNCkKZG90cGxvdChjZ29DQzIsc2hvd0NhdGVnb3J5ID0gMzAsCiAgICAgICAgdGl0bGUgPSBwYXN0ZTAoIkdPIENlbGx1bGFyIENvbXBhcnRtZW50ICIsZ3JvdXBzTmFtZSkpKwogIHRoZW1lKGF4aXMudGV4dC54ID0gZWxlbWVudF90ZXh0KGFuZ2xlID0gOTAsIHZqdXN0ID0gMC41LCBoanVzdD0xKSkKZGV2Lm9mZigpCmBgYAoKR08gQlAKYGBge3IsZmlnLmhlaWdodCA9IDEyLGZpZy53aWR0aCA9IDEyfQojIyMjQ0MKY2dvQlAgPC0gY29tcGFyZUNsdXN0ZXIoZ2VuZUNsdXN0ZXIgPSBsaXN0QWxsLCAKICAgICAgICAgICAgICAgICAgICAgIHVuaXZlcnNlID0gQWxsR2VuZU5hbWVzLAogICAgICAgICAgICAgICAgICAgICAgZnVuID0gImVucmljaEdPIiwKICAgICAgICAgICAgICAgICAgICAgIE9yZ0RiPW9yZy5Icy5lZy5kYiwKICAgICAgICAgICAgICAgICAgICAgIGtleVR5cGU9IlNZTUJPTCIsCiAgICAgICAgICAgICAgICAgICAgICBvbnQgPSAiQlAiLCAKICAgICAgICAgICAgICAgICAgICAgIHB2YWx1ZUN1dG9mZj0wLjA1LAogICAgICAgICAgICAgICAgICAgICAgcXZhbHVlQ3V0b2ZmID0gMC4xMCkKY2dvQlAyIDwtIHNpbXBsaWZ5KGNnb0JQLCBjdXRvZmY9MC43LCBieT0icC5hZGp1c3QiLCBzZWxlY3RfZnVuPW1pbikKIyMjI3dyaXRlIGFzIHNwcmVhZHNoZWV0CndyaXRlLmNzdihhcy5kYXRhLmZyYW1lKGNnb0JQMikscGFzdGUwKCJHT19CUF8iLGdyb3Vwc05hbWUsIi5jc3YiKSkKCmRvdHBsb3QoY2dvQlAyLHNob3dDYXRlZ29yeSA9IDMwLAogICAgICAgIHRpdGxlID0gcGFzdGUwKCJHTyBCaW9sb2dpY2FsIFByb2Nlc3MgIixncm91cHNOYW1lKSkrCiAgdGhlbWUoYXhpcy50ZXh0LnggPSBlbGVtZW50X3RleHQoYW5nbGUgPSA5MCwgdmp1c3QgPSAwLjUsIGhqdXN0PTEpKQoKYGBgCgpgYGB7cn0KcG5nKHBhc3RlMCgiR09fQlBfIixncm91cHNOYW1lLCIucG5nIiksIHdpZHRoID0gMTAyNCwgaGVpZ2h0ID0gMTIyNCkKZG90cGxvdChjZ29CUDIsc2hvd0NhdGVnb3J5ID0gMzAsCiAgICAgICAgdGl0bGUgPSBwYXN0ZTAoIkdPIEJpb2xvZ2ljYWwgUHJvY2VzcyAiLGdyb3Vwc05hbWUpKSsKICB0aGVtZShheGlzLnRleHQueCA9IGVsZW1lbnRfdGV4dChhbmdsZSA9IDkwLCB2anVzdCA9IDAuNSwgaGp1c3Q9MSkpCmRldi5vZmYoKQpgYGAKCgpHTyBNRgpgYGB7cixmaWcuaGVpZ2h0ID0gMTAsZmlnLndpZHRoID0gMTJ9CiMjIyNNRgpjZ29NRiA8LSBjb21wYXJlQ2x1c3RlcihnZW5lQ2x1c3RlciA9IGxpc3RBbGwsIAogICAgICAgICAgICAgICAgICAgICAgdW5pdmVyc2UgPSBBbGxHZW5lTmFtZXMsCiAgICAgICAgICAgICAgICAgICAgICBmdW4gPSAiZW5yaWNoR08iLAogICAgICAgICAgICAgICAgICAgICAgT3JnRGI9b3JnLkhzLmVnLmRiLCAKICAgICAgICAgICAgICAgICAgICAgIGtleVR5cGU9IlNZTUJPTCIsCiAgICAgICAgICAgICAgICAgICAgICBvbnQgPSAiTUYiLCAKICAgICAgICAgICAgICAgICAgICAgIHB2YWx1ZUN1dG9mZj0wLjA1LAogICAgICAgICAgICAgICAgICAgICAgcXZhbHVlQ3V0b2ZmID0gMC4xMCkKY2dvTUYyIDwtIHNpbXBsaWZ5KGNnb01GLCBjdXRvZmY9MC43LCBieT0icC5hZGp1c3QiLCBzZWxlY3RfZnVuPW1pbikKIyMjI3dyaXRlIGFzIHNwcmVhZHNoZWV0CndyaXRlLmNzdihhcy5kYXRhLmZyYW1lKGNnb01GMikscGFzdGUwKCJHT19NRl8iLGdyb3Vwc05hbWUsIi5jc3YiKSkKCmRvdHBsb3QoY2dvTUYyLHNob3dDYXRlZ29yeSA9IDMwLAogICAgICAgIHRpdGxlID0gcGFzdGUwKCJHTyBNb2xlY3VsYXIgRnVuY3Rpb24gICIsZ3JvdXBzTmFtZSkpKwogIHRoZW1lKGF4aXMudGV4dC54ID0gZWxlbWVudF90ZXh0KGFuZ2xlID0gOTAsIHZqdXN0ID0gMC41LCBoanVzdD0xKSkKCmBgYAoKCgpgYGB7cn0KcG5nKHBhc3RlMCgiR09fTUZfIixncm91cHNOYW1lLCIucG5nIiksIHdpZHRoID0gMTQyNCwgaGVpZ2h0ID0gODI0KQpkb3RwbG90KGNnb01GMixzaG93Q2F0ZWdvcnkgPSAzMCwKICAgICAgICB0aXRsZSA9IHBhc3RlMCgiR08gTW9sZWN1bGFyIEZ1bmN0aW9uICAiLGdyb3Vwc05hbWUpKSsKICB0aGVtZShheGlzLnRleHQueCA9IGVsZW1lbnRfdGV4dChhbmdsZSA9IDkwLCB2anVzdCA9IDAuNSwgaGp1c3Q9MSkpCmRldi5vZmYoKQpgYGAKCmBgYHtyLGZpZy5oZWlnaHQgPSA3LGZpZy53aWR0aCA9IDEyfQojZGJzIDwtIGMoIkdPX01vbGVjdWxhcl9GdW5jdGlvbl8yMDE4IiwgIkdPX0NlbGx1bGFyX0NvbXBvbmVudF8yMDE4IiwgIkdPX0Jpb2xvZ2ljYWxfUHJvY2Vzc18yMDE4IikKZGJzIDwtIGMoIlJlYWN0b21lXzIwMTYiLCJXaWtpUGF0aHdheXNfMjAxOV9Nb3VzZSIpCgppZiAod2Vic2l0ZUxpdmUpIHsgICAgZW5yaWNoZWQxIDwtIGVucmljaHIoKHN1YnNldChDbHVzdGVyZWRHZW5lcyRTeW1ib2xzS20sQ2x1c3RlcmVkR2VuZXM9PTEpKSwgZGJzKX0KaWYgKHdlYnNpdGVMaXZlKSBwbG90RW5yaWNoKGVucmljaGVkMVtbMV1dLCBzaG93VGVybXMgPSAzMCwgbnVtQ2hhciA9IDgwLCB5ID0gIkNvdW50Iiwgb3JkZXJCeSA9ICJQLnZhbHVlIiwgdGl0bGUgPSJSZWFjdG9tZSBFbnJpY2htZW50IEFuYWx5c2lzIENsdXN0ZXIgMSIpCmlmICh3ZWJzaXRlTGl2ZSkgeyAgICBlbnJpY2hlZDIgPC0gZW5yaWNocigoc3Vic2V0KENsdXN0ZXJlZEdlbmVzJFN5bWJvbHNLbSxDbHVzdGVyZWRHZW5lcz09MikpLCBkYnMpfQppZiAod2Vic2l0ZUxpdmUpIHBsb3RFbnJpY2goZW5yaWNoZWQyW1sxXV0sIHNob3dUZXJtcyA9IDMwLCBudW1DaGFyID0gODAsIHkgPSAiQ291bnQiLCBvcmRlckJ5ID0gIlAudmFsdWUiLCB0aXRsZSA9IlJlYWN0b21lIEVucmljaG1lbnQgQW5hbHlzaXMgQ2x1c3RlciAyIikKaWYgKHdlYnNpdGVMaXZlKSB7ICAgIGVucmljaGVkMyA8LSBlbnJpY2hyKChzdWJzZXQoQ2x1c3RlcmVkR2VuZXMkU3ltYm9sc0ttLENsdXN0ZXJlZEdlbmVzPT0zKSksIGRicyl9CmlmICh3ZWJzaXRlTGl2ZSkgcGxvdEVucmljaChlbnJpY2hlZDNbWzFdXSwgc2hvd1Rlcm1zID0gMzAsIG51bUNoYXIgPSA4MCwgeSA9ICJDb3VudCIsIG9yZGVyQnkgPSAiUC52YWx1ZSIsIHRpdGxlID0iUmVhY3RvbWUgRW5yaWNobWVudCBBbmFseXNpcyBDbHVzdGVyIDMiKQppZiAod2Vic2l0ZUxpdmUpIHsgICAgZW5yaWNoZWQ0IDwtIGVucmljaHIoKHN1YnNldChDbHVzdGVyZWRHZW5lcyRTeW1ib2xzS20sQ2x1c3RlcmVkR2VuZXM9PTQpKSwgZGJzKX0KaWYgKHdlYnNpdGVMaXZlKSBwbG90RW5yaWNoKGVucmljaGVkNFtbMV1dLCBzaG93VGVybXMgPSAzMCwgbnVtQ2hhciA9IDgwLCB5ID0gIkNvdW50Iiwgb3JkZXJCeSA9ICJQLnZhbHVlIiwgdGl0bGUgPSJSZWFjdG9tZSBFbnJpY2htZW50IEFuYWx5c2lzIENsdXN0ZXIgNCIpCgppZiAod2Vic2l0ZUxpdmUpIHBsb3RFbnJpY2goZW5yaWNoZWQxW1syXV0sIHNob3dUZXJtcyA9IDMwLCBudW1DaGFyID0gODAsIHkgPSAiQ291bnQiLCBvcmRlckJ5ID0gIlAudmFsdWUiLCB0aXRsZSA9Ildpa2lQYXRod2F5cyBFbnJpY2htZW50IEFuYWx5c2lzIENsdXN0ZXIgMSIpCmlmICh3ZWJzaXRlTGl2ZSkgcGxvdEVucmljaChlbnJpY2hlZDJbWzJdXSwgc2hvd1Rlcm1zID0gMzAsIG51bUNoYXIgPSA4MCwgeSA9ICJDb3VudCIsIG9yZGVyQnkgPSAiUC52YWx1ZSIsIHRpdGxlID0iV2lraVBhdGh3YXlzIEVucmljaG1lbnQgQW5hbHlzaXMgQ2x1c3RlciAyIikKaWYgKHdlYnNpdGVMaXZlKSBwbG90RW5yaWNoKGVucmljaGVkM1tbMl1dLCBzaG93VGVybXMgPSAzMCwgbnVtQ2hhciA9IDgwLCB5ID0gIkNvdW50Iiwgb3JkZXJCeSA9ICJQLnZhbHVlIiwgdGl0bGUgPSJXaWtpUGF0aHdheXMgRW5yaWNobWVudCBBbmFseXNpcyBDbHVzdGVyIDMiKQppZiAod2Vic2l0ZUxpdmUpIHBsb3RFbnJpY2goZW5yaWNoZWQ0W1syXV0sIHNob3dUZXJtcyA9IDMwLCBudW1DaGFyID0gODAsIHkgPSAiQ291bnQiLCBvcmRlckJ5ID0gIlAudmFsdWUiLCB0aXRsZSA9Ildpa2lQYXRod2F5cyBFbnJpY2htZW50IEFuYWx5c2lzIENsdXN0ZXIgNCIpCmBgYAoKCgpzYXZlOiBvbmNlIGhhcHB5IHdpdGggY2x1c3RlcmluZyBzYXZlIHdvcmtzcGFjZSBzbyB0aGF0IGl0IGNhbiBiZSByZWNhbGxlZApgYGB7cn0Kc2F2ZS5pbWFnZShmaWxlPSJLbUZlYjIwMjEuUkRhdGEiKQpgYGAKCgoKCkFkZCBhIG5ldyBjaHVuayBieSBjbGlja2luZyB0aGUgKkluc2VydCBDaHVuayogYnV0dG9uIG9uIHRoZSB0b29sYmFyIG9yIGJ5IHByZXNzaW5nICpDdHJsK0FsdCtJKi4KCldoZW4geW91IHNhdmUgdGhlIG5vdGVib29rLCBhbiBIVE1MIGZpbGUgY29udGFpbmluZyB0aGUgY29kZSBhbmQgb3V0cHV0IHdpbGwgYmUgc2F2ZWQgYWxvbmdzaWRlIGl0IChjbGljayB0aGUgKlByZXZpZXcqIGJ1dHRvbiBvciBwcmVzcyAqQ3RybCtTaGlmdCtLKiB0byBwcmV2aWV3IHRoZSBIVE1MIGZpbGUpLgoKVGhlIHByZXZpZXcgc2hvd3MgeW91IGEgcmVuZGVyZWQgSFRNTCBjb3B5IG9mIHRoZSBjb250ZW50cyBvZiB0aGUgZWRpdG9yLiBDb25zZXF1ZW50bHksIHVubGlrZSAqS25pdCosICpQcmV2aWV3KiBkb2VzIG5vdCBydW4gYW55IFIgY29kZSBjaHVua3MuIEluc3RlYWQsIHRoZSBvdXRwdXQgb2YgdGhlIGNodW5rIHdoZW4gaXQgd2FzIGxhc3QgcnVuIGluIHRoZSBlZGl0b3IgaXMgZGlzcGxheWVkLgo=
